# Supplementary material for: Durvalumab plus tremelimumab for the treatment of advanced neuroendocrine neoplasms of gastroenteropancreatic and lung origin
Source: Nat Commun. 2023 May 23;14:2973. doi: 10.1038/s41467-023-38611-5 (PMC10204675; doi:10.1038/s41467-023-38611-5)
Supplement: Supplementary file 1 — Supplementary Information [file 41467_2023_38611_MOESM1_ESM.pdf]

SUPPLEMENTARY INFORMATION

Supplementary Figure 1. Tumor size changes by RECIST 1.1. Spider plot (left) showing changes in tumor size from baseline for each patient. Patients having as their best response a complete response are shown in dark green, those with a partial response in light green, those with stable disease in yellow, and those with progression disease in red. Swimmer plot (right) showing the first date of SD or response, progression, or exitus. A) Typical / atypical lung carcinoids, n = 27; B) low grade (Grade 1-2) gastrointestinal NENs, n = 31; C) low grade (Grade 1-2) pancreatic NENs, n = 32; D) High grade (grade 3) gastroenteropancreatic NENs, n = 33.

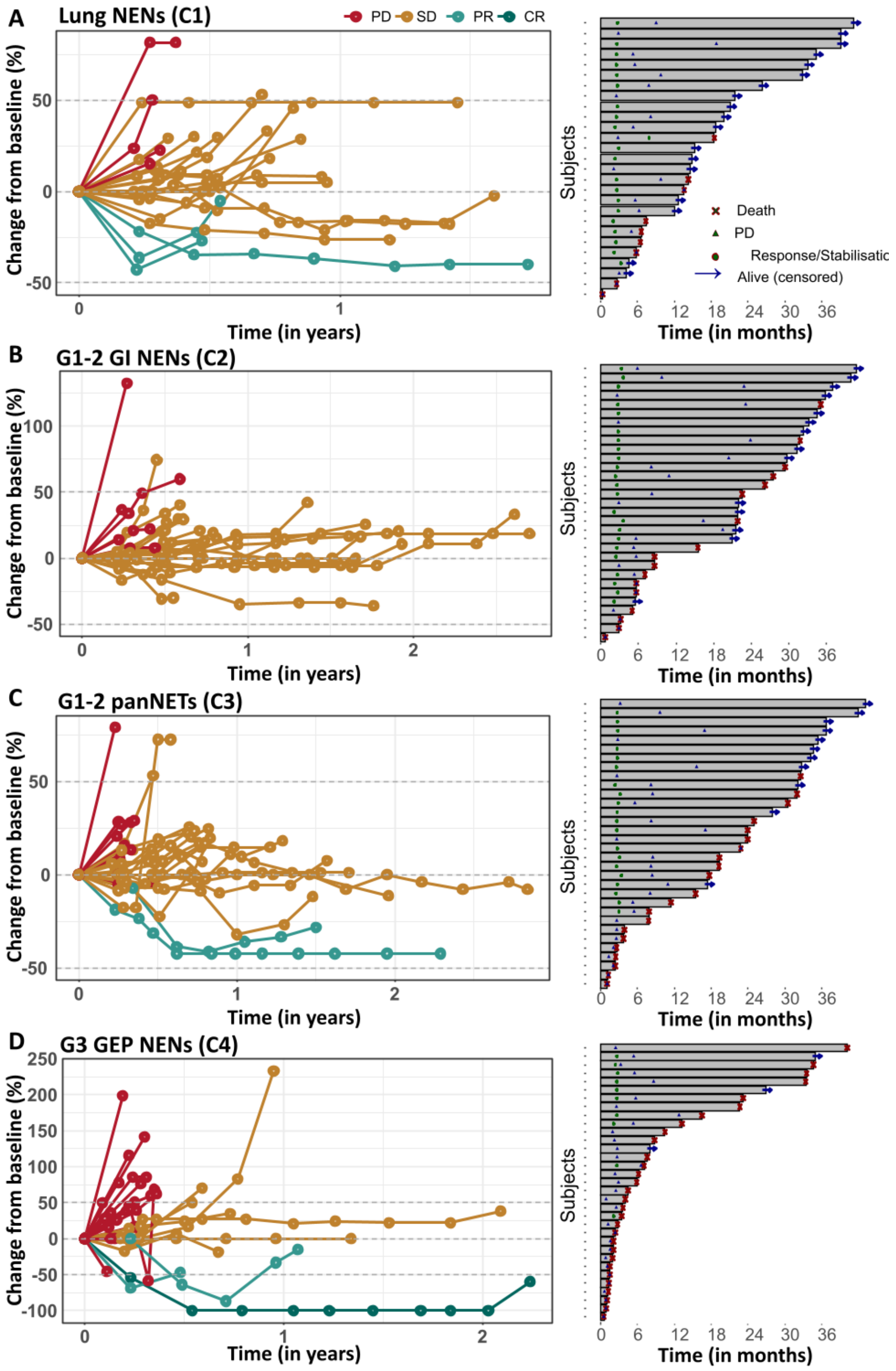

Supplementary Table 1. Baseline patient characteristics

Characteristic	Lung-NENs (N = 27)	G1/2 GI-NETs (N = 31)	G1/2 pan-NETs (N = 32)	G3 GEP-NENs (N = 33)	DUNE trial (N = 123)
Median age (range); years	62 (34-83)	63 (38-84)	66 (47-86)	55 (34-78)	62 (34-86)
Sex; n (%)					
Male	18 (66.7)	18 (58.1)	14 (43.8)	22 (66.7)	72 (58.5)
Female	9 (33.3)	13 (41.9)	18 (56.3)	11 (33.3)	51 (41.5)
ECOG PS; n (%)					
score 0	11 (40.7)	13 (41.9)	16 (50)	13 (39.4)	53 (43.1)
score 1	16 (59.3)	18 (58.1)	16 (50)	20 (60.6)	70 (56.9)
Histopathological diagnosis (central review); n (%) [a]					
well differentiated / Typical / NET [a]	21 (77.8)	28 (90.3)	29 (90.6)	15 (45.5)	93 (75.6)
moderately differentiated / Atypical /NET [a]	6 (22.2)	3 (9.7)	3 (9.4)	0 (0)	12 (9.8)
poorly differentiated /NEC [a]	0 (0)	0 (0)	0 (0)	18 (54.5)	18 (14.6)
Histological diagnosis, cell type; n (%)					
Large Cell	-	-	-	3 (9.1)	-
Small Cell	-	-	-	4 (12.1)	-
uk	-	-	-	11 (33.3)	-
NA (i.e. NETs)	-	-	-	15 (45.5)	-
Primary tumour location; n (%)					
Oesophagus	-	0 (0)	-	1 (3)	-
Gastric	-	1 (3.2)	-	3 (9.1)	-
Gallbladder	-	0 (0)	-	1 (3)	-
Pancreas	-	0 (0)	-	14 (42.2)	-
Small intestine	-	28 (90.3)	-	1 (3)	-
Colorectal	-	2 (6.5)	-	6 (18.2)	-
Digestive NOS	-	0 (0)	-	3 (9.1)	-
UK	-	0 (0)	-	4 (12.1)	-
Clinical stage, n (%)					

I - III	8 (29.6)	1 (3.2)	1 (3.1)	3 (9.1)	13 (10.6)
IV	18 (66.7)	30 (96.8)	31 (96.9)	30 (90.9)	109 (88.6)
Uk	1 (3.7)	0 (0)	0 (0)	0 (0)	1 (0.8)
Ki-67; n (%)					
< 2	1 (3.7)	6 (19.4)	1 (3.1)	0 (0)	8 (6.5)
2-20	19 (70.4)	25 (80.6)	31 (96.9)	0 (0)	75 (61)
> 20	5 (18.5)	0 (0)	0 (0)	33 (100)*	38 (30.9)
Uk	2 (7.4)	0 (0)	0 (0)	0 (0)	2 (1.6)
Mitotic index HPF; n (%)					
< 2	9 (33.3)	13 (52.0)	12 (50.0)	1 (7.1)	26 (41.3)
2-20	16 (59.4)	11 (35.5)	12 (38.7)	5 (16.1)	28 (90.3)
> 20	-	1 (3.2)	0 (0.0)	8 (25.8)	9 (29.0)
Uk	2 (7.4)	6 (19.4)	8 (25.0)	19 (57.6)	60 (48.8)
PD-L1 Combined Positive Score (central review); n (%)					
Negative (0 - 1)	13 (48.1)	22 (71)	16 (50)	14 (42.4)	65 (52.8)
Positive (> 1)	6 (22.2)	3 (9.7)	5 (15.6)	7 (21.2)	21 (17.1)
Uk	8 (29.6)	6 (19.4)	11 (34.4)	12 (36.4)	37 (30.1)
Previous lines; n (%)					
1	15 (55.6)	12 (38.7)	1 (3.1)	33 (100)	61 (49.6)
2	10 (37)	12 (38.7)	14 (43.8)	0 (0)	36 (29.3)
≥ 3	2 (7.4)	7 (22.6)	17 (53.1)	0 (0)	26 (21.1)
Clinical stage, n (%)					
I - III	8 (29.6)	1 (3.2)	1 (3.1)	3 (9.1)	13 (10.6)
IV	18 (66.7)	30 (96.8)	31 (96.9)	30 (90.9)	109 (88.6)
Uk	1 (3.7)	0 (0)	0 (0)	0 (0)	1 (0.8)
Ki-67; n (%)					
< 2	1 (3.7)	6 (19.4)	1 (3.1)	0 (0)	8 (6.5)
2-20	19 (70.4)	25 (80.6)	31 (96.9)	0 (0)	75 (61)
> 20	5 (18.5)	0 (0)	0 (0)	33 (100)*	38 (30.9)
Uk	2 (7.4)	0 (0)	0 (0)	0 (0)	2 (1.6)

Mitotic index HPF; n (%)					
< 2	9 (33.3)	13 (52.0)	12 (50.0)	1 (7.1)	26 (41.3)
2-20	16 (59.4)	11 (35.5)	12 (38.7)	5 (16.1)	28 (90.3)
> 20	-	1 (3.2)	0 (0.0)	8 (25.8)	9 (29.0)
Uk	2 (7.4)	6 (19.4)	8 (25.0)	19 (57.6)	60 (48.8)
PD-L1 Combined Positive Score (central review); n (%)					
Negative (0 - 1)	13 (48.1)	22 (71)	16 (50)	14 (42.4)	65 (52.8)
Positive (> 1)	6 (22.2)	3 (9.7)	5 (15.6)	7 (21.2)	21 (17.1)
Uk	8 (29.6)	6 (19.4)	11 (34.4)	12 (36.4)	37 (30.1)
Previous lines; n (%)					
1	15 (55.6)	12 (38.7)	1 (3.1)	33 (100)	61 (49.6)
2	10 (37)	12 (38.7)	14 (43.8)	0 (0)	36 (29.3)
≥ 3	2 (7.4)	7 (22.6)	17 (53.1)	0 (0)	26 (21.1)
Type of previous lines, n (%)					
Chemotherapy (CT)	5 (18.5)	7 (22.6)	16 (50)	0 (0)	28 (22.8)
Platinum-based CT	0 (0)	0 (0)	0 (0)	33 (100)	33 (26.8)
SSA	22 (81.5)	29 (93.5)	25 (78.1)	2 (6.1)	78 (63.4)
Everolimus	17 (63)	19 (61.3)	25 (78.1)	0 (0)	61 (49.6)
Sunitinib	0 (0)	2 (10.5)	18 (56.3)	0 (0)	20 (16.3)
Radiopharmaceuticals	0 (0)	6 (19.4)	3 (9.4)	0 (0)	9 (7.3)
others	11 (40.1)	17 (54.8)	20 (62.5)	5 (15.6)	53 (43.1)

* In high grade GEP-NENs 20 (60.6%) patients had Ki-67 > 50.
[a] Well / moderately / poorly differentiated categories apply to cohorts 1-3 (low grade NENs). Typical / atypical categories apply to cohort 1 (Lung NENs). NET / NEC categories apply to cohort 4 (G3 GEP NENs).

Abbreviations: ECOG PS, Eastern Cooperative Oncology Group performance status; SSA, Somatostatin analogues; CT, Chemotherapy; PD-L1, Programmed Death ligand 1; GEP, Gastroenteropancreatic; GI, gastrointestinal; NECs, neuroendocrine carcinomas; NENs, neuroendocrine neoplasms; NETs, neuroendocrine tumors; pan, pancreatic; Uk, unknown; NOS, not otherwise specified.

Supplementary Table 2. Durvalumab plus Tremelimumab activity

		Lung-NENs (N = 27)*	G1/2 GI-NETs (N = 31)*	G1/2 pan-NETs (N = 32)*	G3 GEP-NENs (N = 33)*
**RECIST 1.1**	Best Overall response, n (%)				
	CR	0 (0)	0 (0)	0 (0)	1 (3)
	PR	3 (11.1)	0 (0)	2 (6.3)	2 (6.1)
	SD	15 (55.6)	23 (74.2)	17 (53.1)	6 (18.2)
	PD	7 (25.9)	6 (19.4)	10 (31.3)	20 (60.6)
	ORR; % (95% CI)	11.1 (3.2-26.8)	0 (0)	6.3 (1.3-18.6)	9.1 (2.6-22.3)
	CBR; % (95% CI)	66.7 (47.9-82.1)	74.2 (57.1-87.0)	59.4 (42.2-75.0)	27.3 (14.4-43.9)
	CBR 9m Rate; % (95% CI)	25.9 (12.4-44.3)	35.5 (20.5-53.0)	25 (12.6-41.7)	6.1 (1.3-18.1)
**ir RECIST 1.1**	Best Overall response, n (%)				
	CR	0 (0)	0 (0)	0 (0)	1 (3)
	PR	3 (11.1)	0 (0)	2 (6.3)	2 (6.1)
	SD	18 (66.7)	23 (74.2)	18 (56.3)	7 (21.2)
	PD	6 (22.2)	8 (25.8)	12 (37.5)	23 (69.7)
	ORR; % (95% CI)	11.1 (3.2-26.8)	0 (0)	6.3 (1.3-18.6)	9.1 (2.6-22.3)
	CBR; % (95% CI)	77.8 (59.8-90.2)	74.2 (57.1-87.0)	62.5 (45.2-77.6)	30.3 (16.8-47.1)
	CBR 9m Rate; % (95% CI)	25.9 (12.4-44.3)	35.5 (20.5-53.0)	25 (12.6-41.7)	6.1 (1.3-18.1)
DoR; median (range); m		3 (2.7 -15.5)	-	16.3 (10.6-22)	10.1 (3.9-24.3)
p Value **(RECIST 1.1 vs irRECIST 1.1)[1]**		<0.001	<0.001	<0.001	<0.001

1 Cohen's kappa test comparing the distribution of best objective responses within each cohort and in the full dataset. The test measures the agreement between both methods, A significant p-value (<0.05) means that the agreement is significant and both methods are equivalent at the statistical level.
* Two patients in C1 and C2, three patients in C3, and four patients in C4 were only evaluated by irRECIST and not by RECIST. Abbreviations: ORR, Objective Response Rate; CBR, Clinical Benfit Rate; DoR, Duration of the Response; CR, Complete Response; PR, Partial Response; SD, Stable Disease; PD, Progression disease; RECIST, Response evaluation criteria in solid tumors; GEP, Gastroenteropancreatic; GI, gastrointestinal; NECs, neuroendocrine carcinomas; NENs, neuroendocrine neoplasms; NETs, neuroendocrine tumors; pan, pancreatic.

Supplementary Table 3. ORR vs PD-L1

		Lung-NENs (N = 27)		G1/2 GI-NETs (N = 31)		G1/2 pan-NETs (N = 32)		G3 GEP-NENs (N = 33)	
**PD-L1 CPS**		Negative	Positive	Negative	Positive	Negative	Positive	Negative	Positive
**ORR n (%)**	**Yes**	0 (0)	2 (33.3)	0 (0)	0 (0)	0 (0)	1 (20)	1 (7.1)	0 (0)
	**No**	13 (100)	4 (66.7)	22 (100)	3 (100)	16 (100)	4 (80)	13 (92.9)	7 (100)
p-value [1]		0.088		-		0.238		1.000	

ORR: calculated as the best response during treatment by irRECIST 1.1; PD-L1 CPS assessed by centralized review. 1: Fisher Exact test

Supplementary Table 4. OS vs PD-L1

Characteristic	No long-survivors (OS <12m) (N = 23)	Long-survivors (OS >12m) (N = 10)	*p-value*
Median age (range); years	55 (34-78)	57 (42-75)	0.875 [1]
Sex; n (%)			
Male	14 (60.9)	8 (80)	0.430 [2]
Female	9 (39.1)	2 (20)	
ECOG PS; n (%)			
score 0	7 (30.4)	6 (60)	0.139 [2]
score 1	16 (69.6)	4 (40)	
Histopathological diagnosis (central review); n (%)			
NET	12 (52.2)	3 (30)	0.283 [2]
NEC	11 (47.8)	7 (70)	
Clinical stage, n (%)			
III	1 (4.3)	2 (20)	0.073 [2]
IV	22 (95.7)	8 (80)	
Ki-67; n (%)			
> 20	9 (39.1)	4 (40)	1.000 [2]
> 50	14 (60.9)	6 (60)	
Extranodal locations; n (%)			
0-2	3 (13)	4 (40)	0.276 [2]
> 2	5 (21.7)	1 (10)	
Uk	15 (65.2)	5 (50)	
PD-L1 Combined Positive Score (central review); n (%)			
Negative (0 - 1)	10 (43.5)	4 (40)	0.624 [2]
Positive (> 1)	6 (26.1)	1 (10)	
Uk	7 (30.3)	5 (50)	

1: U of Mann–Whitney test; 2: Fisher Exact test
Abbreviations: NECs, neuroendocrine carcinomas; NETs, neuroendocrine tumors; OS, overall survival; PD-L1, programmed death ligand 1; ECOG PS, Eastern Cooperative Oncology Group performance status

Supplementary Table 5. Durvalumab plus Tremelimumab safety. Most prevalent treatment-related adverse events according to maximum grade (Common Terminology Criteria for Adverse Events, v.4.03). Only TRAEs with frequency ≥ 5 %.

Event; n (%)	Lung-NENs (N = 27)		G1/2 GI-NETs (N = 31)		G1/2 pan-NETs (N = 32)		G3 GEP-NENs (N = 33)		DUNE trial (N = 123)	
	**Any grade**	**Grade ≥ 3**	**Any grade**	**Grade ≥ 3**	**Any grade**	**Grade ≥ 3**	**Any grade**	**Grade ≥ 3**	**Any grade**	**Grade ≥ 3**
Fatigue	8 (29.6)	2 (7.4)	15 (48.4)	0 (0)	20 (62.5)	2 (6.3)	12 (36.4)	0 (0)	55 (44.7)	4 (3.3)
Diarrhoea	11 (40.7)	1* (3.7)	12 (38.7)	3 (9.7)	11 (34.4)	2 (6.3)	6 (18.2)	2 (6.1)	40 (32.5)	8 (6.5)
Pruritus	4 (14.8)	0 (0)	10 (32.3)	0 (0)	10 (31.3)	0 (0)	5 (15.2)	0 (0)	29 (23.6)	0 (0)
Nausea	1 (3.7)	0 (0)	6 (19.4)	0 (0)	8 (25)	0 (0)	3 (9.1)	1 (3)	18 (14.6)	1 (0.8)
Hypothyroidism	2 (7.4)	1 (3.7)	5 (16.1)	0 (0)	2 (6.3)	0 (0)	4 (12.1)	0 (0)	13 (10.6)	1 (0.8)
Arthralgia	2 (7.4)	0 (0)	4 (12.9)	0 (0)	6 (18.8)	0 (0)	1 (3)	0 (0)	13 (10.6)	0 (0)
Vomiting	2 (7.4)	1 (3.7)	3 (9.7)	0 (0)	3 (9.4)	1 (3.1)	4 (12.1)	1 (3)	12 (9.8)	3 (2.4)
Skin and subcutaneous disorders	3 (11.1)	0 (0)	3 (9.7)	0 (0)	5 (15.6)	0 (0)	1 (3)	0 (0)	12 (9.8)	0 (0)
Mucositis oral	2 (7.4)	0 (0)	2 (6.5)	0 (0)	5 (15.6)	0 (0)	2 (6.1)	0 (0)	11 (8.9)	0 (0)
Gastrointestinal disorders	4 (14.8)	0 (0)	6 (19.4)	0 (0)	1 (3.1)	0 (0)	0 (0)	0 (0)	11 (8.9)	0 (0)
Anorexia	2 (7.4)	0 (0)	4 (12.9)	0 (0)	1 (3.1)	0 (0)	4 (12.1)	0 (0)	11 (8.9)	0 (0)
Rash	0 (0)	0 (0)	5 (16.1)	0 (0)	4 (12.5)	0 (0)	1 (3)	0 (0)	10 (8.1)	0 (0)
Abdominal pain	2 (7.4)	0 (0)	5 (16.1)	0 (0)	2 (6.3)	0 (0)	1 (3)	0 (0)	10 (8.1)	0 (0)
Constipation	1 (3.7)	0 (0)	1 (3.2)	0 (0)	6 (18.8)	0 (0)	1 (3)	0 (0)	9 (7.3)	0 (0)
Rash maculo-papular	1 (3.7)	0 (0)	1 (3.2)	0 (0)	2 (6.3)	0 (0)	4 (12.1)	0 (0)	8 (6.5)	0 (0)
AST increased	0 (0)	0 (0)	1 (3.2)	1 (3.2)	6 (18.8)	5 (15.6)	1 (3)	0 (0)	8 (6.5)	6 (4.9)

ALT increased	0<br>(0)	0<br>(0)	1<br>(3.2)	1<br>(3.2)	7<br>(21.9)	5<br>(15.6)	0<br>(0)	0<br>(0)	8<br>(6.5)	6<br>(4.9)
Dry skin	0<br>(0)	0<br>(0)	4<br>(12.9)	0<br>(0)	2<br>(6.3)	0<br>(0)	1<br>(3)	0<br>(0)	7<br>(5.7)	0<br>(0)

*One patient died as a consequence of treatment-related diarrhoea and encephalitis infection G5.
One patient died as a consequence of treatment-related hepatic failure G5.
One patient died as a consequence of treatment-related myasthenia gravis G5.
One patient died as a consequence of treatment-related upper respiratory infection G5.

Abbreviations: TRAE, Treatment-related Adverse Event; GEP, Gastroenteropancreatic; GI, gastrointestinal; NECs, neuroendocrine carcinomas; NENs, neuroendocrine neoplasms; NETs, neuroendocrine tumors; pan, pancreatic.

SUPPLEMENTARY NOTE

Investigational Drug Substance (s)	Durvalumab (MEDI4736) and Tremelimumab
Study Number	ESR 15-11561-61-DUNE
Version Number	5.0
Date	JUNE 09th, 2021

A PHASE II STUDY OF DURVALUMAB (MEDI4736) PLUS TREMELIMUMAB FOR THE TREATMENT OF PATIENTS WITH ADVANCED NEUROENDOCRINE NEOPLASMS OF GASTROENTEROPANCREATIC OR LUNG ORIGIN (THE DUNE TRIAL)

Sponsor: GETNE

(Spanish Group of Neuroendocrine and Endocrine Tumors)

[redacted]

Coordinating Investigator: [redacted]

[redacted]

SPONSOR'S SIGNATURE PAGE

Study title: A phase II study of durvalumab (MEDI4736) plus tremelimumab for the treatment of patients

with advanced neuroendocrine neoplasms of gastroenteropancreatic or lung origin (the DUNE trial).

Study code: ESR 15-11561-61 – DUNE

EudraCT Number: 2016-002858-20

Version number and date: 5.0, June 09th,2021

[redacted]

GETNE Chairman	Signature	Signature date (DD-mm-YYYY)

[redacted]

Coordinating Investigator	Signature	Signature date (DD-mm-YYYY)

INVESTIGATOR'S SIGNATURE PAGE

Study title: A phase II study of durvalumab (MEDI4736) plus tremelimumab for the treatment of patients

with advanced neuroendocrine neoplasms of gastroenteropancreatic or lung origin (the DUNE trial).

Study code: ESR 15-11561-61 – DUNE

EudraCT Number: 2016-002858-20

Version number and date: 5.0, June 09th ,2021

______________________________________ ___________________________

Principal Investigator's signature Signature date (DD-mm-YYYY)

____

Investigator's name (capital letters)

PROTOCOL SYNOPSIS

Clinical Protocol ESR 15-11561-61

Study Title: A phase II study of durvalumab (MEDI4736) plus tremelimumab for the treatment of patients with advanced neuroendocrine neoplasms of gastroenteropancreatic or lung origin (the DUNE trial)

Protocol Number: ESR 15-11561-61

EudraCT Number: 2016-002858-20

Clinical Phase: II

Study Duration: 3 years

Investigational Product(s) and Reference Therapy:

Durvalumab will be supplied in glass vials containing 500 mg of liquid solution at a concentration of 50 mg/mL for intravenous (IV) administration.

Tremelimumab is supplied as a sterile solution for IV infusion, filled in 20 mL clear glass vials with a rubber stopper and aluminum seal. Each vial contains 20 mg/mL (with a nominal fill of 20 mL, accounting to 400 mg/vial or with a nominal fill of 1.25 mL, accounting to 25 mg/vial) of tremelimumab, in an isotonic solution at pH 5.5.

Research Hypothesis

Well-differentiated gastroenteropancreatic and lung neuroendocrine tumors are generally malignancies with a prolonged natural history. However, clinical behavior is heterogeneous and when tumor progression is observed, treatment options are limited. The most used therapy for neuroendocrine tumors management are somatostatin analogs, with clear evidence of antitumor activity demonstrated by two phase III clinical trials in gastroenteropancreatic neuroendocrine tumors. However, even the use in lung carcinoids is quite usual, no antitumoral activity has been demonstrated in prospective clinical trials. In recent years, targeted therapies as sunitinib and everolimus have been approved for pancreatic neuroendocrine tumors and recently everolimus have showed a significant reduction in the risk of progression or death in patients with non-functional neuroendocrine tumors of lung and gastrointestinal origin. After the failure of these therapies, no drugs have demonstrated efficacy. Only Interferon alpha-2b is an option for these patients who have worsening symptoms of carcinoid syndrome while on treatment with somatostatin analogs and have showed some evidence of antitumoral activity in neuroendocrine tumors. The efficacy of Interferon alpha 2-b is not fully understood, but one of its antitumoral mechanisms of action could be related via stimulation of T cells. Tremelimumab and Durvalumab combination could be more efficient drugs to improve immune system activation and could obtain a significantly higher clinical benefit in these patients. We have analyzed presence of tumor-infiltrating lymphocytes (TILS) in these tumor types. We found a high number of TILS that could be activated through Tremelimumab and Durvalumab. In cases of advanced high-grade G3 neuroendocrine carcinomas (NEC), regardless of the site of the primary tumor, combination chemotherapy using cisplatin/etoposide is recommended in first-line setting (provided that the patient has adequate organ function and performance status). There is no established second-line therapy for poorly differentiated neuroendocrine carcinomas of the gastroenteropancreatic origin.

The programmed death 1 (PD-1) receptor and PD-1 ligands 1 and 2 (PD-L1, PD-L2) play integral roles in immune regulation. Expressed on activated T cells, PD-1 is activated by PD-L1 and PD-L2 expressed by stromal cells, tumor cells, or both, initiating T-cell death and localized immune suppression, potentially providing an immune-tolerant environment for tumor development and growth. Conversely, inhibition of this interaction can enhance local T-cell responses and mediate antitumor activity in nonclinical animal models.

Tremelimumab and Durvalumab would be the first immune combination agents showing efficacy in neuroendocrine neoplasms of different origins.

Objectives:

Primary Objectives:

Primary endpoint for cohorts 1, 2 and 3:

Nine-months clinical benefit rate (CBR) by Response Evaluation Criteria In Solid Tumors (RECIST 1.1), which is defined as the percentage of patients achieving complete response (CR), partial response (PR), or stable disease (SD) at month 9 after durvalumab plus tremelimumab was started.

Primary endpoint for cohort 4:

Nine-months overall survival rate, which is defined as the percentage of patients alive at month 9 after durvalumab plus tremelimumab was started.

Secondary Objective(s):

- Overall response rate (ORR) by irRECIST.
- To assess the duration of response according to irRECIST.
- To assess the median progression-free survival time (PFS) according to irRECIST.
- To assess the safety profile of Durvalumab and Tremelimumab in subjects with advanced neuroendocrine neoplasms.
- To assess the median overall survival (OS) time.
- To assess response status according to irRECIST at 6, 9 and 12 months after start of study treatment.

Exploratory Objective(s):

- To evaluate biochemical response (changes in CgA and NSE levels) and its association with response rate and progression-free survival.
- To assess whether baseline tumor and blood biomarkers may be predictive of response to durvalumab and tremelimumab therapy.
- To explore additional hypotheses related to biomarkers and relationship to durvalumab and tremelimumab efficacy and/or toxicity and neuroendocrine tumors evolution that may arise from internal or external research activities.

**Study Design:**<br><br>Prospective, multi-center, open label, stratified, exploratory, phase II study evaluating the efficacy and safety of durvalumab plus tremelimumab in different cohorts of patients with neuroendocrine neoplasms.<br><br>The study will include patients in four different cohorts:<br><br>● **Cohort 1**: Well-moderately differentiated lung neuroendocrine tumors (classically known as typical and atypical carcinoids) after progression to somatostatin analogs and one prior targeted therapy or chemotherapy.<br><br>● **Cohort 2**: G1/G2 (WHO grade 1 and 2) gastrointestinal neuroendocrine tumors after progression to somatostatin analogs and one prior targeted therapy.<br><br>● **Cohort 3**: G1/G2 (WHO grade 1 and 2) pancreatic neuroendocrine tumors after progression to standard therapies (chemotherapy, somatostatin analogs and target therapy), who have received between two and four prior lines of treatment.<br><br>● **Cohort 4**: Neuroendocrine neoplasms (WHO grade 3) of gastroenteropancreatic origin or unknown primary site (excluding lung primary tumors), patients will be treated in second line only, after progression to first-line chemotherapy with a platinum based regimen.
**Number of Centers:** 20
**Number of Subjects:** 126 patients: 31 patients for cohorts 1 to 3, and 33 for cohort 4.
**Study Population:**<br><br>Patients with advanced/metastatic, histologically confirmed, grade 1/2 (G1/G2) of the 2010 WHO classification neuroendocrine tumors of the pancreas, gastrointestinal tract and lung origins and grade 3 (G3) of gastroenteropancreatic system or unknown primary site (excluding lung primaries) after progression to previous therapies.

Inclusion Criteria:

For inclusion in the study, patients should fulfill the following criteria:

1. Written informed consent obtained from the subject prior to performing any protocol-related procedures.
2. Age >18 years at time of study entry.
3. Subjects must have histologically confirmed diagnosis of one of the following advanced/metastatic neuroendocrine tumor types:
   a) **Cohort 1**: Well-moderately differentiated neuroendocrine tumors of the lung (mitotic count ≤10 mitoses x 10 HPF), also known as typical and atypical lung carcinoids, that have progressed to prior somatostatin analog therapy and/or one prior targeted therapy or chemotherapy (only one prior systemic therapy, with the exception of patients that have been treated with somatostatin analogues and other systemic treatment, when two prior treatments are allowed).
   b) **Cohort 2**: Well-moderately differentiated G1/G2 (WHO grade 1 and 2) gastrointestinal neuroendocrine tumors after progression to somatostatin analogs and one targeted therapy (prior targeted therapy could be everolimus or a multikinase inhibitor). Prior therapies with interferon alpha-2b or radionucleotide therapy are allowed.
   c) **Cohort 3**: Well-moderately differentiated neuroendocrine tumors G1/G2 (WHO grade 1 and 2) from pancreatic origin after progression to standard therapies (chemotherapy, somatostatin analogs and target therapy); patients must be treated with at least two prior systemic treatment lines and a maximum of four previous treatment lines.
   d) **Cohort 4**: Neuroendocrine neoplasms (WHO grade 3) of gastroenteropancreatic origin or unknown primary site (excluding lung primary tumors), patients will be treated in second line only, after progression to first-line chemotherapy with a platinum based regimen.
4. For patients included in cohorts 1, 2 and 3: WHO Classification G1/G2 (mitotic count ≤10 mitoses x 10 HPF) lung typical and atypical carcinoids for cohort 1, G1/G2 (Ki67≤20% or mitotic count ≤20 mitoses x 10 HPF) gastrointestinal for cohort 2 (including stomach, small intestine and colorectal origins), G1/G2 (Ki67≤20% or mitotic count ≤20 mitoses x 10 HPF) pancreatic for cohort 3.
5. For patients included in cohort 4: WHO classification G3 (Ki67>20% or mitotic count >20 mitoses x 10 HPF) gastroenteropancreatic neuroendocrine carcinomas (NEC) or liver metastases of G3 NEC of unknown primary site.
6. Subjects must have evidence of measurable disease meeting the following criteria:
   a) In case of more than one target lesion, it should be identified at least 1 lesion of ≥ 1.0 cm in the longest diameter for a non lymph node, or ≥ 1.5 cm in the short-axis diameter for a lymph node, which is serially measurable according to RECIST 1.1 using computerized tomography/magnetic resonance imaging (CT/MRI). If there is only one target lesion and it is a non-lymph node, it should have a longest diameter of ≥ 1.5 cm.
   b) Lesions that have had external beam radiotherapy (EBRT) or loco-regional therapies such as radiofrequency (RF) ablation or liver embolization must show evidence of progressive disease based on RECIST 1.1 to be deemed a target lesion.
   c) Subjects must show evidence of disease progression by radiologic image techniques within 12 months (an additional month will be allowed to accommodate actual dates of performance of

scans, i.e., within ≤ 13 months) prior to signing informed consent, according to RECIST 1.1.

7. Eastern Cooperative Oncology Group (ECOG) performance status of 0 or 1.
8. Life expectancy of at least 12 weeks.
9. Adequate normal organ and marrow function as defined below:
   - Haemoglobin ≥ 9.0 g/dL.
   - Absolute neutrophil count (ANC) ≥ 1.5 x 10^9/L (≥ 1500 per mm^3).
   - Platelet count ≥ 100 x 10^9/L (≥100,000 per mm^3).
10. Serum bilirubin ≤ 1.5 x institutional upper limit of normal (ULN). This will not apply to subjects with confirmed Gilbert's syndrome (persistent or recurrent hyperbilirubinemia that is predominantly unconjugated in the absence of hemolysis or hepatic pathology), who will be allowed only in consultation with their physician.
11. AST (SGOT)/ALT (SGPT) ≤ 2.5 x institutional upper limit of normal unless liver metastases are present, in which case it must be ≤ 5x ULN.
12. Serum creatinine CL>40 mL/min by the Cockcroft-Gault formula (Cockcroft and Gault 1976) or by 24-hour urine collection for determination of creatinine clearance:

Males:

$$\text{Creatinine CL (mL/min)} = \frac{\text{Weight (kg)} \times (140 - \text{Age})}{72 \times \text{serum creatinine (mg/dL)}}$$

Females:

$$\text{Creatinine CL (mL/min)} = \frac{\text{Weight (kg)} \times (140 - \text{Age})}{72 \times \text{serum creatinine (mg/dL)}} \times 0.85$$

13. Female subjects must either be of non-reproductive potential (ie, post-menopausal by history: ≥60 years old and no menses for ≥1 year without an alternative medical cause; OR history of hysterectomy, OR history of bilateral tubal ligation, OR history of bilateral oophorectomy) or must have a negative serum pregnancy test upon study entry.
14. Subject is willing and able to comply with the protocol for the duration of the study including undergoing treatment and scheduled visits and examinations including follow up.

Exclusion Criteria:

Subjects who meet any of the following criteria will be excluded from this study:

1. Involvement in the planning and/or conduct of the study.
2. Participation in another clinical study with an investigational product during the last 4 weeks.
3. WHO Classification G3 neuroendocrine neoplasms of lung origin (oat cell/large cell lung cancer).
4. Prior treatment with anti-PDL-1/anti-PD-1 or anti-CTL-4 therapy.
5. Uncontrolled intercurrent illness including, but not limited to, ongoing or active infection, symptomatic congestive heart failure, uncontrolled hypertension, unstable angina pectoris, cardiac arrhythmia, active peptic ulcer disease or gastritis, active bleeding diathesis including any subject known to have evidence of acute or chronic hepatitis B (e.g., HBsAg reactive), hepatitis C (e.g., HCV RNA [qualitative] is detected) or known history of Human Immunodeficiency Virus (HIV) (HIV 1/2 antibodies), or psychiatric illness/social situations that would limit compliance with study requirements or compromise the ability of the subject to give written informed consent.
6. Known history of previous clinical diagnosis of tuberculosis.
7. Current or prior use of immunosuppressive medication within 28 days before the first dose of durvalumab or tremelimumab, with the exceptions of intranasal and inhaled corticosteroids or systemic corticosteroids at physiological doses, which are not to exceed 10 mg/day of prednisone, or an equivalent corticosteroid.
8. Active or prior documented autoimmune disease within the past 2 years NOTE: Subjects with vitiligo, Grave's disease, or psoriasis not requiring systemic treatment (within the past 2 years) are not excluded.
9. Active or prior documented inflammatory bowel disease (e.g., Crohn's disease, ulcerative colitis).
10. History of allogeneic organ transplant.
11. History of hypersensitivity to durvalumab, tremelimumab or any excipient.
12. Subjects having a diagnosis of immunodeficiency or are receiving systemic steroid therapy or any other form of immunosuppressive therapy within 28 days prior to the first dose of trial treatment.
13. Knowledge of active central nervous system (CNS) metastases and/or carcinomatous meningitis. Subjects with previously treated brain metastases may participate provided they have stable brain metastases [without evidence of progression by imaging confirmed [by magnetic resonance imaging (MRI) if MRI was used at prior imaging, or confirmed by computed tomography (CT) imaging if CT used at prior imaging] for at least four weeks prior to the first dose of trial treatment; also, any neurologic symptoms must have returned to baseline], have no evidence of new or enlarging brain metastases,and have not used steroids for brain metastases for at least 7 days prior to trial treatment. This exception does not include carcinomatous meningitis, as subjects with carcinomatous meningitis are excluded regardless of clinical stability.
14. Receipt of live attenuated vaccination within 30 days prior to study entry or within 30 days of receiving durvalumab or tremelimumab. Note: The killed virus vaccines used for seasonal influenza vaccines for injection are allowed; however intranasal influenza vaccines (e.g., FluMist®) are live attenuated vaccines, and are not allowed.
15. Subjects having known history of, or any evidence of interstitial lung disease or active, noninfectious

pneumonitis.

16. Any prior Grade ≥3 immune-related adverse event (irAE) while receiving any previous immunotherapy agent, or any unresolved irAE >Grade 1.
17. Subjects who have received any anti-cancer treatment within 21 days or any investigational agent within 30 days prior to the first dose of study drug and should have recovered from any toxicity related to previous anti-cancer treatment. This does not apply to the use of somatostatin analogues for symptomatic therapy.
18. Major surgery within 3 weeks prior to the first dose of study drug.
19. Subjects having > 1+ proteinuria on urine dipstick testing will undergo 24h urine collection for quantitative assessment of proteinuria. Subjects with urine protein ≥ 1 g/24h will be ineligible.
20. Significant cardiovascular impairment: history of congestive heart failure greater than New York Heart Association (NYHA) Class II, unstable angina; myocardial infarction or stroke within 6 months of the first dose of study drug, or cardiac arrhythmia requiring medical treatment.
21. Mean QT interval corrected for heart rate (QTc) ≥470 ms calculated from 3 electrocardiograms (ECGs) using Fredericia's Correction.
22. Bleeding or thrombotic disorders or use of anticoagulants, such as warfarin, or similar agents requiring therapeutic international normalized ratio (INR) monitoring. Treatment with low molecular weight heparin (LMWH) is allowed.
23. Active hemoptysis (bright red blood of at least 0.5 teaspoon) within 3 weeks prior to the first dose of study drug.
24. Patients with tumoral disease in the head and neck region, such as paratracheal or periesophageal lymph node involvement, or with infiltration of structures in the digestive tract, or vascular pathways that represent a risk of increased bleeding.
25. Patients of cohort 1 with neuroendocrine tumors of pulmonary origin or pulmonary metastases with evidence of active bleeding.
26. Patients with evidence of digestive bleeding.
27. Active infection (any infection requiring treatment).
28. Active malignancy (except for differentiated thyroid carcinoma, or definitively treated melanoma in-situ, basal or squamous cell carcinoma of the skin, or carcinoma in-situ of the cervix) within the past 24 months.
29. Female patients who are pregnant or breastfeeding or male or female patients of reproductive potential who are not willing to employ highly effective birth control from screening to 180 days after the last dose of durvalumab + tremelimumab combination therapy or 90 days after the last dose of durvalumab monotherapy, whichever is the longer time period.
30. Documented active alcohol or drug abuse.
31. Patients with a prior history of non-compliance with medical regimens.
32. Any condition that, in the opinion of the investigator, would interfere with evaluation of study treatment or interpretation of patient safety or study results.

GETNE
Grupo Español de Tumores Neuroendocrinos y Endocrinos

Investigational Product(s), Dose, and Mode of Administration:

Durvalumab, 1500 mg Q4W (equivalent to 20 mg/kg Q4W) for 12 months in patients ≥ 30kg.

Tremelimumab 75 mg Q4W (equivalent to 1 mg/kg Q4W) for up to 4 doses/cycles in patients ≥ 30kg.

Weight-based dosing should be used for patients <30 kg durvalumab 20 mg/kg Q4 and tremelimumab 1 mg/kg Q4.

Study Assessments and Criteria for Evaluation:

Safety Assessments:

The safety objective of this trial is to characterize the safety and tolerability of durvalumab plus tremelimumab in subjects with advanced neuroendocrine neoplasms. The primary safety analysis will be based on subjects who experienced toxicities as defined by CTCAE, v4.03 The attribution to drug, time-of-onset, duration of the event, its resolution, and any concomitant medications administered will be recorded. AEs will be analyzed including but not limited to all AEs, SAEs, fatal AEs, and laboratory changes. Furthermore, specific immune-related adverse events (irAEs) will be collected and designated as immune related events of clinical interest (ECIs).

Efficacy Assessments:

The primary efficacy objective of this study is to evaluate the anti-tumor activity of durvalumab plus tremelimumab in subjects with advanced neuroendocrine neoplasms. 9-months clinical benefit rate by Response Evaluation Criteria in Solid Tumors. (RECIST 1.1), which is defined as the percentage of patients achieving complete response, partial response (PR), or stable disease (SD) at month 9 after durvalumab plus tremelimumab was started for cohorts 1, 2 and 3, and 9-months overall survival rate, which is defined as the percentage of patients alive at 9 months after durvalumab plus tremelimumab was started will be used as the primaries endpoints per RECIST 1.1. **Pharmacodynamic / Pharmacokinetic Assessments (if applicable):**

Correlative blood samples for pharmacodynamics studies will be obtained during the course of the trial.

Archival tumor tissue will be required at baseline.

Statistical Methods and Data Analysis:

Strategic decisions about future development of immune therapy in neuroendocrine tumors will be available after the results of this trial.

Summary tables (descriptive statistics and frequency tables) will be provided for all baseline variables, efficacy variables, and safety variables, as appropriate. Continuous variables will be summarized with descriptive statistics (mean, standard deviation, range, and median). Ninety-five (95) percent confidence highest probability density (HPD) credible intervals (95% CI) may also be presented, as appropriate. Frequency counts and percentage of subjects within each category will be provided for categorical data.

The primary efficacy analysis will be performed using the binomial test procedure. Missing data will be treated using statistical multiple random imputation.

Secondary endpoints will be summarized with descriptive statistics. Continuous variables will be summarized with n, mean, standard deviation, and range, frequency counts and percentage of subjects within each category will be provided for categorical data. Multivariate regression models will be used to study relations between explanatory variables and primary endpoint. Survival analysis will be performed to analyze PFS, Kaplan-Meier curves will be presented and possible comparisons will be tested using the log-rank test or the Cox proportional hazard model for multivariate analysis, hazard ratios (HR) and their 95% confidence interval (CI95%) will be provided. Patients with lost of follow-up or treatment discontinuation will be included in the final analysis of primary endpoint if they have at least one tumor evaluation and considered as censored data for survival endpoints.

R software version 3.2.1 will be used for all analysis.

Sample Size Determination:

Sample size has been calculated using one-sample Superiority test (function One Sample Proportion. (NIS) of the Trial Size package of R software). According to previous knowledge, it is estimated that the reference value for the likelihood to be progression-free at 9 months is 30% and we expect an increase of 20% with a superiority margin of 10%. With a unilateral alpha level of 5% and 80% power, we estimate to include 28 patients per group, with an expected lost to follow-up rate of 10%, a final sample size in each 1, 2 and 3 cohort will include 31 patients.

For cohort 4, and according to previous knowledge, it is estimated that the reference proportion of patients being alive at 9 months is 13% and we expect an increase of 10% with a superiority margin of 5%. With a unilateral alpha level of 5% and 80% power, we estimate to include 30 patients per group, with an expected lost to follow-up rate of 10%, a final sample size will include 33 patients.

Summarizing, total sample size will include 126 patients: 31 patients for cohorts 1 to 3, and 33 for cohort 4.

GETNE Grupo Español de Tumores Neuroendocrinos y Endocrinos

SCHEDULE OF STUDY ASSESSMENTS (according to section 8)

Phase	Screening Phase		Treatment Phase								End of treatment [r]	Follow-up [s]
Period	Screening [,b]	Baseline[a,b]	Study Treatment									
Visit	V1<br>-28 to -2	V2<br>Day -3 to C1D1	V3<br>C1D1	V4<br>C1D15	V5<br>C2D1<br>(C1D28)	V6<br>C2D15	V7<br>C3D1<br>(C2D28)	V8<br>C3D15	V9<br>C4D1<br>(C3D28)	V10-V16<br>C5D1<br>C12D1		
Informed consent	X											
Inclusion/Exclusion	X	X										
Allocation		X										
Medical history and demographic Data	X											
ECOG PS[C]	X	X		X	X	X	X	X	X	X	X	X
Prior Therapies for neuroendocrine neoplasms	X											
Vitals signs[d]	X		X	X	X	X	X	X	X	X	X	X
Physical exam[f]	X	X[e]		X	X	X	X	X	X	X	X	X
12/lead ECG[g]	X		Only when clinically indicated									
Biochemistry and hematology[h]	X	X[h]		X	X	X	X	X	X	X	X	X
Hepatitis B and C; HIV	X											
Thyroid function test (TSH and fT3 and fT4)[i]	X			X	X	X	X	X	X	X	X	X
Urinalysis[j]	X			X	X	X	X	X	X	X	X	X
Pregnancy test[k]	X		Only when clinically indicated									
Durvalumab administration			X		X		X		X	X		
Tremelimumab administration			X		X		X		X			
Tumor assessments (CT/MRI)[l]	X		CT/MRI of chest/abdomen/pelvis and other areas of known disease at screening plus any areas of newly suspected disease should be performed every 12 weeks until confirmed PD.									
Octreoscan / PET/CT[m]	X											
Survival[n]					X		X		X	X	X	X
Biomarkers[o]		X			X						X (end of treatment/ progression)	
Archival tumor block or slides[p]		X										
Concomitant medication	X	X	X	X	X	X	X	X	X	X	X	X
AEs/SAEs[q]	X	X	X	X	X	X	X	X	X	X	X	X

AEs = adverse events, BP = blood pressure, CT = computerized tomography, ECG = electrocardiogram, ECOG = Eastern Cooperative Oncology Group, HR = heart rate, MRI = magnetic resonance imaging, PET = positron-emission tomography, RECIST 1.1 = Response Evaluation Criteria in Solid Tumors , RR = respiratory rate, SAEs = serious adverse events, surg = surgical, 99m-Tc MDP = 99m-technetium-methylene diphosphonate, w/in = within.

a) 72 hours before Cycle 1 Day 1 (C1D1). The **baseline assessment can be performed on C1D1, prior to treatment.** Informed consent may be taken up to 4 weeks prior to C1D1.

b) Efforts should be made to conduct study visits on the day scheduled (± 1 day). Clinical laboratory assessments may be conducted anytime within 72 hours prior to the scheduled visit.

c) ECOG will be performed at the Screening and Baseline Visits and at every subsequent treatment visit thereafter.

d) Assessments will include vital signs (supine BP, HR, RR, and body temperature), weight, and height.

Height will be measured at the Screening Visit only. Elevated BP (≥140/90 mmHg) should be confirmed by 3 measurements (at least 5 minutes apart). If systolic BP is ≥160 mmHg or diastolic BP ≥100 mmHg, BP should be confirmed by repeat measurements after an hour. Subjects will have their blood pressure and pulse measured before, during, and after the infusion at the following times (based on a 60-minute infusion):

- At the beginning of the infusion (at 0 minutes)
- At 30 minutes during the infusion (±5 minutes)
- At the end of the infusion (at 60 minutes ±5 minutes)
- In the 1 hour observation period post-infusion: 30 and 60 minutes after the infusion (ie, 90 and 120 minutes from the start of the infusion) (±5 minutes) – for the first infusion only and then for subsequent infusions as clinically indicated

If the infusion takes longer than 60 minutes, then blood pressure and pulse measurements should follow the principles as described above or more frequently if clinically indicated. According to section 8.2.4.

e) Required if screening physical examination was performed > 7 days prior C1D1.

f) A comprehensive physical examination (including a neurological examination) will be performed at the Screening or Baseline Visit, on each visit of the Treatment phase, and at the off-treatment assessment. A symptom-directed physical examination will be performed on Cycle 1/Day 1 and at any time during the study, as clinically indicated.

g) Single 12-lead ECG. Subjects must be in the recumbent position for a period of 5 minutes prior to the ECG.

h) Assessments scheduled may be performed within 72 hours prior to the visit. Assessments during follow up are made every 8-12 weeks, according to investigator's criteria. According to section 8.2.5.

i) Free T3 and free T4 will only be measured if TSH is abnormal. They should also be measured if there is clinical suspicion of an adverse event related to the endocrine system. Assessments during follow up are made every 8-12 weeks, according to investigator's criteria.

j) Assessments during follow up are made every 8-12 weeks, according to investigator's criteria.

k) A serum or urine pregnancy test will be performed at the Screening Visit, at the Baseline Visit (or within 72 hours prior to the first dose of study medication)

l) Screening tumor assessments using CT of the chest/abdomen/pelvis and other areas of known disease or newly suspected disease should be performed within 28 days prior to C1D1. Scans of the abdomen, pelvis and other areas of the body may be done with MRI instead of CT, but evaluation of the chest should be done with CT. CT scans should be performed with oral and iodinated IV contrast and MRI scans with IV gadolinium chelate unless there is a medical contraindication to contrast. If iodinated IV contrast is contraindicated, chest CT should be done without IV contrast.

m) Somatostatin receptor scintigraphy (octreoscan) or PET-CT should be performed and/or available within the previous 6 months before C1D1, only on cohort 1-3. It is not applicable on cohort 4.

n) Survival data will be collected every 4 weeks until end of treatment phase is declared. All anticancer therapies will be collected. Survival data and other cancer treatments received will be collected every 6 months until close of the study database. The study sponsor may elect to discontinue survival follow-up.

o) Collection of blood sample to obtain plasma, serum, or other components to be used for biomarker studies.

Samples will be obtained at baseline, Cycle 2/Day 1, and at end-of-treatment visit.

p) All subjects will have collection of most recent archived, tumor-biopsy sections for identification of predictive biomarkers.

q) Throughout the study from the signature of Informed Consent. SAE irrespective of relationship to study treatment must be reported as soon as possible but not later than one business day. AEs and concomitant meds

collected 28 days from last dose.

r) See section 8.1.3, APPENDIX 2 and APPENDIX 3.

s) See section 8.1.3, APPENDIX 2 and APPENDIX 3.

LIST OF TABLES **¡Error! Marcador no definido.**

LIST OF APPENDICES 23

ABBREVIATIONS AND DEFINITION OF TERMS 24

1. INTRODUCTION 28

1.1 Disease Background 28

1.1.1 Immunotherapies 30

1.1.2 Durvalumab 30

1.1.3 Tremelimumab 31

1.1.4 Durvalumab in combination with tremelimumab 31

1.2 Research hypothesis 32

1.3 Rationale for conducting this study 32

1.3.1 Durvalumab + tremelimumab combination therapy dose rationale 33

1.3.2 Rationale for 4 cycles of combination therapy followed by durvalumab monotherapy 34

1.4 Benefit/risk and ethical assessment 35

1.4.1 Potential benefits 35

1.4.1.1 Durvalumab 35

1.4.1.2 Tremelimumab 35

1.4.1.3 Durvalumab + tremelimumab 35

1.4.2 Potential risks 36

1.4.2.1 Durvalumab 36

1.4.2.3 Durvalumab + tremelimumab 39

1.4.2.4 Fixed Dosing for durvalumab and tremelimumab 41

2. STUDY OBJECTIVES 42

2.1 Primary objective(s) 42
2.2 Secondary objective(s) 43
2.3 Exploratory objective(s) 43

3. STUDY DESIGN 43
3.1 Overview of study design 43
3.2 Study schema 44
3.3 Study Oversight for Safety Evaluation 45

4. PATIENT SELECTION, ENROLLMENT, ALLOCATION, RESTRICTIONS,

DISCONTINUATION AND WITHDRAWAL 45
4.1 Inclusion criteria 45
4.2 Exclusion criteria 47
4.3 Withdrawal of Subjects from Study Treatment and/or Study 49
4.4 Replacement of subjects 50

5. INVESTIGATIONAL PRODUCT(s) 50
5.1 Durvalumab and tremelimumab 50
5.1.1 Formulation/packaging/storage 50
5.2 Dose and treatment regimens 51
5.2.1 Treatment regimen 51
5.2.2 Duration of treatment and criteria for retreatment 51
5.2.3 Study drug preparation of durvalumab and tremelimumab 52
5.2.4 Monitoring of dose administration 54
5.2.5 Accountability and dispensation 54
5.2.6 Disposition of unused investigational study drug 54

6. TREATMENT PLAN 56
6.1 Subject enrollment and allocation 56
6.1.1 Procedures for allocation 56
6.1.2 Procedures for handling subjects incorrectly enrolled 56
6.2 Dose Modification and Toxicity Management 56
6.2.1 Durvalumab and tremelimumab 56

7. RESTRICTIONS DURING THE STUDY AND CONCOMITANT TREATMENT(s) 57
7.1 Restrictions during the study 57
7.2 Concomitant treatment(s) 58
7.2.1 Permitted concomitant medications 58
7.2.2 Excluded Concomitant Medications 58

8. STUDY PROCEDURES 59
8.1 Schedule of study procedures 59
8.1.1 Screening Phase 59
8.1.2 Treatment Phase 60
8.1.3 End of Treatment 63
8.2 Description of study procedures 64
8.2.1 Medical history and physical examination, electrocardiogram, weight and vital signs 64
8.2.2 Physical examination 64
8.2.3 Electrocardiograms 64
8.2.4 Vital signs 64

8.2.5 Clinical laboratory tests 65
8.3 Biological sampling procedures 66
8.3.1 Biomarker/Pharmacodynamic sampling and evaluation methods 67
8.3.2 Estimate of volume of blood to be collected 68
8.3.3 Archival tumor samples 68
8.3.3.1 Archival tumor samples 68
8.3.4 Withdrawal of informed consent for donated biological samples 69

9. DISEASE EVALUATION AND METHODS 70
Primary Objective 70
Secondary objectives (irRECIST) 70
9.1 Efficacy variable 71

10. ASSESSMENT OF SAFETY 71
10.1 Safety Parameters 71
10.1.1 Definition of adverse events 71
10.1.2 Definition of serious adverse events 72
10.1.3 Durvalumab + tremelimumab adverse events of special interest 72
10.2 Assessment of safety parameters 75
10.2.1 Assessment of severity 75
10.2.2 Assessment of relationship 75
10.3 Recording of adverse events and serious adverse events 75
10.3.1 Study recording period and follow-up for adverse events and serious adverse events 76
10.3.2 Reporting of serious adverse events 77
10.3.2.1 Reporting of deaths 78
10.3.3 Other events requiring reporting 78
10.3.3.1 Overdose 78
10.3.3.2 Hepatic function abnormality 78
10.3.3.3 Pregnancy 79

11. STATISTICAL METHODS AND SAMPLE SIZE DETERMINATION 80
11.1. Description of analysis sets 80
11.2. Methods of statistical analyses 81
11.3. Determination of sample size 81

12. ETHICAL AND REGULATORY REQUIREMENTS 82
12.1. Ethical conduct of the study 82
12.2. Ethics and regulatory review 82
12.3. Informed consent 82
12.4. Changes to the protocol and informed consent form 83

12.5. Audits and inspections 83
12.6. Confidentiality 83
12.7. Insurance 84
12.8. Publications 84
13.1. Training of study site personnel 84
13.2. Monitoring of the study 84
13.3. Source data 85
13.4. Retention of Records 85
13.5. Quality assurance 85
13.6. Study timetable and end of study 86

14. DATA MANAGEMENT 86

15. INVESTIGATIONAL PRODUCT AND OTHER TREATMENTS 87
15.1. Identity of investigational product(s) 87

16. LIST OF REFERENCES 87
Appendix 1 Dosing Modification and Toxicity Management Guidelines for Immune-Mediated, Infusion-Related, and Non-Immune–Mediated Reactions (MEDI4736 Monotherapy or Combination Therapy With Tremelimumab or Tremelimumab Monotherapy) 96
Appendix 2 Schedule of study procedures follow-up for subjects who have completed durvalumab and tremelimumab treatment and achieved disease control (until confirmed progression of disease) and subjects who have discontinued durvalumab or tremelimumab due to toxicity in the absence of confirmed progression of disease. ¡Error! Marcador no definido.
Appendix 3 Schedule of study procedures: follow-up for subjects who have discontinued durvalumab or tremelimumab treatment due to confirmed progression of disease at the investigator discretion 140
Appendix 4 Durvalumab dose calculations 141
Appendix 5 Durvalumab Dose Volume Calculations 142
Appendix 6 Tremelimumab Dose Calculations 143
Appendix 7 Tremelimumab Dose Volume Calculations 145
Appendix 8 Contact Details 146

LIST OF TABLES

Table 1. Groups of neuroendocrine neoplasms following the WHO classification

Table 2. Effective methods of contraception (2 methods must be used)

Table 3. Prohibited and Rescue Medications

Table 4. Hematology Laboratory Tests

Table 5. Clinical chemistry (Serum or Plasma) Laboratory Tests

Table 6. Urinalysis Tests[a]

Tabla 7. List of investigational products for this study.

LIST OF APPENDICES

Appendix 1. Dosing Modification and Toxicity Management Guidelines for Immune-Mediated, Infusion-Related, and Non-Immune–Mediated Reactions

Appendix 2. Schedule of study procedures: follow-up for subjects who have completed durvalumab and tremelimumab treatment and achieved disease control (until confirmed progression of disease) and subjects who have discontinued durvalumab or tremelimumab due to toxicity in the absence of confirmed progression of disease.

Appendix 3. Schedule of study procedures: follow-up for subjects who have discontinued nivolumab or tremelimumab treatment due to confirmed progression of disease at the investigator discretion.

Appendix 4. Durvalumab DOSE CALCULATIONS

Appendix 5. Durvalumab VOLUME CALCULATIONS

Appendix 6. Tremelimumab DOSE CALCULATIONS

Appendix 7. Tremelimumab VOLUME CALCULATIONS

Appendix 8. Contact details.

Appendix 9. Sites and Investigators participants.

ABBREVIATIONS AND DEFINITION OF TERMS

The following abbreviations and special terms are used in this study Clinical Study Protocol.

Abbreviation	Explanation or special term
AchE	Acetylcholinesterase
ADA	Anti-drug antibody
AE	Adverse event
AESI	Adverse event of special interest
ALK	Anaplastic lymphoma kinase
ALT	Alanine aminotransferase
APF12	Proportion of patients alive and progression free at 12 months from randomization
AST	Aspartate aminotransferase
AUC	Area under the curve
AUC0-28day	Area under the plasma drug concentration-time curve from time zero to Day 28 post-dose
AUCss	Area under the plasma drug concentration-time curve at steady state
BoR	Best objective response
BP	Blood pressure
C	Cycle
CD	Cluster of differentiation
CI	Confidence interval
CL	Clearance
C_{max}	Maximum plasma concentration
$C_{max,ss}$	Maximum plasma concentration at steady state
CR	Complete response
CSA	Clinical study agreement
CSR	Clinical study report
CT	Computed tomography
CTCAE	Common Terminology Criteria for Adverse Event
CTLA-4	Cytotoxic T–lymphocyte-associated antigen 4
$C_{trough,ss}$	Trough concentration at steady state
CXCL	Chemokine (C-X-C motif) ligand
DoR	Duration of response
EC	Ethics Committee, synonymous to Institutional Review Board and Independent Ethics Committee
ECG	Electrocardiogram
ECOG	Eastern Cooperative Oncology Group
eCRF	Electronic case report form
EdoR	Expected duration of response
EGFR	Epidermal growth factor receptor
EU	European Union
FAS	Full analysis set
FDA	Food and Drug Administration
GCP	Good Clinical Practice
GI	Gastrointestinal
GMP	Good Manufacturing Practice
hCG	Human chorionic gonadotropin
HIV	Human immunodeficiency virus
HR	Hazard Ratio
IB	Investigator’s Brochure
ICF	Informed consent form
ICH	International Conference on Harmonisation

IDMC	Independent Data Monitoring Committee
IFN	Interferon
IgE	Immunoglobulin E
IgG	Immunoglobulin G
IHC	Immunohistochemistry
IL	Interleukin
ILS	Interstitial lung disease
IM	Intramuscular
IMT	Immunomodulatory therapy
IP	Investigational product
irAE	Immune-related adverse event
IRB	Institutional Review Board
irRECIST	Immune-related Response Evaluation Criteria in Solid Tumors
ITT	Intent-to-Treat
IV	Intravenous
IVRS	Interactive Voice Response System
IWRS	Interactive Web Response System
mAb	Monoclonal antibody
MDSC	Myeloid-derived suppressor cell
MedDRA	Medical Dictionary for Regulatory Activities
MHLW	Minister of Health, Labor, and Welfare
miRNA	Micro-ribonucleic acid
MRI	Magnetic resonance imaging
NCI	National Cancer Institute
NE	Not evaluable
NSCLC	Non–small-cell lung cancer
OAE	Other significant adverse event
ORR	Objective response rate
OS	Overall survival
PBMC	Peripheral blood mononuclear cell
PD	Progressive disease
PD-1	Programmed cell death 1
PD-L1	Programmed cell death ligand 1
PD-L2	Programmed cell death ligand 2
PDx	Pharmacodynamic(s)
PFS	Progression-free survival
PFS2	Time to second progression
PGx	Pharmacogenetic research
PK	Pharmacokinetic(s)
PR	Partial response
q2w	Every 2 weeks
q3w	Every 3 weeks
q4w	Every 4 weeks
q6w	Every 6 weeks
q8w	Every 8 weeks
QTcF	QT interval corrected for heart rate using Fridericia's formula
RECIST 1.1	Response Evaluation Criteria in Solid Tumors, version 1.1
RNA	Ribonucleic acid
RR	Response rate

RT-QPCR	Reverse transcription quantitative polymerase chain reaction
SAE	Serious adverse event
SAP	Statistical analysis plan
SAS	Safety analysis set
SD	Stable disease
SNP	Single nucleotide polymorphism
SoC	Standard of Care
sPD-L1	Soluble programmed cell death ligand 1
T_3	Triiodothyronine
T_4	Thyroxine
TSH	Thyroid-stimulating hormone
ULN	Upper limit of normal
US	United States

1. INTRODUCTION

Neuroendocrine neoplasms constitute a heterogeneous group of tumors derived from the Kultchitzky cells of the diffuse neuroendocrine system located in the gastrointestinal tract (origin of classical carcinoid tumors), pancreatic islet cells (origin of pancreatic endocrine tumors) and lung (origin of typical and atypical carcinoids). Advanced disease is common at diagnosis leading to limited options of curative strategies, so general prognosis is poor. Neuroendocrine neoplasms are considered rare diseases, however, taken all together into account and the natural history of well-differentiated tumors, their prevalence is increasing. Systemic treatment options have been limited for several years. However, in the last decade, the better knowledge of molecular biology of neuroendocrine neoplasms has increased the interest to develop targeted agents in this field, changing the scenario of possible therapies for advanced disease. Even though, treatment options are still limited.

Immune balance has always been related with neuroendocrine neoplasms. Prolonged stabilizations have often been described without treatment and initial immune modulators, such as interferon, have demonstrated antitumoral effects in these tumors. Recent advances in immunotherapy have permitted the development of new targeted agents with higher efficacy and lower toxicity that opens a new treatment option for patients with advanced neuroendocrine tumors.

1.1 Disease Background

Well-differentiated gastroenteropancreatic and lung neuroendocrine tumors are generally malignancies with a prolonged natural history. However, clinical behavior is heterogeneous and when tumor progression is observed, treatment options are limited. Although advanced neuroendocrine neoplasms represent less than 2% of all gastrointestinal cancers, they constitute the second most prevalent advanced tumor of the gastrointestinal tract after colorectal cancer (Yao et al, 2008). The biological behavior of neuroendocrine neoplasms depends mainly on their histological characteristics, defined by the differentiation grade and Ki67 expression. The most recent 2010 WHO classification divides neuroendocrine neoplasms in three groups regarding Ki67 value and mitotic count: neuroendocrine tumors that include grade 1 (G1) and grade 2 (G2) with Ki67 value ≤20% and mitotic count ≤20 mitoses x 10 HPF, and neuroendocrine carcinomas that include grade 3 (G3) with Ki67>20% and/or mitotic count >20 mitoses x 10 HPF.

Table 1. Groups of neuroendocrine neoplasms following the WHO classification

	Lung and Thymus	GEP-NETs	
Grade	Mitotic count (10 HPF)	Mitotic count (10 HPF)	Ki-67 (%)
G1	< 2	<2	≤2
G2	2-10	2-20	3-20
G3	>10	>20	>20

The medical treatment of advanced well-moderately differentiated (G1/G2) neuroendocrine neoplasms has included somatostatin analogs, interferon and cytotoxic agents. The lack of effectiveness of conventional cytotoxic agents has prompted exploration of new targeted drugs exploiting phenotypical features of neuroendocrine neoplasms. Neuroendocrine neoplasms are characterized by being remarkably vascular and expressing several growth factors, including vascular endothelial growth factor (VEGF), platelet-derived growth factor (PDGF), insulin-like growth factor 1 (IGF-1), basic fibroblast growth factor (bFGF), and transforming growth factor (TGF)-α and -β. In addition, expression of several receptors of these growth factors and ligands has been described, including stem cell factor receptor (c-KIT), epidermal growth factor receptors (EGFR), VEGF receptors (VEGFR)-2 and 3, IGF receptors (IGF-R), and PDGF receptors (PDGFR). The (over)expression of some of these factors has been associated with poor prognosis and decreased progression-free survival (PFS), as well as with tumor growth, aggressiveness, and disease extent in patients with gastroenteropancreatic neuroendocrine neoplasms. Several targeted agents have been studied in the treatment

of neuroendocrine neoplasms including antiangiogenic compounds (such as bevacizumab), inhibitors of multiple receptors with kinase activity (such as sunitinib), and inhibitors of intracellular downstream effector proteins such as the mammalian target of rapamycin (mTOR) everolimus. Three phase III studies with everolimus and sunitinib have demonstrated efficacy in G1/G2 neuroendocrine tumors of the gastroenteropancreatic system becoming new treatment options in the management of these patients (Raymond et al 2011; Yao et al 2011; Yao et al 2015). Everolimus has also demonstrated efficacy in G1/G2 lung neuroendocrine tumors (typical and atypical carcinoids), becoming the first drug that has demonstrated a significant increase in progression-free survival in this setting (Yao et al 2015).

For advanced high-grade neuroendocrine carcinomas (G3), regardless of the site of the primary tumor, combination chemotherapy using cisplatin-based regimens is recommended early (provided that the patient has adequate organ function and performance status). Responses are frequently seen, however impact in overall survival is limited and life expectancy of these patients is around 12-16 months. There is no established second-line therapy for G3 neuroendocrine carcinomas of the gastroenteropancreatic system.

Immune responses directed against tumors are one of the body's natural defense against the growth and proliferation of cancer cells. However, over time and under pressure from immune attack, cancers develop strategies to evade immune-mediated killing allowing them to develop unchecked. One such mechanism involves upregulation of surface proteins that deliver inhibitory signals to cytotoxic T cells. Programmed cell death ligand 1 (PD-L1) is one such protein, and is upregulated in a broad range of cancers with a high frequency, with up to 88% expression in some tumor types. In a number of these cancers, including lung (Mu et al, 2011), renal (Krambeck et al, 2007), pancreatic (Nomi et al, 2007; Loos et al, 2008), ovarian cancer (Hamanishi et al, 2007), and hematologic malignancies (Andorsky et al, 2011; Brusa et al, 2013) tumor cell expression of PD-L1 is associated with reduced survival and an unfavorable prognosis.

Programmed cell death ligand 1 is part of a complex system of receptors and ligands that are involved in controlling T-cell activation. PD-L1 acts at multiple sites in the body to help regulate normal immune responses and is used by tumors to help evade detection and elimination by the host immune system tumor response. In the lymph nodes, PD-L1 on antigen-presenting cells binds to PD-1 or CD80 on activated T cells and delivers an inhibitory signal to the T cell (Keir et al, 2008; Park et al, 2010). This results in reduced T-cell activation and fewer activated T cells in circulation. In the tumor microenvironment, PD-L1 expressed on tumor cells binds to PD-1 and CD80 on activated T cells reaching the tumor. This delivers an inhibitory signal to those T cells, preventing them from killing target cancer cells and protecting the tumor from immune elimination (Zou and Chen, 2008).

Immune responses directed against tumors are one of the body's natural defenses against the growth and proliferation of cancer cells. T cells play a critical role in antitumor immunity and their infiltration and activity have been linked to improved prognosis in a number of cancers (Pagès et al, 2010; Suzuki et al, 2011). Immune evasion, primarily through suppression of T-cell activity, is now recognized as one of the hallmarks of cancer. Such evasion can occur via a range of mechanisms including production of suppressive cytokines such as IL-10, secretion of chemokines and growth factors that recruit and sustain suppressive regulatory T cells (Tregs) and inflammatory macrophages, and expression of inhibitory surface molecules such as B7-H1. Tumor types characterized as being responsive to immunotherapy-based approaches include melanoma, renal cell carcinoma (RCC), bladder cancer , and malignant mesothelioma (Bograd et al, 2011). Inhibition of CTLA-4 signaling is a validated approach to cancer therapy, as shown by the approval in 2011 of ipilimumab for the treatment of metastatic melanoma based on statistically significant and clinically meaningful improvement in OS (Robert et al, 2011).

In general, tumor response rates to anti-CTLA-4 therapy are low (~10%). However, in patients who respond, the responses are generally durable, lasting several months even in patients with aggressive tumors such as refractory metastatic melanoma. Because these agents work through activation of the immune system and not by directly targeting the tumor, responses can occur late and some patients may have perceived progression of

their disease in advance of developing disease stabilization or a tumor response. In some cases, early growth of pre-existing lesions or the appearance of new lesions may have been due to immune-cell infiltration into the tumor and not due to proliferation and extension of neoplastic cells, per se (Wolchok et al, 2009). Overall, although the impact on conventionally-defined PFS can be small, durable response or stable disease seen in a proportion of patients can lead to significant prolongation of OS. The melanoma data with ipilimumab clearly demonstrate that a small proportion of patients with an objective response had significant prolongation of OS, supporting the development of this class of agents in other tumors. Although Phase 2 and Phase 3 studies of tremelimumab in metastatic melanoma did not meet the primary endpoints of response rate and OS, respectively, the data suggest activity of tremelimumab in melanoma (Ribas et al, 2013). In a large Phase 3 randomized study comparing tremelimumab with dacarbazine (DTIC)/temozolomide in patients with advanced melanoma, the reported median OS in the final analysis was 12.58 months for tremelimumab versus 10.71 months for DTIC/temozolomide (HR = 1.1416, p = 0.1272; Ribas et al, 2013).

1.1.1 Immunotherapies

It is increasingly understood that cancers are recognized by the immune system, and, under some circumstances, the immune system may control or even eliminate tumors (Dunn et al, 2004). Studies in mouse models of transplantable tumors have demonstrated that manipulation of co-stimulatory or co-inhibitory signals can amplify T-cell responses against tumors (Peggs et al 2009). This amplification may be accomplished by blocking co-inhibitory molecules, such as cytotoxic T-lymphocyte-associated antigen 4 (CTLA-4) or programmed cell death 1 (PD-1), from binding with their ligands, B7 or B7-H1 (programmed cell death ligand 1 [PD-L1]).

1.1.2 Durvalumab

The non-clinical and clinical experience is fully described in the last version of the durvalumab Investigator's Brochure .

Durvalumab is a human monoclonal antibody (mAb) of the immunoglobulin G (IgG) 1 kappa subclass that inhibits binding of PD-L1 and is being developed by [redacted] for use in the treatment of cancer ([redacted] is a wholly owned subsidiary of [redacted] will be referred to as [redacted] throughout this document). As durvalumab is an engineered mAb, it does not induce antibody-dependent cellular cytotoxicity or complement-dependent cytotoxicity. The proposed mechanism of action for durvalumab is interference of the interaction of PD-L1.

PD-L1 is expressed in a broad range of cancers with a high frequency, up to 88% in some types of cancers. In a number of these cancers, including lung, the expression of PD-L1 is associated with reduced survival and an unfavorable prognosis. In lung cancer, only 12% of patients with tumors expressing PD-L1 survived for more than 3 years, compared with 20% of patients with tumors lacking PD-L1 (Mu et al, 2011). Based on these findings, an anti-PD-L1 antibody could be used therapeutically to enhance anti-tumor immune responses in patients with cancer. Results of several non-clinical studies using mouse tumor models support this hypothesis, where antibodies directed against PD-L1 or its receptor PD-1 showed anti-tumor activity (Hirano et al 2005, Iwai et al 2002, Okudaira et al 2009, Zhang et al 2008).

Durvalumab has been given to humans as part of ongoing studies as a single drug or in combination with other drugs.

As of the data cut-off date (12 July 2017) across the entire clinical development program, an estimated 4067 patients have been exposed to 1 or more doses of durvalumab in [redacted]-sponsored Phase I to III studies, either as monotherapy or in combination, and 5911 patients where the treatment arm is blinded. Additionally, approximately 4000 patients have been exposed to 1 or more doses of durvalumab in ESR/IITs.
Estimates of overall cumulative patient exposure based on actual exposure data from any completed clinical trials and the enrolment/randomisation schemes for ongoing open label and blinded trials are: 3723 patients

received durvalumab monotherapy, 3372 patients received durvalumab in combination with tremelimumab.Details on the safety profile of durvalumab monotherapy, tremelimumab monotherapy and durvalumab plus tremelimumab are summarized in Section 1.4.2. Refer to the current durvalumab Investigator's Brochure for a complete summary of non-clinical and clinical information including safety, efficacy and pharmacokinetics.

1.1.3 Tremelimumab

The non-clinical and clinical experience is fully described in the last version of the tremelimumab Investigator's Brochure.

Tremelimumab is an IgG-2 kappa isotype mAb directed against the cytotoxic T-lymphocyte-associated protein 4 (CTLA-4) also known as CD152 (cluster of differentiation 152). This is an immunomodulatory therapy (IMT) that is being developed by [redacted] for use in the treatment of cancer.

Binding of CTLA-4 to its target ligands (B7-1 and B7-2) provides a negative regulatory signal, which limits T-cell activation. Anti-CTLA-4 inhibitors antagonize the binding of CTLA-4 to B7 ligands and enhance human T-cell activation as demonstrated by increased cytokine (interleukin [IL]-2 and interferon [IFN] gamma) production in vitro in whole blood or peripheral blood mononuclear cell (PBMC) cultures (Tarhini and Kikwood 2008). In addition, blockade of CTLA-4 binding to B7 by anti-CTLA-4 antibodies results in markedly enhanced T-cell activation and anti-tumor activity in animal models, including killing of established murine solid tumors and induction of protective anti-tumor immunity. See the last version of tremelimumab IB, , for more information. Therefore, it is expected that treatment with an anti-CTLA-4 antibody, such as tremelimumab, will lead to increased activation of the human immune system, increasing anti-tumor activity in patients with solid tumors.

An extensive program of non-clinical and clinical studies has been conducted for tremelimumab both as monotherapy and combination therapy with conventional anticancer agents to support various cancer indications using different dose schedules.

To date Nov/2017 tremelimumab has been given to more than 1000 patients as part of ongoing studies either as monotherapy or in combination with other anticancer agents. Refer to the current tremelimumab Investigator's Brochure for a complete summary of non-clinical and clinical information including safety, efficacy and pharmacokinetics.

1.1.4 Durvalumab in combination with tremelimumab

Targeting both PD-1 and CTLA-4 pathways may have additive or synergistic activity (Pardoll 2012) because the mechanisms of action of CTLA-4 and PD-1 are non-redundant; therefore, [redacted] is also investigating the use of durvalumab + tremelimumab combination therapy for the treatment of cancer.

Durvalumab + tremelimumab: Study D4190C00006: As of 28 February 2017, durvalumab PK (n=347) and tremelimumab PK (n=353) data were available from the dose-escalation and dose-expansion phases following durvalumab Q4W (3, 10, 15, or 20 mg/kg) or Q2W (10 mg/kg) in combination with tremelimumab Q4W (1, 3, or 10 mg/kg). An approximately dose-proportional increase in PK exposure (C max and AUC 0 to 28 days [AUC 0-28]) of both durvalumab and tremelimumab was observed over the dose range of 3 to 20 mg/kg durvalumab Q4W and 1 to 10 mg/kg tremelimumab Q4W. Exposures following multiple doses demonstrated accumulation consistent with PK parameters estimated from the first dose. The observed PK exposures of durvalumab and tremelimumab following combination were consistent with respective monotherapy data, indicating no PK interaction between these 2 agents.

As of DCO of28 February 2017, ADA data were available from 99 patients for durvalumab and 95 patients for tremelimumab in Study D4190C00006. The ADA incidence was 2.0% (2/99 patients) for durvalumab and 5.3% (5/95 patients) for tremelimumab. There was no clear relationship between ADA and the dose of either

durvalumab or tremelimumab, and no apparent association between ADA and safety or efficacy.

Durvalumab has also been combined with other anticancer agents, including gefitinib, dabrafenib, and trametinib. To date, no PK interaction has been observed between durvalumab and these agents.

1.2 Research hypothesis

The most used therapy for neuroendocrine tumors are somatostatin analogs, with clear evidence of antitumor activity demonstrated by two phase III clinical trials in gastroenteropancreatic neuroendocrine tumors. However, even the use in lung carcinoids is quite usual, no antitumoral activity has been demonstrated in prospective clinical trials. In recent years, target therapy as sunitinib and everolimus have been approved for pancreatic neuroendocrine tumors and recently everolimus have showed a significant reduction in the risk of progression or death in patients with nonfunctional neuroendocrine tumors of lung or gastrointestinal origin. After the failure of these therapies, no other drugs have demonstrated efficacy. Only Interferon alpha-2b is an option for these patients who have worsening symptoms of carcinoid syndrome while on treatment with somatostatin analogs and have showed some evidence of antitumoral activity in neuroendocrine tumors. The efficacy of Interferon alpha 2-b is not fully understood, but one of its antitumoral mechanisms of action could be related via stimulation of T cells. Tremelimumab and Durvalumab are much more efficient drugs to improve immune system activation and could obtain a significantly higher clinical benefit in these patients. The presence of tumor-infiltrating lymphocytes (TILS) in these type of tumors have been described enhancing the hypothesis of immunotherapy in this setting.

Following the knowledge of increased tumor-specific antigens with the presence of more mutational load, dedifferentiation process and treatments previously received we hypothesize that immunotherapy could have a higher effect in the setting of refractory neuroendocrine neoplasms, when tumor behavior is more aggressive and the probability of immune escape process is higher.

1.3 Rationale for conducting this study

Well and moderately differentiated neuroendocrine tumors of the gastroenteropancreatic tract and lung are orphan tumors after progression a targeted therapy. For gastrointestinal origin tumors, somatostatin analogues have been the cornerstone of antihormonal therapy during decades. During the last 5 years, antiproliferative effects have also been demonstrated in this setting. Recently, for neuroendocrine tumors of the lung (typical and atypical carcinoids), everolimus has demonstrated impact in tumor growth control and improvement in progression-free survival. However, after progression to a targeted therapy, no other drugs are approved.

Within the lack of treatment options, combinations of somatostatin analogues and interferon alpha have widely been used, mainly for their effect in tumor hormone release and for the evidence of tumor growth control in many retrospective analyses.

The hypothesis of efficacy of interferon alpha in these settings could be via stimulation of T cells. Recently, PD-L1 expression has been observed in neuroendocrine tumors such as small cell neuroendocrine carcinomas of the lung and Merkel cell carcinoma. These facts could provide a timely opportunity to develop specific studies with targeted therapies that stimulate the immune system against the tumor. Currently, several clinical trials are ongoing in these G3 high grade neuroendocrine tumors of the lung (small cell lung cancer) and skin (Merkel Cell carcinomas). However, there are no clinical trials ongoing in G3 gastroenteropancreatic neuroendocrine neoplasms (which is the most frequent site of G3 neuroendocrine carcinomas after the lung origin) and well-moderately differentiated (G1/G2) neuroendocrine tumors, which are the ones where immunotherapy with interferon alpha has demonstrated some activity.

Tremelimumab and Durvalumab would be the first immune combination agents showing efficacy in G1/G2/G3 advanced neuroendocrine neoplasms of gastroenteropancreatic or lung origin.

As an antibody that blocks the interaction between PD-L1 and its receptors, durvalumab may relieve PD-L1-

dependent immunosuppressive effects and, therefore, enhance the cytotoxic activity of anti-tumor T-cells. This hypothesis is supported by emerging clinical data from other mAbs targeting the PD-L1/PD-1 pathway, which provide early evidence of clinical activity and a manageable safety profile (Brahmer et al 2012, Topalian et al 2012). Responses have been observed in patients with PD-L1-positive tumors and patients with PD-L1-negative tumors. In addition, durvalumab monotherapy has shown durable responses in NSCLC in Study 1108 (see Section 1.4.1.1).

The rationale for combining durvalumab and tremelimumab is that the mechanisms of CTLA-4 and PD-1 are non-redundant, suggesting that targeting both pathways may have additive or synergistic activity (Pardoll 2012). In fact, combining immunotherapy agents has been shown to result in improved response rates (RRs) relative to monotherapy. For example, the concurrent administration of nivolumab and ipilimumab to patients with advanced melanoma induced higher objective response rates (ORRs) than those obtained with single-agent therapy. Importantly, responses appeared to be deep and durable (Wolchok et al 2013). Similar results have been observed in an ongoing study of durvalumab + tremelimumab in NSCLC (Antonia et al 2014), with further updated details presented in this clinical study protocol.

1.3.1 Durvalumab + tremelimumab combination therapy dose rationale

The durvalumab + tremelimumab doses and regimen selected for this study are based on the goal of selecting an optimal combination dose of durvalumab and tremelimumab that would yield sustained target suppression (sPD-L1), demonstrate promising efficacy, and have an acceptable safety profile.

In order to reduce the dosing frequency of durvalumab to align with the q4w dosing of tremelimumab, while ensuring an acceptable PK/PDx, safety, and efficacy profile, cohorts were narrowed to 15 and 20 mg/kg durvalumab q4w. PK simulations from the durvalumab monotherapy data indicated that a similar area under the plasma drug concentration-time curve at steady state (AUC_{ss}; 4 weeks) was expected following both 10 mg/kg q2w and 20 mg/kg q4w durvalumab. The observed durvalumab PK data from the D4190C00006 study were well in line with the predicted monotherapy PK data developed preclinically. This demonstrates similar exposure of durvalumab 20 mg/kg q4w and 10 mg/kg q2w, with no alterations in PK when durvalumab and tremelimumab (doses ranging from 1 to 3 mg/kg) are dosed together. While the median Cmax at steady state ($C_{max,ss}$) is expected to be higher with 20 mg/kg q4w (approximately 1.5 fold) and median trough concentration at steady state ($C_{trough,ss}$) is expected to be higher with 10 mg/kg q2w (approximately 1.25 fold), this is not expected to impact the overall safety and efficacy profile, based on existing preclinical and clinical data.

Monotonic increases in PDx activity were observed with increasing doses of tremelimumab relative to the activity observed in patients treated with durvalumab monotherapy. There was evidence of augmented PDx activity relative to durvalumab monotherapy with combination doses containing 1 mg/kg tremelimumab, inclusive of both the 15 and 20 mg/kg durvalumab plus 1 mg/kg tremelimumab combinations.

Patients treated with doses of tremelimumab above 1 mg/kg had a higher rate of adverse events (AEs), including discontinuations due to AEs, serious AEs (SAEs), and severe AEs. Between the 10 mg/kg durvalumab + 1 mg/kg tremelimumab and 10 mg/kg durvalumab + 3 mg/kg tremelimumab cohorts treated at the q2w schedule, the number of patients reporting any AE, Grade 3 AEs, SAEs, and treatment-related AEs was higher in the 10 mg/kg durvalumab + 3 mg/kg tremelimumab cohort than the 10 mg/kg durvalumab + 1 mg/kg tremelimumab cohort. A similar pattern was noted in the q4w regimens, suggesting that, as the dose of tremelimumab increased above 1 mg/kg, a higher rate of treatment-related events may be anticipated. Further, the SAEs frequently attributed to immunotherapy, pneumonitis and colitis, were more commonly seen in cohorts using either 3 or 10 mg/kg of tremelimumab compared to the 1-mg/kg dose cohorts. Together, these data suggest that a combination using a tremelimumab dose of 1 mg/kg appeared to minimize the rate of toxicity when combined with durvalumab. As a result, all combination doses utilizing either the 3 or 10 mg/kg doses of tremelimumab were eliminated in the final dose selection.

In contrast, cohorts assessing higher doses of durvalumab with a constant dose of tremelimumab did not show

an increase in the rate of AEs. The data suggested that increasing doses of durvalumab may not impact the safety of the combination as much as the tremelimumab dose. Further, safety data between the 10-mg/kg and 20-mg/kg cohorts were similar, with no change in safety events with increasing dose of durvalumab.

In Study D4190C00006, of all treatment cohorts, the cohort of 11 patients treated in the 20 mg/kg durvalumab + 1 mg/kg tremelimumab group had the fewest AEs, Grade ≥3 AEs, SAEs, and treatment discontinuations due to AEs, but still showed strong evidence of clinical activity. This cohort had a lower number of treatment-related Grade ≥3 AEs or treatment-related SAEs. No dose-limiting toxicities were reported.

Preliminary clinical activity of the durvalumab and tremelimumab combination did not appear to change with increasing doses of tremelimumab. The 15- and 20-mg/kg durvalumab q4w cohorts demonstrated objective responses at all doses of tremelimumab, and increasing doses of tremelimumab did not provide deeper or more rapid responses.

Efficacy data suggested that the 20 mg/kg durvalumab + 1 mg/kg tremelimumab dose cohort may demonstrate equivalent clinical activity to another dose combinations. Of the 45 patients, full analysis set,, there were 7 patients (15,6%) with PR, 19 patients (42,2%) with SD, and 15 patients (33,3%) with PD. Four patients were not evaluable for response .

Additionally, of all cohorts, the 20 mg/kg durvalumab + 1 mg/kg tremelimumab dose cohort had the fewest AEs, Grade ≥3 AEs, SAEs, and treatment discontinuations due to AEs, but still showed some evidence of clinical activity. All together, the data suggested that a 20 mg/kg durvalumab + 1 mg/kg tremelimumab dose combination should be selected for further development.

Refer to the current durvalumab Investigator's Brochure for a complete summary of non-clinical and clinical information on the durvalumab + tremelimumab combination, including safety, efficacy and pharmacokinetics.

1.3.2 Rationale for 4 cycles of combination therapy followed by durvalumab monotherapy

Long-term follow up on melanoma patients treated with ipilimumab, an anti-CTLA-4 targeting antibody (dosed every 3 weeks [q3w] for 4 doses and then discontinued), shows that patients responding to ipilimumab derive long-term benefit, with a 3-year OS rate of approximately 22%. Furthermore, the survival curve in this population reached a plateau at 3 years and was maintained through 10 years of follow up (Schadendorf et al, 2013).

Similar data have been presented for other anti-PD-1/PD-L1 targeting antibodies:

Nivolumab (anti-PD-1) was dosed q2w for up to 96 weeks in a large Phase I dose-escalation and expansion study, and showed responses were maintained for a median of 22.94 months for melanoma (doses 0.1 mg/kg to 10 mg/kg), 17 months for NSCLC (doses 1, 3, and 10 mg/kg), and 12.9 months for renal cell carcinoma patients (doses 1 and 10 mg/kg) at the time of data analysis (Hodi et al 2014, Bramer et al 2014, Drake et al 2013). Furthermore, responses were maintained beyond treatment discontinuation in the majority of patients who stopped nivolumab treatment (either due to protocol specified end of treatment, complete response [CR], or toxicity) for up to 56 weeks at the time of data analysis (Topalian et al 2014).

MPDL3280a (anti-PD-L1) and the combination of nivolumab with ipilimumab, in which patients were dosed for a finite time period and responses maintained beyond treatment discontinuation have been reported (Herbest et al 2013, Wolchok et al 2013).

Similar long term results may be expected with use of other immune-mediated cancer therapeutics including anti-CTLA-4 antibodies such as tremelimumab, anti PD-L1 antibodies such as durvalumab, or the combination

of the two.

1.4 Benefit/risk and ethical assessment

Natural history of neuroendocrine tumors is usually prolonged. G1/G2 neoplasms are slowing growing tumors that require continued therapy to control tumor growth, increasing the risk of long-term side effects. The prolonged effect of immunotherapy could be of especial interest in this setting, reducing the risk of chronic toxicity and maintaining the long-term benefit.

1.4.1 Potential benefits

1.4.1.1 Durvalumab

The majority of the safety and efficacy data currently available for durvalumab are based on the first time in-human, single-agent study (Study 1108) in patients with advanced solid tumors. Data from Study 1108 were presented at the European Society for Medical Oncology 2014 Congress. Overall, 456 of 694 subjects treated with durvalumab 10 mg/kg Q2W were evaluable for response (defined as having ≥ 24 weeks follow-up, measurable disease at baseline, and ≥ 1 follow-up scan, or discontinued due to disease progression or death without any follow-up scan). In PD-L1 unselected patients, the objective response rate (ORR), based on investigator assessment per Response Evaluation Criteria in Solid Tumors (RECIST)v1.1, ranged from 0% in uveal melanoma (n = 23) to 20.0% in bladder cancer (n = 15), and disease control rate at 24 weeks (DCR-24w) ranged from 4.2% in triple-negative breast cancer (TNBC; n = 24) to 39.1% in advanced cutaneous melanoma (n = 23). PD-L1 status was known for 383 of the 456 response evaluable subjects. Across the PD-L1-positive tumors, ORR was highest for bladder cancer, advanced cutaneous melanoma, hepatocellular carcinoma (HCC; n = 3 each, 33.3% each), NSCLC (n = 86, 26.7%), and squamous cell carcinoma of the head and neck (SCCHN; n = 22, 18.2%). In the PD-L1-positive subset, DCR-24w was highest in advanced cutaneous melanoma (n = 3, 66.7%), NSCLC (n = 86, 36.0%), HCC and bladder cancer (n = 3 each, 33.3% each), and SCCHN (n = 22, 18.2%)(Antonia et al 2014b).

1.4.1.2 Tremelimumab

In a single-arm, Phase II study (Study A3671008) of tremelimumab administered at 15 mg/kg every 90 days to patients with refractory melanoma, an RR of 7% and a median OS of 10 months in the second-line setting (as compared to approximately 6 months with best supportive care reported from a retrospective analysis; Korn et al 2008) were observed (Kirkwood et al 2010). In a randomized, open-label, first-line Phase III study of tremelimumab (administered at 15 mg/kg every 90 days) versus chemotherapy (dacarbazine or temozolomide) in advanced melanoma (Study A3671009), results of the final analysis showed an RR of 11% and a median OS of 12.58 months in this first-line setting as compared to 10.71 months with standard chemotherapy; however, these results were not statistically significant (Ribas et al 2013). Additionally, a Phase II maintenance study (Study A3671015) in patients with Stage IIIB or IV NSCLC who had responded or remained stable failed to achieve statistical significance. The primary endpoint of PFS at 3 months was 22.7% in the tremelimumab arm (15 mg/kg) compared with 11.9% in the best supportive care arm (Study A3671015).

1.4.1.3 Durvalumab + tremelimumab

The preclinical and clinical justification for this combination as noted in Section 1.1.4 also supports the synergy of this combination. Available data, such as those presented by Wolchok et al, suggest that the combination of agents targeting PD-1/PD-L1 and CTLA-4 may have profound and durable benefits in patients with melanoma (Wolchok et al 2013). Of the 102 subjects with advanced NSCLC treated with durvalumab in combination with tremelimumab in Study D4190C00006, 63 subjects with at least 16 weeks of follow-up were evaluable for response (defined as measurable disease at baseline and at least 1 follow-up scan; this included discontinuations due to disease progression or death without follow-up scan). Of the 63 evaluable subjects, 17 (27%) had a best overall response of PR,14 (22%) had SD,22 (35%) had PD, and 10 (16%) were not evaluable.

The ORR(confirmed and unconfirmed CR or PR) was 27% and the DCR (CR, PR, or SD) was 49% as assessed by RECIST v1.1.

Current experience with single-agent IMT studies suggests that clinical responses may be restricted to a subset of any given patient population and that it might be beneficial to enrich the patient population by selecting patients likely to respond to therapy. To date, no assay has been established or validated, and no single approach has proven accurate, for patient enrichment for IMTs. However, independent data from multiple sources using different assays and scoring methods suggests that PD-L1 expression on tumor cells and/or tumor infiltrating cells may be associated with greater clinical benefit.

Data from ongoing studies with durvalumab and other agents targeting the PD-1/PD-L1 pathway suggest, as shown in a number of tumor types (eg, NSCLC, renal cell carcinoma, and melanoma), that monotherapy may be more efficacious (in terms of ORR) in patients who are PD-L1-positive.

Given these findings, a number of ongoing studies are assessing the activity of agents in patients with PD-L1–positive tumors. There is also an unmet medical need in patients with PD-L1–negative tumors that needs to be addressed. Data, as of 27 January 2015 from Study 006 show that with the addition of tremelimumab to durvalumab, the ORR can be increased to 25% in patients with PD-L1 negative NSCLC.

1.4.2 Potential risks

1.4.2.1 Durvalumab

Risks with durvalumab include, but are not limited to, diarrhea/colitis, abdominal pain, pneumonitis/ILD, endocrinopathies (hypo- and hyperthyroidism, type I diabetes mellitus, hypophysitis and adrenal insufficiency, Thyroiditis) hepatitis/increases in transaminases, nephritis/increases in creatinine, pancreatitis/increases in amylase and lipase, rash/pruritus/dermatitis, pemphigoid, myocarditis, myositis/polymyositis, inflammatory events including neuromuscular toxicities (myasthenia gravis), infusion-related reactions, immune thrombocytopenia, hypersensitivity reactions and infections/serious infections.

For information on all identified and potential risks with durvalumab please always refer to the current version of the durvalumab IB.

In monotherapy clinical studies AEs (all grades) reported very commonly (≥ 10% of patients) are cough/productive cough, diarrhoea, abdominal pain, hypothyroidism, rash pruritus, pyrexia, and upper respiratory tract infections

Approximately 9% of patients experienced an AE that resulted in permanent discontinuation of durvalumab and approximately 6% of patients experienced an SAE that was considered to be related to durvalumab by the study investigator.

ImAEs: immune-mediated pneumonitis, hepatitis, colitis, hypothyroidism, hyperthyroidism, adrenal insufficiency, Type 1 diabetes mellitus, hypophysitis/hypopituitarism, nephritis, rash and pemphigoid. Other immune-mediated adverse reactions including myocarditis and myositis/polymyositis. These imAEs should

be managed based on dosing and Toxicity Management Guidelines described in appendix 1 of this protocol.

Infusion-related reactions: as with the administration of any Ig, infusion-related reactions may occur and may be severe and should be managed according to Toxicity Management Guidelines and study protocol.

As patients with previous history of bleeding and/or who are taking anticoagulant medications may have a higher risk of subsequent bleeding, the investigator should devote special attention to monitoring of these patients in the context of possible bleeding.

Further information on these risks can be found in the current version of the durvalumab IB.

In monotherapy clinical studies AEs (all grades) reported very commonly (≥10% of patients) are fatigue, cough , decreased appetite , dyspnoea , nausea , constipation , diarrhoea , pyrexia , back pain, anaemia, vomiting, pruritus, arthralgia, headache, asthenia, oedema peripheral and rash.

A total of 178 patients (9.4%) discontinued from study treatment due to an AE. A total of 108 patients (5.7%) had serious AEs (SAEs) that were considered by the investigator as related to durvalumab.

Most treatment-related AEs were manageable by delaying or interrupting the durvalumab dose, symptomatic treatment, and in the case of events suspected to have an immune basis, the use of established treatment guidelines for immune-mediated toxicity (see the Dosing Modification and Toxicity Management Guidelines).

Serious adverse reactions for durvalumab monotherapy considered expected

MedDRA (v21.1) SOC	PT	Number (%) of subjects exposed (N=3006)		
		Suspected SARs n (%) [a]	Occurrence of life-threatening suspected SARs n (%) [b]	Occurrence of fatal suspected SARs n (%) [b]
Respiratory, thoracic, and mediastinal disorders	Pneumonitis	28 (0.9)	0	0
	Interstitial lung disease	7 (0.2)	0	0
Injury, poisoning, and procedural complications	Infusion related reaction	6 (0.2)	0	0
Infections and infestations	Pneumonia	7 (0.2)	0	0
	Pneumocystis jirovecii pneumonia	2 (<0.1)	0	0
	Lung infection	2 (<0.1)	0	0
Gastrointestinal disorders	Colitis	4 (0.1)	0	0
	Diarrhoea	6 (0.2)	0	0
	Abdominal pain	2 (<0.1)	0	0
Endocrine disorders	Adrenal insufficiency	3 (<0.1)	0	0
	Hyperthyroidism	2 (<0.1)	0	0
	Hypopituitarism	2 (<0.1)	0	0
	Hypothyroidism	3 (<0.1)	0	0
Hepatobiliary disorders	Hepatitis	2 (<0.1)	0	0
	Autoimmune hepatitis	2 (<0.1)	0	0
Investigations	AST increased	2 (<0.1)	0	0
	ALT increased	2 (<0.1)	0	0

MedDRA (v19v21.1) SOC	PT	Number (%) of subjects exposed (N=18893006)		
		Suspected SARs n (%) [a]	Occurrence of life-threatening suspected SARs n (%) [b]	Occurrence of fatal suspected SARs n (%) [b]
Renal and urinary disorders	Nephritis	2 (<0.1)	0	0

Pooled dataset from studies CD-ON-MEDI4736-1108 (DCO 16 October 2017), ATLANTIC (DCO 3 June 2016) and , MYSTIC, EAGLE, ARCTIC, CONDOR, HAWK, PACIFIC (DCO 22 March 2018), and D4190C00002.

[a] n = number of subjectspatients who have experienced the SAR.

[b] All fatal and all life-threatening events for durvalumab monotherapy are considered unexpected for reporting purposes and are excluded from this table.

AST = aspartate aminotransferase; DCO = data cut-off; MedDRA = Medical Dictionary for Regulatory Activities; PT = preferred term; SAR = serious adverse reaction; SOC = system organ class.

The majority of treatment-related AEs were manageable with dose delays, symptomatic treatment, and in the case of events suspected to have an immune basis, the use of established treatment guidelines for immune-mediated toxicity (please see Appendix 1)

A detailed summary of durvalumab monotherapy AE data can be found in the current version of the durvalumab IB.

1.4.2.2 Tremelimumab

Potential risks, based on the mechanism of action of tremelimumab and related molecules (ipilimumab) include potentially immune-mediated gastrointestinal (GI) events including enterocolitis,, abdominal pain, dehydration, nausea and vomiting, and decreased appetite (anorexia); dermatitis including urticaria, skin exfoliation, and dry skin; endocrinopathies including hypophysitis, adrenal insufficiency, and hyperthyroidism and hypothyroidism; pancreatitis including autoimmune pancreatitis and lipase and amylase elevation; respiratory tract events including pneumonitis and interstitial lung disease (ILD); nervous system events including encephalitis, peripheral motor and sensory neuropathies, and Guillain-Barré syndrome; cytopenias including thrombocytopenia, anemia, and neutropenia; infusion-related reactions; anaphylaxis; and serious allergic reactions. The profile of AEs and the spectrum of event severity have remained stable across the tremelimumab clinical program and are consistent with the pharmacology of the target. To date, no tumor type or stage appears to be associated with unique AEs (except for vitiligo that appears to be confined to patients with melanoma). Overall, 944 of the 973 patients (97.0%) treated with tremelimumab monotherapy as of the data cutoff date of 1 November 2015 (not including 569 patients who have been treated in the ongoing blinded Phase IIb Study D4880C00003) experienced at least 1 AE. The events resulted in discontinuation of tremelimumab in 10.0% of patients, were serious in 36.5%, were Grade ≥3 in severity in 49.8%, were fatal in 67.7%, and were considered to be treatment related in 79.1% of patients. The frequency of any AEs and Grade ≥3 AEs was generally similar across the tremelimumab dose groups. However, a higher percentage of patients in the 10 mg/kg every 28 days and 15 mg/kg every 90 days groups compared with the All Doses <10 mg/kg group experienced treatment-related AEs, SAEs, AEs resulting in discontinuation of investigational product (IP), and deaths.

Serious adverse reactions for the tremelimumab monotherapy pool considered expected

MedDRA (v22.0) SOC	PT	Number (%) of subjects exposed Tremelimumab RSI pool (N=1665)		
		Suspected SARs [a] n (%)	Occurrence of life-threatening suspected SARs [b] n (%)	Occurrence of fatal suspected SARs [b] n (%)
Endocrine disorders	Hypophysitis	13 (0.8)	0	0
	Adrenal insufficiency	7 (0.4)	0	0
	Hyperthyroidism	5 (0.3)	0	0
	Hypothyroidism	4 (0.2)	0	0
	Lipase increased	2 (0.1)	0	0
Gastrointestinal disorders	Diarrhoea	183 (11.0)	0	0
	Colitis	68 (4.1)	0	0
	Enterocolitis	4 (0.2)	0	0
Hepatobiliary disorders	Autoimmune hepatitis	2 (0.1)	0	0
Skin and subcutaneous tissue disorders	Rash	8 (0.5)	0	0
	Pruritus	2 (0.1)	0	0

Includes legacy studies (A3671002, A3671008, A3671009, A3671022, A3671001, A3671011, A3671014, A3671015) and D4880C00003, D4880C00010, D4881C00024, D4884C00001, D4193C00003, D4190C00022 and D4191C00004. n = Number of subjects who have experienced the SAR.

[a] n = number of subjects who have experienced the SAR.

[b] All fatal and all life-threatening events for tremelimumab monotherapy are considered unexpected for reporting purposes.

ADR Adverse drug reaction; DCO Data cut-off; MedDRA Medical Dictionary for Regulatory Activities; PT Preferred term; RSI Reference safety information; SAR Serious adverse reaction; SOC System organ class.

A detailed summary of tremelimumab monotherapy AE data can be found in the current version of the tremelimumab IB.

1.4.2.3 Durvalumab + tremelimumab

Safety data have been pooled for 5 durvalumab and tremelimumab combination studies (D4190C00002, D4190C00006, D4190C00010, D4190C00011 and D4193C00003 [CONDOR]) for patients who received a dose of 20 mg/kg Q4W durvalumab plus 1 mg/kg tremelimumab Q4W, or equivalent durvalumab 10 mg/kg Q2W + tremelimumab 1 mg/kg Q4W; a total of 1088 patients are included in the pooled data set with a DCO of the 12 July 2017.

In durvalumab+tremelimumab combination studies at the dose of durvalumab 20 mg/kg and tremelimumab 1 mg/kg AEs (all grades) reported very commonly (≥ 10% of patients) are cough/productive cough, alteration of hepatic enzymes ALT/AST, diarrhoea, abdominal pain, hypothyroidism, rash, pruritus, pyrexia, peripheral oedema, and upper respiratory tract infections-

Approximately 15% of patients experienced an AE that resulted in permanent discontinuation of study drug and approximately 15% of patients experienced an SAE that was considered to be related to durvalumab and tremelimumab by the study investigator.

- Potential imAEs for durvalumab and durvalumab in combination with tremelimumab include:
    - immune-mediated pneumonitis, hepatitis, diarrhoea/colitis, intestinal perforation, hypothyroidism, adrenal insufficiency, hypophysitis/hypopituitarism, type I diabetes mellitus, nephritis, rash/dermatitis (including pemphigoid)
    - Pancreatitis
    - Other rare or less frequent events with a potential immune-mediated aetiology, eg, pericarditis, sarcoidosis, uveitis, and other events involving the eye (eg, keratitis and optic neuritis), skin (eg, scleroderma and vitiligo), and haematological (eg, haemolytic anaemia and immune thrombocytopenic purpura), rheumatological events (polymyalgia rheumatic and autoimmune arthritis) and neuropathy/neuromuscular toxicities (eg, myasthenia gravis, Guillain Barre syndrome).
- Hypersensitivity reactions including
    - Anaphylaxis and allergic reaction
    - Cytokine release syndrome
    - Immune complex disease
- Other infections

Infusion-related reactions: as with the administration of any Ig, infusion-related reactions may occur and may be severe and should be managed according to Toxicity Management Guidelines and study protocol.

Serious adverse reactions for durvalumab plus tremelimumab combination considered expected.

MedDRA (v23.0) SOC	PT	Number (%) of subjects exposed (N=2068)		
		Suspected SARs n (%) [a]	Occurrence of life-threatening suspected SARs n (%) [b]	Occurrence of fatal suspected SARs n (%) [b]
Gastrointestinal disorders	Colitis	40 (1.9)	0	0
	Diarrhoea	47 (2.3)	0	0
	Lipase increased	7 (0.3)	0	0
	Autoimmune colitis	5 (0.2)	0	0
	Abdominal pain	4 (0.2)	0	0
	Enterocolitis	6 (0.3)	0	0
	Amylase increased	3 (0.1)	0	0
Respiratory, thoracic, and mediastinal disorders	Pneumonitis	29 (1.4)	0	0
	Interstitial lung disease	9 (0.4)	0	0
Endocrine disorders	Adrenal insufficiency	14 (0.7)	0	0
	Hyperthyroidism	4 (0.2)	0	0
	Hypopituitarism	5 (0.2)	0	0
	Hypophysitis	4 (0.2)	0	0
General disorders and administration site conditions	Pyrexia	7 (0.3)	0	0
	Oedema peripheral	2 (<0.1)	0	0
Hepatobiliary disorders	Autoimmune hepatitis	5 (0.2)	0	0
	Hepatitis	4 (0.2)	0	0
Infections and infestations	Pneumonia	9 (0.4)	0	0
Cardiac disorders	Myocarditis	2 (<0.1)	0	0
Injury, poisoning, and procedural complications	Infusion related reaction	3 (0.1)	0	0
Renal and Urinary disorders	Nephritis	2 (<0.1)	0	0
Skin and subcutaneous tissue disorders	Pemphigoid	2 (<0.1)	0	0
Investigations	AST increased	5 (0.2)	0	0
	ALT increased	2 (<0.1)	0	0
	Transaminases increased	2 (<0.1)	0	0

Pooled dataset from studies D4190C00002, D4190C00006, D4190C00010, D4190C00011, D4190C00021, D4190C00022, ARCTIC, EAGLE, MYSTIC, and CONDOR.

[a] n = number of subjects who have experienced the SAR.

[b] All fatal and all life-threatening events for durvalumab + tremelimumab are considered unexpected for reporting purposes and are excluded from this table.

ALT = alanine aminotransferase; AST = aspartate aminotransferase; DCO = data cut-off; MedDRA = Medical Dictionary for Regulatory Activities; PT = preferred term; SAR = serious adverse reaction; SOC = system organ class.

A detailed summary of durvalumab + tremelimumab combination AE data can be found in the current version of the durvalumab IB.

1.4.2.4 Fixed Dosing for durvalumab and tremelimumab

A population PK model was developed for durvalumab using monotherapy data from a Phase 1 study (*study 1108; N=292; doses= 0.1 to 10 mg/kg Q2W or 15 mg/kg Q3W; solid tumors*). Population PK analysis indicated only minor impact of body weight (WT) on PK of durvalumab (coefficient of ≤ 0.5). The impact of body WT-based (10 mg/kg Q2W) and fixed dosing (750 mg Q2W) of durvalumab was evaluated by comparing predicted steady state PK concentrations (5th, median and 95th percentiles) using the population PK model. A fixed dose of 750 mg was selected to approximate 10 mg/kg (based on median body WT of ~75 kg). A total of 1000 patients were simulated using body WT distribution of 40–120 kg. Simulation results demonstrate that body WT-based and fixed dosing regimens yield similar median steady state PK concentrations with slightly less

overall between-subject variability with fixed dosing regimen.

Similarly, a population PK model was developed for tremelimumab using data from Phase 1 through Phase 3 (*N=654; doses= 0.01 to 15 mg/kg Q4W or Q90D; metastatic melanoma*) [Wang et al. 2014]. Population PK model indicated minor impact of body WT on PK of tremelimumab (coefficient of ≤ 0.5). The WT-based (1 mg/kg Q4W) and fixed dosing (75 mg/kg Q4W; based on median body WT of ~75 kg) regimens were compared using predicted PK concentrations (5th, median and 95th percentiles) using population PK model in a simulated population of 1000 patients with body weight distribution of 40 to 120 kg. Similar to durvalumab, simulations indicated that both body WT-based and fixed dosing regimens of tremelimumab yield similar median steady state PK concentrations with slightly less between-subject variability with fixed dosing regimen.

Similar findings have been reported by others [Ng et al 2006, Wang et al 2009, Zhang et al 2012, Narwal et al 2013]. Wang and colleagues investigated 12 monoclonal antibodies and found that fixed and body size-based dosing perform similarly, with fixed dosing being better for 7 of 12 antibodies. In addition, they investigated 18 therapeutic proteins and peptides and showed that fixed dosing performed better for 12 of 18 in terms of reducing the between-subject variability in pharmacokinetic/pharmacodynamics parameters [Zhang et al 2012].

A fixed dosing approach is preferred by the prescribing community due to ease of use and reduced dosing errors. Given expectation of similar pharmacokinetic exposure and variability, we considered it feasible to switch to fixed dosing regimens. Based on average body WT of 75 kg, a fixed dose of 750 mg Q2W MEDI4736 (equivalent to 10 mg/kg Q2W), 1500 mg Q4W durvalumab (equivalent to 20 mg/kg Q4W) and 75 mg Q4W tremelimumab (equivalent to 1 mg/kg Q4W) is included in the current study.

Fixed dosing of durvalumab and tremelimumab is recommended only for subjects with more or equal than 30kg body weight due to endotoxin exposure. Patients with a body weight less than 30 kg should be dosed using a weight-based dosing schedule.

2. STUDY OBJECTIVES

2.1 Primary objective(s)

The study will include patients with advanced neuroendocrine neoplasms in four different cohorts with the following primary endpoints:

Primary endpoint for cohorts 1, 2 and 3:

Nine-months clinical benefit rate (CBR) by Response Evaluation Criteria In Solid Tumors (RECIST 1.1), which is defined as the percentage of patients achieving complete response (CR), partial response (PR), or stable disease (SD) at month 9 after durvalumab plus tremelimumab was started.

Primary endpoint for cohort 4:

Nine-months overall survival rate, which is defined as the percentage of patients alive at month 9 after

durvalumab plus tremelimumab was started.

2.2 Secondary objective(s)

- Overall response rate (ORR) by irRECIST.
- To assess the duration of response according to irRECIST.
- To assess the median progression-free survival time (PFS) according to irRECIST.
- To assess the safety profile of Durvalumab and Tremelimumab in subjects with advanced neuroendocrine neoplasms.
- To assess the median overall survival (OS) time.
- To assess response status according to irRECIST at 6 and 12 months after start of study treatment.

2.3 Exploratory objective(s)

- To evaluate biochemical response (changes in CgA and NSE levels) and its association with response rate and progression-free survival.
- To assess whether baseline tumor and blood biomarkers may be predictive of response to durvalumab and tremelimumab therapy.
- To explore additional hypotheses related to biomarkers and relationship to durvalumab and tremelimumab efficacy and/or toxicity and neuroendocrine tumors evolution that may arise from internal or external research activities.

3. STUDY DESIGN

3.1 Overview of study design

This is a prospective, multi-center, open label, stratified, exploratory phase II study evaluating the efficacy and safety of durvalumab plus tremelimumab in different cohorts of patients with neuroendocrine neoplasms.

The study will include patients in four different cohorts:

- **Cohort 1**: Well-moderately differentiated lung neuroendocrine tumors (classically known as typical and atypical carcinoids) after progression to somatostatin analogs and one prior targeted therapy or chemotherapy.
- **Cohort 2**: G1/G2 (WHO grade 1 and 2) gastrointestinal neuroendocrine tumors after progression to somatostatin analogs and one prior targeted therapy.
- **Cohort 3**: G1/G2 (WHO grade 1 and 2) pancreatic neuroendocrine tumors after progression to standard therapies (chemotherapy, somatostatin analogs and target therapy), who have received between two and four prior lines of treatment.
- **Cohort 4**: Neuroendocrine neoplasms (WHO grade 3) of gastroenteropancreatic origin or unknown primary site (excluding lung primary tumors), patients will be treated in second line only, after

progression to first-line chemotherapy with a platinum based regimen.

The patient recruitment period of the study will last approximately 24 months. The maximum treatment period for each subject on study is anticipated to be approximately 12 months (13 administrations of durvalumab). However, subjects will continue on active follow-up to determine secondary endpoints of duration of response, median progression-free survival, median overall survival and safety profile.

This study will be conducted in 3 phases: a screening phase, a treatment phase stratified by tumor origin (four different cohort) and a follow up phase.

3.2 Study schema

Screening phase

Screening will occur between Day -28 and Day -1. The purpose of the screening period is to establish protocol eligibility. Informed consent will be obtained up to 4 weeks prior to Cycle 1 Day 1 and after the study has been fully explained to each subject and prior to the conduct of any screening procedures or assessments.

The purpose of the baseline visit is to establish disease characteristics prior to allocation and treatment and to confirm protocol eligibility as specified in the inclusion/exclusion criteria. Results of baseline assessments must be obtained prior to the first dose of study drug (Cycle 1/Day 1). Baseline assessments may be performed on Day -1 or on Cycle 1/Day 1 prior to dosing. Clinical laboratory tests including pregnancy test (where applicable) can be performed within 72 hours of the first dose of study drug. Subjects who complete the baseline visit and continue to meet the criteria for inclusion/exclusion will begin the treatment phase of this study.

Determinations performed by routine clinical practice before signing the informed consent will be valid, provided they are carried out following this protocol requirements and date of baseline TC is performed within the previous 4 weeks from treatment initiation. It should be clearly stated that patient consented her/his participation to ESR 15-11561-61–DUNE trial in patient record.

Treatment phase

The treatment phase will begin at the time of allocation of the first patient and will consist of the study treatment cycles. The treatment phase will end when the last patient discontinue the study drug.

The treatment phase will include two stages for each cohort. The first stage will dose the first patient in each cohort in a safety run period for one cycle (4 weeks). If no unexpected side effects are observed in the first patient treated in each cohort during the safety run period, recruitment will continue up to 31 patients for cohorts 1 to 3, and 33 for cohort 4.

Subjects will be allocated in each primary tumor cohort to receive durvalumab 1500 mg Q4W for 12 months plus tremelimumab 75 mg Q4W for up to 4 doses during the first 4 cycles of combined therapy. Cycles are defined by 4 weeks or 28 days. Subjects will undergo safety and efficacy assessment as defined per protocol.

If a toxicities pause occurs, cycles not administered for that toxicity can be delayed for a maximum of 4 weeks, after that time that cycle will be considered lost and the next cycle will be administered.
Remember no restarts are allowed after a delay of more than 12 weeks due to toxicity or more than 4 weeks of delay if **the reason is different from toxicity** without consulting the Coordinating Investigator.

Follow-up phase

Patients will finish the treatment phase after the administration of 12 months (13 cycles) of durvalumab or after disease progression during the treatment phase. For patients that complete all scheduled treatment, the follow-up phase will begin to determine survival endpoints. Tumor assessments will continue every 12 weeks

and clinical appointments will start monthly during the first 3 months of follow-up, every 2 months until first year, and continue every 6 months until disease progression.

3.3 Study Oversight for Safety Evaluation

A subject may elect to discontinue study drug at any time for safety, medical, or personal reasons. Patients who choose to discontinue study drug prior to disease progression will be followed in the post study treatment follow up period and continue to undergo regularly scheduled disease assessment until documentation of disease progression or start of an alternative anticancer treatment. All subjects who discontinue study drug will be followed for overall survival and all post progression cancer treatments administered will be recorded. Subjects may at any time withdraw consent for further study participation. No further data will be collected on subjects once consent has been withdrawn. The investigator will promptly explain to the subject involved that the study drug will be discontinued for that subject and provide appropriate medical treatment and other necessary measures for the subject. A subject who has ceased to return for visits will be followed up by mail, phone, or other means as much as possible to gather information such as the reason for failure to return and the status of treatment compliance, presence or absence of adverse events, and clinical courses of signs and symptoms, and the information will be recorded in the CRF.

Subjects who discontinue early from the study or treatment will be discontinued for 1 of these primary reasons: adverse event(s), lost to follow-up, subject choice, progressive disease, or administrative/other. In addition to the primary reason, the subject may have indicated 1 or more of these reasons as secondary reasons for discontinuation. Study disposition information will be collected on the appropriate CRF. A subject removed from the study for any reason may not be replaced.

Safety will be assessed by monitoring and recording all AEs including all CTCAE grades (for both increasing and decreasing severity) and serious adverse events (SAEs); regular monitoring of hematology, clinical chemistry, and urine values; physical examinations; and regular measurement of vital signs, and electrocardiograms (ECGs) as detailed in the schedule of visits and procedures.

4. PATIENT SELECTION, ENROLLMENT, ALLOCATION, RESTRICTIONS, DISCONTINUATION AND WITHDRAWAL

Approximately 126 patients will be enrolled and allocated in a balanced manner into either the four cohorts of patients at approximately 20 sites in Spain. Each patient must meet all of the inclusion criteria (Section 4.1) and none of the exclusion criteria (Section 4.2) for this study. Under no circumstances, will there be exceptions to this rule.

4.1 Inclusion criteria

For inclusion in the study, patients should fulfill the following criteria:

1. Written informed consent obtained from the subject prior to performing any protocol-related procedures.

2. Age > 18 years at time of study entry.

3. Subjects must have histologically confirmed diagnosis of one of the following advanced/metastatic neuroendocrine tumor types:

   a) **Cohort 1:** Well-moderately differentiated neuroendocrine tumors of the lung (mitotic count ≤10 mitoses x 10 HPF), also known as typical and atypical lung carcinoids, that have progressed to prior somatostatin analog therapy and/or one prior targeted therapy or chemotherapy (only one prior

systemic therapy, with the exception of patients that have been treated with somatostatin analogues and other systemic treatment, when two prior treatments are allowed).

b) **Cohort 2:** Well-moderately differentiated neuroendocrine tumors G1/G2 (WHO grade 1 and 2) gastrointestinal neuroendocrine tumors after progression to somatostatin analogs and one targeted therapy (prior targeted therapy could be everolimus or a multikinase inhibitor). Prior therapies with interferon alpha-2b or radionucleotide therapy are allowed.

c) **Cohort 3:** Well-moderately differentiated neuroendocrine tumors G1/G2 (WHO grade 1 and 2) from pancreatic origin after progression to standard therapies (chemotherapy, somatostatin analogs and target therapy); patients must be treated with at least two prior systemic treatment lines and a maximum of four previous treatment lines.

d) **Cohort 4**: Neuroendocrine neoplasms (WHO grade 3) of gastroenteropancreatic origin or unknown primary site (excluding lung primary tumors), patients will be treated in second line only, after progression to first-line chemotherapy with a platinum based regimen. Patients will be treated in second line."

4. For patients included in cohorts 1, 2 and 3: WHO Classification G1/G2 (mitotic count ≤10 mitoses x 10 HPF) lung typical and atypical carcinoids for cohort 1, G1/G2 (Ki67≤20% or mitotic count ≤20 mitoses x 10 HPF) gastrointestinal for cohort 2 (including stomach, small intestine and colorectal origins), G1/G2 (Ki67≤20% or mitotic count ≤20 mitoses x 10 HPF) pancreatic for cohort 3.

5. For patients included in cohort 4: WHO classification G3 (Ki67≥20% or mitotic count >20 mitoses x 10 HPF) gastroenteropancreatic neuroendocrine carcinomas (NEC) or liver metastases of G3 NEC of unknown primary site.

6. Subjects must have evidence of measurable disease meeting the following criteria:

   a) In case of more than one target lesion, it should be identified at least 1 lesion of ≥ 1.0 cm in the longest diameter for a non lymph node, or ≥ 1.5 cm in the short-axis diameter for a lymph node, which is serially measurable according to RECIST 1.1 using computerized tomography/magnetic resonance imaging (CT/MRI). If there is only one target lesion and it is a non-lymph node, it should have a longest diameter of ≥ 1.5 cm.

   b) Lesions that have had external beam radiotherapy (EBRT) or loco-regional therapies such as radiofrequency (RF) ablation or liver embolization must show evidence of progressive disease based on RECIST 1.1 to be deemed a target lesion.

   c) Subjects must show evidence of disease progression by radiologic image techniques within 12 months (an additional month will be allowed to accommodate actual dates of performance of scans, i.e., within ≤ 13 months) prior to signing informed consent, according to RECIST 1.1.

7. Eastern Cooperative Oncology Group (ECOG) performance status of 0 or 1.

8. Life expectancy of at least 12 weeks.

9. Adequate normal organ and marrow function as defined below:

   - Haemoglobin ≥ 9.0 g/dL.

   - Absolute neutrophil count (ANC) ≥ 1.5 x 10^9/L (≥ 1500 per mm^3).

   - Platelet count ≥ 100 x 10^9/L (≥ 100,000 per mm^3).

10. Serum bilirubin ≤ 1.5 x institutional upper limit of normal (ULN). This will not apply to subjects with

confirmed Gilbert's syndrome (persistent or recurrent hyperbilirubinemia that is predominantly unconjugated in the absence of hemolysis or hepatic pathology), who will be allowed only in consultation with their physician.

11. AST (SGOT)/ALT (SGPT) ≤ 2.5 x institutional upper limit of normal unless liver metastases are present, in which case it must be ≤ 5x ULN.

12. Serum creatinine CL>40 mL/min by the Cockcroft-Gault formula (Cockcroft and Gault 1976) or by 24-hour urine collection for determination of creatinine clearance:

Males:

$$\text{Creatinine CL (mL/min)} = \frac{\text{Weight (kg)} \times (140 - \text{Age})}{72 \times \text{serum creatinine (mg/dL)}}$$

Females:

$$\text{Creatinine CL (mL/min)} = \frac{\text{Weight (kg)} \times (140 - \text{Age})}{72 \times \text{serum creatinine (mg/dL)}} \times 0.85$$

13. Female subjects must either be of non-reproductive potential (ie, post-menopausal by history: ≥60 years old and no menses for ≥1 year without an alternative medical cause; OR history of hysterectomy, OR history of bilateral tubal ligation, OR history of bilateral oophorectomy) or must have a negative serum pregnancy test upon study entry.

14. Subject is willing and able to comply with the protocol for the duration of the study including undergoing treatment and scheduled visits and examinations including follow up.

4.2 Exclusion criteria

Subjects who meet any of the following criteria will be excluded from this study:

1. Involvement in the planning and/or conduct of the study.

2. Participation in another clinical study with an investigational product during the last 4 weeks.

3. WHO Classification G3 neuroendocrine neoplasms of lung origin (oat cell/large cell lung cancer).

4. Prior treatment with anti-PDL-1/anti-PD-1 or anti-CTL4 therapy.

5. Uncontrolled intercurrent illness including, but not limited to, ongoing or active infection, symptomatic congestive heart failure, uncontrolled hypertension, unstable angina pectoris, cardiac arrhythmia, active peptic ulcer disease or gastritis, active bleeding diathesis including any subject known to have evidence of acute or chronic hepatitis B (e.g., HBsAg reactive), hepatitis C (e.g., HCV RNA [qualitative] is detected) or known history of Human Immunodeficiency Virus (HIV) (HIV 1/2 antibodies), or psychiatric illness/social situations that would limit compliance with study requirements or compromise the ability of the subject to give written informed consent.

6. Known history of previous clinical diagnosis of tuberculosis.

7. Current or prior use of immunosuppressive medication within 28 days before the first dose of durvalumab or tremelimumab, with the exceptions of intranasal and inhaled corticosteroids or systemic corticosteroids at physiological doses, which are not to exceed 10 mg/day of prednisone, or an equivalent corticosteroid.

8. Active or prior documented autoimmune disease within the past 2 years

NOTE: Subjects with vitiligo, Grave's disease, or psoriasis not requiring systemic treatment (within the past

2 years) are not excluded.

9. Active or prior documented inflammatory bowel disease (e.g., Crohn's disease, ulcerative colitis).

10. History of allogeneic organ transplant.

11. History of hypersensitivity to durvalumab, tremelimumab or any excipient.

12. Subjects having a diagnosis of immunodeficiency or are receiving systemic steroid therapy or any other form of immunosuppressive therapy within 28 days prior to the first dose of trial treatment.

13. Knowledge of active central nervous system (CNS) metastases and/or carcinomatous meningitis. Subjects with previously treated brain metastases may participate provided they have stable brain metastases [without evidence of progression by imaging confirmed [by magnetic resonance imaging (MRI) if MRI was used at prior imaging, or confirmed by computed tomography (CT) imaging if CT used at prior imaging] for at least four weeks prior to the first dose of trial treatment; also, any neurologic symptoms must have returned to baseline], have no evidence of new or enlarging brain metastases, and have not used steroids for brain metastases for at least 7 days prior to trial treatment. This exception does not include carcinomatous meningitis, as subjects with carcinomatous meningitis are excluded regardless of clinical stability.

14. Receipt of live attenuated vaccination within 30 days prior to study entry or within 30 days of receiving durvalumab or tremelimumab. Note: The killed virus vaccines used for seasonal influenza vaccines for injection are allowed; however intranasal influenza vaccines (e.g., FluMist®) are live attenuated vaccines, and are not allowed.

15. Subjects having known history of, or any evidence of interstitial lung disease or active, noninfectious pneumonitis.

16. Any prior Grade ≥3 immune-related adverse event (irAE) while receiving any previous immunotherapy agent, or any unresolved irAE >Grade 1.

17. Subjects who have received any anti-cancer treatment within 21 days or any investigational agent within 30 days prior to the first dose of study drug and should have recovered from any toxicity related to previous anti-cancer treatment. This does not apply to the use of somatostatin analogues for symptomatic therapy.

18. Major surgery within 3 weeks prior to the first dose of study drug.

19. Subjects having > 1+ proteinuria on urine dipstick testing will undergo 24h urine collection for quantitative assessment of proteinuria. Subjects with urine protein ≥ 1 g/24h will be ineligible.

20. Significant cardiovascular impairment: history of congestive heart failure greater than New York Heart Association (NYHA) Class II, unstable angina; myocardial infarction or stroke within 6 months of the first dose of study drug, or cardiac arrhythmia requiring medical treatment.

21. Mean QT interval corrected for heart rate (QTc) ≥470 ms calculated from 3 electrocardiograms (ECGs) using Fredericia's Correction.

22. Bleeding or thrombotic disorders or use of anticoagulants, such as warfarin, or similar agents requiring therapeutic international normalized ratio (INR) monitoring. Treatment with low molecular weight heparin (LMWH) is allowed.

23. Active hemoptysis (bright red blood of at least 0.5 teaspoon) within 3 weeks prior to the first dose of study drug.

24. Patients with tumoral disease in the head and neck region, such as paratracheal or periesophageal lymph node involvement, or with infiltration of structures in the digestive tract, or vascular pathways that represent a

risk of increased bleeding.

25. Patients of cohort 1 with neuroendocrine tumors of pulmonary origin or pulmonary metastases with evidence of active bleeding.

26. Patients with evidence of digestive bleeding.

27. Active infection (any infection requiring treatment).

28. Active malignancy (except for differentiated thyroid carcinoma, or definitively treated melanoma in-situ, basal or squamous cell carcinoma of the skin, or carcinoma in-situ of the cervix) within the past 24 months.

29. Female patients who are pregnant or breastfeeding or male or female patients of reproductive potential who are not willing to employ highly effective birth control from screening to 180 days after the last dose of durvalumab + tremelimumab combination therapy or 90 days after the last dose of durvalumab monotherapy, whichever is the longer time period.

30. Documented active alcohol or drug abuse.

31. Patients with a prior history of non-compliance with medical regimens.

32. Any condition that, in the opinion of the investigator, would interfere with evaluation of study treatment or interpretation of patient safety or study results.

4.3 Withdrawal of Subjects from Study Treatment and/or Study

Permanent discontinuation of investigational product/study treatment

An individual subject will not receive any further investigational product if any of the following occur in the subject in question:

1. Withdrawal of consent or lost to follow-up.

2. Adverse event that, in the opinion of the investigator or the sponsor, contraindicates further dosing.

3. Subject is determined to have met one or more of the exclusion criteria for study participation at study entry and continuing investigational therapy might constitute a safety risk.

4. Pregnancy or intent to become pregnant.

5. Any AE that meets criteria for discontinuation as defined in Section 10.3.

6. Grade ≥ 3 infusion reaction.

7. Subject noncompliance that, in the opinion of the investigator or sponsor, warrants withdrawal; eg, refusal to adhere to scheduled visits.

8. Initiation of alternative anticancer therapy including another investigational agent.

9. Confirmed PD by RECIST 1.1 (CT scan or MRI at 4-6 weeks that confirms progression) and investigator determination that the subject is no longer benefiting from treatment with durvalumab + tremelimumab. Patients with documented tumor progression by image (all time-points but especially at 12 weeks), the progression should be confirmed with an additional imaging test (same characteristics) within 4-6 weeks. Treatment should continue according to protocol, until disease progression is confirmed, according the aforementioned procedure.

10. No restarts are allowed after a delay of more than 12 weeks due to toxicity or more than 4 weeks of delay

if **the reason is different from toxicity** without consulting the Coordinating Investigator.

Subjects who are permanently discontinued from further receipt of investigational product, regardless of the reason (withdrawal of consent, due to an AE, other), will be identified as having permanently discontinued treatment.

Subjects who are permanently discontinued from receiving investigational product will be followed for safety per Section 10.0 and Appendix 1 or 2, including the collection of any protocol-specified blood specimens, unless consent is withdrawn or the subject is lost to follow-up or enrolled in another clinical study. All subjects will be followed for survival. Subjects who decline to return to the site for evaluations will be offered follow-up by phone every **3 months as an alternative**.

Withdrawal of consent

If consent is withdrawn, the subject will not receive any further investigational product or further study observation.

4.4 Replacement of subjects

If a patient withdraws from participation in the study, then his or her enrollment/allocation code cannot be reused. Withdrawn patients will not be replaced.

5. INVESTIGATIONAL PRODUCT(s)

5.1 Durvalumab and tremelimumab

The sponsor will supply durvalumab and tremelimumab to the site pharmacies as a solution for infusion after dilution.

5.1.1 Formulation/packaging/storage

The trial medication (i.e., Durvalumab and Tremelimumab) and its packaging will be labeled in accordance with annex 13 of EU to Good Manufacturing Practice.

Durvalumab

Durvalumab will be supplied as a 500-mg vial solution for infusion after dilution. The solution contains 50 mg/mL durvalumab, 26 mM histidine/histidine-hydrochloride, 275 mM trehalose dihydrate, and 0.02% (weight/volume) polysorbate 80; it has a pH of 6.0. The nominal fill volume is 10 mL. Investigational product vials are stored at 2°C to 8°C (36°F to 46°F) and must not be frozen. Durvalumab must be used within the individually assigned expiry date on the label.

Tremelimumab

Tremelimumab will be supplied as a 400-mg vial or 25 mg solution for infusion after dilution. The solution contains 20 mg/mL of tremelimumab, 20 mM histidine/histidine hydrochloride, 222 mM trehalose dihydrate, 0.02% (w/v) polysorbate 80, and 0.27 mM disodium edetate dihydrate (EDTA);it has a pH of 5.5.The nominal fill volume is 20 mL. Investigational product vials are stored at 2°C to 8°C (36°F to 46°F) and must not be

frozen. Tremelimumab must be used within the individually assigned expiry date on the label.

5.2 Dose and treatment regimens

5.2.1 Treatment regimen

Patients will receive 1500 mg durvalumab via IV infusion q4w for up to 4 doses/cycles and 75 mg tremelimumab via IV infusion q4w for up to 4 doses/cycles, and then continue 1500 mg durvalumab q4w starting on Week 16 for up to 8 months (9 doses). Dosing outside the window should be discussed with the Study Physician. Tremelimumab will be administered first. Durvalumab infusion will start approximately 1 hour after the end of tremelimumab infusion. The duration will be approximately 1 hour for each infusion. A 1-hour observation period is required after the first infusion of durvalumab and tremelimumab. If no clinically significant infusion reactions are observed during or after the first cycle, subsequent infusion observation periods can be at the Investigator's discretion (suggested 30 minutes after each durvalumab and tremelimumab infusion).

5.2.2 Duration of treatment and criteria for retreatment

Retreatment is allowed (once only) for patients meeting the retreatment criteria below. The same treatment guidelines followed during the initial 12-month treatment period will be followed during the retreatment period, including the same dose and frequency of treatments and the same schedule of assessments.

Patients receiving the combination of durvalumab and tremelimumab may undergo retreatment in 2 clinical scenarios, described below:

1. Patients who achieve and maintain disease control (ie, CR, PR, or SD) through to the end of the 12-month treatment period may restart treatment with the combination upon evidence of PD, with or without confirmation according to RECIST 1.1, during follow-up.

2. Patients who complete the 4 dosing cycles of the combination of durvalumab and tremelimumab portion of the regimen (with clinical benefit per Investigator judgment), but subsequently have evidence of PD during the durvalumab monotherapy portion of the combination regimen, with or without confirmation according to RECIST 1.1, may restart treatment with the combination.

Before restarting their assigned treatment, the Investigator should ensure that the patient:

1. Does not have any significant, unacceptable, or irreversible toxicities that indicate continuing treatment will not further benefit the patient.

2. Still fulfills the eligibility criteria for this study, including re-consenting to restart durvalumab and tremelimumab.

3. Has not had received an intervening systemic anticancer therapy after their assigned treatment discontinuation.

4. Has had a baseline tumor assessment within 28 days of restarting their assigned treatment; all further scans should occur with the same frequency as during the initial 12 months of treatment (relative to the date of randomization) until study treatment is stopped (maximum of 12 months of further treatment).

During the retreatment period, patients receiving durvalumab + tremelimumab may resume durvalumab dosing at 1500 mg q4w with 75 mg of tremelimumab q4w for 4 doses each. Patients will then continue with durvalumab monotherapy at 1500 mg q4w, beginning at Week 16, 4 weeks after the last dose of combination therapy (a total of 9 additional doses).

Treatment through progression is at the Investigator's discretion, and the Investigator should ensure that

patients do not have any significant, unacceptable, or irreversible toxicities that indicate that continuing treatment will not further benefit the patient. A patient with a confirmed progression receiving durvalumab + tremelimumab cannot continue therapy or obtain retreatment if dosing is ongoing in the combination portion of therapy (q4w dosing) and progression occurs in a target lesion that has previously shown a confirmed response.

Patients who the sponsor and/or the Investigator determine may not continue treatment will enter follow-up.

5.2.3 Study drug preparation of durvalumab and tremelimumab

Based on average body WT of 75 kg, a fixed dose of 750 mg Q2W durvalumab (equivalent to 10 mg/kg Q2W), 1500 mg Q4W durvalumab (equivalent to 20 mg/kg Q4W) and 75 mg Q4W tremelimumab (equivalent to 1 mg/kg Q4W) is included in the current study.

Preparation of durvalumab doses for administration with an IV bag

The dose of durvalumab for administration must be prepared by the Investigator's or site's designated IP manager using aseptic technique. Total time from needle puncture of the durvalumab vial to the start of administration should not exceed:

- 24 hours at 2°C to 8°C
- 4 hours at room temperature

If in-use storage time exceeds these limits, a new dose must be prepared from new vials. Infusion solutions must be allowed to equilibrate to room temperature prior to commencement of administration.

No incompatibilities between durvalumab and polyvinylchloride or polyolefin IV bags have been observed. Dose of 1500mg durvalumab for patients ≥30 kg will be administered using an IV bag containing 0.9% (w/v) saline (compatibility with dextrose has been established and may be used as a diluent option, if preferred), with a final durvalumab concentration ranging from 1 to 20 mg/mL, and delivered through an IV administration set with a 0.2- or 0.22-µm in-line filter. Remove 30.0 mL of IV solution from the IV bag prior to addition of durvalumab. Next, 30.0 mL of durvalumab (ie, 1500 mg of durvalumab) is added to the IV bag such that final concentration is within 1 to 20 mg/mL (IV bag volumes 100 to 1000 mL). Mix the bag by gently inverting to ensure homogeneity of the dose in the bag.

Patient weight at baseline should be used for dosing calculations unless there is a ≥10% change in weight. Dosing day weight can be used for dosing calculations instead of baseline weight per institutional standard.

For patients <30kg, Calculate the dose volume of durvalumab and tremelimumab and number of vials needed for the subject to achieve the accurate dose.

Durvalumab will be administered at room temperature (approximately 25°C) by controlled infusion via an infusion pump into a peripheral or central vein. Following preparation of durvalumab, the entire contents of the IV bag should be administered as an IV infusion over approximately 60 minutes (±5 minutes), using a 0.2, or 0.22-µm in-line filter. Less than 55 minutes is considered a deviation.

The IV line will be flushed with a volume of IV solution (0.9% [w/v] saline equal to the priming volume of the infusion set used after the contents of the IV bag are fully administered, or complete the infusion according to institutional policy to ensure the full dose is administered and document if the line was not flushed.

Standard infusion time is 1 hour. However, if there are interruptions during infusion, the total allowed time should not exceed 8 hours at room temperature. The table below summarizes time allowances and

temperatures.

Durvalumab hold and infusion times

Maximum time from needle puncture to start of administration	4 hours at room temperature, 24 hours at 2°C to 8°C
Maximum time for IV bag infusion, including interruptions	8 hours at room temperature

In the event that either preparation time or infusion time exceeds the time limits outlined in the table, a new dose must be prepared from new vials. Durvalumab does not contain preservatives, and any unused portion must be discarded.

Preparation of tremelimumab doses for administration with an IV bag

The dose of tremelimumab for administration must be prepared by the Investigator's or site's designated IP manager using aseptic technique. Total time from needle puncture of the tremelimumab vial to the start of administration should not exceed:

- 24 hours at 2°C to 8°C
- 4 hours at room temperature

It is recommended that the prepared final IV bag be stored in the dark at 2ºC-8°C until needed. If storage time exceeds these limits, a new dose must be prepared from new vials. The refrigerated infusion solutions in the prepared final IV bag should be equilibrated at room temperature for about 2 hours prior to administration. Tremelimumab does not contain preservatives and any unused portion must be discarded.

No incompatibilities between tremelimumab and polyvinylchloride or polyolefin IV bags have been observed. Doses of 75 mg tremelimumab for patients ≥30 kg will be administered using an IV bag containing 0.9% (w/v) saline, with a final tremelimumab concentration ranging from 0.1 mg/mL to 10 mg/mL, and delivered through an IV administration set with a 0.2 µm or 0.22 µm in-line filter. Remove 3.8 mL of IV solution from the IV bag prior to addition of tremelimumab. Next, 3.8 mL of tremelimumab (ie, 75 mg of tremelimumab) is added to the IV bag such that final concentration is within 0.1 mg/mL to 10 mg/mL (IV bag volumes 50 to 500 mL). Mix the bag by gently inverting to ensure homogeneity of the dose in the bag.

Patient weight at baseline should be used for dosing calculations unless there is a ≥10% change in weight. Dosing day weight can be used for dosing calculations instead of baseline weight per institutional standard.

For patients <30 kg, Calculate the dose volume for tremelimumab and number of vials needed for subject to achieve the accurate dose.

Tremelimumab will be administered at room temperature (approximately 25°C) by controlled infusion via an infusion pump into a peripheral or central vein. Following preparation of tremelimumab, the entire contents of the IV bag should be administered as an IV infusion over approximately 60 minutes (±5 minutes), using a 0.2, or 0.22-µm in-line filter. Less than 55 minutes is considered a deviation.

The IV line will be flushed with a volume of 0.9% (w/v) saline equal to the priming volume of the infusion set used after the contents of the IV bag are fully administered, or complete the infusion according to institutional policy to ensure the full dose is administered and document if the line was not flushed.

Standard infusion time is 1 hour. However, if there are interruptions during infusion, the total allowed time should not exceed 8 hours at room temperature. The table below summarizes time allowances and

temperatures.

Tremelimumab hold and infusion times

Maximum time from needle puncture to start of administration	4 hours at room temperature, 24 hours at 2ºC to 8ºC
Maximum time for IV bag infusion, including interruptions.	8 hours at room temperature

In the event that either preparation time or infusion time exceeds the time limits outlined in the table, a new dose must be prepared from new vials. Tremelimumab does not contain preservatives, and any unused portion must be discarded.

5.2.4 Monitoring of dose administration

Patients will be monitored during and after the infusion with assessment of vital signs at the times specified in the Study Protocol.

In the event of a ≤ Grade 2 infusion-related reaction, the infusion rate of study drug may be decreased by 50% or interrupted until resolution of the event and re-initiated at 50% of the initial rate until completion of the infusion. For patients with a ≤Grade 2 infusion-related reaction, subsequent infusions may be administered at 50% of the initial rate. Acetaminophen and/or an antihistamine (eg, diphenhydramine) or equivalent medications per institutional standard may be administered at the discretion of the investigator. If the infusion-related reaction is ≥Grade 3 or higher in severity, study drug will be discontinued.

As with any antibody, allergic reactions to dose administration are possible. Appropriate drugs and medical equipment to treat acute anaphylactic reactions must be immediately available, and study personnel must be trained to recognize and treat anaphylaxis. The study site must have immediate access to emergency resuscitation teams and equipment in addition to the ability to admit patients to an intensive care unit if necessary.

5.2.5 Accountability and dispensation

The trial medication will be sent to the investigator's site pharmacy preceded by the Regulatory Green Light. The medication is to be used exclusively in the clinical trial according to the instructions of this trial protocol.

When a drug shipment is received, the Investigator or designee will check the amount and condition of the delivery, drug expiration date, and sign the Receipt of Shipment Form provided. The Receipt of Shipment Form should be faxed or emailed to the CRO (see appendix 8). The original form will preliminarily be retained at the site and will be collected at the next monitoring visit by the monitor and stored in the Trial Master File at CRO. A copy remains in the Investigator File at the site. In case of shipment problems the Investigator or designee shall contact the CRA as soon as possible.

An Investigational Product Accountability Log will be provided for the trial medication. The record must be continuously updated and contain the dates, quantities and compounds of drugs received, medication identification number(s), the patient identification number to whom the trial medication was dispensed, date and quantity of medication dispense and the initials of the dispenser.

5.2.6 Disposition of unused investigational study drug

Trial medication will be monitored by the CRA at the respective hospital pharmacy prior to destruction after having completed a final inventory. Local or institutional regulations may require immediate destruction of the study drug used for safety reasons, e.g., cytotoxicity or to maintain the storage capacity and functionality of the storage at the site. In these cases, it may be acceptable to destroy it by the research staff, including partially used and empty vials, dispensed before a monitoring inspection, if the verification of original documents of empty boxes that indicate the information of batch number and dispensing date to the patient on

the label. This documentation will be verified against the quantity shipped, dispensed, returned and destroyed.

Prior to the destruction a final trial medication reconciliation statement must be completed. Drug supplies will be destroyed according to the legal requirements in Spain.

All trial medication inventory forms must be made available for inspection by a Sponsor authorized representative or designee and regulatory agency inspectors. The Investigator is responsible for the accountability of all used and unused study supplies at the site.

6. TREATMENT PLAN

6.1 Subject enrollment and allocation

6.1.1 Procedures for allocation

Patients will be allocated in one of the following cohorts regarding primary tumor site and WHO grading classification:

- Cohort 1: Well-moderately differentiated lung neuroendocrine tumors (classically known as typical and atypical carcinoids) after progression to somatostatin analogs and one prior targeted therapy or chemotherapy.
- Cohort 2: G1/G2 (WHO grade 1 and 2) gastrointestinal neuroendocrine tumors after progression to somatostatin analogs and one prior targeted therapy.
- Cohort 3: G1/G2 (WHO grade 1 and 2) pancreatic neuroendocrine tumors after progression to standard therapies (chemotherapy, somatostatin analogs and target therapy), who have received between two and four prior lines of treatment.
- Cohort 4: Neuroendocrine neoplasms (WHO grade 3) of gastroenteropancreatic origin or unknown primary site (excluding lung primary tumors) after progression to first-line chemotherapy with a platinum based regimen. Patients will be treated in second line.

6.1.2 Procedures for handling subjects incorrectly enrolled

Patients incorrectly enrolled in the trial will be considered as protocol deviations. If investigator considerer that the patient is obtaining benefit of the investigational treatment, patients could continue on therapy. They will not be considered for the primary endpoint of the trial, neither for efficacy nor biomarker secondary and exploratory analyses. Patients incorrectly enrolled that continue on treatment can be assessed for safety profile endpoint.

6.2 Dose Modification and Toxicity Management

6.2.1 Durvalumab and tremelimumab

For adverse events (AEs) that are considered at least partly due to administration of durvalumab and/or tremelimumab the following dose adjustment guidance may be applied:

- Treat each of the toxicities with maximum supportive care (including holding the agent suspected of causing the toxicity where required).
- If the symptoms promptly resolve with supportive care, consideration should be given to continuing the same dose of durvalumab or tremelimumab along with appropriate continuing supportive care. If medically appropriate, dose modifications are permitted for durvalumab and tremelimumab (see Appendix 1).
- All dose modifications should be documented with clear reasoning and documentation of the approach taken.

In addition, there are certain circumstances in which durvalumab or tremelimumab should be permanently discontinued.

Following the first dose of durvalumab or tremelimumab, subsequent administration of durvalumab or tremelimumab can be modified based on toxicities observed (see Appendix 1).

Based on the mechanism of action of durvalumab or tremelimumab leading to T-cell activation and

proliferation, there is the possibility of observing immune related Adverse Events (irAEs) during the conduct of this study. Potential irAEs include immune-mediated enterocolitis, dermatitis, hepatitis/hepatotoxicity, endocrinopathy, pneumonitis, nephritis, pancreatitis and neuropathy or neurologic events. Subjects should be monitored for signs and symptoms of irAEs. In the absence of an alternate etiology (e.g., infection or PD) signs or symptoms of enterocolitis, dermatitis, hepatitis/hepatotoxicity, endocrinopathy, pneumonitis, nephritis, pancreatitis and neuropathy or neurologic events should be considered to be immune-related.

Dose modification recommendations and toxicity management guidelines for immune-mediated reactions, for infusion-related reactions, and for non-immune-mediated reactions are detailed in Appendix 1.

In addition, management guidelines for adverse events of special interest (AESIs) are detailed in Section 10.1.3. All toxicities will be graded according to NCI CTCAE v4.03.

7. RESTRICTIONS DURING THE STUDY AND CONCOMITANT TREATMENT(s)

7.1 Restrictions during the study

The following restrictions apply while the patient is receiving study treatment and for the specified times before and after:

- Female subjects of childbearing potential who are sexually active with a non-sterilized male partner must use one highly effective method of contraception (Table 2) from the time of screening and must agree to continue using such precautions for 180 days after the last dose of durvalumab + tremelimumab combination therapy or 90 days after the last dose of durvalumab monotherapy, whichever is the longer time period. Male partners of a female subject must use male condom plus spermicide throughout this period. Cessation of birth control after this point should be discussed with a responsible physician. Not engaging in sexual activity for the total duration of the trial and the drug washout period is an acceptable practice; however, occasional abstinence, the rhythm method, and the withdrawal method are not acceptable methods of contraception..

- Females of childbearing potential are defined as those who are not surgically sterile (ie, bilateral tubal ligation, bilateral oophorectomy, or complete hysterectomy) or post-menopausal (defined 12 months with no menses without an alternative medical cause).

- Non-sterilized male subjects who are sexually active with a female partner of childbearing potential must use male condom plus spermicide from screening through 180 days after receipt of the final dose of durvalumab + tremelimumab combination therapy or 90 days after receipt of the final dose of durvalumab monotherapy, whichever is the longer time period. Female partners of a male subject must use a highly effective method of contraception throughout this period.

- Highly effective methods of contraception are described in Table 2. A highly effective method of contraception is defined as one that results in a low failure rate (i.e. less than 1% per year) when used consistently and correctly. Note that some contraception methods are not considered highly effective (e.g. male or female condom with or without spermicide; female cap, diaphragm, or sponge with or without spermicide; non-copper containing intrauterine device; progestogen-only oral hormonal contraceptive pills where inhibition of ovulation is not the primary mode of action [excluding Cerazette/desogestrel which is considered highly effective]; and triphasic combined oral contraceptive

pills).

- Restrictions relating to concomitant medications are described in Section 7.2.

Table 2. Highly Effective[a] Methods of Contraception

Barrier/ Intrauterine	Hormonal Methods
• Copper T intrauterine<br>• Levonorgestrel-relasing intrauterine system (eg, Mirema)[b]	• Etonogestrel implants: e.g. Implanon or Norplan<br>• Intravaginal device; e.g. ethinylestradiol and etonogestrel<br>• Medroxyprogesterone injection: e.g. Depo-Provera<br>• Normal and low dose combined oral contraceptive pill<br>• Norelgestromin/ethinylestradiol transdermal system<br>• Cerazette (desogestrel)

[a] Highly effective (i.e. failure rate of <1% per year) [b] This is also considered a hormonal method

Blood donation

Subjects should not donate blood while participating in this study, or for at least 90 days following the last infusion of durvalumab or tremelimumab, whichever occurs longest.

7.2 Concomitant treatment(s)

The Principal Investigator must be informed as soon as possible about any medication taken from the time of screening until the end of the clinical phase of the study (final study visit). Any concomitant medication(s), including herbal preparations, taken during the study will be recorded in the CRF.

Restricted, prohibited, and permitted concomitant medications are described in the following tables.

7.2.1 Permitted concomitant medications

Investigators may prescribe concomitant medications or treatments (e.g., acetaminophen, diphenhydramine) deemed necessary to provide adequate prophylactic or supportive care except for those medications identified as “excluded” as listed in Section 7.2.2.

Concomitant treatment with somatostatin analogues (octreotide or lanreotide) are allowed for hormonal symptom control.

7.2.2 Excluded Concomitant Medications

The following medications are considered exclusionary during the study.

1. Any investigational anticancer therapy other than the protocol specified therapies.
2. Any concurrent chemotherapy, radiotherapy (except palliative radiotherapy), immunotherapy, biologic or hormonal therapy for cancer treatment. Concurrent use of hormones for noncancer-related conditions (e.g., insulin for diabetes and hormone replacement therapy) is acceptable. NOTE: Local treatment of isolated lesions for palliative intent is acceptable (e.g., by local surgery or radiotherapy).
3. Immunosuppressive medications including, but not limited to systemic corticosteroids at doses not exceeding 10 mg/day of prednisone or equivalent, methotrexate, azathioprine, and TNF-α blockers. Use of immunosuppressive medications for the management of investigational product-related AEs or in subjects with contrast allergies is acceptable. In addition, use of inhaled and intranasal corticosteroids is permitted. A

temporary period of steroids will be allowed for different indications, at the discretion of the principal investigator (e.g., chronic obstructive pulmonary disease, radiation, nausea, etc).
4. Live attenuated vaccines within 30 days of durvalumab and tremelimumab dosing (ie, 30 days prior to the first dose, during treatment with durvalumab and tremelimumab for 30 days post discontinuation of durvalumab and tremelimumab. Inactivated vaccines, such as the injectable influenza vaccine, are permitted.

Table 3. Prohibited and Rescue Medications

Prohibited medication/class of drug	Usage
Additional investigational anticancer therapy concurrent with those under investigation in this study	Should not be given whilst the patient is on IP treatment
mAbs against CTLA-4, PD-1, or PD-L1	Should not be given whilst the patient is on IP treatment through 90 days after the last dose of IP.
Any concurrent chemotherapy, local therapy (except palliative radiotherapy for non-target lesions, eg, radiotherapy, surgery, radiofrequency ablation), biologic therapy, or hormonal therapy for cancer treatment	Should not be given whilst the patient is on IP treatment (including SoC). (Concurrent use of hormones for non-cancer-related conditions [eg, insulin for diabetes and hormone replacement therapy] is acceptable.)
Immunosuppressive medications, including, but not limited to, systemic corticosteroids at doses exceeding 10 mg/day of prednisone or its equivalent, methotrexate, azathioprine, and tumor necrosis factor α blockers	Should not be given whilst the patient is on IP treatment (including SoC). (Use of immunosuppressive medications for the management of IP-related AEs or in patients with contrast allergies is acceptable. In addition, use of inhaled, topical, and intranasal corticosteroids is permitted.
Live attenuated vaccines	Should not be given through 30 days after the last dose of IP (including SoC) during the study.
**Rescue/supportive medication/class of drug**	**Usage**
Concomitant medications or treatments (eg, acetaminophen or diphenhydramine) deemed necessary by the Investigator to provide adequate prophylactic or supportive care, except for those medications identified as "prohibited" as listed above.	To be administered as prescribed by the Investigator
Best supportive care (including antibiotics, nutritional support, growth factor support, correction of metabolic disorders, optimal symptom control, and pain management [including palliative radiotherapy, etc])	Should be used when necessary for all patients

8. STUDY PROCEDURES

8.1 Schedule of study procedures

Before study entry, throughout the study, and following study drug discontinuation, various clinical and diagnostic laboratory evaluations are outlined. The purpose of obtaining these detailed measurements is to ensure adequate safety and tolerability assessments. Clinical evaluations and laboratory studies may be repeated more frequently if clinically indicated. The Schedules of Assessments during the screening and treatment period is provided after the Protocol Synopsis.

8.1.1 Screening Phase

Screening procedures will be performed up to 28 days before Day 1, unless otherwise specified. All subjects must first read, understand, and sign the IEC-approved ICF before any study-specific screening procedures are performed. After signing the ICF, completing all screening procedures, and being deemed eligible for entry, subjects will be enrolled in the study. Procedures performed prior to the signing of the ICF are considered

standard of care and may be used as screening assessments if they fall within the 28-day screening window.

The following procedures will be performed during the Screening Visit:

- Informed Consent
- Review of eligibility criteria
- Medical history and demographics
- Complete physical exam
- ECOG Performance Status
- Vital signs, weight and height
- 12-lead ECG (in triplicate [2-5 minutes apart])
- Review of prior/concomitant medications
- Record any Aes or SAEs (since ICF signature)
- Imaging by CT/MRI
- Review of octreoscan/PET (up to 6 months prior inclusion) only on cohorts 1 to 3.
- Collect blood samples for biomarkers analysis
- Archival tumor block or slides
- Clinical laboratory tests for:
    - Hematology (see Table 4)
    - Clinical chemistry (see Table 5)
    - TSH, fT3 and fT4
    - Coagulation (PT, PTT, INR)
    - Creatinine Clearance
    - Serum pregnancy test (for women of childbearing potential only)
    - Hepatitis and HIV serologies
    - Urinalysis (see Table 6)
    - Disease-specific tumor markers

8.1.2 Treatment Phase

Procedures to be conducted during the treatment phase of the study are presented in the Schedule of Assessments. Screening procedures performed within 72 hours of Cycle 1 Day 1 (C1D1) do not need to be repeated on C1D1.

Efforts should be made to conduct study visits on the day scheduled (± 1 day). Clinical laboratory assessments may be conducted anytime within 72 hours prior to the scheduled visit, unless otherwise specified in the Schedule of Visits and Procedures. Results of the laboratory assessments should be reviewed always prior each

cycle dose administration. Whenever possible, subjects should be evaluated at approximately the same time of the day (e.g., morning or afternoon) at each visit, and reasonable efforts should be made to conduct all evaluations in the same test order at each visit.

Tumor assessments should be performed at time points indicated in the Schedule of Visits and Procedures.

Cycle 1/Day 1

- Obtain vital signs (resting BP, HR, RR, body temperature) and weight.
- Physical examination is not mandatory if performed at baseline (day -1) however a symptom-directed physical examination will be performed on Cycle 1/Day 1 and at any time during the study, as clinically indicated.
- Administer study drug:
  - Record all concomitant medication use.
  - Record any AEs or SAEs.

Cycle 1/Day 15

- Obtain vital signs (resting BP, HR, RR, body temperature) and weight.
- Evaluate ECOG performance status.
- Perform a comprehensive physical examination (including a neurological evaluation). A symptom-directed physical examination will be performed at any time during the study, as clinically indicated.
- Collect blood samples for biochemistry and hematology analyses (see Tables 4 and 5), including TSH, fT3 and fT4.
- Urinalysis (see Table 6)
- Record all concomitant medication use.
- Record any AEs or SAEs.

Cycle 2/Day 1

- Evaluate ECOG performance status.
- Obtain vital signs (supine BP, HR, RR, body temperature) and weight.
- Perform a comprehensive physical examination (including a neurological evaluation). A symptom-directed physical examination will be performed at any time during the study, as clinically indicated.
- Collect blood samples for biochemistry and hematology analyses (see Tables 4 and 5), including TSH,

fT3 and fT4.

- Urinalysis (see Table 6)
- Administer study drug.
- Collect blood samples for biomarker analysis.
- Record all concomitant medication use.
- Record any AEs or SAEs.
- Record survival data.

Cycle 2/Day 15

- Evaluate ECOG performance status.
- Obtain vital signs (supine BP, HR, RR, body temperature) and weight.
- Perform a comprehensive physical examination (including a neurological evaluation). A symptom-directed physical examination will be performed at any time during the study, as clinically indicated.
- Collect blood samples for biochemistry and hematology analyses (see Tables 4 and 5), including TSH, fT3 and fT4.
- Urinalysis (see Table 6)
- Record all concomitant medication use.
- Record any AEs or SAEs.

Cycle 3/Day 1

- Evaluate ECOG performance status.
- Obtain vital signs (supine BP, HR, RR, body temperature) and weight.
- Perform a comprehensive physical examination (including a neurological evaluation). A symptom-directed physical examination will be performed at any time during the study, as clinically indicated.
- Collect blood samples for biochemistry and hematology analyses (see Tables 4 and 5), including TSH,

fT3 and fT4.

- Urinalysis (see Table 6)
- Administer study drugs.
- Record all concomitant medication use.
- Record any AEs or SAEs.
- Record Survival data

Cycle 3/Day 15

- Evaluate ECOG performance status.
- Obtain vital signs (supine BP, HR, RR, body temperature) and weight.
- Perform a comprehensive physical examination (including a neurological evaluation). A symptom-directed physical examination will be performed at any time during the study, as clinically indicated.
- Collect blood samples for biochemistry and hematology analyses (see Tables 4 and 5), including TSH, fT3 and fT4.
- Urinalysis (see Table 6)
- Record all concomitant medication use.
- Record any AEs or SAEs.

Cycle 4 Through Last Cycle (cycle 12)/Day 1

- Evaluate ECOG performance status.
- Obtain vital signs (supine BP, HR, RR, body temperature) and weight.
- Perform a comprehensive physical examination (including a neurological evaluation). A symptom-directed physical examination will be performed at any time during the study, as clinically indicated.
- Collect blood samples for biochemistry and hematology analyses (see Tables 4 and 5), including TSH, fT3 and fT4
- Urinalysis (see Table 6)
- Administer study drugs. (from C5D1 to C12D1 tremelimumab no longer administered, only durvalumab).
- Record all concomitant medication use.
- Record any AEs or SAEs.
- Record survival data.
- Tumor assessment every 12 weeks until confirmed PD.

8.1.3 End of Treatment

End of treatment is defined as the last planned dosing visit within the 12-month dosing period. For subjects who discontinue durvalumab or tremelimumab prior to 12 months, end of treatment is considered the last visit

where the decision is made to discontinue treatment. All required procedures may be completed within ± 7 days of the end of treatment visit. Repeat disease assessment is not required if performed within 28 days prior to the end of treatment visit.

Blood samples for biomarker analysis are collected.

Assessments for subjects who have completed durvalumab and tremelimumab treatment and achieved disease control, or have discontinued durvalumab or tremelimumab due to toxicity in the absence of confirmed progressive disease are provided in APPENDIX 2.

Assessments for subjects who have discontinued durvalumab or tremelimumab treatment due to confirmed PD are presented in APPENDIX 3.

All subjects will be followed for survival until the end of the study regardless of further treatments, or until the sponsor ends the study.

Follow-up period

TC/MRI every 12 weeks until progression. Monthly visits during the first 3 months after end of treatment, every 2 months until one year, and therefore every 6 months. Determinations to be done are listed in appendix 2 and 3.

8.2 Description of study procedures

8.2.1 Medical history and physical examination, electrocardiogram, weight and vital signs

Findings from medical history (obtained at screening) and physical examination shall be given a baseline grade according to the procedure for AEs. Increases in severity of pre-existing conditions during the study will be considered AEs, with resolution occurring when the grade returns to the pre-study grade or below.

Physical examinations will be performed on study days noted in the Schedule of Assessments.

8.2.2 Physical examination

Physical examinations will be performed according to the assessment schedule. Full physical examinations will include assessments of the head, eyes, ears, nose, and throat and the respiratory, cardiovascular, GI, urogenital, musculoskeletal, neurological, dermatological, hematologic/lymphatic, and endocrine systems. Height will be measured at Screening only. Targeted physical examinations are to be used by the Investigator on the basis of clinical observations and symptomatology. Situations in which physical examination results should be reported as AEs are described in Section 10.

8.2.3 Electrocardiograms

Resting 12-lead ECGs will be recorded at screening and as clinically indicated throughout the study. ECGs should be obtained after the patient has been in a supine position for 5 minutes and recorded while the patient remains in that position.

At Screening, a single ECG will be obtained on which QTcF must be <470 ms.

In case of clinically significant ECG abnormalities, including a QTcF value >470 ms, 2 additional 12-lead ECGs should be obtained over a brief period (eg, 30 minutes) to confirm the finding.

Situations in which ECG results should be reported as AEs are described in Section 10.0.

8.2.4 Vital signs

Vital signs (blood pressure [BP], pulse, temperature, and respiration rate) will be evaluated according to the

assessment schedules.

On infusion days, patients receiving durvalumab + tremelimumab treatment will be monitored during and after infusion of IP as presented in the bulleted list below.

Supine BP will be measured using a semi-automatic BP recording device with an appropriate cuff size, after the patient has rested for at least 5 minutes. BP and pulse will be collected from patients receiving durvalumab + tremelimumab treatment before, during, and after each infusion at the following times (based on a 60-minute infusion):

Prior to the beginning of the infusion (measured once from approximately 30 minutes before up to 0 minutes [ie, the beginning of the infusion]).

Approximately 30 minutes during the infusion (**halfway** through infusion).

At the end of the infusion (approximately 60 minutes ±5 minutes).

A 1-hour observation period is required after the first infusion of durvalumab and tremelimumab.

If no clinically significant infusion reactions are observed during or after the first cycle, subsequent infusion observation periods can be at the Investigator’s discretion (suggested 30 minutes after each durvalumab and tremelimumab infusion).

If the infusion takes longer than 60 minutes, then BP and pulse measurements should follow the principles as described above or be taken more frequently if clinically indicated. The date and time of collection and measurement will be recorded on the appropriate eCRF. Additional monitoring with assessment of vital signs is at the discretion of the Investigator per standard clinical practice or as clinically indicated.

Body weight is also recorded along with vital signs.

Situations in which vital signs results should be reported as AEs are described in Section 10.3. A complete physical examination will be performed and will include an assessment of the following (as clinically indicated): general appearance, respiratory, cardiovascular, abdominal, skin, head and neck (including ears, eyes, nose and throat), lymph nodes, thyroid, musculoskeletal (including spine and extremities), genital/rectal, and neurological systems and at screening only, height.

8.2.5 Clinical laboratory tests

The following clinical laboratory tests will be performed in local hospitals (see the Schedule of Assessments):

- Coagulation parameters: Activated partial thromboplastin time and International normalised ratio to

be assessed at baseline and as clinically indicated

- Pregnancy test (female subjects of childbearing potential only)
  - Urine human chorionic gonadotropin
  - Serum beta-human chorionic gonadotropin (at screening only)
- Thyroid Stimulating Hormone
  - Free T3 and free T4 only if TSH is abnormal
- Other laboratory tests
  - Hepatitis B surface antigen, hepatitis C antibody
  - HIV antibody

Table 4. Hematology Laboratory Tests

Basophils	Mean corpuscular volume	Total white cell count
Eosinophils	Monocytes	Mean corpuscular hemoglobin concentration
Hematocrit	Neutrophils	Mean corpuscular hemoglobin
Hemoglobin	Platelet count	
Lymphocytes	Red blood cell count	

Table 5. Clinical chemistry (Serum or Plasma) Laboratory Tests

Albumin	Glucose
Alkaline phosphatase	Lactate dehydrogenase
Alanine aminotransferase	Lipase
Amylase	Magnesium
Aspartate aminotransferase	NSE (Enolase)
Bicarbonate	Potassium
Calcium	Sodium
CgA (Chromogranine)	Total bilirubin[a]
Chloride	Total protein
Creatinine	Urea or blood urea nitrogen, depending on local practice
Gamma glutamyltransferase[b]	Uric acid

[a] If Total bilirubin is ≥2xULN (and no evidence of Gilbert's syndrome) then fraction into direct and indirect bilirubin
[b] At baseline and as clinically indicated.

Table 6. Urinalysis Tests [a]

Bilirubin	pH
Blood	Protein
Glucose	Specific gravity
Ketones	Colour and appearance

[a] Microscopy should be used as appropriate to investigate white blood cells and use the high power field for red blood cells

8.3 Biological sampling procedures

Blood samples for the development of exploratory predictive biomarkers will be collected from all subjects prior to the first dose of study drug, and on Cycle 2/Day 1 and at end-of treatment visit or after documented disease progression, whichever occurs first. Biomarker discovery and validation will be performed to identify blood or tumor biomarkers which may be useful to predict subject response, as determined by evaluation of primary or secondary efficacy endpoints. Plasma samples from study subjects will undergo global proteomic and/or enzyme-linked immunosorbent assay (ELISA)-based analyses or multiplex bead-based immunoassay in an effort to identify protein biomarkers. In addition, DNA and RNA analyses will be performed in search of predictive or prognostic biomarkers and also biomarkers identified in other durvalumab+trametinib clinical

studies may also be assessed in samples collected from subjects enrolled in this study.

Archived, fixed tumor tissue from will be collected for all subjects for confirmation of histology and assessment of somatic mutations of genes which may be important in the development and progression of neuroendocrine tumors. Gene-expression profiling (GEP), proteomic, or immunohistochemical (IHC) analysis will be performed based on the amount of tumor tissue available for analysis. All analyses will be limited to correlations relevant to neuroendocrine tumors and clinical outcomes related to treatment with durvalumab and tremelimumab.

Blood and tumor samples collected during the study will be stored at the study sites until the initial primary efficacy and safety analyses of the study will be completed and results will be available. Then, the study Sponsor will decide whether to perform all or part of the pharmacogenetic/pharmacogenomics assessments.

Data obtained will only be used for research, to assist in developing safer and more effective treatments, and will not be used to change the diagnosis of the subject or alter the therapy of the subject. Any DNA derived from the sample may be stored for up to 15 years to assist in any research scientific questions related to durvalumab or tremelimumab. Sample collection will be registered in Spanish National Registry and will be used in research projects in the particular research line foresee in the informed consent. Samples cannot be transferred to third parties, and studies in the future with these samples will require EC approval. Instructions for the processing, storage, and shipping of samples will be provided in the Laboratory Manual.

8.3.1 Biomarker/Pharmacodynamic sampling and evaluation methods

PD-L1 Testing

Retrospectively, PD-L1 assessment will be performed using Ventana SP263 assay in accordance with the package insert on the Ventana Benchmark platform (Ultra or XT) within the GETNE translational working group to ensure comparability of data across all studies of durvalumab and/or tremelimumab.

Sample collection for PD-L1 testing

- The preferred tumor sample for the determination of a patient's PD-L1 status is the one taken following the completion of the most recent prior line of therapy. Samples taken at this time reflect the current PD-L1 status of the tumor and considered clinically most relevant.
- The preferred sample for PD-L1 testing was less than or equal to 3 months old. In cases where a sample a less than 3 months old was not available, patients were asked to undergo a new biopsy if considered clinically appropriate by their treating physician.
- Samples should be collected via a core needle of 18 gauge or larger or be collected by an incisional or excisional tumor biopsy. The provision of 2 cores is advised in order to provide sufficient tissue for PD-L1 assessment and processed to FFPE in a single block. Where institutional practice uses a smaller gauge needle, samples should be evaluated for tumor cell quantity (i.e. >100 tumor cells) to allow for adequate PD-L1 immunohistochemistry analyses.
- When the collection of a new sample is not clinically appropriate, archival samples may be utilized provided the specimen it is not older than 5 years of age. When archival samples are used to assess PD-L1 status, the age of the sample / date of collection should be captured.
- Samples submitted for PD-L1 testing should be formalin fixed and embedded in paraffin. Samples from fine needle aspirates (FNA) or decalcified bone are not appropriate for PD-L1 analysis.

Sample data collection for PD-L1 testing

The following fields of data should be collected from the site/institution collecting and if, indicated shipping

of the samples:

Patient identifier (e-code or unique identifier)

- Specimen identifier (written on the specimen)
- Site identifier
- Specimen collection date
- Type of specimen submitted
- Quantity of specimen
- Date of sectioning
- Archival of fresh tumor
- Tumor type
- Primary tumor location
- Metastatic tumor location (if applicable)
- Fixative

The following fields of data should be collected from PD-L1 testing laboratory:

- Are the negative and positive controls stained correctly
- Is the H&E material acceptable
- Is morphology acceptable
- Total percent positivity of PD-L1 in tumor cells
- PD-L1 status (positive, negative or NA) in tumor cells
- Total percent positivity of PD-L1 in infiltrating immune cells

Previously frozen tissue is not acceptable for processing to FFPE for PD-L1 testing. Sections should be stored at ambient temperature and protected from light until use or shipment to testing lab by courier at ambient temperature. It is recommended that slides are cut freshly prior to PD-L1 testing and they are used within 90 days of being cut to obtain PD-L1 status

8.3.2 Estimate of volume of blood to be collected

The total of two blood samples will be drawn from each subject in this study (at baseline, C2D1, end of treatment/progression) will be managed according the laboratory manual and stored -80ºC (-20ºC if not available) in the investigators centers. At the end of the study treatment period and follow-up up, and regarding results obtained, the sponsor and investigators will decide if biomarker analyses will be performed.

8.3.3 Archival tumor samples

8.3.3.1 Archival tumor samples

Archival tumor samples in formalin-fixed paraffin-embedded tissue block will be identified at screening period. Samples will only be used at the end of the study treatment and always regarding results obtained with

the investigational drugs.

8.3.4 Withdrawal of informed consent for donated biological samples

If a subject withdraws consent to the use of donated samples, the samples will be disposed of/destroyed, and the action documented.

Participation in biological samples analyses are optional, and patients can participate in the study without giving the consent to use their biological samples.

The Principal Investigator:

- Ensures that biological samples from that subject, if stored at the study site, are immediately identified, disposed of /destroyed, and the action documented
- Ensures the laboratory(ies) holding the samples is/are informed about the withdrawn consent immediately and that samples are disposed/destroyed, the action documented and the signed document returned to the study site
- Ensures that the subject is informed about the sample disposal.

9. DISEASE EVALUATION AND METHODS

Primary Objective

Disease evaluation according RECIST 1.1.

A copy of the RECIST 1.1 guideline will be included in the ISF.

RECIST 1.1 will be modified so that PD must be confirmed at the next scheduled visit, preferably, and no earlier than 4 weeks after the initial assessment of PD in the absence of clinically significant deterioration. Treatment with durvalumab + tremelimumab would continue between the initial assessment of progression and confirmation for progression.

Secondary objectives (irRECIST)

The response to immunotherapy may differ from the typical responses observed with cytotoxic chemotherapy including the following (Wolchok et al 2009, Nishino et al 2013):

- Response to immunotherapy may be delayed
- Response to immunotherapy may occur after PD by conventional criteria
- The appearance of new lesions may not represent PD with immunotherapy
- SD while on immunotherapy may be durable and represent clinical benefit.

Based on the above-described unique response to immunotherapy and based on guidelines from regulatory agencies, e.g., European Medicines Agency's "Guideline on the evaluation of anti-cancer medicinal products in man" (EMA/CHMP/205/95/Rev.4) for immune modulating anti-cancer compounds, the study may wish to implement the following in addition to standard RECIST 1.1 criteria:

- RECIST 1.1 will be modified so that PD must be confirmed at the next scheduled visit, preferably, and no earlier than 4 weeks after the initial assessment of PD in the absence of clinically significant deterioration. Treatment with durvalumab + tremelimumab would continue between the initial assessment of progression and confirmation for progression.
- In addition, subjects may continue to receive durvalumab + tremelimumab beyond confirmed PD in the absence of clinically significant deterioration and if investigators consider that subjects continue to receive benefit from treatment.

Progression would be considered confirmed if the following criteria are met:

- $\geq$20% increase in the sum diameters of target lesions compared with the nadir at 2 consecutive visits with an absolute increase of 5 mm, and/or
- Clinically significant progression (worsening) of non-target lesions or new lesions at the confirmatory PD time point compared with the first time point where progression of non-target lesions or new lesions was identified, and/or
- Additional new unequivocal lesions at the confirmatory PD time point compared with the first time point at which new lesions were identified.

In the absence of clinically significant deterioration, the Investigator should continue study treatment until progression is confirmed.

If progression is not confirmed, then the patient should continue on study treatment and on treatment

assessments.

Modification of RECIST as described may discourage the early discontinuation of durvalumab + tremelimumab and provide a more complete evaluation of its anti-tumor activity than would be seen with conventional response criteria. Nonetheless, the efficacy analysis will be conducted by programmatically deriving each efficacy endpoint based on RECIST 1.1 criteria.

Of note, clinically significant deterioration is considered to be a rapid tumor progression that necessitates treatment with anti-cancer therapy other than durvalumab + tremelimumab or with symptomatic progression that requires urgent medical intervention (e.g., central nervous system metastasis, respiratory failure due to tumor compression, spinal cord compression).

9.1 Efficacy variable

The RECIST 1.1 criteria will be used to assess patient response to treatment by determining progression-free survival rates at 6, 9 and 12 months, median progression-free survival, overall response rate and median overall survival using Investigator assessments. The management of patients will be based in part upon the results of the RECIST 1.1 assessment conducted by the Investigator.

10. ASSESSMENT OF SAFETY

The Principal Investigator is responsible for ensuring that all staff involved in the study is familiar with the content of this section.

10.1 Safety Parameters

10.1.1 Definition of adverse events

The International Conference on Harmonization (ICH) Guideline for Good Clinical Practice (GCP) E6(R1) defines an AE as:

Any untoward medical occurrence in a patient or clinical investigation subject administered a pharmaceutical product and which does not necessarily have a causal relationship with this treatment. An AE can therefore be any unfavorable and unintended sign (including an abnormal laboratory finding), symptom, or disease temporally associated with the use of a medicinal product, whether or not considered related to the medicinal product.

An AE includes but is not limited to any clinically significant worsening of a subject's pre-existing condition. An abnormal laboratory finding (including ECG finding) that requires an action or intervention by the investigator, or a finding judged by the investigator to represent a change beyond the range of normal physiologic fluctuation, should be reported as an AE.

Adverse events may be treatment emergent (ie, occurring after initial receipt of investigational product) or non treatment emergent. A non treatment-emergent AE is any new sign or symptom, disease, or other untoward medical event that begins after written informed consent has been obtained but before the subject has received investigational product.

Elective treatment or surgery or pre planned treatment or surgery (that was scheduled prior to the subject being enrolled into the study) for a documented pre-existing condition, that did not worsen from baseline, is not considered an AE (serious or non serious). An untoward medical event occurring during the pre scheduled

elective procedure or routinely scheduled treatment should be recorded as an AE or SAE.

The term AE is used to include both serious and non-serious AEs.

10.1.2 Definition of serious adverse events

A serious adverse event is an AE occurring during any study phase (i.e., screening, run-in, treatment, wash-out, follow-up), at any dose of the study drugs that fulfils one or more of the following criteria:

1- Results in death

2- Is immediately life-threatening

3- Requires inpatient hospitalization or prolongation of existing hospitalization

4- Results in persistent or significant disability or incapacity

5- Is a congenital abnormality or birth defect in offspring of the subject

6- Is an important medical event that may jeopardize the patient or may require medical intervention to prevent one of the outcomes listed above:

7- Medical or scientific judgment should be exercised in deciding whether expedited reporting is appropriate in this situation. Examples of medically important events are intensive treatment in an emergency room or at home for allergic bronchospasm, blood dyscrasias, or convulsions that do not result in hospitalizations; or development of drug dependency or drug abuse.

The causality of SAEs (their relationship to all study treatment/procedures) will be assessed by the investigator(s) and communicated to the sponsor.

10.1.3 Durvalumab + tremelimumab adverse events of special interest

An adverse event of special interest (AESI) is one of scientific and medical interest specific to understanding of the Investigational Product and may require close monitoring and rapid communication by the investigator to the sponsor. An AESI may be serious or non-serious. The rapid reporting of AESIs allows ongoing surveillance of these events in order to characterize and understand them in association with the use of this investigational product. A report is required using the SAE form, indicating an event of special interest.

AESIs for durvalumab and tremelimumab include but are not limited to events with a potential inflammatory or immune-mediated mechanism and which may require more frequent monitoring and/or interventions such as steroids, immunosuppressants and/or hormone replacement therapy. These AESIs are being closely monitored in clinical studies with durvalumab monotherapy and combination therapy. An immune-related adverse event (irAE) is defined as an adverse event that is associated with drug exposure and is consistent with an immune-mediated mechanism of action and where there is no clear alternative aetiology. Serologic, immunologic, and histologic (biopsy) data, as appropriate, should be used to support an irAE diagnosis. Appropriate efforts should be made to rule out neoplastic, infectious, metabolic, toxin, or other etiologic causes of the irAE.

Events with an inflammatory or immune mediated mechanism could occur in nearly all organs. Potential risks with an immune-mediated aetiology that are rare or less frequent include, but are not limited to, Guillain-Barre Syndrome, myasthenia gravis, pericarditis, sarcoidosis, uveitis, and other events involving the eye (eg, keratitis and optic neuritis), skin (eg, scleroderma, vitiligo and pemphigoid), haematological (eg, haemolytic anaemia and immune thrombocytopenic purpura), rheumatological events (polymyalgia rheumatic and autoimmune arthritis), vasculitis, non-infectious meningitis and non-infectious encephalitis.

If the Investigator has any questions in regards to an adverse event (AE) being an irAE, the Investigator should

promptly contact the Study Physician.

AESIs observed with durvalumab and tremelimumab include:

-
- Diarrhea / Colitis and intestinal perforation
- Pneumonitis / ILD
- hepatitis / transaminase increases
- Endocrinopathies (i.e. events of hypophysitis/hypopituitarism, thyroiditis, adrenal insufficiency, hyper- and hypothyroidism and type I diabetes mellitus)
- Rash / Dermatitis
- Nephritis / Blood creatinine increases
- Pancreatitis / serum lipase and amylase increases
- Myocarditis
- Myositis / Polymyositis
- Intestinal Perforations
- Other inflammatory responses that are rare/less frequent with a potential immune-mediated etiology include, but are not limited to:
- Pericarditis
- Neuromuscular toxicities (such as Guillain-Barre syndrome and myasthenia gravis)
- Sarcoidosis
- Uveitis
- Other events involving the eye and skin
- Hematological events
- Rheumatological events
- Vasculitis
- Non-infectious meningitis
- Non-infectious encephalitis.

It is possible that events with an inflammatory or immune mediated mechanism could occur in nearly all organs.

In addition, infusion-related reactions and hypersensitivity/anaphylactic reactions with a different underlying pharmacological etiology are also considered AESIs.

Further information on these risks (e.g. presenting symptoms) can be found in the current version of the

durvalumab and tremelimumab Investigator Brochure.

Guidelines for the management of patients with immune-related AEs (irAEs) are provided in Appendix 1. In addition to the dose modification guidelines provided in Appendix 1, it is recommended that irAEs are managed according to the general treatment guidelines outlined for ipilimumab (Weber et al, 2012). These guidelines recommend the following:

- Patients should be evaluated to identify any alternative etiology.
- In the absence of a clear alternative etiology, all events of an inflammatory nature should be considered immune related.
- Symptomatic and topical therapy should be considered for low-grade events.
- Systemic corticosteroids should be considered for a persistent low-grade event or for a severe event.
- More potent immunosuppressives should be considered for events not responding to systemic steroids (eg, infliximab or mycophenolate).

If the Investigator has any questions in regards to an AE being an irAE, the Investigator should immediately contact the Study Physician.

Infusion reactions

AEs of infusion reactions (also termed infusion-related reactions) are of special interest to [redacted] and are defined, for the purpose of this protocol, as all AEs occurring from the start of IP infusion up to 48 hours after the infusion start time. For all infusion reactions, SAEs should be reported to the sponsor as described in Section 10.3.

Hypersensitivity reactions

Hypersensitivity reactions as well as infusion-related reactions have been reported with anti-PD-L1 and anti-PD-1 therapy (Brahmer et al 2012). As with the administration of any foreign protein and/or other biologic agents, reactions following the infusion of mAbs can be caused by various mechanisms, including acute anaphylactic (IgE-mediated) and anaphylactoid reactions against the mAbs and serum sickness. Acute allergic reactions may occur, may be severe, and may result in death. Acute allergic reactions may include hypotension, dyspnea, cyanosis, respiratory failure, urticaria, pruritus, angioedema, hypotonia, arthralgia, bronchospasm, wheeze, cough, dizziness, fatigue, headache, hypertension, myalgia, vomiting, and unresponsiveness. Guidelines for the management of patients with hypersensitivity (including anaphylactic reaction) and infusion-related reactions are provided in Appendix 1.

Hepatic function abnormalities (hepatotoxicity)

Hepatic function abnormality is defined as any increase in ALT or AST to greater than 3 × ULN and concurrent increase in total bilirubin to be greater than 2 × ULN. Concurrent findings are those that derive from a single blood draw or from separate blood draws taken within 8 days of each other. Follow-up investigations and inquiries will be initiated promptly by the investigational site to determine whether the findings are reproducible and/or whether there is objective evidence that clearly supports causation by a disease (eg, cholelithiasis and bile duct obstruction with distended gallbladder) or an agent other than the IP. Guidelines

for management of patients with hepatic function abnormality are provided in section 10.3.3.2.

For the consultation of the complete list of adverse events see the last version of IB provided in the ISF.

10.2 Assessment of safety parameters

10.2.1 Assessment of severity

Assessment of severity is one of the responsibilities of the investigator in the evaluation of AEs and SAEs. Severity will be graded according to the NCI CTCAE v4.03.

The determination of severity for all other events not listed in the CTCAE should be made by the investigator based upon medical judgment and the severity categories of Grade 1 to 5 as defined below:

Grade 1 (mild): An event that is usually transient and may require only minimal treatment or therapeutic intervention. The event does not generally interfere with usual activities of daily living.

Grade 2 (moderate): An event that is usually alleviated with additional specific therapeutic intervention. The event interferes with usual activities of daily living, causing discomfort but poses no significant or permanent risk of harm to the subject.

Grade 3 (severe): An event that requires intensive therapeutic intervention. The event interrupts usual activities of daily living, or significantly affects the clinical status of the subject.

Grade 4 (life threatening): An event, and/or its immediate sequelae, that is associated with an imminent risk of death or with physical or mental disabilities that affect or limit the ability of the subject to perform activities of daily living (eating, ambulation, toileting, etc).

Grade 5 (fatal): Death (loss of life) as a result of an event.

It is important to distinguish between serious criteria and severity of an AE. Severity is a measure of intensity whereas seriousness is defined by the criteria in Section 9.2.1. A Grade 3 AE need not necessarily be considered an SAE. For example, a Grade 3 headache that persists for several hours may not meet the regulatory definition of an SAE and would be considered a non serious event, whereas a Grade 2 seizure resulting in a hospital admission would be considered an SAE.

10.2.2 Assessment of relationship

Relationship of adverse events with study drugs will be assessed by investigators based on toxicity profile described for each study drug (see investigator brochure).

10.3 Recording of adverse events and serious adverse events

Adverse events will be recorded in the eCRF using a recognized medical term or diagnosis that accurately reflects the event. Adverse events will be assessed by the investigator for severity, relationship to the investigational product, possible etiologies, and whether the event meets criteria of an SAE and therefore requires immediate notification and not later than 24 h, to the Sponsor. The following variables will be

collected for each AE:

- AE (verbatim)
- The date when the AE started and stopped
- Changes in NCI CTCAE grade and the maximum CTC grade attained
- Whether the AE is serious or not
- Investigator causality rating against durvalumab or tremelimumab (yes or no)
- Action taken with regard to durvalumab + tremelimumab
- Outcome

In addition, the following variables will be collected for SAEs as applicable:

- Date AE met criteria for serious AE
- Date Investigator became aware of serious AE
- AE is serious due to criteria
- Date of hospitalization
- Date of discharge
- Probable cause of death
- Date of death
- Autopsy performed
- Description of AE
- Causality assessment in relation to Study procedure(s)

Events, which are unequivocally due to disease progression, should not be reported as an AE during the study.

10.3.1 Study recording period and follow-up for adverse events and serious adverse events

Adverse events and serious adverse events will be recorded from time of signature of informed consent, throughout the treatment period and including the follow-up period (90 days after the last dose of durvalumab + tremelimumab).

During the course of the study all AEs and SAEs should be proactively followed up for each subject. Every effort should be made to obtain a resolution for all events, even if the events continue after discontinuation/study completion.

If a subject discontinues from treatment for reasons other than disease progression, and therefore continues to have tumor assessments, drug or procedure-related SAEs must be captured until the patient is considered to have confirmed PD and will have no further tumor assessments.

The investigator is responsible for following all SAEs until resolution, until the subject returns to baseline status, or until the condition has stabilized with the expectation that it will remain chronic, even if this extends

beyond study participation.

Follow-up of unresolved adverse events

Any AEs that are unresolved at the subject's last visit in the study are followed up by the investigator for as long as medically indicated, but without further recording in the eCRF. After 90 days, only subjects with ongoing investigational product-related SAEs will continue to be followed for safety.

The sponsor and [redacted] retain the right to request additional information for any subject with ongoing AE(s)/SAE(s) at the end of the study, if judged necessary.

Post study events

After the subject has been permanently withdrawn from the study, there is no obligation for the investigator to actively report information on new AE or SAEs occurring in former study subjects after the 90-day safety follow-up period for patients treated with durvalumab + tremelimumab. However, if an investigator learns of any SAEs, including death, at any time after the subject has been permanently withdrawn from study, and he/she considers there is a reasonable possibility that the event is related to study treatment,the investigator should notify the study sponsor, who will inform [redacted] Drug Safety.

10.3.2 Reporting of serious adverse events

All SAEs will be reported, whether or not considered causally related to the investigational product, or to the study procedure(s).. Sponsor is responsible for informing the Health Authorities of the SAE as per local requirements.

After the initial notification of an AE or SAE, the investigator is required to follow up each case and obtain more data on the patient's state. All the AEs and SAEs documented in previous visits must be reviewed in subsequent visits. All AEs and SAEs must be monitored until their resolution, stabilization, explanation of the event by another cause, or until the follow-up period ends. This is applicable to all the patients, including those who withdraw early.

The investigator will include in the follow-up any additional research that may clarify the nature and/or causality of the AE or SAE. This might include additional laboratory tests or studies, histopathologic examinations, or the consultation of other healthcare professionals. Any co-investigator can ask the investigator to carry out additional evaluations to definitively clarify the nature and/or the causality of AEs or SAEs. If the patient dies while participating in the study or during a follow-up period agreed upon mutually, the investigator will provide a copy of any postmortem finding requested, including histopathology.

The sponsor is responsible for notifying all serious or unexpected AEs potentially related to the treatments being investigated that occur during the study to the AEMPS, competent bodies of the autonomous communities where the clinical trial is being conducted within the time frame established by Spanish law and will concurrently forward all such reports to [redacted]. A copy of the SAE report must be sent by e-mail to [redacted] at the time the event is reported to the Health Authorities [redacted] It is the responsibility of the sponsor to compile all necessary information and ensure that the Health Authorities receives a report according to the reporting

requirement timelines and to ensure that these reports are also submitted to [redacted] at the same time.

SAE report and accompanying cover page will be sent by way of email to

DEPARTAMENTO DE INVESTIGACIÓN [redacted]
**Fax:** [redacted]
**Email:** [redacted]

If a non-serious AE becomes serious, this and other relevant follow-up information must also be provided to [redacted] and the Health Authorities.

Serious adverse events that do not require expedited reporting to the Health Authorities still need to be reported [redacted] preferably using the MedDRA coding language for serious adverse events. This information should be reported on a monthly basis and under no circumstance less frequently than quarterly.

10.3.2.1 Reporting of deaths

All deaths that occur during the study, or within the protocol-defined 90-day post-last dose of durvalumab + tremelimumab safety follow-up period must be reported as follows:

- Death that is clearly the result of disease progression should be documented but should not be reported as an SAE.
- Where death is not due (or not clearly due) to progression of the disease under study, the AE causing the death must be reported to as a SAE within **24 hours** (see Section 10.3.2 for further details). The report should contain a comment regarding the co-involvement of progression of disease, if appropriate, and should assign main and contributory causes of death.
- Deaths with an unknown cause should always be reported as a SAE.

Deaths that occur following the protocol-defined 90-day post-last-dose of durvalumab safety follow-up period will be documented as events for survival analysis, but will not be reported as an SAE.

10.3.3 Other events requiring reporting

10.3.3.1 Overdose

An overdose is defined as a subject receiving a dose of durvalumab + tremelimumab in excess of that specified in the Investigator's Brochure, unless otherwise specified in this protocol.
Any overdose of a study subject with durvalumab + tremelimumab, with or without associated AEs/SAEs, is required to be reported within 24 hours of knowledge of the event to the sponsor ,who will send a copy to [redacted] Patient Safety, or designed using the designated Safety e-mailbox (see Section 10.3.2 for contact information). If the overdose results in an AE, the AE must also be recorded as an AE (see Section 10.3). Overdose does not automatically make an AE serious, but if the consequences of the overdose are serious, for example death or hospitalization, the event is serious and must be recorded and reported as an SAE (see Section 10.3 and Section 10.3.2). There is currently no specific treatment in the event of an overdose of durvalumab or tremelimumab.

The investigator will use clinical judgment to treat any overdose.

10.3.3.2 Hepatic function abnormality

Hepatic function abnormality (as defined in Section 10.1.3.) in a study subject, with or without associated clinical manifestations, is required to be reported as "hepatic function abnormal" ***within 24 hours of***

knowledge of the event to the sponsor ,who will send a copy to [redacted] Patient Safety, using the designated Safety e-mailbox (see Section 10.3.2 for contact information), unless a definitive underlying diagnosis for the abnormality (e.g., cholelithiasis or bile duct obstruction) that is unrelated to investigational product has been confirmed.

- If the definitive underlying diagnosis for the abnormality has been established and is unrelated to investigational product, the decision to continue dosing of the study subject will be based on the clinical judgment of the investigator.
- If no definitive underlying diagnosis for the abnormality is established, dosing of the study subject must be interrupted immediately. Follow-up investigations and inquiries must be initiated by the investigational site without delay.

Grade 1-2 events should be considered AEs, not SAEs. However, Grade 1-2 events should be reported as SAEs if requires hospitalization or if they are medically important events based on the investigator judgement.

Grade 3 events, should be reported as SAE if requires hospitalization or if they are medically important events based on the investigator judgement.

All grade 4 events should be reported as SAEs.

Cases where a patient shows elevations in liver biochemistry may require further evaluation and occurrences of AST or ALT ≥3 × ULN together with total bilirubin ≥2 × ULN may need to be reported as SAEs.
Each reported event of hepatic function abnormality will be followed by the investigator and evaluated by the sponsor.

10.3.3.3 Pregnancy

a) Maternal exposure

If a patient becomes pregnant during the course of the study, the IPs should be discontinued immediately.
Pregnancy itself is not regarded as an AE unless there is a suspicion that the IP under study may have interfered with the effectiveness of a contraceptive medication. Congenital abnormalities or birth defects and spontaneous miscarriages should be reported and handled as SAEs. Elective abortions without complications should not be handled as Aes. The outcome of all pregnancies (spontaneous miscarriage, elective termination, ectopic pregnancy, normal birth, or congenital abnormality) should be followed up and documented even if the patient was discontinued from the study.
If any pregnancy occurs in the course of the study, then the Investigator or other site personnel should inform the appropriate sponsor representatives within 1 day, ie, immediately, but **no later than 24 hours** of when he or she becomes aware of it.
The designated sponsor representative will work with the Investigator to ensure that all relevant information is provided to the sponsor, who will inform [redacted] Patient Safety data entry site.
The same timelines apply when outcome information is available.

b) Paternal exposure

Male patients should refrain from fathering a child or donating sperm during the study and for 180 days after the last dose of durvalumab + tremelimumab combination therapy or 90 days after the last dose of durvalumab monotherapy, whichever is the longer time period.
Pregnancy of the patient's partner is not considered to be an AE. However, the outcome of all pregnancies (spontaneous miscarriage, elective termination, ectopic pregnancy, normal birth, or congenital abnormality) occurring from the date of the first dose until 90 days after the last dose should, if possible, be followed up and documented.

Where a report of pregnancy is received, prior to obtaining information about the pregnancy, the Investigator must obtain the consent of the patient's partner. Therefore, the local study team should adopt the generic ICF template in line with local procedures and submit it to the relevant Ethics Committees (ECs) prior to use.

11. STATISTICAL METHODS AND SAMPLE SIZE DETERMINATION

11.1. Description of analysis sets

The statistical analyses described in this section will be performed as further outlined in the Statistical Analysis Plan, which will be included in the clinical study report for this protocol.

Analysis Sets

The analysis sets will be defined as follows:

- **Full Analysis Set** will include all allocated subjects. This will be primary analysis set for the efficacy endpoints.
- **Per Protocol Analysis Set** will include those subjects who were allocated and received at least one dose of the assigned study drug and had no major protocol deviations. The subjects will complete both baseline and at least one post-baseline tumor assessments (week 12).
- **Safety Analysis Set** will include all subjects who were allocated and received at least one dose of the study drug and had at least one post-baseline safety evaluation (week 12). This will be the analysis set for all safety evaluations.
- **Pharmacodynamic Analysis Set**: All the subjects who have received at least one dose of study drug and have evaluable pharmacodynamic data.

Demographic and other baseline characteristics

Demographic and other baseline characteristics will be summarized and listed. For continuous demographic/baseline variables including age, weight, and vital signs, results will be summarized and presented as n, number of not available data (NA), mean, standard deviation, median, and minimum and maximum values. For categorical variables such as race/ethnicity, the number and percentage of subjects will be used.

Prior and concomitant medications

Concomitant medications will be assigned an 11-digit code using the World Health Organization Drug Dictionary (WHO DD) drug codes. Concomitant medications will be further coded to the appropriate Anatomical-Therapeutic-Chemical (ATC) code indicating therapeutic classification. Prior and concomitant medications will be summarized and listed by drug and drug class.

Efficacy Analyses

All efficacy analyses will be based primarily on the Full Analysis Set and secondarily on the Per Protocol Analysis Set.

Data cut-off for the primary study analysis will happen following after the last patient included in the study has performed the first tumor evaluation (week 12 after first dose of study drug).The following tumor evaluations will take place every 12 weeks until documentation of disease progression or start of another

anticancer therapy.

Analysis of primary efficacy variable

The analysis of clinical benefit rate and overall survival rate will be performed independently for each study cohort when the last patient included in the corresponding cohort of the study will arrived to 9 months after inclusion in the study or have progressed. The primary objective of the study will be based on the investigator assessment.

11.2. Methods of statistical analyses

Strategic decisions about future development of immune therapy in neuroendocrine tumors will be available after the results of this trial.

Summary tables (descriptive statistics and frequency tables) will be provided for all baseline variables, efficacy variables, and safety variables, as appropriate. Continuous variables will be summarized with descriptive statistics (mean, standard deviation, range, and median). Ninety-five (95) percent confidence intervals (95% CI) may also be presented, as appropriate. Frequency counts and percentage of subjects within each category will be provided for categorical data.

The primary efficacy analysis will be performed using the binomial test procedure. Missing data will be treated using statistical multiple random imputation.

Secondary endpoints will be summarized with descriptive statistics. Continuous variables will be summarized with n, mean, standard deviation, and range, frequency counts and percentage of subjects within each category will be provided for categorical data. Multivariate regression models will be used to study relations between explanatory variables and primary endpoint. Survival analysis will be performed to analyze PFS, Kaplan-Meier curves will be presented and possible comparisons will be tested using the log-rank test or the Cox proportional hazard model for multivariate analysis, hazard ratios (HR) and their 95% confidence interval (CI95%) will be provided. Patients with lost of follow-up or treatment discontinuation will be included in the final analysis of primary endpoint if they have at least one tumor evaluation and considered as censored data for survival endpoints.

R software version 3.2.1 will be used for all analysis.

Safety Analyses

Safety analyses will be based on the Safety Analysis Set. All safety analyses will be summarized separately by cohort. Adverse events and serious adverse events, laboratory test results, physical examination findings and vital signs, and their changes from baseline will be summarized using descriptive statistics. Abnormal values will be flagged.

11.3. Determination of sample size

Sample size has been calculated using one-sample Superiority test (function One Sample Proportion. NIS of the Trial Size package of R software). According to previous knowledge, it is estimated that the reference value for the likelihood to be progression-free at 9 months is 30% and we expect an increase of 20% with a superiority margin of 10%. With a unilateral alpha level of 5% and 80% power, we estimate to include 28 patients per group, with an expected lost to follow-up rate of 10%, a final sample size in each 1, 2 and 3 cohort will include 31 patients.

For cohort 4, and according to previous knowledge, it is estimated that the reference proportion of patients being alive at 9 months is 13% and we expect an increase of 10% with a superiority margin of 5%. With a unilateral alpha level of 5% and 80% power, we estimate to include 30 patients per group, with an expected lost to follow-up rate of 10%, a final sample size will include 33 patients. Summarizing, total sample size will

include 126 patients: 31 patients for cohorts 1 to 3, and 33 patients for cohort 4.

12. ETHICAL AND REGULATORY REQUIREMENTS

12.1. Ethical conduct of the study

The study will be performed in accordance with ethical principles that have their origin in the Declaration of Helsinki and are consistent with ICH/Good Clinical Practice, and applicable regulatory requirements including those related to Subject data protection.

12.2. Ethics and regulatory review

The protocol, ICF, and appropriate related documents must be reviewed and approved by an EC constituted and functioning in accordance with ICH E6, Section 3, and any local regulations,. Any protocol amendment and/or revision to the ICF will be resubmitted to the EC for review and approval, except for changes involving only logistical or administrative aspects of the study (e.g., change in CRA[s] or change of telephone number[s]). Documentation of EC compliance with ICH and any local regulations regarding constitution and review conduct will be provided to the sponsor.

A signed letter of study approval from the EC Chairman must be obtained prior to study start and the release of any study drug to the site by the sponsor or its designee. If the EC decides to suspend or terminate the study, the sponsor will immediately send the notice of study suspension or termination by the EC to the investigators. Study progress is to be reported to the EC annually (or as required) by the sponsor. If the investigator is required to report to the EC, he/she will forward a copy to the sponsor at the time of each periodic report. The sponsor will submit periodic reports and inform the EC of any reportable adverse events according to local legislation. Upon completion of the study, the investigator will provide the EC with a brief report of the outcome of the study, if required.

12.3. Informed consent

As part of administering the informed consent document, the investigator must explain to each subject (or guardian/legally authorized representative) the nature of the study, its purpose, the procedures involved, the expected duration, the potential risks and benefits involved, and any potential discomfort. Each subject must be informed that participation in the study is voluntary and that he/she may withdraw from the study at any time and that withdrawal of consent will not affect his/her subsequent medical treatment or relationship with the treating physician. This informed consent should be given by means of a standard written statement, written in non-technical language. The subject should understand the statement before signing and dating it and will be given a copy of the signed document. If a subject is unable to read or if a legally acceptable representative is unable to read, an impartial witness should be present during the entire informed consent discussion. After the written informed consent form and any other written information to be provided to subjects is read and explained to the subject or the subject's legally acceptable representative, and after the subject or the subject's legally acceptable representative has orally consented to the subject's participation in the trial and, if capable of doing so, has signed and personally dated the ICF, the witness should sign and personally date the consent form. The subject will be asked to sign an informed consent at the Screening Visit prior to any study-specific procedures being performed. No subject can enter the study before his/her informed consent has been obtained. The form must be signed and dated by the appropriate parties. The original, signed ICF for each subject will be verified by the sponsor and kept on file, according to local procedure, at the study center.

The subject or the subject's legally authorized representative should be informed in a timely manner if new information becomes available that may be relevant to the subject's willingness to continue participation in

the trial. The communication of this information should be documented.

12.4. Changes to the protocol and informed consent form

There are to be no changes to the protocol without written approval from the sponsor. Protocols will be followed as written. Any change to the protocol requires a written protocol amendment or administrative change that must be approved by the sponsor before implementation. Amendments affecting the safety of subjects, the scope of the investigation, or the scientific quality of the study require submission to Health Authorities as well as additional approval by the EC. These requirements should in no way prevent any immediate action from being taken by the investigator, or by the sponsor, in the interest of preserving the safety of all subjects included in the study. If an immediate change to the protocol is felt by the investigator to be necessary for safety reasons, the sponsor's appropriate study team member must be notified promptly and the EC for the site must be informed immediately. A protocol change intended to eliminate an immediate hazard may be implemented immediately, provided that the Health Authorities and EC are subsequently notified by protocol amendment.

Changes affecting only administrative aspects of the study do not require formal protocol amendments or EC approval, but the EC (if regionally required, the heads of the medical institutions) must be kept informed of such changes. In these cases, the sponsor will send a letter to the EC (if regionally required, the heads of the medical institutions) detailing such changes.

12.5. Audits and inspections

In addition to routine monitoring procedures, the sponsor's Clinical Quality Assurance department conducts audits of clinical research activities in accordance with the sponsor's Standard Operating Procedures (SOPs) to evaluate compliance with the principles of ICH GCP and all applicable local regulations. A government regulatory authority may also wish to conduct an inspection (during the study or after its completion). If an inspection is requested by a regulatory authority, the investigator must inform the sponsor immediately that this request has been made.

12.6. Confidentiality

The contents of this protocol and any amendments and results obtained during the course of this study will be kept confidential by the investigator, the investigator's staff, and the EC and will not be disclosed in whole or in part to others or used for any purpose other than reviewing or performing the study without the written consent of the sponsor. No data collected as part of this study will be utilized in any written work, including publications, without the written consent of the sponsor. These obligations of confidentiality and non-use shall in no way diminish such obligations as set forth in the Confidentiality Agreement between the sponsor and the investigator.

All persons assisting in the performance of this study must be bound by the obligations of confidentiality and non-use set forth in the Confidentiality Agreement between the investigator and sponsor (provided by the sponsor).

All laboratory specimens, evaluation forms, reports, and other records will be identified in a manner designed to maintain subject confidentiality. Each patient will be given a Unique Patient Number (UPN) for this study, provided by the Sponsor. The investigator will custody a log matching the UPN with personal data of patients. All data will be recorded i the appropriate CRFs using this identification number. This number will be provided to the central laboratory to ensure traceability of study samples. Each Investigator will ensure that all site personnel involved will respect the confidentiality of any information about trial subjects. Management of personal data from subjects participating in the trial, particularly as regards consent, will comply with European Directive on Data Privacy (2016/679) and the Spanish Organic Law 3/2018 of 5th December on the protection

of personal data.

At each site, all records will be kept in a secure storage area with limited access. Clinical information will not be released without the written permission of the subject. Subject identity is confidential and may only be known by the Investigator, trial personnel, appointed auditors and monitors, and Health Authorities.

Each Investigator and all employees and coworkers involved with this trial may not disclose or use for any purpose other than performance of the trial, any data, record, or other unpublished, confidential information disclosed to those individuals for the purpose of the trial. Prior written agreement from the sponsor or its designee must be collected for the disclosure of any said confidential information to other parties.

12.7. Insurance

The sponsor has contracted an insurance policy to cover the responsibilities of the investigator and other parties participating in the study, according to the applicable Spanish legislation.

Insurance company: [redacted]

Policy Number: [redacted]

12.8. Publications

The sponsor commits to responsible publication of both the positive and negative results from its clinical trials as required by all governing regulatory and health authorities.

Investigators will not publish the global study results (all sites) unless the sponsor has not done so in a suitable time period after the clinical study report (CSR) has been available. Should the Investigator(s) independently seek to publish results of this study which occur at their study site(s), they must inform the study sponsor of any/all drafts (including, but not limited to papers, manuscripts or abstracts) at least 60 days before submission to the congress, meeting or journal. The sponsor and Investigator(s) will agree with all aspects related to any proposed publications with regards to the following: 1) any proposed publications will be drafted in agreement with international recommendations, such as those from the International Committee of Medical Journal Editors (ICMJE) and all elements of the Consort Statement (2010), to maintain integrity of the trial results in all communications; 2) any proposed publications will state the Clinical Research Ethics Committees which approved the trial and the funding sources of the trial; 3) any proposed publications will occur before disclosure of results to lay people; 4) any proposed publications will not report premature or partial data prior to completion of the analysis of the overall results of the trial.

13. STUDY MANAGEMENT

13.1. Training of study site personnel

The principal investigator will maintain a record of all center staff involved in the clinical trial (doctors, nurses and other staff involved) ensuring that they receive appropriate training to perform the study, and that any new information of relevance to the study will be transmitted to them.

Researchers will be instructed about the procedures of the trial in the investigator meeting and/or initiation visits made by monitors at each participating center prior to the study start.

13.2. Monitoring of the study

The sponsor's or representative (e.g., CRO's CRA) will maintain contact with the investigator and designated staff by telephone, and/or letter, and/or email between study visits. Monitoring visits to each investigational site will be conducted by the assigned CRA as described in the monitoring plan. The investigator (if regionally

required, the heads of the medical institutions) will allow the CRA to inspect the clinical, laboratory, and pharmacy facilities to assure compliance with Good Clinical Practices and local regulatory requirements. The eCRFs and subject's corresponding original medical records (source documents) are to be fully available for review by the sponsor's representatives at regular intervals. These reviews verify adherence to the study protocol and data accuracy in accordance with federal regulations. All records at the investigational site, including source documents, are subject to inspection by the regulatory authorities and to review by the Ethical Committee.

13.3. Source data

In accordance with ICH E6, Section 6.10, source documents include but are not limited to the following:

- Clinic, office, hospital charts;
- Copies or transcribed healthcare provider notes which have been certified for accuracy after production;
- Recorded data from automated instruments such as x-rays, and other imaging reports: e.g., sonograms, CT scans, MRIs, nuclear medicine scans, ECGs, rhythm strips, electroencephalograms (EEGs), polysomnography, and pulmonary function tests (regardless of how these images are stored, including microfiche and photographic negatives);
- Records of telephone contacts;
- Drug distribution and accountability logs maintained in pharmacies or by research personnel;
- Laboratory results and other laboratory test outputs: e.g., urine pregnancy test result documentation;
- Correspondence regarding a study subject's treatment between physicians or memoranda sent to the EC;

13.4. Retention of Records

The circumstances of completion or termination of the study notwithstanding, the investigator (if regionally required, the heads of the medical institutions) has the responsibility to retain all study documents, including but not limited to the protocol, the Investigator's Brochure, regulatory agency registration documents, ICFs, and EC correspondence. The investigational site should retain study documents until at least 25 years after the finalization of the study.

It is requested that at the completion of the required retention period or, should the investigator retire or relocate, the investigator contact the sponsor, allowing the sponsor the option of permanently retaining the study records.

13.5. Quality assurance

This study will be organized, performed, and reported in compliance with the protocol, standard operating practices (SOPs), working practice documents, and applicable regulations and guidelines. Site visit audits will be made periodically by the sponsor's or CRO's qualified compliance auditing team, which is an independent

function from the study conduct team.

13.6. Study timetable and end of study

End of study is defined as Last Subject Last Visit.

- Recruitment period will be: 33 months
- Research Agreement executed: Nov 2016
- Projected IRB/IEC approval: Feb 2017
- First Subject In: Apr 2017
- 50% Enrollment: Dec 2017
- Last Subject In (100% enrollment): Dec 2019
- Last Subject Last Visit: Dec 2021
- Clinical Study Report Dec 2022

14. DATA MANAGEMENT

All software applications used in the collection and validation of data must be properly validated following standard computer system validation and must be compliant to all regulatory requirements.

The Data Management Plan (DMP) defines and documents the procedures necessary to ensure data quality. These activities must be followed to ensure data are properly entered, validated, coded, integrated, reconciled and reviewed.

Data required by the protocol are collected on an electronic Case Report Form (eCRF) and entered into a validated data management system which is compliant to all regulatory requirements. As defined by ICH Guidelines, the Case Report Form (CRF) is a printed, optical, or electronic document designed to record all of the protocol required information to be reported to the sponsor on each trial subject'. In this study, CRF should refer to electronic data collection form. Data collected on the CRF must follow the instructions described in the CRF Completion Guidelines. The Investigator has ultimate responsibility for the collection and reporting of all clinical, safety and laboratory data entered on the CRF.

Any corrections to entries made on the CRF must be documented in a valid audit trail where the corrections must be dated, initialed, the reason for change stated, and original data not obscured. Only data required by the protocol for the purposes of the study should be collected.

The primary objective of the study will be clinical benefit rate at 9 months and survival rate at 9 months by investigator. Images of tumor evaluations will be anonymized correctly identified with trial number patient.

14.1. Study governance and oversight

The safety of all [redacted] products is closely monitored on an ongoing basis by [redacted] representatives in consultation with Patient Safety. Issues identified will be addressed; for instance, this could

involve amendments to the study protocol and letters to Investigators.

15. INVESTIGATIONAL PRODUCT AND OTHER TREATMENTS

15.1. Identity of investigational product(s)

Table 7. List of investigational products for this study

Investigational product	Dosage form and strength	Manufacturer
Durvalumab	1500 mg, solution, IV	MedImmune
Tremelimumab	75 mg, solution, IV	MedImmune

16. LIST OF REFERENCES

Andorsky et al 2011

Andorsky DJ, Yamada RE, Said J, Pinkus GS, Betting DJ, and Timmerman JM. Programmed death ligand 1 is expressed by non-hodgkin lymphomas and inhibits the activity of tumor-associated T cells. Clin Cancer Res. 2011. 17(13):4232-4244

Antonia et al 2014b

Antonia S, Ou SI, Khleif SN, Brahmer J, Blake-Haskins A, Robbins PB, et al. Clinical activity and safety of MEDI4736, an anti-programmed cell death ligand-1 (PD-L1) antibody in patients with NSCLC. Poster presented at the European Society for Medical Oncology (ESMO) Meeting; 2014 Sep 26-30; Madrid, Spain.

Antonia et al 2014a

Antonia S, Goldberg S, Balmanoukian A, Narwal R, Robbins P, D'Angelo G, et al. A Phase 1 open-label study to evaluate the safety and tolerability of MEDI4736, an anti-programmed cell death-ligand 1 (PD-L1) antibody, in combination with tremelimumab in patients with advanced non-small cell lung cancer (NSCLC). Poster presented at European Society of Medical Oncology (ESMO) Meeting; 2014 Sep 26-30; Madrid, Spain.

Antonia et al 2015

Antonia et al. Phase ib study of medi4736 in combination with treme in patients with advanced NSCLC. Chicago, Il ASCO May30-June4,ASCO 2015, poster #3014.

Berry et al 1991

Berry G, Kitchin RM, Mock PA. A comparison of 2 simple hazard ratio estimators based on the log rank test. Stat Med 1991;10(5):749-55.

Bograd et al 2011

Bograd AJ, Suzuki K, Vertes E, Colovos C, Morales EA, Sadelain M, et al. Immune responses and immunotherapeutic interventions in malignant pleural mesothelioma. Cancer Immunol Immunother.

2011;60:1509-27.

Bonomi 2010

Bonomi PD. Implications of key trials in advanced non small cell lung cancer. Cancer 2010;116(5):1155-64.

Brahmer et al 2012

Brahmer JR, Tykodi SS, Chow LQM, Hwu WJ, Topalian SL, Hwu P, et al. Safety and activity of anti-PD-L1 antibody in patients with advanced cancer. N Engl J Med 2012;366(26):2455-65.

Brahmer et al 2014

Brahmer JR, Horn L, Gandhi L, Spigel DR, Antonia SJ, Rizvi NA et al. Nivolumab (anti-PD-1, BMS-936558, ONO-4538) in patients (pts) with advanced non-small-cell lung cancer (NSCLC): Survival and clinical activity by subgroup analysis[ASCO abstract 8112]. J Clin Oncol 2014;32(15)(Suppl).

Brusa et al 2013

Brusa D, Serra S, Coscia M, Rossi D, D'Arena G, Laurenti L, et al. The PD-1/PD-L1 axis contributes to T-cell dysfunction in chronic lymphocytic leukemia. Haematologica. 2013 Jun; 98(6):953-963.

Burt et al 2011

Burt BM, Rodig SJ, Tilleman TR, Elbardissi AW, Bueno R, Sugarbaker DJ. Circulating and tumor-infiltrating myeloid cells predict survival in human pleural mesothelioma. Cancer. 2011;117(22):5234-44.

Ciuleanu et al 2009

Ciuleanu T, Brodowicz T, Zielinski C, Kim JH, Krzakowski M, Laack E, et al. Maintenance pemetrexed plus best supportive care versus placebo plus best supportive care for non-small-cell lung cancer: a randomized, double-blind, phase 3 study. Lancet 2009;374(9699):1432-40.

Collett 2003

Collett D. Modelling survival data in medical research: 2nd ed. Chapman and Hall/CRC; 2003.

D'Addario et al 2010

D'Addario G, Fruh M, Reck M, Baumann P, Klepetko W, Felip E, et al. Metastatic non-small-cell lung cancer: ESMO clinical practice guidelines for diagnosis, treatment and follow-up. Ann Oncol 2010;21(Suppl 5):116-9.

Drake et al 2013

Drake CG, McDermott DF, Sznol M, Choueiri TK, Kluger HM, Powderly JD et al. Survival, safety, and response duration results of nivolumab (Anti-PD-1; BMS-936558; ONO-4538) in a phase I trial in patients with previously treated metastatic renal cell carcinoma (mRCC): Long-term patient follow-up[abstract 4514]. J Clin Oncol 2013;31(Suppl).

Dunn et al 2004

Dunn GP, Old LJ, Schreiber RD. The three Es of cancer immunoediting. Annu Rev Immunol 2004;22:329-60.

Ellis et al 2008

Ellis S, Carroll KJ, Pemberton K. Analysis of duration of response in oncology trials. Contemp Clin Trials

2008;29(4):456-65.

Forde et al 2014

Forde PM, Kelly RJ, Brahmer JR. New strategies in lung cancer: translating immunotherapy into clinical practice. Clin Cancer Res 2014;20(5):1067-73.

Gail and Simon 1985

Gail M, Simon R. Tests for qualitative interactions between treatment effects and patient subsets. Biometrics 1985;41(2):361–72.

GLOBOCAN 2012

GLOBOCAN. Lung cancer, estimated incidence, mortality and prevalence worldwide in 2012. International Agency for Research on Cancer, World Health Organization; Lyon, 2012.Available at http://globocan.iarc.fr/Pages/fact_sheets_cancer.aspx. Accessed on 16 April 2015.

Gong et al 2009

Gong AY, Zhou R, Hu G, Li X, Splinter PL, O'Hara SP, et al. MicroRNA-513 regulates B7-H1 translation and is involved in IFN-gamma-induced B7-H1 expression in cholangiocytes. J Immunol 2009;182(3):1325-33.

Hamanshi et al 2007

Hamanishi J, Mandai M, Iwasaki M, Okazaki T, Tanaka Y, Yamaguchi K, et al. Programmed cell death 1 ligand 1 and tumor-infiltrating CD8+ T lymphocytes are prognostic factors of human ovarian cancer. Proc Nat Acad Sci U S A. 2007;104(9):3360-5.

Herbst et al 2013

Herbst RS, Gordon MS, Fine GD, Sosman JA, Soria JC, Hamid O, et al. A study of MPDL3280A, an engineered PD-L1 antibody in patients with locally advanced or metastatic tumours [abstract 3000]. J Clin Oncol 2013;31(Suppl 15).

Hirano et al 2005

Hirano F, Kaneko K, Tamura H, Dong H, Wang S, Ichikawa M, et al. Blockade of B7-H1 and PD-1 by monoclonal antibodies potentiates cancer therapeutic immunity. Cancer Res 2005;65(3):1089-96.

Hodi et al 2010

Hodi FS, O'Day SJ, McDermott DF, Weber RW, Sosman JA, Haanan JB, et al. Improved survival with ipilimumab in patients with metastatic melanoma [published erratum appears in N Engl J Med 2010;363(13):1290]. N Engl J Med 2010;363(8):711-23.

Hodi et al 2014

Hodi FS, Sznol M, Kluger HM, McDermott DF, Carvajal RD, Lawrence DP et al. Long-term survival of ipilimumab-naive patients (pts) with advanced melanoma (MEL) treated with nivolumab (anti-PD-1, BMS-936558, ONO-4538) in a phase I trial [ASCO abstract 9002]. J Clin Oncol 2014; 32(15)(Suppl).

Howlander et al 2014

Howlander N, Noone AM, Krapcho M, Garshell J, Neyman N, Altekruse SF, et al. SEER cancer statistics review, 1975-2011. Bethesda (MD): National Cancer Institute,. Available from:

URL:http://seer.cancer.gov/csr/1975_2011/based on November 2013 SEER data submission (posted to the SEER web site, April 2014).

IASLC Staging Manual in Thoracic Oncology

Goldstraw P, ed. Staging manual in thoracic oncology. 7th ed. IASLC 2010. (Available on request)

Iwai et al 2002

Iwai Y, Ishida M, Tanaka Y, Okazaki T, Honjo T, Minato N. Involvement of PD-L1 on tumour cells in the escape from host immune system and tumour immunotherapy by PD-L1 blockade.Proc Natl Acad Sci USA 2002;99(19):12293-7.

Keir et al 2008

Keir ME, Butte MJ, Freeman GJ, Sharpe AH. PD-1 and its ligands in tolerance and immunity. Annu Rev Immunol. 2008;26:677-704.

Kirkwood et al 2010

Kirkwood JM, Lorigan P, Hersey P, Hauschild A, Robert C, McDermott D, et al. Phase II trial of tremelimumab (CP-675,206) in patients with advanced refractory or relapsed melanoma. Clin Cancer Res 2010;16(3):1042-8.

Kitano et al 2014

Kitano S, Postow MA, ZieglerCG, Kuk D, Panageas KS, Cortez C, et al. Computational algorithm-driven evaluation of monocytic myeloid-derived suppressor cell frequency for prediction of clinical outcomes. Cancer Immunol Res 2014;2(8):812-21.

Klein et al 2007

Klein JP, Logan B, Harhoff M, Andersen PK. Analyzing survival curves at a fixed point in time. Stat Med 2007;26(24):4505-19.

Korn et al 2008

Korn EL, Liu PY, Lee SJ, Chapman JA, Niedzwiecki D, Suman VJ, et al. Meta-analysis of phase II cooperative group trials in metastatic stage IV melanoma to determine progression-free and overall survival benchmarks for future phase II trials. J Clin Oncol 2008;26(4):527-34.

Krambeck et al 2007

Krambeck AE, Dong H, Thompson RH, Kuntz SM, Lohse CM, Leibovich BC, et al. Survivin and B7-H1 are collaborative predictors of survival and represent potential therapeutic targets for patients with renal cell carcinoma. Clin Cancer Res. 2007;13(6):1749- 56.

Kresowik and Griffith 2009

Kresowik TP, Griffith TS. Bacillus Calmette–Guerin immunotherapy for urothelial carcinoma of the bladder.

Immunother. 2009;1(2):281-8.

Lan and DeMets 1983

Lan KKG, DeMets DL. Discrete sequential boundaries for clinical trials.Biometrika 1983;70(3):659-63.

Loos et al 2008

Loos M, Giese NA, Kleeff J, Giese T, Gaida MM, Bergmann F, et al. Clinical significance and regulation of the costimulatory molecule B7-H1 in pancreatic adenocarcinoma. Cancer Lett. 2008;268(1):98-109.

McDermott 2009

McDermott DF. Immunotherapy of metastatic renal cell carcinoma.Cancer. 2009;115(10 suppl):2298–305.

Meyer et al 2014

Meyer C, Cagnon L, Costa-Nunes Cm, Baumgaaertner P, Montandon N, Leyvraz L, et al. Frequencies of circulating MDSC correlate with clinical outcome of melanoma patients treated with ipilimumab. Cancer Immunol Immunother 2014;63(3):247-57.

Mu et al 2011

Mu CY, Huang JA, Chen Y, Chen C, Zhang XG. High expression of PD-L1 in lung cancer may contribute to poor prognosis and tumor cells immune escape through suppressing tumor infiltrating dendritic cells maturation. Med Oncol. 2011 Sep;28(3):682-8.

Nakano et al 2001

Nakano O, Sato M, Naito Y, Suzuki K, Orikasa S, Aizawa M, et al. Proliferative activity of intratumoral CD8+ T-lymphocytes as a prognostic factor in human renal cell carcinoma: clinicopathologic demonstration of antitumor immunity. Cancer Res. 2001;61:5132-6.

Narwal et al 2013

Narwal R, Roskos LK, Robbie GJ (2013) Population Pharmacokinetics of Sifalimumab, an Investigational Anti-Interferon alpha Monoclonal Antibody, in Systemic Lupus Erythematosus. Clin Pharmacokinet 52:1017–1027

Ng et al 2006

Ng CM, Lum BL, Gimenez V, et al. Rationale for fixed dosing of pertuzumab in cancer patients based on population pharmacokinetic analysis. Pharm Res. 2006;23(6):1275–84.

NCCN 2014

National Comprehensive Cancer Network Clinical Practice Guidelines in Oncology (NCCN Guidelines®).Non-Small Cell Lung Cancer. Version 4.2014. www.nccn.org.

Nishino et al 2013

Nishino M, Biobbie-Hurder A, Gargano M, Suda M, Ramaiya NH, Hodi FS. Developing a common language for tumor response to immunotherapy: immune-related response criteria using unidimensional measurements. Clin Cancer Res 2013;19(14):3936-43.

Nomi et al, 2007

Nomi T, Sho M, Akahori T, Hamada K, Kubo A, Kanehiro H, et al. Clinical significance and therapeutic

potential of the programmed death-1 ligand/programmed death-1 pathway in human pancreatic adenocarcinoma. Cllin. Cancer Res.2007 Apri 1;13(7):2151-7.

Okudaira et al 2009

Okudaira K, Hokari R, Tsuzuki Y, Okada Y, Komoto S, Watanabe C, et al. Blockade of B7-H1 or B7-DC induces an anti-tumour effect in a mouse pancreatic cancer model. Int J Oncol 2009;35(4):741-9.

Pagès et al, 2010

Pagès F, Galon J, Dieu-Nosjean M.C., Tartour E., Sautès-Fridman C., Fridman W.H.2010. Immune infiltration in human tumors: a prognostic factor that should not be ignored. Oncogene. 29:1093-1102 10.1038/onc.2009.416

Pardoll 2012

Pardoll DM. The blockade of immune checkpoints in cancer immunotherapy.Nat Rev Cancer 2012;12(4):252-64.

Park et al, 2010

Park J, Omiya R, Matsumura Y, Sakoda Y, Kuramasu A, Augustine MM, et al. B7-H1/CD80 interaction is required for the induction and maintenance of peripheral T/cell tolerance. Blood.

Paz-Ares et al 2013

Paz-Ares LG, de Marinis F, Dediu M, Thomas M, Pujol JL, Bidoli P, et al. PARAMOUNT: Final overall survival results of the phase III study of maintenance pemetrexed versus placebo immediately after induction treatment with pemetrexed plus cisplatin for advanced nonsquamous non-small-cell lung cancer. J Clin Oncol 2013;31(23):2895-902.

Peggs et al 2009

Peggs KS, Quezada SA, Allison JP. Cancer immunotherapy: co-stimulatory agonists and coinhibitory antagonists. Clin Exp Immunol 2009;157:9-19.

Pisters and LeChevalier 2005

Pisters KM, LeChevalier T. Adjuvant chemotherapy in completely resected non-small-cell lung cancer. J Clin Oncol 2005;23(14):3270-8.

Raymond et al 2011

Raymond E, Dahan L, Raoul JL, Bang YJ, Borbath I, Lombard-Bohas C et al. Sunitinib malate for the treatment of pancreatic neuroendocrine tumors. N Engl J Med. 2011 Feb 10, 364(6): 501-13. doi:10.1056/NEJMoa1003825.

Reck et al 2014

Reck M, Popat S, Reinmuth N, De Ruysscher D, Kerr KM, Peters S; ESMO Guidelines Working Group. Metastatic non-small-cell lung cancer (NSCLC): ESMO clinical practice guidelines for diagnosis, treatment and follow-up. Ann Oncol 2014;25(Suppl 3):iii27-39.

Ribas et al 2013

Ribas A, Kefford R, Marshall MA, Punt CJA, Haanen JB, Marmol M, et al. Phase III randomized clinical trial comparing tremelimumab with standard-of-care chemotherapy in patients with advanced melanoma. J Clin

Oncol 2013;31:616-22.

Robert et al, 2011

Robert C, Thomas L, Bondarenko I, Steven O'Day S, Weber J, Garbe C. Ipilimumab plus Dacarbazine for Previously Untreated Metastatic Melanoma. N Engl J Med 2011; 364:2517-2526.

Sandler et al 2006

Sandler A, Gray R, Perry MC, Brahmer J, Schiller JH, Dowlati A, et al. Paclitaxel-carboplatin alone or with bevacizumab for non-small-cell lung cancer. N Engl J Med 2006;355(24):2524-50.

Scagliotti et al 2008

Scagliotti GV, Parikh P, von Pawel J, Biesma B, Vansteenkiste J, Manegold C, et al. Phase III study comparing cisplatin plus gemcitabine with cisplatin plus pemetrexed in chemotherapy-naive patients with advanced-stage non-small-cell lung cancer. J Clin Oncol 2008;26(21)3543-51.

Schadendorf et al 2013

Schadendorf D, Hodi FS, Robert C et al. Pooled analysis of long-term survival data from phase II and phase III trials of ipilimumab in metastatic or locally advanced, unresectable melanoma. Presented at: European Cancer Congress 2013 (ECCO-ESMO-ESTRO); September 27-October 1, 2013; Amsterdam, The Netherlands. Abstract 24.

Schiller et al 2002

Schiller JH, Harringdon D, Belani CP, Langer C, Sandler A, Krook J, et al. Comparison of four chemotherapy regimens for advanced non-small-cell lung cancer. N Engl J Med 2002;10(346):92-8.

Schwarzenbach et al 2014

Schwarzenbach H, Nishida N, Calin GA, Pantel K. Clinical evidence of circulating cell-free microRNAs in cancer. Nat Rev Clin Oncol 2014;11(3):145-56.

Selke and Siegmund 1983

Selke T, Siegmund D. Sequential analysis of the proportional hazards model. Biometrika 1983;70:315-26.

Sun and Chen 2010

Sun X, Chen C. Comparison of Finkelstein's Method with the conventional approach for interval-censored data analysis. Stat Biopharm Res 2010;2(1):97-108.

Suzuki et al, 2011

Suzuki K, Kachala SS, Kadota K, Shen R, Mo Q, Beer DG, et al. Prognostic immune markers in non-small cell lung cancer. Clin Cancer res. 2011;17(16):5247-56. doi:10.1158/1078-0432.CCR-10-2805.

Tarhini and Kirkwood 2008

Tarhini AA, Kirkwood JM. Tremelimumab (CP-675,206): a fully human anticytotoxic T lymphocyte-associated antigen 4 monoclonal antibody for treatment of patients with advanced cancers. Expert Opin Biol Ther 2008;8(10):1583-93.

Topalian et al 2012

Topalian SL, Hodi FS, Brahmer JR, Gettinger SN, Smith DC, McDermott DF, et al. Safety, activity, and

immune correlates of anti-PD-1 antibody in cancer. N Engl J Med 2012;366(26):2443-54.

Topalian et al 2014

Topalian SL, Sznol M, McDermott DF, Kluger HM, Carvajal RD, Sharfman WH et al. Survival, durable tumor remission, and long-term safety in patients with advanced melanoma receiving nivolumab. J Clin Oncol, 2014 Apr 1; 32(10):1020-30.

Wang et al 2009

Wang DD, Zhang S, Zhao H, et al. Fixed dosing versus body size based dosing of monoclonal antibodies in adult clinical trials. J Clin Pharmacol. 2009;49(9):1012–24.

Wang et al 2013

Wang Z, Han J, Cui Y, Fan K, Zhou X. Circulating microRNA-21 as noninvasive predictive biomarker for response in cancer immunotherapy. Med Hypotheses 2013;81(1):41-3.

Wang et al 2014

Wang E, Kang D, et al. Population pharmacokinetic and pharmacodynamics analysis of tremelimumab in patients with metastatic melanoma. J Clin Pharmacol. 2014 Oct; 54(10):1108-16.

Weber et al 2012

Weber J, Kähler KC, Hauschild A. Management of immune-related adverse events and kinetics of response with ipilimumab. J Clin Oncol 2012;30(21):2691-7.

Whitehead and Whitehead 1991

Whitehead A, Whitehead J. A general parametric approach to the meta-analysis of randomized clinical trials. Stat Med 1991;10(11):1665-77.

Wolchok et al 2009

Wolchok JD, Hoos A, O'Day S, Weber JS, Hammid O, Lebbé, et al. Guidelines for the evaluation of immune therapy activity in solid tumors: immune-related response criteria. Clin Cancer Res. 2009;15:7412-20.

Wolchok et al 2013

Wolchok JD, Kluger H, Callahan MK, Postow MA, Rizvi NA, Lesokhin AM, et al. Nivolumab plus ipilimumab in advanced melanoma. N Engl J Med 2013;369(2):122-33.

Yao et al, 2008

Yao JC, Hassan M, Phan A, et al. One hundred years after "carcinoid": epidemiology of and prognostic factors for neuroendocrine tumors in 35.825 cases in the United States. J Clin Oncol. 2008;26:30663-3072.

Yao et al, 2011

Yao J., Shah M., Ito T., Bohas C., Wolin E., Van Cutsem E., et al (2011). Everolimus for advanced pancreatic neuroendocrine tumors. N Engl J Med 364: 514-523.

Yao et al, 2015

Yao JC, Fazio N, Singh S, et al. Everolimus for the treatment of advanced, non-functional neuroendocrine tumours of the lung or gastrointestinal tract (RADIANT-4): a randomised, placebo-controlled, phase 3 study.

Lancet 2015; Advanced online publication 15 December.

Yuan et al 2011

Yuan J, Adamow M, Ginsberg BA, Rasalan TS, Ritter E, Gallardo HF, et al. Integrated NY-ESO-1 antibody and CD8+ T-cell responses correlated with clinical benefit in advanced melanoma patients treated with ipilimumab. Proc Natl Acad Sci USA 2011;108(40):16723-8.

Zhang et al 2008

Zhang C, Wu S, Xue X, Li M, Qin X, Li W, et al. Anti-tumour immunotherapy by blockade of the PD-1/pd-L1 pathway with recombinant human PD-1IgV. Cytotherapy 2008;10(7):711-9. doi: 10.1080.14653240802320237.

Zhang et al 2012

Zhang S, Shi R, Li C, et al. Fixed dosing versus body size-based dosing of therapeutic peptides and proteins in adults. J Clin Pharmacol. 2012;52(1):18–28.

Zou and Chen, 2008

Zou W, chen L. Inhibitory B7-family molecules in the tumour microenvironment. Nat Rev Immunol. 2008;8(6):467-77.

Appendix 1.Dosing Modification and Toxicity Management Guidelines (TMGs) for Durvalumab Monotherapy, Durvalumab in Combination with other Products, or Tremelimumab Monotherapy. 17 November 2020.

Note: Annex is to be used in any clinical trial protocol within which patients are treated with Durvalumab Monotherapy, Durvalumab in Combination with other Products, or Tremelimumab

	–

			–
			–

			–
		•	–
			–
			–
		•	–

			–
			–
			–
		**1.**	–
		**1.**	
			–

			–
			–
			–
			–
			–
			-

		–
		–
		–

General Considerations Regarding Immune-Mediated Reactions

General Considerations Regarding Immune-Mediated Reactions

These guidelines are provided as a recommendation to support investigators in the management of potential immune-mediated adverse events (imAEs).

Immune-mediated events can occur in nearly any organ or tissue, therefore, these guidelines may not include all the possible immune-mediated reactions. Investigators are advised to take into consideration the appropriate practice guidelines and other society guidelines (e.g., NCCN, ESMO) in the management of these events. Refer to the section of the table titled "Other -Immune-Mediated Reactions" for general guidance on imAEs not noted in the "Specific Immune-Mediated Reactions" section.

Early identification and management of immune-mediated adverse events (imAEs) are essential to ensure safe use of the study drug. Monitor patients closely for symptoms and signs that may be clinical manifestations of underlying immune-mediated adverse events. Patients with suspected imAEs should be thoroughly evaluated to rule out any alternative etiologies (e.g., disease progression, concomitant medications, infections). In the absence of a clear alternative etiology, all such events should be managed as if they were immune-mediated. Institute medical management promptly, including specialty consultation as appropriate. In general, withhold study drug/study regimen for severe (Grade 3) imAEs. Permanently discontinue study drug/study regimen for life-threatening (Grade 4) imAEs, recurrent severe (Grade 3) imAEs that require systemic immunosuppressive treatment, or an inability to reduce corticosteroid dose to 10 mg or less of prednisone or equivalent per day within 12 weeks of initiating corticosteroids.

Based on the severity of the imAE, durvalumab should be withheld and corticosteroids administered. Upon improvement to Grade ≤ 1, corticosteroid should be tapered over ≥ 28 days. More potent immunosuppressive agents such as TNF inhibitors (e.g., infliximab) should be considered for events not responding to systemic steroids. Alternative immunosuppressive agents not listed in this guideline may be considered at the discretion of the investigator based on clinical practice and relevant guidelines. With long-term steroid and other immunosuppressive use, consider need for Pneumocystis jirovecii pneumonia (PJP, formerly known as Pneumocystis carinii pneumonia) prophylaxis, gastrointestinal protection, and glucose monitoring.

Dose modifications of study drug/study regimen should be based on severity of treatment-emergent toxicities graded per NCI CTCAE version in the applicable study protocol.

AE Adverse event; CTC Common Toxicity Criteria; CTCAE Common Terminology Criteria for Adverse Events; imAE immune-mediated adverse event; NCI National Cancer Institute; NCCN National Comprehensive Cancer Network; ESMO European Society for Medical Oncology

Pediatric Considerations Regarding Immune-Mediated Reactions	
**Dose Modifications**	**Toxicity Management**
**The criteria for permanent discontinuation of study drug/study regimen based on CTC grade/severity is the same for pediatric patients as it is for adult patients, as well as to permanently discontinue study drug/study regimen if unable to reduce corticosteroid ≤ a dose equivalent to that required for corticosteroid replacement therapy within 12 weeks of initiating corticosteroids.**	– **All recommendations for specialist consultation should occur with a pediatric specialist in the specialty recommended.**<br>– **The recommendations for steroid dosing (i.e., mg/kg/day) provided for adult patients should also be used for pediatric patients.**<br>– **The recommendations for IVIG and plasmapheresis use provided for adult patients may be considered for pediatric patients.**<br>– **The infliximab 5 mg/kg IV one time dose recommended for adults is the same as recommended for pediatric patients ≥ 6 years old. For subsequent dosing and dosing in children < 6 years old, consult a pediatric specialist.**<br>– **For pediatric dosing of mycophenolate mofetil, consult a pediatric specialist.**<br>– **With long-term steroid and other immunosuppressive use, consider need for PJP prophylaxis, gastrointestinal protection, and glucose monitoring.**

Specific Immune-Mediated Reactions

Adverse Events	Severity Grade of the Event	Dose Modifications	Toxicity Management
**Pneumonitis/Interstitial Lung Disease (ILD)**	**Any Grade**<br>**(Refer to NCI CTCAE applicable version in study protocol for defining the CTC grade/severity)**	**General Guidance**	**For Any Grade**<br>**– Monitor patients for signs and symptoms of pneumonitis or ILD (new onset or worsening shortness of breath or cough). Evaluate patients with imaging and pulmonary function tests, including other diagnostic procedures as described below.**<br>**– Suspected pneumonitis should be confirmed with radiographic imaging and other infectious and disease-related aetiologies excluded, and managed as described below.**<br>**– Initial work-up may include clinical evaluation, monitoring of oxygenation via pulse oximetry (resting and exertion), laboratory work-up, and high- resolution CT scan.**<br>**– Consider Pulmonary and Infectious Diseases consults.**
	**Grade 1**	**No dose modifications required. However, consider holding study drug/study regimen dose as clinically appropriate and during diagnostic work-up for other etiologies.**	**For Grade 1**<br>**– Monitor and closely follow up in 2 to 4 days for clinical symptoms, pulse oximetry (resting and exertion), and laboratory work-up, and then as clinically indicated.**

	**Grade 2**	**Hold study drug/study regimen dose until Grade 2 resolution to Grade ≤1.**<br>● **If toxicity worsens, then treat as Grade 3 or Grade 4.**<br>● **If toxicity improves to Grade ≤1, then the decision to reinitiate study drug/study regimen will be based upon treating physician's clinical judgment and after completion of steroid taper.**	**For Grade 2**<br>– **Monitor symptoms daily and consider hospitalization.**<br>– **Promptly start systemic steroids (e.g., prednisone 1 to 2 mg/kg/day PO or IV equivalent).**<br>– **Reimage as clinically indicated, consider chest CT with contrast and repeat in 3-4 weeks.**<br>– **If no improvement within 2 to 3 days, additional workup should be considered and prompt treatment with IV methylprednisolone 2 to 4 mg/kg/day started.**<br>– **If no improvement within 2 to 3 days despite IV methylprednisolone at 2 to 4 mg/kg/day, promptly start immunosuppressive therapy such as TNF inhibitors (e.g., infliximab at 5 mg/kg IV once, may be repeated at 2 and 6 weeks after initial dose at the discretion of the treating provider). Caution: It is important to rule out sepsis and refer to infliximab label for general guidance before using infliximab. Consider, as necessary, discussing with study physician.**

**Grade 3 or 4**	**Permanently discontinue study drug/study regimen.**	**For Grade 3 or 4**<br>– **Promptly initiate empiric IV methylprednisolone 1 to 4 mg/kg/day or equivalent.**<br>– **Obtain Pulmonary and Infectious Diseases Consults; consider discussing with study physician, as needed.**<br>– **Hospitalize the patient.**<br>– **Supportive care (e.g., oxygen).**<br>– **If no improvement within 2 to 3 days, additional workup should be considered and prompt treatment with additional immunosuppressive therapy such as TNF inhibitors (e.g., infliximab at 5 mg/kg IV, may be repeated at 2 and 6 weeks after initial dose at the discretion of the treating provider). Caution: rule out sepsis and refer to infliximab label for general guidance before using infliximab.**

Diarrhea/Colitis	Any Grade (Refer to NCI CTCAE applicable version in study protocol for defining the CTC grade/severity)	General Guidance	For Any Grade
			– Monitor for symptoms that may be related to diarrhea/enterocolitis (abdominal pain, cramping, or changes in bowel habits such as increased frequency over baseline or blood in stool) or related to bowel perforation (such as sepsis, peritoneal signs, and ileus).<br>– WHEN SYMPTOMS OR EVALUATION INDICATE AN INTESTINAL PERFORATION IS SUSPECTED, CONSULT A SURGEON EXPERIENCED IN ABDOMINAL SURGERY IMMEDIATELY WITHOUT ANY DELAY.<br>– PERMANENTLY DISCONTINUE STUDY DRUG FOR ANY GRADE OF INTESTINAL PERFORATION.<br>– Patients should be thoroughly evaluated to rule out any alternative etiology (e.g., disease progression, other medications, or infections), including testing for Clostridium difficile toxin, etc.<br>– Steroids should be considered in the absence of clear alternative etiology, even for low-grade events, in order to prevent potential progression to higher grade events, including intestinal perforation.<br>– Use analgesics carefully; they can mask symptoms of perforation and peritonitis.

	**Grade 1**	**No dose modifications.**	**For Grade 1**<br>– **Monitor closely for worsening symptoms.**<br>– **Consider symptomatic treatment, including hydration, electrolyte replacement, dietary changes (e.g., American Dietetic Association colitis diet), loperamide, and other supportive care measures.**<br>– **If symptoms persist,consider checking lactoferrin; if positive, treat as Grade 2 below. If negative and no infection, continue Grade 1 management.**

	**Grade 2**	**Hold study drug/study regimen until resolution to Grade ≤1**<br>· **If toxicity worsens, then treat as Grade 3 or Grade 4.**<br>· **If toxicity improves to Grade ≤1, then study drug/study regimen can be resumed after completion of steroid taper.**	**For Grade 2**<br>– **Consider symptomatic treatment, including hydration, electrolyte replacement, dietary changes (e.g., American Dietetic Association colitis diet), and loperamide and/or budesonide.**<br>– **Promptly start prednisone 1 to 2 mg/kg/day PO or IV equivalent.**<br>– **If event is not responsive within 2 to 3 days or worsens despite prednisone at 1 to 2 mg/kg/day PO or IV equivalent, consult a GI specialist for consideration of further workup, such as imaging and/or colonoscopy, to confirm colitis and rule out perforation.**<br>– **If still no improvement within 2 to 3 days despite 1 to 2 mg/kg IV methylprednisolone, promptly start immunosuppressants such as infliximab at 5 mg/kg IV, may be repeated at 2 and 6 weeks after initial dose at the discretion of the treating provider. a Caution: it is important to rule out bowel perforation and refer to infliximab label for general guidance before using infliximab.**<br>– **Consider, as necessary, discussing with study physician if no resolution to Grade ≤1 in 3 to 4 days.**

	Grade 3 or 4	Grade 3<br>· For patient treated with PDL-1 inhibitors, hold study drug/study regimen until resolution to Grade ≤1; study drug/study regimen can be resumed after completion of steroid taper. Permanently discontinue study drug/study regimen for Grade 3 if toxicity does not improve to Grade ≤1 within 14 days.<br>· Permanently discontinue study drug for 1) Grade 3 colitis in patients treated with CTLA-4 inhibitors or 2) Any grade of intestinal perforation in any patient treated with ICI.<br>Grade 4<br>Permanently discontinue study drug/study regimen.	For Grade 3 or 4<br>– Promptly initiate empiric IV methylprednisolone 1 to 2 mg/kg/day or equivalent.<br>– Monitor stool frequency and volume and maintain hydration.<br>– Urgent GI consult and imaging and/or colonoscopy as appropriate.<br>– If still no improvement within 2 days, continue steroids and promptly add further immunosuppressants (e.g., infliximab at 5 mg/kg IV, may be repeated at 2 and 6 weeks after initial dose at the discretion of the treating provider). Caution: Ensure GI consult to rule out bowel perforation and refer to infliximab label for general guidance before using infliximab.<br>– If perforation is suspected, consult a surgeon experienced in abdominal surgery immediately without any delay.
Hepatitis<br>(elevated LFTs)<br><br>PLEASE SEE shaded area immediately below this section to find guidance for management of “Hepatitis (elevated LFTS)” in HCC patients	Any Grade<br>(Refer to NCI CTCAE applicable version in study protocol for defining the CTC grade/severity)	General Guidance	For Any Grade<br>– Monitor and evaluate liver function test: AST, ALT, ALP, and TB.<br>– Evaluate for alternative etiologies (e.g., viral hepatitis, disease progression, concomitant medications).
	Grade 1	· No dose modifications.<br>· If it worsens, then treat as Grade 2.	For Grade 1<br>– Continue LFT monitoring per protocol.

**Infliximab should not be used for management of immune-related hepatitis.**	**Grade 2**	· **Hold study drug/study regimen dose until Grade 2 resolution to Grade ≤1.**<br>· **If toxicity worsens, then treat as Grade 3 or Grade 4.**<br>· **If toxicity improves to Grade ≤1 or baseline, resume study drug/study regimen after completion of steroid taper.**<br>· **Permanently discontinue study drug/study regimen for any case meeting Hy's law criteria (AST and/or ALT >3 × ULN + bilirubin >2 × ULN without initial findings of cholestasis (i.e., elevated alkaline P04) and in the absence of any alternative cause.b**	**For Grade 2**<br>– **Regular and frequent checking of LFTs (e.g., every 1 to 2 days) until LFT elevations improve or resolve.**<br>– **If no resolution to Grade ≤1 in 1 to 2 days, consider discussing with study physician, as needed.**<br>– **If event is persistent (>2 to 3 days) or worsens, promptly start prednisone 1 to 2 mg/kg/day PO or IV equivalent.**

**Grade 3 or 4**	**For Grade 3**<br>**For elevations in transaminases ≤8 × ULN, or elevations in bilirubin ≤5 × ULN:**<br>· **Hold study drug/study regimen dose until resolution to Grade ≤1 or baseline**<br>· **Resume study drug/study regimen if elevations downgrade to Grade ≤1 or baseline within 14 days and after completion of steroid taper.**<br>· **Permanently discontinue study drug/study regimen if the elevations do not downgrade to Grade ≤1 or baseline within 14 days**<br>· **For elevations in transaminases >8 × ULN or elevations in bilirubin >5 × ULN, discontinue study drug/study regimen.**<br><br>**For Grade 4**<br>**Permanently discontinue study drug/study regimen.**	**For Grade 3 or 4**<br>– **Promptly initiate empiric IV methylprednisolone at 1 to 2 mg/kg/day or equivalent.**<br>– **If still no improvement within 2 to 3 days despite 1 to 2 mg/kg/day methylprednisolone IV or equivalent, promptly start treatment with an immunosuppressants (e.g., mycophenolate mofetil 0.5 – 1 g every 12 hours then taper in consultation with hepatology consult). Discuss with study physician if mycophenolate is not available. Infliximab should NOT be used.**<br>– **Perform Hepatology Consult, abdominal workup, and imaging as appropriate.**

**Hepatitis**<br>**(elevated LFTs)**<br><br>**THIS shaded area is guidance only for management of "Hepatitis (elevated LFTs)" in HCC patients**<br><br>**Infliximab should not be used for management of immune-related hepatitis.**<br>**See instructions at bottom of shaded area if transaminase rise is not isolated but (at any time) occurs in setting of either increasing bilirubin or signs of DILI/liver decompensation**	**Any Elevations of AST, ALT, or TB as Described Below**	**General Guidance**	**For Any Elevations Described**<br>– **Monitor and evaluate liver function test: AST, ALT, ALP, and TB.**<br>– **Evaluate for alternative etiologies (e.g., viral hepatitis, disease progression, concomitant medications, worsening of liver cirrhosis [e.g., portal vein thrombosis]).**<br>– **For HBV+ patients: evaluate quantitative HBV viral load, quantitative HBsAg, or HBeAg.**<br>– **For HCV+ patients: evaluate quantitative HCV viral load.**<br>– **Consider consulting Hepatology or Infectious Diseases specialists regarding changing or starting antiviral HBV medications if HBV viral load is >2000 IU/ml.**<br>– **Consider consulting Hepatology or Infectious Diseases specialists regarding changing or starting antiviral HCV medications if HCV viral load has increased by ≥2-fold.**<br>– **For HCV+ with HBcAb+: Evaluate for both HBV and HCV as above.**
	**Isolated AST or ALT >ULN and ≤5.0×ULN, whether normal or elevated at baseline**	· **No dose modifications.**<br>· **If ALT/AST elevations represents significant worsening based on investigator assessment, then treat as described for elevations in the row below.**<br>· **For all transaminase elevations , see instructions at bottom of shaded area if transaminase rise is not isolated but (at any time) occurs in setting of either**	

	**increasing bilirubin or signs of DILI/liver decompensation**	

	Isolated AST or ALT >5.0×ULN and ≤8.0×ULN, if normal at baseline<br><br>Isolated AST or ALT >2.0×baseline and ≤12.5×ULN, if elevated >ULN at baseline	· Hold study drug/study regimen dose until resolution to AST or ALT ≤5.0×ULN .<br>· If toxicity worsens, then treat as described for elevations in the rows below.If toxicity improves to AST or ALT ≤5.0×ULN , resume study drug/study regimen after completion of steroid taper.<br>· Permanently discontinue study drug/study regimen for any case meeting Hy's law criteria, in the absence of any alternative cause.b	– Regular and frequent checking of LFTs (e.g., every 1 to 3 days) until elevations of these are improving or resolved.<br>Recommend consult hepatologist; consider abdominal ultrasound, including Doppler assessment of liver perfusion.<br>– Consider, as necessary, discussing with study physician.<br>– If event is persistent (>2 to 3 days) or worsens, and investigator suspects toxicity to be an imAE, start prednisone 1 to 2 mg/kg/day PO or IV equivalent.<br>– If still no improvement within 2 to 3 days despite 1 to 2 mg/kg/day of prednisone PO or IV equivalent, consider additional workup. If still no improvement within 2 to 3 days despite 2mg/kg/day of IV methylprednisolone, consider additional abdominal workup (including liver biopsy) and imaging (i.e., liver ultrasound), and consider starting immunosuppressants (e.g.,.., mycophenolate mofetil 0.5 – 1 g every 12 hours then taper in consultation with hepatology consult).a Discuss with study physician if mycophenolate mofetil is not available. Infliximab should NOT be used.

	**Isolated AST or ALT >8.0×ULN and ≤20.0×ULN, if normal at baseline**<br><br>**Isolated AST or ALT >12.5×ULN and ≤20.0×ULN, if elevated >ULN at baseline**	· **Hold study drug/study regimen dose until resolution to AST or ALT ≤5.0×ULN Resume study drug/study regimen if elevations downgrade to AST or ALT ≤5.0×ULN within 14 days and after completion of steroid taper.**<br>· **Permanently discontinue study drug/study regimen if the elevations do not downgrade to AST or ALT ≤5.0×ULN within 14 days**	– **Regular and frequent checking of LFTs (e.g., every 1-2 days) until elevations of these are improving or resolved.**<br>– **Consult hepatologist (unless investigator is hepatologist); obtain abdominal ultrasound, including Doppler assessment of liver perfusion; and consider liver biopsy.**<br>– **Consider discussing with study physician, as needed.**<br>– **If investigator suspects toxicity to be immune-mediated, promptly initiate empiric IV methylprednisolone at 1 to 2 mg/kg/day or equivalent.**<br>– **If no improvement within 2 to 3 days despite 1 to 2 mg/kg/day methylprednisolone IV or equivalent, obtain liver biopsy (if it has not been done already) and promptly start treatment with an immunosuppressant (e.g.,., mycophenolate mofetil 0.5 – 1 g every 12 hours then taper in consultation with a hepatologist). Discuss with study physician if mycophenolate is not available. Infliximab should NOT be used.**
	**Isolated AST or ALT >20×ULN, whether normal or elevated at baseline**	**Permanently discontinue study drug/study regimen.**	**Same as above**<br>**(except would recommend obtaining liver biopsy early)**

If transaminase rise is not isolated but (at any time) occurs in setting of either increasing total/direct bilirubin (≥1.5×ULN, if normal at baseline; or 2×baseline, if >ULN at baseline) or signs of DILI/liver decompensation (e.g., fever, elevated INR):

- **Manage dosing for each level of transaminase rise as instructed for the next highest level of transaminase rise**
- **For example, manage dosing for second level of transaminase rise (i.e., AST or ALT >5.0×ULN and ≤8.0×ULN, if normal at baseline, or AST or ALT >2.0×baseline and ≤12.5×ULN, if elevated >ULN at baseline) as instructed for the third level of transaminase rise (i.e., AST or ALT >8.0×ULN and ≤20.0×ULN, if normal at baseline, or AST or ALT >12.5×ULN and ≤20.0×ULN, if elevated >ULN at baseline)**
- **For the third and fourth levels of transaminase rises, permanently discontinue study drug/study regimen**

**Nephritis or renal dysfunction**<br>**(elevated serum creatinine)**	**Any Grade**<br>**(Refer to NCI CTCAE applicable version in study protocol for defining the CTC grade/severity)**	**General Guidance**	**For Any Grade**<br>– **Consult a nephrologist.**<br>– **Monitor for signs and symptoms that may be related to changes in renal function (e.g., routine urinalysis, elevated serum BUN and creatinine, decreased creatinine clearance, electrolyte imbalance, decreased urine output, or proteinuria).**<br>– **Patients should be thoroughly evaluated to rule out any alternative etiology (e.g., disease progression, infections, recent IV contrast, medications, fluid status).**<br>– **Consider using steroids in the absence of a clear alternative etiology even for low-grade events (Grade 2), in order to prevent potential progression to higher grade events.**

**Grade 1**	**No dose modifications.**	**For Grade 1**<br>– **Monitor serum creatinine weekly and any accompanying symptoms.**<br>· **If creatinine returns to baseline, resume its regular monitoring per study protocol.**<br>· **If creatinine worsens, depending on the severity, treat as Grade 2, 3, or 4.**<br>– **Consider symptomatic treatment, including hydration, electrolyte replacement, and diuretics.**
**Grade 2**	**Hold study drug/study regimen until resolution to Grade ≤1 or baseline.**<br>· **If toxicity worsens, then treat as Grade 3 or 4.**<br>· **If toxicity improves to Grade ≤1 or baseline, then resume study drug/study regimen after completion of steroid taper.**	**For Grade 2**<br>– **Consider symptomatic treatment, including hydration, electrolyte replacement, and diuretics.**<br>– **Carefully monitor serum creatinine every 2 to 3 days and as clinically warranted.**<br>– **Consult nephrologist and consider renal biopsy if clinically indicated.**<br>– **If event is persistent beyond 3 to 5 days or worsens, promptly start prednisone 1 to 2 mg/kg/day PO or IV equivalent.**<br>– **If event is not responsive within 3 to 5 days or worsens despite prednisone at 1 to 2 mg/kg/day PO or IV equivalent, consider additional workup.When event returns to baseline, resume study drug/study regimen and routine serum creatinine monitoring per study protocol.**

	**Grade 3 or 4**	**Permanently discontinue study drug/study regimen.**	**For Grade 3 or 4**<br>– **Carefully monitor serum creatinine daily.**<br>– **Consult nephrologist and consider renal biopsy if clinically indicated.**<br>– **Promptly start prednisone 1 to 2 mg/kg/day PO or IV equivalent.**<br>– **If event is not responsive within 3 to 5 days or worsens despite prednisone at 1 to 2 mg/kg/day PO or IV equivalent, consider additional workup and prompt treatment with an immunosuppressant in consultation with a nephrologist.**
**Rash or Dermatitis**<br><br>**(Including Pemphigoid)**	**Any Grade**<br>**(Refer to NCI CTCAE applicable version in study protocol for definition of severity/grade depending on type of skin rash)**	**General Guidance**	**For Any Grade**<br>– **Monitor for signs and symptoms of dermatitis (rash and pruritus).**<br>– **HOLD STUDY DRUG IF STEVENS-JOHNSON SYNDROME (SJS), TOXIC EPIDERMAL NECROLYSIS (TEN), OR OTHER SEVERE CUTANEOUS ADVERSE REACTION (SCAR) IS SUSPECTED.**<br>– **PERMANENTLY DISCONTINUE STUDY DRUG IF SJS, TEN, OR SCAR IS CONFIRMED.**
	**Grade 1**	**No dose modifications.**	**For Grade 1**<br>– **Consider symptomatic treatment, including oral antipruritics (e.g., diphenhydramine or hydroxyzine) and topical therapy (e.g. emolient, lotion, or institutional standard).**

	**Grade 2**	**For persistent (>1 week) Grade 2 events, hold scheduled study drug/study regimen until resolution to Grade ≤1 or baseline.**<br>· **If toxicity worsens, then treat as Grade 3.**<br>· **If toxicity improves to Grade ≤1 or baseline, then resume drug/study regimen after completion of steroid taper.**	**For Grade 2**<br>– **Obtain dermatology consult.**<br>– **Consider symptomatic treatment, including oral antipruritics (e.g., diphenhydramine or hydroxyzine) and topical therapy**<br>– **Consider moderate-strength topical steroid.**<br>– **If no improvement of rash/skin lesions occurs within 3 days or is worsening despite symptomatic treatment and/or use of moderate strength topical steroid, consider discussing with study physician, as needed, and promptly start systemic steroids such as prednisone 1 to 2 mg/kg/day PO or IV equivalent.**<br>– **Consider skin biopsy if the event persists for >1 week or recurs.**
	**Grade 3 or 4**	**For Grade 3**<br>· **Hold study drug/study regimen until resolution to Grade ≤1 or baseline.**<br>· **If toxicity improves to Grade ≤1 or baseline, then resume drug/study regimen after completion of steroid taper.**<br>· **If toxicity worsens, then treat as Grade 4.**<br><br>**For Grade 4**<br>**Permanently discontinue study drug/study regimen.**	**For Grade 3 or 4**<br>– **Consult dermatology.**<br>– **Promptly initiate empiric IV methylprednisolone 1 to 2 mg/kg/day or equivalent.**<br>– **Consider hospitalization.**<br>– **Monitor extent of rash [Rule of Nines].**<br>– **Consider skin biopsy (preferably more than 1) as clinically feasible. Consider, as necessary, discussing with study physician.**

Endocrinopathy	Any Grade	General Guidance	For Any Grade
**(e.g., hyperthyroidism, thyroiditis, hypothyroidism, type 1 diabetes mellitus, hypophysitis, hypopituitarism, and adrenal insufficiency)**	**(Depending on the type of endocrinopathy, refer to NCI CTCAE applicable version in study protocol for defining the CTC grade/severity)**		**– Consider consulting an endocrinologist for endocrine events.**<br>**– Consider discussing with study physician, as needed.**<br>**– Monitor patients for signs and symptoms of endocrinopathies. Non-specific symptoms include headache, fatigue, behaviour changes, mental status changes, photophobia, visual field cuts, vertigo, abdominal pain, unusual bowel habits, polydipsia, polyuria, hypotension, and weakness.**<br>**– Patients should be thoroughly evaluated to rule out any alternative etiology (e.g., disease progression including brain metastases, or infections).**<br>**– Depending on the suspected endocrinopathy, monitor and evaluate thyroid function tests: TSH, free T3 and free T4 and other relevant endocrine and related labs (e.g., blood glucose and ketone levels, HgA1c).If a patient experiences an AE that is thought to be possibly of autoimmune nature (e.g., thyroiditis, pancreatitis, hypophysitis, or diabetes insipidus), the investigator should send a blood sample for appropriate autoimmune antibody testing.**<br>**– Investigators should ask subjects with endocrinopathies who may require prolonged or continued hormonal replacement, to consult their primary care physicians or endocrinologists about further monitoring and treatment after completion of the study.**

	**Grade 1**	**No dose modifications.**	**For Grade 1**<br>– **Monitor patient with appropriate endocrine function tests.**<br>– **For suspected hypophysitis/hypopituitarism, consider consulting an endocrinologist to guide assessment of early-morning ACTH, cortisol, TSH and free T4; also consider gonadotropins, sex hormones, and prolactin levels, as well as cosyntropin stimulation test (though it may not be useful in diagnosing early secondary adrenal insufficiency).**<br>– **If TSH < 0.5 × LLN, or TSH >2 × ULN, or consistently out of range in 2 subsequent measurements, include free T4 at subsequent cycles as clinically indicated and consider consultation of an endocrinologist.**

	**Grade 2, 3, or 4**	· **For Grade 2-4 endocrinopathies other than hypothyroidism and type 1 diabetes mellitus, consider holding study drug/study regimen dose until acute symptoms resolve.**<br>· **Study drug/study regimen can be resumed once patient stabilizes and after completion of steroid taper.**<br>· **Patients with endocrinopathies who may require prolonged or continued steroid replacement (e.g., adrenal insufficiency) can be retreated with study drug/study regimen if the patient is clinically stable as per investigator or treating physician's clinical judgement.**<br>· **If toxicity worsens, then treat based on severity.**	**For Grade 2, 3, or 4**<br>– **Consult endocrinologist to guide evaluation of endocrine function and, as indicated by suspected endocrinopathy and as clinically indicated, consider pituitary scan.**<br>– **For all patients with abnormal endocrine work up, except those with isolated hypothyroidism or type 1 DM, and as guided by an endocrinologist, consider short-term corticosteroids (e.g., 1 to 2 mg/kg/day methylprednisolone or IV equivalent) and prompt initiation of treatment with relevant hormone replacement (e.g., hydrocortisone, sex hormones).**<br>– **Isolated hypothyroidism may be treated with replacement therapy, without study drug/study regimen interruption, and without corticosteroids.**<br>– **Isolated type 1 diabetes mellitus (DM) may be treated with appropriate diabetic therapy, and without corticosteroids. Only hold study drug/study regimen in setting of hyperglycemia when diagnostic workup is positive for diabetic ketoacidosis.**<br>– **For patients with normal endocrine workup (laboratory assessment or MRI scans), repeat laboratory assessments/MRI as clinically indicated.**

**Amylase/Lipase increased**	**Any Grade**<br>**(Refer to NCI CTCAE applicable version in study protocol for defining the CTC grade/severity)**	**General Guidance**	**For Any Grade**<br>**– For modest asymptomatic elevations in serum amylase and lipase, corticosteroid treatment is not indicated as long as there are no other signs or symptoms of pancreatic inflammation.**<br>**– Assess for signs/symptoms of pancreatitis**<br>**– Consider appropriate diagnostic testing (e.g., abdominal CT with contrast, MRCP if clinical suspicion of pancreatitis and no radiologic evidence on CT)**<br>**– If isolated elevation of enzymes without evidence of pancreatitis, continue immunotherapy. Consider other causes of elevated amylase/lipase**<br>**– If evidence of pancreatitis, manage according to pancreatitis recommendations**
	**Grade 1**	**No dose modifications.**	
	**Grade 2, 3, or 4**	**For Grade 2, 3, or 4**<br>**In consultation with relevant pancreatic specialist consider continuing study drug/study regimen if no clinical/radiologic evidence of pancreatitis ± improvement in amylase/lipase.**	
**Acute Pancreatitis**	**Any Grade**<br>**(Refer to NCI CTCAE applicable version in study protocol for defining the CTC grade/severity)**	**General Guidance**	**For Any Grade**<br>**– Consider Gastroenterology referral**
	**Grade 1**	**No dose modifications.**	**For Grade 1**<br>**– IV hydration**<br>**– Manage as per amylase/lipase increased (asymptomatic)**

	**Grade 2, 3, or 4**	**For Grade 2**<br>**Hold study drug/study regimen dose until resolution to Grade ≤1.**<br>**For Grade 3 or 4**<br>**Permanently discontinue study drug/study regimen.**	**For Grade 2, 3, or 4**<br>– **Promptly start systemic steroids prednisone 1 to 2 mg/kg/day PO or IV equivalent.**<br>– **IV hydration**
**Neurotoxicity**<br>**(to include but not limited to non-infectious meningitis, non-infectious encephalitis, and autonomic neuropathy, excluding Myasthenia Gravis and Guillain-Barre)**	**Any Grade**<br>**(Depending on the type of neurotoxicity, refer to NCI CTCAE applicable version in study protocol for defining the CTC grade/severity)**	**General Guidance**	**For Any Grade**<br>– **Patients should be evaluated to rule out any alternative etiology (e.g., disease progression, infections, metabolic syndromes, or medications).**<br>– **Monitor patient for general symptoms (headache, nausea, vertigo, behavior change, or weakness).**<br>– **Consider appropriate diagnostic testing (e.g., electromyogram and nerve conduction investigations).**<br>– **Perform symptomatic treatment with neurological consult as appropriate.**<br>– **FOR TRANSVERSE MYELITIS, PERMANENTLY DISCONTINUE FOR ANY GRADE.**
	**Grade 1**	**No dose modifications.**	**For Grade 1**<br>– **See "Any Grade" recommendations above.**

**Grade 2**	• **For acute motor neuropathies or neurotoxicity, hold study drug/study regimen dose until resolution to Grade ≤1.**<br>• **For sensory neuropathy/neuropathic pain, consider holding study drug/study regimen dose until resolution to Grade ≤1.**<br>• **Permanently discontinue study drug/study regimen if Grade 2 imAE does not resolve to Grade ≤1 within 30 days.**<br>• **If toxicity worsens, then treat as Grade 3 or 4.**	**For Grade 2**<br>– **Consider, as necessary, discussing with the study physician.**<br>– **Obtain neurology consult.**<br>– **Sensory neuropathy/neuropathic pain may be managed by appropriate medications (e.g., gabapentin or duloxetine).**<br>– **Promptly start systemic steroids prednisone 1 to 2 mg/kg/day PO or IV equivalent.**<br>– **If no improvement within 2 to 3 days despite 1 to 2 mg/kg/day prednisone PO or IV equivalent, consider additional workup and promptly treat with an additional immunosuppressant (e.g., IV IG or other immunosuppressant depending on the specific imAE).**
**Grade 3 or 4**	**For Grade 3 or 4**<br>**Permanently discontinue study drug/study regimen.**	**For Grade 3 or 4**<br>– **Consider, as necessary, discussing with study physician.**<br>– **Obtain neurology consult.**<br>– **Consider hospitalization.**<br>– **Promptly initiate empiric IV methylprednisolone 1 to 2 mg/kg/day or equivalent.**<br>– **If no improvement within 2 to 3 days despite IV corticosteroids, consider additional workup and promptly treat with an additional immunosuppressant (e.g., IV IG or other immunosuppressant depending on the specific imAE).**<br>– **Once stable, gradually taper steroids over ≥28 days.**

**Peripheral neuromotor syndromes**<br>**(such as Guillain-Barre and myasthenia gravis)**	**Any Grade**<br>**(Refer to NCI CTCAE applicable version in study protocol for defining the CTC grade/severity)**	**General Guidance**	**For Any Grade**<br>**– The prompt diagnosis of immune-mediated peripheral neuromotor syndromes is important, since certain patients may unpredictably experience acute decompensations that can result in substantial morbidity or in the worst case, death. Special care should be taken for certain sentinel symptoms that may predict a more severe outcome, such as prominent dysphagia, rapidly progressive weakness, and signs of respiratory insufficiency or autonomic instability.**<br>**– Patients should be evaluated to rule out any alternative etiology (e.g., disease progression, infections, metabolic syndromes or medications). It should be noted that the diagnosis of immune-mediated peripheral neuromotor syndromes can be particularly challenging in patients with underlying cancer, due to the multiple potential confounding effects of cancer (and its treatments) throughout the neuraxis. Given the importance of prompt and accurate diagnosis, it is essential to have a low threshold to obtain a neurological consult.**<br>**– Neurophysiologic diagnostic testing (e.g., electromyogram and nerve conduction investigations, and “repetitive stimulation” if myasthenia is suspected) are routinely indicated upon suspicion of such conditions and may be best facilitated by means of a neurology consultation.**<br>**– It is important to consider that the use of steroids as the primary treatment of Guillain-Barre is not typically considered effective. Patients requiring treatment should be started with IV IG and followed by plasmapheresis if not responsive to IV IG.**

	**Grade 1**	**No dose modifications.**	**For Grade 1**<br>– **Consider discussing with the study physician, as needed.**<br>– **Care should be taken to monitor patients for sentinel symptoms of a potential decompensation as described above.**<br>– **Consult a neurologist.**

	**Grade 2**	**Hold study drug/study regimen dose until resolution to Grade ≤1.**<br>**Permanently discontinue study drug/study regimen if it does not resolve to Grade ≤1 within 30 days or if there are signs of respiratory insufficiency or autonomic instability.**	**For Grade 2**<br>**– Consider discussing with the study physician, as needed.**<br>**– Care should be taken to monitor patients for sentinel symptoms of a potential decompensation as described above.**<br>**– Consult a neurologist.**<br>**– Sensory neuropathy/neuropathic pain may be managed by appropriate medications (e.g., gabapentin or duloxetine).**<br>**MYASTHENIA GRAVIS:**<br>**o Steroids may be successfully used to treat myasthenia gravis. It is important to consider that steroid therapy (especially with high doses) may result in transient worsening of myasthenia and should typically be administered in a monitored setting under supervision of a consulting neurologist.**<br>**o Patients unable to tolerate steroids may be candidates for treatment with plasmapheresis or IV IG. Such decisions are best made in consultation with a neurologist, taking into account the unique needs of each patient.**<br>**o If myasthenia gravis-like neurotoxicity is present, consider starting AChE inhibitor therapy in addition to steroids. Such therapy, if successful, can also serve to reinforce the diagnosis.**<br>**o Avoid medications that can worsen myasthenia gravis.**<br>**GUILLAIN-BARRE:**<br>**o It is important to consider here that the use of steroids as the primary treatment of Guillain-Barre is not typically considered effective.**<br>**o Patients requiring treatment should be started with IV IG and followed by plasmapheresis if not responsive to IV IG.**

Grade 3 or 4	For Grade 3	For Grade 3 or 4
	**·** **Hold study drug/study regimen dose until resolution to Grade ≤1.**<br>**·** **Permanently discontinue study drug/study regimen if Grade 3 imAE does not resolve to Grade ≤1 within 30 days or if there are signs of respiratory insufficiency or autonomic instability.**<br><br>**For Grade 4**<br>**Permanently discontinue study drug/study regimen.**	**–** **Consider discussing with study physician, as needed.**<br>**–** **Recommend hospitalization.**<br>**–** **Monitor symptoms and consult a neurologist.**<br>MYASTHENIA GRAVIS:<br>**o** **Steroids may be successfully used to treat myasthenia gravis. They should typically be administered in a monitored setting under supervision of a consulting neurologist.**<br>**o** **Patients unable to tolerate steroids may be candidates for treatment with plasmapheresis or IV IG.**<br>**o** **If myasthenia gravis-like neurotoxicity present, consider starting AChE inhibitor therapy in addition to steroids. Such therapy, if successful, can also serve to reinforce the diagnosis.**<br>**o** **Avoid medications that can worsen myasthenia gravis.**<br>GUILLAIN-BARRE:<br>**o** **It is important to consider here that the use of steroids as the primary treatment of Guillain-Barre is not typically considered effective.**<br>**o** **Patients requiring treatment should be started with IV IG and followed by plasmapheresis if not responsive to IV IG.**

Myocarditis	Any Grade	General Guidance	For Any Grade
	(Refer to NCI CTCAE applicable version in study protocol for defining the CTC grade/severity)	Discontinue drug permanently if biopsy-proven immune-mediated myocarditis.	– The prompt diagnosis of immune-mediated myocarditis is important, particularly in patients with baseline cardiopulmonary disease and reduced cardiac function.<br>– Consider discussing with the study physician, as needed.<br>– Monitor patients for signs and symptoms of myocarditis (new onset or worsening chest pain, arrhythmia, shortness of breath, peripheral edema). As some symptoms can overlap with lung toxicities, simultaneously evaluate for and rule out pulmonary toxicity as well as other causes (e.g., pulmonary embolism, congestive heart failure, malignant pericardial effusion). Consult a cardiologist early, to promptly assess whether and when to complete a cardiac biopsy, including any other diagnostic procedures.<br>– Initial work-up should include clinical evaluation, BNP, cardiac enzymes, ECG, echocardiogram (ECHO), monitoring of oxygenation via pulse oximetry (resting and exertion), and additional laboratory work-up as indicated. Spiral CT or cardiac MRI can complement ECHO to assess wall motion abnormalities when needed.<br>– Patients should be thoroughly evaluated to rule out any alternative etiology (e.g., disease progression, other medications, or infections)

	**Grade 1**	**No dose modifications required unless clinical suspicion is high, in which case hold study drug/study regimen dose during diagnostic work-up for other etiologies. If study drug/study regimen is held, resume after complete resolution to Grade 0.**	**For Grade 1**<br>**- Monitor and closely follow up in 2 to 4 days for clinical symptoms, BNP, cardiac enzymes, ECG, ECHO, pulse oximetry (resting and exertion), and laboratory work-up as clinically indicated.**<br>**- Consider using steroids if clinical suspicion is high.**
	**Grade 2, 3 or 4**	**· If Grade 2 -- Hold study drug/study regimen dose until resolution to Grade 0. If toxicity rapidly improves to Grade 0, then the decision to reinitiate study drug/study regimen will be based upon treating physician's clinical judgment and after completion of steroid taper. If toxicity does not rapidly improve, permanently. discontinue study drug/study regimen.**<br>**· If Grade 3-4, permanently discontinue study drug/study regimen.**	**For Grade 2-4**<br>**– Monitor symptoms daily, hospitalize.**<br>**– Promptly start IV methylprednisolone 2 to 4 mg/kg/day or equivalent after Cardiology consultation has determined whether and when to complete diagnostic procedures including a cardiac biopsy.**<br>**– Supportive care (e.g., oxygen).**<br>**– If no improvement within 2 to 3 days despite IV methylprednisolone at 2 to 4 mg/kg/day, promptly start immunosuppressive therapy such as TNF inhibitors (e.g., infliximab at 5 mg/kg IV, may be repeated at 2 and 6 weeks after initial dose at the discretion of the treating provider). Caution: It is important to rule out sepsis and refer to infliximab label for general guidance before using infliximab. Infliximab is contraindicated for patients who have heart failure.**

**Myositis/ Polymyositis**	**Any Grade (Refer to NCI CTCAE applicable version in study protocol for defining the CTC grade/severity)**	**General Guidance**	**For Any Grade**
			– Monitor patients for signs and symptoms of poly/myositis. Typically, muscle weakness/pain occurs in proximal muscles including upper arms, thighs, shoulders, hips, neck and back, but rarely affects the extremities including hands and fingers; also difficulty breathing and/or trouble swallowing can occur and progress rapidly. Increased general feelings of tiredness and fatigue may occur, and there can be new-onset falling, difficulty getting up from a fall, and trouble climbing stairs, standing up from a seated position, and/or reaching up.<br>– If poly/myositis is suspected, a Neurology consultation should be obtained early, with prompt guidance on diagnostic procedures. Myocarditis may co-occur with poly/myositis; refer to guidance under Myocarditis. Given breathing complications, refer to guidance under Pneumonitis/ILD.<br>Given possibility of an existent (but previously unknown) autoimmune disorder, consider Rheumatology consultation.<br>– Consider, as necessary, discussing with the study physician.<br>– Initial work-up should include clinical evaluation, creatine kinase, aldolase, LDH, BUN/creatinine, erythrocyte sedimentation rate or C-reactive protein level, urine myoglobin, and additional laboratory work-up as indicated, including a number of possible rheumatological/antibody tests (i.e., consider whether a rheumatologist consultation is indicated and could guide need for rheumatoid factor, antinuclear antibody, anti-smooth muscle, antisynthetase [such as anti-Jo-1], and/or signal-recognition particle antibodies). Confirmatory testing may include electromyography,

nerve conduction studies, MRI of the muscles, and/or a muscle biopsy. Consider Barium swallow for evaluation of dysphagia or dysphonia.

– **Patients should be thoroughly evaluated to rule out any alternative etiology (e.g., disease progression, other medications, or infections).**

**Grade 1**	· **No dose modifications.**	**For Grade 1**<br>– **Monitor and closely follow up in 2 to 4 days for clinical symptoms and initiate evaluation as clinically indicated.**<br>– **Consider Neurology consult.**<br>– **Consider, as necessary, discussing with the study physician.**

	**Grade 2**	• **Hold study drug/study regimen dose until resolution to Grade ≤1.**<br>• **Permanently discontinue study drug/study regimen if it does not resolve to Grade ≤1 within 30 days or if there are signs of respiratory insufficiency.**	**For Grade 2**<br>– **Monitor symptoms daily and consider hospitalization.**<br>– **Obtain Neurology consult, and initiate evaluation.**<br>– **Consider, as necessary, discussing with the study physician.**<br>– **If clinical course is rapidly progressive (particularly if difficulty breathing and/or trouble swallowing), promptly start IV methylprednisolone 2 to 4 mg/kg/day systemic steroids along with receiving input from Neurology consultant**<br>– **If clinical course is not rapidly progressive, start systemic steroids (e.g., prednisone 1 to 2 mg/kg/day PO or IV equivalent); if no improvement within 2 to 3 days, continue additional work up and start treatment with IV methylprednisolone 2 to 4 mg/kg/day**<br>– **If after start of IV methylprednisolone at 2 to 4 mg/kg/day there is no improvement within 2 to 3 days, consider starting another immunosuppressive therapy such as a TNF inhibitor (e.g., infliximab at 5 mg/kg IV, may be repeated at 2 and 6 weeks after initial dose at the discretion of the treating provider). Caution: It is important to rule out sepsis and refer to infliximab label for general guidance before using infliximab.**

GETNE
Grupo Español de Tumores Neuroendocrinos y Endocrinos

	**Grade 3 or 4**	**For Grade 3**<br>· **Hold study drug/study regimen dose until resolution to Grade ≤1.**<br>· **Permanently discontinue study drug/study regimen if Grade 3 imAE does not resolve to Grade ≤1 within 30 days or if there are signs of respiratory insufficiency.**<br><br>**For Grade 4**<br>· **Permanently discontinue study drug/study regimen.**	**For Grade 3 or 4**<br>– **Monitor symptoms closely; recommend hospitalization.**<br>– **Obtain Neurology consult**<br>– **Consider discussing with the study physician, as needed.**<br>– **Promptly start IV methylprednisolone 2 to 4 mg/kg/day systemic steroids along with receiving input from Neurology consultant.**<br>– **If after start of IV methylprednisolone at 2 to 4 mg/kg/day there is no improvement within 2 to 3 days, consider starting another immunosuppressive therapy such as a TNF inhibitor (e.g., infliximab at 5 mg/kg IV, may be repeated at 2 and 6 weeks after initial dose at the discretion of the treating provider). Caution: It is important to rule out sepsis and refer to infliximab label for general guidance before using infliximab.**<br>– **Consider whether patient may require IV IG, plasmapheresis.**

aASCO Educational Book 2015 "Managing Immune Checkpoint Blocking Antibody Side Effects" by Michael Postow MD.
bFDA Liver Guidance Document 2009 Guidance for Industry: Drug Induced Liver Injury – Premarketing Clinical Evaluation.

cNCCN Clinical Practice Guidelines in Oncology "Management of Immunotherapy-Related Toxicities" Version 1.2020 – December 2019

AChE Acetylcholine esterase; ADL Activities of daily living; AE Adverse event; ALP Alkaline phosphatase test; ALT Alanine aminotransferase; AST Aspartate aminotransferase; BUN Blood urea nitrogen; CT Computed tomography; CTCAE Common Terminology Criteria for Adverse Events; ILD Interstitial lung disease; imAE immune-mediated adverse event; IG Immunoglobulin; IV Intravenous; GI Gastrointestinal; LFT Liver function tests; LLN Lower limit of normal; MRI Magnetic resonance imaging; NCI National Cancer Institute; NCCN National Comprehensive Cancer Network; PJP Pneumocystis jirovecii pneumonia (formerly known as Pneumocystis carinii pneumonia); PO By mouth; T3 Triiodothyronine; T4 Thyroxine; TB Total bilirubin; TNF Tumor necrosis factor; TSH Thyroid-stimulating hormone; ULN Upper limit of normal.

Other–Immune-Mediated Reactions

Severity Grade of the Event (Refer to NCI CTCAE applicable version in study protocol for defining the CTC grade/severity)	Dose Modifications	Toxicity Management
Any Grade	Note: It is possible that events with an inflammatory or immune mediated mechanism could occur in nearly all organs, some of them are not noted specifically in these guidelines (e.g. immune thrombocytopenia, haemolytic anaemia, uveitis, vasculitis).	– The study physician may be contacted for immune-mediated reactions not listed in the “specific immune-mediated reactions” section<br>– Thorough evaluation to rule out any alternative etiology (e.g., disease progression, concomitant medications, and infections)<br>– Consultation with relevant specialist<br>– Treat accordingly, as per institutional standard.
Grade 1	No dose modifications.	Monitor as clinically indicated
Grade 2	· Hold study drug/study regimen until resolution to ≤Grade 1 or baseline.<br>· If toxicity worsens, then treat as Grade 3 or Grade 4.<br>· Study drug/study regimen can be resumed once event stabilizes to Grade ≤1 after completion of steroid taper.<br>· Consider whether study drug/study regimen should be permanently discontinued in Grade 2 events with high likelihood for morbidity and/or mortality when they do not rapidly improve to Grade <1 upon treatment with systemic steroids and following full taper	For Grade 2, 3, or 4<br>Treat accordingly, as per institutional standard, appropriate clinical practice guidelines, and other society guidelines (e.g., NCCN, ESMO)
Grade 3	Hold study drug/study regimen	

**Grade 4**	**Permanently discontinue study drug/study regimen**

Note: As applicable, for early phase studies, the following sentence may be added: "Any event greater than or equal to Grade 2, please discuss with Study Physician."
AE Adverse event; CTCAE Common Terminology Criteria for Adverse Events; NCI National Cancer Institute.

Infusion-Related Reactions

Severity Grade of the Event (Refer to NCI CTCAE applicable version in study protocol for defining the CTC grade/severity)	Dose Modifications	Toxicity Management
**Any Grade**	**General Guidance**	**For Any Grade**<br>– Manage per institutional standard at the discretion of investigator.<br>– Monitor patients for signs and symptoms of infusion-related reactions (e.g., fever and/or shaking chills, flushing and/or itching, alterations in heart rate and blood pressure, dyspnea or chest discomfort, or skin rashes) and anaphylaxis (e.g., generalized urticaria, angioedema, wheezing, hypotension, or tachycardia).
**Grade 1 or 2**	**For Grade 1**<br>The infusion rate of study drug/study regimen may be decreased by 50% or temporarily interrupted until resolution of the event.<br>**For Grade 2**<br>· The infusion rate of study drug/study regimen may be decreased 50% or temporarily interrupted until resolution of the event.<br>· Subsequent infusions may be given at 50% of the initial infusion rate.	**For Grade 1 or 2**<br>– Acetaminophen and/or antihistamines may be administered per institutional standard at the discretion of the investigator.<br>– Consider premedication per institutional standard prior to subsequent doses.<br>– Steroids should not be used for routine premedication of Grade ≤2 infusion reactions.

GETNE Grupo Español de Tumores Neuroendocrinos y Endocrinos

**Grade 3 or 4**	**For Grade 3 or 4**<br>**Permanently discontinue study drug/study regimen.**	**For Grade 3 or 4**<br>– **Manage severe infusion-related reactions per institutional standards (e.g., IM epinephrine, followed by IV diphenhydramine and famotidine, and IV glucocorticoid).**

CTCAE Common Terminology Criteria for Adverse Events; IM intramuscular; IV intravenous; NCI National Cancer Institute.

Non-Immune-Mediated Reactions

Severity Grade of the Event (Refer to NCI CTCAE applicable version in study protocol for defining the CTC grade/severity)	Dose Modifications	Toxicity Management
**Any Grade**	**Note: Dose modifications are not required for AEs not deemed to be related to study treatment (i.e., events due to underlying disease) or for laboratory abnormalities not deemed to be clinically significant.**	**Treat accordingly, as per institutional standard.**
**Grade 1**	**No dose modifications.**	**Treat accordingly, as per institutional standard.**
**Grade 2**	**Hold study drug/study regimen until resolution to ≤Grade 1 or baseline.**	**Treat accordingly, as per institutional standard.**
**Grade 3**	**Hold study drug/study regimen until resolution to ≤Grade 1 or baseline.**<br>**For AEs that downgrade to ≤Grade 2 within 7 days or resolve to ≤Grade 1 or baseline within 14 days, resume study drug/study regimen administration. Otherwise, discontinue study drug/study regimen.**	**Treat accordingly, as per institutional standard.**

**Grade 4**	**Discontinue study drug/study regimen (Note: For Grade 4 labs, decision to discontinue should be based on accompanying clinical signs/symptoms, the Investigator's clinical judgment, and consultation with the Sponsor.).**	**Treat accordingly, as per institutional standard.**

Note: As applicable, for early phase studies, the following sentence may be added: "Any event greater than or equal to Grade 2, please discuss with Study Physician."
AE Adverse event; CTCAE Common Terminology Criteria for Adverse Events; NCI National Cancer Institute.

Appendix 2 Schedule of study procedures follow-up for subjects who have completed durvalumab and tremelimumab treatment and achieved disease control (until confirmed progression of disease) and subjects who have discontinued durvalumab or tremelimumab due to toxicity in the absence of confirmed progression of disease.

Evaluation	Time Since Last Dose							
	Day (±3)	Months (±1 week)						12 Months and every 6 Months (±2 weeks)
	30	2	3	4	6	8	10	
Physical examination[a]	x	x	x	x	x	x	x	
Vital signs (temperature, respiratory rate, blood, pressure, pulse)	x	x	x	x	x	x	x	
Weight	x	x	x	x	x	x	x	
Urine hCG or serum βhCG	x							
AE/SAE assessment	x	x	x					
Concomitant medications	x	x	x					
Palliative radiotherapy	As clinically indicated							
ECOG performance status	x	x	x		X (and month 9)			x
Subsequent anti/cancer therapy	x	x	x	x	x	x	x	x
Survival status: phone contact with subjects who refuse to return for evaluations and agree to be contacted		x	x	x	x	x	x	x (every 2 months)
Hematology	x	Every 8-12 weeks, according to investigator's criteria						x
Serum chemistry	x	Every 8-12 weeks, according to investigator's criteria						
Thyroid function tests)TSH, and fT3 and fT4)[b]	x	Every 8-12 weeks, according to investigator's criteria						
SPD-L1 concentration)to assess target engagement)			x					
Tumour assessment (CT or MRI)	For subjects who **achieve disease control following 12 months of treatment**, tumour assessments should be performed every 12 weeks relative to the date of **first infusion** thereafter until confirmed PD by RECIST 1.1 by investigational site review. Please refer to SCHEDULE OF STUDY ASSESSMENTS for timings of confirmatory scans.<br>For subjects who **discontinue study drug due to toxicity (or symptomatic deterioration)**, tumour assessments should be performed relative to the relative to the date of first infusion as follows: every 12 weeks until confirmed PD by RECIST 1.1 by investigational site review. Please refer to SCHEDULE OF STUDY ASSESSMENTS for timings of confirmatory scans.<br>Upon confirmed PD, scans should be conducted according to local standard clinical practice until a new treatment is started (these scans are optional).							

a Full physical exam
b Free T3 and free T4 will only be measured if TSH is abnormal. They should also be measured if there is clinical suspicion of an adverse event related to the endocrine system.
c For patient questionnaires different approaches based on indication and study design.

Appendix 3 Schedule of study procedures: follow-up for subjects who have discontinued durvalumab or tremelimumab treatment due to confirmed progression of disease at the investigator discretion

Evaluation	Time Since Last Dose							
	Day (±3)	Months (±1 week)						12 Months and every 6 Months (±2 weeks)
	30	2	3	4	6	8	10	
Physical examination[a]	x							
Vital signs (temperature, respiratory rate, blood, pressure, pulse)	x							
Weight	x							
AE/SAE assessment	x	x	x					
Concomitant medications	x	x	x					
Palliative radiotherapy	As clinically indicated							
ECOG performance status[b]	x	x	x					
Subsequent anti/cancer therapy	x	x	x	x	x	x	x	x
Survival status: phone contact with subjects who refuse to return for evaluations and agree to be contacted		x	x	x	x	x	x	x (every 2 months)
Urine hCG or serum βhCG	x							
Hematology	x	x	x					
Serum chemistry	x	x	x					
Thyroid function tests)TSH, and fT3 and fT4)[c]			x					
Pharmacokinetic assessment, if applicable			x					
SPD-L1 concentration)to assess target engagement)			x					
Tumour assessment (CT or MRI)	For subjects who continue on MEDI4736 post-confirmed progression at the investigator's discretion (following consultation with the sponsor), tumour assessments should be performed relative to the date of first infusion per SCHEDULE OF STUDY ASSESSMENTS until study drug is stopped.<br>For subjects who discontinue study drug following confirmed progression, scans should be conducted according to local clinical practice until a new treatment is started (these scans are optional).							

[A] Full physical exam
[B] PS to be collected if available at the 2 monthly calls to obtain subsequent anti/cancer therapy and survival status
c Free T3 and free T4 will only be measured if TSH is abnormal. They should also be measured if there is clinical suspicion of an adverse event related to the endocrine system.
[D] For patient questionnaires different approaches based on indication and study design.

Appendix 4 Durvalumab dose calculations

For durvalumab done depending on subject weight:

1. Cohort dose: X mg/kg

2. Subject weight: Y kg

3. Dose for subject: XY mg = X(mg/kg) x Y(kg)

4. Dose to be added into infusion bag:

Dose (mL) = XY mg / 50 (mg/mL)

Where 50 mg/mL is durvalumab nominal concentration.

The corresponding volume of durvalumab should be rounded to the nearest tenth mL (0.1 mL). Dose adjustments for each cycle are only needed for greater than 10% change in weight.

5. The theoretical number of vials required for dose preparation is the next greatest whole number of vials from the following formula:

Number of vials = Dose (mL) / 10.0 (mL/vial)

Example:

1. Cohort dose: 10 mg/kg

2. Subject weight: 30 kg

3. Dose for subject: 300 mg = 10 (mg/kg) x 30(kg)

4. Dose to be added into infusion bag:

Dose (mL) = 300 mg / 50 (mg/mL) = 6.0 mL

5. The theoretical number of vials required for dose preparation:

Number of vials = 6.0(mL) / 10.0 (mL/vial) = 1 vials

Appendix 5 Durvalumab Dose Volume Calculations

For durvalumab flat dosing:

1. Cohort dose: X g

2. Dose to be added into infusion bag:

Dose (mL) = X g x 1000/50 (mg/mL)

where 50 mg/mL is durvalumab nominal concentration.

The corresponding volume of durvalumab should be rounded to the nearest tenth mL (0.1 mL)

3. The theoretical number of vials required for dose preparation is the next greatest whole number of vials from the following formula:

Number of vials = Dose (mL) / 10.0 (mL/vial)

Example:

1. Cohort dose: 1.5 g

2. Dose to be added into infusion bag:

Dose (mL) = 1.5 g x 1000 / 50 (mg/mL) = 30.0 mL

3. The theoretical number of vials required for dose preparation:

Number of vials = 30.0 (mL) / 10.0 (mL/vial) = 3 vials

Appendix 6 Tremelimumab Dose Calculations

For tremelimumab dosing done depending on subject weight:

1. Cohort dose: X mg/kg

2. Subject weight: Y kg

3. Dose for subject: XY mg = X(mg/kg) x Y(kg)

4. Dose to be added into infusion bag:

Dose (mL) = XY mg / 20 (mg/mL)

Where 20 mg/mL is tremelimumab nominal concentration.

The corresponding volume of tremelimumab should be rounded to the nearest tenth mL (0.1 mL). Dose adjustments for each cycle are only needed for greater then 10% change in weight.

5. The theoretical number of vials required for dose preparation is the next greatest whole number of

vials from the following formula:

Number of vials = Dose (mL) / 20.0 (mL/vial)

Or

Number of vials = Dose (mL) / 1.25 (mL/vial)

Example:

1. Cohort dose: 1 mg/kg

2. Subject weight: 30 kg

3. Dose for subject: 30 mg = 1 (mg/kg) x 30(kg)

4. Dose to be added into infusion bag:

Dose (mL) = 30 mg / 20 (mg/mL) = 1.5 mL

5. The theoretical number of vials required for dose preparation:

Number of vials = 1.5(mL) / 20.0 (mL/vial) = 1 vials

or

Number of vials = 1.5(mL) / 1.25 (mL/vial) = 2 vials

Appendix 7 Tremelimumab Dose Volume Calculations

For tremelimumab flat dosing:

1. Cohort dose: X mg

2. Dose to be added into infusion bag:

Dose (mL) = X mg /20 (mg/mL)

where 20 mg/mL is tremelimumab nominal concentration.

The corresponding volume of tremelimumab should be rounded to the nearest tenth mL (0.1 mL)

3. The theoretical number of vials required for dose preparation is the next greatest whole number of

vials from the following formula:

Number of vials = Dose (mL) / 20 (mL/vial)

or

Number of vials = Dose (mL) / 1.25 (mL/vial)

Example:

1. Cohort dose: 75 mg

2. Dose to be added into infusion bag:

Dose (mL) = 75 mg / 20 (mg/mL) = 3.8 mL

3. The theoretical number of vials required for dose preparation:

Number of vials = 3.8 (mL) / 20 (mL/vial) = 1 vial

or

Number of vials = 3.8 (mL) / 1.25 (mL/vial) = 3 vials

Appendix 8 Contact Details

Sponsor: GETNE (Spanish Group of Neuroendocrine and Endocrine Tumors)

C/ Secretari Coloma, 64-68 esc. B entresuelo 5ª

08024 Barcelona

Phone: + 34 93 434 44 12

e-mail: getne@getne.org

Coordinating Investigator: [redacted]

CRO: [redacted]

Appendix 9 Sites and Investigators Participants

	SITE	Principal Investigator
[redacted]	[redacted]	[redacted]
[redacted]	[redacted]	[redacted]
[redacted]	[redacted]	[redacted]
[redacted]	[redacted]	[redacted]
[redacted]	[redacted]	[redacted]
[redacted]	[redacted]	[redacted]
[redacted]	[redacted]	[redacted]
[redacted]	[redacted]	[redacted]
[redacted]	[redacted]	[redacted]
[redacted]	[redacted]	[redacted]
[redacted]	[redacted]	[redacted]
[redacted]	[redacted]	[redacted]
[redacted]	[redacted]	[redacted]
[redacted]	[redacted]	[redacted]
[redacted]	[redacted]	[redacted]
[redacted]	[redacted]	[redacted]
[redacted]	[redacted]	[redacted]
[redacted]	[redacted]	[redacted]
[redacted]	[redacted]	[redacted]
[redacted]	[redacted]	[redacted]

GETNE
Grupo Español de Tumores Neuroendocrinos y Endocrinos

Investigational Drug Substance (s)	Durvalumab (MEDI4736) and Tremelimumab
Study Number	ESR 15-11561-61-DUNE
Version Number	4.0
Date	APRIL 09th, 2020

A PHASE II STUDY OF DURVALUMAB (MEDI4736) PLUS TREMELIMUMAB FOR THE TREATMENT OF PATIENTS WITH ADVANCED NEUROENDOCRINE NEOPLASMS OF GASTROENTEROPANCREATIC OR LUNG ORIGIN (THE DUNE TRIAL)

Sponsor: GETNE

(Spanish Group of Neuroendocrine and Endocrine Tumors)

[redacted]

Coordinating Investigator: Dr. Jaume Capdevila

[redacted]

SPONSOR'S SIGNATURE PAGE

Study title: A phase II study of durvalumab (MEDI4736) plus tremelimumab for the treatment of patients with advanced neuroendocrine neoplasms of gastroenteropancreatic or lung origin (the DUNE trial).

Study code: ESR 15-11561-61 – DUNE

EudraCT Number: 2016-002858-20

Version number and date: 4.0, April 09th,2020

[redacted]

GETNE Chairman	Signature	Signature date (DD-mm-YYYY)

[redacted]

Coordinating Investigator	Signature	Signature date (DD-mm-YYYY)

GETNE
Grupo Español de Tumores
Neuroendocrinos y Endocrinos

INVESTIGATOR'S SIGNATURE PAGE

Study title: A phase II study of durvalumab (MEDI4736) plus tremelimumab for the treatment of patients with advanced neuroendocrine neoplasms of gastroenteropancreatic or lung origin (the DUNE trial).

Study code: ESR 15-11561-61 – DUNE

EudraCT Number: 2016-002858-20

Version number and date: 4.0, April 09th ,2020

____
Principal Investigator's signature

____
Signature date
(DD-mm-YYYY)

____
Investigator's name (capital letters)

PROTOCOL SYNOPSIS

Clinical Protocol ESR 15-11561-61

**Study Title:** A phase II study of durvalumab (MEDI4736) plus tremelimumab for the treatment of patients with advanced neuroendocrine neoplasms of gastroenteropancreatic or lung origin (the DUNE trial)
**Protocol Number:** ESR 15-11561-61<br><br>**EudraCT Number:** 2016-002858-20
**Clinical Phase:** II
**Study Duration:** 3 years
**Investigational Product(s) and Reference Therapy:**<br><br>Durvalumab will be supplied in glass vials containing 500 mg of liquid solution at a concentration of 50 mg/mL for intravenous (IV) administration.<br><br>Tremelimumab is supplied as a sterile solution for IV infusion, filled in 20 mL clear glass vials with a rubber stopper and aluminum seal. Each vial contains 20 mg/mL (with a nominal fill of 20 mL, accounting to 400 mg/vial or with a nominal fill of 1.25 mL, accounting to 25 mg/vial) of tremelimumab, in an isotonic solution at pH 5.5.
**Research Hypothesis**<br><br>Well-differentiated gastroenteropancreatic and lung neuroendocrine tumors are generally malignancies with a prolonged natural history. However, clinical behavior is heterogeneous and when tumor progression is observed, treatment options are limited. The most used therapy for neuroendocrine tumors management are somatostatin analogs, with clear evidence of antitumor activity demonstrated by two phase III clinical trials in gastroenteropancreatic neuroendocrine tumors. However, even the use in lung carcinoids is quite usual, no antitumoral activity has been demonstrated in prospective clinical trials. In recent years, targeted therapies as sunitinib and everolimus have been approved for pancreatic neuroendocrine tumors and recently everolimus have showed a significant reduction in the risk of progression or death in patients with non-functional neuroendocrine tumors of lung and gastrointestinal origin. After the failure of these therapies, no drugs have demonstrated efficacy. Only Interferon alpha-2b is an option for these patients who have worsening symptoms of carcinoid syndrome while on treatment with somatostatin analogs and have showed some evidence of antitumoral activity in neuroendocrine tumors. The efficacy of Interferon alpha 2-b is not fully understood, but one of its antitumoral mechanisms of action could be related via stimulation of T cells. Tremelimumab and Durvalumab combination could be more efficient drugs to improve immune system activation and could obtain a significantly higher clinical benefit in these patients. We have analyzed presence of tumor-infiltrating lymphocytes (TILS) in these tumor types. We found a high number of TILS that could be activated through Tremelimumab and Durvalumab. In cases of advanced high-grade G3 neuroendocrine carcinomas (NEC), regardless of the site of the primary tumor, combination chemotherapy using cisplatin/etoposide is recommended in first-line setting (provided that the patient has adequate organ function and performance status). There is no established second-line therapy for poorly differentiated neuroendocrine carcinomas of the

gastroenteropancreatic origin.

The programmed death 1 (PD-1) receptor and PD-1 ligands 1 and 2 (PD-L1, PD-L2) play integral roles in immune regulation. Expressed on activated T cells, PD-1 is activated by PD-L1 and PD-L2 expressed by stromal cells, tumor cells, or both, initiating T-cell death and localized immune suppression, potentially providing an immune-tolerant environment for tumor development and growth. Conversely, inhibition of this interaction can enhance local T-cell responses and mediate antitumor activity in nonclinical animal models.

Tremelimumab and Durvalumab would be the first immune combination agents showing efficacy in neuroendocrine neoplasms of different origins.

Objectives:

Primary Objectives:

Primary endpoint for cohorts 1, 2 and 3:

Nine-months clinical benefit rate (CBR) by Response Evaluation Criteria In Solid Tumors (RECIST 1.1), which is defined as the percentage of patients achieving complete response (CR), partial response (PR), or stable disease (SD) at month 9 after durvalumab plus tremelimumab was started.

Primary endpoint for cohort 4:

Nine-months overall survival rate, which is defined as the percentage of patients alive at month 9 after durvalumab plus tremelimumab was started.

Secondary Objective(s):

- Overall response rate (ORR) by irRECIST.
- To assess the duration of response according to irRECIST.
- To assess the median progression-free survival time (PFS) according to irRECIST.
- To assess the safety profile of Durvalumab and Tremelimumab in subjects with advanced neuroendocrine neoplasms.
- To assess the median overall survival (OS) time.
- To assess response status according to irRECIST at 6, 9 and 12 months after start of study treatment.

Exploratory Objective(s):

- To evaluate biochemical response (changes in CgA and NSE levels) and its association with response rate and progression-free survival.
- To assess whether baseline tumor and blood biomarkers may be predictive of response to durvalumab and tremelimumab therapy.
- To explore additional hypotheses related to biomarkers and relationship to durvalumab and tremelimumab efficacy and/or toxicity and neuroendocrine tumors evolution that may

arise from internal or external research activities.

Study Design:

Prospective, multi-center, open label, stratified, exploratory, phase II study evaluating the efficacy and safety of durvalumab plus tremelimumab in different cohorts of patients with neuroendocrine neoplasms.

The study will include patients in four different cohorts:

- **Cohort 1**: Well-moderately differentiated lung neuroendocrine tumors (classically known as typical and atypical carcinoids) after progression to somatostatin analogs and one prior targeted therapy or chemotherapy.
- **Cohort 2**: G1/G2 (WHO grade 1 and 2) gastrointestinal neuroendocrine tumors after progression to somatostatin analogs and one prior targeted therapy.
- **Cohort 3**: G1/G2 (WHO grade 1 and 2) pancreatic neuroendocrine tumors after progression to standard therapies (chemotherapy, somatostatin analogs and target therapy), who have received between two and four prior lines of treatment.
- **Cohort 4**: Neuroendocrine neoplasms (WHO grade 3) of gastroenteropancreatic origin or unknown primary site (excluding lung primary tumors), patients will be treated in second line only, after progression to first-line chemotherapy with a platinum based regimen.

Number of Centers: 20

Number of Subjects: 126 patients: 31 patients for cohorts 1 to 3, and 33 for cohort 4.

Study Population:

Patients with advanced/metastatic, histologically confirmed, grade 1/2 (G1/G2) of the 2010 WHO classification neuroendocrine tumors of the pancreas, gastrointestinal tract and lung origins and grade 3 (G3) of gastroenteropancreatic system or unknown primary site (excluding lung primaries) after progression to previous therapies.

Inclusion Criteria:

For inclusion in the study, patients should fulfill the following criteria:

1. Written informed consent obtained from the subject prior to performing any protocol-related procedures.
2. Age >18 years at time of study entry.
3. Subjects must have histologically confirmed diagnosis of one of the following advanced/metastatic neuroendocrine tumor types:
   a) **Cohort 1**: Well-moderately differentiated neuroendocrine tumors of the lung (mitotic count ≤10 mitoses x 10 HPF), also known as typical and atypical lung carcinoids, that have progressed to prior somatostatin analog therapy and/or one prior targeted therapy or chemotherapy (only one prior systemic therapy, with the exception of patients that have been treated with somatostatin analogues and other systemic

treatment, when two prior treatments are allowed).

b) **Cohort 2**: Well-moderately differentiated G1/G2 (WHO grade 1 and 2) gastrointestinal neuroendocrine tumors after progression to somatostatin analogs and one targeted therapy (prior targeted therapy could be everolimus or a multikinase inhibitor). Prior therapies with interferon alpha-2b or radionucleotide therapy are allowed.

c) **Cohort 3**: Well-moderately differentiated neuroendocrine tumors G1/G2 (WHO grade 1 and 2) from pancreatic origin after progression to standard therapies (chemotherapy, somatostatin analogs and target therapy); patients must be treated with at least two prior systemic treatment lines and a maximum of four previous treatment lines.

d) **Cohort 4**: Neuroendocrine neoplasms (WHO grade 3) of gastroenteropancreatic origin or unknown primary site (excluding lung primary tumors), patients will be treated in second line only, after progression to first-line chemotherapy with a platinum based regimen.

4. For patients included in cohorts 1, 2 and 3: WHO Classification G1/G2 (mitotic count ≤10 mitoses x 10 HPF) lung typical and atypical carcinoids for cohort 1, G1/G2 (Ki67≤20% or mitotic count ≤20 mitoses x 10 HPF) gastrointestinal for cohort 2 (including stomach, small intestine and colorectal origins), G1/G2 (Ki67≤20% or mitotic count ≤20 mitoses x 10 HPF) pancreatic for cohort 3.
5. For patients included in cohort 4: WHO classification G3 (Ki67>20% or mitotic count >20 mitoses x 10 HPF) gastroenteropancreatic neuroendocrine carcinomas (NEC) or liver metastases of G3 NEC of unknown primary site.
6. Subjects must have evidence of measurable disease meeting the following criteria:

a) In case of more than one target lesion, it should be identified at least 1 lesion of ≥ 1.0 cm in the longest diameter for a non lymph node, or ≥ 1.5 cm in the short-axis diameter for a lymph node, which is serially measurable according to RECIST 1.1 using computerized tomography/magnetic resonance imaging (CT/MRI). If there is only one target lesion and it is a non-lymph node, it should have a longest diameter of ≥ 1.5 cm.

b) Lesions that have had external beam radiotherapy (EBRT) or loco-regional therapies such as radiofrequency (RF) ablation or liver embolization must show evidence of progressive disease based on RECIST 1.1 to be deemed a target lesion.

c) Subjects must show evidence of disease progression by radiologic image techniques within 12 months (an additional month will be allowed to accommodate actual dates of performance of scans, i.e., within ≤ 13 months) prior to signing informed consent, according to RECIST 1.1.

7. Eastern Cooperative Oncology Group (ECOG) performance status of 0 or 1.
8. Life expectancy of at least 12 weeks.
9. Adequate normal organ and marrow function as defined below:

- Haemoglobin ≥ 9.0 g/dL.
- Absolute neutrophil count (ANC) ≥ 1.5 x 10^9/L (≥ 1500 per mm^3).
- Platelet count ≥ 100 x 10^9/L (≥100,000 per mm^3).

10. Serum bilirubin ≤ 1.5 x institutional upper limit of normal (ULN). This will not apply to subjects with confirmed Gilbert's syndrome (persistent or recurrent hyperbilirubinemia that is predominantly unconjugated in the absence of hemolysis or hepatic pathology), who will be allowed only in consultation with their physician.
11. AST (SGOT)/ALT (SGPT) ≤ 2.5 x institutional upper limit of normal unless liver metastases are present, in which case it must be ≤ 5x ULN.
12. Serum creatinine CL>40 mL/min by the Cockcroft-Gault formula (Cockcroft and Gault 1976) or by 24-hour urine collection for determination of creatinine clearance:

Males:

$$\text{Creatinine CL (mL/min)} = \frac{\text{Weight (kg)} \times (140 - \text{Age})}{72 \times \text{serum creatinine (mg/dL)}}$$

Females:

$$\text{Creatinine CL (mL/min)} = \frac{\text{Weight (kg)} \times (140 - \text{Age})}{72 \times \text{serum creatinine (mg/dL)}} \times 0.85$$

13. Female subjects must either be of non-reproductive potential (ie, post-menopausal by history: ≥60 years old and no menses for ≥1 year without an alternative medical cause; OR history of hysterectomy, OR history of bilateral tubal ligation, OR history of bilateral oophorectomy) or must have a negative serum pregnancy test upon study entry.
14. Subject is willing and able to comply with the protocol for the duration of the study including undergoing treatment and scheduled visits and examinations including follow up.

Exclusion Criteria:

Subjects who meet any of the following criteria will be excluded from this study:

1. Involvement in the planning and/or conduct of the study.
2. Participation in another clinical study with an investigational product during the last 4 weeks.
3. WHO Classification G3 neuroendocrine neoplasms of lung origin (oat cell/large cell lung cancer).
4. Prior treatment with anti-PDL-1/anti-PD-1 or anti-CTL-4 therapy.
5. Uncontrolled intercurrent illness including, but not limited to, ongoing or active infection, symptomatic congestive heart failure, uncontrolled hypertension, unstable angina pectoris, cardiac arrhythmia, active peptic ulcer disease or gastritis, active bleeding diathesis

including any subject known to have evidence of acute or chronic hepatitis B (e.g., HBsAg reactive), hepatitis C (e.g., HCV RNA [qualitative] is detected) or known history of Human Immunodeficiency Virus (HIV) (HIV 1/2 antibodies), or psychiatric illness/social situations that would limit compliance with study requirements or compromise the ability of the subject to give written informed consent.

6. Known history of previous clinical diagnosis of tuberculosis.
7. Current or prior use of immunosuppressive medication within 28 days before the first dose of durvalumab or tremelimumab, with the exceptions of intranasal and inhaled corticosteroids or systemic corticosteroids at physiological doses, which are not to exceed 10 mg/day of prednisone, or an equivalent corticosteroid.
8. Active or prior documented autoimmune disease within the past 2 years NOTE: Subjects with vitiligo, Grave's disease, or psoriasis not requiring systemic treatment (within the past 2 years) are not excluded.
9. Active or prior documented inflammatory bowel disease (e.g., Crohn's disease, ulcerative colitis).
10. History of allogeneic organ transplant.
11. History of hypersensitivity to durvalumab, tremelimumab or any excipient.
12. Subjects having a diagnosis of immunodeficiency or are receiving systemic steroid therapy or any other form of immunosuppressive therapy within 28 days prior to the first dose of trial treatment.
13. Knowledge of active central nervous system (CNS) metastases and/or carcinomatous meningitis. Subjects with previously treated brain metastases may participate provided they have stable brain metastases [without evidence of progression by imaging confirmed [by magnetic resonance imaging (MRI) if MRI was used at prior imaging, or confirmed by computed tomography (CT) imaging if CT used at prior imaging] for at least four weeks prior to the first dose of trial treatment; also, any neurologic symptoms must have returned to baseline], have no evidence of new or enlarging brain metastases,and have not used steroids for brain metastases for at least 7 days prior to trial treatment. This exception does not include carcinomatous meningitis, as subjects with carcinomatous meningitis are excluded regardless of clinical stability.
14. Receipt of live attenuated vaccination within 30 days prior to study entry or within 30 days of receiving durvalumab or tremelimumab. Note: The killed virus vaccines used for seasonal influenza vaccines for injection are allowed; however intranasal influenza vaccines (e.g., FluMist®) are live attenuated vaccines, and are not allowed.
15. Subjects having known history of, or any evidence of interstitial lung disease or active, noninfectious pneumonitis.
16. Any prior Grade ≥3 immune-related adverse event (irAE) while receiving any previous immunotherapy agent, or any unresolved irAE >Grade 1.
17. Subjects who have received any anti-cancer treatment within 21 days or any

investigational agent within 30 days prior to the first dose of study drug and should have recovered from any toxicity related to previous anti-cancer treatment. This does not apply to the use of somatostatin analogues for symptomatic therapy.

18. Major surgery within 3 weeks prior to the first dose of study drug.
19. Subjects having > 1+ proteinuria on urine dipstick testing will undergo 24h urine collection for quantitative assessment of proteinuria. Subjects with urine protein ≥ 1 g/24h will be ineligible.
20. Significant cardiovascular impairment: history of congestive heart failure greater than New York Heart Association (NYHA) Class II, unstable angina; myocardial infarction or stroke within 6 months of the first dose of study drug, or cardiac arrhythmia requiring medical treatment.
21. Mean QT interval corrected for heart rate (QTc) ≥470 ms calculated from 3 electrocardiograms (ECGs) using Fredericia's Correction.
22. Bleeding or thrombotic disorders or use of anticoagulants, such as warfarin, or similar agents requiring therapeutic international normalized ratio (INR) monitoring. Treatment with low molecular weight heparin (LMWH) is allowed.
23. Active hemoptysis (bright red blood of at least 0.5 teaspoon) within 3 weeks prior to the first dose of study drug.
24. Patients with tumoral disease in the head and neck region, such as paratracheal or periesophageal lymph node involvement, or with infiltration of structures in the digestive tract, or vascular pathways that represent a risk of increased bleeding.
25. Patients of cohort 1 with neuroendocrine tumors of pulmonary origin or pulmonary metastases with evidence of active bleeding.
26. Patients with evidence of digestive bleeding.
27. Active infection (any infection requiring treatment).
28. Active malignancy (except for differentiated thyroid carcinoma, or definitively treated melanoma in-situ, basal or squamous cell carcinoma of the skin, or carcinoma in-situ of the cervix) within the past 24 months.
29. Female patients who are pregnant or breastfeeding or male or female patients of reproductive potential who are not willing to employ highly effective birth control from screening to 180 days after the last dose of durvalumab + tremelimumab combination therapy or 90 days after the last dose of durvalumab monotherapy, whichever is the longer time period.
30. Documented active alcohol or drug abuse.
31. Patients with a prior history of non-compliance with medical regimens.
32. Any condition that, in the opinion of the investigator, would interfere with evaluation of study treatment or interpretation of patient safety or study results.

Investigational Product(s), Dose, and Mode of Administration:

Durvalumab, 1500 mg Q4W (equivalent to 20 mg/kg Q4W) for 12 months in patients ≥ 30kg.

Tremelimumab 75 mg Q4W (equivalent to 1 mg/kg Q4W) for up to 4 doses/cycles in patients ≥ 30kg.

Weight-based dosing should be used for patients <30 kg durvalumab 20 mg/kg Q4 and tremelimumab 1 mg/kg Q4.

Study Assessments and Criteria for Evaluation:

Safety Assessments:

The safety objective of this trial is to characterize the safety and tolerability of durvalumab plus tremelimumab in subjects with advanced neuroendocrine neoplasms. The primary safety analysis will be based on subjects who experienced toxicities as defined by CTCAE, v4.03 The attribution to drug, time-of-onset, duration of the event, its resolution, and any concomitant medications administered will be recorded. AEs will be analyzed including but not limited to all AEs, SAEs, fatal AEs, and laboratory changes. Furthermore, specific immune-related adverse events (irAEs) will be collected and designated as immune related events of clinical interest (ECIs).

Efficacy Assessments:

The primary efficacy objective of this study is to evaluate the anti-tumor activity of durvalumab plus tremelimumab in subjects with advanced neuroendocrine neoplasms. 9-months clinical benefit rate by Response Evaluation Criteria in Solid Tumors. (RECIST 1.1), which is defined as the percentage of patients achieving complete response, partial response (PR), or stable disease (SD) at month 9 after durvalumab plus tremelimumab was started for cohorts 1, 2 and 3, and 9-months overall survival rate, which is defined as the percentage of patients alive at 9 months after durvalumab plus tremelimumab was started will be used as the primaries endpoints per RECIST 1.1. **Pharmacodynamic / Pharmacokinetic Assessments (if applicable):**

Correlative blood samples for pharmacodynamics studies will be obtained during the course of the trial.

Archival tumor tissue will be required at baseline.

Statistical Methods and Data Analysis:

Strategic decisions about future development of immune therapy in neuroendocrine tumors will be available after the results of this trial.

Summary tables (descriptive statistics and frequency tables) will be provided for all baseline variables, efficacy variables, and safety variables, as appropriate. Continuous variables will be summarized with descriptive statistics (mean, standard deviation, range, and median). Ninety-five (95) percent confidence highest probability density (HPD) credible intervals (95% CI) may also be presented, as appropriate. Frequency counts and percentage of subjects within each category

will be provided for categorical data.

The primary efficacy analysis will be performed using the binomial test procedure. Missing data will be treated using statistical multiple random imputation.

Secondary endpoints will be summarized with descriptive statistics. Continuous variables will be summarized with n, mean, standard deviation, and range, frequency counts and percentage of subjects within each category will be provided for categorical data. Multivariate regression models will be used to study relations between explanatory variables and primary endpoint. Survival analysis will be performed to analyze PFS, Kaplan-Meier curves will be presented and possible comparisons will be tested using the log-rank test or the Cox proportional hazard model for multivariate analysis, hazard ratios (HR) and their 95% confidence interval (CI95%) will be provided. Patients with lost of follow-up or treatment discontinuation will be included in the final analysis of primary endpoint if they have at least one tumor evaluation and considered as censored data for survival endpoints.

R software version 3.2.1 will be used for all analysis.

Sample Size Determination:

Sample size has been calculated using one-sample Superiority test (function One Sample Proportion. (NIS) of the Trial Size package of R software). According to previous knowledge, it is estimated that the reference value for the likelihood to be progression-free at 9 months is 30% and we expect an increase of 20% with a superiority margin of 10%. With a unilateral alpha level of 5% and 80% power, we estimate to include 28 patients per group, with an expected lost to follow-up rate of 10%, a final sample size in each 1, 2 and 3 cohort will include 31 patients.

For cohort 4, and according to previous knowledge, it is estimated that the reference proportion of patients being alive at 9 months is 13% and we expect an increase of 10% with a superiority margin of 5%. With a unilateral alpha level of 5% and 80% power, we estimate to include 30 patients per group, with an expected lost to follow-up rate of 10%, a final sample size will include 33 patients.

Summarizing, total sample size will include 126 patients: 31 patients for cohorts 1 to 3, and 33 for cohort 4.

GETNE
Grupo Español de Tumores Neuroendocrinos y Endocrinos

SCHEDULE OF STUDY ASSESSMENTS (according to section 8)

Phase	Screening Phase		Treatment Phase								End of treatment [r]	Follow-up [s]
Period	Screening [,b]	Baseline[a,b]	Study Treatment									
Visit	V1 -28 to -2	V2 Day -3 to C1D1	V3 C1D1	V4 C1D15	V5 C2D1 (C1D28)	V6 C2D15	V7 C3D1 (C2D28)	V8 C3D15	V9 C4D1 (C3D28)	V10-V16 C5D1 C12D1		
Informed consent	X											
Inclusion/Exclusion	X	X										
Allocation		X										
Medical history and demographic Data	X											
ECOG PS[c]	X	X		X	X	X	X	X	X	X	X	X
Prior Therapies for neuroendocrine neoplasms	X											
Vitals signs[d]	X		X	X	X	X	X	X	X	X	X	X
Physical exam[f]	X	X[e]		X	X	X	X	X	X	X	X	X
12/lead ECG[g]	X		Only when clinically indicated									
Biochemistry and hematology[h]	X	X[h]		X	X	X	X	X	X	X	X	X
Hepatitis B and C; HIV	X											
Thyroid function test (TSH and fT3 and fT4)[i]	X			X	X	X	X	X	X	X	X	X
Urinalysis[j]	X			X	X	X	X	X	X	X	X	X
Pregnancy test[k]	X		Only when clinically indicated									
Durvalumab administration			X		X		X		X	X		
Tremelimumab administration			X		X		X		X			
Tumor assessments (CT/MRI)[l]	X		CT/MRI of chest/abdomen/pelvis and other areas of known disease at screening plus any areas of newly suspected disease should be performed every 12 weeks until confirmed PD.									
Octreoscan / PET/CT[m]	X											
Survival[n]					X		X		X	X	X	X
Biomarkers[o]		X			X						X (end of treatment/ progression)	
Archival tumor block or slides[p]		X										
Concomitant medication	X	X	X	X	X	X	X	X	X	X	X	X
AEs/SAEs[q]	X	X	X	X	X	X	X	X	X	X	X	X

AEs = adverse events, BP = blood pressure, CT = computerized tomography, ECG = electrocardiogram, ECOG = Eastern Cooperative Oncology Group, HR = heart rate, MRI = magnetic resonance imaging, PET = positron-emission tomography, RECIST 1.1 = Response Evaluation Criteria in Solid Tumors , RR = respiratory rate, SAEs = serious adverse events, surg = surgical, 99m-Tc MDP = 99m-technetium-methylene diphosphonate, w/in = within.

a) 72 hours before Cycle 1 Day 1 (C1D1). The **baseline assessment can be performed on C1D1, prior to treatment.** Informed consent may be taken up to 4 weeks prior to C1D1.

b) Efforts should be made to conduct study visits on the day scheduled (± 1 day). Clinical laboratory assessments may be conducted anytime within 72 hours prior to the scheduled visit.

c) ECOG will be performed at the Screening and Baseline Visits and at every subsequent treatment visit thereafter.

d) Assessments will include vital signs (supine BP, HR, RR, and body temperature), weight, and height. Height will be measured at the Screening Visit only. Elevated BP (≥140/90 mmHg) should be confirmed by 3 measurements (at least 5 minutes apart). If systolic BP is ≥160 mmHg or diastolic BP ≥100 mmHg, BP should be confirmed by repeat measurements after an hour. Subjects will have their blood pressure and pulse measured before, during, and after the infusion at the following times (based on a 60-minute infusion):

- At the beginning of the infusion (at 0 minutes)
- At 30 minutes during the infusion (±5 minutes)
- At the end of the infusion (at 60 minutes ±5 minutes)
- In the 1 hour observation period post-infusion: 30 and 60 minutes after the infusion (ie, 90 and 120 minutes from the start of the infusion) (±5 minutes) – for the first infusion only and then for subsequent infusions as clinically indicated

If the infusion takes longer than 60 minutes, then blood pressure and pulse measurements should follow the principles as described above or more frequently if clinically indicated. According to section 8.2.4.

e) Required if screening physical examination was performed > 7 days prior C1D1.

f) A comprehensive physical examination (including a neurological examination) will be performed at the Screening or Baseline Visit, on each visit of the Treatment phase, and at the off-treatment assessment. A symptom-directed physical examination will be performed on Cycle 1/Day 1 and at any time during the study, as clinically indicated.

g) Single 12-lead ECG. Subjects must be in the recumbent position for a period of 5 minutes prior to the ECG.

h) Assessments scheduled may be performed within 72 hours prior to the visit. Assessments during follow up are made every 8-12 weeks, according to investigator's criteria. According to section 8.2.5.

i) Free T3 and free T4 will only be measured if TSH is abnormal. They should also be measured if there is clinical suspicion of an adverse event related to the endocrine system. Assessments during follow up are made every 8-12 weeks, according to investigator's criteria.

j) Assessments during follow up are made every 8-12 weeks, according to investigator's criteria.

k) A serum or urine pregnancy test will be performed at the Screening Visit, at the Baseline Visit (or within 72 hours prior to the first dose of study medication)

l) Screening tumor assessments using CT of the chest/abdomen/pelvis and other areas of known disease or newly suspected disease should be performed within 28 days prior to C1D1. Scans of the abdomen, pelvis and other areas of the body may be done with MRI instead of CT, but evaluation of the chest should be done with CT. CT scans should be performed with oral and iodinated IV contrast and MRI scans with IV gadolinium chelate unless there is a medical contraindication to contrast. If iodinated IV contrast is contraindicated, chest CT should be done without IV contrast.

m) Somatostatin receptor scintigraphy (octreoscan) or PET-CT should be performed and/or available within the previous 6 months before C1D1, only on cohort 1-3. It is not applicable on cohort 4.

n) Survival data will be collected every 4 weeks until end of treatment phase is declared. All anticancer therapies will be collected. Survival data and other cancer treatments received will be collected every 6 months until close of the study database. The study sponsor may elect to discontinue survival follow-up.

o) Collection of blood sample to obtain plasma, serum, or other components to be used for biomarker studies. Samples will be obtained at baseline, Cycle 2/Day 1, and at end-of-treatment visit.

p) All subjects will have collection of most recent archived, tumor-biopsy sections for identification of predictive biomarkers.

q) Throughout the study from the signature of Informed Consent. SAE irrespective of relationship to study treatment must be reported as soon as possible but not later than one business day. AEs and concomitant meds collected 28 days from last dose.

r) See section 8.1.3, APPENDIX 2 and APPENDIX 3.

s) See section 8.1.3, APPENDIX 2 and APPENDIX 3.

LIST OF TABLES 19

LIST OF APPENDICES 20

ABBREVIATIONS AND DEFINITION OF TERMS 21

1. INTRODUCTION 24
1.1 Disease Background 24
1.1.1 Immunotherapies 26
1.1.2 Durvalumab 26
1.1.3 Tremelimumab 27
1.1.4 Durvalumab in combination with tremelimumab 28
1.2 Research hypothesis 28
1.3 Rationale for conducting this study 29
1.3.1 Durvalumab + tremelimumab combination therapy dose rationale 30
1.3.2 Rationale for 4 cycles of combination therapy followed by durvalumab monotherapy 31
1.4 Benefit/risk and ethical assessment 32
1.4.1 Potential benefits 32
1.4.1.1 Durvalumab 32
1.4.1.2 Tremelimumab 32
1.4.1.3 Durvalumab + tremelimumab 33
1.4.2 Potential risks 33
1.4.2.1 Durvalumab 33
1.4.2.2 Tremelimumab 34
1.4.2.3 Durvalumab + tremelimumab 35
1.4.2.4 Fixed Dosing for durvalumab and tremelimumab 36

2. STUDY OBJECTIVES 37
2.1 Primary objective(s) 37
2.2 Secondary objective(s) 37
2.3 Exploratory objective(s) 37

3. STUDY DESIGN 38
3.1 Overview of study design 38
3.2 Study schema 38
3.3 Study Oversight for Safety Evaluation 39

4. PATIENT SELECTION, ENROLLMENT, ALLOCATION, RESTRICTIONS, DISCONTINUATION AND WITHDRAWAL 40

4.1 Inclusion criteria 40
4.2 Exclusion criteria 42
4.3 Withdrawal of Subjects from Study Treatment and/or Study 44
4.4 Replacement of subjects 45

5. INVESTIGATIONAL PRODUCT(s) 45
5.1 Durvalumab and tremelimumab 45
5.1.1 Formulation/packaging/storage 45
5.2 Dose and treatment regimens 46
5.2.1 Treatment regimen 46
5.2.2 Duration of treatment and criteria for retreatment 46
5.2.3 Study drug preparation of durvalumab and tremelimumab 47
5.2.4 Monitoring of dose administration 49
5.2.5 Accountability and dispensation 50
5.2.6 Disposition of unused investigational study drug 50

6. TREATMENT PLAN 51
6.1 Subject enrollment and allocation 51
6.1.1 Procedures for allocation 51
6.1.2 Procedures for handling subjects incorrectly enrolled 51
6.2 Dose Modification and Toxicity Management 51
6.2.1 Durvalumab and tremelimumab 51

7. RESTRICTIONS DURING THE STUDY AND CONCOMITANT TREATMENT(s) 52
7.1 Restrictions during the study 52
7.2 Concomitant treatment(s) 53
7.2.1 Permitted concomitant medications 53
7.2.2 Excluded Concomitant Medications 54

8. STUDY PROCEDURES 55
8.1 Schedule of study procedures 55
8.1.1 Screening Phase 55
8.1.2 Treatment Phase 56
8.1.3 End of Treatment 59
8.2 Description of study procedures 59
8.2.1 Medical history and physical examination, electrocardiogram, weight and vital signs 59
8.2.2 Physical examination 59
8.2.3 Electrocardiograms 60

8.2.4 Vital signs 60
8.2.5 Clinical laboratory tests 61
8.3 Biological sampling procedures 62
8.3.1 Biomarker/Pharmacodynamic sampling and evaluation methods 62
8.3.2 Estimate of volume of blood to be collected 64
8.3.3 Archival tumor samples 64
8.3.3.1 Archival tumor samples 64
8.3.4 Withdrawal of informed consent for donated biological samples 64

9. DISEASE EVALUATION AND METHODS 65
Primary Objective 65
Secondary objectives (irRECIST) 65
9.1 Efficacy variable 66

10. ASSESSMENT OF SAFETY 66
10.1 Safety Parameters 66
10.1.1 Definition of adverse events 66
10.1.2 Definition of serious adverse events 67
10.1.3 Durvalumab + tremelimumab adverse events of special interest 67
10.2 Assessment of safety parameters 69
10.2.1 Assessment of severity 69
10.2.2 Assessment of relationship 70
10.3 Recording of adverse events and serious adverse events 70
10.3.1 Study recording period and follow-up for adverse events and serious adverse events 71
10.3.2 Reporting of serious adverse events 72
10.3.2.1 Reporting of deaths 72
10.3.3 Other events requiring reporting 73
10.3.3.1 Overdose 73
10.3.3.2 Hepatic function abnormality 73
10.3.3.3 Pregnancy 74

11. STATISTICAL METHODS AND SAMPLE SIZE DETERMINATION 75
11.1. Description of analysis sets 75
11.2. Methods of statistical analyses 76
11.3. Determination of sample size 76

12. ETHICAL AND REGULATORY REQUIREMENTS 77
12.1. Ethical conduct of the study 77

12.2. Ethics and regulatory review 77
12.3. Informed consent 77
12.4. Changes to the protocol and informed consent form 78
12.5. Audits and inspections 78
12.6. Confidentiality 78
12.7. Insurance 79
12.8. Publications 79
13.1. Training of study site personnel 80
13.2. Monitoring of the study 80
13.3. Source data 80
13.4. Retention of Records 81
13.5. Quality assurance 81
13.6. Study timetable and end of study 81

14. DATA MANAGEMENT 82
14.1. Study governance and oversight 82

15. INVESTIGATIONAL PRODUCT AND OTHER TREATMENTS 82
15.1. Identity of investigational product(s) 82

16. LIST OF REFERENCES 83
Appendix 1 Dosing Modification and Toxicity Management Guidelines for Immune-Mediated, Infusion-Related, and Non-Immune–Mediated Reactions (MEDI4736 Monotherapy or Combination Therapy With Tremelimumab or Tremelimumab Monotherapy). 91
Appendix 2 Schedule of study procedures follow-up for subjects who have completed durvalumab and tremelimumab treatment and achieved disease control (until confirmed progression of disease) and subjects who have discontinued durvalumab or tremelimumab due to toxicity in the absence of confirmed progression of disease. 115
Appendix 3 Schedule of study procedures: follow-up for subjects who have discontinued durvalumab or tremelimumab treatment due to confirmed progression of disease at the investigator discretion 116
Appendix 4 Durvalumab dose calculations 116
Appendix 5 Durvalumab Dose Volume Calculations 117
Appendix 6 Tremelimumab Dose Calculations 118
Appendix 7 Tremelimumab Dose Volume Calculations 119
Appendix 8 Contact Details 120

LIST OF TABLES

Table 1. Groups of neuroendocrine neoplasms following the WHO classification

Table 2. Effective methods of contraception (2 methods must be used)

Table 3. Prohibited and Rescue Medications

Table 4. Hematology Laboratory Tests

Table 5. Clinical chemistry (Serum or Plasma) Laboratory Tests

Table 6. Urinalysis Tests[a]

Tabla 7. List of investigational products for this study.

LIST OF APPENDICES

Appendix 1. Dosing Modification and Toxicity Management Guidelines for Immune-Mediated, Infusion-Related, and Non-Immune–Mediated Reactions

Appendix 2. Schedule of study procedures: follow-up for subjects who have completed durvalumab and tremelimumab treatment and achieved disease control (until confirmed progression of disease) and subjects who have discontinued durvalumab or tremelimumab due to toxicity in the absence of confirmed progression of disease.

Appendix 3. Schedule of study procedures: follow-up for subjects who have discontinued nivolumab or tremelimumab treatment due to confirmed progression of disease at the investigator discretion.

Appendix 4. Durvalumab DOSE CALCULATIONS

Appendix 5. Durvalumab VOLUME CALCULATIONS

Appendix 6. Tremelimumab DOSE CALCULATIONS

Appendix 7. Tremelimumab VOLUME CALCULATIONS

Appendix 8. Contact details.

Appendix 9. Sites and Investigators participants.

ABBREVIATIONS AND DEFINITION OF TERMS

The following abbreviations and special terms are used in this study Clinical Study Protocol.

Abbreviation	Explanation or special term
AchE	Acetylcholinesterase
ADA	Anti-drug antibody
AE	Adverse event
AESI	Adverse event of special interest
ALK	Anaplastic lymphoma kinase
ALT	Alanine aminotransferase
APF12	Proportion of patients alive and progression free at 12 months from randomization
AST	Aspartate aminotransferase
AUC	Area under the curve
AUC0-28day	Area under the plasma drug concentration-time curve from time zero to Day 28 post-dose
AUCss	Area under the plasma drug concentration-time curve at steady state
BoR	Best objective response
BP	Blood pressure
C	Cycle
CD	Cluster of differentiation
CI	Confidence interval
CL	Clearance
C_{max}	Maximum plasma concentration
$C_{max,ss}$	Maximum plasma concentration at steady state
CR	Complete response
CSA	Clinical study agreement
CSR	Clinical study report
CT	Computed tomography
CTCAE	Common Terminology Criteria for Adverse Event
CTLA-4	Cytotoxic T–lymphocyte-associated antigen 4
$C_{trough,ss}$	Trough concentration at steady state
CXCL	Chemokine (C-X-C motif) ligand
DoR	Duration of response
EC	Ethics Committee, synonymous to Institutional Review Board and Independent Ethics Committee
ECG	Electrocardiogram
ECOG	Eastern Cooperative Oncology Group
eCRF	Electronic case report form
EdoR	Expected duration of response
EGFR	Epidermal growth factor receptor
EU	European Union
FAS	Full analysis set
FDA	Food and Drug Administration

GCP	Good Clinical Practice
GI	Gastrointestinal
GMP	Good Manufacturing Practice
hCG	Human chorionic gonadotropin
HIV	Human immunodeficiency virus
HR	Hazard Ratio
IB	Investigator's Brochure
ICF	Informed consent form
ICH	International Conference on Harmonisation
IDMC	Independent Data Monitoring Committee
IFN	Interferon
IgE	Immunoglobulin E
IgG	Immunoglobulin G
IHC	Immunohistochemistry
IL	Interleukin
ILS	Interstitial lung disease
IM	Intramuscular
IMT	Immunomodulatory therapy
IP	Investigational product
irAE	Immune-related adverse event
IRB	Institutional Review Board
irRECIST	Immune-related Response Evaluation Criteria in Solid Tumors
ITT	Intent-to-Treat
IV	Intravenous
IVRS	Interactive Voice Response System
IWRS	Interactive Web Response System
mAb	Monoclonal antibody
MDSC	Myeloid-derived suppressor cell
MedDRA	Medical Dictionary for Regulatory Activities
MHLW	Minister of Health, Labor, and Welfare
miRNA	Micro-ribonucleic acid
MRI	Magnetic resonance imaging
NCI	National Cancer Institute
NE	Not evaluable
NSCLC	Non–small-cell lung cancer
OAE	Other significant adverse event
ORR	Objective response rate
OS	Overall survival
PBMC	Peripheral blood mononuclear cell
PD	Progressive disease
PD-1	Programmed cell death 1
PD-L1	Programmed cell death ligand 1
PD-L2	Programmed cell death ligand 2
PDx	Pharmacodynamic(s)

PFS	Progression-free survival
PFS2	Time to second progression
PGx	Pharmacogenetic research
PK	Pharmacokinetic(s)
PR	Partial response
q2w	Every 2 weeks
q3w	Every 3 weeks
q4w	Every 4 weeks
q6w	Every 6 weeks
q8w	Every 8 weeks
QTcF	QT interval corrected for heart rate using Fridericia's formula
RECIST 1.1	Response Evaluation Criteria in Solid Tumors, version 1.1
RNA	Ribonucleic acid
RR	Response rate
RT-QPCR	Reverse transcription quantitative polymerase chain reaction
SAE	Serious adverse event
SAP	Statistical analysis plan
SAS	Safety analysis set
SD	Stable disease
SNP	Single nucleotide polymorphism
SoC	Standard of Care
sPD-L1	Soluble programmed cell death ligand 1
T_3	Triiodothyronine
T_4	Thyroxine
TSH	Thyroid-stimulating hormone
ULN	Upper limit of normal
US	United States

1. INTRODUCTION

Neuroendocrine neoplasms constitute a heterogeneous group of tumors derived from the Kultchitzky cells of the diffuse neuroendocrine system located in the gastrointestinal tract (origin of classical carcinoid tumors), pancreatic islet cells (origin of pancreatic endocrine tumors) and lung (origin of typical and atypical carcinoids). Advanced disease is common at diagnosis leading to limited options of curative strategies, so general prognosis is poor. Neuroendocrine neoplasms are considered rare diseases, however, taken all together into account and the natural history of well-differentiated tumors, their prevalence is increasing. Systemic treatment options have been limited for several years. However, in the last decade, the better knowledge of molecular biology of neuroendocrine neoplasms has increased the interest to develop targeted agents in this field, changing the scenario of possible therapies for advanced disease. Even though, treatment options are still limited.

Immune balance has always been related with neuroendocrine neoplasms. Prolonged stabilizations have often been described without treatment and initial immune modulators, such as interferon, have demonstrated antitumoral effects in these tumors. Recent advances in immunotherapy have permitted the development of new targeted agents with higher efficacy and lower toxicity that opens a new treatment option for patients with advanced neuroendocrine tumors.

1.1 Disease Background

Well-differentiated gastroenteropancreatic and lung neuroendocrine tumors are generally malignancies with a prolonged natural history. However, clinical behavior is heterogeneous and when tumor progression is observed, treatment options are limited. Although advanced neuroendocrine neoplasms represent less than 2% of all gastrointestinal cancers, they constitute the second most prevalent advanced tumor of the gastrointestinal tract after colorectal cancer (Yao et al, 2008). The biological behavior of neuroendocrine neoplasms depends mainly on their histological characteristics, defined by the differentiation grade and Ki67 expression. The most recent 2010 WHO classification divides neuroendocrine neoplasms in three groups regarding Ki67 value and mitotic count: neuroendocrine tumors that include grade 1 (G1) and grade 2 (G2) with Ki67 value ≤20% and mitotic count ≤20 mitoses x 10 HPF, and neuroendocrine carcinomas that include grade 3 (G3) with Ki67>20% and/or mitotic count >20 mitoses x 10 HPF.

Table 1. Groups of neuroendocrine neoplasms following the WHO classification

	Lung and Thymus	GEP-NETs	
Grade	Mitotic count (10 HPF)	Mitotic count (10 HPF)	Ki-67 (%)
G1	< 2	<2	≤2
G2	2-10	2-20	3-20
G3	>10	>20	>20

The medical treatment of advanced well-moderately differentiated (G1/G2) neuroendocrine neoplasms has included somatostatin analogs, interferon and cytotoxic agents. The lack of effectiveness of conventional cytotoxic agents has prompted exploration of new targeted drugs exploiting phenotypical features of neuroendocrine neoplasms. Neuroendocrine neoplasms are characterized by being remarkably vascular and expressing several growth factors, including

vascular endothelial growth factor (VEGF), platelet-derived growth factor (PDGF), insulin-like growth factor 1 (IGF-1), basic fibroblast growth factor (bFGF), and transforming growth factor (TGF)-α and -β. In addition, expression of several receptors of these growth factors and ligands has been described, including stem cell factor receptor (c-KIT), epidermal growth factor receptors (EGFR), VEGF receptors (VEGFR)-2 and 3, IGF receptors (IGF-R), and PDGF receptors (PDGFR). The (over)expression of some of these factors has been associated with poor prognosis and decreased progression-free survival (PFS), as well as with tumor growth, aggressiveness, and disease extent in patients with gastroenteropancreatic neuroendocrine neoplasms. Several targeted agents have been studied in the treatment of neuroendocrine neoplasms including antiangiogenic compounds (such as bevacizumab), inhibitors of multiple receptors with kinase activity (such as sunitinib), and inhibitors of intracellular downstream effector proteins such as the mammalian target of rapamycin (mTOR) everolimus. Three phase III studies with everolimus and sunitinib have demonstrated efficacy in G1/G2 neuroendocrine tumors of the gastroenteropancreatic system becoming new treatment options in the management of these patients (Raymond et al 2011; Yao et al 2011; Yao et al 2015). Everolimus has also demonstrated efficacy in G1/G2 lung neuroendocrine tumors (typical and atypical carcinoids), becoming the first drug that has demonstrated a significant increase in progression-free survival in this setting (Yao et al 2015).

For advanced high-grade neuroendocrine carcinomas (G3), regardless of the site of the primary tumor, combination chemotherapy using cisplatin-based regimens is recommended early (provided that the patient has adequate organ function and performance status). Responses are frequently seen, however impact in overall survival is limited and life expectancy of these patients is around 12-16 months. There is no established second-line therapy for G3 neuroendocrine carcinomas of the gastroenteropancreatic system.

Immune responses directed against tumors are one of the body's natural defense against the growth and proliferation of cancer cells. However, over time and under pressure from immune attack, cancers develop strategies to evade immune-mediated killing allowing them to develop unchecked. One such mechanism involves upregulation of surface proteins that deliver inhibitory signals to cytotoxic T cells. Programmed cell death ligand 1 (PD-L1) is one such protein, and is upregulated in a broad range of cancers with a high frequency, with up to 88% expression in some tumor types. In a number of these cancers, including lung (Mu et al, 2011), renal (Krambeck et al, 2007), pancreatic (Nomi et al, 2007; Loos et al, 2008), ovarian cancer (Hamanishi et al, 2007), and hematologic malignancies (Andorsky et al, 2011; Brusa et al, 2013) tumor cell expression of PD-L1 is associated with reduced survival and an unfavorable prognosis.

Programmed cell death ligand 1 is part of a complex system of receptors and ligands that are involved in controlling T-cell activation. PD-L1 acts at multiple sites in the body to help regulate normal immune responses and is used by tumors to help evade detection and elimination by the host immune system tumor response. In the lymph nodes, PD-L1 on antigen-presenting cells binds to PD-1 or CD80 on activated T cells and delivers an inhibitory signal to the T cell (Keir et al, 2008; Park et al, 2010). This results in reduced T-cell activation and fewer activated T cells in circulation. In the tumor microenvironment, PD-L1 expressed on tumor cells binds to PD-1 and CD80 on activated T cells reaching the tumor. This delivers an inhibitory signal to those T cells, preventing them from killing target cancer cells and protecting the tumor from immune elimination (Zou and Chen, 2008).

Immune responses directed against tumors are one of the body's natural defenses against the growth and proliferation of cancer cells. T cells play a critical role in antitumor immunity and their infiltration and activity have been linked to improved prognosis in a number of cancers (Pagès et al, 2010; Suzuki et al, 2011). Immune evasion, primarily through suppression of T-cell activity, is now recognized as one of the hallmarks of cancer. Such evasion can occur via a range of mechanisms including production of suppressive cytokines such as IL-10, secretion of chemokines and growth factors that recruit and sustain suppressive regulatory T cells (Tregs) and inflammatory macrophages, and expression of inhibitory surface molecules such as B7-H1. Tumor types characterized as being responsive to immunotherapy-based approaches include melanoma, renal cell carcinoma (RCC), bladder cancer , and malignant mesothelioma (Bograd et al, 2011). Inhibition of CTLA-4 signaling is a validated approach to cancer therapy, as shown by the approval in 2011 of ipilimumab for the treatment of metastatic melanoma based on statistically significant and clinically meaningful improvement in OS (Robert et al, 2011).

In general, tumor response rates to anti-CTLA-4 therapy are low (~10%). However, in patients who respond, the responses are generally durable, lasting several months even in patients with aggressive tumors such as refractory metastatic melanoma. Because these agents work through activation of the immune system and not by directly targeting the tumor, responses can occur late and some patients may have perceived progression of their disease in advance of developing disease stabilization or a tumor response. In some cases, early growth of pre-existing lesions or the appearance of new lesions may have been due to immune-cell infiltration into the tumor and not due to proliferation and extension of neoplastic cells, per se (Wolchok et al, 2009). Overall, although the impact on conventionally-defined PFS can be small, durable response or stable disease seen in a proportion of patients can lead to significant prolongation of OS. The melanoma data with ipilimumab clearly demonstrate that a small proportion of patients with an objective response had significant prolongation of OS, supporting the development of this class of agents in other tumors. Although Phase 2 and Phase 3 studies of tremelimumab in metastatic melanoma did not meet the primary endpoints of response rate and OS, respectively, the data suggest activity of tremelimumab in melanoma (Ribas et al, 2013). In a large Phase 3 randomized study comparing tremelimumab with dacarbazine (DTIC)/temozolomide in patients with advanced melanoma, the reported median OS in the final analysis was 12.58 months for tremelimumab versus 10.71 months for DTIC/temozolomide (HR = 1.1416, p = 0.1272; Ribas et al, 2013).

1.1.1 Immunotherapies

It is increasingly understood that cancers are recognized by the immune system, and, under some circumstances, the immune system may control or even eliminate tumors (Dunn et al, 2004). Studies in mouse models of transplantable tumors have demonstrated that manipulation of co-stimulatory or co-inhibitory signals can amplify T-cell responses against tumors (Peggs et al 2009). This amplification may be accomplished by blocking co-inhibitory molecules, such as cytotoxic T-lymphocyte-associated antigen 4 (CTLA-4) or programmed cell death 1 (PD-1), from binding with their ligands, B7 or B7-H1 (programmed cell death ligand 1 [PD-L1]).

1.1.2 Durvalumab

The non-clinical and clinical experience is fully described in the last version of the durvalumab Investigator's Brochure .

Durvalumab is a human monoclonal antibody (mAb) of the immunoglobulin G (IgG) 1 kappa

subclass that inhibits binding of PD-L1 and is being developed by AstraZeneca/MedImmune for use in the treatment of cancer (MedImmune is a wholly owned subsidiary of AstraZeneca; AstraZeneca/MedImmune will be referred to as AstraZeneca throughout this document). As durvalumab is an engineered mAb, it does not induce antibody-dependent cellular cytotoxicity or complement-dependent cytotoxicity. The proposed mechanism of action for durvalumab is interference of the interaction of PD-L1.

PD-L1 is expressed in a broad range of cancers with a high frequency, up to 88% in some types of cancers. In a number of these cancers, including lung, the expression of PD-L1 is associated with reduced survival and an unfavorable prognosis. In lung cancer, only 12% of patients with tumors expressing PD-L1 survived for more than 3 years, compared with 20% of patients with tumors lacking PD-L1 (Mu et al, 2011). Based on these findings, an anti-PD-L1 antibody could be used therapeutically to enhance anti-tumor immune responses in patients with cancer. Results of several non-clinical studies using mouse tumor models support this hypothesis, where antibodies directed against PD-L1 or its receptor PD-1 showed anti-tumor activity (Hirano et al 2005, Iwai et al 2002, Okudaira et al 2009, Zhang et al 2008).

Durvalumab has been given to humans as part of ongoing studies as a single drug or in combination with other drugs.

As of the data cut-off date (12 July 2017) across the entire clinical development program, an estimated 4067 patients have been exposed to 1 or more doses of durvalumab in AstraZeneca-sponsored Phase I to III studies, either as monotherapy or in combination, and 5911 patients where the treatment arm is blinded. Additionally, approximately 4000 patients have been exposed to 1 or more doses of durvalumab in ESR/IITs.
Estimates of overall cumulative patient exposure based on actual exposure data from any completed clinical trials and the enrolment/randomisation schemes for ongoing open label and blinded trials are: 3723 patients received durvalumab monotherapy, 3372 patients received durvalumab in combination with tremelimumab.Details on the safety profile of durvalumab monotherapy, tremelimumab monotherapy and durvalumab plus tremelimumab are summarized in Section 1.4.2. Refer to the current durvalumab Investigator's Brochure for a complete summary of non-clinical and clinical information including safety, efficacy and pharmacokinetics.

1.1.3 Tremelimumab

The non-clinical and clinical experience is fully described in the last version of the tremelimumab Investigator's Brochure.

Tremelimumab is an IgG-2 kappa isotype mAb directed against the cytotoxic T-lymphocyte-associated protein 4 (CTLA-4) also known as CD152 (cluster of differentiation 152). This is an immunomodulatory therapy (IMT) that is being developed by AstraZeneca for use in the treatment of cancer.

Binding of CTLA-4 to its target ligands (B7-1 and B7-2) provides a negative regulatory signal, which limits T-cell activation. Anti-CTLA-4 inhibitors antagonize the binding of CTLA-4 to B7 ligands and enhance human T-cell activation as demonstrated by increased cytokine (interleukin [IL]-2 and interferon [IFN] gamma) production in vitro in whole blood or peripheral blood mononuclear cell (PBMC) cultures (Tarhini and Kikwood 2008). In addition, blockade of CTLA-4 binding to B7 by anti-CTLA-4 antibodies results in markedly enhanced T-cell activation and anti-tumor activity in animal models, including killing of established murine solid tumors and

induction of protective anti-tumor immunity. See the last version of tremelimumab IB, , for more information. Therefore, it is expected that treatment with an anti-CTLA-4 antibody, such as tremelimumab, will lead to increased activation of the human immune system, increasing anti-tumor activity in patients with solid tumors.

An extensive program of non-clinical and clinical studies has been conducted for tremelimumab both as monotherapy and combination therapy with conventional anticancer agents to support various cancer indications using different dose schedules.

To date Nov/2017 tremelimumab has been given to more than 1000 patients as part of ongoing studies either as monotherapy or in combination with other anticancer agents. Refer to the current tremelimumab Investigator's Brochure for a complete summary of non-clinical and clinical information including safety, efficacy and pharmacokinetics.

1.1.4 Durvalumab in combination with tremelimumab

Targeting both PD-1 and CTLA-4 pathways may have additive or synergistic activity (Pardoll 2012) because the mechanisms of action of CTLA-4 and PD-1 are non-redundant; therefore, AstraZeneca is also investigating the use of durvalumab + tremelimumab combination therapy for the treatment of cancer.

Durvalumab + tremelimumab: Study D4190C00006: As of 28 February 2017, durvalumab PK (n=347) and tremelimumab PK (n=353) data were available from the dose-escalation and dose-expansion phases following durvalumab Q4W (3, 10, 15, or 20 mg/kg) or Q2W (10 mg/kg) in combination with tremelimumab Q4W (1, 3, or 10 mg/kg). An approximately dose-proportional increase in PK exposure (C max and AUC 0 to 28 days [AUC 0-28]) of both durvalumab and tremelimumab was observed over the dose range of 3 to 20 mg/kg durvalumab Q4W and 1 to 10 mg/kg tremelimumab Q4W. Exposures following multiple doses demonstrated accumulation consistent with PK parameters estimated from the first dose. The observed PK exposures of durvalumab and tremelimumab following combination were consistent with respective monotherapy data, indicating no PK interaction between these 2 agents.

As of DCO of28 February 2017, ADA data were available from 99 patients for durvalumab and 95 patients for tremelimumab in Study D4190C00006. The ADA incidence was 2.0% (2/99 patients) for durvalumab and 5.3% (5/95 patients) for tremelimumab. There was no clear relationship between ADA and the dose of either durvalumab or tremelimumab, and no apparent association between ADA and safety or efficacy.

Durvalumab has also been combined with other anticancer agents, including gefitinib, dabrafenib, and trametinib. To date, no PK interaction has been observed between durvalumab and these agents.

1.2 Research hypothesis

The most used therapy for neuroendocrine tumors are somatostatin analogs, with clear evidence of antitumor activity demonstrated by two phase III clinical trials in gastroenteropancreatic neuroendocrine tumors. However, even the use in lung carcinoids is quite usual, no antitumoral activity has been demonstrated in prospective clinical trials. In recent years, target therapy as sunitinib and everolimus have been approved for pancreatic neuroendocrine tumors and recently everolimus have showed a significant reduction in the risk of progression or death in patients with

nonfunctional neuroendocrine tumors of lung or gastrointestinal origin. After the failure of these therapies, no other drugs have demonstrated efficacy. Only Interferon alpha-2b is an option for these patients who have worsening symptoms of carcinoid syndrome while on treatment with somatostatin analogs and have showed some evidence of antitumoral activity in neuroendocrine tumors. The efficacy of Interferon alpha 2-b is not fully understood, but one of its antitumoral mechanisms of action could be related via stimulation of T cells. Tremelimumab and Durvalumab are much more efficient drugs to improve immune system activation and could obtain a significantly higher clinical benefit in these patients. The presence of tumor-infiltrating lymphocytes (TILS) in these type of tumors have been described enhancing the hypothesis of immunotherapy in this setting.

Following the knowledge of increased tumor-specific antigens with the presence of more mutational load, dedifferentiation process and treatments previously received we hypothesize that immunotherapy could have a higher effect in the setting of refractory neuroendocrine neoplasms, when tumor behavior is more aggressive and the probability of immune escape process is higher.

1.3 Rationale for conducting this study

Well and moderately differentiated neuroendocrine tumors of the gastroenteropancreatic tract and lung are orphan tumors after progression a targeted therapy. For gastrointestinal origin tumors, somatostatin analogues have been the cornerstone of antihormonal therapy during decades. During the last 5 years, antiproliferative effects have also been demonstrated in this setting. Recently, for neuroendocrine tumors of the lung (typical and atypical carcinoids), everolimus has demonstrated impact in tumor growth control and improvement in progression-free survival. However, after progression to a targeted therapy, no other drugs are approved.

Within the lack of treatment options, combinations of somatostatin analogues and interferon alpha have widely been used, mainly for their effect in tumor hormone release and for the evidence of tumor growth control in many retrospective analyses.

The hypothesis of efficacy of interferon alpha in these settings could be via stimulation of T cells. Recently, PD-L1 expression has been observed in neuroendocrine tumors such as small cell neuroendocrine carcinomas of the lung and Merkel cell carcinoma. These facts could provide a timely opportunity to develop specific studies with targeted therapies that stimulate the immune system against the tumor. Currently, several clinical trials are ongoing in these G3 high grade neuroendocrine tumors of the lung (small cell lung cancer) and skin (Merkel Cell carcinomas). However, there are no clinical trials ongoing in G3 gastroenteropancreatic neuroendocrine neoplasms (which is the most frequent site of G3 neuroendocrine carcinomas after the lung origin) and well-moderately differentiated (G1/G2) neuroendocrine tumors, which are the ones where immunotherapy with interferon alpha has demonstrated some activity.

Tremelimumab and Durvalumab would be the first immune combination agents showing efficacy in G1/G2/G3 advanced neuroendocrine neoplasms of gastroenteropancreatic or lung origin.

As an antibody that blocks the interaction between PD-L1 and its receptors, durvalumab may relieve PD-L1-dependent immunosuppressive effects and, therefore, enhance the cytotoxic activity of anti-tumor T-cells. This hypothesis is supported by emerging clinical data from other mAbs targeting the PD-L1/PD-1 pathway, which provide early evidence of clinical activity and a manageable safety profile (Brahmer et al 2012, Topalian et al 2012). Responses have been

observed in patients with PD-L1-positive tumors and patients with PD-L1-negative tumors. In addition, durvalumab monotherapy has shown durable responses in NSCLC in Study 1108 (see Section 1.4.1.1).

The rationale for combining durvalumab and tremelimumab is that the mechanisms of CTLA-4 and PD-1 are non-redundant, suggesting that targeting both pathways may have additive or synergistic activity (Pardoll 2012). In fact, combining immunotherapy agents has been shown to result in improved response rates (RRs) relative to monotherapy. For example, the concurrent administration of nivolumab and ipilimumab to patients with advanced melanoma induced higher objective response rates (ORRs) than those obtained with single-agent therapy. Importantly, responses appeared to be deep and durable (Wolchok et al 2013). Similar results have been observed in an ongoing study of durvalumab + tremelimumab in NSCLC (Antonia et al 2014), with further updated details presented in this clinical study protocol.

1.3.1 Durvalumab + tremelimumab combination therapy dose rationale

The durvalumab + tremelimumab doses and regimen selected for this study are based on the goal of selecting an optimal combination dose of durvalumab and tremelimumab that would yield sustained target suppression (sPD-L1), demonstrate promising efficacy, and have an acceptable safety profile.

In order to reduce the dosing frequency of durvalumab to align with the q4w dosing of tremelimumab, while ensuring an acceptable PK/PDx, safety, and efficacy profile, cohorts were narrowed to 15 and 20 mg/kg durvalumab q4w. PK simulations from the durvalumab monotherapy data indicated that a similar area under the plasma drug concentration-time curve at steady state (AUC_{ss}; 4 weeks) was expected following both 10 mg/kg q2w and 20 mg/kg q4w durvalumab. The observed durvalumab PK data from the D4190C00006 study were well in line with the predicted monotherapy PK data developed preclinically. This demonstrates similar exposure of durvalumab 20 mg/kg q4w and 10 mg/kg q2w, with no alterations in PK when durvalumab and tremelimumab (doses ranging from 1 to 3 mg/kg) are dosed together. While the median Cmax at steady state ($C_{max,ss}$) is expected to be higher with 20 mg/kg q4w (approximately 1.5 fold) and median trough concentration at steady state ($C_{trough,ss}$) is expected to be higher with 10 mg/kg q2w (approximately 1.25 fold), this is not expected to impact the overall safety and efficacy profile, based on existing preclinical and clinical data.

Monotonic increases in PDx activity were observed with increasing doses of tremelimumab relative to the activity observed in patients treated with durvalumab monotherapy. There was evidence of augmented PDx activity relative to durvalumab monotherapy with combination doses containing 1 mg/kg tremelimumab, inclusive of both the 15 and 20 mg/kg durvalumab plus 1 mg/kg tremelimumab combinations.

Patients treated with doses of tremelimumab above 1 mg/kg had a higher rate of adverse events (AEs), including discontinuations due to AEs, serious AEs (SAEs), and severe AEs. Between the 10 mg/kg durvalumab + 1 mg/kg tremelimumab and 10 mg/kg durvalumab + 3 mg/kg tremelimumab cohorts treated at the q2w schedule, the number of patients reporting any AE, Grade 3 AEs, SAEs, and treatment-related AEs was higher in the 10 mg/kg durvalumab + 3 mg/kg tremelimumab cohort than the 10 mg/kg durvalumab + 1 mg/kg tremelimumab cohort. A similar pattern was noted in the q4w regimens, suggesting that, as the dose of tremelimumab increased above 1 mg/kg, a higher rate of treatment-related events may be anticipated. Further, the SAEs

frequently attributed to immunotherapy, pneumonitis and colitis, were more commonly seen in cohorts using either 3 or 10 mg/kg of tremelimumab compared to the 1-mg/kg dose cohorts. Together, these data suggest that a combination using a tremelimumab dose of 1 mg/kg appeared to minimize the rate of toxicity when combined with durvalumab. As a result, all combination doses utilizing either the 3 or 10 mg/kg doses of tremelimumab were eliminated in the final dose selection.

In contrast, cohorts assessing higher doses of durvalumab with a constant dose of tremelimumab did not show an increase in the rate of AEs. The data suggested that increasing doses of durvalumab may not impact the safety of the combination as much as the tremelimumab dose. Further, safety data between the 10-mg/kg and 20-mg/kg cohorts were similar, with no change in safety events with increasing dose of durvalumab.

In Study D4190C00006, of all treatment cohorts, the cohort of 11 patients treated in the 20 mg/kg durvalumab + 1 mg/kg tremelimumab group had the fewest AEs, Grade ≥3 AEs, SAEs, and treatment discontinuations due to AEs, but still showed strong evidence of clinical activity. This cohort had a lower number of treatment-related Grade ≥3 AEs or treatment-related SAEs. No dose-limiting toxicities were reported.

Preliminary clinical activity of the durvalumab and tremelimumab combination did not appear to change with increasing doses of tremelimumab. The 15- and 20-mg/kg durvalumab q4w cohorts demonstrated objective responses at all doses of tremelimumab, and increasing doses of tremelimumab did not provide deeper or more rapid responses.

Efficacy data suggested that the 20 mg/kg durvalumab + 1 mg/kg tremelimumab dose cohort may demonstrate equivalent clinical activity to another dose combinations. Of the 45 patients, full analysis set,, there were 7 patients (15,6%) with PR, 19 patients (42,2%) with SD, and 15 patients (33,3%) with PD. Four patients were not evaluable for response .

Additionally, of all cohorts, the 20 mg/kg durvalumab + 1 mg/kg tremelimumab dose cohort had the fewest AEs, Grade ≥3 AEs, SAEs, and treatment discontinuations due to AEs, but still showed some evidence of clinical activity. All together, the data suggested that a 20 mg/kg durvalumab + 1 mg/kg tremelimumab dose combination should be selected for further development.

Refer to the current durvalumab Investigator’s Brochure for a complete summary of non-clinical and clinical information on the durvalumab + tremelimumab combination, including safety, efficacy and pharmacokinetics.

1.3.2 Rationale for 4 cycles of combination therapy followed by durvalumab monotherapy

Long-term follow up on melanoma patients treated with ipilimumab, an anti-CTLA-4 targeting antibody (dosed every 3 weeks [q3w] for 4 doses and then discontinued), shows that patients responding to ipilimumab derive long-term benefit, with a 3-year OS rate of approximately 22%. Furthermore, the survival curve in this population reached a plateau at 3 years and was maintained through 10 years of follow up (Schadendorf et al, 2013).

Similar data have been presented for other anti-PD-1/PD-L1 targeting antibodies:

Nivolumab (anti-PD-1) was dosed q2w for up to 96 weeks in a large Phase I dose-escalation and expansion study, and showed responses were maintained for a median of 22.94 months for

melanoma (doses 0.1 mg/kg to 10 mg/kg), 17 months for NSCLC (doses 1, 3, and 10 mg/kg), and 12.9 months for renal cell carcinoma patients (doses 1 and 10 mg/kg) at the time of data analysis (Hodi et al 2014, Bramer et al 2014, Drake et al 2013). Furthermore, responses were maintained beyond treatment discontinuation in the majority of patients who stopped nivolumab treatment (either due to protocol specified end of treatment, complete response [CR], or toxicity) for up to 56 weeks at the time of data analysis (Topalian et al 2014).

MPDL3280a (anti-PD-L1) and the combination of nivolumab with ipilimumab, in which patients were dosed for a finite time period and responses maintained beyond treatment discontinuation have been reported (Herbest et al 2013, Wolchok et al 2013).

Similar long term results may be expected with use of other immune-mediated cancer therapeutics including anti-CTLA-4 antibodies such as tremelimumab, anti PD-L1 antibodies such as durvalumab, or the combination of the two.

1.4 Benefit/risk and ethical assessment

Natural history of neuroendocrine tumors is usually prolonged. G1/G2 neoplasms are slowing growing tumors that require continued therapy to control tumor growth, increasing the risk of long-term side effects. The prolonged effect of immunotherapy could be of especial interest in this setting, reducing the risk of chronic toxicity and maintaining the long-term benefit.

1.4.1 Potential benefits

1.4.1.1 Durvalumab

The majority of the safety and efficacy data currently available for durvalumab are based on the first time in-human, single-agent study (Study 1108) in patients with advanced solid tumors. Data from Study 1108 were presented at the European Society for Medical Oncology 2014 Congress. Overall, 456 of 694 subjects treated with durvalumab 10 mg/kg Q2W were evaluable for response (defined as having ≥ 24 weeks follow-up, measurable disease at baseline, and ≥ 1 follow-up scan, or discontinued due to disease progression or death without any follow-up scan). In PD-L1 unselected patients, the objective response rate (ORR), based on investigator assessment per Response Evaluation Criteria in Solid Tumors (RECIST)v1.1, ranged from 0% in uveal melanoma (n = 23) to 20.0% in bladder cancer (n = 15), and disease control rate at 24 weeks (DCR-24w) ranged from 4.2% in triple-negative breast cancer (TNBC; n = 24) to 39.1% in advanced cutaneous melanoma (n = 23). PD-L1 status was known for 383 of the 456 response evaluable subjects. Across the PD-L1-positive tumors, ORR was highest for bladder cancer, advanced cutaneous melanoma, hepatocellular carcinoma (HCC; n = 3 each, 33.3% each), NSCLC (n = 86, 26.7%), and squamous cell carcinoma of the head and neck (SCCHN; n = 22, 18.2%). In the PD-L1-positive subset, DCR-24w was highest in advanced cutaneous melanoma (n = 3, 66.7%), NSCLC (n = 86, 36.0%), HCC and bladder cancer (n = 3 each, 33.3% each), and SCCHN (n = 22, 18.2%)(Antonia et al 2014b).

1.4.1.2 Tremelimumab

In a single-arm, Phase II study (Study A3671008) of tremelimumab administered at 15 mg/kg every 90 days to patients with refractory melanoma, an RR of 7% and a median OS of 10 months in the second-line setting (as compared to approximately 6 months with best supportive care reported from a retrospective analysis; Korn et al 2008) were observed (Kirkwood et al 2010). In a

randomized, open-label, first-line Phase III study of tremelimumab (administered at 15 mg/kg every 90 days) versus chemotherapy (dacarbazine or temozolomide) in advanced melanoma (Study A3671009), results of the final analysis showed an RR of 11% and a median OS of 12.58 months in this first-line setting as compared to 10.71 months with standard chemotherapy; however, these results were not statistically significant (Ribas et al 2013). Additionally, a Phase II maintenance study (Study A3671015) in patients with Stage IIIB or IV NSCLC who had responded or remained stable failed to achieve statistical significance. The primary endpoint of PFS at 3 months was 22.7% in the tremelimumab arm (15 mg/kg) compared with 11.9% in the best supportive care arm (Study A3671015).

1.4.1.3 Durvalumab + tremelimumab

The preclinical and clinical justification for this combination as noted in Section 1.1.4 also supports the synergy of this combination. Available data, such as those presented by Wolchok et al, suggest that the combination of agents targeting PD-1/PD-L1 and CTLA-4 may have profound and durable benefits in patients with melanoma (Wolchok et al 2013). Of the 102 subjects with advanced NSCLC treated with durvalumab in combination with tremelimumab in Study D4190C00006, 63 subjects with at least 16 weeks of follow-up were evaluable for response (defined as measurable disease at baseline and at least 1 follow-up scan; this included discontinuations due to disease progression or death without follow-up scan). Of the 63 evaluable subjects, 17 (27%) had a best overall response of PR,14 (22%) had SD,22 (35%) had PD, and 10 (16%) were not evaluable. The ORR(confirmed and unconfirmed CR or PR) was 27% and the DCR (CR, PR, or SD) was 49% as assessed by RECIST v1.1.

Current experience with single-agent IMT studies suggests that clinical responses may be restricted to a subset of any given patient population and that it might be beneficial to enrich the patient population by selecting patients likely to respond to therapy. To date, no assay has been established or validated, and no single approach has proven accurate, for patient enrichment for IMTs. However, independent data from multiple sources using different assays and scoring methods suggests that PD-L1 expression on tumor cells and/or tumor infiltrating cells may be associated with greater clinical benefit.

Data from ongoing studies with durvalumab and other agents targeting the PD-1/PD-L1 pathway suggest, as shown in a number of tumor types (eg, NSCLC, renal cell carcinoma, and melanoma), that monotherapy may be more efficacious (in terms of ORR) in patients who are PD-L1-positive.

Given these findings, a number of ongoing studies are assessing the activity of agents in patients with PD-L1–positive tumors. There is also an unmet medical need in patients with PD-L1–negative tumors that needs to be addressed. Data, as of 27 January 2015 from Study 006 show that with the addition of tremelimumab to durvalumab, the ORR can be increased to 25% in patients with PD-L1 negative NSCLC.

1.4.2 Potential risks

1.4.2.1 Durvalumab

Risks with durvalumab include cough/productive cough, pneumonitis, ILD, dysphonia, ALT/AST increased, hepatitis, diarrhoea, abdominal pain, colitis, hypothyroidism, hyperthyroidism, blood TSH increased, blood TSH decreased, adrenal insufficiency, Type 1 diabetes mellitus,

hypophysitis/hypopituitarism, diabetes insipidus, blood creatinine increased, dysuria, nephritis, rash, pruritus, night sweats, dermatitis, myocarditis, pyrexia, oedema peripheral, upper respiratory tract infections, pneumonia, oral candidiasis, dental and oral soft tissue infections, influenza, myalgia, myositis, polymyositis and infusion related reaction.

ImAEs: immune-mediated pneumonitis, hepatitis, colitis, hypothyroidism, hyperthyroidism, adrenal insufficiency, Type 1 diabetes mellitus, hypophysitis/hypopituitarism, nephritis, rash and pemphigoid. Other immune-mediated adverse reactions including myocarditis and myositis/polymyositis. These imAEs should be managed based on dosing and Toxicity Management Guidelines described in appendix 1 of this protocol.

Infusion-related reactions: as with the administration of any Ig, infusion-related reactions may occur and may be severe and should be managed according to Toxicity Management Guidelines and study protocol.

As patients with previous history of bleeding and/or who are taking anticoagulant medications may have a higher risk of subsequent bleeding, the investigator should devote special attention to monitoring of these patients in the context of possible bleeding.

Further information on these risks can be found in the current version of the durvalumab IB.

In monotherapy clinical studies AEs (all grades) reported very commonly (≥10% of patients) are fatigue, cough , decreased appetite , dyspnoea , nausea , constipation , diarrhoea , pyrexia , back pain, anaemia, vomiting, pruritus, arthralgia, headache, asthenia, oedema peripheral and rash.

A total of 178 patients (9.4%) discontinued from study treatment due to an AE. A total of 108 patients (5.7%) had serious AEs (SAEs) that were considered by the investigator as related to durvalumab.

Most treatment-related AEs were manageable by delaying or interrupting the durvalumab dose, symptomatic treatment, and in the case of events suspected to have an immune basis, the use of established treatment guidelines for immune-mediated toxicity (see the Dosing Modification and Toxicity Management Guidelines).

Serious adverse reactions for durvalumab monotherapy considered expected

MedDRA (v19.1) SOC	PT	Number (%) of subjects exposed (N=1889)		
		Suspected SARs n (%) [a]	Occurrence of life-threatening suspected SARs n (%) [b]	Occurrence of fatal suspected SARs n (%) [b]
Respiratory, thoracic and mediastinal disorders	Pneumonitis	23 (1.2)	0	0
Injury, poisoning and procedural complications	Infusion related reaction	5 (0.3)	0	0
Infections and infestations	Pneumonia	4 (0.2)	0	0
	Lung infection	2 (0.1)	0	0
Gastrointestinal disorders	Colitis	4 (0.2)	0	0
	Diarrhoea	4 (0.2)	0	0
	Abdominal pain	2 (0.1)	0	0
Endocrine disorders	Adrenal insufficiency	2 (0.1)	0	0
Hepatobiliary disorders	Autoimmune hepatitis	2 (0.1)	0	0
Investigations	AST increased	2 (0.1)	0	0

Pooled dataset from studies CD-ON-MEDI4736-1108 (DCO 16 October 2017), ATLANTIC (DCO 3 June 2016) and PACIFIC (DCO 22 March 2018).

[a] n = number of subjects who have experienced the SAR.

[b] All fatal and all life-threatening events for durvalumab monotherapy are considered unexpected for reporting purposes.

AST aspartate aminotransferase; DCO data cut-off; MedDRA Medical Dictionary for Regulatory Activities; PT preferred term; SAR serious adverse reaction; SOC system organ class.

A detailed summary of durvalumab monotherapy AE data can be found in the current version of the durvalumab IB.

1.4.2.2 Tremelimumab

Potential risks, based on the mechanism of action of tremelimumab and related molecules (ipilimumab) include potentially immune-mediated gastrointestinal (GI) events including enterocolitis,, abdominal pain, dehydration, nausea and vomiting, and decreased appetite (anorexia); dermatitis including urticaria, skin exfoliation, and dry skin; endocrinopathies including hypophysitis, adrenal insufficiency, and hyperthyroidism and hypothyroidism; pancreatitis including autoimmune pancreatitis and lipase and amylase elevation; respiratory tract events including pneumonitis and interstitial lung disease (ILD); nervous system events including encephalitis, peripheral motor and sensory neuropathies, and Guillain-Barré syndrome; cytopenias including thrombocytopenia, anemia, and neutropenia; infusion-related reactions; anaphylaxis; and serious allergic reactions. The profile of AEs and the spectrum of event severity have remained stable across the tremelimumab clinical program and are consistent with the pharmacology of the target. To date, no tumor type or stage appears to be associated with unique AEs (except for vitiligo that appears to be confined to patients with melanoma). Overall, 944 of the 973 patients (97.0%) treated with tremelimumab monotherapy as of the data cutoff date of 1 November 2015 (not including 569 patients who have been treated in the ongoing blinded Phase IIb Study D4880C00003) experienced at least 1 AE. The events resulted in discontinuation of tremelimumab in 10.0% of patients, were serious in 36.5%, were Grade ≥3 in severity in 49.8%, were fatal in 67.7%, and were considered to be treatment related in 79.1% of patients. The frequency of any AEs and Grade ≥3 AEs was generally similar across the tremelimumab dose groups. However, a higher percentage of patients in the 10 mg/kg every 28 days and 15 mg/kg every 90 days groups compared with the All Doses <10 mg/kg group experienced treatment-related AEs, SAEs, AEs resulting in discontinuation of investigational product (IP), and deaths.

Serious adverse reactions for the tremelimumab monotherapy pool considered expected

MedDRA (v22.0) SOC	PT	Number (%) of subjects exposed Tremelimumab RSI pool (N=1665)		
		Suspected SARs [a] n (%)	Occurrence of life-threatening suspected SARs [b] n (%)	Occurrence of fatal suspected SARs [b] n (%)
Endocrine disorders	Hypophysitis	13 (0.8)	0	0
	Adrenal insufficiency	7 (0.4)	0	0
	Hyperthyroidism	5 (0.3)	0	0
	Hypothyroidism	4 (0.2)	0	0
	Lipase increased	2 (0.1)	0	0
Gastrointestinal disorders	Diarrhoea	183 (11.0)	0	0
	Colitis	68 (4.1)	0	0
	Enterocolitis	4 (0.2)	0	0
Hepatobiliary disorders	Autoimmune hepatitis	2 (0.1)	0	0
Skin and subcutaneous tissue disorders	Rash	8 (0.5)	0	0
	Pruritus	2 (0.1)	0	0

Includes legacy studies (A3671002, A3671008, A3671009, A3671022, A3671001, A3671011, A3671014, A3671015) and D4880C00003, D4880C00010, D4881C00024, D4884C00001, D4193C00003, D4190C00022 and D4191C00004. n = Number of subjects who have experienced the SAR.

a n = number of subjects who have experienced the SAR.

b All fatal and all life-threatening events for tremelimumab monotherapy are considered unexpected for reporting purposes.

ADR Adverse drug reaction; DCO Data cut-off; MedDRA Medical Dictionary for Regulatory Activities; PT Preferred term; RSI Reference safety information; SAR Serious adverse reaction; SOC System organ class.

A detailed summary of tremelimumab monotherapy AE data can be found in the current version of the tremelimumab IB.

1.4.2.3 Durvalumab + tremelimumab

Safety data have been pooled for 5 durvalumab and tremelimumab combination studies (D4190C00002, D4190C00006, D4190C00010, D4190C00011 and D4193C00003 [CONDOR]) for patients who received a dose of 20 mg/kg Q4W durvalumab plus 1 mg/kg tremelimumab Q4W, or equivalent durvalumab 10 mg/kg Q2W + tremelimumab 1 mg/kg Q4W; a total of 1088 patients are included in the pooled data set with a DCO of the 12 July 2017.
Overall, AEs reported in ≥10% of patients were fatigue, diarrhoea, nausea, decreased appetite, dyspnoea, pruritus, anaemia, constipation, pyrexia, vomiting, cough, back pain, abdominal pain, rash, arthralgia, hypothyroidism, asthenia, hyponatraemia, weight decreased, and oedema peripheral.

A total of 166 patients (15.3%) had SAEs that were considered treatment-related by the investigators. A total of 165 patients (15.2%) discontinued from study treatment due to an AE.

- Potential imAEs for durvalumab and durvalumab in combination with tremelimumab include:
    - Pancreatitis
    - Other rare or less frequent events with a potential immune-mediated aetiology, eg,

GETNE
Grupo Español de Tumores Neuroendocrinos y Endocrinos

pericarditis, sarcoidosis, uveitis, and other events involving the eye (eg, keratitis and optic neuritis), skin (eg, scleroderma and vitiligo), and haematological (eg, haemolytic anaemia and immune thrombocytopenic purpura), rheumatological events (polymyalgia rheumatic and autoimmune arthritis) and neuropathy/neuromuscular toxicities (eg, myasthenia gravis, Guillain Barre syndrome).

- Hypersensitivity reactions including
  - Anaphylaxis and allergic reaction
  - Cytokine release syndrome
  - Immune complex disease
- Other infections

Infusion-related reactions: as with the administration of any Ig, infusion-related reactions may occur and may be severe and should be managed according to Toxicity Management Guidelines and study protocol.

Serious adverse reactions for durvalumab plus tremelimumab combination considered expected.

MedDRA (v22.0) SOC	PT	Number (%) of subjects exposed (N=1278)		
		Suspected SARs n (%) [a]	Occurrence of life-threatening suspected SARs n (%) [b]	Occurrence of fatal suspected SARs n (%) [b]
Gastrointestinal disorders	Colitis	33 (2.6)	0	0
	Diarrhoea	31 (2.4)	0	0
	Lipase increased	4 (0.3)	0	0
	Autoimmune colitis	4 (0.3)	0	0
	Abdominal pain	3 (0.2)	0	0
	Enterocolitis	3 (0.2)	0	0
	Amylase increased	2 (0.2)	0	0
Respiratory, thoracic and mediastinal disorders	Pneumonitis	14 (1.1)	0	0
	Interstitial lung disease	4 (0.3)	0	0
Endocrine disorders	Adrenal insufficiency	10 (0.8)	0	0
	Hyperthyroidism	4 (0.3)	0	0
	Hypopituitarism	4 (0.3)	0	0
	Hypophysitis	2 (0.2)	0	0
General disorders and administration site conditions	Pyrexia	4 (0.3)	0	0
Hepatobiliary disorders	AST increased	4 (0.3)	0	0
	ALT increased	2 (0.2)	0	0
	Autoimmune hepatitis	2 (0.2)	0	0
	Hepatitis	2 (0.2)	0	0
Infections and infestations	Pneumonia	3 (0.2)	0	0
Cardiac disorders	Myocarditis	2 (0.2)	0	0
Injury, poisoning and procedural complications	Infusion related reaction	2 (0.2)	0	0

Pooled dataset from studies D4190C00002, D4190C00006, D4190C00010, D4190C00011, D4190C00021, D4190C00022 and CONDOR.
[a] n = number of subjects who have experienced the SAR.
[b] All fatal and all life-threatening events for durvalumab + tremelimumab are considered unexpected for reporting purposes.
ALT alanine aminotransferase; AST aspartate aminotransferase; DCO data cut-off; MedDRA Medical Dictionary for Regulatory Activities; PT preferred term; SAR serious adverse reaction; SOC system organ class.

A detailed summary of durvalumab + tremelimumab combination AE data can be found in the current version of the durvalumab IB.

1.4.2.4 Fixed Dosing for durvalumab and tremelimumab

A population PK model was developed for durvalumab using monotherapy data from a Phase 1 study (*study 1108; N=292; doses= 0.1 to 10 mg/kg Q2W or 15 mg/kg Q3W; solid tumors*). Population PK analysis indicated only minor impact of body weight (WT) on PK of durvalumab (coefficient of ≤ 0.5). The impact of body WT-based (10 mg/kg Q2W) and fixed dosing (750 mg Q2W) of durvalumab was evaluated by comparing predicted steady state PK concentrations (5th, median and 95th percentiles) using the population PK model. A fixed dose of 750 mg was selected to approximate 10 mg/kg (based on median body WT of ~75 kg). A total of 1000 patients were simulated using body WT distribution of 40–120 kg. Simulation results demonstrate that body WT-based and fixed dosing regimens yield similar median steady state PK concentrations with slightly less overall between-subject variability with fixed dosing regimen.

Similarly, a population PK model was developed for tremelimumab using data from Phase 1 through Phase 3 (*N=654; doses= 0.01 to 15 mg/kg Q4W or Q90D; metastatic melanoma*) [Wang et al. 2014]. Population PK model indicated minor impact of body WT on PK of tremelimumab (coefficient of ≤ 0.5). The WT-based (1 mg/kg Q4W) and fixed dosing (75 mg/kg Q4W; based on median body WT of ~75 kg) regimens were compared using predicted PK concentrations (5th, median and 95th percentiles) using population PK model in a simulated population of 1000 patients with body weight distribution of 40 to 120 kg. Similar to durvalumab, simulations indicated that both body WT-based and fixed dosing regimens of tremelimumab yield similar median steady state PK concentrations with slightly less between-subject variability with fixed dosing regimen.

Similar findings have been reported by others [Ng et al 2006, Wang et al 2009, Zhang et al 2012, Narwal et al 2013]. Wang and colleagues investigated 12 monoclonal antibodies and found that fixed and body size-based dosing perform similarly, with fixed dosing being better for 7 of 12 antibodies. In addition, they investigated 18 therapeutic proteins and peptides and showed that fixed dosing performed better for 12 of 18 in terms of reducing the between-subject variability in pharmacokinetic/pharmacodynamics parameters [Zhang et al 2012].

A fixed dosing approach is preferred by the prescribing community due to ease of use and reduced dosing errors. Given expectation of similar pharmacokinetic exposure and variability, we considered it feasible to switch to fixed dosing regimens. Based on average body WT of 75 kg, a fixed dose of 750 mg Q2W MEDI4736 (equivalent to 10 mg/kg Q2W), 1500 mg Q4W durvalumab (equivalent to 20 mg/kg Q4W) and 75 mg Q4W tremelimumab (equivalent to 1 mg/kg Q4W) is included in the current study.

Fixed dosing of durvalumab and tremelimumab is recommended only for subjects with more or equal than 30kg body weight due to endotoxin exposure. Patients with a body weight less than 30 kg should be dosed using a weight-based dosing schedule.

2. STUDY OBJECTIVES

2.1 Primary objective(s)

The study will include patients with advanced neuroendocrine neoplasms in four different cohorts with the following primary endpoints:

Primary endpoint for cohorts 1, 2 and 3:

Nine-months clinical benefit rate (CBR) by Response Evaluation Criteria In Solid Tumors (RECIST 1.1), which is defined as the percentage of patients achieving complete response (CR), partial response (PR), or stable disease (SD) at month 9 after durvalumab plus tremelimumab was started.

Primary endpoint for cohort 4:

Nine-months overall survival rate, which is defined as the percentage of patients alive at month 9 after durvalumab plus tremelimumab was started.

2.2 Secondary objective(s)

- Overall response rate (ORR) by irRECIST.
- To assess the duration of response according to irRECIST.
- To assess the median progression-free survival time (PFS) according to irRECIST.
- To assess the safety profile of Durvalumab and Tremelimumab in subjects with advanced neuroendocrine neoplasms.
- To assess the median overall survival (OS) time.
- To assess response status according to irRECIST at 6 and 12 months after start of study treatment.

2.3 Exploratory objective(s)

- To evaluate biochemical response (changes in CgA and NSE levels) and its association with response rate and progression-free survival.
- To assess whether baseline tumor and blood biomarkers may be predictive of response to durvalumab and tremelimumab therapy.
- To explore additional hypotheses related to biomarkers and relationship to durvalumab and tremelimumab efficacy and/or toxicity and neuroendocrine tumors evolution that may arise from internal or external research activities.

3. STUDY DESIGN

3.1 Overview of study design

This is a prospective, multi-center, open label, stratified, exploratory phase II study evaluating the

efficacy and safety of durvalumab plus tremelimumab in different cohorts of patients with neuroendocrine neoplasms.

The study will include patients in four different cohorts:

- **Cohort 1**: Well-moderately differentiated lung neuroendocrine tumors (classically known as typical and atypical carcinoids) after progression to somatostatin analogs and one prior targeted therapy or chemotherapy.
- **Cohort 2**: G1/G2 (WHO grade 1 and 2) gastrointestinal neuroendocrine tumors after progression to somatostatin analogs and one prior targeted therapy.
- **Cohort 3**: G1/G2 (WHO grade 1 and 2) pancreatic neuroendocrine tumors after progression to standard therapies (chemotherapy, somatostatin analogs and target therapy), who have received between two and four prior lines of treatment.
- **Cohort 4**: Neuroendocrine neoplasms (WHO grade 3) of gastroenteropancreatic origin or unknown primary site (excluding lung primary tumors), patients will be treated in second line only, after progression to first-line chemotherapy with a platinum based regimen.

The patient recruitment period of the study will last approximately 24 months. The maximum treatment period for each subject on study is anticipated to be approximately 12 months (13 administrations of durvalumab). However, subjects will continue on active follow-up to determine secondary endpoints of duration of response, median progression-free survival, median overall survival and safety profile.

This study will be conducted in 3 phases: a screening phase, a treatment phase stratified by tumor origin (four different cohort) and a follow up phase.

3.2 Study schema

Screening phase

Screening will occur between Day -28 and Day -1. The purpose of the screening period is to establish protocol eligibility. Informed consent will be obtained up to 4 weeks prior to Cycle 1 Day 1 and after the study has been fully explained to each subject and prior to the conduct of any screening procedures or assessments.

The purpose of the baseline visit is to establish disease characteristics prior to allocation and treatment and to confirm protocol eligibility as specified in the inclusion/exclusion criteria. Results of baseline assessments must be obtained prior to the first dose of study drug (Cycle 1/Day 1). Baseline assessments may be performed on Day -1 or on Cycle 1/Day 1 prior to dosing. Clinical laboratory tests including pregnancy test (where applicable) can be performed within 72 hours of the first dose of study drug. Subjects who complete the baseline visit and continue to meet the criteria for inclusion/exclusion will begin the treatment phase of this study.

Determinations performed by routine clinical practice before signing the informed consent will be valid, provided they are carried out following this protocol requirements and date of baseline TC is performed within the previous 4 weeks from treatment initiation. It should be clearly stated that patient consented her/his participation to ESR 15-11561-61–DUNE trial in patient record.

Treatment phase

The treatment phase will begin at the time of allocation of the first patient and will consist of the study treatment cycles. The treatment phase will end when the last patient discontinue the study drug.

The treatment phase will include two stages for each cohort. The first stage will dose the first patient in each cohort in a safety run period for one cycle (4 weeks). If no unexpected side effects are observed in the first patient treated in each cohort during the safety run period, recruitment will continue up to 31 patients for cohorts 1 to 3, and 33 for cohort 4.

Subjects will be allocated in each primary tumor cohort to receive durvalumab 1500 mg Q4W for 12 months plus tremelimumab 75 mg Q4W for up to 4 doses during the first 4 cycles of combined therapy. Cycles are defined by 4 weeks or 28 days. Subjects will undergo safety and efficacy assessment as defined per protocol.

If a toxicities pause occurs, cycles not administered for that toxicity can be delayed for a maximum of 4 weeks, after that time that cycle will be considered lost and the next cycle will be administered. Remember no restarts are allowed after a delay of more than 12 weeks due to toxicity or more than 4 weeks of delay if **the reason is different from toxicity** without consulting the Coordinating Investigator.

Follow-up phase

Patients will finish the treatment phase after the administration of 12 months (13 cycles) of durvalumab or after disease progression during the treatment phase. For patients that complete all scheduled treatment, the follow-up phase will begin to determine survival endpoints. Tumor assessments will continue every 12 weeks and clinical appointments will start monthly during the first 3 months of follow-up, every 2 months until first year, and continue every 6 months until disease progression.

3.3 Study Oversight for Safety Evaluation

A subject may elect to discontinue study drug at any time for safety, medical, or personal reasons. Patients who choose to discontinue study drug prior to disease progression will be followed in the post study treatment follow up period and continue to undergo regularly scheduled disease assessment until documentation of disease progression or start of an alternative anticancer treatment. All subjects who discontinue study drug will be followed for overall survival and all post progression cancer treatments administered will be recorded. Subjects may at any time withdraw consent for further study participation. No further data will be collected on subjects once consent has been withdrawn. The investigator will promptly explain to the subject involved that the study drug will be discontinued for that subject and provide appropriate medical treatment and other necessary measures for the subject. A subject who has ceased to return for visits will be followed up by mail, phone, or other means as much as possible to gather information such as the reason for failure to return and the status of treatment compliance, presence or absence of adverse events, and clinical courses of signs and symptoms, and the information will be recorded in the CRF.

Subjects who discontinue early from the study or treatment will be discontinued for 1 of these

primary reasons: adverse event(s), lost to follow-up, subject choice, progressive disease, or administrative/other. In addition to the primary reason, the subject may have indicated 1 or more of these reasons as secondary reasons for discontinuation. Study disposition information will be collected on the appropriate CRF. A subject removed from the study for any reason may not be replaced.

Safety will be assessed by monitoring and recording all AEs including all CTCAE grades (for both increasing and decreasing severity) and serious adverse events (SAEs); regular monitoring of hematology, clinical chemistry, and urine values; physical examinations; and regular measurement of vital signs, and electrocardiograms (ECGs) as detailed in the schedule of visits and procedures.

4. PATIENT SELECTION, ENROLLMENT, ALLOCATION, RESTRICTIONS, DISCONTINUATION AND WITHDRAWAL

Approximately 126 patients will be enrolled and allocated in a balanced manner into either the four cohorts of patients at approximately 20 sites in Spain. Each patient must meet all of the inclusion criteria (Section 4.1) and none of the exclusion criteria (Section 4.2) for this study. Under no circumstances, will there be exceptions to this rule.

4.1 Inclusion criteria

For inclusion in the study, patients should fulfill the following criteria:

1. Written informed consent obtained from the subject prior to performing any protocol-related procedures.

2. Age > 18 years at time of study entry.

3. Subjects must have histologically confirmed diagnosis of one of the following advanced/metastatic neuroendocrine tumor types:

   a) **Cohort 1:** Well-moderately differentiated neuroendocrine tumors of the lung (mitotic count ≤10 mitoses x 10 HPF), also known as typical and atypical lung carcinoids, that have progressed to prior somatostatin analog therapy and/or one prior targeted therapy or chemotherapy (only one prior systemic therapy, with the exception of patients that have been treated with somatostatin analogues and other systemic treatment, when two prior treatments are allowed).

   b) **Cohort 2:** Well-moderately differentiated neuroendocrine tumors G1/G2 (WHO grade 1 and 2) gastrointestinal neuroendocrine tumors after progression to somatostatin analogs and one targeted therapy (prior targeted therapy could be everolimus or a multikinase inhibitor). Prior therapies with interferon alpha-2b or radionucleotide therapy are allowed.

   c) **Cohort 3:** Well-moderately differentiated neuroendocrine tumors G1/G2 (WHO grade 1 and 2) from pancreatic origin after progression to standard therapies (chemotherapy, somatostatin analogs and target therapy); patients must be treated with at least two prior systemic treatment lines and a maximum of four previous treatment lines.

   d) **Cohort 4**: Neuroendocrine neoplasms (WHO grade 3) of gastroenteropancreatic origin

or unknown primary site (excluding lung primary tumors), patients will be treated in second line only, after progression to first-line chemotherapy with a platinum based regimen. Patients will be treated in second line."

4. For patients included in cohorts 1, 2 and 3: WHO Classification G1/G2 (mitotic count ≤10 mitoses x 10 HPF) lung typical and atypical carcinoids for cohort 1, G1/G2 (Ki67≤20% or mitotic count ≤20 mitoses x 10 HPF) gastrointestinal for cohort 2 (including stomach, small intestine and colorectal origins), G1/G2 (Ki67≤20% or mitotic count ≤20 mitoses x 10 HPF) pancreatic for cohort 3.

5. For patients included in cohort 4: WHO classification G3 (Ki67≥20% or mitotic count >20 mitoses x 10 HPF) gastroenteropancreatic neuroendocrine carcinomas (NEC) or liver metastases of G3 NEC of unknown primary site.

6. Subjects must have evidence of measurable disease meeting the following criteria:

a) In case of more than one target lesion, it should be identified at least 1 lesion of ≥ 1.0 cm in the longest diameter for a non lymph node, or ≥ 1.5 cm in the short-axis diameter for a lymph node, which is serially measurable according to RECIST 1.1 using computerized tomography/magnetic resonance imaging (CT/MRI). If there is only one target lesion and it is a non-lymph node, it should have a longest diameter of ≥ 1.5 cm.

b) Lesions that have had external beam radiotherapy (EBRT) or loco-regional therapies such as radiofrequency (RF) ablation or liver embolization must show evidence of progressive disease based on RECIST 1.1 to be deemed a target lesion.

c) Subjects must show evidence of disease progression by radiologic image techniques within 12 months (an additional month will be allowed to accommodate actual dates of performance of scans, i.e., within ≤ 13 months) prior to signing informed consent, according to RECIST 1.1.

7. Eastern Cooperative Oncology Group (ECOG) performance status of 0 or 1.

8. Life expectancy of at least 12 weeks.

9. Adequate normal organ and marrow function as defined below:

- Haemoglobin ≥ 9.0 g/dL.

- Absolute neutrophil count (ANC) ≥ 1.5 x 10^9/L (≥ 1500 per mm^3).

- Platelet count ≥ 100 x 10^9/L (≥ 100,000 per mm^3).

10. Serum bilirubin ≤ 1.5 x institutional upper limit of normal (ULN). This will not apply to subjects with confirmed Gilbert's syndrome (persistent or recurrent hyperbilirubinemia that is predominantly unconjugated in the absence of hemolysis or hepatic pathology), who will be allowed only in consultation with their physician.

11. AST (SGOT)/ALT (SGPT) ≤ 2.5 x institutional upper limit of normal unless liver metastases are present, in which case it must be ≤ 5x ULN.

12. Serum creatinine CL>40 mL/min by the Cockcroft-Gault formula (Cockcroft and Gault 1976) or

by 24-hour urine collection for determination of creatinine clearance:

Males:

$$\text{Creatinine CL (mL/min)} = \frac{\text{Weight (kg)} \times (140 - \text{Age})}{72 \times \text{serum creatinine (mg/dL)}}$$

Females:

$$\text{Creatinine CL (mL/min)} = \frac{\text{Weight (kg)} \times (140 - \text{Age})}{72 \times \text{serum creatinine (mg/dL)}} \times 0.85$$

13. Female subjects must either be of non-reproductive potential (ie, post-menopausal by history: ≥60 years old and no menses for ≥1 year without an alternative medical cause; OR history of hysterectomy, OR history of bilateral tubal ligation, OR history of bilateral oophorectomy) or must have a negative serum pregnancy test upon study entry.

14. Subject is willing and able to comply with the protocol for the duration of the study including undergoing treatment and scheduled visits and examinations including follow up.

4.2 Exclusion criteria

Subjects who meet any of the following criteria will be excluded from this study:

1. Involvement in the planning and/or conduct of the study.

2. Participation in another clinical study with an investigational product during the last 4 weeks.

3. WHO Classification G3 neuroendocrine neoplasms of lung origin (oat cell/large cell lung cancer).

4. Prior treatment with anti-PDL-1/anti-PD-1 or anti-CTL4 therapy.

5. Uncontrolled intercurrent illness including, but not limited to, ongoing or active infection, symptomatic congestive heart failure, uncontrolled hypertension, unstable angina pectoris, cardiac arrhythmia, active peptic ulcer disease or gastritis, active bleeding diathesis including any subject known to have evidence of acute or chronic hepatitis B (e.g., HBsAg reactive), hepatitis C (e.g., HCV RNA [qualitative] is detected) or known history of Human Immunodeficiency Virus (HIV) (HIV 1/2 antibodies), or psychiatric illness/social situations that would limit compliance with study requirements or compromise the ability of the subject to give written informed consent.

6. Known history of previous clinical diagnosis of tuberculosis.

7. Current or prior use of immunosuppressive medication within 28 days before the first dose of durvalumab or tremelimumab, with the exceptions of intranasal and inhaled corticosteroids or systemic corticosteroids at physiological doses, which are not to exceed 10 mg/day of prednisone, or an equivalent corticosteroid.

8. Active or prior documented autoimmune disease within the past 2 years

NOTE: Subjects with vitiligo, Grave's disease, or psoriasis not requiring systemic treatment (within the past 2 years) are not excluded.

9. Active or prior documented inflammatory bowel disease (e.g., Crohn's disease, ulcerative

colitis).

10. History of allogeneic organ transplant.

11. History of hypersensitivity to durvalumab, tremelimumab or any excipient.

12. Subjects having a diagnosis of immunodeficiency or are receiving systemic steroid therapy or any other form of immunosuppressive therapy within 28 days prior to the first dose of trial treatment.

13. Knowledge of active central nervous system (CNS) metastases and/or carcinomatous meningitis. Subjects with previously treated brain metastases may participate provided they have stable brain metastases [without evidence of progression by imaging confirmed [by magnetic resonance imaging (MRI) if MRI was used at prior imaging, or confirmed by computed tomography (CT) imaging if CT used at prior imaging] for at least four weeks prior to the first dose of trial treatment; also, any neurologic symptoms must have returned to baseline], have no evidence of new or enlarging brain metastases, and have not used steroids for brain metastases for at least 7 days prior to trial treatment. This exception does not include carcinomatous meningitis, as subjects with carcinomatous meningitis are excluded regardless of clinical stability.

14. Receipt of live attenuated vaccination within 30 days prior to study entry or within 30 days of receiving durvalumab or tremelimumab. Note: The killed virus vaccines used for seasonal influenza vaccines for injection are allowed; however intranasal influenza vaccines (e.g., FluMist®) are live attenuated vaccines, and are not allowed.

15. Subjects having known history of, or any evidence of interstitial lung disease or active, noninfectious pneumonitis.

16. Any prior Grade ≥3 immune-related adverse event (irAE) while receiving any previous immunotherapy agent, or any unresolved irAE >Grade 1.

17. Subjects who have received any anti-cancer treatment within 21 days or any investigational agent within 30 days prior to the first dose of study drug and should have recovered from any toxicity related to previous anti-cancer treatment. This does not apply to the use of somatostatin analogues for symptomatic therapy.

18. Major surgery within 3 weeks prior to the first dose of study drug.

19. Subjects having > 1+ proteinuria on urine dipstick testing will undergo 24h urine collection for quantitative assessment of proteinuria. Subjects with urine protein ≥ 1 g/24h will be ineligible.

20. Significant cardiovascular impairment: history of congestive heart failure greater than New York Heart Association (NYHA) Class II, unstable angina; myocardial infarction or stroke within 6 months of the first dose of study drug, or cardiac arrhythmia requiring medical treatment.

21. Mean QT interval corrected for heart rate (QTc) ≥470 ms calculated from 3 electrocardiograms (ECGs) using Fredericia's Correction.

22. Bleeding or thrombotic disorders or use of anticoagulants, such as warfarin, or similar agents requiring therapeutic international normalized ratio (INR) monitoring. Treatment with low molecular weight heparin (LMWH) is allowed.

23. Active hemoptysis (bright red blood of at least 0.5 teaspoon) within 3 weeks prior to the first dose of study drug.

24. Patients with tumoral disease in the head and neck region, such as paratracheal or periesophageal lymph node involvement, or with infiltration of structures in the digestive tract, or vascular pathways that represent a risk of increased bleeding.

25. Patients of cohort 1 with neuroendocrine tumors of pulmonary origin or pulmonary metastases with evidence of active bleeding.

26. Patients with evidence of digestive bleeding.

27. Active infection (any infection requiring treatment).

28. Active malignancy (except for differentiated thyroid carcinoma, or definitively treated melanoma in-situ, basal or squamous cell carcinoma of the skin, or carcinoma in-situ of the cervix) within the past 24 months.

29. Female patients who are pregnant or breastfeeding or male or female patients of reproductive potential who are not willing to employ highly effective birth control from screening to 180 days after the last dose of durvalumab + tremelimumab combination therapy or 90 days after the last dose of durvalumab monotherapy, whichever is the longer time period.

30. Documented active alcohol or drug abuse.

31. Patients with a prior history of non-compliance with medical regimens.

32. Any condition that, in the opinion of the investigator, would interfere with evaluation of study treatment or interpretation of patient safety or study results.

4.3 Withdrawal of Subjects from Study Treatment and/or Study

Permanent discontinuation of investigational product/study treatment

An individual subject will not receive any further investigational product if any of the following occur in the subject in question:

1. Withdrawal of consent or lost to follow-up.

2. Adverse event that, in the opinion of the investigator or the sponsor, contraindicates further dosing.

3. Subject is determined to have met one or more of the exclusion criteria for study participation at study entry and continuing investigational therapy might constitute a safety risk.

4. Pregnancy or intent to become pregnant.

5. Any AE that meets criteria for discontinuation as defined in Section 10.3.

6. Grade ≥ 3 infusion reaction.

7. Subject noncompliance that, in the opinion of the investigator or sponsor, warrants withdrawal; eg, refusal to adhere to scheduled visits.

8. Initiation of alternative anticancer therapy including another investigational agent.

9. Confirmed PD by RECIST 1.1 (CT scan or MRI at 4-6 weeks that confirms progression) and investigator determination that the subject is no longer benefiting from treatment with durvalumab + tremelimumab. Patients with documented tumor progression by image (all time-points but especially at 12 weeks), the progression should be confirmed with an additional imaging test (same characteristics) within 4-6 weeks. Treatment should continue according to protocol, until disease progression is confirmed, according the aforementioned procedure.

10. No restarts are allowed after a delay of more than 12 weeks due to toxicity or more than 4 weeks of delay if **the reason is different from toxicity** without consulting the Coordinating Investigator.

Subjects who are permanently discontinued from further receipt of investigational product, regardless of the reason (withdrawal of consent, due to an AE, other), will be identified as having permanently discontinued treatment.

Subjects who are permanently discontinued from receiving investigational product will be followed for safety per Section 10.0 and Appendix 1 or 2, including the collection of any protocol-specified blood specimens, unless consent is withdrawn or the subject is lost to follow-up or enrolled in another clinical study. All subjects will be followed for survival. Subjects who decline to return to the site for evaluations will be offered follow-up by phone every **3 months as an alternative**.

Withdrawal of consent

If consent is withdrawn, the subject will not receive any further investigational product or further study observation.

4.4 Replacement of subjects

If a patient withdraws from participation in the study, then his or her enrollment/allocation code cannot be reused. Withdrawn patients will not be replaced.

5. INVESTIGATIONAL PRODUCT(s)

5.1 Durvalumab and tremelimumab

The sponsor will supply durvalumab and tremelimumab to the site pharmacies as a solution for infusion after dilution.

5.1.1 Formulation/packaging/storage

The trial medication (i.e., Durvalumab and Tremelimumab) and its packaging will be labeled in accordance with annex 13 of EU to Good Manufacturing Practice.

Durvalumab

Durvalumab will be supplied as a 500-mg vial solution for infusion after dilution. The solution

contains 50 mg/mL durvalumab, 26 mM histidine/histidine-hydrochloride, 275 mM trehalose dihydrate, and 0.02% (weight/volume) polysorbate 80; it has a pH of 6.0. The nominal fill volume is 10 mL. Investigational product vials are stored at 2°C to 8°C (36°F to 46°F) and must not be frozen. Durvalumab must be used within the individually assigned expiry date on the label.

Tremelimumab

Tremelimumab will be supplied as a 400-mg vial or 25 mg solution for infusion after dilution. The solution contains 20 mg/mL of tremelimumab, 20 mM histidine/histidine hydrochloride, 222 mM trehalose dihydrate, 0.02% (w/v) polysorbate 80, and 0.27 mM disodium edetate dihydrate (EDTA);it has a pH of 5.5.The nominal fill volume is 20 mL. Investigational product vials are stored at 2°C to 8°C (36°F to 46°F) and must not be frozen. Tremelimumab must be used within the individually assigned expiry date on the label.

5.2 Dose and treatment regimens

5.2.1 Treatment regimen

Patients will receive 1500 mg durvalumab via IV infusion q4w for up to 4 doses/cycles and 75 mg tremelimumab via IV infusion q4w for up to 4 doses/cycles, and then continue 1500 mg durvalumab q4w starting on Week 16 for up to 8 months (9 doses). Dosing outside the window should be discussed with the Study Physician. Tremelimumab will be administered first. Durvalumab infusion will start approximately 1 hour after the end of tremelimumab infusion. The duration will be approximately 1 hour for each infusion. A 1-hour observation period is required after the first infusion of durvalumab and tremelimumab. If no clinically significant infusion reactions are observed during or after the first cycle, subsequent infusion observation periods can be at the Investigator's discretion (suggested 30 minutes after each durvalumab and tremelimumab infusion).

5.2.2 Duration of treatment and criteria for retreatment

Retreatment is allowed (once only) for patients meeting the retreatment criteria below. The same treatment guidelines followed during the initial 12-month treatment period will be followed during the retreatment period, including the same dose and frequency of treatments and the same schedule of assessments.

Patients receiving the combination of durvalumab and tremelimumab may undergo retreatment in 2 clinical scenarios, described below:

1. Patients who achieve and maintain disease control (ie, CR, PR, or SD) through to the end of the 12-month treatment period may restart treatment with the combination upon evidence of PD, with or without confirmation according to RECIST 1.1, during follow-up.

2. Patients who complete the 4 dosing cycles of the combination of durvalumab and tremelimumab portion of the regimen (with clinical benefit per Investigator judgment), but subsequently have evidence of PD during the durvalumab monotherapy portion of the combination regimen, with or without confirmation according to RECIST 1.1, may restart treatment with the combination.

Before restarting their assigned treatment, the Investigator should ensure that the patient:

1. Does not have any significant, unacceptable, or irreversible toxicities that indicate continuing

treatment will not further benefit the patient.

2. Still fulfills the eligibility criteria for this study, including re-consenting to restart durvalumab and tremelimumab.

3. Has not had received an intervening systemic anticancer therapy after their assigned treatment discontinuation.

4. Has had a baseline tumor assessment within 28 days of restarting their assigned treatment; all further scans should occur with the same frequency as during the initial 12 months of treatment (relative to the date of randomization) until study treatment is stopped (maximum of 12 months of further treatment).

During the retreatment period, patients receiving durvalumab + tremelimumab may resume durvalumab dosing at 1500 mg q4w with 75 mg of tremelimumab q4w for 4 doses each. Patients will then continue with durvalumab monotherapy at 1500 mg q4w, beginning at Week 16, 4 weeks after the last dose of combination therapy (a total of 9 additional doses).

Treatment through progression is at the Investigator's discretion, and the Investigator should ensure that patients do not have any significant, unacceptable, or irreversible toxicities that indicate that continuing treatment will not further benefit the patient. A patient with a confirmed progression receiving durvalumab + tremelimumab cannot continue therapy or obtain retreatment if dosing is ongoing in the combination portion of therapy (q4w dosing) and progression occurs in a target lesion that has previously shown a confirmed response.

Patients who the sponsor and/or the Investigator determine may not continue treatment will enter follow-up.

5.2.3 Study drug preparation of durvalumab and tremelimumab

Based on average body WT of 75 kg, a fixed dose of 750 mg Q2W durvalumab (equivalent to 10 mg/kg Q2W), 1500 mg Q4W durvalumab (equivalent to 20 mg/kg Q4W) and 75 mg Q4W tremelimumab (equivalent to 1 mg/kg Q4W) is included in the current study.

Preparation of durvalumab doses for administration with an IV bag

The dose of durvalumab for administration must be prepared by the Investigator's or site's designated IP manager using aseptic technique. Total time from needle puncture of the durvalumab vial to the start of administration should not exceed:

- 24 hours at 2°C to 8°C
- 4 hours at room temperature

If in-use storage time exceeds these limits, a new dose must be prepared from new vials. Infusion solutions must be allowed to equilibrate to room temperature prior to commencement of administration.

No incompatibilities between durvalumab and polyvinylchloride or polyolefin IV bags have been observed. Dose of 1500mg durvalumab for patients ≥30 kg will be administered using an IV bag containing 0.9% (w/v) saline (compatibility with dextrose has been established and may be used as a diluent option, if preferred), with a final durvalumab concentration ranging from 1 to 20 mg/mL,

and delivered through an IV administration set with a 0.2- or 0.22-µm in-line filter. Remove 30.0 mL of IV solution from the IV bag prior to addition of durvalumab. Next, 30.0 mL of durvalumab (ie, 1500 mg of durvalumab) is added to the IV bag such that final concentration is within 1 to 20 mg/mL (IV bag volumes 100 to 1000 mL). Mix the bag by gently inverting to ensure homogeneity of the dose in the bag.

Patient weight at baseline should be used for dosing calculations unless there is a ≥10% change in weight. Dosing day weight can be used for dosing calculations instead of baseline weight per institutional standard.

For patients <30kg, Calculate the dose volume of durvalumab and tremelimumab and number of vials needed for the subject to achieve the accurate dose.

Durvalumab will be administered at room temperature (approximately 25°C) by controlled infusion via an infusion pump into a peripheral or central vein. Following preparation of durvalumab, the entire contents of the IV bag should be administered as an IV infusion over approximately 60 minutes (±5 minutes), using a 0.2, or 0.22-µm in-line filter. Less than 55 minutes is considered a deviation.

The IV line will be flushed with a volume of IV solution (0.9% [w/v] saline equal to the priming volume of the infusion set used after the contents of the IV bag are fully administered, or complete the infusion according to institutional policy to ensure the full dose is administered and document if the line was not flushed.

Standard infusion time is 1 hour. However, if there are interruptions during infusion, the total allowed time should not exceed 8 hours at room temperature. The table below summarizes time allowances and temperatures.

Durvalumab hold and infusion times

Maximum time from needle puncture to start of administration	4 hours at room temperature, 24 hours at 2°C to 8°C
Maximum time for IV bag infusion, including interruptions	8 hours at room temperature

In the event that either preparation time or infusion time exceeds the time limits outlined in the table, a new dose must be prepared from new vials. Durvalumab does not contain preservatives, and any unused portion must be discarded.

Preparation of tremelimumab doses for administration with an IV bag

The dose of tremelimumab for administration must be prepared by the Investigator's or site's designated IP manager using aseptic technique. Total time from needle puncture of the tremelimumab vial to the start of administration should not exceed:

- 24 hours at 2°C to 8°C
- 4 hours at room temperature

It is recommended that the prepared final IV bag be stored in the dark at 2ºC-8°C until needed. If storage time exceeds these limits, a new dose must be prepared from new vials. The refrigerated infusion solutions in the prepared final IV bag should be equilibrated at room temperature for about 2 hours prior to administration. Tremelimumab does not contain preservatives and any unused

portion must be discarded.

No incompatibilities between tremelimumab and polyvinylchloride or polyolefin IV bags have been observed. Doses of 75 mg tremelimumab for patients ≥30 kg will be administered using an IV bag containing 0.9% (w/v) saline, with a final tremelimumab concentration ranging from 0.1 mg/mL to 10 mg/mL, and delivered through an IV administration set with a 0.2 µm or 0.22 µm in-line filter. Remove 3.8 mL of IV solution from the IV bag prior to addition of tremelimumab. Next, 3.8 mL of tremelimumab (ie, 75 mg of tremelimumab) is added to the IV bag such that final concentration is within 0.1 mg/mL to 10 mg/mL (IV bag volumes 50 to 500 mL). Mix the bag by gently inverting to ensure homogeneity of the dose in the bag.

Patient weight at baseline should be used for dosing calculations unless there is a ≥10% change in weight. Dosing day weight can be used for dosing calculations instead of baseline weight per institutional standard.

For patients <30 kg, Calculate the dose volume for tremelimumab and number of vials needed for subject to achieve the accurate dose.

Tremelimumab will be administered at room temperature (approximately 25°C) by controlled infusion via an infusion pump into a peripheral or central vein. Following preparation of tremelimumab, the entire contents of the IV bag should be administered as an IV infusion over approximately 60 minutes (±5 minutes), using a 0.2, or 0.22-µm in-line filter. Less than 55 minutes is considered a deviation.

The IV line will be flushed with a volume of 0.9% (w/v) saline equal to the priming volume of the infusion set used after the contents of the IV bag are fully administered, or complete the infusion according to institutional policy to ensure the full dose is administered and document if the line was not flushed.

Standard infusion time is 1 hour. However, if there are interruptions during infusion, the total allowed time should not exceed 8 hours at room temperature. The table below summarizes time allowances and temperatures.

Tremelimumab hold and infusion times

Maximum time from needle puncture to start of administration	4 hours at room temperature, 24 hours at 2ºC to 8ºC
Maximum time for IV bag infusion, including interruptions.	8 hours at room temperature

In the event that either preparation time or infusion time exceeds the time limits outlined in the table, a new dose must be prepared from new vials. Tremelimumab does not contain preservatives, and any unused portion must be discarded.

5.2.4 Monitoring of dose administration

Patients will be monitored during and after the infusion with assessment of vital signs at the times specified in the Study Protocol.

In the event of a ≤ Grade 2 infusion-related reaction, the infusion rate of study drug may be decreased by 50% or interrupted until resolution of the event and re-initiated at 50% of the initial rate until completion of the infusion. For patients with a ≤Grade 2 infusion-related reaction, subsequent infusions may be administered at 50% of the initial rate. Acetaminophen and/or an

antihistamine (eg, diphenhydramine) or equivalent medications per institutional standard may be administered at the discretion of the investigator. If the infusion-related reaction is ≥Grade 3 or higher in severity, study drug will be discontinued.

As with any antibody, allergic reactions to dose administration are possible. Appropriate drugs and medical equipment to treat acute anaphylactic reactions must be immediately available, and study personnel must be trained to recognize and treat anaphylaxis. The study site must have immediate access to emergency resuscitation teams and equipment in addition to the ability to admit patients to an intensive care unit if necessary.

5.2.5 Accountability and dispensation

The trial medication will be sent to the investigator's site pharmacy preceded by the Regulatory Green Light. The medication is to be used exclusively in the clinical trial according to the instructions of this trial protocol.

When a drug shipment is received, the Investigator or designee will check the amount and condition of the delivery, drug expiration date, and sign the Receipt of Shipment Form provided. The Receipt of Shipment Form should be faxed or emailed to the CRO (see appendix 8). The original form will preliminarily be retained at the site and will be collected at the next monitoring visit by the monitor and stored in the Trial Master File at CRO. A copy remains in the Investigator File at the site. In case of shipment problems the Investigator or designee shall contact the CRA as soon as possible.

An Investigational Product Accountability Log will be provided for the trial medication. The record must be continuously updated and contain the dates, quantities and compounds of drugs received, medication identification number(s), the patient identification number to whom the trial medication was dispensed, date and quantity of medication dispense and the initials of the dispenser.

5.2.6 Disposition of unused investigational study drug

Trial medication will be monitored by the CRA at the respective hospital pharmacy prior to destruction after having completed a final inventory. Local or institutional regulations may require immediate destruction of the study drug used for safety reasons, e.g., cytotoxicity or to maintain the storage capacity and functionality of the storage at the site. In these cases, it may be acceptable to destroy it by the research staff, including partially used and empty vials, dispensed before a monitoring inspection, if the verification of original documents of empty boxes that indicate the information of batch number and dispensing date to the patient on the label. This documentation will be verified against the quantity shipped, dispensed, returned and destroyed.

Prior to the destruction a final trial medication reconciliation statement must be completed. Drug supplies will be destroyed according to the legal requirements in Spain.

All trial medication inventory forms must be made available for inspection by a Sponsor authorized representative or designee and regulatory agency inspectors. The Investigator is responsible for the accountability of all used and unused study supplies at the site.

6. TREATMENT PLAN

6.1 Subject enrollment and allocation

6.1.1 Procedures for allocation

Patients will be allocated in one of the following cohorts regarding primary tumor site and WHO grading classification:

- Cohort 1: Well-moderately differentiated lung neuroendocrine tumors (classically known as typical and atypical carcinoids) after progression to somatostatin analogs and one prior targeted therapy or chemotherapy.
- Cohort 2: G1/G2 (WHO grade 1 and 2) gastrointestinal neuroendocrine tumors after progression to somatostatin analogs and one prior targeted therapy.
- Cohort 3: G1/G2 (WHO grade 1 and 2) pancreatic neuroendocrine tumors after progression to standard therapies (chemotherapy, somatostatin analogs and target therapy), who have received between two and four prior lines of treatment.
- Cohort 4: Neuroendocrine neoplasms (WHO grade 3) of gastroenteropancreatic origin or unknown primary site (excluding lung primary tumors) after progression to first-line chemotherapy with a platinum based regimen. Patients will be treated in second line.

6.1.2 Procedures for handling subjects incorrectly enrolled

Patients incorrectly enrolled in the trial will be considered as protocol deviations. If investigator considerer that the patient is obtaining benefit of the investigational treatment, patients could continue on therapy. They will not be considered for the primary endpoint of the trial, neither for efficacy nor biomarker secondary and exploratory analyses. Patients incorrectly enrolled that continue on treatment can be assessed for safety profile endpoint.

6.2 Dose Modification and Toxicity Management

6.2.1 Durvalumab and tremelimumab

For adverse events (AEs) that are considered at least partly due to administration of durvalumab and/or tremelimumab the following dose adjustment guidance may be applied:

- Treat each of the toxicities with maximum supportive care (including holding the agent suspected of causing the toxicity where required).
- If the symptoms promptly resolve with supportive care, consideration should be given to continuing the same dose of durvalumab or tremelimumab along with appropriate continuing supportive care. If medically appropriate, dose modifications are permitted for durvalumab and tremelimumab (see Appendix 1).
- All dose modifications should be documented with clear reasoning and documentation of the approach taken.

In addition, there are certain circumstances in which durvalumab or tremelimumab should be permanently discontinued.

Following the first dose of durvalumab or tremelimumab, subsequent administration of durvalumab or tremelimumab can be modified based on toxicities observed (see Appendix 1).

Based on the mechanism of action of durvalumab or tremelimumab leading to T-cell activation and proliferation, there is the possibility of observing immune related Adverse Events (irAEs) during the conduct of this study. Potential irAEs include immune-mediated enterocolitis, dermatitis, hepatitis/hepatotoxicity, endocrinopathy, pneumonitis, nephritis, pancreatitis and neuropathy or neurologic events. Subjects should be monitored for signs and symptoms of irAEs. In the absence of an alternate etiology (e.g., infection or PD) signs or symptoms of enterocolitis, dermatitis, hepatitis/hepatotoxicity, endocrinopathy, pneumonitis, nephritis, pancreatitis and neuropathy or neurologic events should be considered to be immune-related.

Dose modification recommendations and toxicity management guidelines for immune-mediated reactions, for infusion-related reactions, and for non-immune-mediated reactions are detailed in Appendix 1.

In addition, management guidelines for adverse events of special interest (AESIs) are detailed in Section 10.1.3. All toxicities will be graded according to NCI CTCAE v4.03.

7. RESTRICTIONS DURING THE STUDY AND CONCOMITANT TREATMENT(s)

7.1 Restrictions during the study

The following restrictions apply while the patient is receiving study treatment and for the specified times before and after:

- Female subjects of childbearing potential who are sexually active with a non-sterilized male partner must use one highly effective method of contraception (Table 2) from the time of screening and must agree to continue using such precautions for 180 days after the last dose of durvalumab + tremelimumab combination therapy or 90 days after the last dose of durvalumab monotherapy, whichever is the longer time period. Male partners of a female subject must use male condom plus spermicide throughout this period. Cessation of birth control after this point should be discussed with a responsible physician. Not engaging in sexual activity for the total duration of the trial and the drug washout period is an acceptable practice; however, occasional abstinence, the rhythm method, and the withdrawal method are not acceptable methods of contraception..
- Females of childbearing potential are defined as those who are not surgically sterile (ie, bilateral tubal ligation, bilateral oophorectomy, or complete hysterectomy) or post-menopausal (defined 12 months with no menses without an alternative medical cause).
- Non-sterilized male subjects who are sexually active with a female partner of childbearing potential must use male condom plus spermicide from screening through 180 days after receipt of the final dose of durvalumab + tremelimumab combination therapy or 90 days after receipt of the final dose of durvalumab monotherapy, whichever is the longer time period. Female partners of a male subject must use a highly effective method of

contraception throughout this period.

- Highly effective methods of contraception are described in Table 2. A highly effective method of contraception is defined as one that results in a low failure rate (i.e. less than 1% per year) when used consistently and correctly. Note that some contraception methods are not considered highly effective (e.g. male or female condom with or without spermicide; female cap, diaphragm, or sponge with or without spermicide; non-copper containing intrauterine device; progestogen-only oral hormonal contraceptive pills where inhibition of ovulation is not the primary mode of action [excluding Cerazette/desogestrel which is considered highly effective]; and triphasic combined oral contraceptive pills).
- Restrictions relating to concomitant medications are described in Section 7.2.

Table 2. Highly Effective[a] Methods of Contraception

Barrier/ Intrauterine	Hormonal Methods
● Copper T intrauterine<br>● Levonorgestrel-relasing intrauterine system (eg, Mirema)[b]	● Etonogestrel implants: e.g. Implanon or Norplan<br>● Intravaginal device; e.g. ethinylestradiol and etonogestrel<br>● Medroxyprogesterone injection: e.g. Depo-Provera<br>● Normal and low dose combined oral contraceptive pill<br>● Norelgestromin/ethinylestradiol transdermal system<br>● Cerazette (desogestrel)

[a] Highly effective (i.e. failure rate of <1% per year) [b] This is also considered a hormonal method

Blood donation

Subjects should not donate blood while participating in this study, or for at least 90 days following the last infusion of durvalumab or tremelimumab, whichever occurs longest.

7.2 Concomitant treatment(s)

The Principal Investigator must be informed as soon as possible about any medication taken from the time of screening until the end of the clinical phase of the study (final study visit). Any concomitant medication(s), including herbal preparations, taken during the study will be recorded in the CRF.

Restricted, prohibited, and permitted concomitant medications are described in the following tables.

7.2.1 Permitted concomitant medications

Investigators may prescribe concomitant medications or treatments (e.g., acetaminophen, diphenhydramine) deemed necessary to provide adequate prophylactic or supportive care except for those medications identified as “excluded” as listed in Section 7.2.2.

Concomitant treatment with somatostatin analogues (octreotide or lanreotide) are allowed for

hormonal symptom control.

7.2.2 Excluded Concomitant Medications

The following medications are considered exclusionary during the study.

1. Any investigational anticancer therapy other than the protocol specified therapies.
2. Any concurrent chemotherapy, radiotherapy (except palliative radiotherapy), immunotherapy, biologic or hormonal therapy for cancer treatment. Concurrent use of hormones for noncancer-related conditions (e.g., insulin for diabetes and hormone replacement therapy) is acceptable. NOTE: Local treatment of isolated lesions for palliative intent is acceptable (e.g., by local surgery or radiotherapy).
3. Immunosuppressive medications including, but not limited to systemic corticosteroids at doses not exceeding 10 mg/day of prednisone or equivalent, methotrexate, azathioprine, and TNF-α blockers. Use of immunosuppressive medications for the management of investigational product-related AEs or in subjects with contrast allergies is acceptable. In addition, use of inhaled and intranasal corticosteroids is permitted. A temporary period of steroids will be allowed for different indications, at the discretion of the principal investigator (e.g., chronic obstructive pulmonary disease, radiation, nausea, etc).
4. Live attenuated vaccines within 30 days of durvalumab and tremelimumab dosing (ie, 30 days prior to the first dose, during treatment with durvalumab and tremelimumab for 30 days post discontinuation of durvalumab and tremelimumab. Inactivated vaccines, such as the injectable influenza vaccine, are permitted.

Table 3. Prohibited and Rescue Medications

Prohibited medication/class of drug	Usage
Additional investigational anticancer therapy concurrent with those under investigation in this study	Should not be given whilst the patient is on IP treatment
mAbs against CTLA-4, PD-1, or PD-L1	Should not be given whilst the patient is on IP treatment through 90 days after the last dose of IP.
Any concurrent chemotherapy, local therapy (except palliative radiotherapy for non-target lesions, eg, radiotherapy, surgery, radiofrequency ablation), biologic therapy, or hormonal therapy for cancer treatment	Should not be given whilst the patient is on IP treatment (including SoC). (Concurrent use of hormones for non-cancer-related conditions [eg, insulin for diabetes and hormone replacement therapy] is acceptable.)
Immunosuppressive medications, including, but not limited to, systemic corticosteroids at doses exceeding 10 mg/day of prednisone or its equivalent, methotrexate, azathioprine, and tumor necrosis factor α blockers	Should not be given whilst the patient is on IP treatment (including SoC). (Use of immunosuppressive medications for the management of IP-related AEs or in patients with contrast allergies is acceptable. In addition, use of inhaled, topical, and intranasal corticosteroids is permitted.
Live attenuated vaccines	Should not be given through 30 days after the last dose of IP (including SoC) during the study.
**Rescue/supportive medication/class of drug**	**Usage**
Concomitant medications or treatments (eg, acetaminophen or diphenhydramine) deemed necessary by the Investigator to provide adequate prophylactic or supportive care, except for those medications identified as "prohibited" as listed above.	To be administered as prescribed by the Investigator
Best supportive care (including antibiotics, nutritional support, growth factor support, correction of metabolic disorders, optimal symptom control, and pain management [including palliative radiotherapy, etc])	Should be used when necessary for all patients

8. STUDY PROCEDURES

8.1 Schedule of study procedures

Before study entry, throughout the study, and following study drug discontinuation, various clinical and diagnostic laboratory evaluations are outlined. The purpose of obtaining these detailed measurements is to ensure adequate safety and tolerability assessments. Clinical evaluations and laboratory studies may be repeated more frequently if clinically indicated. The Schedules of Assessments during the screening and treatment period is provided after the Protocol Synopsis.

8.1.1 Screening Phase

Screening procedures will be performed up to 28 days before Day 1, unless otherwise specified. All subjects must first read, understand, and sign the IEC-approved ICF before any study-specific screening procedures are performed. After signing the ICF, completing all screening procedures, and being deemed eligible for entry, subjects will be enrolled in the study. Procedures performed prior to the signing of the ICF are considered standard of care and may be used as screening assessments if they fall within the 28-day screening window.

The following procedures will be performed during the Screening Visit:

- Informed Consent
- Review of eligibility criteria
- Medical history and demographics
- Complete physical exam
- ECOG Performance Status
- Vital signs, weight and height
- 12-lead ECG (in triplicate [2-5 minutes apart])
- Review of prior/concomitant medications
- Record any Aes or SAEs (since ICF signature)
- Imaging by CT/MRI
- Review of octreoscan/PET (up to 6 months prior inclusion) only on cohorts 1 to 3.
- Collect blood samples for biomarkers analysis
- Archival tumor block or slides
- Clinical laboratory tests for:
    - Hematology (see Table 4)
    - Clinical chemistry (see Table 5)
    - TSH, fT3 and fT4

- Coagulation (PT, PTT, INR)
- Creatinine Clearance
- Serum pregnancy test (for women of childbearing potential only)
- Hepatitis and HIV serologies
- Urinalysis (see Table 6)
- Disease-specific tumor markers

8.1.2 Treatment Phase

Procedures to be conducted during the treatment phase of the study are presented in the Schedule of Assessments. Screening procedures performed within 72 hours of Cycle 1 Day 1 (C1D1) do not need to be repeated on C1D1.

Efforts should be made to conduct study visits on the day scheduled (± 1 day). Clinical laboratory assessments may be conducted anytime within 72 hours prior to the scheduled visit, unless otherwise specified in the Schedule of Visits and Procedures. Results of the laboratory assessments should be reviewed always prior each cycle dose administration. Whenever possible, subjects should be evaluated at approximately the same time of the day (e.g., morning or afternoon) at each visit, and reasonable efforts should be made to conduct all evaluations in the same test order at each visit.

Tumor assessments should be performed at time points indicated in the Schedule of Visits and Procedures.

Cycle 1/Day 1

- Obtain vital signs (resting BP, HR, RR, body temperature) and weight.
- Physical examination is not mandatory if performed at baseline (day -1) however a symptom-directed physical examination will be performed on Cycle 1/Day 1 and at any time during the study, as clinically indicated.
- Administer study drug:
  - Record all concomitant medication use.
  - Record any AEs or SAEs.

Cycle 1/Day 15

- Obtain vital signs (resting BP, HR, RR, body temperature) and weight.
- Evaluate ECOG performance status.
- Perform a comprehensive physical examination (including a neurological evaluation). A symptom-directed physical examination will be performed at any time during the study, as clinically indicated.
- Collect blood samples for biochemistry and hematology analyses (see Tables 4 and 5),

including TSH, fT3 and fT4.

- Urinalysis (see Table 6)
- Record all concomitant medication use.
- Record any AEs or SAEs.

Cycle 2/Day 1

- Evaluate ECOG performance status.
- Obtain vital signs (supine BP, HR, RR, body temperature) and weight.
- Perform a comprehensive physical examination (including a neurological evaluation). A symptom-directed physical examination will be performed at any time during the study, as clinically indicated.
- Collect blood samples for biochemistry and hematology analyses (see Tables 4 and 5), including TSH, fT3 and fT4.
- Urinalysis (see Table 6)
- Administer study drug.
- Collect blood samples for biomarker analysis.
- Record all concomitant medication use.
- Record any AEs or SAEs.
- Record survival data.

Cycle 2/Day 15

- Evaluate ECOG performance status.
- Obtain vital signs (supine BP, HR, RR, body temperature) and weight.
- Perform a comprehensive physical examination (including a neurological evaluation). A symptom-directed physical examination will be performed at any time during the study, as clinically indicated.
- Collect blood samples for biochemistry and hematology analyses (see Tables 4 and 5), including TSH, fT3 and fT4.
- Urinalysis (see Table 6)
- Record all concomitant medication use.
- Record any AEs or SAEs.

Cycle 3/Day 1

- Evaluate ECOG performance status.

- Obtain vital signs (supine BP, HR, RR, body temperature) and weight.
- Perform a comprehensive physical examination (including a neurological evaluation). A symptom-directed physical examination will be performed at any time during the study, as clinically indicated.
- Collect blood samples for biochemistry and hematology analyses (see Tables 4 and 5), including TSH, fT3 and fT4.
- Urinalysis (see Table 6)
- Administer study drugs.
- Record all concomitant medication use.
- Record any AEs or SAEs.
- Record Survival data

Cycle 3/Day 15

- Evaluate ECOG performance status.
- Obtain vital signs (supine BP, HR, RR, body temperature) and weight.
- Perform a comprehensive physical examination (including a neurological evaluation). A symptom-directed physical examination will be performed at any time during the study, as clinically indicated.
- Collect blood samples for biochemistry and hematology analyses (see Tables 4 and 5), including TSH, fT3 and fT4.
- Urinalysis (see Table 6)
- Record all concomitant medication use.
- Record any AEs or SAEs.

Cycle 4 Through Last Cycle (cycle 12)/Day 1

- Evaluate ECOG performance status.
- Obtain vital signs (supine BP, HR, RR, body temperature) and weight.
- Perform a comprehensive physical examination (including a neurological evaluation). A symptom-directed physical examination will be performed at any time during the study, as clinically indicated.
- Collect blood samples for biochemistry and hematology analyses (see Tables 4 and 5), including TSH, fT3 and fT4
- Urinalysis (see Table 6)
- Administer study drugs. (from C5D1 to C12D1 tremelimumab no longer administered, only durvalumab).

- Record all concomitant medication use.
- Record any AEs or SAEs.
- Record survival data.
- Tumor assessment every 12 weeks until confirmed PD.

8.1.3 End of Treatment

End of treatment is defined as the last planned dosing visit within the 12-month dosing period. For subjects who discontinue durvalumab or tremelimumab prior to 12 months, end of treatment is considered the last visit where the decision is made to discontinue treatment. All required procedures may be completed within ± 7 days of the end of treatment visit. Repeat disease assessment is not required if performed within 28 days prior to the end of treatment visit.

Blood samples for biomarker analysis are collected.

Assessments for subjects who have completed durvalumab and tremelimumab treatment and achieved disease control, or have discontinued durvalumab or tremelimumab due to toxicity in the absence of confirmed progressive disease are provided in APPENDIX 2.

Assessments for subjects who have discontinued durvalumab or tremelimumab treatment due to confirmed PD are presented in APPENDIX 3.

All subjects will be followed for survival until the end of the study regardless of further treatments, or until the sponsor ends the study.

Follow-up period

TC/MRI every 12 weeks until progression. Monthly visits during the first 3 months after end of treatment, every 2 months until one year, and therefore every 6 months. Determinations to be done are listed in appendix 2 and 3.

8.2 Description of study procedures

8.2.1 Medical history and physical examination, electrocardiogram, weight and vital signs

Findings from medical history (obtained at screening) and physical examination shall be given a baseline grade according to the procedure for AEs. Increases in severity of pre-existing conditions during the study will be considered AEs, with resolution occurring when the grade returns to the pre-study grade or below.

Physical examinations will be performed on study days noted in the Schedule of Assessments.

8.2.2 Physical examination

Physical examinations will be performed according to the assessment schedule. Full physical examinations will include assessments of the head, eyes, ears, nose, and throat and the respiratory, cardiovascular, GI, urogenital, musculoskeletal, neurological, dermatological, hematologic/lymphatic, and endocrine systems. Height will be measured at Screening only. Targeted physical examinations are to be used by the Investigator on the basis of clinical

observations and symptomatology. Situations in which physical examination results should be reported as AEs are described in Section 10.

8.2.3 Electrocardiograms

Resting 12-lead ECGs will be recorded at screening and as clinically indicated throughout the study. ECGs should be obtained after the patient has been in a supine position for 5 minutes and recorded while the patient remains in that position.

At Screening, a single ECG will be obtained on which QTcF must be <470 ms.

In case of clinically significant ECG abnormalities, including a QTcF value >470 ms, 2 additional 12-lead ECGs should be obtained over a brief period (eg, 30 minutes) to confirm the finding.

Situations in which ECG results should be reported as AEs are described in Section 10.0.

8.2.4 Vital signs

Vital signs (blood pressure [BP], pulse, temperature, and respiration rate) will be evaluated according to the assessment schedules.

On infusion days, patients receiving durvalumab + tremelimumab treatment will be monitored during and after infusion of IP as presented in the bulleted list below.

Supine BP will be measured using a semi-automatic BP recording device with an appropriate cuff size, after the patient has rested for at least 5 minutes. BP and pulse will be collected from patients receiving durvalumab + tremelimumab treatment before, during, and after each infusion at the following times (based on a 60-minute infusion):

Prior to the beginning of the infusion (measured once from approximately 30 minutes before up to 0 minutes [ie, the beginning of the infusion]).

Approximately 30 minutes during the infusion (**halfway** through infusion).

At the end of the infusion (approximately 60 minutes ±5 minutes).

A 1-hour observation period is required after the first infusion of durvalumab and tremelimumab.

If no clinically significant infusion reactions are observed during or after the first cycle, subsequent infusion observation periods can be at the Investigator's discretion (suggested 30 minutes after each durvalumab and tremelimumab infusion).

If the infusion takes longer than 60 minutes, then BP and pulse measurements should follow the principles as described above or be taken more frequently if clinically indicated. The date and time of collection and measurement will be recorded on the appropriate eCRF. Additional monitoring with assessment of vital signs is at the discretion of the Investigator per standard clinical practice or as clinically indicated.

Body weight is also recorded along with vital signs.

Situations in which vital signs results should be reported as AEs are described in Section 10.3. A complete physical examination will be performed and will include an assessment of the following (as clinically indicated): general appearance, respiratory, cardiovascular, abdominal, skin, head

and neck (including ears, eyes, nose and throat), lymph nodes, thyroid, musculoskeletal (including spine and extremities), genital/rectal, and neurological systems and at screening only, height.

8.2.5 Clinical laboratory tests

The following clinical laboratory tests will be performed in local hospitals (see the Schedule of Assessments):

- Coagulation parameters: Activated partial thromboplastin time and International normalised ratio to be assessed at baseline and as clinically indicated
- Pregnancy test (female subjects of childbearing potential only)
  - Urine human chorionic gonadotropin
  - Serum beta-human chorionic gonadotropin (at screening only)
- Thyroid Stimulating Hormone
  - Free T3 and free T4 only if TSH is abnormal
- Other laboratory tests
  - Hepatitis B surface antigen, hepatitis C antibody
  - HIV antibody

Table 4. Hematology Laboratory Tests

Basophils	Mean corpuscular volume	Total white cell count
Eosinophils	Monocytes	Mean corpuscular hemoglobin concentration
Hematocrit	Neutrophils	Mean corpuscular hemoglobin
Hemoglobin	Platelet count	
Lymphocytes	Red blood cell count	

Table 5. Clinical chemistry (Serum or Plasma) Laboratory Tests

Albumin	Glucose
Alkaline phosphatase	Lactate dehydrogenase
Alanine aminotransferase	Lipase
Amylase	Magnesium
Aspartate aminotransferase	NSE (Enolase)
Bicarbonate	Potassium
Calcium	Sodium
CgA (Chromogranine)	Total bilirubin[a]
Chloride	Total protein
Creatinine	Urea or blood urea nitrogen, depending on local practice
Gamma glutamyltransferase[b]	Uric acid

[a] If Total bilirubin is ≥2xULN (and no evidence of Gilbert's syndrome) then fraction into direct and indirect bilirubin
[b] At baseline and as clinically indicated.

Table 6. Urinalysis Tests [a]

Bilirubin	pH
Blood	Protein
Glucose	Specific gravity
Ketones	Colour and appearance

[a] Microscopy should be used as appropriate to investigate white blood cells and use the high power field for red blood cells

8.3 Biological sampling procedures

Blood samples for the development of exploratory predictive biomarkers will be collected from all subjects prior to the first dose of study drug, and on Cycle 2/Day 1 and at end-of treatment visit or after documented disease progression, whichever occurs first. Biomarker discovery and validation will be performed to identify blood or tumor biomarkers which may be useful to predict subject response, as determined by evaluation of primary or secondary efficacy endpoints. Plasma samples from study subjects will undergo global proteomic and/or enzyme-linked immunosorbent assay (ELISA)-based analyses or multiplex bead-based immunoassay in an effort to identify protein biomarkers. In addition, DNA and RNA analyses will be performed in search of predictive or prognostic biomarkers and also biomarkers identified in other durvalumab+trametinib clinical studies may also be assessed in samples collected from subjects enrolled in this study.

Archived, fixed tumor tissue from will be collected for all subjects for confirmation of histology and assessment of somatic mutations of genes which may be important in the development and progression of neuroendocrine tumors. Gene-expression profiling (GEP), proteomic, or immunohistochemical (IHC) analysis will be performed based on the amount of tumor tissue available for analysis. All analyses will be limited to correlations relevant to neuroendocrine tumors and clinical outcomes related to treatment with durvalumab and tremelimumab.

Blood and tumor samples collected during the study will be stored at the study sites until the initial primary efficacy and safety analyses of the study will be completed and results will be available. Then, the study Sponsor will decide whether to perform all or part of the pharmacogenetic/pharmacogenomics assessments.

Data obtained will only be used for research, to assist in developing safer and more effective treatments, and will not be used to change the diagnosis of the subject or alter the therapy of the subject. Any DNA derived from the sample may be stored for up to 15 years to assist in any research scientific questions related to durvalumab or tremelimumab. Sample collection will be registered in Spanish National Registry and will be used in research projects in the particular research line foresee in the informed consent. Samples cannot be transferred to third parties, and studies in the future with these samples will require EC approval. Instructions for the processing, storage, and shipping of samples will be provided in the Laboratory Manual.

8.3.1 Biomarker/Pharmacodynamic sampling and evaluation methods

PD-L1 Testing

Retrospectively, PD-L1 assessment will be performed using Ventana SP263 assay in accordance with the package insert on the Ventana Benchmark platform (Ultra or XT) within the GETNE translational working group to ensure comparability of data across all studies of durvalumab and/or tremelimumab.

Sample collection for PD-L1 testing

- The preferred tumor sample for the determination of a patient's PD-L1 status is the one taken following the completion of the most recent prior line of therapy. Samples taken at this time reflect the current PD-L1 status of the tumor and considered clinically most relevant.

- The preferred sample for PD-L1 testing was less than or equal to 3 months old. In cases where a sample a less than 3 months old was not available, patients were asked to undergo a new biopsy if considered clinically appropriate by their treating physician.
- Samples should be collected via a core needle of 18 gauge or larger or be collected by an incisional or excisional tumor biopsy. The provision of 2 cores is advised in order to provide sufficient tissue for PD-L1 assessment and processed to FFPE in a single block. Where institutional practice uses a smaller gauge needle, samples should be evaluated for tumor cell quantity (i.e. >100 tumor cells) to allow for adequate PD-L1 immunohistochemistry analyses.
- When the collection of a new sample is not clinically appropriate, archival samples may be utilized provided the specimen it is not older than 5 years of age. When archival samples are used to assess PD-L1 status, the age of the sample / date of collection should be captured.
- Samples submitted for PD-L1 testing should be formalin fixed and embedded in paraffin. Samples from fine needle aspirates (FNA) or decalcified bone are not appropriate for PD-L1 analysis.

Sample data collection for PD-L1 testing

The following fields of data should be collected from the site/institution collecting and if, indicated shipping of the samples:

Patient identifier (e-code or unique identifier)

- Specimen identifier (written on the specimen)
- Site identifier
- Specimen collection date
- Type of specimen submitted
- Quantity of specimen
- Date of sectioning
- Archival of fresh tumor
- Tumor type
- Primary tumor location
- Metastatic tumor location (if applicable)
- Fixative

The following fields of data should be collected from PD-L1 testing laboratory:

- Are the negative and positive controls stained correctly
- Is the H&E material acceptable

- Is morphology acceptable
- Total percent positivity of PD-L1 in tumor cells
- PD-L1 status (positive, negative or NA) in tumor cells
- Total percent positivity of PD-L1 in infiltrating immune cells

Previously frozen tissue is not acceptable for processing to FFPE for PD-L1 testing. Sections should be stored at ambient temperature and protected from light until use or shipment to testing lab by courier at ambient temperature. It is recommended that slides are cut freshly prior to PD-L1 testing and they are used within 90 days of being cut to obtain PD-L1 status

8.3.2 Estimate of volume of blood to be collected

The total of two blood samples will be drawn from each subject in this study (at baseline, C2D1, end of treatment/progression) will be managed according the laboratory manual and stored -80ºC (-20ºC if not available) in the investigators centers. At the end of the study treatment period and follow-up up, and regarding results obtained, the sponsor and investigators will decide if biomarker analyses will be performed.

8.3.3 Archival tumor samples

8.3.3.1 Archival tumor samples

Archival tumor samples in formalin-fixed paraffin-embedded tissue block will be identified at screening period. Samples will only be used at the end of the study treatment and always regarding results obtained with the investigational drugs.

8.3.4 Withdrawal of informed consent for donated biological samples

If a subject withdraws consent to the use of donated samples, the samples will be disposed of/destroyed, and the action documented.

Participation in biological samples analyses are optional, and patients can participate in the study without giving the consent to use their biological samples.

The Principal Investigator:

- Ensures that biological samples from that subject, if stored at the study site, are immediately identified, disposed of /destroyed, and the action documented
- Ensures the laboratory(ies) holding the samples is/are informed about the withdrawn consent immediately and that samples are disposed/destroyed, the action documented and the signed document returned to the study site
- Ensures that the subject is informed about the sample disposal.

9. DISEASE EVALUATION AND METHODS

Primary Objective

Disease evaluation according RECIST 1.1.

A copy of the RECIST 1.1 guideline will be included in the ISF.

RECIST 1.1 will be modified so that PD must be confirmed at the next scheduled visit, preferably, and no earlier than 4 weeks after the initial assessment of PD in the absence of clinically significant deterioration. Treatment with durvalumab + tremelimumab would continue between the initial assessment of progression and confirmation for progression.

Secondary objectives (irRECIST)

The response to immunotherapy may differ from the typical responses observed with cytotoxic chemotherapy including the following (Wolchok et al 2009, Nishino et al 2013):

- Response to immunotherapy may be delayed
- Response to immunotherapy may occur after PD by conventional criteria
- The appearance of new lesions may not represent PD with immunotherapy
- SD while on immunotherapy may be durable and represent clinical benefit.

Based on the above-described unique response to immunotherapy and based on guidelines from regulatory agencies, e.g., European Medicines Agency's "Guideline on the evaluation of anti-cancer medicinal products in man" (EMA/CHMP/205/95/Rev.4) for immune modulating anti-cancer compounds, the study may wish to implement the following in addition to standard RECIST 1.1 criteria:

- RECIST 1.1 will be modified so that PD must be confirmed at the next scheduled visit, preferably, and no earlier than 4 weeks after the initial assessment of PD in the absence of clinically significant deterioration. Treatment with durvalumab + tremelimumab would continue between the initial assessment of progression and confirmation for progression.
- In addition, subjects may continue to receive durvalumab + tremelimumab beyond confirmed PD in the absence of clinically significant deterioration and if investigators consider that subjects continue to receive benefit from treatment.

Progression would be considered confirmed if the following criteria are met:

- ≥20% increase in the sum diameters of target lesions compared with the nadir at 2 consecutive visits with an absolute increase of 5 mm, and/or
- Clinically significant progression (worsening) of non-target lesions or new lesions at the confirmatory PD time point compared with the first time point where progression of non-target lesions or new lesions was identified, and/or
- Additional new unequivocal lesions at the confirmatory PD time point compared with the first time point at which new lesions were identified.

In the absence of clinically significant deterioration, the Investigator should continue study treatment until progression is confirmed.

If progression is not confirmed, then the patient should continue on study treatment and on treatment assessments.

Modification of RECIST as described may discourage the early discontinuation of durvalumab + tremelimumab and provide a more complete evaluation of its anti-tumor activity than would be seen with conventional response criteria. Nonetheless, the efficacy analysis will be conducted by programmatically deriving each efficacy endpoint based on RECIST 1.1 criteria.

Of note, clinically significant deterioration is considered to be a rapid tumor progression that necessitates treatment with anti-cancer therapy other than durvalumab + tremelimumab or with symptomatic progression that requires urgent medical intervention (e.g., central nervous system metastasis, respiratory failure due to tumor compression, spinal cord compression).

9.1 Efficacy variable

The RECIST 1.1 criteria will be used to assess patient response to treatment by determining progression-free survival rates at 6, 9 and 12 months, median progression-free survival, overall response rate and median overall survival using Investigator assessments. The management of patients will be based in part upon the results of the RECIST 1.1 assessment conducted by the Investigator.

10. ASSESSMENT OF SAFETY

The Principal Investigator is responsible for ensuring that all staff involved in the study is familiar with the content of this section.

10.1 Safety Parameters

10.1.1 Definition of adverse events

The International Conference on Harmonization (ICH) Guideline for Good Clinical Practice (GCP) E6(R1) defines an AE as:

Any untoward medical occurrence in a patient or clinical investigation subject administered a pharmaceutical product and which does not necessarily have a causal relationship with this treatment. An AE can therefore be any unfavorable and unintended sign (including an abnormal laboratory finding), symptom, or disease temporally associated with the use of a medicinal product, whether or not considered related to the medicinal product.

An AE includes but is not limited to any clinically significant worsening of a subject's pre-existing condition. An abnormal laboratory finding (including ECG finding) that requires an action or intervention by the investigator, or a finding judged by the investigator to represent a change beyond the range of normal physiologic fluctuation, should be reported as an AE.

Adverse events may be treatment emergent (ie, occurring after initial receipt of investigational product) or non treatment emergent. A non treatment-emergent AE is any new sign or symptom, disease, or other untoward medical event that begins after written informed consent has been

obtained but before the subject has received investigational product.

Elective treatment or surgery or pre planned treatment or surgery (that was scheduled prior to the subject being enrolled into the study) for a documented pre-existing condition, that did not worsen from baseline, is not considered an AE (serious or non serious). An untoward medical event occurring during the pre scheduled elective procedure or routinely scheduled treatment should be recorded as an AE or SAE.

The term AE is used to include both serious and non-serious AEs.

10.1.2 Definition of serious adverse events

A serious adverse event is an AE occurring during any study phase (i.e., screening, run-in, treatment, wash-out, follow-up), at any dose of the study drugs that fulfils one or more of the following criteria:

1- Results in death

2- Is immediately life-threatening

3- Requires inpatient hospitalization or prolongation of existing hospitalization

4- Results in persistent or significant disability or incapacity

5- Is a congenital abnormality or birth defect in offspring of the subject

6- Is an important medical event that may jeopardize the patient or may require medical intervention to prevent one of the outcomes listed above:

7- Medical or scientific judgment should be exercised in deciding whether expedited reporting is appropriate in this situation. Examples of medically important events are intensive treatment in an emergency room or at home for allergic bronchospasm, blood dyscrasias, or convulsions that do not result in hospitalizations; or development of drug dependency or drug abuse.

The causality of SAEs (their relationship to all study treatment/procedures) will be assessed by the investigator(s) and communicated to the sponsor.

10.1.3 Durvalumab + tremelimumab adverse events of special interest

An adverse event of special interest (AESI) is one of scientific and medical interest specific to understanding of the Investigational Product and may require close monitoring and rapid communication by the investigator to the sponsor. An AESI may be serious or non-serious. The rapid reporting of AESIs allows ongoing surveillance of these events in order to characterize and understand them in association with the use of this investigational product. A report is required using the SAE form, indicating an event of special interest.

AESIs for durvalumab and tremelimumab include but are not limited to events with a potential inflammatory or immune-mediated mechanism and which may require more frequent monitoring and/or interventions such as steroids, immunosuppressants and/or hormone replacement therapy. These AESIs are being closely monitored in clinical studies with durvalumab monotherapy and combination therapy. An immune-related adverse event (irAE) is defined as an adverse event that

is associated with drug exposure and is consistent with an immune-mediated mechanism of action and where there is no clear alternative aetiology. Serologic, immunologic, and histologic (biopsy) data, as appropriate, should be used to support an irAE diagnosis. Appropriate efforts should be made to rule out neoplastic, infectious, metabolic, toxin, or other etiologic causes of the irAE.

Events with an inflammatory or immune mediated mechanism could occur in nearly all organs. Potential risks with an immune-mediated aetiology that are rare or less frequent include, but are not limited to, Guillain-Barre Syndrome, myasthenia gravis, pericarditis, sarcoidosis, uveitis, and other events involving the eye (eg, keratitis and optic neuritis), skin (eg, scleroderma, vitiligo and pemphigoid), haematological (eg, haemolytic anaemia and immune thrombocytopenic purpura), rheumatological events (polymyalgia rheumatic and autoimmune arthritis), vasculitis, non-infectious meningitis and non-infectious encephalitis.

If the Investigator has any questions in regards to an adverse event (AE) being an irAE, the Investigator should promptly contact the Study Physician.

AESIs observed with durvalumab and tremelimumab include:

- Colitis
- Pneumonitis
- ALT/AST increases / hepatitis / hepatotoxicity
- Neuropathy / neuromuscular toxicity (i.e. events of encephalitis, peripheral motor and sensory neuropathies, Guillain-Barré, and myasthenia gravis)
- Endocrinopathy (i.e. events of hypophysitis, adrenal insufficiency, and hyper- and hypothyroidism)
- Dermatitis
- Nephritis
- Pemphigoid
- Pancreatitis (or labs suggestive of pancreatitis - increased serum lipase , increased serum amylase)
- Other inflammatory responses that are rare with a potential immune-mediated aetiology include, but are not limited to, myocarditis, pericarditis, and uveitis.

Further information on these risks (e.g. presenting symptoms) can be found in the current version of the durvalumab and tremelimumab Investigator Brochure.

Guidelines for the management of patients with immune-related AEs (irAEs) are provided in Appendix 1. In addition to the dose modification guidelines provided in Appendix 1, it is recommended that irAEs are managed according to the general treatment guidelines outlined for ipilimumab (Weber et al, 2012). These guidelines recommend the following:

- Patients should be evaluated to identify any alternative etiology.
- In the absence of a clear alternative etiology, all events of an inflammatory nature should be

considered immune related.

- Symptomatic and topical therapy should be considered for low-grade events.
- Systemic corticosteroids should be considered for a persistent low-grade event or for a severe event.
- More potent immunosuppressives should be considered for events not responding to systemic steroids (eg, infliximab or mycophenolate).

If the Investigator has any questions in regards to an AE being an irAE, the Investigator should immediately contact the Study Physician.

Infusion reactions

AEs of infusion reactions (also termed infusion-related reactions) are of special interest to AstraZeneca and are defined, for the purpose of this protocol, as all AEs occurring from the start of IP infusion up to 48 hours after the infusion start time. For all infusion reactions, SAEs should be reported to the sponsor as described in Section 10.3.

Hypersensitivity reactions

Hypersensitivity reactions as well as infusion-related reactions have been reported with anti-PD-L1 and anti-PD-1 therapy (Brahmer et al 2012). As with the administration of any foreign protein and/or other biologic agents, reactions following the infusion of mAbs can be caused by various mechanisms, including acute anaphylactic (IgE-mediated) and anaphylactoid reactions against the mAbs and serum sickness. Acute allergic reactions may occur, may be severe, and may result in death. Acute allergic reactions may include hypotension, dyspnea, cyanosis, respiratory failure, urticaria, pruritus, angioedema, hypotonia, arthralgia, bronchospasm, wheeze, cough, dizziness, fatigue, headache, hypertension, myalgia, vomiting, and unresponsiveness. Guidelines for the management of patients with hypersensitivity (including anaphylactic reaction) and infusion-related reactions are provided in Appendix 1.

Hepatic function abnormalities (hepatotoxicity)

Hepatic function abnormality is defined as any increase in ALT or AST to greater than 3 × ULN and concurrent increase in total bilirubin to be greater than 2 × ULN. Concurrent findings are those that derive from a single blood draw or from separate blood draws taken within 8 days of each other. Follow-up investigations and inquiries will be initiated promptly by the investigational site to determine whether the findings are reproducible and/or whether there is objective evidence that clearly supports causation by a disease (eg, cholelithiasis and bile duct obstruction with distended gallbladder) or an agent other than the IP. Guidelines for management of patients with hepatic function abnormality are provided in section 10.3.3.2.

For the consultation of the complete list of adverse events see the last version of IB provided in the ISF.

10.2 Assessment of safety parameters

10.2.1 Assessment of severity

Assessment of severity is one of the responsibilities of the investigator in the evaluation of AEs and

SAEs. Severity will be graded according to the NCI CTCAE v4.03.

The determination of severity for all other events not listed in the CTCAE should be made by the investigator based upon medical judgment and the severity categories of Grade 1 to 5 as defined below:

Grade 1 (mild): An event that is usually transient and may require only minimal treatment or therapeutic intervention. The event does not generally interfere with usual activities of daily living.

Grade 2 (moderate): An event that is usually alleviated with additional specific therapeutic intervention. The event interferes with usual activities of daily living, causing discomfort but poses no significant or permanent risk of harm to the subject.

Grade 3 (severe): An event that requires intensive therapeutic intervention. The event interrupts usual activities of daily living, or significantly affects the clinical status of the subject.

Grade 4 (life threatening): An event, and/or its immediate sequelae, that is associated with an imminent risk of death or with physical or mental disabilities that affect or limit the ability of the subject to perform activities of daily living (eating, ambulation, toileting, etc).

Grade 5 (fatal): Death (loss of life) as a result of an event.

It is important to distinguish between serious criteria and severity of an AE. Severity is a measure of intensity whereas seriousness is defined by the criteria in Section 9.2.1. A Grade 3 AE need not necessarily be considered an SAE. For example, a Grade 3 headache that persists for several hours may not meet the regulatory definition of an SAE and would be considered a non serious event, whereas a Grade 2 seizure resulting in a hospital admission would be considered an SAE.

10.2.2 Assessment of relationship

Relationship of adverse events with study drugs will be assessed by investigators based on toxicity profile described for each study drug (see investigator brochure).

10.3 Recording of adverse events and serious adverse events

Adverse events will be recorded in the eCRF using a recognized medical term or diagnosis that accurately reflects the event. Adverse events will be assessed by the investigator for severity, relationship to the investigational product, possible etiologies, and whether the event meets criteria of an SAE and therefore requires immediate notification and not later than 24 h, to the Sponsor. The following variables will be collected for each AE:

- AE (verbatim)
- The date when the AE started and stopped
- Changes in NCI CTCAE grade and the maximum CTC grade attained
- Whether the AE is serious or not
- Investigator causality rating against durvalumab or tremelimumab (yes or no)
- Action taken with regard to durvalumab + tremelimumab

- Outcome

In addition, the following variables will be collected for SAEs as applicable:

- Date AE met criteria for serious AE
- Date Investigator became aware of serious AE
- AE is serious due to criteria
- Date of hospitalization
- Date of discharge
- Probable cause of death
- Date of death
- Autopsy performed
- Description of AE
- Causality assessment in relation to Study procedure(s)

Events, which are unequivocally due to disease progression, should not be reported as an AE during the study.

10.3.1 Study recording period and follow-up for adverse events and serious adverse events

Adverse events and serious adverse events will be recorded from time of signature of informed consent, throughout the treatment period and including the follow-up period (90 days after the last dose of durvalumab + tremelimumab).

During the course of the study all AEs and SAEs should be proactively followed up for each subject. Every effort should be made to obtain a resolution for all events, even if the events continue after discontinuation/study completion.

If a subject discontinues from treatment for reasons other than disease progression, and therefore continues to have tumor assessments, drug or procedure-related SAEs must be captured until the patient is considered to have confirmed PD and will have no further tumor assessments.

The investigator is responsible for following all SAEs until resolution, until the subject returns to baseline status, or until the condition has stabilized with the expectation that it will remain chronic, even if this extends beyond study participation.

Follow-up of unresolved adverse events

Any AEs that are unresolved at the subject's last visit in the study are followed up by the investigator for as long as medically indicated, but without further recording in the eCRF. After 90 days, only subjects with ongoing investigational product-related SAEs will continue to be followed for safety.

The sponsor and AstraZeneca/MedImmune retain the right to request additional information for any

subject with ongoing AE(s)/SAE(s) at the end of the study, if judged necessary.

Post study events

After the subject has been permanently withdrawn from the study, there is no obligation for the investigator to actively report information on new AE or SAEs occurring in former study subjects after the 90-day safety follow-up period for patients treated with durvalumab + tremelimumab. However, if an investigator learns of any SAEs, including death, at any time after the subject has been permanently withdrawn from study, and he/she considers there is a reasonable possibility that the event is related to study treatment,the investigator should notify the study sponsor, who will inform AstraZeneca/MedImmune Drug Safety.

10.3.2 Reporting of serious adverse events

All SAEs will be reported, whether or not considered causally related to the investigational product, or to the study procedure(s).. Sponsor is responsible for informing the Health Authorities of the SAE as per local requirements.

After the initial notification of an AE or SAE, the investigator is required to follow up each case and obtain more data on the patient's state. All the AEs and SAEs documented in previous visits must be reviewed in subsequent visits. All AEs and SAEs must be monitored until their resolution, stabilization, explanation of the event by another cause, or until the follow-up period ends. This is applicable to all the patients, including those who withdraw early.

The investigator will include in the follow-up any additional research that may clarify the nature and/or causality of the AE or SAE. This might include additional laboratory tests or studies, histopathologic examinations, or the consultation of other healthcare professionals. Any co-investigator can ask the investigator to carry out additional evaluations to definitively clarify the nature and/or the causality of AEs or SAEs. If the patient dies while participating in the study or during a follow-up period agreed upon mutually, the investigator will provide a copy of any postmortem finding requested, including histopathology.

The sponsor is responsible for notifying all serious or unexpected AEs potentially related to the treatments being investigated that occur during the study to the AEMPS, competent bodies of the autonomous communities where the clinical trial is being conducted within the time frame established by Spanish law and will concurrently forward all such reports to AstraZeneca. A copy of the SAE report must be sent by e-mail to AstraZeneca at the time the event is reported to the Health Authorities **(AEMailboxClinicalTrialTCS@astrazeneca.com)**. It is the responsibility of the sponsor to compile all necessary information and ensure that the Health Authorities receives a report according to the reporting requirement timelines and to ensure that these reports are also submitted to AstraZeneca at the same time.

SAE report and accompanying cover page will be sent by way of email to

[redacted]

If a non-serious AE becomes serious, this and other relevant follow-up information must also be

provided to AstraZeneca and the Health Authorities.

Serious adverse events that do not require expedited reporting to the Health Authorities still need to be reported to AstraZeneca preferably using the MedDRA coding language for serious adverse events. This information should be reported on a monthly basis and under no circumstance less frequently than quarterly.

10.3.2.1 Reporting of deaths

All deaths that occur during the study, or within the protocol-defined 90-day post-last dose of durvalumab + tremelimumab safety follow-up period must be reported as follows:

- Death that is clearly the result of disease progression should be documented but should not be reported as an SAE.
- Where death is not due (or not clearly due) to progression of the disease under study, the AE causing the death must be reported to as a SAE within **24 hours** (see Section 10.3.2 for further details). The report should contain a comment regarding the co-involvement of progression of disease, if appropriate, and should assign main and contributory causes of death.
- Deaths with an unknown cause should always be reported as a SAE.

Deaths that occur following the protocol-defined 90-day post-last-dose of durvalumab safety follow-up period will be documented as events for survival analysis, but will not be reported as an SAE.

10.3.3 Other events requiring reporting

10.3.3.1 Overdose

An overdose is defined as a subject receiving a dose of durvalumab + tremelimumab in excess of that specified in the Investigator's Brochure, unless otherwise specified in this protocol.
Any overdose of a study subject with durvalumab + tremelimumab, with or without associated AEs/SAEs, is required to be reported within 24 hours of knowledge of the event to the sponsor ,who will send a copy to AstraZeneca/MedImmune Patient Safety, or designed using the designated Safety e-mailbox (see Section 10.3.2 for contact information). If the overdose results in an AE, the AE must also be recorded as an AE (see Section 10.3). Overdose does not automatically make an AE serious, but if the consequences of the overdose are serious, for example death or hospitalization, the event is serious and must be recorded and reported as an SAE (see Section 10.3 and Section 10.3.2). There is currently no specific treatment in the event of an overdose of durvalumab or tremelimumab.

The investigator will use clinical judgment to treat any overdose.

10.3.3.2 Hepatic function abnormality

Hepatic function abnormality (as defined in Section 10.1.3.) in a study subject, with or without associated clinical manifestations, is required to be reported as "hepatic function abnormal" ***within 24 hours of knowledge of the event*** to the sponsor ,who will send a copy to AstraZeneca/MedImmune Patient Safety, using the designated Safety e-mailbox (see Section 10.3.2 for contact information), unless a definitive underlying diagnosis for the abnormality (e.g.,

cholelithiasis or bile duct obstruction) that is unrelated to investigational product has been confirmed.

- If the definitive underlying diagnosis for the abnormality has been established and is unrelated to investigational product, the decision to continue dosing of the study subject will be based on the clinical judgment of the investigator.
- If no definitive underlying diagnosis for the abnormality is established, dosing of the study subject must be interrupted immediately. Follow-up investigations and inquiries must be initiated by the investigational site without delay.

Grade 1-2 events should be considered AEs, not SAEs. However, Grade 1-2 events should be reported as SAEs if requires hospitalization or if they are medically important events based on the investigator judgement.

Grade 3 events, should be reported as SAE if requires hospitalization or if they are medically important events based on the investigator judgement.

All grade 4 events should be reported as SAEs.

Cases where a patient shows elevations in liver biochemistry may require further evaluation and occurrences of AST or ALT ≥3 × ULN together with total bilirubin ≥2 × ULN may need to be reported as SAEs.
Each reported event of hepatic function abnormality will be followed by the investigator and evaluated by the sponsor.

10.3.3.3 Pregnancy

a) Maternal exposure

If a patient becomes pregnant during the course of the study, the IPs should be discontinued immediately.
Pregnancy itself is not regarded as an AE unless there is a suspicion that the IP under study may have interfered with the effectiveness of a contraceptive medication. Congenital abnormalities or birth defects and spontaneous miscarriages should be reported and handled as SAEs. Elective abortions without complications should not be handled as Aes. The outcome of all pregnancies (spontaneous miscarriage, elective termination, ectopic pregnancy, normal birth, or congenital abnormality) should be followed up and documented even if the patient was discontinued from the study.
If any pregnancy occurs in the course of the study, then the Investigator or other site personnel should inform the appropriate sponsor representatives within 1 day, ie, immediately, but **no later than 24 hours** of when he or she becomes aware of it.
The designated sponsor representative will work with the Investigator to ensure that all relevant information is provided to the sponsor, who will inform AstraZeneca Patient Safety data entry site.
The same timelines apply when outcome information is available.

b) Paternal exposure

Male patients should refrain from fathering a child or donating sperm during the study and for 180 days after the last dose of durvalumab + tremelimumab combination therapy or 90 days after the last dose of durvalumab monotherapy, whichever is the longer time period.

Pregnancy of the patient's partner is not considered to be an AE. However, the outcome of all pregnancies (spontaneous miscarriage, elective termination, ectopic pregnancy, normal birth, or congenital abnormality) occurring from the date of the first dose until 90 days after the last dose should, if possible, be followed up and documented.

Where a report of pregnancy is received, prior to obtaining information about the pregnancy, the Investigator must obtain the consent of the patient's partner. Therefore, the local study team should adopt the generic ICF template in line with local procedures and submit it to the relevant Ethics Committees (ECs) prior to use.

11. STATISTICAL METHODS AND SAMPLE SIZE DETERMINATION

11.1. Description of analysis sets

The statistical analyses described in this section will be performed as further outlined in the Statistical Analysis Plan, which will be included in the clinical study report for this protocol.

Analysis Sets

The analysis sets will be defined as follows:

- **Full Analysis Set** will include all allocated subjects. This will be primary analysis set for the efficacy endpoints.
- **Per Protocol Analysis Set** will include those subjects who were allocated and received at least one dose of the assigned study drug and had no major protocol deviations. The subjects will complete both baseline and at least one post-baseline tumor assessments (week 12).
- S**afety Analysis Set** will include all subjects who were allocated and received at least one dose of the study drug and had at least one post-baseline safety evaluation (week 12). This will be the analysis set for all safety evaluations.
- **Pharmacodynamic Analysis Set**: All the subjects who have received at least one dose of study drug and have evaluable pharmacodynamic data.

Demographic and other baseline characteristics

Demographic and other baseline characteristics will be summarized and listed. For continuous demographic/baseline variables including age, weight, and vital signs, results will be summarized and presented as n, number of not available data (NA), mean, standard deviation, median, and minimum and maximum values. For categorical variables such as race/ethnicity, the number and percentage of subjects will be used.

Prior and concomitant medications

Concomitant medications will be assigned an 11-digit code using the World Health Organization Drug Dictionary (WHO DD) drug codes. Concomitant medications will be further coded to the appropriate Anatomical-Therapeutic-Chemical (ATC) code indicating therapeutic classification. Prior and concomitant medications will be summarized and listed by drug and drug class.

Efficacy Analyses

All efficacy analyses will be based primarily on the Full Analysis Set and secondarily on the Per Protocol Analysis Set.

Data cut-off for the primary study analysis will happen following after the last patient included in the study has performed the first tumor evaluation (week 12 after first dose of study drug).The following tumor evaluations will take place every 12 weeks until documentation of disease progression or start of another anticancer therapy.

Analysis of primary efficacy variable

The analysis of clinical benefit rate and overall survival rate will be performed independently for each study cohort when the last patient included in the corresponding cohort of the study will arrived to 9 months after inclusion in the study or have progressed. The primary objective of the study will be based on the investigator assessment.

11.2. Methods of statistical analyses

Strategic decisions about future development of immune therapy in neuroendocrine tumors will be available after the results of this trial.

Summary tables (descriptive statistics and frequency tables) will be provided for all baseline variables, efficacy variables, and safety variables, as appropriate. Continuous variables will be summarized with descriptive statistics (mean, standard deviation, range, and median). Ninety-five (95) percent confidence intervals (95% CI) may also be presented, as appropriate. Frequency counts and percentage of subjects within each category will be provided for categorical data.

The primary efficacy analysis will be performed using the binomial test procedure. Missing data will be treated using statistical multiple random imputation.

Secondary endpoints will be summarized with descriptive statistics. Continuous variables will be summarized with n, mean, standard deviation, and range, frequency counts and percentage of subjects within each category will be provided for categorical data. Multivariate regression models will be used to study relations between explanatory variables and primary endpoint. Survival analysis will be performed to analyze PFS, Kaplan-Meier curves will be presented and possible comparisons will be tested using the log-rank test or the Cox proportional hazard model for multivariate analysis, hazard ratios (HR) and their 95% confidence interval (CI95%) will be provided. Patients with lost of follow-up or treatment discontinuation will be included in the final analysis of primary endpoint if they have at least one tumor evaluation and considered as censored data for survival endpoints.

R software version 3.2.1 will be used for all analysis.

Safety Analyses

Safety analyses will be based on the Safety Analysis Set. All safety analyses will be summarized separately by cohort. Adverse events and serious adverse events, laboratory test results, physical examination findings and vital signs, and their changes from baseline will be summarized using descriptive statistics. Abnormal values will be flagged.

11.3. Determination of sample size

Sample size has been calculated using one-sample Superiority test (function One Sample Proportion. NIS of the Trial Size package of R software). According to previous knowledge, it is estimated that the reference value for the likelihood to be progression-free at 9 months is 30% and we expect an increase of 20% with a superiority margin of 10%. With a unilateral alpha level of 5% and 80% power, we estimate to include 28 patients per group, with an expected lost to follow-up rate of 10%, a final sample size in each 1, 2 and 3 cohort will include 31 patients.

For cohort 4, and according to previous knowledge, it is estimated that the reference proportion of

patients being alive at 9 months is 13% and we expect an increase of 10% with a superiority margin of 5%. With a unilateral alpha level of 5% and 80% power, we estimate to include 30 patients per group, with an expected lost to follow-up rate of 10%, a final sample size will include 33 patients. Summarizing, total sample size will include 126 patients: 31 patients for cohorts 1 to 3, and 33 patients for cohort 4.

12. ETHICAL AND REGULATORY REQUIREMENTS

12.1. Ethical conduct of the study

The study will be performed in accordance with ethical principles that have their origin in the Declaration of Helsinki and are consistent with ICH/Good Clinical Practice, and applicable regulatory requirements including those related to Subject data protection.

12.2. Ethics and regulatory review

The protocol, ICF, and appropriate related documents must be reviewed and approved by an EC constituted and functioning in accordance with ICH E6, Section 3, and any local regulations,. Any protocol amendment and/or revision to the ICF will be resubmitted to the EC for review and approval, except for changes involving only logistical or administrative aspects of the study (e.g., change in CRA[s] or change of telephone number[s]). Documentation of EC compliance with ICH and any local regulations regarding constitution and review conduct will be provided to the sponsor.

A signed letter of study approval from the EC Chairman must be obtained prior to study start and the release of any study drug to the site by the sponsor or its designee. If the EC decides to suspend or terminate the study, the sponsor will immediately send the notice of study suspension or termination by the EC to the investigators. Study progress is to be reported to the EC annually (or as required) by the sponsor. If the investigator is required to report to the EC, he/she will forward a copy to the sponsor at the time of each periodic report. The sponsor will submit periodic reports and inform the EC of any reportable adverse events according to local legislation. Upon completion of the study, the investigator will provide the EC with a brief report of the outcome of the study, if required.

12.3. Informed consent

As part of administering the informed consent document, the investigator must explain to each subject (or guardian/legally authorized representative) the nature of the study, its purpose, the procedures involved, the expected duration, the potential risks and benefits involved, and any potential discomfort. Each subject must be informed that participation in the study is voluntary and that he/she may withdraw from the study at any time and that withdrawal of consent will not affect his/her subsequent medical treatment or relationship with the treating physician. This informed consent should be given by means of a standard written statement, written in non-technical language. The subject should understand the statement before signing and dating it and will be given a copy of the signed document. If a subject is unable to read or if a legally acceptable representative is unable to read, an impartial witness should be present during the entire informed consent discussion. After the written informed consent form and any other written information to be provided to subjects is read and explained to the subject or the subject's legally acceptable representative, and after the subject or the subject's legally acceptable representative has orally

consented to the subject's participation in the trial and, if capable of doing so, has signed and personally dated the ICF, the witness should sign and personally date the consent form. The subject will be asked to sign an informed consent at the Screening Visit prior to any study-specific procedures being performed. No subject can enter the study before his/her informed consent has been obtained. The form must be signed and dated by the appropriate parties. The original, signed ICF for each subject will be verified by the sponsor and kept on file, according to local procedure, at the study center.

The subject or the subject's legally authorized representative should be informed in a timely manner if new information becomes available that may be relevant to the subject's willingness to continue participation in the trial. The communication of this information should be documented.

12.4. Changes to the protocol and informed consent form

There are to be no changes to the protocol without written approval from the sponsor. Protocols will be followed as written. Any change to the protocol requires a written protocol amendment or administrative change that must be approved by the sponsor before implementation. Amendments affecting the safety of subjects, the scope of the investigation, or the scientific quality of the study require submission to Health Authorities as well as additional approval by the EC. These requirements should in no way prevent any immediate action from being taken by the investigator, or by the sponsor, in the interest of preserving the safety of all subjects included in the study. If an immediate change to the protocol is felt by the investigator to be necessary for safety reasons, the sponsor's appropriate study team member must be notified promptly and the EC for the site must be informed immediately. A protocol change intended to eliminate an immediate hazard may be implemented immediately, provided that the Health Authorities and EC are subsequently notified by protocol amendment.

Changes affecting only administrative aspects of the study do not require formal protocol amendments or EC approval, but the EC (if regionally required, the heads of the medical institutions) must be kept informed of such changes. In these cases, the sponsor will send a letter to the EC (if regionally required, the heads of the medical institutions) detailing such changes.

12.5. Audits and inspections

In addition to routine monitoring procedures, the sponsor's Clinical Quality Assurance department conducts audits of clinical research activities in accordance with the sponsor's Standard Operating Procedures (SOPs) to evaluate compliance with the principles of ICH GCP and all applicable local regulations. A government regulatory authority may also wish to conduct an inspection (during the study or after its completion). If an inspection is requested by a regulatory authority, the investigator must inform the sponsor immediately that this request has been made.

12.6. Confidentiality

The contents of this protocol and any amendments and results obtained during the course of this study will be kept confidential by the investigator, the investigator's staff, and the EC and will not be disclosed in whole or in part to others or used for any purpose other than reviewing or performing the study without the written consent of the sponsor. No data collected as part of this study will be utilized in any written work, including publications, without the written consent of the sponsor. These obligations of confidentiality and non-use shall in no way diminish such obligations as set

forth in the Confidentiality Agreement between the sponsor and the investigator.

All persons assisting in the performance of this study must be bound by the obligations of confidentiality and non-use set forth in the Confidentiality Agreement between the investigator and sponsor (provided by the sponsor).

All laboratory specimens, evaluation forms, reports, and other records will be identified in a manner designed to maintain subject confidentiality. Each patient will be given a Unique Patient Number (UPN) for this study, provided by the Sponsor. The investigator will custody a log matching the UPN with personal data of patients. All data will be recorded i the appropriate CRFs using this identification number. This number will be provided to the central laboratory to ensure traceability of study samples. Each Investigator will ensure that all site personnel involved will respect the confidentiality of any information about trial subjects. Management of personal data from subjects participating in the trial, particularly as regards consent, will comply with European Directive on Data Privacy (95/46/EC) and the Spanish Organic Law 15/1999 of 13th December on the protection of personal data.

At each site, all records will be kept in a secure storage area with limited access. Clinical information will not be released without the written permission of the subject. Subject identity is confidential and may only be known by the Investigator, trial personnel, appointed auditors and monitors, and Health Authorities.

Each Investigator and all employees and coworkers involved with this trial may not disclose or use for any purpose other than performance of the trial, any data, record, or other unpublished, confidential information disclosed to those individuals for the purpose of the trial. Prior written agreement from the sponsor or its designee must be collected for the disclosure of any said confidential information to other parties.

12.7. Insurance

The sponsor has contracted an insurance policy to cover the responsibilities of the investigator and other parties participating in the study, according to the applicable Spanish legislation.

Insurance company: **CHUBB Insurance Company of Europe SE.**

Policy Number: **9950243799502437**

12.8. Publications

The sponsor commits to responsible publication of both the positive and negative results from its clinical trials as required by all governing regulatory and health authorities.

Investigators will not publish the global study results (all sites) unless the sponsor has not done so in a suitable time period after the clinical study report (CSR) has been available. Should the Investigator(s) independently seek to publish results of this study which occur at their study site(s), they must inform the study sponsor of any/all drafts (including, but not limited to papers, manuscripts or abstracts) at least 60 days before submission to the congress, meeting or journal. The sponsor and Investigator(s) will agree with all aspects related to any proposed publications with regards to the following: 1) any proposed publications will be drafted in agreement with international recommendations, such as those from the International Committee of Medical Journal Editors (ICMJE) and all elements of the Consort Statement (2010), to maintain integrity of the trial

results in all communications; 2) any proposed publications will state the Clinical Research Ethics Committees which approved the trial and the funding sources of the trial; 3) any proposed publications will occur before disclosure of results to lay people; 4) any proposed publications will not report premature or partial data prior to completion of the analysis of the overall results of the trial.

13. STUDY MANAGEMENT

13.1. Training of study site personnel

The principal investigator will maintain a record of all center staff involved in the clinical trial (doctors, nurses and other staff involved) ensuring that they receive appropriate training to perform the study, and that any new information of relevance to the study will be transmitted to them.

Researchers will be instructed about the procedures of the trial in the investigator meeting and/or initiation visits made by monitors at each participating center prior to the study start.

13.2. Monitoring of the study

The sponsor's or representative (e.g., CRO's CRA) will maintain contact with the investigator and designated staff by telephone, and/or letter, and/or email between study visits. Monitoring visits to each investigational site will be conducted by the assigned CRA as described in the monitoring plan. The investigator (if regionally required, the heads of the medical institutions) will allow the CRA to inspect the clinical, laboratory, and pharmacy facilities to assure compliance with Good Clinical Practices and local regulatory requirements. The eCRFs and subject's corresponding original medical records (source documents) are to be fully available for review by the sponsor's representatives at regular intervals. These reviews verify adherence to the study protocol and data accuracy in accordance with federal regulations. All records at the investigational site, including source documents, are subject to inspection by the regulatory authorities and to review by the Ethical Committee.

13.3. Source data

In accordance with ICH E6, Section 6.10, source documents include but are not limited to the following:

- Clinic, office, hospital charts;
- Copies or transcribed healthcare provider notes which have been certified for accuracy after production;
- Recorded data from automated instruments such as x-rays, and other imaging reports: e.g., sonograms, CT scans, MRIs, nuclear medicine scans, ECGs, rhythm strips, electroencephalograms (EEGs), polysomnography, and pulmonary function tests (regardless of how these images are stored, including microfiche and photographic negatives);
- Records of telephone contacts;
- Drug distribution and accountability logs maintained in pharmacies or by research

personnel;

- Laboratory results and other laboratory test outputs: e.g., urine pregnancy test result documentation;
- Correspondence regarding a study subject's treatment between physicians or memoranda sent to the EC;

13.4. Retention of Records

The circumstances of completion or termination of the study notwithstanding, the investigator (if regionally required, the heads of the medical institutions) has the responsibility to retain all study documents, including but not limited to the protocol, the Investigator's Brochure, regulatory agency registration documents, ICFs, and EC correspondence. The investigational site should retain study documents until at least 25 years after the finalization of the study.

It is requested that at the completion of the required retention period or, should the investigator retire or relocate, the investigator contact the sponsor, allowing the sponsor the option of permanently retaining the study records.

13.5. Quality assurance

This study will be organized, performed, and reported in compliance with the protocol, standard operating practices (SOPs), working practice documents, and applicable regulations and guidelines. Site visit audits will be made periodically by the sponsor's or CRO's qualified compliance auditing team, which is an independent function from the study conduct team.

13.6. Study timetable and end of study

End of study is defined as Last Subject Last Visit.

- Recruitment period will be: 24 months
- Research Agreement executed: Nov 2016
- Projected IRB/IEC approval: Feb 2017
- First Subject In: Apr 2017
- 50% Enrollment: Apr 2018
- Last Subject In (100% enrollment): Nov 2019
- Last Subject Last Visit: Apr 2020
- Clinical Study Report Aug 2020

14. DATA MANAGEMENT

All software applications used in the collection and validation of data must be properly validated following standard computer system validation and must be compliant to all regulatory requirements.

The Data Management Plan (DMP) defines and documents the procedures necessary to ensure

data quality. These activities must be followed to ensure data are properly entered, validated, coded, integrated, reconciled and reviewed.

Data required by the protocol are collected on an electronic Case Report Form (eCRF) and entered into a validated data management system which is compliant to all regulatory requirements. As defined by ICH Guidelines, the Case Report Form (CRF) is a printed, optical, or electronic document designed to record all of the protocol required information to be reported to the sponsor on each trial subject'. In this study, CRF should refer to electronic data collection form. Data collected on the CRF must follow the instructions described in the CRF Completion Guidelines. The Investigator has ultimate responsibility for the collection and reporting of all clinical, safety and laboratory data entered on the CRF.

Any corrections to entries made on the CRF must be documented in a valid audit trail where the corrections must be dated, initialed, the reason for change stated, and original data not obscured. Only data required by the protocol for the purposes of the study should be collected.

The primary objective of the study will be clinical benefit rate at 9 months and survival rate at 9 months by investigator. Images of tumor evaluations will be anonymized correctly identified with trial number patient. 14.1. Study governance and oversight

The safety of all AstraZeneca products is closely monitored on an ongoing basis by AstraZeneca representatives in consultation with Patient Safety. Issues identified will be addressed; for instance, this could involve amendments to the study protocol and letters to Investigators.

15. INVESTIGATIONAL PRODUCT AND OTHER TREATMENTS

15.1. Identity of investigational product(s)

Table 7. List of investigational products for this study

Investigational product	Dosage form and strength	Manufacturer
Durvalumab	1500 mg, solution, IV	MedImmune
Tremelimumab	75 mg, solution, IV	MedImmune

16. LIST OF REFERENCES

Andorsky et al 2011

Andorsky DJ, Yamada RE, Said J, Pinkus GS, Betting DJ, and Timmerman JM. Programmed death ligand 1 is expressed by non-hodgkin lymphomas and inhibits the activity of tumor-associated T cells. Clin Cancer Res. 2011. 17(13):4232-4244

Antonia et al 2014b

Antonia S, Ou SI, Khleif SN, Brahmer J, Blake-Haskins A, Robbins PB, et al. Clinical activity and safety of MEDI4736, an anti-programmed cell death ligand-1 (PD-L1) antibody in patients with NSCLC. Poster presented at the European Society for Medical Oncology (ESMO) Meeting; 2014

Sep 26-30; Madrid, Spain.

Antonia et al 2014a

Antonia S, Goldberg S, Balmanoukian A, Narwal R, Robbins P, D'Angelo G, et al. A Phase 1 open-label study to evaluate the safety and tolerability of MEDI4736, an anti-programmed cell death-ligand 1 (PD-L1) antibody, in combination with tremelimumab in patients with advanced non-small cell lung cancer (NSCLC). Poster presented at European Society of Medical Oncology (ESMO) Meeting; 2014 Sep 26-30; Madrid, Spain.

Antonia et al 2015

Antonia et al. Phase ib study of medi4736 in combination with treme in patients with advanced NSCLC. Chicago, Il ASCO May30-June4,ASCO 2015, poster #3014.

Berry et al 1991

Berry G, Kitchin RM, Mock PA. A comparison of 2 simple hazard ratio estimators based on the log rank test. Stat Med 1991;10(5):749-55.

Bograd et al 2011

Bograd AJ, Suzuki K, Vertes E, Colovos C, Morales EA, Sadelain M, et al. Immune responses and immunotherapeutic interventions in malignant pleural mesothelioma. Cancer Immunol Immunother. 2011;60:1509-27.

Bonomi 2010

Bonomi PD. Implications of key trials in advanced non small cell lung cancer. Cancer 2010;116(5):1155-64.

Brahmer et al 2012

Brahmer JR, Tykodi SS, Chow LQM, Hwu WJ, Topalian SL, Hwu P, et al. Safety and activity of anti-PD-L1 antibody in patients with advanced cancer. N Engl J Med 2012;366(26):2455-65.

Brahmer et al 2014

Brahmer JR, Horn L, Gandhi L, Spigel DR, Antonia SJ, Rizvi NA et al. Nivolumab (anti-PD-1, BMS-936558, ONO-4538) in patients (pts) with advanced non-small-cell lung cancer (NSCLC): Survival and clinical activity by subgroup analysis[ASCO abstract 8112]. J Clin Oncol 2014;32(15)(Suppl).

Brusa et al 2013

Brusa D, Serra S, Coscia M, Rossi D, D'Arena G, Laurenti L, et al. The PD-1/PD-L1 axis contributes to T-cell dysfunction in chronic lymphocytic leukemia. Haematologica. 2013 Jun; 98(6):953-963.

Burt et al 2011

Burt BM, Rodig SJ, Tilleman TR, Elbardissi AW, Bueno R, Sugarbaker DJ. Circulating and tumor-infiltrating myeloid cells predict survival in human pleural mesothelioma. Cancer.

2011;117(22):5234-44.

Ciuleanu et al 2009

Ciuleanu T, Brodowicz T, Zielinski C, Kim JH, Krzakowski M, Laack E, et al. Maintenance pemetrexed plus best supportive care versus placebo plus best supportive care for non-small-cell lung cancer: a randomized, double-blind, phase 3 study. Lancet 2009;374(9699):1432-40.

Collett 2003

Collett D. Modelling survival data in medical research: 2nd ed. Chapman and Hall/CRC; 2003.

D'Addario et al 2010

D'Addario G, Fruh M, Reck M, Baumann P, Klepetko W, Felip E, et al. Metastatic non-small-cell lung cancer: ESMO clinical practice guidelines for diagnosis, treatment and follow-up. Ann Oncol 2010;21(Suppl 5):116-9.

Drake et al 2013

Drake CG, McDermott DF, Sznol M, Choueiri TK, Kluger HM, Powderly JD et al. Survival, safety, and response duration results of nivolumab (Anti-PD-1; BMS-936558; ONO-4538) in a phase I trial in patients with previously treated metastatic renal cell carcinoma (mRCC): Long-term patient follow-up[abstract 4514]. J Clin Oncol 2013;31(Suppl).

Dunn et al 2004

Dunn GP, Old LJ, Schreiber RD. The three Es of cancer immunoediting. Annu Rev Immunol 2004;22:329-60.

Ellis et al 2008

Ellis S, Carroll KJ, Pemberton K. Analysis of duration of response in oncology trials. Contemp Clin Trials 2008;29(4):456-65.

Forde et al 2014

Forde PM, Kelly RJ, Brahmer JR. New strategies in lung cancer: translating immunotherapy into clinical practice. Clin Cancer Res 2014;20(5):1067-73.

Gail and Simon 1985

Gail M, Simon R. Tests for qualitative interactions between treatment effects and patient subsets. Biometrics 1985;41(2):361–72.

GLOBOCAN 2012

GLOBOCAN. Lung cancer, estimated incidence, mortality and prevalence worldwide in 2012. International Agency for Research on Cancer, World Health Organization; Lyon, 2012.Available at http://globocan.iarc.fr/Pages/fact_sheets_cancer.aspx. Accessed on 16 April 2015.

Gong et al 2009

Gong AY, Zhou R, Hu G, Li X, Splinter PL, O'Hara SP, et al. MicroRNA-513 regulates B7-H1

translation and is involved in IFN-gamma-induced B7-H1 expression in cholangiocytes. J Immunol 2009;182(3):1325-33.

Hamanshi et al 2007

Hamanishi J, Mandai M, Iwasaki M, Okazaki T, Tanaka Y, Yamaguchi K, et al. Programmed cell death 1 ligand 1 and tumor-infiltrating CD8+ T lymphocytes are prognostic factors of human ovarian cancer. Proc Nat Acad Sci U S A. 2007;104(9):3360-5.

Herbst et al 2013

Herbst RS, Gordon MS, Fine GD, Sosman JA, Soria JC, Hamid O, et al. A study of MPDL3280A, an engineered PD-L1 antibody in patients with locally advanced or metastatic tumours [abstract 3000]. J Clin Oncol 2013;31(Suppl 15).

Hirano et al 2005

Hirano F, Kaneko K, Tamura H, Dong H, Wang S, Ichikawa M, et al. Blockade of B7-H1 and PD-1 by monoclonal antibodies potentiates cancer therapeutic immunity. Cancer Res 2005;65(3):1089-96.

Hodi et al 2010

Hodi FS, O'Day SJ, McDermott DF, Weber RW, Sosman JA, Haanan JB, et al. Improved survival with ipilimumab in patients with metastatic melanoma [published erratum appears in N Engl J Med 2010;363(13):1290]. N Engl J Med 2010;363(8):711-23.

Hodi et al 2014

Hodi FS, Sznol M, Kluger HM, McDermott DF, Carvajal RD, Lawrence DP et al. Long-term survival of ipilimumab-naive patients (pts) with advanced melanoma (MEL) treated with nivolumab (anti-PD-1, BMS-936558, ONO-4538) in a phase I trial [ASCO abstract 9002]. J Clin Oncol 2014; 32(15)(Suppl).

Howlander et al 2014

Howlander N, Noone AM, Krapcho M, Garshell J, Neyman N, Altekruse SF, et al. SEER cancer statistics review, 1975-2011. Bethesda (MD): National Cancer Institute,. Available from: URL:http://seer.cancer.gov/csr/1975_2011/based on November 2013 SEER data submission (posted to the SEER web site, April 2014).

IASLC Staging Manual in Thoracic Oncology

Goldstraw P, ed. Staging manual in thoracic oncology. 7th ed. IASLC 2010. (Available on request)

Iwai et al 2002

Iwai Y, Ishida M, Tanaka Y, Okazaki T, Honjo T, Minato N. Involvement of PD-L1 on tumour cells in the escape from host immune system and tumour immunotherapy by PD-L1 blockade.Proc Natl Acad Sci USA 2002;99(19):12293-7.

Keir et al 2008

Keir ME, Butte MJ, Freeman GJ, Sharpe AH. PD-1 and its ligands in tolerance and immunity. Annu

Rev Immunol. 2008;26:677-704.

Kirkwood et al 2010

Kirkwood JM, Lorigan P, Hersey P, Hauschild A, Robert C, McDermott D, et al. Phase II trial of tremelimumab (CP-675,206) in patients with advanced refractory or relapsed melanoma. Clin Cancer Res 2010;16(3):1042-8.

Kitano et al 2014

Kitano S, Postow MA, ZieglerCG, Kuk D, Panageas KS, Cortez C, et al. Computational algorithm-driven evaluation of monocytic myeloid-derived suppressor cell frequency for prediction of clinical outcomes. Cancer Immunol Res 2014;2(8):812-21.

Klein et al 2007

Klein JP, Logan B, Harhoff M, Andersen PK. Analyzing survival curves at a fixed point in time. Stat Med 2007;26(24):4505-19.

Korn et al 2008

Korn EL, Liu PY, Lee SJ, Chapman JA, Niedzwiecki D, Suman VJ, et al. Meta-analysis of phase II cooperative group trials in metastatic stage IV melanoma to determine progression-free and overall survival benchmarks for future phase II trials. J Clin Oncol 2008;26(4):527-34.

Krambeck et al 2007

Krambeck AE, Dong H, Thompson RH, Kuntz SM, Lohse CM, Leibovich BC, et al. Survivin and B7-H1 are collaborative predictors of survival and represent potential therapeutic targets for patients with renal cell carcinoma. Clin Cancer Res. 2007;13(6):1749- 56.

Kresowik and Griffith 2009

Kresowik TP, Griffith TS. Bacillus Calmette–Guerin immunotherapy for urothelial carcinoma of the bladder. Immunother. 2009;1(2):281-8.

Lan and DeMets 1983

Lan KKG, DeMets DL. Discrete sequential boundaries for clinical trials.Biometrika 1983;70(3):659-63.

Loos et al 2008

Loos M, Giese NA, Kleeff J, Giese T, Gaida MM, Bergmann F, et al. Clinical significance and regulation of the costimulatory molecule B7-H1 in pancreatic adenocarcinoma. Cancer Lett. 2008;268(1):98-109.

McDermott 2009

McDermott DF. Immunotherapy of metastatic renal cell carcinoma.Cancer. 2009;115(10 suppl):2298–305.

Meyer et al 2014

Meyer C, Cagnon L, Costa-Nunes Cm, Baumgaaertner P, Montandon N, Leyvraz L, et al. Frequencies of circulating MDSC correlate with clinical outcome of melanoma patients treated with ipilimumab. Cancer Immunol Immunother 2014;63(3):247-57.

Mu et al 2011

Mu CY, Huang JA, Chen Y, Chen C, Zhang XG. High expression of PD-L1 in lung cancer may contribute to poor prognosis and tumor cells immune escape through suppressing tumor infiltrating dendritic cells maturation. Med Oncol. 2011 Sep;28(3):682-8.

Nakano et al 2001

Nakano O, Sato M, Naito Y, Suzuki K, Orikasa S, Aizawa M, et al. Proliferative activity of intratumoral CD8+ T-lymphocytes as a prognostic factor in human renal cell carcinoma: clinicopathologic demonstration of antitumor immunity. Cancer Res. 2001;61:5132-6.

Narwal et al 2013

Narwal R, Roskos LK, Robbie GJ (2013) Population Pharmacokinetics of Sifalimumab, an Investigational Anti-Interferon alpha Monoclonal Antibody, in Systemic Lupus Erythematosus. Clin Pharmacokinet 52:1017–1027

Ng et al 2006

Ng CM, Lum BL, Gimenez V, et al. Rationale for fixed dosing of pertuzumab in cancer patients based on population pharmacokinetic analysis. Pharm Res. 2006;23(6):1275–84.

NCCN 2014

National Comprehensive Cancer Network Clinical Practice Guidelines in Oncology (NCCN Guidelines®).Non-Small Cell Lung Cancer. Version 4.2014. www.nccn.org.

Nishino et al 2013

Nishino M, Biobbie-Hurder A, Gargano M, Suda M, Ramaiya NH, Hodi FS. Developing a common language for tumor response to immunotherapy: immune-related response criteria using unidimensional measurements. Clin Cancer Res 2013;19(14):3936-43.

Nomi et al, 2007

Nomi T, Sho M, Akahori T, Hamada K, Kubo A, Kanehiro H, et al. Clinical significance and therapeutic potential of the programmed death-1 ligand/programmed death-1 pathway in human pancreatic adenocarcinoma. Cllin. Cancer Res.2007 Apri 1;13(7):2151-7.

Okudaira et al 2009

Okudaira K, Hokari R, Tsuzuki Y, Okada Y, Komoto S, Watanabe C, et al. Blockade of B7-H1 or B7-DC induces an anti-tumour effect in a mouse pancreatic cancer model. Int J Oncol 2009;35(4):741-9.

Pagès et al, 2010

Pagès F, Galon J, Dieu-Nosjean M.C., Tartour E., Sautès-Fridman C., Fridman W.H.2010. Immune infiltration in human tumors: a prognostic factor that should not be ignored. Oncogene.

29:1093-1102 10.1038/onc.2009.416

Pardoll 2012

Pardoll DM. The blockade of immune checkpoints in cancer immunotherapy.Nat Rev Cancer 2012;12(4):252-64.

Park et al, 2010

Park J, Omiya R, Matsumura Y, Sakoda Y, Kuramasu A, Augustine MM, et al. B7-H1/CD80 interaction is required for the induction and maintenance of peripheral T/cell tolerance. Blood.

Paz-Ares et al 2013

Paz-Ares LG, de Marinis F, Dediu M, Thomas M, Pujol JL, Bidoli P, et al. PARAMOUNT: Final overall survival results of the phase III study of maintenance pemetrexed versus placebo immediately after induction treatment with pemetrexed plus cisplatin for advanced nonsquamous non-small-cell lung cancer. J Clin Oncol 2013;31(23):2895-902.

Peggs et al 2009

Peggs KS, Quezada SA, Allison JP. Cancer immunotherapy: co-stimulatory agonists and coinhibitory antagonists. Clin Exp Immunol 2009;157:9-19.

Pisters and LeChevalier 2005

Pisters KM, LeChevalier T. Adjuvant chemotherapy in completely resected non-small-cell lung cancer. J Clin Oncol 2005;23(14):3270-8.

Raymond et al 2011

Raymond E, Dahan L, Raoul JL, Bang YJ, Borbath I, Lombard-Bohas C et al. Sunitinib malate for the treatment of pancreatic neuroendocrine tumors. N Engl J Med. 2011 Feb 10, 364(6): 501-13. doi:10.1056/NEJMoa1003825.

Reck et al 2014

Reck M, Popat S, Reinmuth N, De Ruysscher D, Kerr KM, Peters S; ESMO Guidelines Working Group. Metastatic non-small-cell lung cancer (NSCLC): ESMO clinical practice guidelines for diagnosis, treatment and follow-up. Ann Oncol 2014;25(Suppl 3):iii27-39.

Ribas et al 2013

Ribas A, Kefford R, Marshall MA, Punt CJA, Haanen JB, Marmol M, et al. Phase III randomized clinical trial comparing tremelimumab with standard-of-care chemotherapy in patients with advanced melanoma. J Clin Oncol 2013;31:616-22.

Robert et al, 2011

Robert C, Thomas L, Bondarenko I, Steven O'Day S, Weber J, Garbe C. Ipilimumab plus Dacarbazine for Previously Untreated Metastatic Melanoma. N Engl J Med 2011; 364:2517-2526.

Sandler et al 2006

Sandler A, Gray R, Perry MC, Brahmer J, Schiller JH, Dowlati A, et al. Paclitaxel-carboplatin alone or with bevacizumab for non-small-cell lung cancer. N Engl J Med 2006;355(24):2524-50.

Scagliotti et al 2008

Scagliotti GV, Parikh P, von Pawel J, Biesma B, Vansteenkiste J, Manegold C, et al. Phase III study comparing cisplatin plus gemcitabine with cisplatin plus pemetrexed in chemotherapy-naive patients with advanced-stage non-small-cell lung cancer. J Clin Oncol 2008;26(21)3543-51.

Schadendorf et al 2013

Schadendorf D, Hodi FS, Robert C et al. Pooled analysis of long-term survival data from phase II and phase III trials of ipilimumab in metastatic or locally advanced, unresectable melanoma. Presented at: European Cancer Congress 2013 (ECCO-ESMO-ESTRO); September 27-October 1, 2013; Amsterdam, The Netherlands. Abstract 24.

Schiller et al 2002

Schiller JH, Harringdon D, Belani CP, Langer C, Sandler A, Krook J, et al. Comparison of four chemotherapy regimens for advanced non-small-cell lung cancer. N Engl J Med 2002;10(346):92-8.

Schwarzenbach et al 2014

Schwarzenbach H, Nishida N, Calin GA, Pantel K. Clinical evidence of circulating cell-free microRNAs in cancer. Nat Rev Clin Oncol 2014;11(3):145-56.

Selke and Siegmund 1983

Selke T, Siegmund D. Sequential analysis of the proportional hazards model. Biometrika 1983;70:315-26.

Sun and Chen 2010

Sun X, Chen C. Comparison of Finkelstein's Method with the conventional approach for interval-censored data analysis. Stat Biopharm Res 2010;2(1):97-108.

Suzuki et al, 2011

Suzuki K, Kachala SS, Kadota K, Shen R, Mo Q, Beer DG, et al. Prognostic immune markers in non-small cell lung cancer. Clin Cancer res. 2011;17(16):5247-56. doi:10.1158/1078-0432.CCR-10-2805.

Tarhini and Kirkwood 2008

Tarhini AA, Kirkwood JM. Tremelimumab (CP-675,206): a fully human anticytotoxic T lymphocyte-associated antigen 4 monoclonal antibody for treatment of patients with advanced cancers. Expert Opin Biol Ther 2008;8(10):1583-93.

Topalian et al 2012

Topalian SL, Hodi FS, Brahmer JR, Gettinger SN, Smith DC, McDermott DF, et al. Safety, activity, and immune correlates of anti-PD-1 antibody in cancer. N Engl J Med 2012;366(26):2443-54.

Topalian et al 2014

Topalian SL, Sznol M, McDermott DF, Kluger HM, Carvajal RD, Sharfman WH et al. Survival, durable tumor remission, and long-term safety in patients with advanced melanoma receiving nivolumab. J Clin Oncol, 2014 Apr 1; 32(10):1020-30.

Wang et al 2009

Wang DD, Zhang S, Zhao H, et al. Fixed dosing versus body size based dosing of monoclonal antibodies in adult clinical trials. J Clin Pharmacol. 2009;49(9):1012–24.

Wang et al 2013

Wang Z, Han J, Cui Y, Fan K, Zhou X. Circulating microRNA-21 as noninvasive predictive biomarker for response in cancer immunotherapy. Med Hypotheses 2013;81(1):41-3.

Wang et al 2014

Wang E, Kang D, et al. Population pharmacokinetic and pharmacodynamics analysis of tremelimumab in patients with metastatic melanoma. J Clin Pharmacol. 2014 Oct; 54(10):1108-16.

Weber et al 2012

Weber J, Kähler KC, Hauschild A. Management of immune-related adverse events and kinetics of response with ipilimumab. J Clin Oncol 2012;30(21):2691-7.

Whitehead and Whitehead 1991

Whitehead A, Whitehead J. A general parametric approach to the meta-analysis of randomized clinical trials. Stat Med 1991;10(11):1665-77.

Wolchok et al 2009

Wolchok JD, Hoos A, O'Day S, Weber JS, Hammid O, Lebbé, et al. Guidelines for the evaluation of immune therapy activity in solid tumors: immune-related response criteria. Clin Cancer Res. 2009;15:7412-20.

Wolchok et al 2013

Wolchok JD, Kluger H, Callahan MK, Postow MA, Rizvi NA, Lesokhin AM, et al. Nivolumab plus ipilimumab in advanced melanoma. N Engl J Med 2013;369(2):122-33.

Yao et al, 2008

Yao JC, Hassan M, Phan A, et al. One hundred years after “carcinoid”: epidemiology of and prognostic factors for neuroendocrine tumors in 35.825 cases in the United States. J Clin Oncol. 2008;26:30663-3072.

Yao et al, 2011

Yao J., Shah M., Ito T., Bohas C., Wolin E., Van Cutsem E., et al (2011). Everolimus for advanced pancreatic neuroendocrine tumors. N Engl J Med 364: 514-523.

Yao et al, 2015

Yao JC, Fazio N, Singh S, et al. Everolimus for the treatment of advanced, non-functional neuroendocrine tumours of the lung or gastrointestinal tract (RADIANT-4): a randomised, placebo-controlled, phase 3 study. Lancet 2015; Advanced online publication 15 December.

Yuan et al 2011

Yuan J, Adamow M, Ginsberg BA, Rasalan TS, Ritter E, Gallardo HF, et al. Integrated NY-ESO-1 antibody and CD8+ T-cell responses correlated with clinical benefit in advanced melanoma patients treated with ipilimumab. Proc Natl Acad Sci USA 2011;108(40):16723-8.

Zhang et al 2008

Zhang C, Wu S, Xue X, Li M, Qin X, Li W, et al. Anti-tumour immunotherapy by blockade of the PD-1/pd-L1 pathway with recombinant human PD-1IgV. Cytotherapy 2008;10(7):711-9. doi: 10.1080.14653240802320237.

Zhang et al 2012

Zhang S, Shi R, Li C, et al. Fixed dosing versus body size-based dosing of therapeutic peptides and proteins in adults. J Clin Pharmacol. 2012;52(1):18–28.

Zou and Chen, 2008

Zou W, chen L. Inhibitory B7-family molecules in the tumour microenvironment. Nat Rev Immunol. 2008;8(6):467-77.

Appendix 1 Dosing Modification and Toxicity Management Guidelines for Immune-Mediated, Infusion-Related, and Non-Immune–Mediated Reactions (MEDI4736 Monotherapy or Combination Therapy With Tremelimumab or Tremelimumab Monotherapy)

Dosing Modification and Toxicity Management Guidelines for Immune-Mediated, Infusion-Related, and Non-Immune–Mediated Reactions (MEDI4736 Monotherapy or Combination Therapy With Tremelimumab or Tremelimumab Monotherapy) 17 October 2019 Version (CTCAE v4.03)	
**General Considerations regarding Immune-Mediated Reactions**	
**Dose Modifications**	**Toxicity Management**
Drug administration modifications of study drug/study regimen will be made to manage potential immune-related AEs based on severity of treatment-emergent toxicities graded per NCI CTCAE v4.03 (unless indicated otherwise).<br><br>In addition to the criteria for permanent discontinuation of study drug/study regimen based on CTC grade/severity (table below), permanently discontinue study drug/study regimen for the following conditions:<br><br>• Inability to reduce corticosteroid to a dose of ≤10 mg of prednisone per day (or equivalent) within 12 weeks of the start of the immune-mediated adverse event (imAE)<br><br>• Grade 3 recurrence of a previously experienced treatment-related imAE following resumption of dosing<br><br>Grade 1 No dose modification<br><br>Grade 2 Hold study drug/study regimen dose until Grade 2 resolution to Grade ≤1.<br><br>If toxicity worsens, then treat as Grade 3 or Grade 4.<br><br>Study drug/study regimen can be resumed once event stabilizes to Grade ≤1 after completion of steroid taper.<br><br>Patients with endocrinopathies who may require prolonged or continued steroid replacement can be retreated with study drug/study regimen on the following conditions:<br><br>1. The event stabilizes and is controlled.<br><br>2. The patient is clinically stable as per Investigator or treating physician's clinical judgement.<br><br>3. Doses of prednisone are at ≤10 mg/day or equivalent.<br><br>Grade 3 Depending on the individual toxicity, study drug/study regimen may be permanently discontinued. Please refer to guidelines below.<br><br>Grade 4 Permanently discontinue study drug/study regimen.	It is recommended that management of immune-mediated adverse events (imAEs) follows the guidelines presented in this table:<br><br>– It is possible that events with an inflammatory or immune mediated mechanism could occur in nearly all organs, some of them not noted specifically in these guidelines.<br><br>– Whether specific immune-mediated events (and/or laboratory indicators of such events) are noted in these guidelines or not, patients should be thoroughly evaluated to rule out any alternative etiology (e.g., disease progression, concomitant medications, and infections) to a possible immune-mediated event. In the absence of a clear alternative etiology, all such events should be managed as if they were immune related. General recommendations follow.<br><br>– Symptomatic and topical therapy should be considered for low-grade (Grade 1 or 2, unless otherwise specified) events.<br><br>– For persistent (>3 to 5 days) low-grade (Grade 2) or severe (Grade ≥3) events, promptly start prednisone 1 to 2 mg/kg/day PO or IV equivalent.<br><br>– Some events with high likelihood for morbidity and/or mortality – e.g., myocarditis, or other similar events even if they are not currently noted in the guidelines – should progress rapidly to high dose IV corticosteroids (methylprednisolone at 2 to 4 mg/kg/day) even if the event is Grade 2, and if clinical suspicion is high and/or there has been clinical confirmation. Consider, as necessary, discussing with the study physician, and promptly pursue specialist consultation.<br><br>– If symptoms recur or worsen during corticosteroid tapering (28 days of taper), increase the corticosteroid dose (prednisone dose [e.g., up to 2 to 4 mg/kg/day PO or IV equivalent]) until stabilization or improvement of symptoms, then resume corticosteroid tapering at a slower rate (>28 days of taper).

Note: For asymptomatic amylase or lipase levels of >2.0×ULN, hold study drug/study regimen, and if complete work up shows no evidence of pancreatitis, study drug/study regimen may be continued or resumed.<br><br>Note: Study drug/study regimen should be permanently discontinued in Grade 3 events with high likelihood for morbidity and/or mortality – e.g., myocarditis, or other similar events even if they are not currently noted in the guidelines. Similarly, consider whether study drug/study regimen should be permanently discontinued in Grade 2 events with high likelihood for morbidity and/or mortality – e.g., myocarditis, or other similar events even if they are not currently noted in the guidelines – when they do not rapidly improve to Grade <1 upon treatment with systemic steroids and following full taper<br><br>Note: There are some exceptions to permanent discontinuation of study drug for Grade 4 events (i.e., hyperthyroidism, hypothyroidism, Type 1 diabetes mellitus).	– More potent immunosuppressives such as TNF inhibitors (e.g., infliximab; also refer to the individual sections of the imAEs for specific type of immunosuppressive) should be considered for events not responding to systemic steroids. Progression to use of more potent immunosuppressives should proceed more rapidly in events with high likelihood for morbidity and/or mortality – e.g., myocarditis, or other similar events even if they are not currently noted in the guidelines – when these events are not responding to systemic steroids.<br><br>– With long-term steroid and other immunosuppressive use, consider need for *Pneumocystis jirovecii* pneumonia (PJP, formerly known as *Pneumocystis carinii* pneumonia) prophylaxis, gastrointestinal protection, and glucose monitoring.<br><br>– Discontinuation of study drug/study regimen is not mandated for Grade 3/Grade 4 inflammatory reactions attributed to local tumor response (e.g., inflammatory reaction at sites of metastatic disease and lymph nodes). Continuation of study drug/study regimen in this situation should be based upon a benefit-risk analysis for that patient.

AE Adverse event; CTC Common Toxicity Criteria; CTCAE Common Terminology Criteria for Adverse Events; imAE immune-mediated adverse event; IV intravenous; NCI National Cancer Institute; PO By mouth.

Pediatric Considerations regarding Immune-Mediated Reactions	
**Dose Modifications**	**Toxicity Management**
The criteria for permanent discontinuation of study drug/study regimen based on CTC grade/severity is the same for pediatric patients as it is for adult patients, as well as to permanently discontinue study drug/study regimen if unable to reduce corticosteroid ≤ a dose equivalent to that required for corticosteroid replacement therapy within 12 weeks of the start of the immune-mediated event	– All recommendations for specialist consultation should occur with a pediatric specialist in the specialty recommended.<br>– The recommendations for dosing of steroids (i.e., mg/kg/day) and for IV IG and plasmapheresis that are provided for adult patients should also be used for pediatric patients.<br>– The infliximab 5 mg/kg IV dose recommended for adults is the same as recommended for pediatric patients ≥ 6 years old. For dosing in children younger than 6 years old, consult with a pediatric specialist.<br>– For pediatric dosing of mycophenolate mofetil, consult with a pediatric specialist.<br><br>– With long-term steroid and other mmunosuppressive use, consider need for PJP prophylaxis, gastrointestinal protection, and glucose monitoring.

Specific Immune-Mediated Reactions			
**Adverse Events**	**Severity Grade of the Event (NCI CTCAE version 4.03)**	**Dose Modifications**	**Toxicity Management**
**Pneumonitis/Interstitial Lung Disease (ILD)**	**Any Grade**	**General Guidance**	**For Any Grade:**<br>– Monitor patients for signs and symptoms of pneumonitis or ILD (new onset or worsening shortness of breath or cough). Patients should be evaluated with imaging and pulmonary function tests, including other diagnostic procedures as described below.<br>– Initial work-up may include clinical evaluation, monitoring of oxygenation via pulse oximetry (resting and exertion), laboratory work-up, and high- resolution CT scan.
	**Grade 1**<br>**(asymptomatic, clinical or diagnostic observations only; intervention not indicated)**	**No dose modifications required. However, consider holding study drug/study regimen dose as clinically appropriate and during diagnostic work-up for other etiologies.**	**For Grade 1 (radiographic changes only):**<br>– Monitor and closely follow up in 2 to 4 days for clinical symptoms, pulse oximetry (resting and exertion), and laboratory work-up and then as clinically indicated.<br>– Consider Pulmonary and Infectious Disease consults.
	**Grade 2**<br>**(symptomatic; medical intervention indicated; limiting instrumental ADL)**	**Hold study drug/study regimen dose until Grade 2 resolution to Grade ≤1.**<br>• If toxicity worsens, then treat as Grade 3 or Grade 4.<br>• If toxicity improves to Grade ≤1, then the decision to reinitiate study drug/study regimen will be based upon treating physician's clinical judgment and after completion of steroid taper.	**For Grade 2 (mild to moderate new symptoms):**<br>– Monitor symptoms daily and consider hospitalization.<br>– Promptly start systemic steroids (e.g., prednisone 1 to 2 mg/kg/day PO or IV equivalent).<br>– Reimage as clinically indicated.<br>– If no improvement within 3 to 5 days, additional workup should be considered and prompt treatment with IV methylprednisolone 2 to 4 mg/kg/day started<br>– If still no improvement within 3 to 5 days despite IV methylprednisolone at 2 to 4 mg/kg/day, promptly start immunosuppressive therapy such as TNF inhibitors (e.g., infliximab at 5 mg/kg every 2 weeks). Caution: It is important to rule out sepsis and refer to infliximab label for general guidance before using infliximab.

GETNE Grupo Español de Tumores Neuroendocrinos y Endocrinos

			– Once the patient is improving, gradually taper steroids over ≥28 days and consider prophylactic antibiotics, antifungals, or anti-PJP treatment (refer to current NCCN guidelines for treatment of cancer-related infections)[a]<br>– Consider Pulmonary and Infectious Disease consults.<br>– Consider, as necessary, discussing with study physician.
	Grade 3 or 4<br>(Grade 3: severe symptoms; limiting self-care ADL; oxygen indicated)<br>(Grade 4: life-threatening respiratory compromise; urgent intervention indicated [e.g., tracheostomy or intubation])	Permanently discontinue study drug/study regimen.	For Grade 3 or 4 (severe or new symptoms, new/worsening hypoxia, life-threatening):<br>– Promptly initiate empiric IV methylprednisolone 1 to 4 mg/kg/day or equivalent.<br>– Obtain Pulmonary and Infectious Disease consults; consider, as necessary, discussing with study physician.<br>– Hospitalize the patient.<br>– Supportive care (e.g., oxygen).<br>– If no improvement within 3 to 5 days, additional workup should be considered and prompt treatment with additional immunosuppressive therapy such as TNF inhibitors (e.g., infliximab at 5 mg/kg every 2 weeks' dose) started. Caution: rule out sepsis and refer to infliximab label for general guidance before using infliximab.<br>– Once the patient is improving, gradually taper steroids over ≥28 days and consider prophylactic antibiotics, antifungals, and, in particular, anti-PJP treatment (refer to current NCCN guidelines for treatment of cancer-related infections).[a]
Diarrhea/Colitis<br>Large intestine perforation/Intestine perforation	Any Grade	General Guidance	For Any Grade:<br>– Monitor for symptoms that may be related to diarrhea/enterocolitis (abdominal pain, cramping, or changes in bowel habits such as increased frequency over baseline or blood in stool) or related to bowel perforation (such as sepsis, peritoneal signs, and ileus).<br>– When symptoms or evaluation indicate a perforation is suspected, consult a surgeon

			experienced in abdominal surgery immediately without any delay.<br>– Patients should be thoroughly evaluated to rule out any alternative etiology (e.g., disease progression, other medications, or infections), including testing for clostridium difficile toxin, etc.<br>– Steroids should be considered in the absence of clear alternative etiology, even for low-grade events, in order to prevent potential progression to higher grade event, including perforation.<br>– Use analgesics carefully; they can mask symptoms of perforation and peritonitis.
	Grade 1<br>(Diarrhea: stool frequency of <4 over baseline per day) (Colitis: asymptomatic; clinical or diagnostic observations only)	No dose modifications.	For Grade 1:<br>– Monitor closely for worsening symptoms.<br>– Consider symptomatic treatment, including hydration, electrolyte replacement, dietary changes (e.g., American Dietetic Association colitis diet), and loperamide. Use probiotics as per treating physician's clinical judgment.
	Grade 2<br>(Diarrhea: stool frequency of 4 to 6 over baseline per day) (Colitis: abdominal pain; mucus or blood in stool)<br>(Perforation: symptomatic; medical intervention indicated*)<br><br>* "medical intervention" is not invasive	Hold study drug/study regimen until resolution to Grade ≤1<br>• If toxicity worsens, then treat as Grade 3 or Grade 4.<br>• If toxicity improves to Grade ≤1, then study drug/study regimen can be resumed after completion of steroid taper.	For Grade 2:<br>– Consider symptomatic treatment, including hydration, electrolyte replacement, dietary changes (e.g., American Dietetic Association colitis diet), and loperamide and/or budesonide.<br>– Promptly start prednisone 1 to 2 mg/kg/day PO or IV equivalent.<br>– If event is not responsive within 3 to 5 days or worsens despite prednisone at 1 to 2 mg/kg/day PO or IV equivalent, GI consult should be obtained for consideration of further workup, such as imaging and/or colonoscopy, to confirm colitis and rule out perforation, and prompt treatment with IV methylprednisolone 2 to 4 mg/kg/day started.<br>– If still no improvement within 3 to 5 days despite 2 to 4 mg/kg IV methylprednisolone, promptly

			start immunosuppressives such as infliximab at 5 mg/kg once every 2 weeks[a]. Caution: it is important to rule out bowel perforation and refer to infliximab label for general guidance before using infliximab.<br>– Consider, as necessary, discussing with study physician if no resolution to Grade ≤1 in 3 to 4 days.<br>– Once the patient is improving, gradually taper steroids over ≥28 days and consider prophylactic antibiotics, antifungals, and anti-PJP treatment (refer to current NCCN guidelines for treatment of cancer-related infections).[a]
	Grade 3 or 4<br>(Grade 3 Diarrhea: stool frequency of ≥7 over baseline per day;<br>Grade 4 Diarrhea: life threatening consequences)<br>(Grade 3 Colitis: severe abdominal pain, change in bowel habits, medi-cal intervention indi-cated, peritoneal signs; Grade 4 Colitis: life-threatening consequences, urgent intervention indicated)<br>(Grade 3 Perforation: severe symptoms, elective* operative intervention indicated; Grade 4 Perforation: life-threatening consequences, urgent	Grade 3<br>Permanently discontinue study drug/study regimen for Grade 3 if toxicity does not improve to Grade ≤1 within 14 days; study drug/study regimen can be resumed after completion of steroid taper.<br><br>Grade 4<br>Permanently discontinue study drug/study regimen.	For Grade 3 or 4:<br>– Promptly initiate empiric IV methylprednisolone 2 to 4 mg/kg/day or equivalent.<br>– Monitor stool frequency and volume and maintain hydration.<br>– Urgent GI consult and imaging and/or colonoscopy as appropriate.<br>– If still no improvement within 3 to 5 days of IV methylprednisolone 2 to 4 mg/kg/day or equivalent, promptly start further immunosuppressives (e.g., infliximab at 5 mg/kg once every 2 weeks). Caution: Ensure GI consult to rule out bowel perforation and refer to infliximab label for general guidance before using infliximab. If perforation is suspected, consult a surgeon experienced in abdominal surgery immediately without any delay.<br>– Once the patient is improving, gradually taper steroids over ≥28 days and consider prophylactic antibiotics, antifungals, and anti-PJP treatment (refer to current NCCN guidelines for treatment of cancer-related infections).[a]

GETNE
Grupo Español de Tumores Neuroendocrinos y Endocrinos

	intervention indicated)<br><br>*This guidance anticipates that Grade 3 operative interventions of perforations are usually not elective		
**Hepatitis (elevated LFTs)**<br>**Infliximab should not be used for management of immune-related hepatitis.**	Any Elevations in AST, ALT or TB as Described Below	General Guidance	For Any Elevations Described:<br>– Monitor and evaluate liver function test: AST, ALT, ALP, and TB.<br>– Evaluate for alternative etiologies (e.g., viral hepatitis, disease progression, concomitant medications).
	AST or ALT >ULN and ≤3.0×ULN if baseline normal, 1.5-3.0×baseline if baseline abnormal; and/or TB > ULN and ≤1.5×ULN if baseline normal, >1.0-1.5×baseline if baseline abnormal	• No dose modifications.<br>• If it worsens, then treat as described for elevations in the row below.	– Continue LFT monitoring per protocol.
	AST or ALT >3.0×ULN and ≤5.0×ULN if baseline normal, >3-5×baseline if baseline abnormal; and/or TB >1.5×ULN and ≤3.0×ULN if baseline normal, >1.5-3.0×baseline if baseline abnormal	• Hold study drug/study regimen dose until resolution to AST or ALT ≤3.0×ULN and/or TB ≤1.5×ULN if baseline normal, or to AST or ALT ≤3.0×baseline and/or TB ≤1.5×baseline if baseline abnormal.<br>• If toxicity worsens, then treat as described for elevation in the row below.<br>• If toxicity improves to AST or ALT ≤3.0×ULN and/or TB ≤1.5×ULN if baseline normal, or to AST or ALT ≤3.0×baseline and/or TB ≤1.5×baseline if baseline abnormal, resume study drug/study regimen after	– Regular and frequent checking of LFTs (e.g., every 1 to 2 days) until elevations of these are improving or resolved.<br>– If no resolution to AST or ALT ≤3.0×ULN and/or TB ≤1.5×ULN if baseline normal, or to AST or ALT ≤3.0×baseline and/or TB ≤1.5×baseline if baseline abnormal, in 1 to 2 days, consider, as necessary, discussing with study physician.<br>– If event is persistent (>3 to 5 days) or worsens, promptly start prednisone 1 to 2 mg/kg/day PO or IV equivalent.<br>– If still no improvement within 3 to 5 days despite 1 to 2 mg/kg/day of prednisone PO or IV equivalent, consider

		completion of steroid taper.	additional work up and start prompt treatment with IV methylprednisolone 2 to 4 mg/kg/day.<br>– If still no improvement within 3 to 5 days despite 2 to 4 mg/kg/day of IV methylprednisolone, promptly start immunosuppressives (i.e., mycophenolate mofetil).[a] Discuss with study physician if mycophenolate mofetil is not available. Infliximab should NOT be used.<br>– Once the patient is improving, gradually taper steroids over ≥28 days and consider prophylactic antibiotics, antifungals, and anti-PJP treatment (refer to current NCCN guidelines for treatment of cancer-related infections).[a]
	AST or ALT >5.0×ULN if baseline normal, >5×baseline if baseline abnormal; and/or TB >3.0×ULN if baseline normal; >3.0×baseline if baseline abnormal	For elevations in transaminases ≤8×ULN and/or in TB ≤5×ULN if baseline normal, or for elevations in transaminases ≤8×baseline and/or TB ≤5×baseline if baseline abnormal:<br>• Hold study drug/study regimen dose until resolution to AST or ALT ≤3.0×ULN and/or TB ≤1.5×ULN if baseline normal, or to AST or ALT ≤3.0×baseline and/or TB ≤1.5×baseline if baseline abnormal<br>• Resume study drug/study regimen if elevations downgrade to AST or ALT ≤3.0×ULN and/or TB ≤1.5×ULN if baseline normal, or to AST or ALT ≤3.0×baseline and/or TB ≤1.5×baseline if baseline abnormal, within 14 days and after completion of steroid taper.<br>• Permanently discontinue study drug/study regimen if the elevations do not downgrade as described	– Promptly initiate empiric IV methylprednisolone at 1 to 4 mg/kg/day or equivalent.<br>– If still no improvement within 3 to 5 days despite 1 to 4 mg/kg/day methylprednisolone IV or equivalent, promptly start treatment with immunosuppressive therapy (i.e., mycophenolate mofetil). Discuss with study physician if mycophenolate is not available. Infliximab should NOT be used.<br>– Request Hepatology consult, and perform abdominal workup and imaging as appropriate.<br>– Once the patient is improving, gradually taper steroids over ≥28 days and consider prophylactic antibiotics, antifungals, and anti-PJP treatment (refer to current NCCN guidelines for treatment of cancer-related infections).[a]

GETNE Grupo Español de Tumores Neuroendocrinos y Endocrinos

		in bullet above within 14 days<br>For elevations in transaminases >8×ULN or elevations in TB >5×ULN if baseline normal, or for elevations in transaminases >8×baseline and/or TB >5×baseline if baseline abnormal, permanently discontinue study drug/study regimen.<br>Permanently discontinue study drug/study regimen for any case meeting Hy's law criteria (AST and/or ALT >3 × ULN + bilirubin >2 × ULN without initial findings of cholestasis (i.e., elevated alkaline P04) and in the absence of any alternative cause.[b]	
Hepatitis (elevated LFTs)<br>Infliximab should not be used for management of immune-related hepatitis.<br>See instructions at bottom of shaded area if transaminase rise is not isolated but (at any time) occurs in setting of either increasing bilirubin or signs of DILI/liver decompensation	Any Elevations in AST, ALT or TB as Described Below	General Guidance	For Any Elevations Described:<br>– Monitor and evaluate liver function test: AST, ALT, ALP, and TB.<br>– Evaluate for alternative etiologies (e.g., viral hepatitis, disease progression, concomitant medications, worsening of liver cirrhosis [e.g., portal vein thrombosis]).<br>– For HBV+ patients: evaluate quantitative HBV viral load, quantitative HBsAg, or HBeAg<br>– For HCV+ patients: evaluate quantitative HCV viral load<br>– Consider consulting hepatologist/Infectious Disease specialist regarding change/implementation in/of antiviral medications for any patient with an elevated HBV viral load >2000 IU/ml<br>– Consider consulting hepatologist/Infectious Disease specialist regarding change/implementation in/of antiviral HCV medications if HCV viral load increased by ≥2-fold<br>– For HCV+ with HBcAB+: Evaluate for both HBV and HCV as above
		• No dose modifications.	

	**Isolated AST or ALT >ULN and ≤5.0×ULN, whether normal or elevated at baseline**	• **If ALT/AST elevations represents significant worsening based on investigator assessment, then treat as described for elevations in the row below.**<br>**For all transaminase elevations, see instructions at bottom of shaded area if transaminase rise is not isolated but (at any time) occurs in setting of either increasing bilirubin or signs of DILI/liver decompensation**	

	Isolated AST or ALT >5.0×ULN and ≤8.0×ULN, if normal at baseline<br><br>Isolated AST or ALT >2.0×baseline and ≤12.5×ULN, if elevated >ULN at baseline	• Hold study drug/study regimen dose untilresolution to AST or ALT ≤5.0×ULN.<br>• If toxicity worsens, then treat as described for elevations in the rows below.<br>If toxicity improves to AST or ALT ≤5.0×ULN, resume study drug/study regimen after completion of steroid taper.	– Regular and frequent checking of LFTs (e.g., every 1 to 3 days) until elevations of these are improving or resolved.<br>– Recommend consult hepatologist; consider abdominal ultrasound, including Doppler assessment of liver perfusion.<br>– Consider, as necessary, discussing with study physician.<br>– If event is persistent (>3 to 5 days) or worsens, and investigator suspects toxicity to be immune-mediated AE, recommend to start prednisone 1 to 2 mg/kg/day PO or IV equivalent.<br>– If still no improvement within 3 to 5 days despite 1 to 2 mg/kg/day of prednisone PO or IV equivalent, consider additional workup and treatment with IV methylprednisolone 2 to 4 mg/kg/day.<br>– If still no improvement within 3 to 5 days despite 2 to 4 mg/kg/day of IV methylprednisolone, consider additional abdominal workup (including liver biopsy) and imaging (i.e., liver ultrasound), and consider starting immunosuppressives (i.e., mycophenolate mofetil).[a] Discuss with study physician if mycophenolate mofetil is not available. Infliximab should NOT be used.
	Isolated AST or ALT >8.0×ULN and ≤20.0×ULN, if normal at baseline<br><br>Isolated AST or ALT >12.5×ULN and ≤20.0×ULN, if elevated >ULN at baseline	• Hold study drug/study regimen dose until resolution to AST or ALT ≤5.0×ULN<br>• Resume study drug/study regimen if elevations downgrade to AST or ALT ≤5.0×ULN within 14 days and after completion of steroid taper.<br>• Permanently discontinue study drug/study regimen if the elevations do not	– Regular and frequent checking of LFTs (e.g., every 1-2 days) until elevations of these are improving or resolved.<br>– Consult hepatologist (unless investigator is hepatologist); obtain abdominal ultrasound, including Doppler assessment of liver perfusion; and consider liver biopsy.<br>– Consider, as necessary, discussing with study physician.<br>– If investigator suspects toxicity to be immune-mediated, promptly initiate empiric IV

		downgrade to AST or ALT ≤5.0×ULN within 14 days<br>Permanently discontinue study drug/study regimen for any case meeting Hy's law criteria, in the absence of any alternative cause.[b]	methylprednisolone at 1 to 4 mg/kg/day or equivalent.<br>– If no improvement within 3 to 5 days despite 1 to 4 mg/kg/day methylprednisolone IV or equivalent, obtain liver biopsy (if it has not been done already) and promptly start treatment with immunosuppressive therapy (mycophenolate mofetil). Discuss with study physician if mycophenolate is not available. Infliximab should NOT be used.<br>– Once the patient is improving, gradually taper steroids over ≥28 days and consider prophylactic antibiotics, antifungals, and anti-PCP treatment (refer to current NCCN guidelines for treatment of cancer-related infections).[a]
	Isolated AST or ALT >20×ULN, whether normal or elevated at baseline	Permanently discontinue study drug/study regimen.	Same as above<br>(except would recommend obtaining liver biopsy early)
If transaminase rise is not isolated but (at any time) occurs in setting of either increasing total/direct bilirubin (≥1.5×ULN, if normal at baseline; or 2×baseline, if >ULN at baseline) or signs of DILI/liver decompensation (e.g., fever, elevated INR):<br>- Manage dosing for each level of transaminase rise as instructed for the next highest level of transaminase riseFor example, manage dosing for second level of transaminase rise (i.e., AST or ALT >5.0×ULN and ≤8.0×ULN, if normal at baseline, or AST or ALT >2.0×baseline and ≤12.5×ULN, if elevated >ULN at baseline) as instructed for the third level of transaminase rise (i.e., AST or ALT >8.0×ULN and ≤20.0×ULN, if normal at baseline, or AST or ALT >12.5×ULN and ≤20.0×ULN, if elevated >ULN at baseline)<br>- For the third and fourth levels of transaminase rises, permanently discontinue study drug/study regimen			
Nephritis or renal dysfunction<br>(elevated serum creatinine)	Any Grade	General Guidance	For Any Grade:<br>– Consult with nephrologist.<br>– Monitor for signs and symptoms that may be related to changes in renal function (e.g., routine urinalysis, elevated serum BUN and creatinine, decreased creatinine clearance, electrolyte imbalance, decrease in urine output, or proteinuria).<br>– Patients should be thoroughly evaluated to rule out any

			**alternative etiology (e.g., disease progression or infections).**<br>– **Steroids should be considered in the absence of clear alternative etiology even for low-grade events (Grade 2), in order to prevent potential progression to higher grade event.**

	**Grade 1**<br>**(Serum creatinine > 1 to 1.5×baseline; > ULN to 1.5×ULN)**	**No dose modifications.**	**For Grade 1:**<br>– **Monitor serum creatinine weekly and any accompanying symptoms.**<br>• **If creatinine returns to baseline, resume its regular monitoring per study protocol.**<br>• **If creatinine worsens, depending on the severity, treat as Grade 2, 3, or 4.**<br>– **Consider symptomatic treatment, including hydration, electrolyte replacement, and diuretics.**
	**Grade 2**<br>**(serum creatinine >1.5 to 3.0×baseline; >1.5 to 3.0×ULN)**	**Hold study drug/study regimen until resolution to Grade ≤1 or baseline.**<br>• **If toxicity worsens, then treat as Grade 3 or 4.**<br>• **If toxicity improves to Grade ≤1 or baseline, then resume study drug/study regimen after completion of steroid taper.**	**For Grade 2:**<br>– **Consider symptomatic treatment, including hydration, electrolyte replacement, and diuretics.**<br>– **Carefully monitor serum creatinine every 2 to 3 days and as clinically warranted.**<br>– **Consult nephrologist and consider renal biopsy if clinically indicated.**<br>– **If event is persistent (>3 to 5 days) or worsens, promptly start prednisone 1 to 2 mg/kg/day PO or IV equivalent.**<br>– **If event is not responsive within 3 to 5 days or worsens despite prednisone at 1 to 2 mg/kg/day PO or IV equivalent, additional workup should be considered and prompt treatment with IV methylprednisolone at 2 to 4 mg/kg/day started.**<br>– **Once the patient is improving, gradually taper steroids over ≥28 days and consider prophylactic antibiotics, antifungals, and anti-PJP treatment (refer to current NCCN guidelines for treatment of cancer-related infections).[a]**<br>– **When event returns to baseline, resume study drug/study regimen and routine serum creatinine monitoring per study protocol.**

	Grade 3 or 4<br>(Grade 3: serum creatinine >3.0×baseline; >3.0 to 6.0×ULN)<br><br>(Grade 4: serum creatinine >6.0×ULN)	Permanently discontinue study drug/study regimen.	For Grade 3 or 4:<br>– Carefully monitor serum creatinine on daily basis.<br>– Consult nephrologist and consider renal biopsy if clinically indicated.<br>– Promptly start prednisone 1 to 2 mg/kg/day PO or IV equivalent.<br>– If event is not responsive within 3 to 5 days or worsens despite prednisone at 1 to 2 mg/kg/day PO or IV equivalent, additional workup should be considered and prompt treatment with IV methylprednisolone 2 to 4 mg/kg/day started.<br>– Once the patient is improving, gradually taper steroids over ≥28 days and consider prophylactic antibiotics, antifungals, and anti-PJP treatment (refer to current NCCN guidelines for treatment of cancer-related infections).[a]
Rash or Dermatitis<br>(including Pemphigoid)	Any Grade<br>(refer to NCI CTCAE v 4.03 for definition of severity/grade depending on type of skin rash)	General Guidance	For Any Grade:<br>– Monitor for signs and symptoms of dermatitis (rash and pruritus).<br>– IF THERE IS ANY BULLOUS FORMATION, THE STUDY PHYSICIAN SHOULD BE CONTACTED AND STUDY DRUG DISCONTINUED IF SUSPECT STEVENS-JOHNSON SYNDROME OR TOXIC EPIDERMAL NECROLYSIS.
	Grade 1	No dose modifications.	For Grade 1:<br>– Consider symptomatic treatment, including oral antipruritics (e.g., diphenhydramine or hydroxyzine) and topical therapy (e.g., urea cream).
	Grade 2	For persistent (>1 to 2 weeks) Grade 2 events, hold scheduled study drug/study regimen until resolution to Grade ≤1 or baseline.<br>• If toxicity worsens, then treat as Grade 3.<br>• If toxicity improves to Grade ≤1 or baseline,	For Grade 2:<br>– Obtain Dermatology consult.<br>– Consider symptomatic treatment, including oral antipruritics (e.g., diphenhydramine or hydroxyzine) and topical therapy (e.g., urea cream).

		**then resume drug/study regimen after completion of steroid taper.**	– **Consider moderate-strength topical steroid.**<br>– **If no improvement of rash/skin lesions occurs within 3 to 5 days or is worsening despite symptomatic treatment and/or use of moderate strength topical steroid, consider, as necessary, discussing with study physician and promptly start systemic steroids such as prednisone 1 to 2 mg/kg/day PO or IV equivalent.**<br>– **Consider skin biopsy if the event is persistent for >1 to 2 weeks or recurs.**

	Grade 3 or 4	For Grade 3:<br>Hold study drug/study regimen until resolution to Grade ≤1 or baseline.<br>If temporarily holding the study drug/study regimen does not provide improvement of the Grade 3 skin rash to Grade ≤1 or baseline within 30 days, then permanently discontinue study drug/study regimen.<br><br>For Grade 4:<br>Permanently discontinue study drug/study regimen.	For Grade 3 or 4:<br>– Consult Dermatology.<br>– Promptly initiate empiric IV methylprednisolone 1 to 4 mg/kg/day or equivalent.<br>– Consider hospitalization.<br>– Monitor extent of rash [Rule of Nines].<br>– Consider skin biopsy (preferably more than 1) as clinically feasible.<br>– Once the patient is improving, gradually taper steroids over ≥28 days and consider prophylactic antibiotics, antifungals, and anti-PJP treatment (refer to current NCCN guidelines for treatment of cancer-related infections).[a]<br>– Consider, as necessary, discussing with study physician.
Endocrinopathy<br>(e.g., hyperthyroidism, thyroiditis, hypothyroidism, Type 1 diabetes mellitus, hypophysitis, hypopituitarism, and adrenal insufficiency; exocrine event of amylase/lipase increased also included in this section)	Any Grade<br>(depending on the type of endocrinopathy, refer to NCI CTCAE v4.03 for defining the CTC grade/severity)	General Guidance	For Any Grade:<br>– Consider consulting an endocrinologist for endocrine events.<br>– Consider, as necessary, discussing with study physician.<br>– Monitor patients for signs and symptoms of endocrinopathies. Non-specific symptoms include headache, fatigue, behavior changes, changed mental status, vertigo, abdominal pain, unusual bowel habits, polydipsia, polyuria, hypotension, and weakness.<br>– Patients should be thoroughly evaluated to rule out any alternative etiology (e.g., disease progression including brain metastases, or infections).<br>– Depending on the suspected endocrinopathy, monitor and evaluate thyroid function tests: TSH, free T3 and free T4 and other relevant endocrine and related labs (e.g., blood glucose and ketone levels, HgA1c).<br>– For asymptomatic elevations in serum amylase and lipase >ULN and <3×ULN, corticosteroid treatment is not indicated as long as there are no other signs or

			symptoms of pancreatic inflammation.<br>– If a patient experiences an AE that is thought to be possibly of autoimmune nature (e.g., thyroiditis, pancreatitis, hypophysitis, or diabetes insipidus), the investigator should send a blood sample for appropriate autoimmune antibody testing.
	Grade 1	No dose modifications.	For Grade 1 (including those with asymptomatic TSH elevation):<br>– Monitor patient with appropriate endocrine function tests.<br>– For suspected hypophysitis/hypopituitarism, consider consultation of an endocrinologist to guide assessment of early-morning ACTH, cortisol, TSH and free T4; also consider gonadotropins, sex hormones, and prolactin levels, as well as cosyntropin stimulation test (though it may not be useful in diagnosing early secondary adrenal insufficiency).<br>– If TSH < 0.5 × LLN, or TSH >2 × ULN, or consistently out of range in 2 subsequent measurements, include free T4 at subsequent cycles as clinically indicated and consider consultation of an endocrinologist.
	Grade 2	For Grade 2 endocrinopathy other than hypothyroidism and Type 1 diabetes mellitus, hold study drug/study regimen dose until patient is clinically stable.<br>• If toxicity worsens, then treat as Grade 3 or Grade 4.<br>Study drug/study regimen can be resumed once event stabilizes and after completion of steroid taper.<br>Patients with endocrinopathies who may require prolonged or continued steroid replacement (e.g., adrenal insufficiency) can be retreated with study drug/study	For Grade 2 (including those with symptomatic endocrinopathy):<br>– Consult endocrinologist to guide evaluation of endocrine function and, as indicated by suspected endocrinopathy and as clinically indicated, consider pituitary scan.<br>– For all patients with abnormal endocrine work up, except those with isolated hypothyroidism or Type 1 DM, and as guided by an endocrinologist, consider short-term corticosteroids (e.g., 1 to 2 mg/kg/day methylprednisolone or IV equivalent) and prompt initiation of treatment with relevant hormone replacement (e.g., hydrocortisone, sex hormones).

		regimen on the following conditions:<br>1. The event stabilizes and is controlled.<br>2. The patient is clinically stable as per investigator or treating physician's clinical judgement.<br>3. Doses of prednisone are ≤10 mg/day or equivalent.	– Isolated hypothyroidism may be treated with replacement therapy, without study drug/study regimen interruption, and without corticosteroids.<br>– Isolated Type 1 diabetes mellitus (DM) may be treated with appropriate diabetic therapy, without study drug/study regimen interruption, and without corticosteroids.<br>– Once patients on steroids are improving, gradually taper immunosuppressive steroids (as appropriate and with guidance of endocrinologist) over ≥28 days and consider prophylactic antibiotics, antifungals, and anti-PJP treatment (refer to current NCCN guidelines for treatment of cancer-related infections).[a]<br>– For patients with normal endocrine workup (laboratory assessment or MRI scans), repeat laboratory assessments/MRI as clinically indicated.
	Grade 3 or 4	For Grade 3 or 4 endocrinopathy other than hypothyroidism and Type 1 diabetes mellitus, hold study drug/study regimen dose until endocrinopathy symptom(s) are controlled.<br>Study drug/study regimen can be resumed once event stabilizes and after completion of steroid taper.<br>Patients with endocrinopathies who may require prolonged or continued steroid replacement (e.g., adrenal insufficiency) can be retreated with study drug/study regimen on the following conditions:<br>1. The event stabilizes and is controlled.<br>2. The patient is clinically stable as per investigator or treating physician's clinical judgement.	For Grade 3 or 4:<br>– Consult endocrinologist to guide evaluation of endocrine function and, as indicated by suspected endocrinopathy and as clinically indicated, consider pituitary scan. Hospitalization recommended.<br>– For all patients with abnormal endocrine work up, except those with isolated hypothyroidism or Type 1 DM, and as guided by an endocrinologist, promptly initiate empiric IV methylprednisolone 1 to 2 mg/kg/day or equivalent, as well as relevant hormone replacement (e.g., hydrocortisone, sex hormones).<br>– For adrenal crisis, severe dehydration, hypotension, or shock, immediately initiate IV corticosteroids with mineralocorticoid activity.<br>– Isolated hypothyroidism may be treated with replacement therapy, without study drug/study regimen interruption, and without corticosteroids.

		3. Doses of prednisone are ≤10 mg/day or equivalent.	– Isolated Type 1 diabetes mellitus may be treated with appropriate diabetic therapy, without study drug/study regimen interruption, and without corticosteroids.<br>– Once patients on steroids are improving, gradually taper immunosuppressive steroids (as appropriate and with guidance of endocrinologist) over ≥28 days and consider prophylactic antibiotics, antifungals, and anti-PJP treatment (refer to current NCCN guidelines for treatment of cancer-related infections).[a]
Neurotoxicity<br>(to include but not be limited to limbic encephalitis and autonomic neuropathy, excluding Myasthenia Gravis and Guillain-Barre)	Any Grade<br>(depending on the type of neurotoxicity, refer to NCI CTCAE v4.03 for defining the CTC grade/severity)	General Guidance	For Any Grade:<br>– Patients should be evaluated to rule out any alternative etiology (e.g., disease progression, infections, metabolic syndromes, or medications).<br>– Monitor patient for general symptoms (headache, nausea, vertigo, behavior change, or weakness).<br>– Consider appropriate diagnostic testing (e.g., electromyogram and nerve conduction investigations).<br>– Perform symptomatic treatment with Neurology consult as appropriate.<br>–

	Grade 1	No dose modifications.	For Grade 1:<br>– See "Any Grade" recommendations above.
	Grade 2	For acute motor neuropathies or neurotoxicity, hold study drug/study regimen dose until resolution to Grade ≤1.<br>For sensory neuropathy/neuropathic pain, consider holding study drug/study regimen dose until resolution to Grade ≤1.<br>If toxicity worsens, then treat as Grade 3 or 4.<br>Study drug/study regimen can be resumed once event improves to Grade ≤1 and after completion of steroid taper.	For Grade 2:<br>– Consider, as necessary, discussing with the study physician.<br>– Obtain Neurology consult.<br>– Sensory neuropathy/neuropathic pain may be managed by appropriate medications (e.g., gabapentin or duloxetine).<br>– Promptly start systemic steroids prednisone 1 to 2 mg/kg/day PO or IV equivalent.<br>– If no improvement within 3 to 5 days despite 1 to 2 mg/kg/day prednisone PO or IV equivalent, consider additional workup and promptly treat with additional immunosuppressive therapy (e.g., IV IG).
	Grade 3 or 4	For Grade 3:<br>Hold study drug/study regimen dose until resolution to Grade ≤1.<br>Permanently discontinue study drug/study regimen if Grade 3 imAE does not resolve to Grade ≤1 within 30 days.<br>For Grade 4:<br>Permanently discontinue study drug/study regimen.	For Grade 3 or 4:<br>– Consider, as necessary, discussing with study physician.<br>– Obtain Neurology consult.<br>– Consider hospitalization.<br>– Promptly initiate empiric IV methylprednisolone 1 to 2 mg/kg/day or equivalent.<br>– If no improvement within 3 to 5 days despite IV corticosteroids, consider additional workup and promptly treat with additional immunosuppressants (e.g., IV IG).<br>– Once stable, gradually taper steroids over ≥28 days.
Peripheral neuromotor syndromes<br>(such as Guillain-Barre and myasthenia gravis)	Any Grade	General Guidance	For Any Grade:<br>– The prompt diagnosis of immune-mediated peripheral neuromotor syndromes is important, since certain patients may unpredictably experience acute decompensations that can result in substantial morbidity or in the worst case, death. Special care should be taken for certain sentinel symptoms that may predict a more severe outcome, such as prominent dysphagia, rapidly progressive weakness, and signs of respiratory

			insufficiency or autonomic instability.<br>– Patients should be evaluated to rule out any alternative etiology (e.g., disease progression, infections, metabolic syndromes or medications). It should be noted that the diagnosis of immune-mediated peripheral neuromotor syndromes can be particularly challenging in patients with underlying cancer, due to the multiple potential confounding effects of cancer (and its treatments) throughout the neuraxis. Given the importance of prompt and accurate diagnosis, it is essential to have a low threshold to obtain a Neurology consult.<br>– Neurophysiologic diagnostic testing (e.g., electromyogram and nerve conduction investigations, and "repetitive stimulation" if myasthenia is suspected) are routinely indicated upon suspicion of such conditions and may be best facilitated by means of a Neurology consultation.<br>– It is important to consider that the use of steroids as the primary treatment of Guillain-Barre is not typically considered effective. Patients requiring treatment should be started with IV IG and followed by plasmapheresis if not responsive to IV IG.
	Grade 1	No dose modifications.	For Grade 1:<br>– Consider, as necessary, discussing with the study physician.<br>– Care should be taken to monitor patients for sentinel symptoms of a potential decompensation as described above.<br>– Obtain a Neurology consult.
	Grade 2	Hold study drug/study regimen dose until resolution to Grade ≤1.<br>Permanently discontinue study drug/study regimen if it does not resolve to Grade ≤1 within 30 days or if there are	For Grade 2:<br>– Consider, as necessary, discussing with the study physician.<br>– Care should be taken to monitor patients for sentinel symptoms of

		signs of respiratory insufficiency or autonomic instability.	a potential decompensation as described above.<br>– Obtain a Neurology consult<br>– Sensory neuropathy/neuropathic pain may be managed by appropriate medications (e.g., gabapentin or duloxetine).<br>*MYASTHENIA GRAVIS:*<br>○ Steroids may be successfully used to treat myasthenia gravis. It is important to consider that steroid therapy (especially with high doses) may result in transient worsening of myasthenia and should typically be administered in a monitored setting under supervision of a consulting neurologist.<br>○ Patients unable to tolerate steroids may be candidates for treatment with plasmapheresis or IV IG. Such decisions are best made in consultation with a neurologist, taking into account the unique needs of each patient.<br>○ If myasthenia gravis-like neurotoxicity is present, consider starting AChE inhibitor therapy in addition to steroids. Such therapy, if successful, can also serve to reinforce the diagnosis.<br>*GUILLAIN-BARRE:*<br>○ It is important to consider here that the use of steroids as the primary treatment of Guillain-Barre is not typically considered effective.<br>○ Patients requiring treatment should be started with IV IG and followed by plasmapheresis if not responsive to IV IG.

	Grade 3 or 4	**For Grade 3:**<br>Hold study drug/study regimen dose until resolution to Grade ≤1.<br>Permanently discontinue study drug/study regimen if Grade 3 imAE does not resolve to Grade ≤1 within 30 days or if there are signs of respiratory insufficiency or autonomic instability.<br><br>**For Grade 4:**<br>Permanently discontinue study drug/study regimen.	For Grade 3 or 4 (severe or life-threatening events):<br>– Consider, as necessary, discussing with study physician.<br>– Recommend hospitalization.<br>– Monitor symptoms and obtain Neurology consult.<br>*MYASTHENIA GRAVIS:*<br>o Steroids may be successfully used to treat myasthenia gravis. They should typically be administered in a monitored setting under supervision of a consulting neurologist.<br>o Patients unable to tolerate steroids may be candidates for treatment with plasmapheresis or IV IG.<br>o If myasthenia gravis-like neurotoxicity present, consider starting AChE inhibitor therapy in addition to steroids. Such therapy, if successful, can also serve to reinforce the diagnosis.<br>*GUILLAIN-BARRE:*<br>o It is important to consider here that the use of steroids as the primary treatment of Guillain-Barre is not typically considered effective.<br>o Patients requiring treatment should be started with IV IG and followed by plasmapheresis if not responsive to IV IG.
Myocarditis	Any Grade	General Guidance<br>Discontinue drug permanently if biopsy-proven immune-mediated myocarditis.	For Any Grade:<br>– The prompt diagnosis of immune-mediated myocarditis is important, particularly in patients with baseline cardiopulmonary disease and reduced cardiac function.<br>– Consider, as necessary, discussing with the study physician.<br>– Monitor patients for signs and symptoms of myocarditis (new onset or worsening chest pain, arrhythmia, shortness of breath,

			peripheral edema). As some symptoms can overlap with lung toxicities, simultaneously evaluate for and rule out pulmonary toxicity as well as other causes (e.g., pulmonary embolism, congestive heart failure, malignant pericardial effusion). A Cardiology consultation should be obtained early, with prompt assessment of whether and when to complete a cardiac biopsy, including any other diagnostic procedures.<br>– Initial work-up should include clinical evaluation, BNP, cardiac enzymes, ECG, echocardiogram (ECHO), monitoring of oxygenation via pulse oximetry (resting and exertion), and additional laboratory work-up as indicated. Spiral CT or cardiac MRI can complement ECHO to assess wall motion abnormalities when needed.<br>– Patients should be thoroughly evaluated to rule out any alternative etiology (e.g., disease progression, other medications, or infections)
	Grade 1<br>(asymptomatic with laboratory [e.g., BNP] or cardiac imaging abnormalities)	No dose modifications required unless clinical suspicion is high, in which case hold study drug/study regimen dose during diagnostic work-up for other etiologies. If study drug/study regimen is held, resume after complete resolution to Grade 0.	For Grade 1 (no definitive findings):<br>- Monitor and closely follow up in 2 to 4 days for clinical symptoms, BNP, cardiac enzymes, ECG, ECHO, pulse oximetry (resting and exertion), and laboratory work-up as clinically indicated.<br>- Consider using steroids if clinical suspicion is high.

	Grade 2, 3 or 4<br>(Grade 2: Symptoms with mild to moderate activity or exertion)<br><br>(Grade 3: Severe with symptoms at rest or with minimal activity or exertion; intervention indicated)<br><br>(Grade 4: Life-threatening consequences; urgent intervention indicated (e.g., continuous IV therapy or mechanical hemodynamic support))	- If Grade 2 -- Hold study drug/study regimen dose until resolution to Grade 0. If toxicity rapidly improves to Grade 0, then the decision to reinitiate study drug/study regimen will be based upon treating physician's clinical judgment and after completion of steroid taper. If toxicity does not rapidly improve, permanently. discontinue study drug/study regimen.<br>If Grade 3-4, permanently discontinue study drug/study regimen.	For Grade 2-4:<br>– Monitor symptoms daily, hospitalize.<br>– Promptly start IV methylprednisolone 2 to 4 mg/kg/day or equivalent after Cardiology consultation has determined whether and when to complete diagnostic procedures including a cardiac biopsy.<br>– Supportive care (e.g., oxygen).<br>– If no improvement within 3 to 5 days despite IV methylprednisolone at 2 to 4 mg/kg/day, promptly start immunosuppressive therapy such as TNF inhibitors (e.g., infliximab at 5 mg/kg every 2 weeks). Caution: It is important to rule out sepsis and refer to infliximab label for general guidance before using infliximab.<br>– Once the patient is improving, gradually taper steroids over ≥28 days and consider prophylactic antibiotics, antifungals, or anti-PJP treatment (refer to current NCCN guidelines for treatment of cancer-related infections).[a]
Myositis/Polymyositis ("Poly/myositis")	Any Grade	General Guidance	For Any Grade:<br>– Monitor patients for signs and symptoms of poly/myositis. Typically, muscle weakness/pain occurs in proximal muscles including upper arms, thighs, shoulders, hips, neck and back, but rarely affects the extremities including hands and fingers; also difficulty breathing and/or trouble swallowing can occur and progress rapidly. Increased general feelings of tiredness and fatigue may occur, and there can be new-onset falling, difficulty getting up from a fall, and trouble climbing stairs, standing up from a seated position, and/or reaching up.<br>– If poly/myositis is suspected, a Neurology consultation should be obtained early, with prompt guidance on diagnostic procedures. Myocarditis may co-occur with poly/myositis;

			refer to guidance under Myocarditis. Given breathing complications, refer to guidance under Pneumonitis/ILD.<br>Given possibility of an existent (but previously unknown) autoimmune disorder, consider Rheumatology consultation.<br>– Consider, as necessary, discussing with the study physician.<br>– Initial work-up should include clinical evaluation, creatine kinase, aldolase, LDH, BUN/creatinine, erythrocyte sedimentation rate or C-reactive protein level, urine myoglobin, and additional laboratory work-up as indicated, including a number of possible rheumatological/antibody tests (i.e., consider whether a rheumatologist consultation is indicated and could guide need for rheumatoid factor, antinuclear antibody, anti-smooth muscle, antisynthetase [such as anti-Jo-1], and/or signal-recognition particle antibodies). Confirmatory testing may include electromyography, nerve conduction studies, MRI of the muscles, and/or a muscle biopsy. Consider Barium swallow for evaluation of dysphagia or dysphonia.<br>Patients should be thoroughly evaluated to rule out any alternative etiology (e.g., disease progression, other medications, or infections).
	Grade 1<br>(mild pain)	- No dose modifications.	For Grade 1:<br>– Monitor and closely follow up in 2 to 4 days for clinical symptoms and initiate evaluation as clinically indicated.<br>– Consider Neurology consult.<br>– Consider, as necessary, discussing with the study physician.
	Grade 2<br>(moderate pain associated with weakness; pain limiting	Hold study drug/study regimen dose until resolution to Grade ≤1.<br>- Permanently discontinue study drug/study regimen if it does not resolve to Grade	For Grade 2:<br>– Monitor symptoms daily and consider hospitalization.<br>– Obtain Neurology consult, and initiate evaluation.

	instrumental activities of daily living [ADLs])	≤1 within 30 days or if there are signs of respiratory insufficiency.	– Consider, as necessary, discussing with the study physician.<br>– If clinical course is rapidly progressive (particularly if difficulty breathing and/or trouble swallowing), promptly start IV methylprednisolone 2 to 4 mg/kg/day systemic steroids along with receiving input from Neurology consultant<br>– If clinical course is *not* rapidly progressive, start systemic steroids (e.g., prednisone 1 to 2 mg/kg/day PO or IV equivalent); if no improvement within 3 to 5 days, continue additional work up and start treatment with IV methylprednisolone 2 to 4 mg/kg/day<br>– If after start of IV methylprednisolone at 2 to 4 mg/kg/day there is no improvement within 3 to 5 days, consider start of immunosuppressive therapy such as TNF inhibitors (e.g., infliximab at 5 mg/kg every 2 weeks). Caution: It is important to rule out sepsis and refer to infliximab label for general guidance before using infliximab.<br>– Once the patient is improving, gradually taper steroids over ≥28 days and consider prophylactic antibiotics, antifungals, or anti-PJP treatment (refer to current NCCN guidelines for treatment of cancer-related infections).[a]
	Grade 3 or 4<br>(pain associated with severe weakness; limiting self-care ADLs)	For Grade 3:<br>Hold study drug/study regimen dose until resolution to Grade ≤1.<br>Permanently discontinue study drug/study regimen if Grade 3 imAE does not resolve to Grade ≤1 within 30 days or if there are signs of respiratory insufficiency.<br><br>For Grade 4:<br>- Permanently discontinue study drug/study regimen.	For Grade 3 or 4 (severe or life-threatening events):<br>– Monitor symptoms closely; recommend hospitalization.<br>– Obtain Neurology consult, and complete full evaluation.<br>– Consider, as necessary, discussing with the study physician.<br>– Promptly start IV methylprednisolone 2 to 4 mg/kg/day systemic steroids along with receiving input from Neurology consultant.

			– If after start of IV methylprednisolone at 2 to 4 mg/kg/day there is no improvement within 3 to 5 days, consider start of immunosuppressive therapy such as TNF inhibitors (e.g., infliximab at 5 mg/kg every 2 weeks). Caution: It is important to rule out sepsis and refer to infliximab label for general guidance before using infliximab.<br>– Consider whether patient may require IV IG, plasmapheresis.<br>– Once the patient is improving, gradually taper steroids over ≥28 days and consider prophylactic antibiotics, antifungals, or anti-PJP treatment (refer to current NCCN guidelines for treatment of cancer-related infections).[a]

[a]ASCO Educational Book 2015 “Managing Immune Checkpoint Blocking Antibody Side Effects” by Michael Postow MD.
[b]FDA Liver Guidance Document 2009 Guidance for Industry: Drug Induced Liver Injury – Premarketing Clinical Evaluation.

AChE Acetylcholine esterase; ADL Activities of daily living; AE Adverse event; ALP Alkaline phosphatase test; ALT Alanine aminotransferase; AST Aspartate aminotransferase; BUN Blood urea nitrogen; CT Computed tomography; CTCAE Common Terminology Criteria for Adverse Events; ILD Interstitial lung disease; imAE immune-mediated adverse event; IG Immunoglobulin; IV Intravenous; GI Gastrointestinal; LFT Liver function tests; LLN Lower limit of normal; MRI Magnetic resonance imaging; NCI National Cancer Institute; NCCN National Comprehensive Cancer Network; PJP *Pneumocystis jirovecii* pneumonia (formerly known as *Pneumocystis carinii* pneumonia); PO By mouth; T3 Triiodothyronine; T4 Thyroxine; TB Total bilirubin; TNF Tumor necrosis factor; TSH Thyroid-stimulating hormone; ULN Upper limit of normal.

Infusion-Related Reactions		
**Severity Grade of the Event (NCI CTCAE version 4.03)**	**Dose Modifications**	**Toxicity Management**
**Any Grade**	**General Guidance**	**For Any Grade:**<br>– Manage per institutional standard at the discretion of investigator.<br>– Monitor patients for signs and symptoms of infusion-related reactions (e.g., fever and/or shaking chills, flushing and/or itching, alterations in heart rate and blood pressure, dyspnea or chest discomfort, or skin rashes) and anaphylaxis (e.g., generalized urticaria, angioedema, wheezing, hypotension, or tachycardia).
**Grade 1 or 2**	**For Grade 1:**<br>The infusion rate of study drug/study regimen may be decreased by 50% or temporarily interrupted until resolution of the event.<br>**For Grade 2:**<br>The infusion rate of study drug/study regimen may be decreased 50% or temporarily interrupted until resolution of the event.<br>Subsequent infusions may be given at 50% of the initial infusion rate.	**For Grade 1 or 2:**<br>– Acetaminophen and/or antihistamines may be administered per institutional standard at the discretion of the investigator.<br>– Consider premedication per institutional standard prior to subsequent doses.<br>– Steroids should not be used for routine premedication of Grade ≤2 infusion reactions.
**Grade 3 or 4**	**For Grade 3 or 4:**<br>Permanently discontinue study drug/study regimen.	**For Grade 3 or 4:**<br>– Manage severe infusion-related reactions per institutional standards (e.g., IM epinephrine, followed by IV diphenhydramine and ranitidine, and IV glucocorticoid).

CTCAE Common Terminology Criteria for Adverse Events; IM intramuscular; IV intravenous; NCI National Cancer Institute.

Non–Immune-Mediated Reactions		
**Severity Grade of the Event (NCI CTCAE version 4.03)**	**Dose Modifications**	**Toxicity Management**
**Any Grade**	Note: Dose modifications are not required for AEs not deemed to be related to study treatment (i.e., events due to underlying disease) or for laboratory abnormalities not deemed to be clinically significant.	Treat accordingly, as per institutional standard.
**Grade 1**	No dose modifications.	Treat accordingly, as per institutional standard.
**Grade 2**	Hold study drug/study regimen until resolution to ≤Grade 1 or baseline.	Treat accordingly, as per institutional standard.
**Grade 3**	Hold study drug/study regimen until resolution to ≤Grade 1 or baseline.<br>For AEs that downgrade to ≤Grade 2 within 7 days or resolve to ≤Grade 1 or baseline within 14 days, resume study drug/study regimen administration. Otherwise, discontinue study drug/study regimen.	Treat accordingly, as per institutional standard.
**Grade 4**	Discontinue study drug/study regimen (Note: For Grade 4 labs, decision to discontinue should be based on accompanying clinical signs/symptoms, the Investigator's clinical judgment, and consultation with the Sponsor.).	Treat accordingly, as per institutional standard.

Note: As applicable, for early phase studies, the following sentence may be added: "Any event greater than or equal to Grade 2, please discuss with Study Physician."
AE Adverse event; CTCAE Common Terminology Criteria for Adverse Events; NCI National Cancer Institute.

Appendix 2 Schedule of study procedures follow-up for subjects who have completed durvalumab and tremelimumab treatment and achieved disease control (until confirmed progression of disease) and subjects who have discontinued durvalumab or tremelimumab due to toxicity in the absence of confirmed progression of disease.

Evaluation	Time Since Last Dose							
	**Day (±3)**	**Months (±1 week)**						**12 Months and every 6 Months (±2 weeks)**
	**30**	**2**	**3**	**4**	**6**	**8**	**10**	
Physical examination[a]	x	x	x	x	x	x	x	
Vital signs (temperature, respiratory rate, blood, pressure, pulse)	x	x	x	x	x	x	x	
Weight	x	x	x	x	x	x	x	
Urine hCG or serum βhCG	x							
AE/SAE assessment	x	x	x					
Concomitant medications	x	x	x					
Palliative radiotherapy	As clinically indicated							
ECOG performance status	x	x	x		X (and month 9)			x
Subsequent anti/cancer therapy	x	x	x	x	x	x	x	x
Survival status: phone contact with subjects who refuse to return for evaluations and agree to be contacted		x	x	x	x	x	x	x (every 2 months)
Hematology	x	Every 8-12 weeks, according to investigator's criteria						x
Serum chemistry	x	Every 8-12 weeks, according to investigator's criteria						
Thyroid function tests)TSH, and fT3 and fT4)[b]	x	Every 8-12 weeks, according to investigator's criteria						
SPD-L1 concentration)to assess target engagement)			x					
Tumour assessment (CT or MRI)	For subjects who **achieve disease control following 12 months of treatment**, tumour assessments should be performed every 12 weeks relative to the date of **first infusion** thereafter until confirmed PD by RECIST 1.1 by investigational site review. Please refer to SCHEDULE OF STUDY ASSESSMENTS for timings of confirmatory scans.<br>For subjects who **discontinue study drug due to toxicity (or symptomatic deterioration)**, tumour assessments should be performed relative to the relative to the date of first infusion as follows: every 12 weeks until confirmed PD by RECIST 1.1 by investigational site review. Please refer to SCHEDULE OF STUDY ASSESSMENTS for timings of confirmatory scans.<br>Upon confirmed PD, scans should be conducted according to local standard clinical practice until a new treatment is started (these scans are optional).							

[a] Full physical exam
[b] Free T3 and free T4 will only be measured if TSH is abnormal. They should also be measured if there is clinical suspicion of an adverse event related to the endocrine system.
[c] For patient questionnaires different approaches based on indication and study design.

Appendix 3 Schedule of study procedures: follow-up for subjects who have discontinued durvalumab or tremelimumab treatment due to confirmed progression of disease at the investigator discretion

Evaluation	Time Since Last Dose							
	Day (±3)	Months (±1 week)						12 Months and every 6 Months (±2 weeks)
	30	2	3	4	6	8	10	
Physical examination[a]	x							
Vital signs (temperature, respiratory rate, blood, pressure, pulse)	x							
Weight	x							
AE/SAE assessment	x	x	x					
Concomitant medications	x	x	x					
Palliative radiotherapy	As clinically indicated							
ECOG performance status[b]	x	x	x					
Subsequent anti/cancer therapy	x	x	x	x	x	x	x	x
Survival status: phone contact with subjects who refuse to return for evaluations and agree to be contacted		x	x	x	x	x	x	x (every 2 months)
Urine hCG or serum βhCG	x							
Hematology	x	x	x					
Serum chemistry	x	x	x					
Thyroid function tests)TSH, and fT3 and fT4)[c]			x					
Pharmacokinetic assessment, if applicable			x					
SPD-L1 concentration)to assess target engagement)			x					
Tumour assessment (CT or MRI)	For subjects who continue on MEDI4736 post-confirmed progression at the investigator's discretion (following consultation with the sponsor), tumour assessments should be performed relative to the date of first infusion per SCHEDULE OF STUDY ASSESSMENTS until study drug is stopped.<br>For subjects who discontinue study drug following confirmed progression, scans should be conducted according to local clinical practice until a new treatment is started (these scans are optional).							

[A] Full physical exam

[B] PS to be collected if available at the 2 monthly calls to obtain subsequent anti/cancer therapy and survival status

c Free T3 and free T4 will only be measured if TSH is abnormal. They should also be measured if there is clinical suspicion of an adverse event related to the endocrine system.

[D] For patient questionnaires different approaches based on indication and study design.

Appendix 4 Durvalumab dose calculations

For durvalumab done depending on subject weight:

1. Cohort dose: X mg/kg
2. Subject weight: Y kg
3. Dose for subject: XY mg = X(mg/kg) x Y(kg)
4. Dose to be added into infusion bag:

   Dose (mL) = XY mg / 50 (mg/mL)

   Where 50 mg/mL is durvalumab nominal concentration.

   The corresponding volume of durvalumab should be rounded to the nearest tenth mL (0.1 mL). Dose adjustments for each cycle are only needed for greater than 10% change in weight.

5. The theoretical number of vials required for dose preparation is the next greatest whole number of vials from the following formula:

   Number of vials = Dose (mL) / 10.0 (mL/vial)

Example:

1. Cohort dose: 10 mg/kg
2. Subject weight: 30 kg
3. Dose for subject: 300 mg = 10 (mg/kg) x 30(kg)
4. Dose to be added into infusion bag:

Dose (mL) = 300 mg / 50 (mg/mL) = 6.0 mL

5. The theoretical number of vials required for dose preparation:

Number of vials = 6.0(mL) / 10.0 (mL/vial) = 1 vials

Appendix 5 Durvalumab Dose Volume Calculations

For durvalumab flat dosing:

1. Cohort dose: X g

2. Dose to be added into infusion bag:

Dose (mL) = X g x 1000/50 (mg/mL)

where 50 mg/mL is durvalumab nominal concentration.

The corresponding volume of durvalumab should be rounded to the nearest tenth mL (0.1 mL)

3. The theoretical number of vials required for dose preparation is the next greatest whole number of vials from the following formula:

Number of vials = Dose (mL) / 10.0 (mL/vial)

Example:

1. Cohort dose: 1.5 g

2. Dose to be added into infusion bag:

Dose (mL) = 1.5 g x 1000 / 50 (mg/mL) = 30.0 mL

3. The theoretical number of vials required for dose preparation:

Number of vials = 30.0 (mL) / 10.0 (mL/vial) = 3 vials

GETNE
Grupo Español de Tumores Neuroendocrinos y Endocrinos

Appendix 6 Tremelimumab Dose Calculations

For tremelimumab dosing done depending on subject weight:

1. Cohort dose: X mg/kg
2. Subject weight: Y kg
3. Dose for subject: XY mg = X(mg/kg) x Y(kg)
4. Dose to be added into infusion bag:

   Dose (mL) = XY mg / 20 (mg/mL)

   Where 20 mg/mL is tremelimumab nominal concentration.

   The corresponding volume of tremelimumab should be rounded to the nearest tenth mL (0.1 mL). Dose adjustments for each cycle are only needed for greater then 10% change in weight.

5. The theoretical number of vials required for dose preparation is the next greatest whole number of vials from the following formula:

   Number of vials = Dose (mL) / 20.0 (mL/vial)

   Or

   Number of vials = Dose (mL) / 1.25 (mL/vial)

Example:

1. Cohort dose: 1 mg/kg
2. Subject weight: 30 kg
3. Dose for subject: 30 mg = 1 (mg/kg) x 30(kg)
4. Dose to be added into infusion bag:

Dose (mL) = 30 mg / 20 (mg/mL) = 1.5 mL

5. The theoretical number of vials required for dose preparation:

Number of vials = 1.5(mL) / 20.0 (mL/vial) = 1 vials

or

Number of vials = 1.5(mL) / 1.25 (mL/vial) = 2 vials

Appendix 7 Tremelimumab Dose Volume Calculations

For tremelimumab flat dosing:

1. Cohort dose: X mg

2. Dose to be added into infusion bag:

Dose (mL) = X mg /20 (mg/mL)

where 20 mg/mL is tremelimumab nominal concentration.

The corresponding volume of tremelimumab should be rounded to the nearest tenth mL (0.1 mL)

3. The theoretical number of vials required for dose preparation is the next greatest whole number of vials from the following formula:

Number of vials = Dose (mL) / 20 (mL/vial)

or

Number of vials = Dose (mL) / 1.25 (mL/vial)

Example:

1. Cohort dose: 75 mg

2. Dose to be added into infusion bag:

Dose (mL) = 75 mg / 20 (mg/mL) = 3.8 mL

3. The theoretical number of vials required for dose preparation:

Number of vials = 3.8 (mL) / 20 (mL/vial) = 1 vial

or

Number of vials = 3.8 (mL) / 1.25 (mL/vial) = 3 vials

GETNE
Grupo Español de Tumores Neuroendocrinos y Endocrinos

Appendix 8 Contact Details

Sponsor: GETNE (Spanish Group of Neuroendocrine and Endocrine Tumors)

[redacted]

Coordinating Investigator: Dr. Jaume Capdevila

[redacted]

CRO: [redacted]

Appendix 9 Sites and Investigators Participants

	SITE	Principal Investigator
1 -	[redacted]	
2 -		
3 -		
4 -		
5 -		
6 -		
7 -		
8 -		
9 -		
10 -		
11 -		
12 -		
13 -		
14 -		
15 -		
16 -		
17 -		
18 -		
19 -		
20 -		

GETNE
Grupo Español de Tumores Neuroendocrinos y Endocrinos

Investigational Drug Substance (s)	Durvalumab (MEDI4736) and Tremelimumab
Study Number	ESR 15-11561-61-DUNE
Version Number	3.1
Date	NOVEMBER 30TH, 2017

A PHASE II STUDY OF DURVALUMAB (MEDI4736) PLUS TREMELIMUMAB FOR THE TREATMENT OF PATIENTS WITH ADVANCED NEUROENDOCRINE NEOPLASMS OF GASTROENTEROPANCREATIC OR LUNG ORIGIN (THE DUNE TRIAL)

Sponsor: GETNE

(Spanish Group of Neuroendocrine and Endocrine Tumors)

[redacted]

Coordinating Investigator: Dr. Jaume Capdevila

[redacted]

GETNE
Grupo Español de Tumores
Neuroendocrinos y Endocrinos

SPONSOR'S SIGNATURE PAGE

Study title: A phase II study of durvalumab (MEDI4736) plus tremelimumab for the treatment of patients with advanced neuroendocrine neoplasms of gastroenteropancreatic or lung origin (the DUNE trial).

Study code: ESR 15-11561-61 – DUNE

EudraCT Number: 2016-002858-20

Version number and date: 3.1, November 30th,2017

[redacted]

GETNE Chairman	Signature	Signature date (DD-mm-YYYY)

[redacted]

Coordinating Investigator	Signature	Signature date (DD-mm-YYYY)

GETNE
Grupo Español de Tumores
Neuroendocrinos y Endocrinos

INVESTIGATOR'S SIGNATURE PAGE

Study title: A phase II study of durvalumab (MEDI4736) plus tremelimumab for the treatment of patients with advanced neuroendocrine neoplasms of gastroenteropancreatic or lung origin (the DUNE trial).

Study code: ESR 15-11561-61 – DUNE

EudraCT Number: 2016-002858-20

Version number and date: 3.1, November 30th ,2017

______________________________ ______________________

Principal Investigator's signature | Signature date (DD-mm-YYYY)

____

Investigator's name (capital letters)

PROTOCOL SYNOPSIS

Clinical Protocol ESR 15-11561-61

Study Title: A phase II study of durvalumab (MEDI4736) plus tremelimumab for the treatment of patients with advanced neuroendocrine neoplasms of gastroenteropancreatic or lung origin (the DUNE trial)

Protocol Number: ESR 15-11561-61

EudraCT Number: 2016-002858-20

Clinical Phase: II

Study Duration: 3 years

Investigational Product(s) and Reference Therapy:

Durvalumab will be supplied in glass vials containing 500 mg of liquid solution at a concentration of 50 mg/mL for intravenous (IV) administration.

Tremelimumab is supplied as a sterile solution for IV infusion, filled in 20 mL clear glass vials with a rubber stopper and aluminum seal. Each vial contains 20 mg/mL (with a nominal fill of 20 mL, accounting to 400 mg/vial) of tremelimumab, in an isotonic solution at pH 5.5.

Research Hypothesis

Well-differentiated gastroenteropancreatic and lung neuroendocrine tumors are generally malignancies with a prolonged natural history. However, clinical behavior is heterogeneous and when tumor progression is observed, treatment options are limited. The most used therapy for neuroendocrine tumors management are somatostatin analogs, with clear evidence of antitumor activity demonstrated by two phase III clinical trials in gastroenteropancreatic neuroendocrine tumors. However, even the use in lung carcinoids is quite usual, no antitumoral activity has been demonstrated in prospective clinical trials. In recent years, targeted therapies as sunitinib and everolimus have been approved for pancreatic neuroendocrine tumors and recently everolimus have showed a significant reduction in the risk of progression or death in patients with non-functional neuroendocrine tumors of lung and gastrointestinal origin. After the failure of these therapies, no drugs have demonstrated efficacy. Only Interferon alpha-2b is an option for these patients who have worsening symptoms of carcinoid syndrome while on treatment with somatostatin analogs and have showed some evidence of antitumoral activity in neuroendocrine tumors. The efficacy of Interferon alpha 2-b is not fully understood, but one of its antitumoral mechanisms of action could be related via stimulation of T cells. Tremelimumab and Durvalumab combination could be more efficient drugs to improve immune system activation and could obtain a significantly higher clinical benefit in these patients. We have analyzed presence of tumor-infiltrating lymphocytes (TILS) in these tumor types. We found a high number of TILS that could be activated through Tremelimumab and Durvalumab. In cases of advanced high-grade G3 neuroendocrine carcinomas (NEC), regardless of the site of the primary tumor, combination chemotherapy using cisplatin/etoposide is recommended in first-line setting (provided that the patient has adequate organ function and performance status). There is no established second-line therapy for poorly differentiated neuroendocrine carcinomas of the

gastroenteropancreatic origin.

The programmed death 1 (PD-1) receptor and PD-1 ligands 1 and 2 (PD-L1, PD-L2) play integral roles in immune regulation. Expressed on activated T cells, PD-1 is activated by PD-L1 and PD-L2 expressed by stromal cells, tumor cells, or both, initiating T-cell death and localized immune suppression, potentially providing an immune-tolerant environment for tumor development and growth. Conversely, inhibition of this interaction can enhance local T-cell responses and mediate antitumor activity in nonclinical animal models.

Tremelimumab and Durvalumab would be the first immune combination agents showing efficacy in neuroendocrine neoplasms of different origins.

Objectives:

Primary Objectives:

Primary endpoint for cohorts 1, 2 and 3:

Nine-months clinical benefit rate (CBR) by Response Evaluation Criteria In Solid Tumors (RECIST 1.1), which is defined as the percentage of patients achieving complete response (CR), partial response (PR), or stable disease (SD) at month 9 after durvalumab plus tremelimumab was started.

Primary endpoint for cohort 4:

Nine-months overall survival rate, which is defined as the percentage of patients alive at month 9 after durvalumab plus tremelimumab was started.

Secondary Objective(s):

- Overall response rate (ORR) by irRECIST.
- To assess the duration of response according to irRECIST.
- To assess the median progression-free survival time (PFS) according to irRECIST.
- To assess the safety profile of Durvalumab and Tremelimumab in subjects with advanced neuroendocrine neoplasms.
- To assess the median overall survival (OS) time.
- To assess response status according to irRECIST at 6, 9 and 12 months after start of study treatment.

Exploratory Objective(s):

- To evaluate biochemical response (changes in CgA and NSE levels) and its association with response rate and progression-free survival.
- To assess whether baseline tumor and blood biomarkers may be predictive of response to durvalumab and tremelimumab therapy.
- To explore additional hypotheses related to biomarkers and relationship to durvalumab and tremelimumab efficacy and/or toxicity and neuroendocrine tumors evolution that may

arise from internal or external research activities.

Study Design:

Prospective, multi-center, open label, stratified, exploratory, phase II study evaluating the efficacy and safety of durvalumab plus tremelimumab in different cohorts of patients with neuroendocrine neoplasms.

The study will include patients in four different cohorts:

- **Cohort 1**: Well-moderately differentiated lung neuroendocrine tumors (classically known as typical and atypical carcinoids) after progression to somatostatin analogs and one prior targeted therapy or chemotherapy.
- **Cohort 2**: G1/G2 (WHO grade 1 and 2) gastrointestinal neuroendocrine tumors after progression to somatostatin analogs and one prior targeted therapy.
- **Cohort 3**: G1/G2 (WHO grade 1 and 2) pancreatic neuroendocrine tumors after progression to standard therapies (chemotherapy, somatostatin analogs and target therapy), who have received between two and four prior lines of treatment.
- **Cohort 4**: Neuroendocrine neoplasms (WHO grade 3) of gastroenteropancreatic origin or unknown primary site (excluding lung primary tumors), patients will be treated in second line only, after progression to first-line chemotherapy with a platinum based regimen.

Number of Centers: 20

Number of Subjects: 126 patients: 31 patients for cohorts 1 to 3, and 33 for cohort 4.

Study Population:

Patients with advanced/metastatic, histologically confirmed, grade 1/2 (G1/G2) of the 2010 WHO classification neuroendocrine tumors of the pancreas, gastrointestinal tract and lung origins and grade 3 (G3) of gastroenteropancreatic system or unknown primary site (excluding lung primaries) after progression to previous therapies.

Inclusion Criteria:

For inclusion in the study, patients should fulfill the following criteria:

1. Written informed consent obtained from the subject prior to performing any protocol-related procedures.
2. Age >18 years at time of study entry.
3. Subjects must have histologically confirmed diagnosis of one of the following advanced/metastatic neuroendocrine tumor types:
   a) **Cohort 1**: Well-moderately differentiated neuroendocrine tumors of the lung (mitotic count ≤10 mitoses x 10 HPF), also known as typical and atypical lung carcinoids, that have progressed to prior somatostatin analog therapy and/or one prior targeted therapy or chemotherapy (only one prior systemic therapy, with the exception of patients that have been treated with somatostatin analogues and other systemic

treatment, when two prior treatments are allowed).

b) **Cohort 2**: Well-moderately differentiated G1/G2 (WHO grade 1 and 2) gastrointestinal neuroendocrine tumors after progression to somatostatin analogs and one targeted therapy (prior targeted therapy could be everolimus or a multikinase inhibitor). Prior therapies with interferon alpha-2b or radionucleotide therapy are allowed.

c) **Cohort 3**: Well-moderately differentiated neuroendocrine tumors G1/G2 (WHO grade 1 and 2) from pancreatic origin after progression to standard therapies (chemotherapy, somatostatin analogs and target therapy); patients must be treated with at least two prior systemic treatment lines and a maximum of four previous treatment lines.

d) **Cohort 4**: Neuroendocrine neoplasms (WHO grade 3) of gastroenteropancreatic origin or unknown primary site (excluding lung primary tumors), patients will be treated in second line only, after progression to first-line chemotherapy with a platinum based regimen.

4. For patients included in cohorts 1, 2 and 3: WHO Classification G1/G2 (mitotic count ≤10 mitoses x 10 HPF) lung typical and atypical carcinoids for cohort 1, G1/G2 (Ki67≤20% and mitotic count ≤20 mitoses x 10 HPF) gastrointestinal for cohort 2 (including stomach, small intestine and colorectal origins), G1/G2 (Ki67≤20% and mitotic count ≤20 mitoses x 10 HPF) pancreatic for cohort 3.

5. For patients included in cohort 4: WHO classification G3 (Ki67>20% or mitotic count >20 mitoses x 10 HPF) gastroenteropancreatic neuroendocrine carcinomas (NEC) or liver metastases of G3 NEC of unknown primary site.

6. Subjects must have evidence of measurable disease meeting the following criteria:

   a) In case of more than one target lesion, it should be identified at least 1 lesion of ≥ 1.0 cm in the longest diameter for a non lymph node, or ≥ 1.5 cm in the short-axis diameter for a lymph node, which is serially measurable according to RECIST 1.1 using computerized tomography/magnetic resonance imaging (CT/MRI). If there is only one target lesion and it is a non-lymph node, it should have a longest diameter of ≥ 1.5 cm.

   b) Lesions that have had external beam radiotherapy (EBRT) or loco-regional therapies such as radiofrequency (RF) ablation or liver embolization must show evidence of progressive disease based on RECIST 1.1 to be deemed a target lesion.

   c) Subjects must show evidence of disease progression by radiologic image techniques within 12 months (an additional month will be allowed to accommodate actual dates of performance of scans, i.e., within ≤ 13 months) prior to signing informed consent, according to RECIST 1.1.

7. Eastern Cooperative Oncology Group (ECOG) performance status of 0 or 1.

8. Life expectancy of at least 12 weeks.

9. Adequate normal organ and marrow function as defined below:

- Haemoglobin ≥ 9.0 g/dL.
- Absolute neutrophil count (ANC) ≥ 1.5 x 10^9/L (≥ 1500 per mm^3).
- Platelet count ≥ 100 x 10^9/L (≥100,000 per mm^3).

10. Serum bilirubin ≤ 1.5 x institutional upper limit of normal (ULN). This will not apply to subjects with confirmed Gilbert's syndrome (persistent or recurrent hyperbilirubinemia that is predominantly unconjugated in the absence of hemolysis or hepatic pathology), who will be allowed only in consultation with their physician.

11. AST (SGOT)/ALT (SGPT) ≤ 2.5 x institutional upper limit of normal unless liver metastases are present, in which case it must be ≤ 5x ULN.

12. Serum creatinine CL>40 mL/min by the Cockcroft-Gault formula (Cockcroft and Gault 1976) or by 24-hour urine collection for determination of creatinine clearance:

Males:

$$\text{Creatinine CL (mL/min)} = \frac{\text{Weight (kg)} \times (140 - \text{Age})}{72 \times \text{serum creatinine (mg/dL)}}$$

Females:

$$\text{Creatinine CL (mL/min)} = \frac{\text{Weight (kg)} \times (140 - \text{Age})}{72 \times \text{serum creatinine (mg/dL)}} \times 0.85$$

13. Female subjects must either be of non-reproductive potential (ie, post-menopausal by history: ≥60 years old and no menses for ≥1 year without an alternative medical cause; OR history of hysterectomy, OR history of bilateral tubal ligation, OR history of bilateral oophorectomy) or must have a negative serum pregnancy test upon study entry.

14. Subject is willing and able to comply with the protocol for the duration of the study including undergoing treatment and scheduled visits and examinations including follow up.

Exclusion Criteria:

Subjects who meet any of the following criteria will be excluded from this study:

1. Involvement in the planning and/or conduct of the study.
2. Participation in another clinical study with an investigational product during the last 4 weeks.
3. WHO Classification G3 neuroendocrine neoplasms of lung origin (oat cell/large cell lung cancer).
4. Prior treatment with anti-PDL-1/anti-PD-1 or anti-CTL-4 therapy.
5. Uncontrolled intercurrent illness including, but not limited to, ongoing or active infection, symptomatic congestive heart failure, uncontrolled hypertension, unstable angina pectoris, cardiac arrhythmia, active peptic ulcer disease or gastritis, active bleeding diathesis

including any subject known to have evidence of acute or chronic hepatitis B (e.g., HBsAg reactive), hepatitis C (e.g., HCV RNA [qualitative] is detected) or known history of Human Immunodeficiency Virus (HIV) (HIV 1/2 antibodies), or psychiatric illness/social situations that would limit compliance with study requirements or compromise the ability of the subject to give written informed consent.

6. Known history of previous clinical diagnosis of tuberculosis.
7. Current or prior use of immunosuppressive medication within 28 days before the first dose of durvalumab or tremelimumab, with the exceptions of intranasal and inhaled corticosteroids or systemic corticosteroids at physiological doses, which are not to exceed 10 mg/day of prednisone, or an equivalent corticosteroid.
8. Active or prior documented autoimmune disease within the past 2 years NOTE: Subjects with vitiligo, Grave's disease, or psoriasis not requiring systemic treatment (within the past 2 years) are not excluded.
9. Active or prior documented inflammatory bowel disease (e.g., Crohn's disease, ulcerative colitis).
10. History of allogeneic organ transplant.
11. History of hypersensitivity to durvalumab, tremelimumab or any excipient.
12. Subjects having a diagnosis of immunodeficiency or are receiving systemic steroid therapy or any other form of immunosuppressive therapy within 28 days prior to the first dose of trial treatment.
13. Knowledge of active central nervous system (CNS) metastases and/or carcinomatous meningitis. Subjects with previously treated brain metastases may participate provided they have stable brain metastases [without evidence of progression by imaging confirmed [by magnetic resonance imaging (MRI) if MRI was used at prior imaging, or confirmed by computed tomography (CT) imaging if CT used at prior imaging] for at least four weeks prior to the first dose of trial treatment; also, any neurologic symptoms must have returned to baseline], have no evidence of new or enlarging brain metastases,and have not used steroids for brain metastases for at least 7 days prior to trial treatment. This exception does not include carcinomatous meningitis, as subjects with carcinomatous meningitis are excluded regardless of clinical stability.
14. Receipt of live attenuated vaccination within 30 days prior to study entry or within 30 days of receiving durvalumab or tremelimumab. Note: The killed virus vaccines used for seasonal influenza vaccines for injection are allowed; however intranasal influenza vaccines (e.g., FluMist®) are live attenuated vaccines, and are not allowed.
15. Subjects having known history of, or any evidence of interstitial lung disease or active, noninfectious pneumonitis.
16. Any prior Grade ≥3 immune-related adverse event (irAE) while receiving any previous immunotherapy agent, or any unresolved irAE >Grade 1.
17. Subjects who have received any anti-cancer treatment within 21 days or any

investigational agent within 30 days prior to the first dose of study drug and should have recovered from any toxicity related to previous anti-cancer treatment. This does not apply to the use of somatostatin analogues for symptomatic therapy.

18. Major surgery within 3 weeks prior to the first dose of study drug.
19. Subjects having > 1+ proteinuria on urine dipstick testing will undergo 24h urine collection for quantitative assessment of proteinuria. Subjects with urine protein ≥ 1 g/24h will be ineligible.
20. Significant cardiovascular impairment: history of congestive heart failure greater than New York Heart Association (NYHA) Class II, unstable angina; myocardial infarction or stroke within 6 months of the first dose of study drug, or cardiac arrhythmia requiring medical treatment.
21. Mean QT interval corrected for heart rate (QTc) ≥470 ms calculated from 3 electrocardiograms (ECGs) using Fredericia's Correction.
22. Bleeding or thrombotic disorders or use of anticoagulants, such as warfarin, or similar agents requiring therapeutic international normalized ratio (INR) monitoring. Treatment with low molecular weight heparin (LMWH) is allowed.
23. Active hemoptysis (bright red blood of at least 0.5 teaspoon) within 3 weeks prior to the first dose of study drug.
24. Patients with tumoral disease in the head and neck region, such as paratracheal or periesophageal lymph node involvement, or with infiltration of structures in the digestive tract, or vascular pathways that represent a risk of increased bleeding.
25. Patients of cohort 1 with neuroendocrine tumors of pulmonary origin or pulmonary metastases with evidence of active bleeding.
26. Patients with evidence of digestive bleeding.
27. Active infection (any infection requiring treatment).
28. Active malignancy (except for differentiated thyroid carcinoma, or definitively treated melanoma in-situ, basal or squamous cell carcinoma of the skin, or carcinoma in-situ of the cervix) within the past 24 months.
29. Female patients who are pregnant or breastfeeding or male or female patients of reproductive potential who are not willing to employ highly effective birth control from screening to 180 days after the last dose of durvalumab + tremelimumab combination therapy or 90 days after the last dose of durvalumab monotherapy, whichever is the longer time period.
30. Documented active alcohol or drug abuse.
31. Patients with a prior history of non-compliance with medical regimens.
32. Any condition that, in the opinion of the investigator, would interfere with evaluation of study treatment or interpretation of patient safety or study results.

Investigational Product(s), Dose, and Mode of Administration:

Durvalumab, 1500 mg Q4W (equivalent to 20 mg/kg Q4W) for 12 months in patients ≥ 30kg.

Tremelimumab 75 mg Q4W (equivalent to 1 mg/kg Q4W) for up to 4 doses/cycles in patients ≥ 30kg.

Weight-based dosing should be used for patients <30 kg durvalumab 20 mg/kg Q4 and tremelimumab 1 mg/kg Q4.

Study Assessments and Criteria for Evaluation:

Safety Assessments:

The safety objective of this trial is to characterize the safety and tolerability of durvalumab plus tremelimumab in subjects with advanced neuroendocrine neoplasms. The primary safety analysis will be based on subjects who experienced toxicities as defined by CTCAE, v4.03 The attribution to drug, time-of-onset, duration of the event, its resolution, and any concomitant medications administered will be recorded. AEs will be analyzed including but not limited to all AEs, SAEs, fatal AEs, and laboratory changes. Furthermore, specific immune-related adverse events (irAEs) will be collected and designated as immune related events of clinical interest (ECIs).

Efficacy Assessments:

The primary efficacy objective of this study is to evaluate the anti-tumor activity of durvalumab plus tremelimumab in subjects with advanced neuroendocrine neoplasms. 9-months clinical benefit rate by Response Evaluation Criteria in Solid Tumors. (RECIST 1.1), which is defined as the percentage of patients achieving complete response, partial response (PR), or stable disease (SD) at month 9 after durvalumab plus tremelimumab was started for cohorts 1, 2 and 3, and 9-months overall survival rate, which is defined as the percentage of patients alive at 9 months after durvalumab plus tremelimumab was started will be used as the primaries endpoints per RECIST 1.1 assessed by the central imaging vendor.

Pharmacodynamic / Pharmacokinetic Assessments (if applicable):

Correlative blood samples for pharmacodynamics studies will be obtained during the course of the trial.

Archival tumor tissue will be required at baseline.

Statistical Methods and Data Analysis:

Strategic decisions about future development of immune therapy in neuroendocrine tumors will be available after the results of this trial.

Summary tables (descriptive statistics and frequency tables) will be provided for all baseline variables, efficacy variables, and safety variables, as appropriate. Continuous variables will be summarized with descriptive statistics (mean, standard deviation, range, and median). Ninety-five (95) percent confidence highest probability density (HPD) credible intervals (95% CI) may also be presented, as appropriate. Frequency counts and percentage of subjects within each category will

be provided for categorical data.

The primary efficacy analysis will be performed using the binomial test procedure. Missing data will be treated using statistical multiple random imputation.

Secondary endpoints will be summarized with descriptive statistics. Continuous variables will be summarized with n, mean, standard deviation, and range, frequency counts and percentage of subjects within each category will be provided for categorical data. Multivariate regression models will be used to study relations between explanatory variables and primary endpoint. Survival analysis will be performed to analyze PFS, Kaplan-Meier curves will be presented and possible comparisons will be tested using the log-rank test or the Cox proportional hazard model for multivariate analysis, hazard ratios (HR) and their 95% confidence interval (CI95%) will be provided. Patients with lost of follow-up or treatment discontinuation will be included in the final analysis of primary endpoint if they have at least one tumor evaluation and considered as censored data for survival endpoints.

R software version 3.2.1 will be used for all analysis.

Sample Size Determination:

Sample size has been calculated using one-sample Superiority test (function One Sample Proportion. (NIS) of the Trial Size package of R software). According to previous knowledge, it is estimated that the reference value for the likelihood to be progression-free at 9 months is 30% and we expect an increase of 20% with a superiority margin of 10%. With a unilateral alpha level of 5% and 80% power, we estimate to include 28 patients per group, with an expected lost to follow-up rate of 10%, a final sample size in each 1, 2 and 3 cohort will include 31 patients.

For cohort 4, and according to previous knowledge, it is estimated that the reference proportion of patients being alive at 9 months is 13% and we expect an increase of 10% with a superiority margin of 5%. With a unilateral alpha level of 5% and 80% power, we estimate to include 30 patients per group, with an expected lost to follow-up rate of 10%, a final sample size will include 33 patients.

Summarizing, total sample size will include 126 patients: 31 patients for cohorts 1 to 3, and 33 for cohort 4.

SCHEDULE OF STUDY ASSESSMENTS (according to section 8)

Phase	Screening Phase		Treatment Phase								End of treatment [r]	Follow-up [s]
Period	Screening [,b]	Baseline[a,b]	Study Treatment									
Visit	V1<br>-28 to -2	V2<br>Day -3 to C1D1	V3<br>C1D1	V4<br>C1D15	V5<br>C2D1<br>(C1D28)	V6<br>C2D15	V7<br>C3D1<br>(C2D28)	V8<br>C3D15	V9<br>C4D1<br>(C3D28)	V10-V16<br>C5D1<br>C12D1		
Informed consent	X											
Inclusion/Exclusion	X	X										
Allocation		X										
Medical history and demographic Data	X											
ECOG PS[c]	X	X		X	X	X	X	X	X	X	X	X
Prior Therapies for neuroendocrine neoplasms	X											
Vitals signs[d]	X		X	X	X	X	X	X	X	X	X	X
Physical exam[f]	X	X[e]		X	X	X	X	X	X	X	X	X
12/lead ECG[g]	X		Only when clinically indicated									
Biochemistry and hematology[h]	X	X[h]		X	X	X	X	X	X	X	X	X
Hepatitis B and C; HIV	X											
Thyroid function test (TSH and fT3 and fT4)[i]	X			X	X	X	X	X	X	X	X	X
Urinalysis[j]	X			X	X	X	X	X	X	X	X	X
Pregnancy test[k]	X		Only when clinically indicated									
Durvalumab administration			X		X		X		X	X		
Tremelimumab administration			X		X		X		X			
Tumor assessments (CT/MRI)[l]	X		CT/MRI of chest/abdomen/pelvis and other areas of known disease at screening plus any areas of newly suspected disease should be performed every 12 weeks until confirmed PD.									
Octreoscan / PET/CT[m]	X											
Survival[n]					X		X		X	X	X	X
Biomarkers[o]		X			X						X<br>(end of treatment/ progression)	
Archival tumor block or slides[p]		X										
Concomitant medication	X	X	X	X	X	X	X	X	X	X	X	X
AEs/SAEs[q]	X	X	X	X	X	X	X	X	X	X	X	X

AEs = adverse events, BP = blood pressure, CT = computerized tomography, ECG = electrocardiogram, ECOG = Eastern Cooperative Oncology Group, HR = heart rate, MRI = magnetic resonance imaging, PET = positron-emission tomography, RECIST 1.1 = Response Evaluation Criteria in Solid Tumors , RR = respiratory rate, SAEs = serious adverse events, surg = surgical, 99m-Tc MDP = 99m-technetium-methylene diphosphonate, w/in = within.

a) 72 hours before Cycle 1 Day 1 (C1D1). The **baseline assessment can be performed on C1D1, prior to treatment.** Informed consent may be taken up to 4 weeks prior to C1D1.

b) Efforts should be made to conduct study visits on the day scheduled (± 1 day). Clinical laboratory assessments may be conducted anytime within 72 hours prior to the scheduled visit.

c) ECOG will be performed at the Screening and Baseline Visits and at every subsequent treatment visit thereafter.

d) Assessments will include vital signs (supine BP, HR, RR, and body temperature), weight, and height. Height will be measured at the Screening Visit only. Elevated BP (≥140/90 mmHg) should be confirmed by 3 measurements (at least 5 minutes apart). If systolic BP is ≥160 mmHg or diastolic BP ≥100 mmHg, BP should be confirmed by repeat measurements after an hour. Subjects will have their blood pressure and pulse measured before, during, and after the infusion at the following times (based on a 60-minute infusion):

- At the beginning of the infusion (at 0 minutes)
- At 30 minutes during the infusion (±5 minutes)
- At the end of the infusion (at 60 minutes ±5 minutes)
- In the 1 hour observation period post-infusion: 30 and 60 minutes after the infusion (ie, 90 and 120 minutes from the start of the infusion) (±5 minutes) – for the first infusion only and then for subsequent infusions as clinically indicated

If the infusion takes longer than 60 minutes, then blood pressure and pulse measurements should follow the principles as described above or more frequently if clinically indicated. According to section 8.2.4.

e) Required if screening physical examination was performed > 7 days prior C1D1.

f) A comprehensive physical examination (including a neurological examination) will be performed at the Screening or Baseline Visit, on each visit of the Treatment phase, and at the off-treatment assessment. A symptom-directed physical examination will be performed on Cycle 1/Day 1 and at any time during the study, as clinically indicated.

g) Single 12-lead ECG. Subjects must be in the recumbent position for a period of 5 minutes prior to the ECG.

h) Assessments scheduled may be performed within 72 hours prior to the visit. Assessments during follow up are made every 8-12 weeks, according to investigator's criteria. According to section 8.2.5.

i) Free T3 and free T4 will only be measured if TSH is abnormal. They should also be measured if there is clinical suspicion of an adverse event related to the endocrine system. Assessments during follow up are made every 8-12 weeks, according to investigator's criteria.

j) Assessments during follow up are made every 8-12 weeks, according to investigator's criteria.

k) A serum or urine pregnancy test will be performed at the Screening Visit, at the Baseline Visit (or within 72 hours prior to the first dose of study medication)

l) Screening tumor assessments using CT of the chest/abdomen/pelvis and other areas of known disease or newly suspected disease should be performed within 28 days prior to C1D1. Scans of the abdomen, pelvis and other areas of the body may be done with MRI instead of CT, but evaluation of the chest should be done with CT. CT scans should be performed with oral and iodinated IV contrast and MRI scans with IV gadolinium chelate unless there is a medical contraindication to contrast. If iodinated IV contrast is contraindicated, chest CT should be done without IV contrast.

m) Somatostatin receptor scintigraphy (octreoscan) or PET-CT should be performed and/or available within the previous 6 months before C1D1, only on cohort 1-3. It is not applicable on cohort 4.

n) Survival data will be collected every 4 weeks until end of treatment phase is declared. All anticancer therapies will be collected. Survival data and other cancer treatments received will be collected every 6 months until close of the study database. The study sponsor may elect to discontinue survival follow-up.

o) Collection of blood sample to obtain plasma, serum, or other components to be used for biomarker studies. Samples will be obtained at baseline, Cycle 2/Day 1, and at end-of-treatment visit.

p) All subjects will have collection of most recent archived, tumor-biopsy sections for identification of predictive biomarkers.

q) Throughout the study from the signature of Informed Consent. SAE irrespective of relationship to study treatment must be reported as soon as possible but not later than one business day. AEs and concomitant meds collected 28 days from last dose.

r) See section 8.1.3, APPENDIX 2 and APPENDIX 3.

s) See section 8.1.3, APPENDIX 2 and APPENDIX 3.

LIST OF TABLES 19

LIST OF APPENDICES 20

ABBREVIATIONS AND DEFINITION OF TERMS 21

1. INTRODUCTION 24
1.1 Disease Background 24
1.1.1 Immunotherapies 26
1.1.2 Durvalumab 26
1.1.3 Tremelimumab 27
1.1.4 Durvalumab in combination with tremelimumab 28
1.2 Research hypothesis 28
1.3 Rationale for conducting this study 29
1.3.1 Durvalumab + tremelimumab combination therapy dose rationale 30
1.3.2 Rationale for 4 cycles of combination therapy followed by durvalumab monotherapy 31
1.4 Benefit/risk and ethical assessment 32
1.4.1 Potential benefits 32
1.4.1.1 Durvalumab 32
1.4.1.2 Tremelimumab 32
1.4.1.3 Durvalumab + tremelimumab 33
1.4.2 Potential risks 33
1.4.2.1 Durvalumab 33
1.4.2.2 Tremelimumab 34
1.4.2.3 Durvalumab + tremelimumab 35
1.4.2.4 Fixed Dosing for durvalumab and tremelimumab 36

2. STUDY OBJECTIVES 37
2.1 Primary objective(s) 37
2.2 Secondary objective(s) 37
2.3 Exploratory objective(s) 37

3. STUDY DESIGN 38
3.1 Overview of study design 38
3.2 Study schema 38
3.3 Study Oversight for Safety Evaluation 39

4. PATIENT SELECTION, ENROLLMENT, ALLOCATION, RESTRICTIONS, DISCONTINUATION AND WITHDRAWAL 40

4.1 Inclusion criteria 40
4.2 Exclusion criteria 42
4.3 Withdrawal of Subjects from Study Treatment and/or Study 44
4.4 Replacement of subjects 45

5. INVESTIGATIONAL PRODUCT(s) 45
5.1 Durvalumab and tremelimumab 45
5.1.1 Formulation/packaging/storage 45
5.2 Dose and treatment regimens 46
5.2.1 Treatment regimen 46
5.2.2 Duration of treatment and criteria for retreatment 46
5.2.3 Study drug preparation of durvalumab and tremelimumab 47
5.2.4 Monitoring of dose administration 49
5.2.5 Accountability and dispensation 50
5.2.6 Disposition of unused investigational study drug 50

6. TREATMENT PLAN 51
6.1 Subject enrollment and allocation 51
6.1.1 Procedures for allocation 51
6.1.2 Procedures for handling subjects incorrectly enrolled 51
6.2 Dose Modification and Toxicity Management 51
6.2.1 Durvalumab and tremelimumab 51

7. RESTRICTIONS DURING THE STUDY AND CONCOMITANT TREATMENT(s) 52
7.1 Restrictions during the study 52
7.2 Concomitant treatment(s) 53
7.2.1 Permitted concomitant medications 53
7.2.2 Excluded Concomitant Medications 54

8. STUDY PROCEDURES 55
8.1 Schedule of study procedures 55
8.1.1 Screening Phase 55
8.1.2 Treatment Phase 56
8.1.3 End of Treatment 59
8.2 Description of study procedures 59
8.2.1 Medical history and physical examination, electrocardiogram, weight and vital signs 59
8.2.2 Physical examination 59
8.2.3 Electrocardiograms 60

8.2.4 Vital signs 60
8.2.5 Clinical laboratory tests 61
8.3 Biological sampling procedures 62
8.3.1 Biomarker/Pharmacodynamic sampling and evaluation methods 62
8.3.2 Estimate of volume of blood to be collected 64
8.3.3 Archival tumor samples 64
8.3.3.1 Archival tumor samples 64
8.3.4 Withdrawal of informed consent for donated biological samples 64

9. DISEASE EVALUATION AND METHODS 65
Primary Objective 65
Secondary objectives (irRECIST) 65
9.1 Efficacy variable 66

10. ASSESSMENT OF SAFETY 66
10.1 Safety Parameters 66
10.1.1 Definition of adverse events 66
10.1.2 Definition of serious adverse events 67
10.1.3 Durvalumab + tremelimumab adverse events of special interest 67
10.2 Assessment of safety parameters 69
10.2.1 Assessment of severity 69
10.2.2 Assessment of relationship 70
10.3 Recording of adverse events and serious adverse events 70
10.3.1 Study recording period and follow-up for adverse events and serious adverse events 71
10.3.2 Reporting of serious adverse events 72
10.3.2.1 Reporting of deaths 72
10.3.3 Other events requiring reporting 73
10.3.3.1 Overdose 73
10.3.3.2 Hepatic function abnormality 73
10.3.3.3 Pregnancy 74

11. STATISTICAL METHODS AND SAMPLE SIZE DETERMINATION 75
11.1. Description of analysis sets 75
11.2. Methods of statistical analyses 76
11.3. Determination of sample size 76

12. ETHICAL AND REGULATORY REQUIREMENTS 77
12.1. Ethical conduct of the study 77

12.2. Ethics and regulatory review 77
12.3. Informed consent 77
12.4. Changes to the protocol and informed consent form 78
12.5. Audits and inspections 78
12.6. Confidentiality 78
12.7. Insurance 79
12.8. Publications 79
13.1. Training of study site personnel 80
13.2. Monitoring of the study 80
13.3. Source data 80
13.4. Retention of Records 81
13.5. Quality assurance 81
13.6. Study timetable and end of study 81

14. DATA MANAGEMENT 82
14.1. Study governance and oversight 82

15. INVESTIGATIONAL PRODUCT AND OTHER TREATMENTS 82
15.1. Identity of investigational product(s) 82

16. LIST OF REFERENCES 83
Appendix 1 Dosing Modification and Toxicity Management Guidelines for Immune-Mediated, Infusion-Related, and Non-Immune–Mediated Reactions (MEDI4736 Monotherapy or Combination Therapy With Tremelimumab or Tremelimumab Monotherapy), 1 November 2017 version. 91
Appendix 2 Schedule of study procedures follow-up for subjects who have completed durvalumab and tremelimumab treatment and achieved disease control (until confirmed progression of disease) and subjects who have discontinued durvalumab or tremelimumab due to toxicity in the absence of confirmed progression of disease. 115
Appendix 3 Schedule of study procedures: follow-up for subjects who have discontinued durvalumab or tremelimumab treatment due to confirmed progression of disease at the investigator discretion 116
Appendix 4 Durvalumab dose calculations 116
Appendix 5 Durvalumab Dose Volume Calculations 117
Appendix 6 Tremelimumab Dose Calculations 118
Appendix 7 Tremelimumab Dose Volume Calculations 119
Appendix 8 Contact Details 120

LIST OF TABLES

Table 1. Groups of neuroendocrine neoplasms following the WHO classification

Table 2. Effective methods of contraception (2 methods must be used)

Table 3. Prohibited and Rescue Medications

Table 4. Hematology Laboratory Tests

Table 5. Clinical chemistry (Serum or Plasma) Laboratory Tests

Table 6. Urinalysis Tests[a]

Tabla 7. List of investigational products for this study.

LIST OF APPENDICES

Appendix 1. Dosing Modification and Toxicity Management Guidelines for Immune-Mediated, Infusion-Related, and Non-Immune–Mediated Reactions

Appendix 2. Schedule of study procedures: follow-up for subjects who have completed durvalumab and tremelimumab treatment and achieved disease control (until confirmed progression of disease) and subjects who have discontinued durvalumab or tremelimumab due to toxicity in the absence of confirmed progression of disease.

Appendix 3. Schedule of study procedures: follow-up for subjects who have discontinued nivolumab or tremelimumab treatment due to confirmed progression of disease at the investigator discretion.

Appendix 4. Durvalumab DOSE CALCULATIONS

Appendix 5. Durvalumab VOLUME CALCULATIONS

Appendix 6. Tremelimumab DOSE CALCULATIONS

Appendix 7. Tremelimumab VOLUME CALCULATIONS

Appendix 8. Contact details.

Appendix 9. Sites and Investigators participants.

ABBREVIATIONS AND DEFINITION OF TERMS

The following abbreviations and special terms are used in this study Clinical Study Protocol.

Abbreviation	Explanation or special term
AchE	Acetylcholinesterase
ADA	Anti-drug antibody
AE	Adverse event
AESI	Adverse event of special interest
ALK	Anaplastic lymphoma kinase
ALT	Alanine aminotransferase
APF12	Proportion of patients alive and progression free at 12 months from randomization
AST	Aspartate aminotransferase
AUC	Area under the curve
AUC0-28day	Area under the plasma drug concentration-time curve from time zero to Day 28 post-dose
AUCss	Area under the plasma drug concentration-time curve at steady state
BICR	Blinded Independent Central Review
BoR	Best objective response
BP	Blood pressure
C	Cycle
CD	Cluster of differentiation
CI	Confidence interval
CL	Clearance
C_{max}	Maximum plasma concentration
$C_{max,ss}$	Maximum plasma concentration at steady state
CR	Complete response
CSA	Clinical study agreement
CSR	Clinical study report
CT	Computed tomography
CTCAE	Common Terminology Criteria for Adverse Event
CTLA-4	Cytotoxic T–lymphocyte-associated antigen 4
$C_{trough,ss}$	Trough concentration at steady state
CXCL	Chemokine (C-X-C motif) ligand
DoR	Duration of response
EC	Ethics Committee, synonymous to Institutional Review Board and Independent Ethics Committee
ECG	Electrocardiogram
ECOG	Eastern Cooperative Oncology Group
eCRF	Electronic case report form
EdoR	Expected duration of response
EGFR	Epidermal growth factor receptor
EU	European Union
FAS	Full analysis set

FDA	Food and Drug Administration
GCP	Good Clinical Practice
GI	Gastrointestinal
GMP	Good Manufacturing Practice
hCG	Human chorionic gonadotropin
HIV	Human immunodeficiency virus
HR	Hazard Ratio
IB	Investigator’s Brochure
ICF	Informed consent form
ICH	International Conference on Harmonisation
IDMC	Independent Data Monitoring Committee
IFN	Interferon
IgE	Immunoglobulin E
IgG	Immunoglobulin G
IHC	Immunohistochemistry
IL	Interleukin
ILS	Interstitial lung disease
IM	Intramuscular
IMT	Immunomodulatory therapy
IP	Investigational product
irAE	Immune-related adverse event
IRB	Institutional Review Board
irRECIST	Immune-related Response Evaluation Criteria in Solid Tumors
ITT	Intent-to-Treat
IV	Intravenous
IVRS	Interactive Voice Response System
IWRS	Interactive Web Response System
mAb	Monoclonal antibody
MDSC	Myeloid-derived suppressor cell
MedDRA	Medical Dictionary for Regulatory Activities
MHLW	Minister of Health, Labor, and Welfare
miRNA	Micro-ribonucleic acid
MRI	Magnetic resonance imaging
NCI	National Cancer Institute
NE	Not evaluable
NSCLC	Non–small-cell lung cancer
OAE	Other significant adverse event
ORR	Objective response rate
OS	Overall survival
PBMC	Peripheral blood mononuclear cell
PD	Progressive disease
PD-1	Programmed cell death 1
PD-L1	Programmed cell death ligand 1
PD-L2	Programmed cell death ligand 2

PDx	Pharmacodynamic(s)
PFS	Progression-free survival
PFS2	Time to second progression
PGx	Pharmacogenetic research
PK	Pharmacokinetic(s)
PR	Partial response
q2w	Every 2 weeks
q3w	Every 3 weeks
q4w	Every 4 weeks
q6w	Every 6 weeks
q8w	Every 8 weeks
QTcF	QT interval corrected for heart rate using Fridericia's formula
RECIST 1.1	Response Evaluation Criteria in Solid Tumors, version 1.1
RNA	Ribonucleic acid
RR	Response rate
RT-QPCR	Reverse transcription quantitative polymerase chain reaction
SAE	Serious adverse event
SAP	Statistical analysis plan
SAS	Safety analysis set
SD	Stable disease
SNP	Single nucleotide polymorphism
SoC	Standard of Care
sPD-L1	Soluble programmed cell death ligand 1
T_3	Triiodothyronine
T_4	Thyroxine
TSH	Thyroid-stimulating hormone
ULN	Upper limit of normal
US	United States

1. INTRODUCTION

Neuroendocrine neoplasms constitute a heterogeneous group of tumors derived from the Kultchitzky cells of the diffuse neuroendocrine system located in the gastrointestinal tract (origin of classical carcinoid tumors), pancreatic islet cells (origin of pancreatic endocrine tumors) and lung (origin of typical and atypical carcinoids). Advanced disease is common at diagnosis leading to limited options of curative strategies, so general prognosis is poor. Neuroendocrine neoplasms are considered rare diseases, however, taken all together into account and the natural history of well-differentiated tumors, their prevalence is increasing. Systemic treatment options have been limited for several years. However, in the last decade, the better knowledge of molecular biology of neuroendocrine neoplasms has increased the interest to develop targeted agents in this field, changing the scenario of possible therapies for advanced disease. Even though, treatment options are still limited.

Immune balance has always been related with neuroendocrine neoplasms. Prolonged stabilizations have often been described without treatment and initial immune modulators, such as interferon, have demonstrated antitumoral effects in these tumors. Recent advances in immunotherapy have permitted the development of new targeted agents with higher efficacy and lower toxicity that opens a new treatment option for patients with advanced neuroendocrine tumors.

1.1 Disease Background

Well-differentiated gastroenteropancreatic and lung neuroendocrine tumors are generally malignancies with a prolonged natural history. However, clinical behavior is heterogeneous and when tumor progression is observed, treatment options are limited. Although advanced neuroendocrine neoplasms represent less than 2% of all gastrointestinal cancers, they constitute the second most prevalent advanced tumor of the gastrointestinal tract after colorectal cancer (Yao et al, 2008). The biological behavior of neuroendocrine neoplasms depends mainly on their histological characteristics, defined by the differentiation grade and Ki67 expression. The most recent 2010 WHO classification divides neuroendocrine neoplasms in three groups regarding Ki67 value and mitotic count: neuroendocrine tumors that include grade 1 (G1) and grade 2 (G2) with Ki67 value ≤20% and mitotic count ≤20 mitoses x 10 HPF, and neuroendocrine carcinomas that include grade 3 (G3) with Ki67>20% and/or mitotic count >20 mitoses x 10 HPF.

Table 1. Groups of neuroendocrine neoplasms following the WHO classification

	Lung and Thymus	GEP-NETs	
Grade	Mitotic count (10 HPF)	Mitotic count (10 HPF)	Ki-67 (%)
G1	< 2	<2	≤2
G2	2-10	2-20	3-20
G3	>10	>20	>20

The medical treatment of advanced well-moderately differentiated (G1/G2) neuroendocrine neoplasms has included somatostatin analogs, interferon and cytotoxic agents. The lack of effectiveness of conventional cytotoxic agents has prompted exploration of new targeted drugs exploiting phenotypical features of neuroendocrine neoplasms. Neuroendocrine neoplasms are characterized by being remarkably vascular and expressing several growth factors, including vascular endothelial growth factor (VEGF), platelet-derived growth factor (PDGF), insulin-like

growth factor 1 (IGF-1), basic fibroblast growth factor (bFGF), and transforming growth factor (TGF)-α and -β. In addition, expression of several receptors of these growth factors and ligands has been described, including stem cell factor receptor (c-KIT), epidermal growth factor receptors (EGFR), VEGF receptors (VEGFR)-2 and 3, IGF receptors (IGF-R), and PDGF receptors (PDGFR). The (over)expression of some of these factors has been associated with poor prognosis and decreased progression-free survival (PFS), as well as with tumor growth, aggressiveness, and disease extent in patients with gastroenteropancreatic neuroendocrine neoplasms. Several targeted agents have been studied in the treatment of neuroendocrine neoplasms including antiangiogenic compounds (such as bevacizumab), inhibitors of multiple receptors with kinase activity (such as sunitinib), and inhibitors of intracellular downstream effector proteins such as the mammalian target of rapamycin (mTOR) everolimus. Three phase III studies with everolimus and sunitinib have demonstrated efficacy in G1/G2 neuroendocrine tumors of the gastroenteropancreatic system becoming new treatment options in the management of these patients (Raymond et al 2011; Yao et al 2011; Yao et al 2015). Everolimus has also demonstrated efficacy in G1/G2 lung neuroendocrine tumors (typical and atypical carcinoids), becoming the first drug that has demonstrated a significant increase in progression-free survival in this setting (Yao et al 2015).

For advanced high-grade neuroendocrine carcinomas (G3), regardless of the site of the primary tumor, combination chemotherapy using cisplatin-based regimens is recommended early (provided that the patient has adequate organ function and performance status). Responses are frequently seen, however impact in overall survival is limited and life expectancy of these patients is around 12-16 months. There is no established second-line therapy for G3 neuroendocrine carcinomas of the gastroenteropancreatic system.

Immune responses directed against tumors are one of the body's natural defense against the growth and proliferation of cancer cells. However, over time and under pressure from immune attack, cancers develop strategies to evade immune-mediated killing allowing them to develop unchecked. One such mechanism involves upregulation of surface proteins that deliver inhibitory signals to cytotoxic T cells. Programmed cell death ligand 1 (PD-L1) is one such protein, and is upregulated in a broad range of cancers with a high frequency, with up to 88% expression in some tumor types. In a number of these cancers, including lung (Mu et al, 2011), renal (Krambeck et al, 2007), pancreatic (Nomi et al, 2007; Loos et al, 2008), ovarian cancer (Hamanishi et al, 2007), and hematologic malignancies (Andorsky et al, 2011; Brusa et al, 2013) tumor cell expression of PD-L1 is associated with reduced survival and an unfavorable prognosis.

Programmed cell death ligand 1 is part of a complex system of receptors and ligands that are involved in controlling T-cell activation. PD-L1 acts at multiple sites in the body to help regulate normal immune responses and is used by tumors to help evade detection and elimination by the host immune system tumor response. In the lymph nodes, PD-L1 on antigen-presenting cells binds to PD-1 or CD80 on activated T cells and delivers an inhibitory signal to the T cell (Keir et al, 2008; Park et al, 2010). This results in reduced T-cell activation and fewer activated T cells in circulation. In the tumor microenvironment, PD-L1 expressed on tumor cells binds to PD-1 and CD80 on activated T cells reaching the tumor. This delivers an inhibitory signal to those T cells, preventing them from killing target cancer cells and protecting the tumor from immune elimination (Zou and Chen, 2008).

Immune responses directed against tumors are one of the body's natural defenses against the

growth and proliferation of cancer cells. T cells play a critical role in antitumor immunity and their infiltration and activity have been linked to improved prognosis in a number of cancers (Pagès et al, 2010; Suzuki et al, 2011). Immune evasion, primarily through suppression of T-cell activity, is now recognized as one of the hallmarks of cancer. Such evasion can occur via a range of mechanisms including production of suppressive cytokines such as IL-10, secretion of chemokines and growth factors that recruit and sustain suppressive regulatory T cells (Tregs) and inflammatory macrophages, and expression of inhibitory surface molecules such as B7-H1. Tumor types characterized as being responsive to immunotherapy-based approaches include melanoma, renal cell carcinoma (RCC), bladder cancer , and malignant mesothelioma (Bograd et al, 2011). Inhibition of CTLA-4 signaling is a validated approach to cancer therapy, as shown by the approval in 2011 of ipilimumab for the treatment of metastatic melanoma based on statistically significant and clinically meaningful improvement in OS (Robert et al, 2011).

In general, tumor response rates to anti-CTLA-4 therapy are low (~10%). However, in patients who respond, the responses are generally durable, lasting several months even in patients with aggressive tumors such as refractory metastatic melanoma. Because these agents work through activation of the immune system and not by directly targeting the tumor, responses can occur late and some patients may have perceived progression of their disease in advance of developing disease stabilization or a tumor response. In some cases, early growth of pre-existing lesions or the appearance of new lesions may have been due to immune-cell infiltration into the tumor and not due to proliferation and extension of neoplastic cells, per se (Wolchok et al, 2009). Overall, although the impact on conventionally-defined PFS can be small, durable response or stable disease seen in a proportion of patients can lead to significant prolongation of OS. The melanoma data with ipilimumab clearly demonstrate that a small proportion of patients with an objective response had significant prolongation of OS, supporting the development of this class of agents in other tumors. Although Phase 2 and Phase 3 studies of tremelimumab in metastatic melanoma did not meet the primary endpoints of response rate and OS, respectively, the data suggest activity of tremelimumab in melanoma (Ribas et al, 2013). In a large Phase 3 randomized study comparing tremelimumab with dacarbazine (DTIC)/temozolomide in patients with advanced melanoma, the reported median OS in the final analysis was 12.58 months for tremelimumab versus 10.71 months for DTIC/temozolomide (HR = 1.1416, p = 0.1272; Ribas et al, 2013).

1.1.1 Immunotherapies

It is increasingly understood that cancers are recognized by the immune system, and, under some circumstances, the immune system may control or even eliminate tumors (Dunn et al, 2004). Studies in mouse models of transplantable tumors have demonstrated that manipulation of co-stimulatory or co-inhibitory signals can amplify T-cell responses against tumors (Peggs et al 2009). This amplification may be accomplished by blocking co-inhibitory molecules, such as cytotoxic T-lymphocyte-associated antigen 4 (CTLA-4) or programmed cell death 1 (PD-1), from binding with their ligands, B7 or B7-H1 (programmed cell death ligand 1 [PD-L1]).

1.1.2 Durvalumab

The non-clinical and clinical experience is fully described in the last version of the durvalumab Investigator’s Brochure .

Durvalumab is a human monoclonal antibody (mAb) of the immunoglobulin G (IgG) 1 kappa subclass that inhibits binding of PD-L1 and is being developed by AstraZeneca/MedImmune for

use in the treatment of cancer (MedImmune is a wholly owned subsidiary of AstraZeneca; AstraZeneca/MedImmune will be referred to as AstraZeneca throughout this document). As durvalumab is an engineered mAb, it does not induce antibody-dependent cellular cytotoxicity or complement-dependent cytotoxicity. The proposed mechanism of action for durvalumab is interference of the interaction of PD-L1.

PD-L1 is expressed in a broad range of cancers with a high frequency, up to 88% in some types of cancers. In a number of these cancers, including lung, the expression of PD-L1 is associated with reduced survival and an unfavorable prognosis. In lung cancer, only 12% of patients with tumors expressing PD-L1 survived for more than 3 years, compared with 20% of patients with tumors lacking PD-L1 (Mu et al, 2011). Based on these findings, an anti-PD-L1 antibody could be used therapeutically to enhance anti-tumor immune responses in patients with cancer. Results of several non-clinical studies using mouse tumor models support this hypothesis, where antibodies directed against PD-L1 or its receptor PD-1 showed anti-tumor activity (Hirano et al 2005, Iwai et al 2002, Okudaira et al 2009, Zhang et al 2008).

Durvalumab has been given to humans as part of ongoing studies as a single drug or in combination with other drugs.

As of the data cut-off date (12 July 2017) across the entire clinical development program, an estimated 4067 patients have been exposed to 1 or more doses of durvalumab in AstraZeneca-sponsored Phase I to III studies, either as monotherapy or in combination, and 5911 patients where the treatment arm is blinded. Additionally, approximately 4000 patients have been exposed to 1 or more doses of durvalumab in ESR/IITs.
Estimates of overall cumulative patient exposure based on actual exposure data from any completed clinical trials and the enrolment/randomisation schemes for ongoing open label and blinded trials are: 3723 patients received durvalumab monotherapy, 3372 patients received durvalumab in combination with tremelimumab.Details on the safety profile of durvalumab monotherapy, tremelimumab monotherapy and durvalumab plus tremelimumab are summarized in Section 1.4.2. Refer to the current durvalumab Investigator’s Brochure for a complete summary of non-clinical and clinical information including safety, efficacy and pharmacokinetics.

1.1.3 Tremelimumab

The non-clinical and clinical experience is fully described in the last version of the tremelimumab Investigator’s Brochure.

Tremelimumab is an IgG-2 kappa isotype mAb directed against the cytotoxic T-lymphocyte-associated protein 4 (CTLA-4) also known as CD152 (cluster of differentiation 152). This is an immunomodulatory therapy (IMT) that is being developed by AstraZeneca for use in the treatment of cancer.

Binding of CTLA-4 to its target ligands (B7-1 and B7-2) provides a negative regulatory signal, which limits T-cell activation. Anti-CTLA-4 inhibitors antagonize the binding of CTLA-4 to B7 ligands and enhance human T-cell activation as demonstrated by increased cytokine (interleukin [IL]-2 and interferon [IFN] gamma) production in vitro in whole blood or peripheral blood mononuclear cell (PBMC) cultures (Tarhini and Kikwood 2008). In addition, blockade of CTLA-4 binding to B7 by anti-CTLA-4 antibodies results in markedly enhanced T-cell activation and anti-tumor activity in animal models, including killing of established murine solid tumors and induction of protective anti-tumor immunity. See the last version of tremelimumab IB, , for more

information. Therefore, it is expected that treatment with an anti-CTLA-4 antibody, such as tremelimumab, will lead to increased activation of the human immune system, increasing anti-tumor activity in patients with solid tumors.

An extensive program of non-clinical and clinical studies has been conducted for tremelimumab both as monotherapy and combination therapy with conventional anticancer agents to support various cancer indications using different dose schedules.

To date Nov/2017 tremelimumab has been given to more than 1000 patients as part of ongoing studies either as monotherapy or in combination with other anticancer agents. Refer to the current tremelimumab Investigator's Brochure for a complete summary of non-clinical and clinical information including safety, efficacy and pharmacokinetics.

1.1.4 Durvalumab in combination with tremelimumab

Targeting both PD-1 and CTLA-4 pathways may have additive or synergistic activity (Pardoll 2012) because the mechanisms of action of CTLA-4 and PD-1 are non-redundant; therefore, AstraZeneca is also investigating the use of durvalumab + tremelimumab combination therapy for the treatment of cancer.

Durvalumab + tremelimumab: Study D4190C00006: As of 28 February 2017, durvalumab PK (n=347) and tremelimumab PK (n=353) data were available from the dose-escalation and dose-expansion phases following durvalumab Q4W (3, 10, 15, or 20 mg/kg) or Q2W (10 mg/kg) in combination with tremelimumab Q4W (1, 3, or 10 mg/kg). An approximately dose-proportional increase in PK exposure (C max and AUC 0 to 28 days [AUC 0-28]) of both durvalumab and tremelimumab was observed over the dose range of 3 to 20 mg/kg durvalumab Q4W and 1 to 10 mg/kg tremelimumab Q4W. Exposures following multiple doses demonstrated accumulation consistent with PK parameters estimated from the first dose. The observed PK exposures of durvalumab and tremelimumab following combination were consistent with respective monotherapy data, indicating no PK interaction between these 2 agents.

As of DCO of28 February 2017, ADA data were available from 99 patients for durvalumab and 95 patients for tremelimumab in Study D4190C00006. The ADA incidence was 2.0% (2/99 patients) for durvalumab and 5.3% (5/95 patients) for tremelimumab. There was no clear relationship between ADA and the dose of either durvalumab or tremelimumab, and no apparent association between ADA and safety or efficacy.

Durvalumab has also been combined with other anticancer agents, including gefitinib, dabrafenib, and trametinib. To date, no PK interaction has been observed between durvalumab and these agents.

1.2 Research hypothesis

The most used therapy for neuroendocrine tumors are somatostatin analogs, with clear evidence of antitumor activity demonstrated by two phase III clinical trials in gastroenteropancreatic neuroendocrine tumors. However, even the use in lung carcinoids is quite usual, no antitumoral activity has been demonstrated in prospective clinical trials. In recent years, target therapy as sunitinib and everolimus have been approved for pancreatic neuroendocrine tumors and recently everolimus have showed a significant reduction in the risk of progression or death in patients with nonfunctional neuroendocrine tumors of lung or gastrointestinal origin. After the failure of these

therapies, no other drugs have demonstrated efficacy. Only Interferon alpha-2b is an option for these patients who have worsening symptoms of carcinoid syndrome while on treatment with somatostatin analogs and have showed some evidence of antitumoral activity in neuroendocrine tumors. The efficacy of Interferon alpha 2-b is not fully understood, but one of its antitumoral mechanisms of action could be related via stimulation of T cells. Tremelimumab and Durvalumab are much more efficient drugs to improve immune system activation and could obtain a significantly higher clinical benefit in these patients. The presence of tumor-infiltrating lymphocytes (TILS) in these type of tumors have been described enhancing the hypothesis of immunotherapy in this setting.

Following the knowledge of increased tumor-specific antigens with the presence of more mutational load, dedifferentiation process and treatments previously received we hypothesize that immunotherapy could have a higher effect in the setting of refractory neuroendocrine neoplasms, when tumor behavior is more aggressive and the probability of immune escape process is higher.

1.3 Rationale for conducting this study

Well and moderately differentiated neuroendocrine tumors of the gastroenteropancreatic tract and lung are orphan tumors after progression a targeted therapy. For gastrointestinal origin tumors, somatostatin analogues have been the cornerstone of antihormonal therapy during decades. During the last 5 years, antiproliferative effects have also been demonstrated in this setting. Recently, for neuroendocrine tumors of the lung (typical and atypical carcinoids), everolimus has demonstrated impact in tumor growth control and improvement in progression-free survival. However, after progression to a targeted therapy, no other drugs are approved.

Within the lack of treatment options, combinations of somatostatin analogues and interferon alpha have widely been used, mainly for their effect in tumor hormone release and for the evidence of tumor growth control in many retrospective analyses.

The hypothesis of efficacy of interferon alpha in these settings could be via stimulation of T cells. Recently, PD-L1 expression has been observed in neuroendocrine tumors such as small cell neuroendocrine carcinomas of the lung and Merkel cell carcinoma. These facts could provide a timely opportunity to develop specific studies with targeted therapies that stimulate the immune system against the tumor. Currently, several clinical trials are ongoing in these G3 high grade neuroendocrine tumors of the lung (small cell lung cancer) and skin (Merkel Cell carcinomas). However, there are no clinical trials ongoing in G3 gastroenteropancreatic neuroendocrine neoplasms (which is the most frequent site of G3 neuroendocrine carcinomas after the lung origin) and well-moderately differentiated (G1/G2) neuroendocrine tumors, which are the ones where immunotherapy with interferon alpha has demonstrated some activity.

Tremelimumab and Durvalumab would be the first immune combination agents showing efficacy in G1/G2/G3 advanced neuroendocrine neoplasms of gastroenteropancreatic or lung origin.

As an antibody that blocks the interaction between PD-L1 and its receptors, durvalumab may relieve PD-L1-dependent immunosuppressive effects and, therefore, enhance the cytotoxic activity of anti-tumor T-cells. This hypothesis is supported by emerging clinical data from other mAbs targeting the PD-L1/PD-1 pathway, which provide early evidence of clinical activity and a manageable safety profile (Brahmer et al 2012, Topalian et al 2012). Responses have been observed in patients with PD-L1-positive tumors and patients with PD-L1-negative tumors. In

addition, durvalumab monotherapy has shown durable responses in NSCLC in Study 1108 (see Section 1.4.1.1).

The rationale for combining durvalumab and tremelimumab is that the mechanisms of CTLA-4 and PD-1 are non-redundant, suggesting that targeting both pathways may have additive or synergistic activity (Pardoll 2012). In fact, combining immunotherapy agents has been shown to result in improved response rates (RRs) relative to monotherapy. For example, the concurrent administration of nivolumab and ipilimumab to patients with advanced melanoma induced higher objective response rates (ORRs) than those obtained with single-agent therapy. Importantly, responses appeared to be deep and durable (Wolchok et al 2013). Similar results have been observed in an ongoing study of durvalumab + tremelimumab in NSCLC (Antonia et al 2014), with further updated details presented in this clinical study protocol.

1.3.1 Durvalumab + tremelimumab combination therapy dose rationale

The durvalumab + tremelimumab doses and regimen selected for this study are based on the goal of selecting an optimal combination dose of durvalumab and tremelimumab that would yield sustained target suppression (sPD-L1), demonstrate promising efficacy, and have an acceptable safety profile.

In order to reduce the dosing frequency of durvalumab to align with the q4w dosing of tremelimumab, while ensuring an acceptable PK/PDx, safety, and efficacy profile, cohorts were narrowed to 15 and 20 mg/kg durvalumab q4w. PK simulations from the durvalumab monotherapy data indicated that a similar area under the plasma drug concentration-time curve at steady state (AUC_{ss}; 4 weeks) was expected following both 10 mg/kg q2w and 20 mg/kg q4w durvalumab. The observed durvalumab PK data from the D4190C00006 study were well in line with the predicted monotherapy PK data developed preclinically. This demonstrates similar exposure of durvalumab 20 mg/kg q4w and 10 mg/kg q2w, with no alterations in PK when durvalumab and tremelimumab (doses ranging from 1 to 3 mg/kg) are dosed together. While the median Cmax at steady state ($C_{max,ss}$) is expected to be higher with 20 mg/kg q4w (approximately 1.5 fold) and median trough concentration at steady state ($C_{trough,ss}$) is expected to be higher with 10 mg/kg q2w (approximately 1.25 fold), this is not expected to impact the overall safety and efficacy profile, based on existing preclinical and clinical data.

Monotonic increases in PDx activity were observed with increasing doses of tremelimumab relative to the activity observed in patients treated with durvalumab monotherapy. There was evidence of augmented PDx activity relative to durvalumab monotherapy with combination doses containing 1 mg/kg tremelimumab, inclusive of both the 15 and 20 mg/kg durvalumab plus 1 mg/kg tremelimumab combinations.

Patients treated with doses of tremelimumab above 1 mg/kg had a higher rate of adverse events (AEs), including discontinuations due to AEs, serious AEs (SAEs), and severe AEs. Between the 10 mg/kg durvalumab + 1 mg/kg tremelimumab and 10 mg/kg durvalumab + 3 mg/kg tremelimumab cohorts treated at the q2w schedule, the number of patients reporting any AE, Grade 3 AEs, SAEs, and treatment-related AEs was higher in the 10 mg/kg durvalumab + 3 mg/kg tremelimumab cohort than the 10 mg/kg durvalumab + 1 mg/kg tremelimumab cohort. A similar pattern was noted in the q4w regimens, suggesting that, as the dose of tremelimumab increased above 1 mg/kg, a higher rate of treatment-related events may be anticipated. Further, the SAEs frequently attributed to immunotherapy, pneumonitis and colitis, were more commonly seen in

cohorts using either 3 or 10 mg/kg of tremelimumab compared to the 1-mg/kg dose cohorts. Together, these data suggest that a combination using a tremelimumab dose of 1 mg/kg appeared to minimize the rate of toxicity when combined with durvalumab. As a result, all combination doses utilizing either the 3 or 10 mg/kg doses of tremelimumab were eliminated in the final dose selection.

In contrast, cohorts assessing higher doses of durvalumab with a constant dose of tremelimumab did not show an increase in the rate of AEs. The data suggested that increasing doses of durvalumab may not impact the safety of the combination as much as the tremelimumab dose. Further, safety data between the 10-mg/kg and 20-mg/kg cohorts were similar, with no change in safety events with increasing dose of durvalumab.

In Study D4190C00006, of all treatment cohorts, the cohort of 11 patients treated in the 20 mg/kg durvalumab + 1 mg/kg tremelimumab group had the fewest AEs, Grade ≥3 AEs, SAEs, and treatment discontinuations due to AEs, but still showed strong evidence of clinical activity. This cohort had a lower number of treatment-related Grade ≥3 AEs or treatment-related SAEs. No dose-limiting toxicities were reported.

Preliminary clinical activity of the durvalumab and tremelimumab combination did not appear to change with increasing doses of tremelimumab. The 15- and 20-mg/kg durvalumab q4w cohorts demonstrated objective responses at all doses of tremelimumab, and increasing doses of tremelimumab did not provide deeper or more rapid responses.

Efficacy data suggested that the 20 mg/kg durvalumab + 1 mg/kg tremelimumab dose cohort may demonstrate equivalent clinical activity to another dose combinations. Of the 45 patients, full analysis set,, there were 7 patients (15,6%) with PR, 19 patients (42,2%) with SD, and 15 patients (33,3%) with PD. Four patients were not evaluable for response .

Additionally, of all cohorts, the 20 mg/kg durvalumab + 1 mg/kg tremelimumab dose cohort had the fewest AEs, Grade ≥3 AEs, SAEs, and treatment discontinuations due to AEs, but still showed some evidence of clinical activity. All together, the data suggested that a 20 mg/kg durvalumab + 1 mg/kg tremelimumab dose combination should be selected for further development.

Refer to the current durvalumab Investigator's Brochure for a complete summary of non-clinical and clinical information on the durvalumab + tremelimumab combination, including safety, efficacy and pharmacokinetics.

1.3.2 Rationale for 4 cycles of combination therapy followed by durvalumab monotherapy

Long-term follow up on melanoma patients treated with ipilimumab, an anti-CTLA-4 targeting antibody (dosed every 3 weeks [q3w] for 4 doses and then discontinued), shows that patients responding to ipilimumab derive long-term benefit, with a 3-year OS rate of approximately 22%. Furthermore, the survival curve in this population reached a plateau at 3 years and was maintained through 10 years of follow up (Schadendorf et al, 2013).

Similar data have been presented for other anti-PD-1/PD-L1 targeting antibodies:

Nivolumab (anti-PD-1) was dosed q2w for up to 96 weeks in a large Phase I dose-escalation and expansion study, and showed responses were maintained for a median of 22.94 months for melanoma (doses 0.1 mg/kg to 10 mg/kg), 17 months for NSCLC (doses 1, 3, and 10 mg/kg), and

12.9 months for renal cell carcinoma patients (doses 1 and 10 mg/kg) at the time of data analysis (Hodi et al 2014, Bramer et al 2014, Drake et al 2013). Furthermore, responses were maintained beyond treatment discontinuation in the majority of patients who stopped nivolumab treatment (either due to protocol specified end of treatment, complete response [CR], or toxicity) for up to 56 weeks at the time of data analysis (Topalian et al 2014).

MPDL3280a (anti-PD-L1) and the combination of nivolumab with ipilimumab, in which patients were dosed for a finite time period and responses maintained beyond treatment discontinuation have been reported (Herbest et al 2013, Wolchok et al 2013).

Similar long term results may be expected with use of other immune-mediated cancer therapeutics including anti-CTLA-4 antibodies such as tremelimumab, anti PD-L1 antibodies such as durvalumab, or the combination of the two.

1.4 Benefit/risk and ethical assessment

Natural history of neuroendocrine tumors is usually prolonged. G1/G2 neoplasms are slowing growing tumors that require continued therapy to control tumor growth, increasing the risk of long-term side effects. The prolonged effect of immunotherapy could be of especial interest in this setting, reducing the risk of chronic toxicity and maintaining the long-term benefit.

1.4.1 Potential benefits

1.4.1.1 Durvalumab

The majority of the safety and efficacy data currently available for durvalumab are based on the first time in-human, single-agent study (Study 1108) in patients with advanced solid tumors. Data from Study 1108 were presented at the European Society for Medical Oncology 2014 Congress. Overall, 456 of 694 subjects treated with durvalumab 10 mg/kg Q2W were evaluable for response (defined as having ≥ 24 weeks follow-up, measurable disease at baseline, and ≥ 1 follow-up scan, or discontinued due to disease progression or death without any follow-up scan). In PD-L1 unselected patients, the objective response rate (ORR), based on investigator assessment per Response Evaluation Criteria in Solid Tumors (RECIST)v1.1, ranged from 0% in uveal melanoma (n = 23) to 20.0% in bladder cancer (n = 15), and disease control rate at 24 weeks (DCR-24w) ranged from 4.2% in triple-negative breast cancer (TNBC; n = 24) to 39.1% in advanced cutaneous melanoma (n = 23). PD-L1 status was known for 383 of the 456 response evaluable subjects. Across the PD-L1-positive tumors, ORR was highest for bladder cancer, advanced cutaneous melanoma, hepatocellular carcinoma (HCC; n = 3 each, 33.3% each), NSCLC (n = 86, 26.7%), and squamous cell carcinoma of the head and neck (SCCHN; n = 22, 18.2%). In the PD-L1-positive subset, DCR-24w was highest in advanced cutaneous melanoma (n = 3, 66.7%), NSCLC (n = 86, 36.0%), HCC and bladder cancer (n = 3 each, 33.3% each), and SCCHN (n = 22, 18.2%)(Antonia et al 2014b).

1.4.1.2 Tremelimumab

In a single-arm, Phase II study (Study A3671008) of tremelimumab administered at 15 mg/kg every 90 days to patients with refractory melanoma, an RR of 7% and a median OS of 10 months in the second-line setting (as compared to approximately 6 months with best supportive care reported from a retrospective analysis; Korn et al 2008) were observed (Kirkwood et al 2010). In a randomized, open-label, first-line Phase III study of tremelimumab (administered at 15 mg/kg every

90 days) versus chemotherapy (dacarbazine or temozolomide) in advanced melanoma (Study A3671009), results of the final analysis showed an RR of 11% and a median OS of 12.58 months in this first-line setting as compared to 10.71 months with standard chemotherapy; however, these results were not statistically significant (Ribas et al 2013). Additionally, a Phase II maintenance study (Study A3671015) in patients with Stage IIIB or IV NSCLC who had responded or remained stable failed to achieve statistical significance. The primary endpoint of PFS at 3 months was 22.7% in the tremelimumab arm (15 mg/kg) compared with 11.9% in the best supportive care arm (Study A3671015).

1.4.1.3 Durvalumab + tremelimumab

The preclinical and clinical justification for this combination as noted in Section 1.1.4 also supports the synergy of this combination. Available data, such as those presented by Wolchok et al, suggest that the combination of agents targeting PD-1/PD-L1 and CTLA-4 may have profound and durable benefits in patients with melanoma (Wolchok et al 2013). Of the 102 subjects with advanced NSCLC treated with durvalumab in combination with tremelimumab in Study D4190C00006, 63 subjects with at least 16 weeks of follow-up were evaluable for response (defined as measurable disease at baseline and at least 1 follow-up scan; this included discontinuations due to disease progression or death without follow-up scan). Of the 63 evaluable subjects, 17 (27%) had a best overall response of PR,14 (22%) had SD,22 (35%) had PD, and 10 (16%) were not evaluable. The ORR(confirmed and unconfirmed CR or PR) was 27% and the DCR (CR, PR, or SD) was 49% as assessed by RECIST v1.1.

Current experience with single-agent IMT studies suggests that clinical responses may be restricted to a subset of any given patient population and that it might be beneficial to enrich the patient population by selecting patients likely to respond to therapy. To date, no assay has been established or validated, and no single approach has proven accurate, for patient enrichment for IMTs. However, independent data from multiple sources using different assays and scoring methods suggests that PD-L1 expression on tumor cells and/or tumor infiltrating cells may be associated with greater clinical benefit.

Data from ongoing studies with durvalumab and other agents targeting the PD-1/PD-L1 pathway suggest, as shown in a number of tumor types (eg, NSCLC, renal cell carcinoma, and melanoma), that monotherapy may be more efficacious (in terms of ORR) in patients who are PD-L1-positive.

Given these findings, a number of ongoing studies are assessing the activity of agents in patients with PD-L1–positive tumors. There is also an unmet medical need in patients with PD-L1–negative tumors that needs to be addressed. Data, as of 27 January 2015 from Study 006 show that with the addition of tremelimumab to durvalumab, the ORR can be increased to 25% in patients with PD-L1 negative NSCLC.

1.4.2 Potential risks

1.4.2.1 Durvalumab

Risks with durvalumab include cough/productive cough, pneumonitis, ILD, dysphonia, ALT/AST increased, hepatitis, diarrhoea, abdominal pain, colitis, hypothyroidism, hyperthyroidism, blood TSH increased, blood TSH decreased, adrenal insufficiency, Type 1 diabetes mellitus, hypophysitis/hypopituitarism, diabetes insipidus, blood creatinine increased, dysuria, nephritis,

rash, pruritus, night sweats, dermatitis, myocarditis, pyrexia, oedema peripheral, upper respiratory tract infections, pneumonia, oral candidiasis, dental and oral soft tissue infections, influenza, myalgia, myositis, polymyositis and infusion related reaction.

ImAEs: immune-mediated pneumonitis, hepatitis, colitis, hypothyroidism, hyperthyroidism, adrenal insufficiency, Type 1 diabetes mellitus, hypophysitis/hypopituitarism, nephritis and rash. Other immune-mediated adverse reactions including myocarditis and myositis/polymyositis. These imAEs should be managed based on dosing and Toxicity Management Guidelines described in appendix 1 of this protocol.

Infusion-related reactions: as with the administration of any Ig, infusion-related reactions may occur and may be severe and should be managed according to Toxicity Management Guidelines and study protocol.

As patients with previous history of bleeding and/or who are taking anticoagulant medications may have a higher risk of subsequent bleeding, the investigator should devote special attention to monitoring of these patients in the context of possible bleeding.

Further information on these risks can be found in the current version of the durvalumab IB.

In monotherapy clinical studies AEs (all grades) reported very commonly (≥10% of patients) are fatigue, cough , decreased appetite , dyspnoea , nausea , constipation , diarrhoea , pyrexia , back pain, anaemia, vomiting, pruritus, arthralgia, headache, asthenia, oedema peripheral and rash.

A total of 178 patients (9.4%) discontinued from study treatment due to an AE. A total of 108 patients (5.7%) had serious AEs (SAEs) that were considered by the investigator as related to durvalumab.

Most treatment-related AEs were manageable by delaying or interrupting the durvalumab dose, symptomatic treatment, and in the case of events suspected to have an immune basis, the use of established treatment guidelines for immune-mediated toxicity (see the Dosing Modification and Toxicity Management Guidelines).

A detailed summary of durvalumab monotherapy AE data can be found in the current version of the durvalumab IB.

1.4.2.2 Tremelimumab

Potential risks, based on the mechanism of action of tremelimumab and related molecules (ipilimumab) include potentially immune-mediated gastrointestinal (GI) events including enterocolitis, intestinal perforation, abdominal pain, dehydration, nausea and vomiting, and decreased appetite (anorexia); dermatitis including urticaria, skin exfoliation, and dry skin; endocrinopathies including hypophysitis, adrenal insufficiency, and hyperthyroidism and hypothyroidism; hepatitis including autoimmune hepatitis and increased serum ALT and AST; pancreatitis including autoimmune pancreatitis and lipase and amylase elevation; respiratory tract events including pneumonitis and interstitial lung disease (ILD); nervous system events including encephalitis, peripheral motor and sensory neuropathies, and Guillain-Barré syndrome; cytopenias including thrombocytopenia, anemia, and neutropenia; infusion-related reactions; anaphylaxis; and serious allergic reactions. The profile of AEs and the spectrum of event severity have remained stable across the tremelimumab clinical program and are consistent with the pharmacology of the

target. To date, no tumor type or stage appears to be associated with unique AEs (except for vitiligo that appears to be confined to patients with melanoma). Overall, 944 of the 973 patients (97.0%) treated with tremelimumab monotherapy as of the data cutoff date of 1 November 2015 (not including 569 patients who have been treated in the ongoing blinded Phase IIb Study D4880C00003) experienced at least 1 AE. The events resulted in discontinuation of tremelimumab in 10.0% of patients, were serious in 36.5%, were Grade ≥3 in severity in 49.8%, were fatal in 67.7%, and were considered to be treatment related in 79.1% of patients. The frequency of any AEs and Grade ≥3 AEs was generally similar across the tremelimumab dose groups. However, a higher percentage of patients in the 10 mg/kg every 28 days and 15 mg/kg every 90 days groups compared with the All Doses <10 mg/kg group experienced treatment-related AEs, SAEs, AEs resulting in discontinuation of investigational product (IP), and deaths.

A detailed summary of tremelimumab monotherapy AE data can be found in the current version of the tremelimumab IB.

1.4.2.3 Durvalumab + tremelimumab

Safety data have been pooled for 5 durvalumab and tremelimumab combination studies (D4190C00002, D4190C00006, D4190C00010, D4190C00011 and D4193C00003 [CONDOR]) for patients who received a dose of 20 mg/kg Q4W durvalumab plus 1 mg/kg tremelimumab Q4W, or equivalent durvalumab 10 mg/kg Q2W + tremelimumab 1 mg/kg Q4W; a total of 1088 patients are included in the pooled data set with a DCO of the 12 July 2017.
Overall, AEs reported in ≥10% of patients were fatigue, diarrhoea, nausea, decreased appetite, dyspnoea, pruritus, anaemia, constipation, pyrexia, vomiting, cough, back pain, abdominal pain, rash, arthralgia, hypothyroidism, asthenia, hyponatraemia, weight decreased, and oedema peripheral.

A total of 166 patients (15.3%) had SAEs that were considered treatment-related by the investigators. A total of 165 patients (15.2%) discontinued from study treatment due to an AE.

- Potential imAEs for durvalumab and durvalumab in combination with tremelimumab include:
  - Pancreatitis
  - Other rare or less frequent events with a potential immune-mediated aetiology, eg, pericarditis, sarcoidosis, uveitis, and other events involving the eye (eg, keratitis and optic neuritis), skin (eg, scleroderma and vitiligo), and haematological (eg, haemolytic anaemia and immune thrombocytopenic purpura), rheumatological events (polymyalgia rheumatic and autoimmune arthritis) and neuropathy/neuromuscular toxicities (eg, myasthenia gravis, Guillain Barre syndrome).
- Hypersensitivity reactions including
  - Anaphylaxis and allergic reaction
  - Cytokine release syndrome
  - Immune complex disease
- Other infections

Infusion-related reactions: as with the administration of any Ig, infusion-related reactions may occur and may be severe and should be managed according to Toxicity Management Guidelines and

study protocol.

A detailed summary of durvalumab + tremelimumab combination AE data can be found in the current version of the durvalumab IB.

1.4.2.4 Fixed Dosing for durvalumab and tremelimumab

A population PK model was developed for durvalumab using monotherapy data from a Phase 1 study (*study 1108; N=292; doses= 0.1 to 10 mg/kg Q2W or 15 mg/kg Q3W; solid tumors*). Population PK analysis indicated only minor impact of body weight (WT) on PK of durvalumab (coefficient of ≤ 0.5). The impact of body WT-based (10 mg/kg Q2W) and fixed dosing (750 mg Q2W) of durvalumab was evaluated by comparing predicted steady state PK concentrations (5th, median and 95th percentiles) using the population PK model. A fixed dose of 750 mg was selected to approximate 10 mg/kg (based on median body WT of ~75 kg). A total of 1000 patients were simulated using body WT distribution of 40–120 kg. Simulation results demonstrate that body WT-based and fixed dosing regimens yield similar median steady state PK concentrations with slightly less overall between-subject variability with fixed dosing regimen.

Similarly, a population PK model was developed for tremelimumab using data from Phase 1 through Phase 3 (*N=654; doses= 0.01 to 15 mg/kg Q4W or Q90D; metastatic melanoma*) [Wang et al. 2014]. Population PK model indicated minor impact of body WT on PK of tremelimumab (coefficient of ≤ 0.5). The WT-based (1 mg/kg Q4W) and fixed dosing (75 mg/kg Q4W; based on median body WT of ~75 kg) regimens were compared using predicted PK concentrations (5th, median and 95th percentiles) using population PK model in a simulated population of 1000 patients with body weight distribution of 40 to 120 kg. Similar to durvalumab, simulations indicated that both body WT-based and fixed dosing regimens of tremelimumab yield similar median steady state PK concentrations with slightly less between-subject variability with fixed dosing regimen.

Similar findings have been reported by others [Ng et al 2006, Wang et al 2009, Zhang et al 2012, Narwal et al 2013]. Wang and colleagues investigated 12 monoclonal antibodies and found that fixed and body size-based dosing perform similarly, with fixed dosing being better for 7 of 12 antibodies. In addition, they investigated 18 therapeutic proteins and peptides and showed that fixed dosing performed better for 12 of 18 in terms of reducing the between-subject variability in pharmacokinetic/pharmacodynamics parameters [Zhang et al 2012].

A fixed dosing approach is preferred by the prescribing community due to ease of use and reduced dosing errors. Given expectation of similar pharmacokinetic exposure and variability, we considered it feasible to switch to fixed dosing regimens. Based on average body WT of 75 kg, a fixed dose of 750 mg Q2W MEDI4736 (equivalent to 10 mg/kg Q2W), 1500 mg Q4W durvalumab (equivalent to 20 mg/kg Q4W) and 75 mg Q4W tremelimumab (equivalent to 1 mg/kg Q4W) is included in the current study.

Fixed dosing of durvalumab and tremelimumab is recommended only for subjects with more or equal than 30kg body weight due to endotoxin exposure. Patients with a body weight less than 30 kg should be dosed using a weight-based dosing schedule.

2. STUDY OBJECTIVES

2.1 Primary objective(s)

The study will include patients with advanced neuroendocrine neoplasms in four different cohorts with the following primary endpoints:

Primary endpoint for cohorts 1, 2 and 3:

Nine-months clinical benefit rate (CBR) by Response Evaluation Criteria In Solid Tumors (RECIST 1.1), which is defined as the percentage of patients achieving complete response (CR), partial response (PR), or stable disease (SD) at month 9 after durvalumab plus tremelimumab was started.

Primary endpoint for cohort 4:

Nine-months overall survival rate, which is defined as the percentage of patients alive at month 9 after durvalumab plus tremelimumab was started.

2.2 Secondary objective(s)

- Overall response rate (ORR) by irRECIST.
- To assess the duration of response according to irRECIST.
- To assess the median progression-free survival time (PFS) according to irRECIST.
- To assess the safety profile of Durvalumab and Tremelimumab in subjects with advanced neuroendocrine neoplasms.
- To assess the median overall survival (OS) time.
- To assess response status according to irRECIST at 6 and 12 months after start of study treatment.

2.3 Exploratory objective(s)

- To evaluate biochemical response (changes in CgA and NSE levels) and its association with response rate and progression-free survival.
- To assess whether baseline tumor and blood biomarkers may be predictive of response to durvalumab and tremelimumab therapy.
- To explore additional hypotheses related to biomarkers and relationship to durvalumab and tremelimumab efficacy and/or toxicity and neuroendocrine tumors evolution that may arise from internal or external research activities.

3. STUDY DESIGN

3.1 Overview of study design

This is a prospective, multi-center, open label, stratified, exploratory phase II study evaluating the efficacy and safety of durvalumab plus tremelimumab in different cohorts of patients with neuroendocrine neoplasms.

The study will include patients in four different cohorts:

- **Cohort 1**: Well-moderately differentiated lung neuroendocrine tumors (classically known as typical and atypical carcinoids) after progression to somatostatin analogs and one prior targeted therapy or chemotherapy.
- **Cohort 2**: G1/G2 (WHO grade 1 and 2) gastrointestinal neuroendocrine tumors after progression to somatostatin analogs and one prior targeted therapy.
- **Cohort 3**: G1/G2 (WHO grade 1 and 2) pancreatic neuroendocrine tumors after progression to standard therapies (chemotherapy, somatostatin analogs and target therapy), who have received between two and four prior lines of treatment.
- **Cohort 4**: Neuroendocrine neoplasms (WHO grade 3) of gastroenteropancreatic origin or unknown primary site (excluding lung primary tumors), patients will be treated in second line only, after progression to first-line chemotherapy with a platinum based regimen.

The patient recruitment period of the study will last approximately 24 months. The maximum treatment period for each subject on study is anticipated to be approximately 12 months (13 administrations of durvalumab). However, subjects will continue on active follow-up to determine secondary endpoints of duration of response, median progression-free survival, median overall survival and safety profile.

This study will be conducted in 3 phases: a screening phase, a treatment phase stratified by tumor origin (four different cohort) and a follow up phase.

3.2 Study schema

Screening phase

Screening will occur between Day -28 and Day -1. The purpose of the screening period is to establish protocol eligibility. Informed consent will be obtained up to 4 weeks prior to Cycle 1 Day 1 and after the study has been fully explained to each subject and prior to the conduct of any screening procedures or assessments.

The purpose of the baseline visit is to establish disease characteristics prior to allocation and treatment and to confirm protocol eligibility as specified in the inclusion/exclusion criteria. Results of baseline assessments must be obtained prior to the first dose of study drug (Cycle 1/Day 1). Baseline assessments may be performed on Day -1 or on Cycle 1/Day 1 prior to dosing. Clinical laboratory tests including pregnancy test (where applicable) can be performed within 72 hours of the first dose of study drug. Subjects who complete the baseline visit and continue to meet the criteria for inclusion/exclusion will begin the treatment phase of this study.

Determinations performed by routine clinical practice before signing the informed consent will be

valid, provided they are carried out following this protocol requirements and date of baseline TC is performed within the previous 4 weeks from treatment initiation. It should be clearly stated that patient consented her/his participation to ESR 15-11561-61–DUNE trial in patient record.

Treatment phase

The treatment phase will begin at the time of allocation of the first patient and will consist of the study treatment cycles. The treatment phase will end when the last patient discontinue the study drug.

The treatment phase will include two stages for each cohort. The first stage will dose the first patient in each cohort in a safety run period for one cycle (4 weeks). If no unexpected side effects are observed in the first patient treated in each cohort during the safety run period, recruitment will continue up to 31 patients for cohorts 1 to 3, and 33 for cohort 4.

Subjects will be allocated in each primary tumor cohort to receive durvalumab 1500 mg Q4W for 12 months plus tremelimumab 75 mg Q4W for up to 4 doses during the first 4 cycles of combined therapy. Cycles are defined by 4 weeks or 28 days. Subjects will undergo safety and efficacy assessment as defined per protocol.

If a toxicities pause occurs, cycles not administered for that toxicity can be delayed for a maximum of 4 weeks, after that time that cycle will be considered lost and the next cycle will be administered. Remember no restarts are allowed after a delay of more than 12 weeks due to toxicity or more than 4 weeks of delay if **the reason is different from toxicity** without consulting the Coordinating Investigator.

Follow-up phase

Patients will finish the treatment phase after the administration of 12 months (13 cycles) of durvalumab or after disease progression during the treatment phase. For patients that complete all scheduled treatment, the follow-up phase will begin to determine survival endpoints. Tumor assessments will continue every 12 weeks and clinical appointments will start monthly during the first 3 months of follow-up, every 2 months until first year, and continue every 6 months until disease progression.

3.3 Study Oversight for Safety Evaluation

A subject may elect to discontinue study drug at any time for safety, medical, or personal reasons. Patients who choose to discontinue study drug prior to disease progression will be followed in the post study treatment follow up period and continue to undergo regularly scheduled disease assessment until documentation of disease progression or start of an alternative anticancer treatment. All subjects who discontinue study drug will be followed for overall survival and all post progression cancer treatments administered will be recorded. Subjects may at any time withdraw consent for further study participation. No further data will be collected on subjects once consent has been withdrawn. The investigator will promptly explain to the subject involved that the study drug will be discontinued for that subject and provide appropriate medical treatment and other necessary measures for the subject. A subject who has ceased to return for visits will be followed up by mail, phone, or other means as much as possible to gather information such as the reason for failure to return and the status of treatment compliance, presence or absence of adverse

events, and clinical courses of signs and symptoms, and the information will be recorded in the CRF.

Subjects who discontinue early from the study or treatment will be discontinued for 1 of these primary reasons: adverse event(s), lost to follow-up, subject choice, progressive disease, or administrative/other. In addition to the primary reason, the subject may have indicated 1 or more of these reasons as secondary reasons for discontinuation. Study disposition information will be collected on the appropriate CRF. A subject removed from the study for any reason may not be replaced.

Safety will be assessed by monitoring and recording all AEs including all CTCAE grades (for both increasing and decreasing severity) and serious adverse events (SAEs); regular monitoring of hematology, clinical chemistry, and urine values; physical examinations; and regular measurement of vital signs, and electrocardiograms (ECGs) as detailed in the schedule of visits and procedures.

4. PATIENT SELECTION, ENROLLMENT, ALLOCATION, RESTRICTIONS, DISCONTINUATION AND WITHDRAWAL

Approximately 126 patients will be enrolled and allocated in a balanced manner into either the four cohorts of patients at approximately 20 sites in Spain. Each patient must meet all of the inclusion criteria (Section 4.1) and none of the exclusion criteria (Section 4.2) for this study. Under no circumstances, will there be exceptions to this rule.

4.1 Inclusion criteria

For inclusion in the study, patients should fulfill the following criteria:

1. Written informed consent obtained from the subject prior to performing any protocol-related procedures.

2. Age > 18 years at time of study entry.

3. Subjects must have histologically confirmed diagnosis of one of the following advanced/metastatic neuroendocrine tumor types:

   a) **Cohort 1:** Well-moderately differentiated neuroendocrine tumors of the lung (mitotic count ≤10 mitoses x 10 HPF), also known as typical and atypical lung carcinoids, that have progressed to prior somatostatin analog therapy and/or one prior targeted therapy or chemotherapy (only one prior systemic therapy, with the exception of patients that have been treated with somatostatin analogues and other systemic treatment, when two prior treatments are allowed).

   b) **Cohort 2:** Well-moderately differentiated neuroendocrine tumors G1/G2 (WHO grade 1 and 2) gastrointestinal neuroendocrine tumors after progression to somatostatin analogs and one targeted therapy (prior targeted therapy could be everolimus or a multikinase inhibitor). Prior therapies with interferon alpha-2b or radionucleotide therapy are allowed.

   c) **Cohort 3:** Well-moderately differentiated neuroendocrine tumors G1/G2 (WHO grade 1 and 2) from pancreatic origin after progression to standard therapies (chemotherapy,

somatostatin analogs and target therapy); patients must be treated with at least two prior systemic treatment lines and a maximum of four previous treatment lines.

d) **Cohort 4**: Neuroendocrine neoplasms (WHO grade 3) of gastroenteropancreatic origin or unknown primary site (excluding lung primary tumors), patients will be treated in second line only, after progression to first-line chemotherapy with a platinum based regimen. Patients will be treated in second line."

4. For patients included in cohorts 1, 2 and 3: WHO Classification G1/G2 (mitotic count ≤10 mitoses x 10 HPF) lung typical and atypical carcinoids for cohort 1, G1/G2 (Ki67≤20% and mitotic count ≤20 mitoses x 10 HPF) gastrointestinal for cohort 2 (including stomach, small intestine and colorectal origins), G1/G2 (Ki67≤20% and mitotic count ≤20 mitoses x 10 HPF) pancreatic for cohort 3.

5. For patients included in cohort 4: WHO classification G3 (Ki67≥20% or mitotic count >20 mitoses x 10 HPF) gastroenteropancreatic neuroendocrine carcinomas (NEC) or liver metastases of G3 NEC of unknown primary site.

6. Subjects must have evidence of measurable disease meeting the following criteria:

   a) In case of more than one target lesion, it should be identified at least 1 lesion of ≥ 1.0 cm in the longest diameter for a non lymph node, or ≥ 1.5 cm in the short-axis diameter for a lymph node, which is serially measurable according to RECIST 1.1 using computerized tomography/magnetic resonance imaging (CT/MRI). If there is only one target lesion and it is a non-lymph node, it should have a longest diameter of ≥ 1.5 cm.

   b) Lesions that have had external beam radiotherapy (EBRT) or loco-regional therapies such as radiofrequency (RF) ablation or liver embolization must show evidence of progressive disease based on RECIST 1.1 to be deemed a target lesion.

   c) Subjects must show evidence of disease progression by radiologic image techniques within 12 months (an additional month will be allowed to accommodate actual dates of performance of scans, i.e., within ≤ 13 months) prior to signing informed consent, according to RECIST 1.1.

7. Eastern Cooperative Oncology Group (ECOG) performance status of 0 or 1.

8. Life expectancy of at least 12 weeks.

9. Adequate normal organ and marrow function as defined below:

   - Haemoglobin ≥ 9.0 g/dL.

   - Absolute neutrophil count (ANC) ≥ 1.5 x 10^9/L (≥ 1500 per mm^3).

   - Platelet count ≥ 100 x 10^9/L (≥ 100,000 per mm^3).

10. Serum bilirubin ≤ 1.5 x institutional upper limit of normal (ULN). This will not apply to subjects with confirmed Gilbert's syndrome (persistent or recurrent hyperbilirubinemia that is predominantly unconjugated in the absence of hemolysis or hepatic pathology), who will be allowed only in consultation with their physician.

11. AST (SGOT)/ALT (SGPT) ≤ 2.5 x institutional upper limit of normal unless liver metastases are

present, in which case it must be ≤ 5x ULN.

12. Serum creatinine CL>40 mL/min by the Cockcroft-Gault formula (Cockcroft and Gault 1976) or by 24-hour urine collection for determination of creatinine clearance:

Males:

$$\text{Creatinine CL (mL/min)} = \frac{\text{Weight (kg)} \times (140 - \text{Age})}{72 \times \text{serum creatinine (mg/dL)}}$$

Females:

$$\text{Creatinine CL (mL/min)} = \frac{\text{Weight (kg)} \times (140 - \text{Age})}{72 \times \text{serum creatinine (mg/dL)}} \times 0.85$$

13. Female subjects must either be of non-reproductive potential (ie, post-menopausal by history: ≥60 years old and no menses for ≥1 year without an alternative medical cause; OR history of hysterectomy, OR history of bilateral tubal ligation, OR history of bilateral oophorectomy) or must have a negative serum pregnancy test upon study entry.

14. Subject is willing and able to comply with the protocol for the duration of the study including undergoing treatment and scheduled visits and examinations including follow up.

4.2 Exclusion criteria

Subjects who meet any of the following criteria will be excluded from this study:

1. Involvement in the planning and/or conduct of the study.

2. Participation in another clinical study with an investigational product during the last 4 weeks.

3. WHO Classification G3 neuroendocrine neoplasms of lung origin (oat cell/large cell lung cancer).

4. Prior treatment with anti-PDL-1/anti-PD-1 or anti-CTL4 therapy.

5. Uncontrolled intercurrent illness including, but not limited to, ongoing or active infection, symptomatic congestive heart failure, uncontrolled hypertension, unstable angina pectoris, cardiac arrhythmia, active peptic ulcer disease or gastritis, active bleeding diathesis including any subject known to have evidence of acute or chronic hepatitis B (e.g., HBsAg reactive), hepatitis C (e.g., HCV RNA [qualitative] is detected) or known history of Human Immunodeficiency Virus (HIV) (HIV 1/2 antibodies), or psychiatric illness/social situations that would limit compliance with study requirements or compromise the ability of the subject to give written informed consent.

6. Known history of previous clinical diagnosis of tuberculosis.

7. Current or prior use of immunosuppressive medication within 28 days before the first dose of durvalumab or tremelimumab, with the exceptions of intranasal and inhaled corticosteroids or systemic corticosteroids at physiological doses, which are not to exceed 10 mg/day of prednisone, or an equivalent corticosteroid.

8. Active or prior documented autoimmune disease within the past 2 years

NOTE: Subjects with vitiligo, Grave's disease, or psoriasis not requiring systemic treatment (within

the past 2 years) are not excluded.

9. Active or prior documented inflammatory bowel disease (e.g., Crohn's disease, ulcerative colitis).

10. History of allogeneic organ transplant.

11. History of hypersensitivity to durvalumab, tremelimumab or any excipient.

12. Subjects having a diagnosis of immunodeficiency or are receiving systemic steroid therapy or any other form of immunosuppressive therapy within 28 days prior to the first dose of trial treatment.

13. Knowledge of active central nervous system (CNS) metastases and/or carcinomatous meningitis. Subjects with previously treated brain metastases may participate provided they have stable brain metastases [without evidence of progression by imaging confirmed [by magnetic resonance imaging (MRI) if MRI was used at prior imaging, or confirmed by computed tomography (CT) imaging if CT used at prior imaging] for at least four weeks prior to the first dose of trial treatment; also, any neurologic symptoms must have returned to baseline], have no evidence of new or enlarging brain metastases, and have not used steroids for brain metastases for at least 7 days prior to trial treatment. This exception does not include carcinomatous meningitis, as subjects with carcinomatous meningitis are excluded regardless of clinical stability.

14. Receipt of live attenuated vaccination within 30 days prior to study entry or within 30 days of receiving durvalumab or tremelimumab. Note: The killed virus vaccines used for seasonal influenza vaccines for injection are allowed; however intranasal influenza vaccines (e.g., FluMist®) are live attenuated vaccines, and are not allowed.

15. Subjects having known history of, or any evidence of interstitial lung disease or active, noninfectious pneumonitis.

16. Any prior Grade ≥3 immune-related adverse event (irAE) while receiving any previous immunotherapy agent, or any unresolved irAE >Grade 1.

17. Subjects who have received any anti-cancer treatment within 21 days or any investigational agent within 30 days prior to the first dose of study drug and should have recovered from any toxicity related to previous anti-cancer treatment. This does not apply to the use of somatostatin analogues for symptomatic therapy.

18. Major surgery within 3 weeks prior to the first dose of study drug.

19. Subjects having > 1+ proteinuria on urine dipstick testing will undergo 24h urine collection for quantitative assessment of proteinuria. Subjects with urine protein ≥ 1 g/24h will be ineligible.

20. Significant cardiovascular impairment: history of congestive heart failure greater than New York Heart Association (NYHA) Class II, unstable angina; myocardial infarction or stroke within 6 months of the first dose of study drug, or cardiac arrhythmia requiring medical treatment.

21. Mean QT interval corrected for heart rate (QTc) ≥470 ms calculated from 3 electrocardiograms (ECGs) using Fredericia's Correction.

22. Bleeding or thrombotic disorders or use of anticoagulants, such as warfarin, or similar agents

requiring therapeutic international normalized ratio (INR) monitoring. Treatment with low molecular weight heparin (LMWH) is allowed.

23. Active hemoptysis (bright red blood of at least 0.5 teaspoon) within 3 weeks prior to the first dose of study drug.

24. Patients with tumoral disease in the head and neck region, such as paratracheal or periesophageal lymph node involvement, or with infiltration of structures in the digestive tract, or vascular pathways that represent a risk of increased bleeding.

25. Patients of cohort 1 with neuroendocrine tumors of pulmonary origin or pulmonary metastases with evidence of active bleeding.

26. Patients with evidence of digestive bleeding.

27. Active infection (any infection requiring treatment).

28. Active malignancy (except for differentiated thyroid carcinoma, or definitively treated melanoma in-situ, basal or squamous cell carcinoma of the skin, or carcinoma in-situ of the cervix) within the past 24 months.

29. Female patients who are pregnant or breastfeeding or male or female patients of reproductive potential who are not willing to employ highly effective birth control from screening to 180 days after the last dose of durvalumab + tremelimumab combination therapy or 90 days after the last dose of durvalumab monotherapy, whichever is the longer time period.

30. Documented active alcohol or drug abuse.

31. Patients with a prior history of non-compliance with medical regimens.

32. Any condition that, in the opinion of the investigator, would interfere with evaluation of study treatment or interpretation of patient safety or study results.

4.3 Withdrawal of Subjects from Study Treatment and/or Study

Permanent discontinuation of investigational product/study treatment

An individual subject will not receive any further investigational product if any of the following occur in the subject in question:

1. Withdrawal of consent or lost to follow-up.

2. Adverse event that, in the opinion of the investigator or the sponsor, contraindicates further dosing.

3. Subject is determined to have met one or more of the exclusion criteria for study participation at study entry and continuing investigational therapy might constitute a safety risk.

4. Pregnancy or intent to become pregnant.

5. Any AE that meets criteria for discontinuation as defined in Section 10.3.

6. Grade ≥ 3 infusion reaction.

7. Subject noncompliance that, in the opinion of the investigator or sponsor, warrants withdrawal; eg, refusal to adhere to scheduled visits.

8. Initiation of alternative anticancer therapy including another investigational agent.

9. Confirmed PD by RECIST 1.1 (CT scan or MRI at 4-6 weeks that confirms progression) and investigator determination that the subject is no longer benefiting from treatment with durvalumab + tremelimumab. Patients with documented tumor progression by image (all time-points but especially at 12 weeks), the progression should be confirmed with an additional imaging test (same characteristics) within 4-6 weeks. Treatment should continue according to protocol, until disease progression is confirmed, according the aforementioned procedure.

10. No restarts are allowed after a delay of more than 12 weeks due to toxicity or more than 4 weeks of delay if **the reason is different from toxicity** without consulting the Coordinating Investigator.

Subjects who are permanently discontinued from further receipt of investigational product, regardless of the reason (withdrawal of consent, due to an AE, other), will be identified as having permanently discontinued treatment.

Subjects who are permanently discontinued from receiving investigational product will be followed for safety per Section 10.0 and Appendix 1 or 2, including the collection of any protocol-specified blood specimens, unless consent is withdrawn or the subject is lost to follow-up or enrolled in another clinical study. All subjects will be followed for survival. Subjects who decline to return to the site for evaluations will be offered follow-up by phone every 3 months as an alternative.

Withdrawal of consent

If consent is withdrawn, the subject will not receive any further investigational product or further study observation.

4.4 Replacement of subjects

If a patient withdraws from participation in the study, then his or her enrollment/allocation code cannot be reused. Withdrawn patients will not be replaced.

5. INVESTIGATIONAL PRODUCT(s)

5.1 Durvalumab and tremelimumab

The sponsor will supply durvalumab and tremelimumab to the site pharmacies as a solution for infusion after dilution.

5.1.1 Formulation/packaging/storage

The trial medication (i.e., Durvalumab and Tremelimumab) and its packaging will be labeled in accordance with annex 13 of EU to Good Manufacturing Practice.

Durvalumab

Durvalumab will be supplied as a 500-mg vial solution for infusion after dilution. The solution contains 50 mg/mL durvalumab, 26 mM histidine/histidine-hydrochloride, 275 mM trehalose dihydrate, and 0.02% (weight/volume) polysorbate 80; it has a pH of 6.0. The nominal fill volume is 10 mL. Investigational product vials are stored at 2°C to 8°C (36°F to 46°F) and must not be frozen. Durvalumab must be used within the individually assigned expiry date on the label.

Tremelimumab

Tremelimumab will be supplied as a 400-mg vial solution for infusion after dilution. The solution contains 20 mg/mL of tremelimumab, 20 mM histidine/histidine hydrochloride, 222 mM trehalose dihydrate, 0.02% (w/v) polysorbate 80, and 0.27 mM disodium edetate dihydrate (EDTA);it has a pH of 5.5.The nominal fill volume is 20 mL. Investigational product vials are stored at 2°C to 8°C (36°F to 46°F) and must not be frozen. Tremelimumab must be used within the individually assigned expiry date on the label.

5.2 Dose and treatment regimens

5.2.1 Treatment regimen

Patients will receive 1500 mg durvalumab via IV infusion q4w for up to 4 doses/cycles and 75 mg tremelimumab via IV infusion q4w for up to 4 doses/cycles, and then continue 1500 mg durvalumab q4w starting on Week 16 for up to 8 months (9 doses). Dosing outside the window should be discussed with the Study Physician. Tremelimumab will be administered first. Durvalumab infusion will start approximately 1 hour after the end of tremelimumab infusion. The duration will be approximately 1 hour for each infusion. A 1-hour observation period is required after the first infusion of durvalumab and tremelimumab. If no clinically significant infusion reactions are observed during or after the first cycle, subsequent infusion observation periods can be at the Investigator's discretion (suggested 30 minutes after each durvalumab and tremelimumab infusion).

5.2.2 Duration of treatment and criteria for retreatment

Retreatment is allowed (once only) for patients meeting the retreatment criteria below. The same treatment guidelines followed during the initial 12-month treatment period will be followed during the retreatment period, including the same dose and frequency of treatments and the same schedule of assessments.

Patients receiving the combination of durvalumab and tremelimumab may undergo retreatment in 2 clinical scenarios, described below:

1. Patients who achieve and maintain disease control (ie, CR, PR, or SD) through to the end of the 12-month treatment period may restart treatment with the combination upon evidence of PD, with or without confirmation according to RECIST 1.1, during follow-up.

2. Patients who complete the 4 dosing cycles of the combination of durvalumab and tremelimumab portion of the regimen (with clinical benefit per Investigator judgment), but subsequently have evidence of PD during the durvalumab monotherapy portion of the combination regimen, with or without confirmation according to RECIST 1.1, may restart treatment with the combination.

Before restarting their assigned treatment, the Investigator should ensure that the patient:

1. Does not have any significant, unacceptable, or irreversible toxicities that indicate continuing treatment will not further benefit the patient.

2. Still fulfills the eligibility criteria for this study, including re-consenting to restart durvalumab and tremelimumab.

3. Has not had received an intervening systemic anticancer therapy after their assigned treatment discontinuation.

4. Has had a baseline tumor assessment within 28 days of restarting their assigned treatment; all further scans should occur with the same frequency as during the initial 12 months of treatment (relative to the date of randomization) until study treatment is stopped (maximum of 12 months of further treatment).

During the retreatment period, patients receiving durvalumab + tremelimumab may resume durvalumab dosing at 1500 mg q4w with 75 mg of tremelimumab q4w for 4 doses each. Patients will then continue with durvalumab monotherapy at 1500 mg q4w, beginning at Week 16, 4 weeks after the last dose of combination therapy (a total of 9 additional doses).

Treatment through progression is at the Investigator's discretion, and the Investigator should ensure that patients do not have any significant, unacceptable, or irreversible toxicities that indicate that continuing treatment will not further benefit the patient. A patient with a confirmed progression receiving durvalumab + tremelimumab cannot continue therapy or obtain retreatment if dosing is ongoing in the combination portion of therapy (q4w dosing) and progression occurs in a target lesion that has previously shown a confirmed response.

Patients who the sponsor and/or the Investigator determine may not continue treatment will enter follow-up.

5.2.3 Study drug preparation of durvalumab and tremelimumab

Based on average body WT of 75 kg, a fixed dose of 750 mg Q2W durvalumab (equivalent to 10 mg/kg Q2W), 1500 mg Q4W durvalumab (equivalent to 20 mg/kg Q4W) and 75 mg Q4W tremelimumab (equivalent to 1 mg/kg Q4W) is included in the current study.

Preparation of durvalumab doses for administration with an IV bag

The dose of durvalumab for administration must be prepared by the Investigator's or site's designated IP manager using aseptic technique. Total time from needle puncture of the durvalumab vial to the start of administration should not exceed:

- 24 hours at 2°C to 8°C
- 4 hours at room temperature

If in-use storage time exceeds these limits, a new dose must be prepared from new vials. Infusion solutions must be allowed to equilibrate to room temperature prior to commencement of administration.

No incompatibilities between durvalumab and polyvinylchloride or polyolefin IV bags have been observed. Dose of 1500mg durvalumab for patients ≥30 kg will be administered using an IV bag

containing 0.9% (w/v) saline (compatibility with dextrose has been established and may be used as a diluent option, if preferred), with a final durvalumab concentration ranging from 1 to 20 mg/mL, and delivered through an IV administration set with a 0.2- or 0.22-µm in-line filter. Remove 30.0 mL of IV solution from the IV bag prior to addition of durvalumab. Next, 30.0 mL of durvalumab (ie, 1500 mg of durvalumab) is added to the IV bag such that final concentration is within 1 to 20 mg/mL (IV bag volumes 100 to 1000 mL). Mix the bag by gently inverting to ensure homogeneity of the dose in the bag.

Patient weight at baseline should be used for dosing calculations unless there is a ≥10% change in weight. Dosing day weight can be used for dosing calculations instead of baseline weight per institutional standard.

For patients <30kg, Calculate the dose volume of durvalumab and tremelimumab and number of vials needed for the subject to achieve the accurate dose.

Durvalumab will be administered at room temperature (approximately 25°C) by controlled infusion via an infusion pump into a peripheral or central vein. Following preparation of durvalumab, the entire contents of the IV bag should be administered as an IV infusion over approximately 60 minutes (±5 minutes), using a 0.2, or 0.22-µm in-line filter. Less than 55 minutes is considered a deviation.

The IV line will be flushed with a volume of IV solution (0.9% [w/v] saline equal to the priming volume of the infusion set used after the contents of the IV bag are fully administered, or complete the infusion according to institutional policy to ensure the full dose is administered and document if the line was not flushed.

Standard infusion time is 1 hour. However, if there are interruptions during infusion, the total allowed time should not exceed 8 hours at room temperature. The table below summarizes time allowances and temperatures.

Durvalumab hold and infusion times

Maximum time from needle puncture to start of administration	4 hours at room temperature, 24 hours at 2°C to 8°C
Maximum time for IV bag infusion, including interruptions	8 hours at room temperature

In the event that either preparation time or infusion time exceeds the time limits outlined in the table, a new dose must be prepared from new vials. Durvalumab does not contain preservatives, and any unused portion must be discarded.

Preparation of tremelimumab doses for administration with an IV bag

The dose of tremelimumab for administration must be prepared by the Investigator's or site's designated IP manager using aseptic technique. Total time from needle puncture of the tremelimumab vial to the start of administration should not exceed:

- 24 hours at 2°C to 8°C
- 4 hours at room temperature

It is recommended that the prepared final IV bag be stored in the dark at 2ºC-8°C until needed. If storage time exceeds these limits, a new dose must be prepared from new vials. The refrigerated

infusion solutions in the prepared final IV bag should be equilibrated at room temperature for about 2 hours prior to administration. Tremelimumab does not contain preservatives and any unused portion must be discarded.

No incompatibilities between tremelimumab and polyvinylchloride or polyolefin IV bags have been observed. Doses of 75 mg tremelimumab for patients ≥30 kg will be administered using an IV bag containing 0.9% (w/v) saline, with a final tremelimumab concentration ranging from 0.1 mg/mL to 10 mg/mL, and delivered through an IV administration set with a 0.2 μm or 0.22 μm in-line filter. Remove 3.8 mL of IV solution from the IV bag prior to addition of tremelimumab. Next, 3.8 mL of tremelimumab (ie, 75 mg of tremelimumab) is added to the IV bag such that final concentration is within 0.1 mg/mL to 10 mg/mL (IV bag volumes 50 to 500 mL). Mix the bag by gently inverting to ensure homogeneity of the dose in the bag.

Patient weight at baseline should be used for dosing calculations unless there is a ≥10% change in weight. Dosing day weight can be used for dosing calculations instead of baseline weight per institutional standard.

For patients <30 kg, Calculate the dose volume for tremelimumab and number of vials needed for subject to achieve the accurate dose.

Tremelimumab will be administered at room temperature (approximately 25°C) by controlled infusion via an infusion pump into a peripheral or central vein. Following preparation of tremelimumab, the entire contents of the IV bag should be administered as an IV infusion over approximately 60 minutes (±5 minutes), using a 0.2, or 0.22-μm in-line filter. Less than 55 minutes is considered a deviation.

The IV line will be flushed with a volume of 0.9% (w/v) saline equal to the priming volume of the infusion set used after the contents of the IV bag are fully administered, or complete the infusion according to institutional policy to ensure the full dose is administered and document if the line was not flushed.

Standard infusion time is 1 hour. However, if there are interruptions during infusion, the total allowed time should not exceed 8 hours at room temperature. The table below summarizes time allowances and temperatures.

Tremelimumab hold and infusion times

Maximum time from needle puncture to start of administration	4 hours at room temperature, 24 hours at 2ºC to 8ºC
Maximum time for IV bag infusion, including interruptions.	8 hours at room temperature

In the event that either preparation time or infusion time exceeds the time limits outlined in the table, a new dose must be prepared from new vials. Tremelimumab does not contain preservatives, and any unused portion must be discarded.

5.2.4 Monitoring of dose administration

Patients will be monitored during and after the infusion with assessment of vital signs at the times specified in the Study Protocol.

In the event of a ≤ Grade 2 infusion-related reaction, the infusion rate of study drug may be decreased by 50% or interrupted until resolution of the event and re-initiated at 50% of the initial

rate until completion of the infusion. For patients with a ≤Grade 2 infusion-related reaction, subsequent infusions may be administered at 50% of the initial rate. Acetaminophen and/or an antihistamine (eg, diphenhydramine) or equivalent medications per institutional standard may be administered at the discretion of the investigator. If the infusion-related reaction is ≥Grade 3 or higher in severity, study drug will be discontinued.

As with any antibody, allergic reactions to dose administration are possible. Appropriate drugs and medical equipment to treat acute anaphylactic reactions must be immediately available, and study personnel must be trained to recognize and treat anaphylaxis. The study site must have immediate access to emergency resuscitation teams and equipment in addition to the ability to admit patients to an intensive care unit if necessary.

5.2.5 Accountability and dispensation

The trial medication will be sent to the investigator's site pharmacy preceded by the Regulatory Green Light. The medication is to be used exclusively in the clinical trial according to the instructions of this trial protocol.

When a drug shipment is received, the Investigator or designee will check the amount and condition of the delivery, drug expiration date, and sign the Receipt of Shipment Form provided. The Receipt of Shipment Form should be faxed or emailed to the CRO (see appendix 8). The original form will preliminarily be retained at the site and will be collected at the next monitoring visit by the monitor and stored in the Trial Master File at CRO. A copy remains in the Investigator File at the site. In case of shipment problems the Investigator or designee shall contact the CRA as soon as possible.

An Investigational Product Accountability Log will be provided for the trial medication. The record must be continuously updated and contain the dates, quantities and compounds of drugs received, medication identification number(s), the patient identification number to whom the trial medication was dispensed, date and quantity of medication dispense and the initials of the dispenser.

5.2.6 Disposition of unused investigational study drug

Trial medication will be monitored by the CRA at the respective hospital pharmacy prior to destruction after having completed a final inventory. Local or institutional regulations may require immediate destruction of the study drug used for safety reasons, e.g., cytotoxicity or to maintain the storage capacity and functionality of the storage at the site. In these cases, it may be acceptable to destroy it by the research staff, including partially used and empty vials, dispensed before a monitoring inspection, if the verification of original documents of empty boxes that indicate the information of batch number and dispensing date to the patient on the label. This documentation will be verified against the quantity shipped, dispensed, returned and destroyed.

Prior to the destruction a final trial medication reconciliation statement must be completed. Drug supplies will be destroyed according to the legal requirements in Spain.

All trial medication inventory forms must be made available for inspection by a Sponsor authorized representative or designee and regulatory agency inspectors. The Investigator is responsible for the accountability of all used and unused study supplies at the site.

6. TREATMENT PLAN

6.1 Subject enrollment and allocation

6.1.1 Procedures for allocation

Patients will be allocated in one of the following cohorts regarding primary tumor site and WHO grading classification:

- Cohort 1: Well-moderately differentiated lung neuroendocrine tumors (classically known as typical and atypical carcinoids) after progression to somatostatin analogs and one prior targeted therapy or chemotherapy.
- Cohort 2: G1/G2 (WHO grade 1 and 2) gastrointestinal neuroendocrine tumors after progression to somatostatin analogs and one prior targeted therapy.
- Cohort 3: G1/G2 (WHO grade 1 and 2) pancreatic neuroendocrine tumors after progression to standard therapies (chemotherapy, somatostatin analogs and target therapy), who have received between two and four prior lines of treatment.
- Cohort 4: Neuroendocrine neoplasms (WHO grade 3) of gastroenteropancreatic origin or unknown primary site (excluding lung primary tumors) after progression to first-line chemotherapy with a platinum based regimen. Patients will be treated in second line.

6.1.2 Procedures for handling subjects incorrectly enrolled

Patients incorrectly enrolled in the trial will be considered as protocol deviations. If investigator considerer that the patient is obtaining benefit of the investigational treatment, patients could continue on therapy. They will not be considered for the primary endpoint of the trial, neither for efficacy nor biomarker secondary and exploratory analyses. Patients incorrectly enrolled that continue on treatment can be assessed for safety profile endpoint.

6.2 Dose Modification and Toxicity Management

6.2.1 Durvalumab and tremelimumab

For adverse events (AEs) that are considered at least partly due to administration of durvalumab and/or tremelimumab the following dose adjustment guidance may be applied:

- Treat each of the toxicities with maximum supportive care (including holding the agent suspected of causing the toxicity where required).
- If the symptoms promptly resolve with supportive care, consideration should be given to continuing the same dose of durvalumab or tremelimumab along with appropriate continuing supportive care. If medically appropriate, dose modifications are permitted for durvalumab and tremelimumab (see Appendix 1).
- All dose modifications should be documented with clear reasoning and documentation of the approach taken.

In addition, there are certain circumstances in which durvalumab or tremelimumab should be permanently discontinued.

Following the first dose of durvalumab or tremelimumab, subsequent administration of durvalumab or tremelimumab can be modified based on toxicities observed (see Appendix 1).

Based on the mechanism of action of durvalumab or tremelimumab leading to T-cell activation and proliferation, there is the possibility of observing immune related Adverse Events (irAEs) during the conduct of this study. Potential irAEs include immune-mediated enterocolitis, dermatitis, hepatitis/hepatotoxicity, endocrinopathy, pneumonitis, nephritis, pancreatitis and neuropathy or neurologic events. Subjects should be monitored for signs and symptoms of irAEs. In the absence of an alternate etiology (e.g., infection or PD) signs or symptoms of enterocolitis, dermatitis, hepatitis/hepatotoxicity, endocrinopathy, pneumonitis, nephritis, pancreatitis and neuropathy or neurologic events should be considered to be immune-related.

Dose modification recommendations and toxicity management guidelines for immune-mediated reactions, for infusion-related reactions, and for non-immune-mediated reactions are detailed in Appendix 1.

In addition, management guidelines for adverse events of special interest (AESIs) are detailed in Section 10.1.3. All toxicities will be graded according to NCI CTCAE v4.03.

7. RESTRICTIONS DURING THE STUDY AND CONCOMITANT TREATMENT(s)

7.1 Restrictions during the study

The following restrictions apply while the patient is receiving study treatment and for the specified times before and after:

- Female subjects of childbearing potential who are sexually active with a non-sterilized male partner must use one highly effective method of contraception (Table 2) from the time of screening and must agree to continue using such precautions for 180 days after the last dose of durvalumab + tremelimumab combination therapy or 90 days after the last dose of durvalumab monotherapy, whichever is the longer time period. Male partners of a female subject must use male condom plus spermicide throughout this period. Cessation of birth control after this point should be discussed with a responsible physician. Not engaging in sexual activity for the total duration of the trial and the drug washout period is an acceptable practice; however, occasional abstinence, the rhythm method, and the withdrawal method are not acceptable methods of contraception..

- Females of childbearing potential are defined as those who are not surgically sterile (ie, bilateral tubal ligation, bilateral oophorectomy, or complete hysterectomy) or post-menopausal (defined 12 months with no menses without an alternative medical cause).

- Non-sterilized male subjects who are sexually active with a female partner of childbearing potential must use male condom plus spermicide from screening through 180 days after receipt of the final dose of durvalumab + tremelimumab combination therapy or 90 days after receipt of the final dose of durvalumab monotherapy, whichever is the longer time period. Female partners of a male subject must use a highly effective method of

contraception throughout this period.

- Highly effective methods of contraception are described in Table 2. A highly effective method of contraception is defined as one that results in a low failure rate (i.e. less than 1% per year) when used consistently and correctly. Note that some contraception methods are not considered highly effective (e.g. male or female condom with or without spermicide; female cap, diaphragm, or sponge with or without spermicide; non-copper containing intrauterine device; progestogen-only oral hormonal contraceptive pills where inhibition of ovulation is not the primary mode of action [excluding Cerazette/desogestrel which is considered highly effective]; and triphasic combined oral contraceptive pills).
- Restrictions relating to concomitant medications are described in Section 7.2.

Table 2. Highly Effective[a] Methods of Contraception

Barrier/ Intrauterine	Hormonal Methods
• Copper T intrauterine<br>• Levonorgestrel-relasing intrauterine system (eg, Mirema)[b]	• Etonogestrel implants: e.g. Implanon or Norplan<br>• Intravaginal device; e.g. ethinylestradiol and etonogestrel<br>• Medroxyprogesterone injection: e.g. Depo-Provera<br>• Normal and low dose combined oral contraceptive pill<br>• Norelgestromin/ethinylestradiol transdermal system<br>• Cerazette (desogestrel)

[a] Highly effective (i.e. failure rate of <1% per year) [b] This is also considered a hormonal method

Blood donation

Subjects should not donate blood while participating in this study, or for at least 90 days following the last infusion of durvalumab or tremelimumab, whichever occurs longest.

7.2 Concomitant treatment(s)

The Principal Investigator must be informed as soon as possible about any medication taken from the time of screening until the end of the clinical phase of the study (final study visit). Any concomitant medication(s), including herbal preparations, taken during the study will be recorded in the CRF.

Restricted, prohibited, and permitted concomitant medications are described in the following tables.

7.2.1 Permitted concomitant medications

Investigators may prescribe concomitant medications or treatments (e.g., acetaminophen, diphenhydramine) deemed necessary to provide adequate prophylactic or supportive care except for those medications identified as "excluded" as listed in Section 7.2.2.

Concomitant treatment with somatostatin analogues (octreotide or lanreotide) are allowed for

hormonal symptom control.

7.2.2 Excluded Concomitant Medications

The following medications are considered exclusionary during the study.

1. Any investigational anticancer therapy other than the protocol specified therapies.
2. Any concurrent chemotherapy, radiotherapy (except palliative radiotherapy), immunotherapy, biologic or hormonal therapy for cancer treatment. Concurrent use of hormones for noncancer-related conditions (e.g., insulin for diabetes and hormone replacement therapy) is acceptable. NOTE: Local treatment of isolated lesions for palliative intent is acceptable (e.g., by local surgery or radiotherapy).
3. Immunosuppressive medications including, but not limited to systemic corticosteroids at doses not exceeding 10 mg/day of prednisone or equivalent, methotrexate, azathioprine, and TNF-α blockers. Use of immunosuppressive medications for the management of investigational product-related AEs or in subjects with contrast allergies is acceptable. In addition, use of inhaled and intranasal corticosteroids is permitted. A temporary period of steroids will be allowed for different indications, at the discretion of the principal investigator (e.g., chronic obstructive pulmonary disease, radiation, nausea, etc).
4. Live attenuated vaccines within 30 days of durvalumab and tremelimumab dosing (ie, 30 days prior to the first dose, during treatment with durvalumab and tremelimumab for 30 days post discontinuation of durvalumab and tremelimumab. Inactivated vaccines, such as the injectable influenza vaccine, are permitted.

Table 3. Prohibited and Rescue Medications

Prohibited medication/class of drug	Usage
Additional investigational anticancer therapy concurrent with those under investigation in this study	Should not be given whilst the patient is on IP treatment
mAbs against CTLA-4, PD-1, or PD-L1	Should not be given whilst the patient is on IP treatment through 90 days after the last dose of IP.
Any concurrent chemotherapy, local therapy (except palliative radiotherapy for non-target lesions, eg, radiotherapy, surgery, radiofrequency ablation), biologic therapy, or hormonal therapy for cancer treatment	Should not be given whilst the patient is on IP treatment (including SoC). (Concurrent use of hormones for non-cancer-related conditions [eg, insulin for diabetes and hormone replacement therapy] is acceptable.)
Immunosuppressive medications, including, but not limited to, systemic corticosteroids at doses exceeding 10 mg/day of prednisone or its equivalent, methotrexate, azathioprine, and tumor necrosis factor α blockers	Should not be given whilst the patient is on IP treatment (including SoC). (Use of immunosuppressive medications for the management of IP-related AEs or in patients with contrast allergies is acceptable. In addition, use of inhaled, topical, and intranasal corticosteroids is permitted.
Live attenuated vaccines	Should not be given through 30 days after the last dose of IP (including SoC) during the study.
**Rescue/supportive medication/class of drug**	**Usage**
Concomitant medications or treatments (eg, acetaminophen or diphenhydramine) deemed necessary by the Investigator to provide adequate prophylactic or supportive care, except for those medications identified as “prohibited” as listed above.	To be administered as prescribed by the Investigator
Best supportive care (including antibiotics, nutritional support, growth factor support, correction of metabolic disorders, optimal symptom control, and pain management [including palliative radiotherapy, etc])	Should be used when necessary for all patients

8. STUDY PROCEDURES

8.1 Schedule of study procedures

Before study entry, throughout the study, and following study drug discontinuation, various clinical and diagnostic laboratory evaluations are outlined. The purpose of obtaining these detailed measurements is to ensure adequate safety and tolerability assessments. Clinical evaluations and laboratory studies may be repeated more frequently if clinically indicated. The Schedules of Assessments during the screening and treatment period is provided after the Protocol Synopsis.

8.1.1 Screening Phase

Screening procedures will be performed up to 28 days before Day 1, unless otherwise specified. All subjects must first read, understand, and sign the IEC-approved ICF before any study-specific screening procedures are performed. After signing the ICF, completing all screening procedures, and being deemed eligible for entry, subjects will be enrolled in the study. Procedures performed prior to the signing of the ICF are considered standard of care and may be used as screening assessments if they fall within the 28-day screening window.

The following procedures will be performed during the Screening Visit:

- Informed Consent
- Review of eligibility criteria
- Medical history and demographics
- Complete physical exam
- ECOG Performance Status
- Vital signs, weight and height
- 12-lead ECG (in triplicate [2-5 minutes apart])
- Review of prior/concomitant medications
- Record any Aes or SAEs (since ICF signature)
- Imaging by CT/MRI
- Review of octreoscan/PET (up to 6 months prior inclusion) only on cohorts 1 to 3.
- Collect blood samples for biomarkers analysis
- Archival tumor block or slides
- Clinical laboratory tests for:
    - Hematology (see Table 4)
    - Clinical chemistry (see Table 5)
    - TSH, fT3 and fT4

- Coagulation (PT, PTT, INR)
- Creatinine Clearance
- Serum pregnancy test (for women of childbearing potential only)
- Hepatitis and HIV serologies
- Urinalysis (see Table 6)
- Disease-specific tumor markers

8.1.2 Treatment Phase

Procedures to be conducted during the treatment phase of the study are presented in the Schedule of Assessments. Screening procedures performed within 72 hours of Cycle 1 Day 1 (C1D1) do not need to be repeated on C1D1.

Efforts should be made to conduct study visits on the day scheduled (± 1 day). Clinical laboratory assessments may be conducted anytime within 72 hours prior to the scheduled visit, unless otherwise specified in the Schedule of Visits and Procedures. Results of the laboratory assessments should be reviewed always prior each cycle dose administration. Whenever possible, subjects should be evaluated at approximately the same time of the day (e.g., morning or afternoon) at each visit, and reasonable efforts should be made to conduct all evaluations in the same test order at each visit.

Tumor assessments should be performed at time points indicated in the Schedule of Visits and Procedures.

Cycle 1/Day 1

- Obtain vital signs (resting BP, HR, RR, body temperature) and weight.
- Physical examination is not mandatory if performed at baseline (day -1) however a symptom-directed physical examination will be performed on Cycle 1/Day 1 and at any time during the study, as clinically indicated.
- Administer study drug:
  - Record all concomitant medication use.
  - Record any AEs or SAEs.

Cycle 1/Day 15

- Obtain vital signs (resting BP, HR, RR, body temperature) and weight.
- Evaluate ECOG performance status.
- Perform a comprehensive physical examination (including a neurological evaluation). A symptom-directed physical examination will be performed at any time during the study, as clinically indicated.
- Collect blood samples for biochemistry and hematology analyses (see Tables 4 and 5),

including TSH, fT3 and fT4.

- Urinalysis (see Table 6)
- Record all concomitant medication use.
- Record any AEs or SAEs.

Cycle 2/Day 1

- Evaluate ECOG performance status.
- Obtain vital signs (supine BP, HR, RR, body temperature) and weight.
- Perform a comprehensive physical examination (including a neurological evaluation). A symptom-directed physical examination will be performed at any time during the study, as clinically indicated.
- Collect blood samples for biochemistry and hematology analyses (see Tables 4 and 5), including TSH, fT3 and fT4.
- Urinalysis (see Table 6)
- Administer study drug.
- Collect blood samples for biomarker analysis.
- Record all concomitant medication use.
- Record any AEs or SAEs.
- Record survival data.

Cycle 2/Day 15

- Evaluate ECOG performance status.
- Obtain vital signs (supine BP, HR, RR, body temperature) and weight.
- Perform a comprehensive physical examination (including a neurological evaluation). A symptom-directed physical examination will be performed at any time during the study, as clinically indicated.
- Collect blood samples for biochemistry and hematology analyses (see Tables 4 and 5), including TSH, fT3 and fT4.
- Urinalysis (see Table 6)
- Record all concomitant medication use.
- Record any AEs or SAEs.

Cycle 3/Day 1

- Evaluate ECOG performance status.

- Obtain vital signs (supine BP, HR, RR, body temperature) and weight.
- Perform a comprehensive physical examination (including a neurological evaluation). A symptom-directed physical examination will be performed at any time during the study, as clinically indicated.
- Collect blood samples for biochemistry and hematology analyses (see Tables 4 and 5), including TSH, fT3 and fT4.
- Urinalysis (see Table 6)
- Administer study drugs.
- Record all concomitant medication use.
- Record any AEs or SAEs.
- Record Survival data

Cycle 3/Day 15

- Evaluate ECOG performance status.
- Obtain vital signs (supine BP, HR, RR, body temperature) and weight.
- Perform a comprehensive physical examination (including a neurological evaluation). A symptom-directed physical examination will be performed at any time during the study, as clinically indicated.
- Collect blood samples for biochemistry and hematology analyses (see Tables 4 and 5), including TSH, fT3 and fT4.
- Urinalysis (see Table 6)
- Record all concomitant medication use.
- Record any AEs or SAEs.

Cycle 4 Through Last Cycle (cycle 12)/Day 1

- Evaluate ECOG performance status.
- Obtain vital signs (supine BP, HR, RR, body temperature) and weight.
- Perform a comprehensive physical examination (including a neurological evaluation). A symptom-directed physical examination will be performed at any time during the study, as clinically indicated.
- Collect blood samples for biochemistry and hematology analyses (see Tables 4 and 5), including TSH, fT3 and fT4
- Urinalysis (see Table 6)
- Administer study drugs. (from C5D1 to C12D1 tremelimumab no longer administered, only durvalumab).

- Record all concomitant medication use.
- Record any AEs or SAEs.
- Record survival data.
- Tumor assessment every 12 weeks until confirmed PD.

8.1.3 End of Treatment

End of treatment is defined as the last planned dosing visit within the 12-month dosing period. For subjects who discontinue durvalumab or tremelimumab prior to 12 months, end of treatment is considered the last visit where the decision is made to discontinue treatment. All required procedures may be completed within ± 7 days of the end of treatment visit. Repeat disease assessment is not required if performed within 28 days prior to the end of treatment visit.

Blood samples for biomarker analysis are collected.

Assessments for subjects who have completed durvalumab and tremelimumab treatment and achieved disease control, or have discontinued durvalumab or tremelimumab due to toxicity in the absence of confirmed progressive disease are provided in APPENDIX 2.

Assessments for subjects who have discontinued durvalumab or tremelimumab treatment due to confirmed PD are presented in APPENDIX 3.

All subjects will be followed for survival until the end of the study regardless of further treatments, or until the sponsor ends the study.

Follow-up period

TC/MRI every 12 weeks until progression. Monthly visits during the first 3 months after end of treatment, every 2 months until one year, and therefore every 6 months. Determinations to be done are listed in appendix 2 and 3.

8.2 Description of study procedures

8.2.1 Medical history and physical examination, electrocardiogram, weight and vital signs

Findings from medical history (obtained at screening) and physical examination shall be given a baseline grade according to the procedure for AEs. Increases in severity of pre-existing conditions during the study will be considered AEs, with resolution occurring when the grade returns to the pre-study grade or below.

Physical examinations will be performed on study days noted in the Schedule of Assessments.

8.2.2 Physical examination

Physical examinations will be performed according to the assessment schedule. Full physical examinations will include assessments of the head, eyes, ears, nose, and throat and the respiratory, cardiovascular, GI, urogenital, musculoskeletal, neurological, dermatological, hematologic/lymphatic, and endocrine systems. Height will be measured at Screening only. Targeted physical examinations are to be used by the Investigator on the basis of clinical

observations and symptomatology. Situations in which physical examination results should be reported as AEs are described in Section 10.

8.2.3 Electrocardiograms

Resting 12-lead ECGs will be recorded at screening and as clinically indicated throughout the study. ECGs should be obtained after the patient has been in a supine position for 5 minutes and recorded while the patient remains in that position.

At Screening, a single ECG will be obtained on which QTcF must be <470 ms.

In case of clinically significant ECG abnormalities, including a QTcF value >470 ms, 2 additional 12-lead ECGs should be obtained over a brief period (eg, 30 minutes) to confirm the finding.

Situations in which ECG results should be reported as AEs are described in Section 10.0.

8.2.4 Vital signs

Vital signs (blood pressure [BP], pulse, temperature, and respiration rate) will be evaluated according to the assessment schedules.

On infusion days, patients receiving durvalumab + tremelimumab treatment will be monitored during and after infusion of IP as presented in the bulleted list below.

Supine BP will be measured using a semi-automatic BP recording device with an appropriate cuff size, after the patient has rested for at least 5 minutes. BP and pulse will be collected from patients receiving durvalumab + tremelimumab treatment before, during, and after each infusion at the following times (based on a 60-minute infusion):

Prior to the beginning of the infusion (measured once from approximately 30 minutes before up to 0 minutes [ie, the beginning of the infusion]).

Approximately 30 minutes during the infusion (**halfway** through infusion).

At the end of the infusion (approximately 60 minutes ±5 minutes).

A 1-hour observation period is required after the first infusion of durvalumab and tremelimumab.

If no clinically significant infusion reactions are observed during or after the first cycle, subsequent infusion observation periods can be at the Investigator's discretion (suggested 30 minutes after each durvalumab and tremelimumab infusion).

If the infusion takes longer than 60 minutes, then BP and pulse measurements should follow the principles as described above or be taken more frequently if clinically indicated. The date and time of collection and measurement will be recorded on the appropriate eCRF. Additional monitoring with assessment of vital signs is at the discretion of the Investigator per standard clinical practice or as clinically indicated.

Body weight is also recorded along with vital signs.

Situations in which vital signs results should be reported as AEs are described in Section 10.3. A complete physical examination will be performed and will include an assessment of the following (as clinically indicated): general appearance, respiratory, cardiovascular, abdominal, skin, head and

neck (including ears, eyes, nose and throat), lymph nodes, thyroid, musculoskeletal (including spine and extremities), genital/rectal, and neurological systems and at screening only, height.

8.2.5 Clinical laboratory tests

The following clinical laboratory tests will be performed in local hospitals (see the Schedule of Assessments):

- Coagulation parameters: Activated partial thromboplastin time and International normalised ratio to be assessed at baseline and as clinically indicated
- Pregnancy test (female subjects of childbearing potential only)
  - Urine human chorionic gonadotropin
  - Serum beta-human chorionic gonadotropin (at screening only)
- Thyroid Stimulating Hormone
  - Free T3 and free T4 only if TSH is abnormal
- Other laboratory tests
  - Hepatitis B surface antigen, hepatitis C antibody
  - HIV antibody

Table 4. Hematology Laboratory Tests

Basophils	Mean corpuscular volume	Total white cell count
Eosinophils	Monocytes	Mean corpuscular hemoglobin concentration
Hematocrit	Neutrophils	Mean corpuscular hemoglobin
Hemoglobin	Platelet count	
Lymphocytes	Red blood cell count	

Table 5. Clinical chemistry (Serum or Plasma) Laboratory Tests

Albumin	Glucose
Alkaline phosphatase	Lactate dehydrogenase
Alanine aminotransferase	Lipase
Amylase	Magnesium
Aspartate aminotransferase	NSE (Enolase)
Bicarbonate	Potassium
Calcium	Sodium
CgA (Chromogranine)	Total bilirubin[a]
Chloride	Total protein
Creatinine	Urea or blood urea nitrogen, depending on local practice
Gamma glutamyltransferase[b]	Uric acid

[a] If Total bilirubin is ≥2xULN (and no evidence of Gilbert's syndrome) then fraction into direct and indirect bilirubin
[b] At baseline and as clinically indicated.

Table 6. Urinalysis Tests [a]

Bilirubin	pH
Blood	Protein
Glucose	Specific gravity
Ketones	Colour and appearance

[a] Microscopy should be used as appropriate to investigate white blood cells and use the high power field for red blood cells

8.3 Biological sampling procedures

Blood samples for the development of exploratory predictive biomarkers will be collected from all subjects prior to the first dose of study drug, and on Cycle 2/Day 1 and at end-of treatment visit or after documented disease progression, whichever occurs first. Biomarker discovery and validation will be performed to identify blood or tumor biomarkers which may be useful to predict subject response, as determined by evaluation of primary or secondary efficacy endpoints. Plasma samples from study subjects will undergo global proteomic and/or enzyme-linked immunosorbent assay (ELISA)-based analyses or multiplex bead-based immunoassay in an effort to identify protein biomarkers. In addition, DNA and RNA analyses will be performed in search of predictive or prognostic biomarkers and also biomarkers identified in other durvalumab+trametinib clinical studies may also be assessed in samples collected from subjects enrolled in this study.

Archived, fixed tumor tissue from will be collected for all subjects for confirmation of histology and assessment of somatic mutations of genes which may be important in the development and progression of neuroendocrine tumors. Gene-expression profiling (GEP), proteomic, or immunohistochemical (IHC) analysis will be performed based on the amount of tumor tissue available for analysis. All analyses will be limited to correlations relevant to neuroendocrine tumors and clinical outcomes related to treatment with durvalumab and tremelimumab.

Blood and tumor samples collected during the study will be stored at the study sites until the initial primary efficacy and safety analyses of the study will be completed and results will be available. Then, the study Sponsor will decide whether to perform all or part of the pharmacogenetic/pharmacogenomics assessments.

Data obtained will only be used for research, to assist in developing safer and more effective treatments, and will not be used to change the diagnosis of the subject or alter the therapy of the subject. Any DNA derived from the sample may be stored for up to 15 years to assist in any research scientific questions related to durvalumab or tremelimumab. Sample collection will be registered in Spanish National Registry and will be used in research projects in the particular research line foresee in the informed consent. Samples cannot be transferred to third parties, and studies in the future with these samples will require EC approval. Instructions for the processing, storage, and shipping of samples will be provided in the Laboratory Manual.

8.3.1 Biomarker/Pharmacodynamic sampling and evaluation methods

PD-L1 Testing

Retrospectively, PD-L1 assessment will be performed using Ventana SP263 assay in accordance with the package insert on the Ventana Benchmark platform (Ultra or XT) within the GETNE translational working group to ensure comparability of data across all studies of durvalumab and/or tremelimumab.

Sample collection for PD-L1 testing

- The preferred tumor sample for the determination of a patient's PD-L1 status is the one taken following the completion of the most recent prior line of therapy. Samples taken at this time reflect the current PD-L1 status of the tumor and considered clinically most relevant.

- The preferred sample for PD-L1 testing was less than or equal to 3 months old. In cases where a sample a less than 3 months old was not available, patients were asked to undergo a new biopsy if considered clinically appropriate by their treating physician.
- Samples should be collected via a core needle of 18 gauge or larger or be collected by an incisional or excisional tumor biopsy. The provision of 2 cores is advised in order to provide sufficient tissue for PD-L1 assessment and processed to FFPE in a single block. Where institutional practice uses a smaller gauge needle, samples should be evaluated for tumor cell quantity (i.e. >100 tumor cells) to allow for adequate PD-L1 immunohistochemistry analyses.
- When the collection of a new sample is not clinically appropriate, archival samples may be utilized provided the specimen it is not older than 5 years of age. When archival samples are used to assess PD-L1 status, the age of the sample / date of collection should be captured.
- Samples submitted for PD-L1 testing should be formalin fixed and embedded in paraffin. Samples from fine needle aspirates (FNA) or decalcified bone are not appropriate for PD-L1 analysis.

Sample data collection for PD-L1 testing

The following fields of data should be collected from the site/institution collecting and if, indicated shipping of the samples:

Patient identifier (e-code or unique identifier)

- Specimen identifier (written on the specimen)
- Site identifier
- Specimen collection date
- Type of specimen submitted
- Quantity of specimen
- Date of sectioning
- Archival of fresh tumor
- Tumor type
- Primary tumor location
- Metastatic tumor location (if applicable)
- Fixative

The following fields of data should be collected from PD-L1 testing laboratory:

- Are the negative and positive controls stained correctly
- Is the H&E material acceptable

- Is morphology acceptable
- Total percent positivity of PD-L1 in tumor cells
- PD-L1 status (positive, negative or NA) in tumor cells
- Total percent positivity of PD-L1 in infiltrating immune cells

Previously frozen tissue is not acceptable for processing to FFPE for PD-L1 testing. Sections should be stored at ambient temperature and protected from light until use or shipment to testing lab by courier at ambient temperature. It is recommended that slides are cut freshly prior to PD-L1 testing and they are used within 90 days of being cut to obtain PD-L1 status

8.3.2 Estimate of volume of blood to be collected

The total of two blood samples will be drawn from each subject in this study (at baseline, C2D1, end of treatment/progression) will be managed according the laboratory manual and stored -80ºC (-20ºC if not available) in the investigators centers. At the end of the study treatment period and follow-up up, and regarding results obtained, the sponsor and investigators will decide if biomarker analyses will be performed.

8.3.3 Archival tumor samples

8.3.3.1 Archival tumor samples

Archival tumor samples in formalin-fixed paraffin-embedded tissue block will be identified at screening period. Samples will only be used at the end of the study treatment and always regarding results obtained with the investigational drugs.

8.3.4 Withdrawal of informed consent for donated biological samples

If a subject withdraws consent to the use of donated samples, the samples will be disposed of/destroyed, and the action documented.

Participation in biological samples analyses are optional, and patients can participate in the study without giving the consent to use their biological samples.

The Principal Investigator:

- Ensures that biological samples from that subject, if stored at the study site, are immediately identified, disposed of /destroyed, and the action documented
- Ensures the laboratory(ies) holding the samples is/are informed about the withdrawn consent immediately and that samples are disposed/destroyed, the action documented and the signed document returned to the study site
- Ensures that the subject is informed about the sample disposal.

9. DISEASE EVALUATION AND METHODS

Primary Objective

Disease evaluation according RECIST 1.1.

A copy of the RECIST 1.1 guideline will be included in the ISF.

RECIST 1.1 will be modified so that PD must be confirmed at the next scheduled visit, preferably, and no earlier than 4 weeks after the initial assessment of PD in the absence of clinically significant deterioration. Treatment with durvalumab + tremelimumab would continue between the initial assessment of progression and confirmation for progression.

Secondary objectives (irRECIST)

The response to immunotherapy may differ from the typical responses observed with cytotoxic chemotherapy including the following (Wolchok et al 2009, Nishino et al 2013):

- Response to immunotherapy may be delayed
- Response to immunotherapy may occur after PD by conventional criteria
- The appearance of new lesions may not represent PD with immunotherapy
- SD while on immunotherapy may be durable and represent clinical benefit.

Based on the above-described unique response to immunotherapy and based on guidelines from regulatory agencies, e.g., European Medicines Agency's "Guideline on the evaluation of anti-cancer medicinal products in man" (EMA/CHMP/205/95/Rev.4) for immune modulating anti-cancer compounds, the study may wish to implement the following in addition to standard RECIST 1.1 criteria:

- RECIST 1.1 will be modified so that PD must be confirmed at the next scheduled visit, preferably, and no earlier than 4 weeks after the initial assessment of PD in the absence of clinically significant deterioration. Treatment with durvalumab + tremelimumab would continue between the initial assessment of progression and confirmation for progression.
- In addition, subjects may continue to receive durvalumab + tremelimumab beyond confirmed PD in the absence of clinically significant deterioration and if investigators consider that subjects continue to receive benefit from treatment.

Progression would be considered confirmed if the following criteria are met:

- ≥20% increase in the sum diameters of target lesions compared with the nadir at 2 consecutive visits with an absolute increase of 5 mm, and/or
- Clinically significant progression (worsening) of non-target lesions or new lesions at the confirmatory PD time point compared with the first time point where progression of non-target lesions or new lesions was identified, and/or
- Additional new unequivocal lesions at the confirmatory PD time point compared with the first time point at which new lesions were identified.

In the absence of clinically significant deterioration, the Investigator should continue study treatment until progression is confirmed.

If progression is not confirmed, then the patient should continue on study treatment and on treatment assessments.

Modification of RECIST as described may discourage the early discontinuation of durvalumab + tremelimumab and provide a more complete evaluation of its anti-tumor activity than would be seen with conventional response criteria. Nonetheless, the efficacy analysis will be conducted by programmatically deriving each efficacy endpoint based on RECIST 1.1 criteria.

Of note, clinically significant deterioration is considered to be a rapid tumor progression that necessitates treatment with anti-cancer therapy other than durvalumab + tremelimumab or with symptomatic progression that requires urgent medical intervention (e.g., central nervous system metastasis, respiratory failure due to tumor compression, spinal cord compression).

9.1 Efficacy variable

The RECIST 1.1 criteria will be used to assess patient response to treatment by determining progression-free survival rates at 6, 9 and 12 months, median progression-free survival, overall response rate and median overall survival using Investigator assessments and central radiology review. All images will be collected and quality checked centrally. Guidelines for imaging collection and storage will be provided in a separate document. The management of patients will be based in part upon the results of the RECIST 1.1 assessment conducted by the Investigator.

10. ASSESSMENT OF SAFETY

The Principal Investigator is responsible for ensuring that all staff involved in the study is familiar with the content of this section.

10.1 Safety Parameters

10.1.1 Definition of adverse events

The International Conference on Harmonization (ICH) Guideline for Good Clinical Practice (GCP) E6(R1) defines an AE as:

Any untoward medical occurrence in a patient or clinical investigation subject administered a pharmaceutical product and which does not necessarily have a causal relationship with this treatment. An AE can therefore be any unfavorable and unintended sign (including an abnormal laboratory finding), symptom, or disease temporally associated with the use of a medicinal product, whether or not considered related to the medicinal product.

An AE includes but is not limited to any clinically significant worsening of a subject's pre-existing condition. An abnormal laboratory finding (including ECG finding) that requires an action or intervention by the investigator, or a finding judged by the investigator to represent a change beyond the range of normal physiologic fluctuation, should be reported as an AE.

Adverse events may be treatment emergent (ie, occurring after initial receipt of investigational product) or non treatment emergent. A non treatment-emergent AE is any new sign or symptom,

disease, or other untoward medical event that begins after written informed consent has been obtained but before the subject has received investigational product.

Elective treatment or surgery or pre planned treatment or surgery (that was scheduled prior to the subject being enrolled into the study) for a documented pre-existing condition, that did not worsen from baseline, is not considered an AE (serious or non serious). An untoward medical event occurring during the pre scheduled elective procedure or routinely scheduled treatment should be recorded as an AE or SAE.

The term AE is used to include both serious and non-serious AEs.

10.1.2 Definition of serious adverse events

A serious adverse event is an AE occurring during any study phase (i.e., screening, run-in, treatment, wash-out, follow-up), at any dose of the study drugs that fulfils one or more of the following criteria:

1- Results in death

2- Is immediately life-threatening

3- Requires inpatient hospitalization or prolongation of existing hospitalization

4- Results in persistent or significant disability or incapacity

5- Is a congenital abnormality or birth defect in offspring of the subject

6- Is an important medical event that may jeopardize the patient or may require medical intervention to prevent one of the outcomes listed above:

7- Medical or scientific judgment should be exercised in deciding whether expedited reporting is appropriate in this situation. Examples of medically important events are intensive treatment in an emergency room or at home for allergic bronchospasm, blood dyscrasias, or convulsions that do not result in hospitalizations; or development of drug dependency or drug abuse.

The causality of SAEs (their relationship to all study treatment/procedures) will be assessed by the investigator(s) and communicated to the sponsor.

10.1.3 Durvalumab + tremelimumab adverse events of special interest

An adverse event of special interest (AESI) is one of scientific and medical interest specific to understanding of the Investigational Product and may require close monitoring and rapid communication by the investigator to the sponsor. An AESI may be serious or non-serious. The rapid reporting of AESIs allows ongoing surveillance of these events in order to characterize and understand them in association with the use of this investigational product. A report is required using the SAE form, indicating an event of special interest.

AESIs for durvalumab and tremelimumab include but are not limited to events with a potential inflammatory or immune-mediated mechanism and which may require more frequent monitoring and/or interventions such as steroids, immunosuppressants and/or hormone replacement therapy. These AESIs are being closely monitored in clinical studies with durvalumab monotherapy and

combination therapy. An immune-related adverse event (irAE) is defined as an adverse event that is associated with drug exposure and is consistent with an immune-mediated mechanism of action and where there is no clear alternative aetiology. Serologic, immunologic, and histologic (biopsy) data, as appropriate, should be used to support an irAE diagnosis. Appropriate efforts should be made to rule out neoplastic, infectious, metabolic, toxin, or other etiologic causes of the irAE.

If the Investigator has any questions in regards to an adverse event (AE) being an irAE, the Investigator should promptly contact the Study Physician.

AESIs observed with durvalumab and tremelimumab include:

- Colitis
- Pneumonitis
- ALT/AST increases / hepatitis / hepatotoxicity
- Neuropathy / neuromuscular toxicity (i.e. events of encephalitis, peripheral motor and sensory neuropathies, Guillain-Barré, and myasthenia gravis)
- Endocrinopathy (i.e. events of hypophysitis, adrenal insufficiency, and hyper- and hypothyroidism)
- Dermatitis
- Nephritis
- Pancreatitis (or labs suggestive of pancreatitis - increased serum lipase , increased serum amylase)
- Other inflammatory responses that are rare with a potential immune-mediated aetiology include, but are not limited to, myocarditis, pericarditis, and uveitis.

Further information on these risks (e.g. presenting symptoms) can be found in the current version of the durvalumab and tremelimumab Investigator Brochure.

Guidelines for the management of patients with immune-related AEs (irAEs) are provided in Appendix 1. In addition to the dose modification guidelines provided in Appendix 1, it is recommended that irAEs are managed according to the general treatment guidelines outlined for ipilimumab (Weber et al, 2012). These guidelines recommend the following:

- Patients should be evaluated to identify any alternative etiology.
- In the absence of a clear alternative etiology, all events of an inflammatory nature should be considered immune related.
- Symptomatic and topical therapy should be considered for low-grade events.
- Systemic corticosteroids should be considered for a persistent low-grade event or for a severe event.
- More potent immunosuppressives should be considered for events not responding to systemic steroids (eg, infliximab or mycophenolate).

If the Investigator has any questions in regards to an AE being an irAE, the Investigator should immediately contact the Study Physician.

Infusion reactions

AEs of infusion reactions (also termed infusion-related reactions) are of special interest to AstraZeneca and are defined, for the purpose of this protocol, as all AEs occurring from the start of IP infusion up to 48 hours after the infusion start time. For all infusion reactions, SAEs should be reported to the sponsor as described in Section 10.3.

Hypersensitivity reactions

Hypersensitivity reactions as well as infusion-related reactions have been reported with anti-PD-L1 and anti-PD-1 therapy (Brahmer et al 2012). As with the administration of any foreign protein and/or other biologic agents, reactions following the infusion of mAbs can be caused by various mechanisms, including acute anaphylactic (IgE-mediated) and anaphylactoid reactions against the mAbs and serum sickness. Acute allergic reactions may occur, may be severe, and may result in death. Acute allergic reactions may include hypotension, dyspnea, cyanosis, respiratory failure, urticaria, pruritus, angioedema, hypotonia, arthralgia, bronchospasm, wheeze, cough, dizziness, fatigue, headache, hypertension, myalgia, vomiting, and unresponsiveness. Guidelines for the management of patients with hypersensitivity (including anaphylactic reaction) and infusion-related reactions are provided in Appendix 1.

Hepatic function abnormalities (hepatotoxicity)

Hepatic function abnormality is defined as any increase in ALT or AST to greater than 3 × ULN and concurrent increase in total bilirubin to be greater than 2 × ULN. Concurrent findings are those that derive from a single blood draw or from separate blood draws taken within 8 days of each other. Follow-up investigations and inquiries will be initiated promptly by the investigational site to determine whether the findings are reproducible and/or whether there is objective evidence that clearly supports causation by a disease (eg, cholelithiasis and bile duct obstruction with distended gallbladder) or an agent other than the IP. Guidelines for management of patients with hepatic function abnormality are provided in section 10.3.3.2.

For the consultation of the complete list of adverse events see the last version of IB provided in the ISF.

10.2 Assessment of safety parameters

10.2.1 Assessment of severity

Assessment of severity is one of the responsibilities of the investigator in the evaluation of AEs and SAEs. Severity will be graded according to the NCI CTCAE v4.03.

The determination of severity for all other events not listed in the CTCAE should be made by the investigator based upon medical judgment and the severity categories of Grade 1 to 5 as defined below:

Grade 1 (mild): An event that is usually transient and may require only minimal treatment or therapeutic intervention. The event does not generally interfere with usual activities of daily living.

Grade 2 (moderate): An event that is usually alleviated with additional specific therapeutic

intervention. The event interferes with usual activities of daily living, causing discomfort but poses no significant or permanent risk of harm to the subject.

Grade 3 (severe): An event that requires intensive therapeutic intervention. The event interrupts usual activities of daily living, or significantly affects the clinical status of the subject.

Grade 4 (life threatening): An event, and/or its immediate sequelae, that is associated with an imminent risk of death or with physical or mental disabilities that affect or limit the ability of the subject to perform activities of daily living (eating, ambulation, toileting, etc).

Grade 5 (fatal): Death (loss of life) as a result of an event.

It is important to distinguish between serious criteria and severity of an AE. Severity is a measure of intensity whereas seriousness is defined by the criteria in Section 9.2.1. A Grade 3 AE need not necessarily be considered an SAE. For example, a Grade 3 headache that persists for several hours may not meet the regulatory definition of an SAE and would be considered a non serious event, whereas a Grade 2 seizure resulting in a hospital admission would be considered an SAE.

10.2.2 Assessment of relationship

Relationship of adverse events with study drugs will be assessed by investigators based on toxicity profile described for each study drug (see investigator brochure).

10.3 Recording of adverse events and serious adverse events

Adverse events will be recorded in the eCRF using a recognized medical term or diagnosis that accurately reflects the event. Adverse events will be assessed by the investigator for severity, relationship to the investigational product, possible etiologies, and whether the event meets criteria of an SAE and therefore requires immediate notification and not later than 24 h, to the Sponsor. The following variables will be collected for each AE:

- AE (verbatim)
- The date when the AE started and stopped
- Changes in NCI CTCAE grade and the maximum CTC grade attained
- Whether the AE is serious or not
- Investigator causality rating against durvalumab or tremelimumab (yes or no)
- Action taken with regard to durvalumab + tremelimumab
- Outcome

In addition, the following variables will be collected for SAEs as applicable:

- Date AE met criteria for serious AE
- Date Investigator became aware of serious AE
- AE is serious due to criteria
- Date of hospitalization

- Date of discharge
- Probable cause of death
- Date of death
- Autopsy performed
- Description of AE
- Causality assessment in relation to Study procedure(s)

Events, which are unequivocally due to disease progression, should not be reported as an AE during the study.

10.3.1 Study recording period and follow-up for adverse events and serious adverse events

Adverse events and serious adverse events will be recorded from time of signature of informed consent, throughout the treatment period and including the follow-up period (90 days after the last dose of durvalumab + tremelimumab).

During the course of the study all AEs and SAEs should be proactively followed up for each subject. Every effort should be made to obtain a resolution for all events, even if the events continue after discontinuation/study completion.

If a subject discontinues from treatment for reasons other than disease progression, and therefore continues to have tumor assessments, drug or procedure-related SAEs must be captured until the patient is considered to have confirmed PD and will have no further tumor assessments.

The investigator is responsible for following all SAEs until resolution, until the subject returns to baseline status, or until the condition has stabilized with the expectation that it will remain chronic, even if this extends beyond study participation.

Follow-up of unresolved adverse events

Any AEs that are unresolved at the subject's last visit in the study are followed up by the investigator for as long as medically indicated, but without further recording in the eCRF. After 90 days, only subjects with ongoing investigational product-related SAEs will continue to be followed for safety.

The sponsor and AstraZeneca/MedImmune retain the right to request additional information for any subject with ongoing AE(s)/SAE(s) at the end of the study, if judged necessary.

Post study events

After the subject has been permanently withdrawn from the study, there is no obligation for the investigator to actively report information on new AE or SAEs occurring in former study subjects after the 90-day safety follow-up period for patients treated with durvalumab + tremelimumab. However, if an investigator learns of any SAEs, including death, at any time after the subject has been permanently withdrawn from study, and he/she considers there is a reasonable possibility that the event is related to study treatment,the investigator should notify the study sponsor, who will

inform AstraZeneca/MedImmune Drug Safety.

10.3.2 Reporting of serious adverse events

All SAEs will be reported, whether or not considered causally related to the investigational product, or to the study procedure(s).. Sponsor is responsible for informing the Health Authorities of the SAE as per local requirements.

After the initial notification of an AE or SAE, the investigator is required to follow up each case and obtain more data on the patient's state. All the AEs and SAEs documented in previous visits must be reviewed in subsequent visits. All AEs and SAEs must be monitored until their resolution, stabilization, explanation of the event by another cause, or until the follow-up period ends. This is applicable to all the patients, including those who withdraw early.

The investigator will include in the follow-up any additional research that may clarify the nature and/or causality of the AE or SAE. This might include additional laboratory tests or studies, histopathologic examinations, or the consultation of other healthcare professionals. Any co-investigator can ask the investigator to carry out additional evaluations to definitively clarify the nature and/or the causality of AEs or SAEs. If the patient dies while participating in the study or during a follow-up period agreed upon mutually, the investigator will provide a copy of any postmortem finding requested, including histopathology.

The sponsor is responsible for notifying all serious or unexpected AEs potentially related to the treatments being investigated that occur during the study to the AEMPS, competent bodies of the autonomous communities where the clinical trial is being conducted within the time frame established by Spanish law and will concurrently forward all such reports to AstraZeneca. A copy of the SAE report must be sent by e-mail to AstraZeneca at the time the event is reported to the Health Authorities **(AEMailboxClinicalTrialTCS@astrazeneca.com)**. It is the responsibility of the sponsor to compile all necessary information and ensure that the Health Authorities receives a report according to the reporting requirement timelines and to ensure that these reports are also submitted to AstraZeneca at the same time.

SAE report and accompanying cover page will be sent by way of email to

[redacted]

If a non-serious AE becomes serious, this and other relevant follow-up information must also be provided to AstraZeneca and the Health Authorities.

Serious adverse events that do not require expedited reporting to the Health Authorities still need to be reported to AstraZeneca preferably using the MedDRA coding language for serious adverse events. This information should be reported on a monthly basis and under no circumstance less frequently than quarterly.

10.3.2.1 Reporting of deaths

All deaths that occur during the study, or within the protocol-defined 90-day post-last dose of

durvalumab + tremelimumab safety follow-up period must be reported as follows:

- Death that is clearly the result of disease progression should be documented but should not be reported as an SAE.
- Where death is not due (or not clearly due) to progression of the disease under study, the AE causing the death must be reported to as a SAE within **24 hours** (see Section 10.3.2 for further details). The report should contain a comment regarding the co-involvement of progression of disease, if appropriate, and should assign main and contributory causes of death.
- Deaths with an unknown cause should always be reported as a SAE.

Deaths that occur following the protocol-defined 90-day post-last-dose of durvalumab safety follow-up period will be documented as events for survival analysis, but will not be reported as an SAE.

10.3.3 Other events requiring reporting

10.3.3.1 Overdose

An overdose is defined as a subject receiving a dose of durvalumab + tremelimumab in excess of that specified in the Investigator's Brochure, unless otherwise specified in this protocol.
Any overdose of a study subject with durvalumab + tremelimumab, with or without associated AEs/SAEs, is required to be reported within 24 hours of knowledge of the event to the sponsor ,who will send a copy to AstraZeneca/MedImmune Patient Safety, or designed using the designated Safety e-mailbox (see Section 10.3.2 for contact information). If the overdose results in an AE, the AE must also be recorded as an AE (see Section 10.3). Overdose does not automatically make an AE serious, but if the consequences of the overdose are serious, for example death or hospitalization, the event is serious and must be recorded and reported as an SAE (see Section 10.3 and Section 10.3.2). There is currently no specific treatment in the event of an overdose of durvalumab or tremelimumab.

The investigator will use clinical judgment to treat any overdose.

10.3.3.2 Hepatic function abnormality

Hepatic function abnormality (as defined in Section 10.1.3.) in a study subject, with or without associated clinical manifestations, is required to be reported as "hepatic function abnormal" ***within 24 hours of knowledge of the event*** to the sponsor ,who will send a copy to AstraZeneca/MedImmune Patient Safety, using the designated Safety e-mailbox (see Section 10.3.2 for contact information), unless a definitive underlying diagnosis for the abnormality (e.g., cholelithiasis or bile duct obstruction) that is unrelated to investigational product has been confirmed.

- If the definitive underlying diagnosis for the abnormality has been established and is unrelated to investigational product, the decision to continue dosing of the study subject will be based on the clinical judgment of the investigator.
- If no definitive underlying diagnosis for the abnormality is established, dosing of the study subject must be interrupted immediately. Follow-up investigations and inquiries must be initiated by the investigational site without delay.

Grade 1-2 events should be considered AEs, not SAEs. However, Grade 1-2 events should be reported as SAEs if requires hospitalization or if they are medically important events based on the investigator judgement.

Grade 3 events, should be reported as SAE if requires hospitalization or if they are medically important events based on the investigator judgement.

All grade 4 events should be reported as SAEs.

Cases where a patient shows elevations in liver biochemistry may require further evaluation and occurrences of AST or ALT ≥3 × ULN together with total bilirubin ≥2 × ULN may need to be reported as SAEs.
Each reported event of hepatic function abnormality will be followed by the investigator and evaluated by the sponsor.

10.3.3.3 Pregnancy

a) Maternal exposure

If a patient becomes pregnant during the course of the study, the IPs should be discontinued immediately.
Pregnancy itself is not regarded as an AE unless there is a suspicion that the IP under study may have interfered with the effectiveness of a contraceptive medication. Congenital abnormalities or birth defects and spontaneous miscarriages should be reported and handled as SAEs. Elective abortions without complications should not be handled as Aes. The outcome of all pregnancies (spontaneous miscarriage, elective termination, ectopic pregnancy, normal birth, or congenital abnormality) should be followed up and documented even if the patient was discontinued from the study.
If any pregnancy occurs in the course of the study, then the Investigator or other site personnel should inform the appropriate sponsor representatives within 1 day, ie, immediately, but **no later than 24 hours** of when he or she becomes aware of it.
The designated sponsor representative will work with the Investigator to ensure that all relevant information is provided to the sponsor, who will inform AstraZeneca Patient Safety data entry site.
The same timelines apply when outcome information is available.

b) Paternal exposure

Male patients should refrain from fathering a child or donating sperm during the study and for 180 days after the last dose of durvalumab + tremelimumab combination therapy or 90 days after the last dose of durvalumab monotherapy, whichever is the longer time period.
Pregnancy of the patient’s partner is not considered to be an AE. However, the outcome of all pregnancies (spontaneous miscarriage, elective termination, ectopic pregnancy, normal birth, or congenital abnormality) occurring from the date of the first dose until 90 days after the last dose should, if possible, be followed up and documented.
Where a report of pregnancy is received, prior to obtaining information about the pregnancy, the Investigator must obtain the consent of the patient’s partner. Therefore, the local study team should adopt the generic ICF template in line with local procedures and submit it to the relevant Ethics Committees (ECs) prior to use.

11. STATISTICAL METHODS AND SAMPLE SIZE DETERMINATION

11.1. Description of analysis sets

The statistical analyses described in this section will be performed as further outlined in the Statistical Analysis Plan, which will be included in the clinical study report for this protocol.

Analysis Sets

The analysis sets will be defined as follows:

- **Full Analysis Set** will include all allocated subjects. This will be primary analysis set for the efficacy endpoints.
- **Per Protocol Analysis Set** will include those subjects who were allocated and received at least one dose of the assigned study drug and had no major protocol deviations. The subjects will complete both baseline and at least one post-baseline tumor assessments (week 12).
- S**afety Analysis Set** will include all subjects who were allocated and received at least one dose of the study drug and had at least one post-baseline safety evaluation (week 12). This will be the analysis set for all safety evaluations.
- **Pharmacodynamic Analysis Set**: All the subjects who have received at least one dose of study drug and have evaluable pharmacodynamic data.

Demographic and other baseline characteristics

Demographic and other baseline characteristics will be summarized and listed. For continuous demographic/baseline variables including age, weight, and vital signs, results will be summarized and presented as n, number of not available data (NA), mean, standard deviation, median, and minimum and maximum values. For categorical variables such as race/ethnicity, the number and percentage of subjects will be used.

Prior and concomitant medications

Concomitant medications will be assigned an 11-digit code using the World Health Organization Drug Dictionary (WHO DD) drug codes. Concomitant medications will be further coded to the appropriate Anatomical-Therapeutic-Chemical (ATC) code indicating therapeutic classification. Prior and concomitant medications will be summarized and listed by drug and drug class.

Efficacy Analyses

All efficacy analyses will be based primarily on the Full Analysis Set and secondarily on the Per Protocol Analysis Set.

Data cut-off for the primary study analysis will happen following after the last patient included in the study has performed the first tumor evaluation (week 12 after first dose of study drug).The following tumor evaluations will take place every 12 weeks until documentation of disease progression or start of another anticancer therapy.

Analysis of primary efficacy variable

The analysis of clinical benefit rate and overall survival rate will be performed independently for each study cohort when the last patient included in the corresponding cohort of the study will arrived to 9 months after inclusion in the study or have progressed. The primary objective of the study will be based on the investigator assessment.

11.2. Methods of statistical analyses

Strategic decisions about future development of immune therapy in neuroendocrine tumors will be available after the results of this trial.

Summary tables (descriptive statistics and frequency tables) will be provided for all baseline variables, efficacy variables, and safety variables, as appropriate. Continuous variables will be summarized with descriptive statistics (mean, standard deviation, range, and median). Ninety-five (95) percent confidence intervals (95% CI) may also be presented, as appropriate. Frequency counts and percentage of subjects within each category will be provided for categorical data.

The primary efficacy analysis will be performed using the binomial test procedure. Missing data will be treated using statistical multiple random imputation.

Concordance between investigator assessment and independent central radiology review will be analyzed using Cohen's Kappa. Although no significant differences are expected, a sensitivity analysis for the primary efficacy analysis will be performed using as response variable independent central radiology assessment.

Secondary endpoints will be summarized with descriptive statistics. Continuous variables will be summarized with n, mean, standard deviation, and range, frequency counts and percentage of subjects within each category will be provided for categorical data. Multivariate regression models will be used to study relations between explanatory variables and primary endpoint. Survival analysis will be performed to analyze PFS, Kaplan-Meier curves will be presented and possible comparisons will be tested using the log-rank test or the Cox proportional hazard model for multivariate analysis, hazard ratios (HR) and their 95% confidence interval (CI95%) will be provided. Patients with lost of follow-up or treatment discontinuation will be included in the final analysis of primary endpoint if they have at least one tumor evaluation and considered as censored data for survival endpoints.

R software version 3.2.1 will be used for all analysis.

Safety Analyses

Safety analyses will be based on the Safety Analysis Set. All safety analyses will be summarized separately by cohort. Adverse events and serious adverse events, laboratory test results, physical examination findings and vital signs, and their changes from baseline will be summarized using descriptive statistics. Abnormal values will be flagged.

11.3. Determination of sample size

Sample size has been calculated using one-sample Superiority test (function One Sample Proportion. NIS of the Trial Size package of R software). According to previous knowledge, it is estimated that the reference value for the likelihood to be progression-free at 9 months is 30% and

we expect an increase of 20% with a superiority margin of 10%. With a unilateral alpha level of 5% and 80% power, we estimate to include 28 patients per group, with an expected lost to follow-up rate of 10%, a final sample size in each 1, 2 and 3 cohort will include 31 patients.

For cohort 4, and according to previous knowledge, it is estimated that the reference proportion of patients being alive at 9 months is 13% and we expect an increase of 10% with a superiority margin of 5%. With a unilateral alpha level of 5% and 80% power, we estimate to include 30 patients per group, with an expected lost to follow-up rate of 10%, a final sample size will include 33 patients. Summarizing, total sample size will include 126 patients: 31 patients for cohorts 1 to 3, and 33 patients for cohort 4.

12. ETHICAL AND REGULATORY REQUIREMENTS

12.1. Ethical conduct of the study

The study will be performed in accordance with ethical principles that have their origin in the Declaration of Helsinki and are consistent with ICH/Good Clinical Practice, and applicable regulatory requirements including those related to Subject data protection.

12.2. Ethics and regulatory review

The protocol, ICF, and appropriate related documents must be reviewed and approved by an EC constituted and functioning in accordance with ICH E6, Section 3, and any local regulations,. Any protocol amendment and/or revision to the ICF will be resubmitted to the EC for review and approval, except for changes involving only logistical or administrative aspects of the study (e.g., change in CRA[s] or change of telephone number[s]). Documentation of EC compliance with ICH and any local regulations regarding constitution and review conduct will be provided to the sponsor.

A signed letter of study approval from the EC Chairman must be obtained prior to study start and the release of any study drug to the site by the sponsor or its designee. If the EC decides to suspend or terminate the study, the sponsor will immediately send the notice of study suspension or termination by the EC to the investigators. Study progress is to be reported to the EC annually (or as required) by the sponsor. If the investigator is required to report to the EC, he/she will forward a copy to the sponsor at the time of each periodic report. The sponsor will submit periodic reports and inform the EC of any reportable adverse events according to local legislation. Upon completion of the study, the investigator will provide the EC with a brief report of the outcome of the study, if required.

12.3. Informed consent

As part of administering the informed consent document, the investigator must explain to each subject (or guardian/legally authorized representative) the nature of the study, its purpose, the procedures involved, the expected duration, the potential risks and benefits involved, and any potential discomfort. Each subject must be informed that participation in the study is voluntary and that he/she may withdraw from the study at any time and that withdrawal of consent will not affect his/her subsequent medical treatment or relationship with the treating physician. This informed consent should be given by means of a standard written statement, written in non-technical language. The subject should understand the statement before signing and dating it and will be

given a copy of the signed document. If a subject is unable to read or if a legally acceptable representative is unable to read, an impartial witness should be present during the entire informed consent discussion. After the written informed consent form and any other written information to be provided to subjects is read and explained to the subject or the subject's legally acceptable representative, and after the subject or the subject's legally acceptable representative has orally consented to the subject's participation in the trial and, if capable of doing so, has signed and personally dated the ICF, the witness should sign and personally date the consent form. The subject will be asked to sign an informed consent at the Screening Visit prior to any study-specific procedures being performed. No subject can enter the study before his/her informed consent has been obtained. The form must be signed and dated by the appropriate parties. The original, signed ICF for each subject will be verified by the sponsor and kept on file, according to local procedure, at the study center.

The subject or the subject's legally authorized representative should be informed in a timely manner if new information becomes available that may be relevant to the subject's willingness to continue participation in the trial. The communication of this information should be documented.

12.4. Changes to the protocol and informed consent form

There are to be no changes to the protocol without written approval from the sponsor. Protocols will be followed as written. Any change to the protocol requires a written protocol amendment or administrative change that must be approved by the sponsor before implementation. Amendments affecting the safety of subjects, the scope of the investigation, or the scientific quality of the study require submission to Health Authorities as well as additional approval by the EC. These requirements should in no way prevent any immediate action from being taken by the investigator, or by the sponsor, in the interest of preserving the safety of all subjects included in the study. If an immediate change to the protocol is felt by the investigator to be necessary for safety reasons, the sponsor's appropriate study team member must be notified promptly and the EC for the site must be informed immediately. A protocol change intended to eliminate an immediate hazard may be implemented immediately, provided that the Health Authorities and EC are subsequently notified by protocol amendment.

Changes affecting only administrative aspects of the study do not require formal protocol amendments or EC approval, but the EC (if regionally required, the heads of the medical institutions) must be kept informed of such changes. In these cases, the sponsor will send a letter to the EC (if regionally required, the heads of the medical institutions) detailing such changes.

12.5. Audits and inspections

In addition to routine monitoring procedures, the sponsor's Clinical Quality Assurance department conducts audits of clinical research activities in accordance with the sponsor's Standard Operating Procedures (SOPs) to evaluate compliance with the principles of ICH GCP and all applicable local regulations. A government regulatory authority may also wish to conduct an inspection (during the study or after its completion). If an inspection is requested by a regulatory authority, the investigator must inform the sponsor immediately that this request has been made.

12.6. Confidentiality

The contents of this protocol and any amendments and results obtained during the course of this

study will be kept confidential by the investigator, the investigator's staff, and the EC and will not be disclosed in whole or in part to others or used for any purpose other than reviewing or performing the study without the written consent of the sponsor. No data collected as part of this study will be utilized in any written work, including publications, without the written consent of the sponsor. These obligations of confidentiality and non-use shall in no way diminish such obligations as set forth in the Confidentiality Agreement between the sponsor and the investigator.

All persons assisting in the performance of this study must be bound by the obligations of confidentiality and non-use set forth in the Confidentiality Agreement between the investigator and sponsor (provided by the sponsor).

All laboratory specimens, evaluation forms, reports, and other records will be identified in a manner designed to maintain subject confidentiality. Each patient will be given a Unique Patient Number (UPN) for this study, provided by the Sponsor. The investigator will custody a log matching the UPN with personal data of patients. All data will be recorded i the appropriate CRFs using this identification number. This number will be provided to the central laboratory to ensure traceability of study samples. Each Investigator will ensure that all site personnel involved will respect the confidentiality of any information about trial subjects. Management of personal data from subjects participating in the trial, particularly as regards consent, will comply with European Directive on Data Privacy (95/46/EC) and the Spanish Organic Law 15/1999 of 13th December on the protection of personal data.

At each site, all records will be kept in a secure storage area with limited access. Clinical information will not be released without the written permission of the subject. Subject identity is confidential and may only be known by the Investigator, trial personnel, appointed auditors and monitors, and Health Authorities.

Each Investigator and all employees and coworkers involved with this trial may not disclose or use for any purpose other than performance of the trial, any data, record, or other unpublished, confidential information disclosed to those individuals for the purpose of the trial. Prior written agreement from the sponsor or its designee must be collected for the disclosure of any said confidential information to other parties.

12.7. Insurance

The sponsor has contracted an insurance policy to cover the responsibilities of the investigator and other parties participating in the study, according to the applicable Spanish legislation.

Insurance company: **CHUBB Insurance Company of Europe SE.**

Policy Number: **9950243799502437**

12.8. Publications

The sponsor commits to responsible publication of both the positive and negative results from its clinical trials as required by all governing regulatory and health authorities.

Investigators will not publish the global study results (all sites) unless the sponsor has not done so in a suitable time period after the clinical study report (CSR) has been available. Should the Investigator(s) independently seek to publish results of this study which occur at their study site(s), they must inform the study sponsor of any/all drafts (including, but not limited to papers,

manuscripts or abstracts) at least 60 days before submission to the congress, meeting or journal. The sponsor and Investigator(s) will agree with all aspects related to any proposed publications with regards to the following: 1) any proposed publications will be drafted in agreement with international recommendations, such as those from the International Committee of Medical Journal Editors (ICMJE) and all elements of the Consort Statement (2010), to maintain integrity of the trial results in all communications; 2) any proposed publications will state the Clinical Research Ethics Committees which approved the trial and the funding sources of the trial; 3) any proposed publications will occur before disclosure of results to lay people; 4) any proposed publications will not report premature or partial data prior to completion of the analysis of the overall results of the trial.

13. STUDY MANAGEMENT

13.1. Training of study site personnel

The principal investigator will maintain a record of all center staff involved in the clinical trial (doctors, nurses and other staff involved) ensuring that they receive appropriate training to perform the study, and that any new information of relevance to the study will be transmitted to them.

Researchers will be instructed about the procedures of the trial in the investigator meeting and/or initiation visits made by monitors at each participating center prior to the study start.

13.2. Monitoring of the study

The sponsor's or representative (e.g., CRO's CRA) will maintain contact with the investigator and designated staff by telephone, and/or letter, and/or email between study visits. Monitoring visits to each investigational site will be conducted by the assigned CRA as described in the monitoring plan. The investigator (if regionally required, the heads of the medical institutions) will allow the CRA to inspect the clinical, laboratory, and pharmacy facilities to assure compliance with Good Clinical Practices and local regulatory requirements. The eCRFs and subject's corresponding original medical records (source documents) are to be fully available for review by the sponsor's representatives at regular intervals. These reviews verify adherence to the study protocol and data accuracy in accordance with federal regulations. All records at the investigational site, including source documents, are subject to inspection by the regulatory authorities and to review by the Ethical Committee.

13.3. Source data

In accordance with ICH E6, Section 6.10, source documents include but are not limited to the following:

- Clinic, office, hospital charts;
- Copies or transcribed healthcare provider notes which have been certified for accuracy after production;
- Recorded data from automated instruments such as x-rays, and other imaging reports: e.g., sonograms, CT scans, MRIs, nuclear medicine scans, ECGs, rhythm strips, electroencephalograms (EEGs), polysomnography, and pulmonary function tests

(regardless of how these images are stored, including microfiche and photographic negatives);

- Records of telephone contacts;
- Drug distribution and accountability logs maintained in pharmacies or by research personnel;
- Laboratory results and other laboratory test outputs: e.g., urine pregnancy test result documentation;
- Correspondence regarding a study subject's treatment between physicians or memoranda sent to the EC;

13.4. Retention of Records

The circumstances of completion or termination of the study notwithstanding, the investigator (if regionally required, the heads of the medical institutions) has the responsibility to retain all study documents, including but not limited to the protocol, the Investigator's Brochure, regulatory agency registration documents, ICFs, and EC correspondence. The investigational site should retain study documents until at least 25 years after the finalization of the study.

It is requested that at the completion of the required retention period or, should the investigator retire or relocate, the investigator contact the sponsor, allowing the sponsor the option of permanently retaining the study records.

13.5. Quality assurance

This study will be organized, performed, and reported in compliance with the protocol, standard operating practices (SOPs), working practice documents, and applicable regulations and guidelines. Site visit audits will be made periodically by the sponsor's or CRO's qualified compliance auditing team, which is an independent function from the study conduct team.

13.6. Study timetable and end of study

End of study is defined as Last Subject Last Visit.

- Recruitment period will be: 24 months
- Research Agreement executed: Nov 2016
- Projected IRB/IEC approval: Feb 2017
- First Subject In: Apr 2017
- 50% Enrollment: Apr 2018
- Last Subject In (100% enrollment): Apr 2019
- Last Subject Last Visit: Apr 2020
- Clinical Study Report Aug 2020

14. DATA MANAGEMENT

All software applications used in the collection and validation of data must be properly validated following standard computer system validation and must be compliant to all regulatory requirements.

The Data Management Plan (DMP) defines and documents the procedures necessary to ensure data quality. These activities must be followed to ensure data are properly entered, validated, coded, integrated, reconciled and reviewed.

Data required by the protocol are collected on an electronic Case Report Form (eCRF) and entered into a validated data management system which is compliant to all regulatory requirements. As defined by ICH Guidelines, the Case Report Form (CRF) is a printed, optical, or electronic document designed to record all of the protocol required information to be reported to the sponsor on each trial subject'. In this study, CRF should refer to electronic data collection form. Data collected on the CRF must follow the instructions described in the CRF Completion Guidelines. The Investigator has ultimate responsibility for the collection and reporting of all clinical, safety and laboratory data entered on the CRF.

Any corrections to entries made on the CRF must be documented in a valid audit trail where the corrections must be dated, initialed, the reason for change stated, and original data not obscured. Only data required by the protocol for the purposes of the study should be collected.

The primary objective of the study will be clinical benefit rate at 9 months and survival rate at 9 months by investigator and reviewed by central radiology review. Images of tumor evaluations will be anonymized correctly identified with trial number patient (code dissociated) and send to Vall d'Hebron University Hospital for revision by an independent experienced radiologist.

14.1. Study governance and oversight

The safety of all AstraZeneca products is closely monitored on an ongoing basis by AstraZeneca representatives in consultation with Patient Safety. Issues identified will be addressed; for instance, this could involve amendments to the study protocol and letters to Investigators.

15. INVESTIGATIONAL PRODUCT AND OTHER TREATMENTS

15.1. Identity of investigational product(s)

Table 7. List of investigational products for this study

Investigational product	Dosage form and strength	Manufacturer
Durvalumab	1500 mg, solution, IV	MedImmune
Tremelimumab	75 mg, solution, IV	MedImmune

16. LIST OF REFERENCES

Andorsky et al 2011

Andorsky DJ, Yamada RE, Said J, Pinkus GS, Betting DJ, and Timmerman JM. Programmed death ligand 1 is expressed by non-hodgkin lymphomas and inhibits the activity of tumor-associated T cells. Clin Cancer Res. 2011. 17(13):4232-4244

Antonia et al 2014b

Antonia S, Ou SI, Khleif SN, Brahmer J, Blake-Haskins A, Robbins PB, et al. Clinical activity and safety of MEDI4736, an anti-programmed cell death ligand-1 (PD-L1) antibody in patients with NSCLC. Poster presented at the European Society for Medical Oncology (ESMO) Meeting; 2014 Sep 26-30; Madrid, Spain.

Antonia et al 2014a

Antonia S, Goldberg S, Balmanoukian A, Narwal R, Robbins P, D'Angelo G, et al. A Phase 1 open-label study to evaluate the safety and tolerability of MEDI4736, an anti-programmed cell death-ligand 1 (PD-L1) antibody, in combination with tremelimumab in patients with advanced non-small cell lung cancer (NSCLC). Poster presented at European Society of Medical Oncology (ESMO) Meeting; 2014 Sep 26-30; Madrid, Spain.

Antonia et al 2015

Antonia et al. Phase ib study of medi4736 in combination with treme in patients with advanced NSCLC. Chicago, Il ASCO May30-June4,ASCO 2015, poster #3014.

Berry et al 1991

Berry G, Kitchin RM, Mock PA. A comparison of 2 simple hazard ratio estimators based on the log rank test. Stat Med 1991;10(5):749-55.

Bograd et al 2011

Bograd AJ, Suzuki K, Vertes E, Colovos C, Morales EA, Sadelain M, et al. Immune responses and immunotherapeutic interventions in malignant pleural mesothelioma. Cancer Immunol Immunother. 2011;60:1509-27.

Bonomi 2010

Bonomi PD. Implications of key trials in advanced non small cell lung cancer. Cancer 2010;116(5):1155-64.

Brahmer et al 2012

Brahmer JR, Tykodi SS, Chow LQM, Hwu WJ, Topalian SL, Hwu P, et al. Safety and activity of anti-PD-L1 antibody in patients with advanced cancer. N Engl J Med 2012;366(26):2455-65.

Brahmer et al 2014

Brahmer JR, Horn L, Gandhi L, Spigel DR, Antonia SJ, Rizvi NA et al. Nivolumab (anti-PD-1, BMS-936558, ONO-4538) in patients (pts) with advanced non-small-cell lung cancer (NSCLC):

Survival and clinical activity by subgroup analysis[ASCO abstract 8112]. J Clin Oncol 2014;32(15)(Suppl).

Brusa et al 2013

Brusa D, Serra S, Coscia M, Rossi D, D'Arena G, Laurenti L, et al. The PD-1/PD-L1 axis contributes to T-cell dysfunction in chronic lymphocytic leukemia. Haematologica. 2013 Jun; 98(6):953-963.

Burt et al 2011

Burt BM, Rodig SJ, Tilleman TR, Elbardissi AW, Bueno R, Sugarbaker DJ. Circulating and tumor-infiltrating myeloid cells predict survival in human pleural mesothelioma. Cancer. 2011;117(22):5234-44.

Ciuleanu et al 2009

Ciuleanu T, Brodowicz T, Zielinski C, Kim JH, Krzakowski M, Laack E, et al. Maintenance pemetrexed plus best supportive care versus placebo plus best supportive care for non-small-cell lung cancer: a randomized, double-blind, phase 3 study. Lancet 2009;374(9699):1432-40.

Collett 2003

Collett D. Modelling survival data in medical research: 2nd ed. Chapman and Hall/CRC; 2003.

D'Addario et al 2010

D'Addario G, Fruh M, Reck M, Baumann P, Klepetko W, Felip E, et al. Metastatic non-small-cell lung cancer: ESMO clinical practice guidelines for diagnosis, treatment and follow-up. Ann Oncol 2010;21(Suppl 5):116-9.

Drake et al 2013

Drake CG, McDermott DF, Sznol M, Choueiri TK, Kluger HM, Powderly JD et al. Survival, safety, and response duration results of nivolumab (Anti-PD-1; BMS-936558; ONO-4538) in a phase I trial in patients with previously treated metastatic renal cell carcinoma (mRCC): Long-term patient follow-up[abstract 4514]. J Clin Oncol 2013;31(Suppl).

Dunn et al 2004

Dunn GP, Old LJ, Schreiber RD. The three Es of cancer immunoediting. Annu Rev Immunol 2004;22:329-60.

Ellis et al 2008

Ellis S, Carroll KJ, Pemberton K. Analysis of duration of response in oncology trials. Contemp Clin Trials 2008;29(4):456-65.

Forde et al 2014

Forde PM, Kelly RJ, Brahmer JR. New strategies in lung cancer: translating immunotherapy into clinical practice. Clin Cancer Res 2014;20(5):1067-73.

Gail and Simon 1985

Gail M, Simon R. Tests for qualitative interactions between treatment effects and patient subsets. Biometrics 1985;41(2):361–72.

GLOBOCAN 2012

GLOBOCAN. Lung cancer, estimated incidence, mortality and prevalence worldwide in 2012. International Agency for Research on Cancer, World Health Organization; Lyon, 2012.Available at http://globocan.iarc.fr/Pages/fact_sheets_cancer.aspx. Accessed on 16 April 2015.

Gong et al 2009

Gong AY, Zhou R, Hu G, Li X, Splinter PL, O'Hara SP, et al. MicroRNA-513 regulates B7-H1 translation and is involved in IFN-gamma-induced B7-H1 expression in cholangiocytes. J Immunol 2009;182(3):1325-33.

Hamanshi et al 2007

Hamanishi J, Mandai M, Iwasaki M, Okazaki T, Tanaka Y, Yamaguchi K, et al. Programmed cell death 1 ligand 1 and tumor-infiltrating CD8+ T lymphocytes are prognostic factors of human ovarian cancer. Proc Nat Acad Sci U S A. 2007;104(9):3360-5.

Herbst et al 2013

Herbst RS, Gordon MS, Fine GD, Sosman JA, Soria JC, Hamid O, et al. A study of MPDL3280A, an engineered PD-L1 antibody in patients with locally advanced or metastatic tumours [abstract 3000]. J Clin Oncol 2013;31(Suppl 15).

Hirano et al 2005

Hirano F, Kaneko K, Tamura H, Dong H, Wang S, Ichikawa M, et al. Blockade of B7-H1 and PD-1 by monoclonal antibodies potentiates cancer therapeutic immunity. Cancer Res 2005;65(3):1089-96.

Hodi et al 2010

Hodi FS, O'Day SJ, McDermott DF, Weber RW, Sosman JA, Haanan JB, et al. Improved survival with ipilimumab in patients with metastatic melanoma [published erratum appears in N Engl J Med 2010;363(13):1290]. N Engl J Med 2010;363(8):711-23.

Hodi et al 2014

Hodi FS, Sznol M, Kluger HM, McDermott DF, Carvajal RD, Lawrence DP et al. Long-term survival of ipilimumab-naive patients (pts) with advanced melanoma (MEL) treated with nivolumab (anti-PD-1, BMS-936558, ONO-4538) in a phase I trial [ASCO abstract 9002]. J Clin Oncol 2014; 32(15)(Suppl).

Howlander et al 2014

Howlander N, Noone AM, Krapcho M, Garshell J, Neyman N, Altekruse SF, et al. SEER cancer statistics review, 1975-2011. Bethesda (MD): National Cancer Institute,. Available from: URL:http://seer.cancer.gov/csr/1975_2011/based on November 2013 SEER data submission

(posted to the SEER web site, April 2014).

IASLC Staging Manual in Thoracic Oncology

Goldstraw P, ed. Staging manual in thoracic oncology. 7th ed. IASLC 2010. (Available on request)

Iwai et al 2002

Iwai Y, Ishida M, Tanaka Y, Okazaki T, Honjo T, Minato N. Involvement of PD-L1 on tumour cells in the escape from host immune system and tumour immunotherapy by PD-L1 blockade.Proc Natl Acad Sci USA 2002;99(19):12293-7.

Keir et al 2008

Keir ME, Butte MJ, Freeman GJ, Sharpe AH. PD-1 and its ligands in tolerance and immunity. Annu Rev Immunol. 2008;26:677-704.

Kirkwood et al 2010

Kirkwood JM, Lorigan P, Hersey P, Hauschild A, Robert C, McDermott D, et al. Phase II trial of tremelimumab (CP-675,206) in patients with advanced refractory or relapsed melanoma. Clin Cancer Res 2010;16(3):1042-8.

Kitano et al 2014

Kitano S, Postow MA, ZieglerCG, Kuk D, Panageas KS, Cortez C, et al. Computational algorithm-driven evaluation of monocytic myeloid-derived suppressor cell frequency for prediction of clinical outcomes. Cancer Immunol Res 2014;2(8):812-21.

Klein et al 2007

Klein JP, Logan B, Harhoff M, Andersen PK. Analyzing survival curves at a fixed point in time. Stat Med 2007;26(24):4505-19.

Korn et al 2008

Korn EL, Liu PY, Lee SJ, Chapman JA, Niedzwiecki D, Suman VJ, et al. Meta-analysis of phase II cooperative group trials in metastatic stage IV melanoma to determine progression-free and overall survival benchmarks for future phase II trials. J Clin Oncol 2008;26(4):527-34.

Krambeck et al 2007

Krambeck AE, Dong H, Thompson RH, Kuntz SM, Lohse CM, Leibovich BC, et al. Survivin and B7-H1 are collaborative predictors of survival and represent potential therapeutic targets for patients with renal cell carcinoma. Clin Cancer Res. 2007;13(6):1749- 56.

Kresowik and Griffith 2009

Kresowik TP, Griffith TS. Bacillus Calmette–Guerin immunotherapy for urothelial carcinoma of the bladder. Immunother. 2009;1(2):281-8.

Lan and DeMets 1983

Lan KKG, DeMets DL. Discrete sequential boundaries for clinical trials.Biometrika

1983;70(3):659-63.

Loos et al 2008

Loos M, Giese NA, Kleeff J, Giese T, Gaida MM, Bergmann F, et al. Clinical significance and regulation of the costimulatory molecule B7-H1 in pancreatic adenocarcinoma. Cancer Lett. 2008;268(1):98-109.

McDermott 2009

McDermott DF. Immunotherapy of metastatic renal cell carcinoma.Cancer. 2009;115(10 suppl):2298–305.

Meyer et al 2014

Meyer C, Cagnon L, Costa-Nunes Cm, Baumgaaertner P, Montandon N, Leyvraz L, et al. Frequencies of circulating MDSC correlate with clinical outcome of melanoma patients treated with ipilimumab. Cancer Immunol Immunother 2014;63(3):247-57.

Mu et al 2011

Mu CY, Huang JA, Chen Y, Chen C, Zhang XG. High expression of PD-L1 in lung cancer may contribute to poor prognosis and tumor cells immune escape through suppressing tumor infiltrating dendritic cells maturation. Med Oncol. 2011 Sep;28(3):682-8.

Nakano et al 2001

Nakano O, Sato M, Naito Y, Suzuki K, Orikasa S, Aizawa M, et al. Proliferative activity of intratumoral CD8+ T-lymphocytes as a prognostic factor in human renal cell carcinoma: clinicopathologic demonstration of antitumor immunity. Cancer Res. 2001;61:5132-6.

Narwal et al 2013

Narwal R, Roskos LK, Robbie GJ (2013) Population Pharmacokinetics of Sifalimumab, an Investigational Anti-Interferon alpha Monoclonal Antibody, in Systemic Lupus Erythematosus. Clin Pharmacokinet 52:1017–1027

Ng et al 2006

Ng CM, Lum BL, Gimenez V, et al. Rationale for fixed dosing of pertuzumab in cancer patients based on population pharmacokinetic analysis. Pharm Res. 2006;23(6):1275–84.

NCCN 2014

National Comprehensive Cancer Network Clinical Practice Guidelines in Oncology (NCCN Guidelines®).Non-Small Cell Lung Cancer. Version 4.2014. www.nccn.org.

Nishino et al 2013

Nishino M, Biobbie-Hurder A, Gargano M, Suda M, Ramaiya NH, Hodi FS. Developing a common language for tumor response to immunotherapy: immune-related response criteria using unidimensional measurements. Clin Cancer Res 2013;19(14):3936-43.

Nomi et al, 2007

Nomi T, Sho M, Akahori T, Hamada K, Kubo A, Kanehiro H, et al. Clinical significance and therapeutic potential of the programmed death-1 ligand/programmed death-1 pathway in human pancreatic adenocarcinoma. Cllin. Cancer Res.2007 Apri 1;13(7):2151-7.

Okudaira et al 2009

Okudaira K, Hokari R, Tsuzuki Y, Okada Y, Komoto S, Watanabe C, et al. Blockade of B7-H1 or B7-DC induces an anti-tumour effect in a mouse pancreatic cancer model. Int J Oncol 2009;35(4):741-9.

Pagès et al, 2010

Pagès F, Galon J, Dieu-Nosjean M.C., Tartour E., Sautès-Fridman C., Fridman W.H.2010. Immune infiltration in human tumors: a prognostic factor that should not be ignored. Oncogene. 29:1093-1102 10.1038/onc.2009.416

Pardoll 2012

Pardoll DM. The blockade of immune checkpoints in cancer immunotherapy.Nat Rev Cancer 2012;12(4):252-64.

Park et al, 2010

Park J, Omiya R, Matsumura Y, Sakoda Y, Kuramasu A, Augustine MM, et al. B7-H1/CD80 interaction is required for the induction and maintenance of peripheral T/cell tolerance. Blood.

Paz-Ares et al 2013

Paz-Ares LG, de Marinis F, Dediu M, Thomas M, Pujol JL, Bidoli P, et al. PARAMOUNT: Final overall survival results of the phase III study of maintenance pemetrexed versus placebo immediately after induction treatment with pemetrexed plus cisplatin for advanced nonsquamous non-small-cell lung cancer. J Clin Oncol 2013;31(23):2895-902.

Peggs et al 2009

Peggs KS, Quezada SA, Allison JP. Cancer immunotherapy: co-stimulatory agonists and coinhibitory antagonists. Clin Exp Immunol 2009;157:9-19.

Pisters and LeChevalier 2005

Pisters KM, LeChevalier T. Adjuvant chemotherapy in completely resected non-small-cell lung cancer. J Clin Oncol 2005;23(14):3270-8.

Raymond et al 2011

Raymond E, Dahan L, Raoul JL, Bang YJ, Borbath I, Lombard-Bohas C et al. Sunitinib malate for the treatment of pancreatic neuroendocrine tumors. N Engl J Med. 2011 Feb 10, 364(6): 501-13. doi:10.1056/NEJMoa1003825.

Reck et al 2014

Reck M, Popat S, Reinmuth N, De Ruysscher D, Kerr KM, Peters S; ESMO Guidelines Working

Group. Metastatic non-small-cell lung cancer (NSCLC): ESMO clinical practice guidelines for diagnosis, treatment and follow-up. Ann Oncol 2014;25(Suppl 3):iii27-39.

Ribas et al 2013

Ribas A, Kefford R, Marshall MA, Punt CJA, Haanen JB, Marmol M, et al. Phase III randomized clinical trial comparing tremelimumab with standard-of-care chemotherapy in patients with advanced melanoma. J Clin Oncol 2013;31:616-22.

Robert et al, 2011

Robert C, Thomas L, Bondarenko I, Steven O'Day S, Weber J, Garbe C. Ipilimumab plus Dacarbazine for Previously Untreated Metastatic Melanoma. N Engl J Med 2011; 364:2517-2526.

Sandler et al 2006

Sandler A, Gray R, Perry MC, Brahmer J, Schiller JH, Dowlati A, et al. Paclitaxel-carboplatin alone or with bevacizumab for non-small-cell lung cancer. N Engl J Med 2006;355(24):2524-50.

Scagliotti et al 2008

Scagliotti GV, Parikh P, von Pawel J, Biesma B, Vansteenkiste J, Manegold C, et al. Phase III study comparing cisplatin plus gemcitabine with cisplatin plus pemetrexed in chemotherapy-naive patients with advanced-stage non-small-cell lung cancer. J Clin Oncol 2008;26(21)3543-51.

Schadendorf et al 2013

Schadendorf D, Hodi FS, Robert C et al. Pooled analysis of long-term survival data from phase II and phase III trials of ipilimumab in metastatic or locally advanced, unresectable melanoma. Presented at: European Cancer Congress 2013 (ECCO-ESMO-ESTRO); September 27-October 1, 2013; Amsterdam, The Netherlands. Abstract 24.

Schiller et al 2002

Schiller JH, Harringdon D, Belani CP, Langer C, Sandler A, Krook J, et al. Comparison of four chemotherapy regimens for advanced non-small-cell lung cancer. N Engl J Med 2002;10(346):92-8.

Schwarzenbach et al 2014

Schwarzenbach H, Nishida N, Calin GA, Pantel K. Clinical evidence of circulating cell-free microRNAs in cancer. Nat Rev Clin Oncol 2014;11(3):145-56.

Selke and Siegmund 1983

Selke T, Siegmund D. Sequential analysis of the proportional hazards model. Biometrika 1983;70:315-26.

Sun and Chen 2010

Sun X, Chen C. Comparison of Finkelstein's Method with the conventional approach for interval-censored data analysis. Stat Biopharm Res 2010;2(1):97-108.

Suzuki et al, 2011

Suzuki K, Kachala SS, Kadota K, Shen R, Mo Q, Beer DG, et al. Prognostic immune markers in non-small cell lung cancer. Clin Cancer res. 2011;17(16):5247-56. doi:10.1158/1078-0432.CCR-10-2805.

Tarhini and Kirkwood 2008

Tarhini AA, Kirkwood JM. Tremelimumab (CP-675,206): a fully human anticytotoxic T lymphocyte-associated antigen 4 monoclonal antibody for treatment of patients with advanced cancers. Expert Opin Biol Ther 2008;8(10):1583-93.

Topalian et al 2012

Topalian SL, Hodi FS, Brahmer JR, Gettinger SN, Smith DC, McDermott DF, et al. Safety, activity, and immune correlates of anti-PD-1 antibody in cancer. N Engl J Med 2012;366(26):2443-54.

Topalian et al 2014

Topalian SL, Sznol M, McDermott DF, Kluger HM, Carvajal RD, Sharfman WH et al. Survival, durable tumor remission, and long-term safety in patients with advanced melanoma receiving nivolumab. J Clin Oncol, 2014 Apr 1; 32(10):1020-30.

Wang et al 2009

Wang DD, Zhang S, Zhao H, et al. Fixed dosing versus body size based dosing of monoclonal antibodies in adult clinical trials. J Clin Pharmacol. 2009;49(9):1012–24.

Wang et al 2013

Wang Z, Han J, Cui Y, Fan K, Zhou X. Circulating microRNA-21 as noninvasive predictive biomarker for response in cancer immunotherapy. Med Hypotheses 2013;81(1):41-3.

Wang et al 2014

Wang E, Kang D, et al. Population pharmacokinetic and pharmacodynamics analysis of tremelimumab in patients with metastatic melanoma. J Clin Pharmacol. 2014 Oct; 54(10):1108-16.

Weber et al 2012

Weber J, Kähler KC, Hauschild A. Management of immune-related adverse events and kinetics of response with ipilimumab. J Clin Oncol 2012;30(21):2691-7.

Whitehead and Whitehead 1991

Whitehead A, Whitehead J. A general parametric approach to the meta-analysis of randomized clinical trials. Stat Med 1991;10(11):1665-77.

Wolchok et al 2009

Wolchok JD, Hoos A, O'Day S, Weber JS, Hammid O, Lebbé, et al. Guidelines for the evaluation of immune therapy activity in solid tumors: immune-related response criteria. Clin Cancer Res. 2009;15:7412-20.

Wolchok et al 2013

Wolchok JD, Kluger H, Callahan MK, Postow MA, Rizvi NA, Lesokhin AM, et al. Nivolumab plus ipilimumab in advanced melanoma. N Engl J Med 2013;369(2):122-33.

Yao et al, 2008

Yao JC, Hassan M, Phan A, et al. One hundred years after "carcinoid": epidemiology of and prognostic factors for neuroendocrine tumors in 35.825 cases in the United States. J Clin Oncol. 2008;26:30663-3072.

Yao et al, 2011

Yao J., Shah M., Ito T., Bohas C., Wolin E., Van Cutsem E., et al (2011). Everolimus for advanced pancreatic neuroendocrine tumors. N Engl J Med 364: 514-523.

Yao et al, 2015

Yao JC, Fazio N, Singh S, et al. Everolimus for the treatment of advanced, non-functional neuroendocrine tumours of the lung or gastrointestinal tract (RADIANT-4): a randomised, placebo-controlled, phase 3 study. Lancet 2015; Advanced online publication 15 December.

Yuan et al 2011

Yuan J, Adamow M, Ginsberg BA, Rasalan TS, Ritter E, Gallardo HF, et al. Integrated NY-ESO-1 antibody and CD8+ T-cell responses correlated with clinical benefit in advanced melanoma patients treated with ipilimumab. Proc Natl Acad Sci USA 2011;108(40):16723-8.

Zhang et al 2008

Zhang C, Wu S, Xue X, Li M, Qin X, Li W, et al. Anti-tumour immunotherapy by blockade of the PD-1/pd-L1 pathway with recombinant human PD-1IgV. Cytotherapy 2008;10(7):711-9. doi: 10.1080.14653240802320237.

Zhang et al 2012

Zhang S, Shi R, Li C, et al. Fixed dosing versus body size-based dosing of therapeutic peptides and proteins in adults. J Clin Pharmacol. 2012;52(1):18–28.

Zou and Chen, 2008

Zou W, chen L. Inhibitory B7-family molecules in the tumour microenvironment. Nat Rev Immunol. 2008;8(6):467-77.

Appendix 1 Dosing Modification and Toxicity Management Guidelines for Immune-Mediated, Infusion-Related, and Non-Immune–Mediated Reactions (MEDI4736 Monotherapy or Combination Therapy With Tremelimumab or Tremelimumab Monotherapy), 1 November 2017 version.

**Dosing Modification and Toxicity Management Guidelines for Immune-Mediated, Infusion-Related, and Non-Immune–Mediated Reactions (MEDI4736 Monotherapy or Combination Therapy With Tremelimumab or Tremelimumab Monotherapy) 1 November 2017 Version**	
**General Considerations**	
**Dose Modifications**	**Toxicity Management**
Drug administration modifications of study drug/study regimen will be made to manage potential immune-related AEs based on severity of treatment-emergent toxicities graded per NCI CTCAE v4.03.<br>In addition to the criteria for permanent discontinuation of study drug/study regimen based on CTC grade/severity (table below), permanently discontinue study drug/study regimen for the following conditions:<br>● Inability to reduce corticosteroid to a dose of ≤10 mg of prednisone per day (or equivalent) **within 12 weeks** after last dose of study drug/study regimen<br>● Recurrence of a previously experienced Grade 3 treatment-related AE following resumption of dosing<br><br>**Grade 1** No dose modification<br>**Grade 2** Hold study drug/study regimen dose until Grade 2 resolution to Grade ≤1.<br>If toxicity worsens, then treat as Grade 3 or Grade 4.<br>Study drug/study regimen can be resumed once event stabilizes to Grade ≤1 after completion of steroid taper.<br>Patients with endocrinopathies who may require prolonged or continued steroid replacement can be retreated with study drug/study regimen on the following conditions:<br>1. The event stabilizes and is controlled.<br>2. The patient is clinically stable as per Investigator or treating physician's clinical judgement.<br>3. Doses of prednisone are at ≤10 mg/day or equivalent.<br>**Grade 3** Depending on the individual toxicity, study drug/study regimen may be permanently discontinued. Please refer to guidelines below.<br>**Grade 4** Permanently discontinue study drug/study regimen.<br>Note: For Grade ≥3 asymptomatic amylase or lipase levels, hold study drug/study regimen, and if complete work up shows no evidence of pancreatitis, study drug/study regimen may be continued or resumed.	It is recommended that management of immune-mediated adverse events (imAEs) follows the guidelines presented in this table:<br>– It is possible that events with an inflammatory or immune mediated mechanism could occur in nearly all organs, some of them not noted specifically in these guidelines.<br>– Whether specific immune-mediated events (and/or laboratory indicators of such events) are noted in these guidelines or not, patients should be thoroughly evaluated to rule out any alternative etiology (e.g., disease progression, concomitant medications, and infections) to a possible immune-mediated event. In the absence of a clear alternative etiology, all such events should be managed as if they were immune related. General recommendations follow.<br>– Symptomatic and topical therapy should be considered for low-grade (Grade 1 or 2, unless otherwise specified) events.<br>– For persistent (>3 to 5 days) low-grade (Grade 2) or severe (Grade ≥3) events, promptly start prednisone 1 to 2 mg/kg/day PO or IV equivalent.<br>– Some events with high likelihood for morbidity and/or mortality – e.g., myo-carditis, or other similar events even if they are not currently noted in the guidelines – should progress rapidly to high dose IV corticosteroids (methylprednisolone at 2 to 4 mg/kg/day) even if the event is Grade 2, and if clinical suspicion is high and/or there has been clinical confirmation. Consider, as necessary, discussing with the study physician, and promptly pursue specialist consultation.<br>– If symptoms recur or worsen during corticosteroid tapering (28 days of taper), increase the corticosteroid dose (prednisone dose [e.g., up to 2 to 4 mg/kg/day PO or IV equivalent]) until stabilization or improvement of symptoms, then resume corticosteroid tapering at a slower rate (>28 days of taper).<br>– More potent immunosuppressives such as TNF inhibitors (e.g., infliximab) (also refer to the individual

Note: Study drug/study regimen should be permanently discontinued in Grade 3 events with high likelihood for morbidity and/or mortality – e.g., myocarditis, or other similar events even if they are not currently noted in the guidelines. Similarly, consider whether study drug/study regimen should be permanently discontinued in Grade 2 events with high likelihood for morbidity and/or mortality – e.g., myocarditis, or other similar events even if they are not currently noted in the guidelines – when they do not rapidly improve to Grade <1 upon treatment with systemic steroids and following full taper<br>Note: There are some exceptions to permanent discontinuation of study drug for Grade 4 events (i.e., hyperthyroidism, hypothyroidism, Type 1 diabetes mellitus).	sections of the imAEs for specific type of immunosuppressive) should be considered for events not responding to systemic steroids. Progression to use of more potent immunosuppressives should proceed more rapidly in events with high likelihood for morbidity and/or mortality – e.g., myocarditis, or other similar events even if they are not currently noted in the guidelines – when these events are not responding to systemic steroids.<br>– With long-term steroid and other immunosuppressive use, consider need for *Pneumocystis jirovecii* pneumonia (PJP, formerly known as *Pneumocystis carinii* pneumonia) prophylaxis, gastrointestinal protection, and glucose monitoring.<br>– Discontinuation of study drug/study regimen is not mandated for Grade 3/Grade 4 inflammatory reactions attributed to local tumor response (e.g., inflammatory reaction at sites of metastatic disease and lymph nodes). Continuation of study drug/study regimen in this situation should be based upon a benefit-risk analysis for that patient.

AE Adverse event; CTC Common Toxicity Criteria; CTCAE Common Terminology Criteria for Adverse Events; imAE immune-mediated adverse event; IV intravenous; NCI National Cancer Institute; PO By mouth.

**Specific Immune-Mediated Reactions**			
**Adverse Events**	**Severity Grade of the Event (NCI CTCAE version 4.03)**	**Dose Modifications**	**Toxicity Management**
**Pneumonitis/Interstitial Lung Disease (ILD)**	**Any Grade**	**General Guidance**	**For Any Grade:**<br>– Monitor patients for signs and symptoms of pneumonitis or ILD (new onset or worsening shortness of breath or cough). Patients should be evaluated with imaging and pulmonary function tests, including other diagnostic procedures as described below.<br>– Initial work-up may include clinical evaluation, monitoring of oxygenation via pulse oximetry (resting and exertion), laboratory work-up, and high- resolution CT scan.
	**Grade 1**<br>(asymptomatic, clinical or diagnostic observations only; intervention not indicated)	No dose modifications required. However, consider holding study drug/study regimen dose as clinically appropriate and during diagnostic work-up for other etiologies.	**For Grade 1 (radiographic changes only):**<br>– Monitor and closely follow up in 2 to 4 days for clinical symptoms, pulse oximetry (resting and exertion), and laboratory work-up and then as clinically indicated.<br>– Consider Pulmonary and Infectious disease consult.

	**Grade 2**<br>(symptomatic; medical intervention indicated; limiting instrumental ADL)	Hold study drug/study regimen dose until Grade 2 resolution to Grade ≤1.<br>● If toxicity worsens, then treat as Grade 3 or Grade 4.<br>● If toxicity improves to Grade ≤1, then the decision to reinitiate study drug/study regimen will be based upon treating physician's clinical judgment and after completion of steroid taper.	**For Grade 2 (mild to moderate new symptoms):**<br>− Monitor symptoms daily and consider hospitalization.<br>− Promptly start systemic steroids (e.g., prednisone 1 to 2 mg/kg/day PO or IV equivalent).<br>− Reimage as clinically indicated.<br>− If no improvement within 3 to 5 days, additional workup should be considered and prompt treatment with IV methylprednisolone 2 to 4 mg/kg/day started<br>− If still no improvement within 3 to 5 days despite IV methylprednisolone at 2 to 4 mg/kg/day, promptly start immunosuppressive therapy such as TNF inhibitors (e.g., infliximab at 5 mg/kg every 2 weeks). Caution: It is important to rule out sepsis and refer to infliximab label for general guidance before using infliximab.<br>− Once the patient is improving, gradually taper steroids over ≥28 days and consider prophylactic antibiotics, antifungals, or anti-PJP treatment (refer to current NCCN guidelines for treatment of cancer-related infections [Category 2B recommendation])[a]<br>− Consider pulmonary and infectious disease consult.<br>− Consider, as necessary, discussing with study physician.

	**Grade 3 or 4**<br>(Grade 3: severe symptoms; limiting self-care ADL; oxygen indicated)<br><br>(Grade 4: life-threatening respiratory compromise; urgent intervention indicated [e.g., tracheostomy or intubation])	Permanently discontinue study drug/study regimen.	**For Grade 3 or 4 (severe or new symptoms, new/worsening hypoxia, life-threatening):**<br>− Promptly initiate empiric IV methylprednisolone 1 to 4 mg/kg/day or equivalent.<br>− Obtain Pulmonary and Infectious disease consult; consider, as necessary, discussing with study physician.<br>− Hospitalize the patient.<br>− Supportive care (e.g., oxygen).<br>− If no improvement within 3 to 5 days, additional workup should be considered and prompt treatment with additional immunosuppressive therapy such as TNF inhibitors (e.g., infliximab at 5 mg/kg every 2 weeks' dose) started. Caution: rule out sepsis and refer to infliximab label for general guidance before using infliximab.<br>− Once the patient is improving, gradually taper steroids over ≥28 days and consider prophylactic antibiotics, antifungals, and, in particular, anti-PJP treatment (refer to current NCCN guidelines for treatment of cancer-related infections [Category 2B recommendation]).[a]
**Diarrhea/Colitis**	**Any Grade**	**General Guidance**	**For Any Grade:**<br>− Monitor for symptoms that may be related to diarrhea/enterocolitis (abdominal pain, cramping, or changes in bowel habits such as increased frequency over baseline or blood in stool) or related to bowel perforation (such as sepsis, peritoneal signs, and ileus).<br>− Patients should be thoroughly evaluated to rule out any alternative etiology (e.g., disease progression, other medications, or infections), including testing for clostridium difficile toxin, etc.<br>− Steroids should be considered in the absence of clear alternative etiology, even for low-grade events, in order to prevent potential progression to higher grade event.<br>− Use analgesics carefully; they can mask symptoms of perforation and peritonitis.
	**Grade 1**<br>(Diarrhea: stool frequency of <4 over baseline per day)<br>(Colitis: asymptomatic; clinical or diagnostic	No dose modifications.	**For Grade 1:**<br>− Monitor closely for worsening symptoms.<br>− Consider symptomatic treatment, including hydration, electrolyte replacement, dietary changes (e.g., American Dietetic Association colitis diet), and loperamide. Use probiotics as per treating physician's clinical judgment.

	observations only)		
	**Grade 2**<br>(Diarrhea: stool frequency of 4 to 6 over baseline per day) (Colitis: abdominal pain; mucus or blood in stool)	Hold study drug/study regimen until resolution to Grade ≤1<br>● If toxicity worsens, then treat as Grade 3 or Grade 4.<br>● If toxicity improves to Grade ≤1, then study drug/study regimen can be resumed after completion of steroid taper.	**For Grade 2:**<br>– Consider symptomatic treatment, including hydration, electrolyte replacement, dietary changes (e.g., American Dietetic Association colitis diet), and loperamide and/or budesonide.<br>– Promptly start prednisone 1 to 2 mg/kg/day PO or IV equivalent.<br>– If event is not responsive within 3 to 5 days or worsens despite prednisone at 1 to 2 mg/kg/day PO or IV equivalent, GI consult should be obtained for consideration of further workup, such as imaging and/or colonoscopy, to confirm colitis and rule out perforation, and prompt treatment with IV methylprednisolone 2 to 4 mg/kg/day started.<br>– If still no improvement within 3 to 5 days despite 2 to 4 mg/kg IV methylprednisolone, promptly start immunosuppressives such as infliximab at 5 mg/kg once every 2 weeks[a]. **Caution:** it is important to rule out bowel perforation and refer to infliximab label for general guidance before using infliximab.<br>– Consider, as necessary, discussing with study physician if no resolution to Grade ≤1 in 3 to 4 days.<br>– Once the patient is improving, gradually taper steroids over ≥28 days and consider prophylactic antibiotics, antifungals, and anti-PJP treatment (refer to current NCCN guidelines for treatment of cancer-related infections [Category 2B recommendation]).[a]
	**Grade 3 or 4**<br>(Grade 3 diarrhea: stool frequency of ≥7 over baseline per day;<br>Grade 4 diarrhea: life threatening consequences)<br>(Grade 3 colitis: severe abdominal pain, change in bowel habits, medi-cal intervention indicated, peritoneal signs;	**Grade 3**<br>Permanently discontinue study drug/study regimen for Grade 3 if toxicity does not improve to Grade ≤1 within 14 days; study drug/study regimen can be resumed after completion of steroid taper.<br><br>**Grade 4**<br>Permanently discontinue study drug/study regimen.	**For Grade 3 or 4:**<br>– Promptly initiate empiric IV methylprednisolone 2 to 4 mg/kg/day or equivalent.<br>– Monitor stool frequency and volume and maintain hydration.<br>– Urgent GI consult and imaging and/or colonoscopy as appropriate.<br>– If still no improvement within 3 to 5 days of IV methylprednisolone 2 to 4 mg/kg/day or equivalent, promptly start further immunosuppressives (e.g., infliximab at 5 mg/kg once every 2 weeks). **Caution**: Ensure GI consult to rule out bowel perforation and refer to infliximab label for general guidance before using infliximab.<br>– Once the patient is improving, gradually taper steroids over ≥28 days and consider

	Grade 4 colitis: life-threatening consequences, urgent intervention indicated)		prophylactic antibiotics, antifungals, and anti-PJP treatment (refer to current NCCN guidelines for treatment of cancer-related infections [Category 2B recommendation]).[a]
**Hepatitis (elevated LFTs)**<br>Infliximab should not be used for management of immune-related hepatitis.	**Any Grade**	**General Guidance**	**For Any Grade:**<br>− Monitor and evaluate liver function test: AST, ALT, ALP, and TB.<br>− Evaluate for alternative etiologies (e.g., viral hepatitis, disease progression, concomitant medications).
	**Grade 1**<br>(AST or ALT >ULN and ≤3.0×ULN and/or TB > ULN and ≤1.5×ULN)	● No dose modifications.<br>● If it worsens, then treat as Grade 2 event.	**For Grade 1:**<br>− Continue LFT monitoring per protocol.
	**Grade 2**<br>(AST or ALT >3.0×ULN and ≤5.0×ULN and/or TB >1.5×ULN and ≤3.0×ULN)	● Hold study drug/study regimen dose until Grade 2 resolution to Grade ≤1.<br>● If toxicity worsens, then treat as Grade 3 or Grade 4.<br>● If toxicity improves to Grade ≤1 or baseline, resume study drug/study regimen after completion of steroid taper.	**For Grade 2:**<br>− Regular and frequent checking of LFTs (e.g., every 1 to 2 days) until elevations of these are improving or resolved.<br>− If no resolution to Grade ≤1 in 1 to 2 days, consider, as necessary, discussing with study physician.<br>− If event is persistent (>3 to 5 days) or worsens, promptly start prednisone 1 to 2 mg/kg/day PO or IV equivalent.<br>− If still no improvement within 3 to 5 days despite 1 to 2 mg/kg/day of prednisone PO or IV equivalent, consider additional work up and start prompt treatment with IV methylprednisolone 2 to 4 mg/kg/day.<br>− If still no improvement within 3 to 5 days despite 2 to 4 mg/kg/day of IV methylprednisolone, promptly start immunosuppressives (i.e., mycophenolate mofetil).[a] Discuss with study physician if mycophenolate mofetil is not available. **Infliximab should NOT be used.**<br>− Once the patient is improving, gradually taper steroids over ≥28 days and consider prophylactic antibiotics, antifungals, and anti-PJP treatment (refer to current NCCN guidelines for treatment of cancer-related infections [Category 2B recommendation]).[a]
	**Grade 3 or 4**<br>(Grade 3: AST or ALT >5.0×ULN and ≤20.0×ULN and/or	**For Grade 3:**<br>For elevations in transaminases ≤8 × ULN, or elevations in bilirubin ≤5 × ULN:<br>● Hold study drug/study regimen dose until	**For Grade 3 or 4:**<br>− Promptly initiate empiric IV methylprednisolone at 1 to 4 mg/kg/day or equivalent.<br>− If still no improvement within 3 to 5 days despite 1 to 4 mg/kg/day

	TB >3.0×ULN and ≤10.0×ULN)<br><br>(Grade 4: AST or ALT >20×ULN and/or TB >10×ULN)	resolution to Grade ≤1 or baseline<br>● Resume study drug/study regimen if elevations downgrade to Grade ≤1 or baseline within 14 days and after completion of steroid taper.<br>● Permanently discontinue study drug/study regimen if the elevations do not downgrade to Grade ≤1 or baseline within 14 days<br>For elevations in transaminases >8 × ULN or elevations in bilirubin >5 × ULN, discontinue study drug/study regimen.<br>Permanently discontinue study drug/study regimen for any case meeting Hy's law criteria (AST and/or ALT >3 × ULN + bilirubin >2 × ULN without initial findings of cholestasis (i.e., elevated alkaline P04) and in the absence of any alternative cause.[b]<br>**For Grade 4:**<br>Permanently discontinue study drug/study regimen.	methylprednisolone IV or equivalent, promptly start treatment with immunosuppressive therapy (i.e., mycophenolate mofetil). Discuss with study physician if mycophenolate is not available. **Infliximab should NOT be used.**<br>− Perform hepatology consult, abdominal workup, and imaging as appropriate.<br>− Once the patient is improving, gradually taper steroids over ≥28 days and consider prophylactic antibiotics, antifungals, and anti-PJP treatment (refer to current NCCN guidelines for treatment of cancer-related infections [Category 2B recommendation]).[a]
**Hepatitis (elevated LFTs)**<br>Infliximab should not be used for management of immune-related hepatitis.<br>See instructions at bottom of shaded area if transaminase rise is not isolated but (at any time) occurs in setting of either **increasing bilirubin or signs of DILI/liver decompensation**	**Any Grade**	**General Guidance**	**For Any Grade:**<br>− Monitor and evaluate liver function test: AST, ALT, ALP, and TB.<br>− Evaluate for alternative etiologies (e.g., viral hepatitis, disease progression, concomitant medications, worsening of liver cirrhosis [e.g., portal vein thrombosis]).<br>− For HBV+ patients: evaluate quantitative HBV viral load, quantitative HBsAg, or HBeAg<br>− For HCV+ patients: evaluate quantitative HCV viral load<br>− Consider consulting hepatologist/Infectious disease specialist regarding change/implementation in/of antiviral medications for any patient with an elevated HBV viral load >2000 IU/ml<br>− Consider consulting hepatologist/Infectious disease specialist regarding change/implementation in/of antiviral HCV medications if HCV viral load increased by ≥2-fold

GETNE Grupo Español de Tumores Neuroendocrinos y Endocrinos

			− For HCV+ with HBcAB+: Evaluate for both HBV and HCV as above.
	**Grade 1**<br>(Isolated AST or ALT >ULN and ≤5.0×ULN, whether normal or elevated at baseline)	• No dose modifications.<br>• If ALT/AST elevations represents significant worsening based on investigator assessment, then treat as Grade 2 event.<br>For all grades, see instructions at bottom of shaded area if transaminase rise is not isolated but (at any time) occurs in setting of either **increasing bilirubin or signs of DILI/liver decompensation.**	
	**Grade 2**<br>(Isolated AST or ALT >5.0×ULN and ≤8.0×ULN, if normal at baseline)<br><br>(Isolated AST or ALT >2.0×baseline and ≤12.5×ULN, if elevated >ULN at baseline)	• Hold study drug/study regimen dose until Grade 2 resolution to Grade ≤1 or baseline.<br>• If toxicity worsens, then treat as Grade 3 or Grade 4.<br>If toxicity improves to Grade ≤1 or baseline, resume study drug/study regimen after completion of steroid taper.	**For Grade 2:**<br>− Regular and frequent checking of LFTs (e.g., every 1 to 3 days) until elevations of these are improving or resolved.<br>− Recommend consult hepatologist; consider abdominal ultrasound, including Doppler assessment of liver perfusion.<br>− Consider, as necessary, discussing with study physician.<br>− If event is persistent (>3 to 5 days) or worsens, and investigator suspects toxicity to be immune-mediated AE, recommend to start prednisone 1 to 2 mg/kg/day PO or IV equivalent.<br>− If still no improvement within 3 to 5 days despite 1 to 2 mg/kg/day of prednisone PO or IV equivalent, consider additional workup and treatment with IV methylprednisolone 2 to 4 mg/kg/day.<br>− If still no improvement within 3 to 5 days despite 2 to 4 mg/kg/day of IV methylprednisolone, consider additional abdominal workup (including liver biopsy) and imaging (i.e., liver ultrasound), and consider starting immunosuppressives (i.e., mycophenolate mofetil).[a] Discuss with study physician if mycophenolate mofetil is not available. **Infliximab should NOT be used.**
	**Grade 3**<br>(Isolated AST or ALT >8.0×ULN and ≤20.0×ULN, if normal at baseline)<br><br>(Isolated AST or ALT >12.5×ULN	• Hold study drug/study regimen dose until resolution to Grade ≤1 or baseline<br>• Resume study drug/study regimen if elevations downgrade to Grade ≤1 or baseline within 14 days and after completion of steroid taper.	**For Grade 3:**<br>− Regular and frequent checking of LFTs (e.g., every 1-2 days) until elevations of these are improving or resolved.<br>− Consult hepatologist (unless investigator is hepatologist); obtain abdominal ultrasound, including Doppler assessment of liver perfusion; and consider liver biopsy.

	and ≤20.0×ULN, if elevated >ULN at baseline)	● Permanently discontinue study drug/study regimen if the elevations do not downgrade to Grade ≤1 or baseline within 14 days<br><br>Permanently discontinue study drug/study regimen for any case meeting Hy's law criteria, in the absence of any alternative cause.[b]	− Consider, as necessary, discussing with study physician.<br>− If investigator suspects toxicity to be immune-mediated, promptly initiate empiric IV methylprednisolone at 1 to 4 mg/kg/day or equivalent.<br>− If no improvement within 3 to 5 days despite 1 to 4 mg/kg/day methylprednisolone IV or equivalent, obtain liver biopsy (if it has not been done already) and promptly start treatment with immunosuppressive therapy (mycophenolate mofetil). Discuss with study physician if mycophenolate is not available. **Infliximab should NOT be used.**<br>− Once the patient is improving, gradually taper steroids over ≥28 days and consider prophylactic antibiotics, antifungals, and anti-PCP treatment (refer to current NCCN guidelines for treatment of cancer-related infections [Category 2B recommendation]).[a]
	**Grade 4**<br><br>(Isolated AST or ALT >20×ULN, whether normal or elevated at baseline)	Permanently discontinue study drug/study regimen.	**For Grade 4:**<br>**Same as above**<br>**(except would recommend obtaining liver biopsy early).**
If transaminase rise is not isolated but (at any time) occurs in setting of either increasing total/direct bilirubin (≥1.5×ULN, if normal at baseline; or 2×baseline, if >ULN at baseline) or signs of DILI/liver decompensation (e.g., fever, elevated INR):<br>- Manage dosing for Grade 1 transaminase rise as instructed for Grade 2 transaminase rise<br>- Manage dosing for Grade 2 transaminase rise as instructed for Grade 3 transaminase rise<br>- Grade 3-4: Permanently discontinue study drug/study regimen			
**Nephritis or renal dysfunction**<br><br>(elevated serum creatinine)	**Any Grade**	**General Guidance**	**For Any Grade:**<br>− Consult with nephrologist.<br>− Monitor for signs and symptoms that may be related to changes in renal function (e.g., routine urinalysis, elevated serum BUN and creatinine, decreased creatinine clearance, electrolyte imbalance, decrease in urine output, or proteinuria).<br>− Patients should be thoroughly evaluated to rule out any alternative etiology (e.g., disease progression or infections).<br>− Steroids should be considered in the absence of clear alternative etiology even for low-grade events (Grade 2), in order to prevent potential progression to higher grade event.
	**Grade 1**<br><br>(Serum creatinine > 1 to 1.5 ×	No dose modifications.	**For Grade 1:**<br>− Monitor serum creatinine weekly and any accompanying symptoms.

	baseline; > ULN to 1.5 × ULN)		● If creatinine returns to baseline, resume its regular monitoring per study protocol.<br>● If creatinine worsens, depending on the severity, treat as Grade 2, 3, or 4.<br>− Consider symptomatic treatment, including hydration, electrolyte replacement, and diuretics.
	**Grade 2**<br>(serum creatinine >1.5 to 3.0 × baseline; >1.5 to 3.0 × ULN)	Hold study drug/study regimen until resolution to Grade ≤1 or baseline.<br>● If toxicity worsens, then treat as Grade 3 or 4.<br>● If toxicity improves to Grade ≤1 or baseline, then resume study drug/study regimen after completion of steroid taper.	**For Grade 2:**<br>− Consider symptomatic treatment, including hydration, electrolyte replacement, and diuretics.<br>− Carefully monitor serum creatinine every 2 to 3 days and as clinically warranted.<br>− Consult nephrologist and consider renal biopsy if clinically indicated.<br>− If event is persistent (>3 to 5 days) or worsens, promptly start prednisone 1 to 2 mg/kg/day PO or IV equivalent.<br>− If event is not responsive within 3 to 5 days or worsens despite prednisone at 1 to 2 mg/kg/day PO or IV equivalent, additional workup should be considered and prompt treatment with IV methylprednisolone at 2 to 4 mg/kg/day started.<br>− Once the patient is improving, gradually taper steroids over ≥28 days and consider prophylactic antibiotics, antifungals, and anti-PJP treatment (refer to current NCCN guidelines for treatment of cancer-related infections [Category 2B recommendation]).[a]<br>− When event returns to baseline, resume study drug/study regimen and routine serum creatinine monitoring per study protocol.
	**Grade 3 or 4**<br>(Grade 3: serum creatinine >3.0 × baseline; >3.0 to 6.0 × ULN;<br><br>Grade 4: serum creatinine >6.0 × ULN)	Permanently discontinue study drug/study regimen.	**For Grade 3 or 4:**<br>− Carefully monitor serum creatinine on daily basis.<br>− Consult nephrologist and consider renal biopsy if clinically indicated.<br>− Promptly start prednisone 1 to 2 mg/kg/day PO or IV equivalent.<br>− If event is not responsive within 3 to 5 days or worsens despite prednisone at 1 to 2 mg/kg/day PO or IV equivalent, additional workup should be considered and prompt treatment with IV methylprednisolone 2 to 4 mg/kg/day started.<br>− Once the patient is improving, gradually taper steroids over ≥28 days and consider prophylactic antibiotics, antifungals, and

			anti-PJP treatment (refer to current NCCN guidelines for treatment of cancer-related infections [Category 2B recommendation]).[a]
**Rash**<br>(excluding bullous skin formations)	**Any Grade**<br>(refer to NCI CTCAE v 4.03 for definition of severity/grade depending on type of skin rash)	**General Guidance**	**For Any Grade:**<br>− Monitor for signs and symptoms of dermatitis (rash and pruritus).<br>− IF THERE IS ANY BULLOUS FORMATION, THE STUDY PHYSICIAN SHOULD BE CONTACTED **AND STUDY DRUG DISCONTINUED.**
	**Grade 1**	No dose modifications.	**For Grade 1:**<br>− Consider symptomatic treatment, including oral antipruritics (e.g., diphenhydramine or hydroxyzine) and topical therapy (e.g., urea cream).
	**Grade 2**	For persistent (>1 to 2 weeks) Grade 2 events, hold scheduled study drug/study regimen until resolution to Grade ≤1 or baseline.<br>● If toxicity worsens, then treat as Grade 3.<br>● If toxicity improves to Grade ≤1 or baseline, then resume drug/study regimen after completion of steroid taper.	**For Grade 2:**<br>− Obtain dermatology consult.<br>− Consider symptomatic treatment, including oral antipruritics (e.g., diphenhydramine or hydroxyzine) and topical therapy (e.g., urea cream).<br>− Consider moderate-strength topical steroid.<br>− If no improvement of rash/skin lesions occurs within 3 to 5 days or is worsening despite symptomatic treatment and/or use of moderate strength topical steroid, consider, as necessary, discussing with study physician and promptly start systemic steroids such as prednisone 1 to 2 mg/kg/day PO or IV equivalent.<br>− Consider skin biopsy if the event is persistent for >1 to 2 weeks or recurs.
	**Grade 3 or 4**	**For Grade 3:**<br>Hold study drug/study regimen until resolution to Grade ≤1 or baseline.<br>If temporarily holding the study drug/study regimen does not provide improvement of the Grade 3 skin rash to Grade ≤1 or baseline within 30 days, then permanently discontinue study drug/study regimen.<br>**For Grade 4:**<br>Permanently discontinue study drug/study regimen.	**For Grade 3 or 4:**<br>− Consult dermatology.<br>− Promptly initiate empiric IV methylprednisolone 1 to 4 mg/kg/day or equivalent.<br>− Consider hospitalization.<br>− Monitor extent of rash [Rule of Nines].<br>− Consider skin biopsy (preferably more than 1) as clinically feasible.<br>− Once the patient is improving, gradually taper steroids over ≥28 days and consider prophylactic antibiotics, antifungals, and anti-PJP treatment (refer to current NCCN guidelines for treatment of cancer-related infections [Category 2B recommendation]).[a]<br>− Consider, as necessary, discussing with study physician.

**Endocrinopathy**<br>(e.g., hyperthyroidism, hypothyroidism, Type 1 diabetes mellitus, hypophysitis, hypopituitarism, and adrenal insufficiency; exocrine event of amylase/lipase increased also included in this section)	**Any Grade**<br>(depending on the type of endocrinopathy, refer to NCI CTCAE v4.03 for defining the CTC grade/severity)	**General Guidance**	**For Any Grade:**<br>− Consider consulting an endocrinologist for endocrine events.<br>− Consider, as necessary, discussing with study physician.<br>− Monitor patients for signs and symptoms of endocrinopathies. Non-specific symptoms include headache, fatigue, behavior changes, changed mental status, vertigo, abdominal pain, unusual bowel habits, polydipsia, polyuria, hypotension, and weakness.<br>− Patients should be thoroughly evaluated to rule out any alternative etiology (e.g., disease progression including brain metastases, or infections).<br>− Depending on the suspected endocrinopathy, monitor and evaluate thyroid function tests: TSH, free T3 and free T4 and other relevant endocrine and related labs (e.g., blood glucose and ketone levels, HgA1c).<br>− For modest asymptomatic elevations in serum amylase and lipase, corticosteroid treatment is not indicated as long as there are no other signs or symptoms of pancreatic inflammation.<br>− If a patient experiences an AE that is thought to be possibly of autoimmune nature (e.g., thyroiditis, pancreatitis, hypophysitis, or diabetes insipidus), the investigator should send a blood sample for appropriate autoimmune antibody testing.
	**Grade 1**	No dose modifications.	**For Grade 1 (including those with asymptomatic TSH elevation):**<br>− Monitor patient with appropriate endocrine function tests.<br>− For suspected hypophysitis/hypopituitarism, consider consultation of an endocrinologist to guide assessment of early-morning ACTH, cortisol, TSH and free T4; also consider gonadotropins, sex hormones, and prolactin levels, as well as cosyntropin stimulation test (though it may not be useful in diagnosing early secondary adrenal insufficiency).<br>− If TSH < 0.5 × LLN, or TSH >2 × ULN, or consistently out of range in 2 subsequent measurements, include free T4 at subsequent cycles as clinically indicated and consider consultation of an endocrinologist.

	**Grade 2**	For Grade 2 endocrinopathy other than hypothyroidism and Type 1 diabetes mellitus, hold study drug/study regimen dose until patient is clinically stable.<br>● If toxicity worsens, then treat as Grade 3 or Grade 4.<br>Study drug/study regimen can be resumed once event stabilizes and after completion of steroid taper.<br>Patients with endocrinopathies who may require prolonged or continued steroid replacement (e.g., adrenal insufficiency) can be retreated with study drug/study regimen on the following conditions:<br>1. The event stabilizes and is controlled.<br>2. The patient is clinically stable as per investigator or treating physician's clinical judgement.<br>3. Doses of prednisone are ≤10 mg/day or equivalent.	**For Grade 2 (including those with symptomatic endocrinopathy):**<br>– Consult endocrinologist to guide evaluation of endocrine function and, as indicated by suspected endocrinopathy and as clinically indicated, consider pituitary scan.<br>– For all patients with abnormal endocrine work up, except those with isolated hypothyroidism or Type 1 DM, and as guided by an endocrinologist, consider short-term corticosteroids (e.g., 1 to 2 mg/kg/day methylprednisolone or IV equivalent) and prompt initiation of treatment with relevant hormone replacement (e.g., hydrocortisone, sex hormones).<br>– Isolated hypothyroidism may be treated with replacement therapy, without study drug/study regimen interruption, and without corticosteroids.<br>– Isolated Type 1 diabetes mellitus (DM) may be treated with appropriate diabetic therapy, without study drug/study regimen interruption, and without corticosteroids.<br>– Once patients on steroids are improving, gradually taper immunosuppressive steroids (as appropriate and with guidance of endocrinologist) over ≥28 days and consider prophylactic antibiotics, antifungals, and anti-PJP treatment (refer to current NCCN guidelines for treatment of cancer-related infections [Category 2B recommendation]).[a]<br>– For patients with normal endocrine workup (laboratory assessment or MRI scans), repeat laboratory assessments/MRI as clinically indicated.

	**Grade 3 or 4**	For Grade 3 or 4 endocrinopathy other than hypothyroidism and Type 1 diabetes mellitus, hold study drug/study regimen dose until endocrinopathy symptom(s) are controlled.<br>Study drug/study regimen can be resumed once event stabilizes and after completion of steroid taper.<br>Patients with endocrinopathies who may require prolonged or continued steroid replacement (e.g., adrenal insufficiency) can be retreated with study drug/study regimen on the following conditions:<br>1. The event stabilizes and is controlled.<br>2. The patient is clinically stable as per investigator or treating physician's clinical judgement.<br>3. Doses of prednisone are ≤10 mg/day or equivalent.	**For Grade 3 or 4:**<br>– Consult endocrinologist to guide evaluation of endocrine function and, as indicated by suspected endocrinopathy and as clinically indicated, consider pituitary scan. Hospitalization recommended.<br>– For all patients with abnormal endocrine work up, except those with isolated hypothyroidism or Type 1 DM, and as guided by an endocrinologist, promptly initiate empiric IV methylprednisolone 1 to 2 mg/kg/day or equivalent, as well as relevant hormone replacement (e.g., hydrocortisone, sex hormones).<br>– For adrenal crisis, severe dehydration, hypotension, or shock, immediately initiate IV corticosteroids with mineralocorticoid activity.<br>– Isolated hypothyroidism may be treated with replacement therapy, without study drug/study regimen interruption, and without corticosteroids.<br>– Isolated Type 1 diabetes mellitus may be treated with appropriate diabetic therapy, without study drug/study regimen interruption, and without corticosteroids.<br>– Once patients on steroids are improving, gradually taper immunosuppressive steroids (as appropriate and with guidance of endocrinologist) over ≥28 days and consider prophylactic antibiotics, antifungals, and anti-PJP treatment (refer to current NCCN guidelines for treatment of cancer-related infections [Category 2B recommendation]).[a]
**Neurotoxicity**<br>(to include but not be limited to limbic encephalitis and autonomic neuropathy, excluding Myasthenia Gravis and Guillain-Barre)	**Any Grade**<br>(depending on the type of neurotoxicity, refer to NCI CTCAE v4.03 for defining the CTC grade/severity)	**General Guidance**	**For Any Grade:**<br>– Patients should be evaluated to rule out any alternative etiology (e.g., disease progression, infections, metabolic syndromes, or medications).<br>– Monitor patient for general symptoms (headache, nausea, vertigo, behavior change, or weakness).<br>– Consider appropriate diagnostic testing (e.g., electromyogram and nerve conduction investigations).<br>– Perform symptomatic treatment with neurological consult as appropriate.<br>–
	**Grade 1**	No dose modifications.	**For Grade 1:**<br>– See "Any Grade" recommendations above.

	**Grade 2**	For acute motor neuropathies or neurotoxicity, hold study drug/study regimen dose until resolution to Grade ≤1.<br>For sensory neuropathy/neuropathic pain, consider holding study drug/study regimen dose until resolution to Grade ≤1.<br>If toxicity worsens, then treat as Grade 3 or 4.<br>Study drug/study regimen can be resumed once event improves to Grade ≤1 and after completion of steroid taper.	**For Grade 2:**<br>– Consider, as necessary, discussing with the study physician.<br>– Obtain neurology consult.<br>– Sensory neuropathy/neuropathic pain may be managed by appropriate medications (e.g., gabapentin or duloxetine).<br>– Promptly start systemic steroids prednisone 1 to 2 mg/kg/day PO or IV equivalent.<br>– If no improvement within 3 to 5 days despite 1 to 2 mg/kg/day prednisone PO or IV equivalent, consider additional workup and promptly treat with additional immunosuppressive therapy (e.g., IV IG).
	**Grade 3 or 4**	**For Grade 3:**<br>Hold study drug/study regimen dose until resolution to Grade ≤1.<br>Permanently discontinue study drug/study regimen if Grade 3 imAE does not resolve to Grade ≤1 within 30 days.<br>**For Grade 4:**<br>Permanently discontinue study drug/study regimen.	**For Grade 3 or 4:**<br>– Consider, as necessary, discussing with study physician.<br>– Obtain neurology consult.<br>– Consider hospitalization.<br>– Promptly initiate empiric IV methylprednisolone 1 to 2 mg/kg/day or equivalent.<br>– If no improvement within 3 to 5 days despite IV corticosteroids, consider additional workup and promptly treat with additional immunosuppressants (e.g., IV IG).<br>– Once stable, gradually taper steroids over ≥28 days.
**Peripheral neuromotor syndromes**<br>(such as Guillain-Barre and myasthenia gravis)	**Any Grade**	**General Guidance**	**For Any Grade:**<br>– The prompt diagnosis of immune-mediated peripheral neuromotor syndromes is important, since certain patients may unpredictably experience acute decompensations that can result in substantial morbidity or in the worst case, death. Special care should be taken for certain sentinel symptoms that may predict a more severe outcome, such as prominent dysphagia, rapidly progressive weakness, and signs of respiratory insufficiency or autonomic instability.<br>– Patients should be evaluated to rule out any alternative etiology (e.g., disease progression, infections, metabolic syndromes or medications). It should be noted that the diagnosis of immune-mediated peripheral neuromotor syndromes can be particularly challenging in patients with underlying cancer, due to the multiple potential confounding effects of cancer (and its treatments) throughout the neuraxis. Given the importance of

			prompt and accurate diagnosis, it is essential to have a low threshold to obtain a neurological consult.<br>− Neurophysiologic diagnostic testing (e.g., electromyogram and nerve conduction investigations, and "repetitive stimulation" if myasthenia is suspected) are routinely indicated upon suspicion of such conditions and may be best facilitated by means of a neurology consultation.<br>− It is important to consider that the use of steroids as the primary treatment of Guillain-Barre is not typically considered effective. Patients requiring treatment should be started with IV IG and followed by plasmapheresis if not responsive to IV IG.
	**Grade 1**	No dose modifications.	**For Grade 1:**<br>− Consider, as necessary, discussing with the study physician.<br>− Care should be taken to monitor patients for sentinel symptoms of a potential decompensation as described above.<br>− Obtain a neurology consult.
	**Grade 2**	Hold study drug/study regimen dose until resolution to Grade ≤1.<br>Permanently discontinue study drug/study regimen if it does not resolve to Grade ≤1 within 30 days or if there are signs of respiratory insufficiency or autonomic instability.	**For Grade 2:**<br>− Consider, as necessary, discussing with the study physician.<br>− Care should be taken to monitor patients for sentinel symptoms of a potential decompensation as described above.<br>− Obtain a neurology consult<br>− Sensory neuropathy/neuropathic pain may be managed by appropriate medications (e.g., gabapentin or duloxetine).<br>*MYASTHENIA GRAVIS:*<br>o Steroids may be successfully used to treat myasthenia gravis. It is important to consider that steroid therapy (especially with high doses) may result in transient worsening of myasthenia and should typically be administered in a monitored setting under supervision of a consulting neurologist.<br>o Patients unable to tolerate steroids may be candidates for treatment with plasmapheresis or IV IG. Such decisions are best made in consultation with a neurologist, taking into account the unique needs of each patient.<br>o If myasthenia gravis-like neurotoxicity is present, consider

			starting AChE inhibitor therapy in addition to steroids. Such therapy, if successful, can also serve to reinforce the diagnosis.<br>*GUILLAIN-BARRE:*<br>o It is important to consider here that the use of steroids as the primary treatment of Guillain-Barre is not typically considered effective.<br>o Patients requiring treatment should be started with IV IG and followed by plasmapheresis if not responsive to IV IG.
	**Grade 3 or 4**	**For Grade 3:**<br>Hold study drug/study regimen dose until resolution to Grade ≤1.<br>Permanently discontinue study drug/study regimen if Grade 3 imAE does not resolve to Grade ≤1 within 30 days or if there are signs of respiratory insufficiency or autonomic instability.<br>**For Grade 4:**<br>Permanently discontinue study drug/study regimen.	**For Grade 3 or 4 (severe or life-threatening events):**<br>− Consider, as necessary, discussing with study physician.<br>− Recommend hospitalization.<br>− Monitor symptoms and obtain neurological consult.<br>*MYASTHENIA GRAVIS:*<br>o Steroids may be successfully used to treat myasthenia gravis. They should typically be administered in a monitored setting under supervision of a consulting neurologist.<br>o Patients unable to tolerate steroids may be candidates for treatment with plasmapheresis or IV IG.<br>o If myasthenia gravis-like neurotoxicity present, consider starting AChE inhibitor therapy in addition to steroids. Such therapy, if successful, can also serve to reinforce the diagnosis.<br>*GUILLAIN-BARRE:*<br>o It is important to consider here that the use of steroids as the primary treatment of Guillain-Barre is not typically considered effective.<br>o Patients requiring treatment should be started with IV IG and followed by plasmapheresis if not responsive to IV IG.
**Myocarditis**	**Any Grade**	**General Guidance**<br>Discontinue drug permanently if biopsy-proven immune-mediated myocarditis.	**For Any Grade:**<br>− The prompt diagnosis of immune-mediated myocarditis is important, particularly in patients with baseline cardiopulmonary disease and reduced cardiac function.

			− Consider, as necessary, discussing with the study physician.<br>− Monitor patients for signs and symptoms of myocarditis (new onset or worsening chest pain, arrhythmia, shortness of breath, peripheral edema). As some symptoms can overlap with lung toxicities, simultaneously evaluate for and rule out pulmonary toxicity as well as other causes (e.g., pulmonary embolism, congestive heart failure, malignant pericardial effusion). A Cardiology consultation should be obtained early, with prompt assessment of whether and when to complete a cardiac biopsy, including any other diagnostic procedures.<br>− Initial work-up should include clinical evaluation, BNP, cardiac enzymes, ECG, echocardiogram (ECHO), monitoring of oxygenation via pulse oximetry (resting and exertion), and additional laboratory work-up as indicated. Spiral CT or cardiac MRI can complement ECHO to assess wall motion abnormalities when needed.<br>− Patients should be thoroughly evaluated to rule out any alternative etiology (e.g., disease progression, other medications, or infections)
	**Grade 1**<br>(asymptomatic with laboratory (e.g., BNP) or cardiac imaging abnormalities)	No dose modifications required unless clinical suspicion is high, in which case hold study drug/study regimen dose during diagnostic work-up for other etiologies. If study drug/study regimen is held, resume after complete resolution to Grade 0.	**For Grade 1 (no definitive findings):**<br>- Monitor and closely follow up in 2 to 4 days for clinical symptoms, BNP, cardiac enzymes, ECG, ECHO, pulse oximetry (resting and exertion), and laboratory work-up as clinically indicated.<br>- Consider using steroids if clinical suspicion is high.
	**Grade 2, 3 or 4**<br>(Grade 2: Symptoms with mild to moderate activity or exertion)<br><br>(Grade 3: Severe with symptoms at rest or with minimal activity or exertion; intervention indicated)<br><br>(Grade 4: Life-threatening	- If Grade 2 -- Hold study drug/study regimen dose until resolution to Grade 0. If toxicity rapidly improves to Grade 0, then the decision to reinitiate study drug/study regimen will be based upon treating physician's clinical judgment and after completion of steroid taper. If toxicity does not rapidly improve, permanently. discontinue study drug/study regimen.<br>If Grade 3-4, permanently discontinue study drug/study regimen.	**For Grade 2-4:**<br>− Monitor symptoms daily, hospitalize.<br>− Promptly start IV methylprednisolone 2 to 4 mg/kg/day or equivalent after Cardiology consultation has determined whether and when to complete diagnostic procedures including a cardiac biopsy.<br>− Supportive care (e.g., oxygen).<br>− If no improvement within 3 to 5 days despite IV methylprednisolone at 2 to 4 mg/kg/day, promptly start immunosuppressive therapy such as TNF inhibitors (e.g., infliximab at 5 mg/kg every 2 weeks). Caution: It is important to rule out sepsis and refer to infliximab label for general guidance before using infliximab.<br>− Once the patient is improving, gradually taper steroids over ≥28 days and consider prophylactic antibiotics, antifungals, or anti-PJP treatment (refer to current NCCN

	consequences; urgent intervention indicated (e.g., continuous IV therapy or mechanical hemodynamic support))		guidelines for treatment of cancer-related infections [Category 2B recommendation]).[a]
**Myositis/Polymyositis (“Poly/myositis”)**	**Any Grade**	**General Guidance**	**For Any Grade:**<br>− Monitor patients for signs and symptoms of poly/myositis. Typically, muscle weakness/pain occurs in proximal muscles including upper arms, thighs, shoulders, hips, neck and back, but rarely affects the extremities including hands and fingers; also difficulty breathing and/or trouble swallowing can occur and progress rapidly. Increased general feelings of tiredness and fatigue may occur, and there can be new-onset falling, difficulty getting up from a fall, and trouble climbing stairs, standing up from a seated position, and/or reaching up.<br>− If poly/myositis is suspected, a Neurology consultation should be obtained early, with prompt guidance on diagnostic procedures. Myocarditis may co-occur with poly/myositis; refer to guidance under Myocarditis. Given breathing complications, refer to guidance under Pneumonitis/ILD.<br>Given possibility of an existent (but previously unknown) autoimmune disorder, consider Rheumatology consultation.<br>− Consider, as necessary, discussing with the study physician.<br>− Initial work-up should include clinical evaluation, creatine kinase, aldolase, LDH, BUN/creatinine, erythrocyte sedimentation rate or C-reactive protein level, urine myoglobin, and additional laboratory work-up as indicated, including a number of possible rheumatological/antibody tests (i.e., consider whether a rheumatologist consultation is indicated and could guide need for rheumatoid factor, antinuclear antibody, anti-smooth muscle, antisynthetase [such as anti-Jo-1], and/or signal-recognition particle antibodies). Confirmatory testing may include electromyography, nerve conduction studies, MRI of the muscles, and/or a muscle biopsy. Consider Barium swallow for evaluation of dysphagia or dysphonia.

			Patients should be thoroughly evaluated to rule out any alternative etiology (e.g., disease progression, other medications, or infections).
	**Grade 1**<br>(mild pain)	- No dose modifications.	**For Grade 1:**<br>− Monitor and closely follow up in 2 to 4 days for clinical symptoms and initiate evaluation as clinically indicated.<br>− Consider Neurology consult.<br>− Consider, as necessary, discussing with the study physician.
	**Grade 2**<br>(moderate pain associated with weakness; pain limiting instrumental activities of daily living [ADLs])	Hold study drug/study regimen dose until resolution to Grade ≤1.<br>- Permanently discontinue study drug/study regimen if it does not resolve to Grade ≤1 within 30 days or if there are signs of respiratory insufficiency.	**For Grade 2:**<br>− Monitor symptoms daily and consider hospitalization.<br>− Obtain Neurology consult, and initiate evaluation.<br>− Consider, as necessary, discussing with the study physician.<br>− If clinical course is rapidly progressive (particularly if difficulty breathing and/or trouble swallowing), promptly start IV methylprednisolone 2 to 4 mg/kg/day systemic steroids along with receiving input from Neurology consultant<br>− If clinical course is *not* rapidly progressive, start systemic steroids (e.g., prednisone 1 to 2 mg/kg/day PO or IV equivalent); if no improvement within 3 to 5 days, continue additional work up and start treatment with IV methylprednisolone 2 to 4 mg/kg/day<br>− If after start of IV methylprednisolone at 2 to 4 mg/kg/day there is no improvement within 3 to 5 days, consider start of immunosuppressive therapy such as TNF inhibitors (e.g., infliximab at 5 mg/kg every 2 weeks). Caution: It is important to rule out sepsis and refer to infliximab label for general guidance before using infliximab.<br>− Once the patient is improving, gradually taper steroids over ≥28 days and consider prophylactic antibiotics, antifungals, or anti-PJP treatment (refer to current NCCN guidelines for treatment of cancer-related infections [Category 2B recommendation]).[a]
	**Grade 3 or 4**<br>(pain associated with severe weakness; limiting self-care ADLs)	**For Grade 3:**<br>Hold study drug/study regimen dose until resolution to Grade ≤1.<br>Permanently discontinue study drug/study regimen if Grade 3 imAE does not resolve to Grade ≤1 within 30 days or if there are	**For Grade 3 or 4 (severe or life-threatening events):**<br>− Monitor symptoms closely; recommend hospitalization.<br>− Obtain Neurology consult, and complete full evaluation.<br>− Consider, as necessary, discussing with the study physician.

		signs of respiratory insufficiency.<br>**For Grade 4:**<br>- Permanently discontinue study drug/study regimen.	− Promptly start IV methylprednisolone 2 to 4 mg/kg/day systemic steroids along with receiving input from Neurology consultant.<br>− If after start of IV methylprednisolone at 2 to 4 mg/kg/day there is no improvement within 3 to 5 days, consider start of immunosuppressive therapy such as TNF inhibitors (e.g., infliximab at 5 mg/kg every 2 weeks). Caution: It is important to rule out sepsis and refer to infliximab label for general guidance before using infliximab.<br>− Consider whether patient may require IV IG, plasmapheresis.<br>− Once the patient is improving, gradually taper steroids over ≥28 days and consider prophylactic antibiotics, antifungals, or anti-PJP treatment (refer to current NCCN guidelines for treatment of cancer-related infections [Category 2B recommendation]).[a]

[a]ASCO Educational Book 2015 “Managing Immune Checkpoint Blocking Antibody Side Effects” by Michael Postow MD.

[b]FDA Liver Guidance Document 2009 Guidance for Industry: Drug Induced Liver Injury – Premarketing Clinical Evaluation.

AChE Acetylcholine esterase; ADL Activities of daily living; AE Adverse event; ALP Alkaline phosphatase test; ALT Alanine aminotransferase; AST Aspartate aminotransferase; BUN Blood urea nitrogen; CT Computed tomography; CTCAE Common Terminology Criteria for Adverse Events; ILD Interstitial lung disease; imAE immune-mediated adverse event; IG Immunoglobulin; IV Intravenous; GI Gastrointestinal; LFT Liver function tests; LLN Lower limit of normal; MRI Magnetic resonance imaging; NCI National Cancer Institute; NCCN National Comprehensive Cancer Network; PJP *Pneumocystis jirovecii* pneumonia (formerly known as *Pneumocystis carinii* pneumonia); PO By mouth; T3 Triiodothyronine; T4 Thyroxine; TB Total bilirubin; TNF Tumor necrosis factor; TSH Thyroid-stimulating hormone; ULN Upper limit of normal.

Infusion-Related Reactions		
**Severity Grade of the Event (NCI CTCAE version 4.03)**	**Dose Modifications**	**Toxicity Management**
**Any Grade**	**General Guidance**	**For Any Grade:**<br>− Manage per institutional standard at the discretion of investigator.<br>− Monitor patients for signs and symptoms of infusion-related reactions (e.g., fever and/or shaking chills, flushing and/or itching, alterations in heart rate and blood pressure, dyspnea or chest discomfort, or skin rashes) and anaphylaxis (e.g., generalized urticaria, angioedema, wheezing, hypotension, or tachycardia).
**Grade 1 or 2**	**For Grade 1:**<br>The infusion rate of study drug/study regimen may be decreased by 50% or temporarily interrupted until resolution of the event.<br>**For Grade 2:**<br>The infusion rate of study drug/study regimen may be decreased 50% or temporarily interrupted until resolution of the event.<br>Subsequent infusions may be given at 50% of the initial infusion rate.	**For Grade 1 or 2:**<br>− Acetaminophen and/or antihistamines may be administered per institutional standard at the discretion of the investigator.<br>− Consider premedication per institutional standard prior to subsequent doses.<br>− Steroids should not be used for routine premedication of Grade ≤2 infusion reactions.
**Grade 3 or 4**	**For Grade 3 or 4:**<br>Permanently discontinue study drug/study regimen.	**For Grade 3 or 4:**<br>− Manage severe infusion-related reactions per institutional standards (e.g., IM epinephrine, followed by IV diphenhydramine and ranitidine, and IV glucocorticoid).

CTCAE Common Terminology Criteria for Adverse Events; IM intramuscular; IV intravenous; NCI National Cancer Institute.

Non–Immune-Mediated Reactions		
**Severity Grade of the Event (NCI CTCAE version 4.03)**	**Dose Modifications**	**Toxicity Management**
**Any Grade**	Note: Dose modifications are not required for AEs not deemed to be related to study treatment (i.e., events due to underlying disease) or for laboratory abnormalities not deemed to be clinically significant.	Treat accordingly, as per institutional standard.
**Grade 1**	No dose modifications.	Treat accordingly, as per institutional standard.
**Grade 2**	Hold study drug/study regimen until resolution to ≤Grade 1 or baseline.	Treat accordingly, as per institutional standard.
**Grade 3**	Hold study drug/study regimen until resolution to ≤Grade 1 or baseline.<br>For AEs that downgrade to ≤Grade 2 within 7 days or resolve to ≤Grade 1 or baseline within 14 days, resume study drug/study regimen administration. Otherwise, discontinue study drug/study regimen.	Treat accordingly, as per institutional standard.
**Grade 4**	Discontinue study drug/study regimen (Note: For Grade 4 labs, decision to discontinue should be based on accompanying clinical signs/symptoms, the Investigator's clinical judgment, and consultation with the Sponsor.).	Treat accordingly, as per institutional standard.

Note: As applicable, for early phase studies, the following sentence may be added: "Any event greater than or equal to Grade 2, please discuss with Study Physician."

AE Adverse event; CTCAE Common Terminology Criteria for Adverse Events; NCI National Cancer Institute.

Appendix 2 Schedule of study procedures follow-up for subjects who have completed durvalumab and tremelimumab treatment and achieved disease control (until confirmed progression of disease) and subjects who have discontinued durvalumab or tremelimumab due to toxicity in the absence of confirmed progression of disease.

Evaluation	Time Since Last Dose							
	Day (±3)	Months (±1 week)						12 Months and every 6 Months (±2 weeks)
	30	2	3	4	6	8	10	
Physical examination[a]	x	x	x	x	x	x	x	
Vital signs (temperature, respiratory rate, blood, pressure, pulse)	x	x	x	x	x	x	x	
Weight	x	x	x	x	x	x	x	
Urine hCG or serum βhCG	x							
AE/SAE assessment	x	x	x					
Concomitant medications	x	x	x					
Palliative radiotherapy	As clinically indicated							
ECOG performance status	x	x	x		X (and month 9)			x
Subsequent anti/cancer therapy	x	x	x	x	x	x	x	x
Survival status: phone contact with subjects who refuse to return for evaluations and agree to be contacted		x	x	x	x	x	x	x (every 2 months)
Hematology	x	Every 8-12 weeks, according to investigator's criteria						x
Serum chemistry	x	Every 8-12 weeks, according to investigator's criteria						
Thyroid function tests)TSH, and fT3 and fT4)[b]	x	Every 8-12 weeks, according to investigator's criteria						
SPD-L1 concentration)to assess target engagement)			x					
Tumour assessment (CT or MRI)	For subjects who **achieve disease control following 12 months of treatment**, tumour assessments should be performed every 12 weeks relative to the date of **first infusion** thereafter until confirmed PD by RECIST 1.1 by investigational site review. Please refer to SCHEDULE OF STUDY ASSESSMENTS for timings of confirmatory scans.<br>For subjects who **discontinue study drug due to toxicity (or symptomatic deterioration)**, tumour assessments should be performed relative to the relative to the date of first infusion as follows: every 12 weeks until confirmed PD by RECIST 1.1 by investigational site review. Please refer to SCHEDULE OF STUDY ASSESSMENTS for timings of confirmatory scans.<br>Upon confirmed PD, scans should be conducted according to local standard clinical practice and submitted for central review until a new treatment is started (these scans are optional).							

[a] Full physical exam
[b] Free T3 and free T4 will only be measured if TSH is abnormal. They should also be measured if there is clinical suspicion of an adverse event related to the endocrine system.
[c] For patient questionnaires different approaches based on indication and study design.

Appendix 3 Schedule of study procedures: follow-up for subjects who have discontinued durvalumab or tremelimumab treatment due to confirmed progression of disease at the investigator discretion

Evaluation	Time Since Last Dose							
	Day (±3)	Months (±1 week)						12 Months and every 6 Months (±2 weeks)
	30	2	3	4	6	8	10	
Physical examination[a]	x							
Vital signs (temperature, respiratory rate, blood, pressure, pulse)	x							
Weight	x							
AE/SAE assessment	x	x	x					
Concomitant medications	x	x	x					
Palliative radiotherapy	As clinically indicated							
ECOG performance status[b]	x	x	x					
Subsequent anti/cancer therapy	x	x	x	x	x	x	x	x
Survival status: phone contact with subjects who refuse to return for evaluations and agree to be contacted		x	x	x	x	x	x	x (every 2 months)
Urine hCG or serum βhCG	x							
Hematology	x	x	x					
Serum chemistry	x	x	x					
Thyroid function tests)TSH, and fT3 and fT4)[c]			x					
Pharmacokinetic assessment, if applicable			x					
SPD-L1 concentration)to assess target engagement)			x					
Tumour assessment (CT or MRI)	For subjects who continue on MEDI4736 post-confirmed progression at the investigator's discretion (following consultation with the sponsor), tumour assessments should be performed relative to the date of first infusion per SCHEDULE OF STUDY ASSESSMENTS until study drug is stopped.<br>For subjects who discontinue study drug following confirmed progression, scans should be conducted according to local clinical practice and submitted for central review until a new treatment is started (these scans are optional).							

[A] Full physical exam
[B] PS to be collected if available at the 2 monthly calls to obtain subsequent anti/cancer therapy and survival status
c Free T3 and free T4 will only be measured if TSH is abnormal. They should also be measured if there is clinical suspicion of an adverse event related to the endocrine system.
[D] For patient questionnaires different approaches based on indication and study design.

Appendix 4 Durvalumab dose calculations

For durvalumab done depending on subject weight:

1. Cohort dose: X mg/kg
2. Subject weight: Y kg
3. Dose for subject: XY mg = X(mg/kg) x Y(kg)
4. Dose to be added into infusion bag:

   Dose (mL) = XY mg / 50 (mg/mL)

   Where 50 mg/mL is durvalumab nominal concentration.

   The corresponding volume of durvalumab should be rounded to the nearest tenth mL (0.1 mL). Dose adjustments for each cycle are only needed for greater than 10% change in weight.

5. The theoretical number of vials required for dose preparation is the next greatest whole number of vials from the following formula:

   Number of vials = Dose (mL) / 10.0 (mL/vial)

Example:

1. Cohort dose: 10 mg/kg

2. Subject weight: 30 kg

3. Dose for subject: 300 mg = 10 (mg/kg) x 30(kg)

4. Dose to be added into infusion bag:

Dose (mL) = 300 mg / 50 (mg/mL) = 6.0 mL

5. The theoretical number of vials required for dose preparation:

Number of vials = 6.0(mL) / 10.0 (mL/vial) = 1 vials

Appendix 5 Durvalumab Dose Volume Calculations

For durvalumab flat dosing:

1. Cohort dose: X g

2. Dose to be added into infusion bag:

Dose (mL) = X g x 1000/50 (mg/mL)

where 50 mg/mL is durvalumab nominal concentration.

The corresponding volume of durvalumab should be rounded to the nearest tenth mL (0.1 mL)

3. The theoretical number of vials required for dose preparation is the next greatest whole number of vials from the following formula:

Number of vials = Dose (mL) / 10.0 (mL/vial)

Example:

1. Cohort dose: 1.5 g

2. Dose to be added into infusion bag:

Dose (mL) = 1.5 g x 1000 / 50 (mg/mL) = 30.0 mL

3. The theoretical number of vials required for dose preparation:

Number of vials = 30.0 (mL) / 10.0 (mL/vial) = 3 vials

Appendix 6 Tremelimumab Dose Calculations

For tremelimumab dosing done depending on subject weight:

1. Cohort dose: X mg/kg
2. Subject weight: Y kg
3. Dose for subject: XY mg = X(mg/kg) x Y(kg)
4. Dose to be added into infusion bag:

   Dose (mL) = XY mg / 20 (mg/mL)

   Where 20 mg/mL is tremelimumab nominal concentration.

   The corresponding volume of tremelimumab should be rounded to the nearest tenth mL (0.1 mL). Dose adjustments for each cycle are only needed for greater then 10% change in weight.

5. The theoretical number of vials required for dose preparation is the next greatest whole number of vials from the following formula:

   Number of vials = Dose (mL) / 20.0 (mL/vial)

Example:

1. Cohort dose: 1 mg/kg

2. Subject weight: 30 kg

3. Dose for subject: 30 mg = 1 (mg/kg) x 30(kg)

4. Dose to be added into infusion bag:

Dose (mL) = 30 mg / 20 (mg/mL) = 1.5 mL

5. The theoretical number of vials required for dose preparation:

Number of vials = 1.5(mL) / 20.0 (mL/vial) = 1 vials

Appendix 7 Tremelimumab Dose Volume Calculations

For tremelimumab flat dosing:

1. Cohort dose: X mg

2. Dose to be added into infusion bag:

Dose (mL) = X mg /20 (mg/mL)

where 20 mg/mL is tremelimumab nominal concentration.

The corresponding volume of tremelimumab should be rounded to the nearest tenth mL (0.1 mL)

3. The theoretical number of vials required for dose preparation is the next greatest whole number of vials from the following formula:

Number of vials = Dose (mL) / 20 (mL/vial)

Example:

1. Cohort dose: 75 mg

2. Dose to be added into infusion bag:

Dose (mL) = 75 mg / 20 (mg/mL) = 3.8 mL

3. The theoretical number of vials required for dose preparation:

Number of vials = 3.8 (mL) / 20 (mL/vial) = 1 vial

Appendix 8 Contact Details

Sponsor: GETNE (Spanish Group of Neuroendocrine and Endocrine Tumors)

[redacted]

Coordinating Investigator: Dr. Jaume Capdevila

[redacted]

CRO: [redacted]

Appendix 9 Sites and Investigators Participants

	SITE	Principal Investigator
1 -	[redacted]	
2 -		
3 -		
4 -		
5 -		
6 -		
7 -		
8 -		
9 -		
10 -		
11 -		
12 -		
13 -		
14 -		
15 -		
16 -		
17 -		
18 -		
19 -		
20 -		
