# Supplementary material for: Durvalumab plus tremelimumab for the treatment of advanced neuroendocrine neoplasms of gastroenteropancreatic and lung origin
Source: Nat Commun. 2023 May 23;14:2973. doi: 10.1038/s41467-023-38611-5 (PMC10204675; doi:10.1038/s41467-023-38611-5)
Supplement: Supplementary file 3 — Reporting Summary [file 41467_2023_38611_MOESM3_ESM.pdf]

nature portfolio

Corresponding author(s): DR. Capdevila

Last updated by author(s): 8MAY2023

Reporting Summary

Nature Portfolio wishes to improve the reproducibility of the work that we publish. This form provides structure for consistency and transparency in reporting. For further information on Nature Portfolio policies, see our Editorial Policies and the Editorial Policy Checklist.

Statistics

For all statistical analyses, confirm that the following items are present in the figure legend, table legend, main text, or Methods section.

n/a	Confirmed	
☐	☒	The exact sample size (*n*) for each experimental group/condition, given as a discrete number and unit of measurement
☐	☒	A statement on whether measurements were taken from distinct samples or whether the same sample was measured repeatedly
☐	☒	The statistical test(s) used AND whether they are one- or two-sided<br>*Only common tests should be described solely by name; describe more complex techniques in the Methods section.*
☐	☒	A description of all covariates tested
☐	☒	A description of any assumptions or corrections, such as tests of normality and adjustment for multiple comparisons
☐	☒	A full description of the statistical parameters including central tendency (e.g. means) or other basic estimates (e.g. regression coefficient) AND variation (e.g. standard deviation) or associated estimates of uncertainty (e.g. confidence intervals)
☐	☒	For null hypothesis testing, the test statistic (e.g. *F*, *t*, *r*) with confidence intervals, effect sizes, degrees of freedom and *P* value noted<br>*Give P values as exact values whenever suitable.*
☒	☐	For Bayesian analysis, information on the choice of priors and Markov chain Monte Carlo settings
☒	☐	For hierarchical and complex designs, identification of the appropriate level for tests and full reporting of outcomes
☐	☒	Estimates of effect sizes (e.g. Cohen's *d*, Pearson's *r*), indicating how they were calculated

Our web collection on statistics for biologists contains articles on many of the points above.

Software and code

Policy information about availability of computer code

Data collection	eCRF from MFAR Clinical Research. 15O9001 validated data caoture toolkit for clinical research
Data analysis	All statistical analyses were performed with R and SPSS (IBM SPSS Statistics Version 26, Armonk, NY). Figures and tables were generated using RStudio (Version 1.2.5033 2009-2019 RStudio, Inc., Boston, MA, US). Sample size was calculated using a one-sample superiority test, function One Sample Proportion NIS of the Trial Size package of R software (version 3.6.3 [2020-02-29] "Holding the Windsock". The R Foundation for Statistical Computing, Vienna, Austria)

For manuscripts utilizing custom algorithms or software that are central to the research but not yet described in published literature, software must be made available to editors and reviewers. We strongly encourage code deposition in a community repository (e.g. GitHub). See the Nature Portfolio guidelines for submitting code & software for further information.

Data

Policy information about availability of data

All manuscripts must include a data availability statement. This statement should provide the following information, where applicable:

- Accession codes, unique identifiers, or web links for publicly available datasets
- A description of any restrictions on data availability
- For clinical datasets or third party data, please ensure that the statement adheres to our policy

Study protocol is available as Supplementary Note in the Supplementary Information file.

March 2021

The raw data are protected and are not available due to data privacy laws. The data that support the findings of this study are available from the corresponding author upon reasonable request (equivalent purposes to those for which the patients grant their consent to use the data: i.e. for research in neuroendocrine neoplasms). Data will be provided anonymously, with no identifiable data. The remaining data are available within the Article, Supplementary Information or Source Data file.

Human research participants

Policy information about studies involving human research participants and Sex and Gender in Research.

Reporting on sex and gender	results apply to both sex or gender.<br>Age and sex in our cohort are reported in table 1. Results are independent of sex and gender or age.<br>Sex was extracted from medical records. Gender self-reporting verbally to investigators was respected if applicable. There was no systematic assessment for gender.
Population characteristics	Eligible patients presented with a histologically confirmed diagnosis of advanced/metastatic NEN and had progressed to standard anticancer therapies according to tumour type. Patients were enrolled in four cohorts according to the type of NET: well-moderately differentiated NETs of the lung, also known as typical and atypical lung carcinoids, that have progressed to prior somatostatin analogues therapy, one prior targeted therapy or chemotherapy (Cohort 1); well-moderately differentiated, World Health Organization (WHO) grade 1 and 2, gastrointestinal NETs after progression to somatostatin analogues and one targeted therapy, interferon or radionuclides (Cohort 2); well-moderately differentiated, WHO grade 1 and 2 NET, from pancreatic origin after progression to at least two and a maximum of four standard therapies, including chemotherapy, somatostatin analogues and target therapy (Cohort 3); WHO grade 3 NENs, of gastroenteropancreatic or unknown primary origin, excluding lung primary carcinomas, after progression to first-line chemotherapy with a platinum-based regimen (Cohort 4). The trial used the WHO 2010 classification for NETs. General inclusion criteria also included patients > 18 years; Eastern Cooperative Oncology Group performance status (ECOG PS) 0-1; life expectancy > 12 weeks; adequate haematologic, hepatic, and renal function; measurable disease according to Response Criteria in Solid Tumours (RECIST) version 1.1 24; and documented radiological disease progression according to RECIST 1.1 within 12 months prior to inclusion The exclusion criteria were as follows: prior treatment with anti-PDL-1/anti-programmed death 1 (PD-1) or anti-CTLA-4 therapy; immunodeficiency or use of immunosuppressive medication history within 28 days before the first dose of durvalumab or tremelimumab, with the exception of intranasal and inhaled corticosteroids or systemic corticosteroids at physiological doses not exceeding 10 mg/day of prednisone, or equivalent; active or prior documented autoimmune disease within the past 2 years; previous or active interstitial lung disease, or non-infectious pneumonitis; presence of active brain metastases or secondary malignancies.
Recruitment	Accrual was competitive and sequential.
Ethics oversight	This study was conducted in accordance with the principles of the Declaration of Helsinki and the International Conference on Harmonization Guidelines for Good Clinical Practice. The study protocol (see supplementary information) was approved in the first instance in 2017 by the competent authority in Spain and the Independent Ethics Committee from Vall d'Hebron University Hospital. Written informed consent was obtained from all patients.

Note that full information on the approval of the study protocol must also be provided in the manuscript.

Field-specific reporting

Please select the one below that is the best fit for your research. If you are not sure, read the appropriate sections before making your selection.

☒ Life sciences ☐ Behavioural & social sciences ☐ Ecological, evolutionary & environmental sciences

For a reference copy of the document with all sections, see nature.com/documents/nr-reporting-summary-flat.pdf

Life sciences study design

All studies must disclose on these points even when the disclosure is negative.

Sample size	Sample size was calculated using a one-sample superiority test, function One Sample Proportion NIS of the Trial Size package of R software (version 3.6.3 [2020-02-29] "Holding the Windsock". The R Foundation for Statistical Computing, Vienna, Austria). For Cohorts 1 to 3, according to previous reports, it was assumed that the 9-m DCR was 30% (null hypothesis) and a potential 20% increase was estimated with a superiority margin of 10% (alternative hypothesis). For cohort 4, according to previous reports 12-14, it was assumed that the 9-m OS rate was 13% (null hypothesis) and a 10% increase with a superiority margin of 5% (alternative hypothesis) was estimated. With a unilateral alpha level of 5% and 80% power and 10% loss to follow-up rate, the required sample size was: 31 in Cohorts 1 to 3, and 33 in Cohort 4 (126 patients in total).
Data exclusions	No data was exluded from the study. Patient who underrwent screenineng and were not eligible according to inclusion/exclusion criteria were declared screening failure and did not receive treatment. Any further data was collected for these patients.

Replication	4 patients received treatment twice, were re-treated as allowed by protocol specifications. This patients were analyzed for their first course but censored for the second treatment round.
Randomization	Dune (NCT03095274) is a non-randomized controlled multicohort phase II clinical trial evaluating durvalumab plus tremelimumab activity and safety in advanced NENs. The trial has only 1 arm (experimental treatment). Therefore, randomization does not apply.
Blinding	Dune was an open-label trial. As there is only one treatment arm studied, there is no need of placebo. The study was designed as a phase II to gather initial efficacy in NENs, which is an uncommon rare disease. Due to small patient number the trial used previous data as reference point to establish futility. This is a world-wide acceptable strategy for phase II trials.

Reporting for specific materials, systems and methods

We require information from authors about some types of materials, experimental systems and methods used in many studies. Here, indicate whether each material, system or method listed is relevant to your study. If you are not sure if a list item applies to your research, read the appropriate section before selecting a response.

Materials & experimental systems

n/a	Involved in the study
☐	☒ Antibodies
☒	☐ Eukaryotic cell lines
☒	☐ Palaeontology and archaeology
☒	☐ Animals and other organisms
☐	☒ Clinical data
☒	☐ Dual use research of concern

Methods

n/a	Involved in the study
☒	☐ ChIP-seq
☒	☐ Flow cytometry
☒	☐ MRI-based neuroimaging

Antibodies

Antibodies used	PD-L1, MLH1, MSH6, PMS2, MSH2. WE monitored PD-L1 combined positive score and microsatelite instability through immunohistochemistry with these antibodies.
Validation	All antibodies used are commertialised and have been previously tested to ensure optimal recognition of their target. We have now provided the reference of each antibody within the methods section.

Clinical data

Policy information about clinical studies

All manuscripts should comply with the ICMJE guidelines for publication of clinical research and a completed CONSORT checklist must be included with all submissions.

Clinical trial registration	NCT03095274
Study protocol	Attached to the submission as supplementary material
Data collection	Dune include patients visited in 20 institutions in Spain.
Outcomes	The primary endpoint for Cohorts 1-3 was the 9-month (9-m) disease control rate (DCR) assessed by local investigators according to RECIST 1.1, defined as the factual percentage of patients achieving CR, partial response (PR), or SD at 9-m after the initiation of durvalumab plus tremelimumab treatment. The primary endpoint for Cohort 4 was 9-m overall survival (OS) rate, defined as the percentage of patients alive at 9-m after initiation of durvalumab plus tremelimumab therapy. The primary endpoint for each cohort was chosen based on the stipulated benchmarks 4-14 that reflected therapeutic success in the respective populations of heavily pre-treated patients with poor prognosis, and included long-lasting disease stabilization (Cohorts 1–3), and prolonged survival (Cohort 4). Secondary activity endpoints included: ORR, duration of response (DoR) defined as the time elapsed from the first response to PD; progression-free survival (PFS) according to irRECIST; and OS. Safety was based on the assessment of AEs, clinical laboratory test results, vital signs, and physical examination. AEs and laboratory values were graded according to the NCI-CTCAE v. 4.03. The trial included an exploratory analysis to correlate the expression of PD-L1 CPS and activity endpoints.
